# Catalytic Enantiodivergent Michael Addition by Subtle Adjustment of Achiral Amino Moiety of Dipeptide Phosphines

**DOI:** 10.1016/j.isci.2020.101138

**Published:** 2020-05-06

**Authors:** Huamin Wang, Xiuzheng Li, Youshao Tu, Junliang Zhang

**Affiliations:** 1 Department of Chemistry, Fudan University, 2005 Songhu Road, Shanghai 200438, P. R. China; 2 Shanghai Key Laboratory of Green Chemistry and Chemical Processes, School of Chemistry and Molecular Engineering, East China Normal University, 3663 N. Zhongshan Road, Shanghai 200062, P. R. China; 3 School of Pharmacy, Anhui Medical University, 81 N. Meishan Road, Hefei 230032, P. R.China; 4 College of Chemistry and Life Science, Advanced Institute of Materials Science, Changchun University of Technology, 2055 N. Yan'an Avenue, Changchun 130012, P. R. China

**Keywords:** Chemistry, Organic Chemistry, Organic Chemistry Methods

## Abstract

Over the past decades, asymmetric catalysis has been intensely investigated as a powerful tool for the preparation of numerous chiral biologically active compounds. However, developing general and practical strategies for preparation of both enantiomers of a chiral molecule via asymmetric catalysis is still a challenge, particularly when the two enantiomers of a chiral catalyst are not easily prepared from natural chiral sources. Inspired by the biologic system, we report herein an unprecedented catalytic enantiodivergent Michael addition of pyridazinones to enones by subtle adjustment of achiral amino moiety of dipeptide phosphine catalysts. These two dipeptide phosphine catalysts, **P5** and **P8**, could deliver both enantiomers of a series of *N*^*2*^-alkylpyridazinones in good yields (up to 99%) with high enantioselectivities (up to 99% ee) via the catalyst-controlled enantiodivergent addition of pyridazinones to enones.

## Highlights

• Enantiodivergent phosphine-catalyzed Michael addition
• Readily available starting materials, mild reaction conditions
• High efficiency, up to 99% yield and 99% ee
• General substrate scope

## Graphical Abstract

**Figure:**
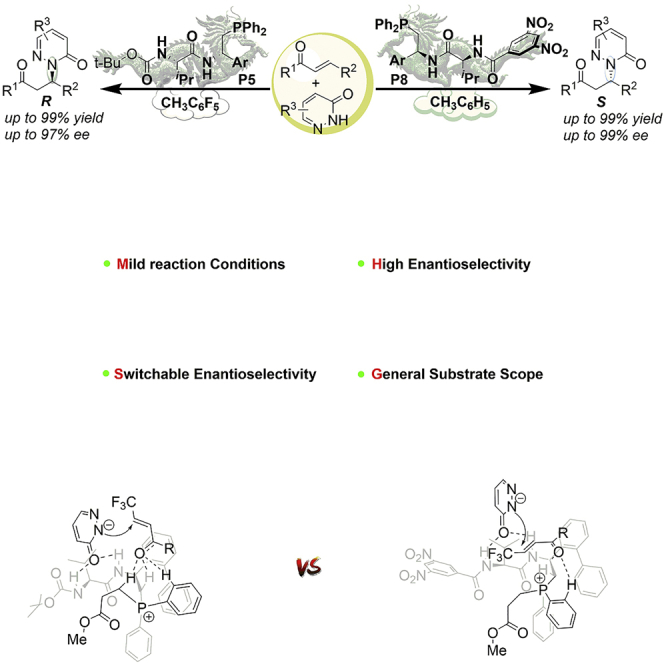

## Introduction

**Scheme 1:**
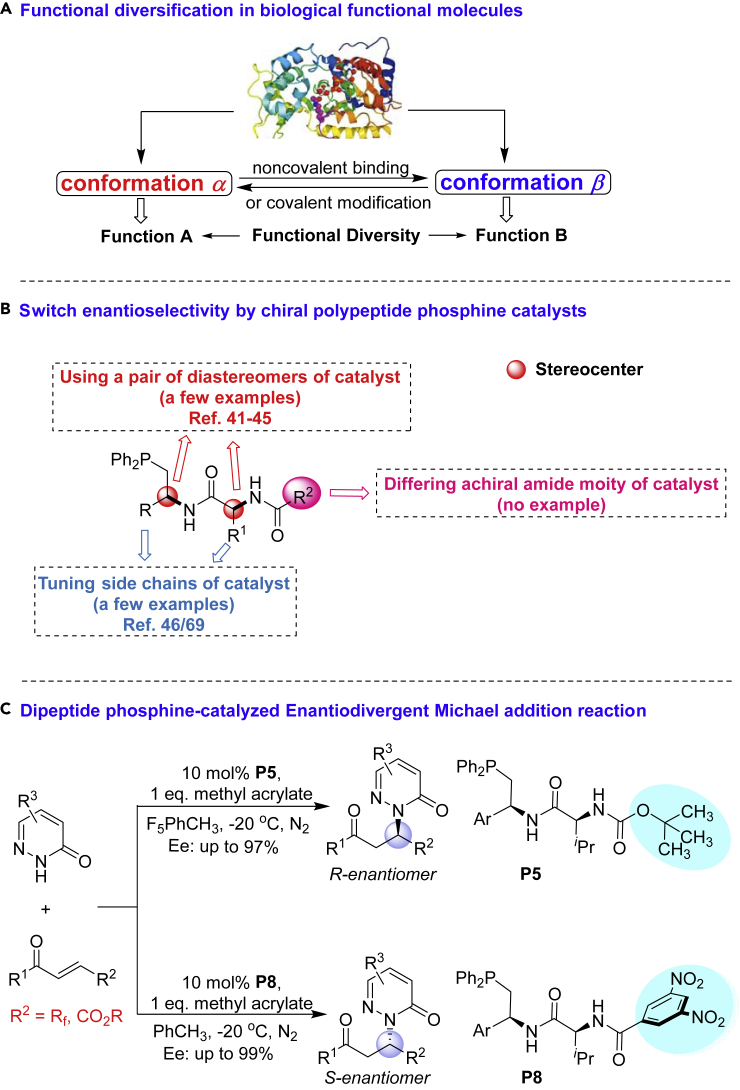
The Strategy for Switching of Enantioselectivity

The development of efficient methods to synthesize both enantiomers of a chiral molecule is of great significance, because drug candidates and their isomers may have distinct therapeutic properties or adverse effects (Wermuth, 2008, Jozwiak et al., 2012). Enantiodivergent methodology (Zanoni et al., 2003, Bartók, 2010, Beletskaya et al., 2018) is an attractive route to afford the mirror image products, which can be achieved with the use of both enantiomers of a chiral catalyst, respectively. However, the two enantiomers of the required chiral catalyst are not always available in nature. In biological systems, minor structural changes in functional molecules (proteins, enzymes, and hormones) by noncovalent binding of allosteric regulators or covalent modification of structure-determining functionalities (Li et al., 2012, Lyons et al., 2013, Lasalde et al., 2014) (e.g., cleavage of peptide domains, ionizable groups, and methylation/glycosylation/phosphorylation of H-bond donors) can display a polypeptide-based distinct three-dimensional architecture, leading to turn on/off their function or acquire another function, enabling the timely regulation of intra- or extracellular events with elegant synergy (Zanoni et al., 2003, Harrison, 2004, Heilmann et al., 2004, Nojiri et al., 2009) (Scheme 1A). For example, sickle cell anemia is an autosomal recessive genetic disease, caused by a single-base mutation in the beta gene of globin causing glutamate mutated to proline. This sickling leads to the RBC membrane damage and increases the likelihood of rupture and anemia (Gyang et al., 2011). Inspired by this intriguing biological process, we hypothesized that some small structural modifications in conformationally flexible chiral organocatalysts without changing any stereocenter might allow to obtain both stereoisomers in the individual form in asymmetric catalysis as well.

**Scheme 2:**
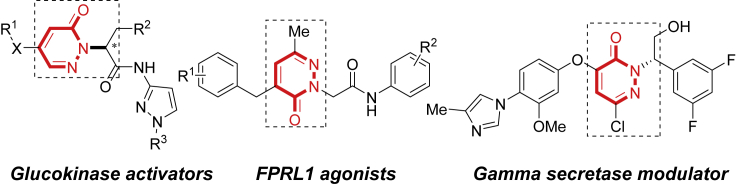
Bioactive Compounds Possessing a Chiral Pyridazinone Scaffold

Considerable research efforts have long been devoted to phosphine-catalyzed asymmetric reactions (Cai et al., 2016, Cowen and Miller, 2009, Fan and Kwon, 2013, Gu et al., 2015, Guo et al., 2018, Han et al., 2016, Lee et al., 2015, Li et al., 2015, Li et al., 2016, Li and Zhang, 2016, Lu et al., 2001, Ni et al., 2017, Ni et al., 2017, Ni et al., 2018, Sankar et al., 2016, Satpathi and Ramasastry, 2016, Wang et al., 2016b, Wang et al., 2018a, Wang et al., 2018b, Wang et al., 2016c, Wang et al., 2016a, Wang et al., 2014, Wei and Shi, 2010, Wei and Shi, 2017, Xie and Huang, 2015, Ye et al., 2008, Zhang et al., 2015, Zhao et al., 2012), whereas the enantiodivergent synthesis directed by chiral natural amine-acid-derived bi- or multifunctional phosphine still poses considerable challenge. Only a few examples of enantiodivergent phosphine-catalyzed reactions were realized so far (Henry et al., 2014, Wang et al., 2015a, Wang et al., 2015b, Wang et al., 2017a, Wang et al., 2017b, Wang et al., 2017c, Ni et al., 2016, Li et al., 2016, Gu et al., 2018, Smaligo et al., 2018) (Scheme 1B), in which the enantioselectivity could be only partially switched by variation of one or multiple stereocenters of phosphine catalysts. Early Lu group (Wang et al., 2015a, Wang et al., 2015b, Wang et al., 2017a, Wang et al., 2017b, Wang et al., 2017c, Ni et al., 2016) observed that the enatioselectivity of phosphine-catalyzed enantioselective γ-additions of allenoates could be moderately switched by a pair of diastereomers of the chiral catalyst. Kwon group (Henry et al., 2014, Smaligo et al., 2018) reported the enantiodivergent [3 + 2] annulations of allenoates and imines to obtain a series of pyrrolines via a pair of diastereomeric phosphine catalysts. To the best of our knowledge, in the area of phosphine catalysis, switching enantioselectivity to gain both enantiomers in high ee without changing any stereocenter of the phosphine catalyst has not been explored so far. Meanwhile, many efficient catalytic asymmetric reactions have been well established in recent decades; however, asymmetric phosphine-catalyzed Michael addition (Zhong et al., 2013, Huang et al., 2017) to non-terminal electron-deficient alkenes are much less developed and represent a challenging task. In view of the biological significance of N2-alkylated pyridazinones (Van der Mey et al., 2001, Berthel et al., 2009, Allerton et al., 2009, Rathish et al., 2009, Cilibrizzi et al., 2009, Ahmad et al., 2010, Parveen et al., 2017) (Scheme 2), herein, we report an enantiodivergent phosphine-catalyzed Michael addition of pyridazinones to enones, which provides a rapid access to two enantiomers of *N*^*2*^-alkylated pyridazinones in good to excellent enantioselectivity (Scheme 1C). The enantioselectivity was well switched by the subtle variation of the amide moiety of chiral dipeptide phosphine catalyst without changing any stereogenic element.

## Results and Discussion

### Research Design

During the course of our study on phosphine-catalyzed (Su et al., 2015, Zhou et al., 2015, Zhou et al., 2016a, Zhou et al., 2016b, Zhou et al., 2017, Chen et al., 2016, Chen and Zhang, 2017, Wang et al., 2017a, Wang et al., 2017b, Wang et al., 2017c, Wang et al., 2018a, Wang et al., 2018b, Wang et al., 2019, Huang et al., 2017, Zhang et al., 2017) diverse transformations of enones, we envisaged that the asymmetric organophosphorus zwitterion intermediate, generated *in situ* by mixing a chiral multifunctional phosphine with methyl acrylate, might provide a mild Brønsted base to activate pyridazinone. The subsequently formed ionic pair, followed by the addition to *β*-substituted enones was feasible.

**Scheme 3:**
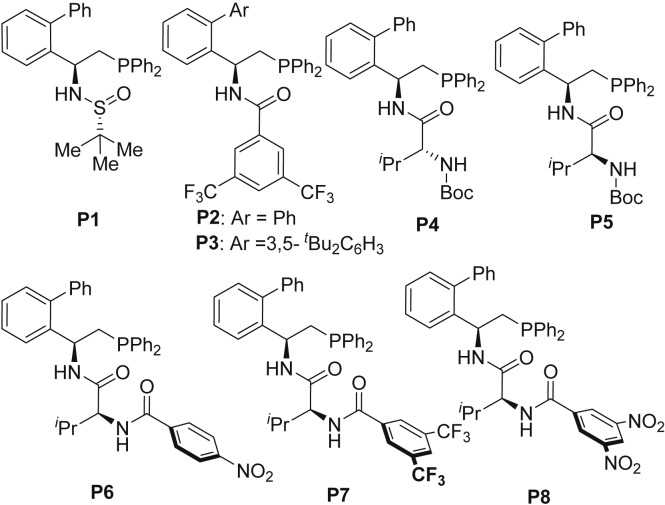
Phosphine Catalysts Employed in This Study

The reaction between *β*-trifluoromethylated enone **1f** and pyridazinone **2a** was investigated in the presence of chiral phosphine catalyst (Scheme 3) and methyl acrylate in DCM at room temperature (Table 1). The chiral sulfinamide phosphine **P1** developed by us (Su et al., 2015, Zhou et al., 2016a) is not efficient to deliver **(−)-3fa** in low yields along with recovery of **1f** (Table 1, entry 1). The variation of the *tert*-butanesulfinamide to 3,5-bis(trifluoromethyl)benzoyl-derived amide (Wang et al., 2017a, Wang et al., 2017b, Wang et al., 2017c, Zhou et al., 2017) could increase the catalytic activity significantly but only 16% ee was obtained (Table 1, entry 2). The Introduction of a bulkier *3*,*5-di-tert*-butylphenyl group at the ortho-position of the phenyl ring gave similar ee (Table 1, entry 3). Gratifyingly, the desired product was obtained in 98% yield with 31% ee upon the use of *N*-Boc-*D*-Val-derived phosphine **P4** (Table 1, entry 4). To our delight, its diastereomer *N*-Boc-*L*-Val-derived **P5** could substantially improve the ee (Table 1, entry 5). To our surprise, the replacement of Boc-amide (**P5**) with other benzoyl-derived amides (**P6–P8**) could reverse the enantioselectivity of the reaction to deliver the **(+)-3fa** as the major enantiomer (Table 1, entries 6–8), in which the catalyst **P8** showed promising result (57% ee). Further solvent screening showed toluene is the best solvent to deliver **(+)-3fa** in 81% ee (Table 1, entry 12). After further systematic screening, the enantiodivergent phosphine-catalyzed addition of pyridazinones to enone was realized by running the reaction at −20°C under the catalysis of **P5** in F_5_C_6_CH_3_ and **P8** in toluene, respectively (Table 1, entries 17–19). Lowering the amount of methyl acrylate from 1.0 to 0.5 equivalent would keep the enantioselectivity unchanged but deliver a relatively lower yield (Table 1, entry 20).

**Table 1:** Screening of Reaction Conditions


Entry	Cat.	Solvent	Yield (%)^a^	(+/−)-3fa, ee (%)^b^
1	**P1**	DCM	Trace	–
2	**P2**	DCM	88	(−)-**3fa**, 16
3	**P3**	DCM	90	(−)-**3fa**, 17
4	**P4**	DCM	98	(−)-**3fa**, 31
5	**P5**	DCM	96	(−)-**3fa**, 51
6	**P6**	DCM	99	(+)-**3fa**, 26
7	**P7**	DCM	99	(+)-**3fa**, 25
8	**P8**	DCM	99	(+)-**3fa**, 57
9	**P8**	CHCl_3_	81	(+)-**3fa**, 67
10	**P8**	THF	73	(+)-**3fa**, 62
11	**P8**	Et_2_O	95	(+)-**3fa**, 72
12	**P8**	Toluene	98	(+)-**3fa**, 81
13	**P8**	PhCF_3_	99	(+)-**3fa**, 73
14	**P8**	*o*-xylene	98	(+)-**3fa**, 80
15	**P8**	F_5_PhCH_3_	97	(+)-**3fa**, 79
16^c^	**P8**	Toluene	98	(+)-**3fa**, 94
17^d^	**P8**	Toluene	97	(+)-**3fa**, 98
18^d^	**P5**	Toluene	95	(−)-**3fa**, 86
19^d^	**P5**	F_5_PhCH_3_	98	(−)-**3fa**, 95
20^e^	**P8**	Toluene	90	(+)-**3fa**, 98

a NMR yield with CH_2_Br_2_ as an internal standard.

b Determined by HPLC analysis on a chiral stationary phase.

c The reaction was performed at −10^o^C and the reaction time was 2 h.

d The reaction was performed at −20^o^C and the reaction time was 3 h.

e 50mol% methyl acrylate was used.

### Scope of the Investigation

**Scheme 4:**
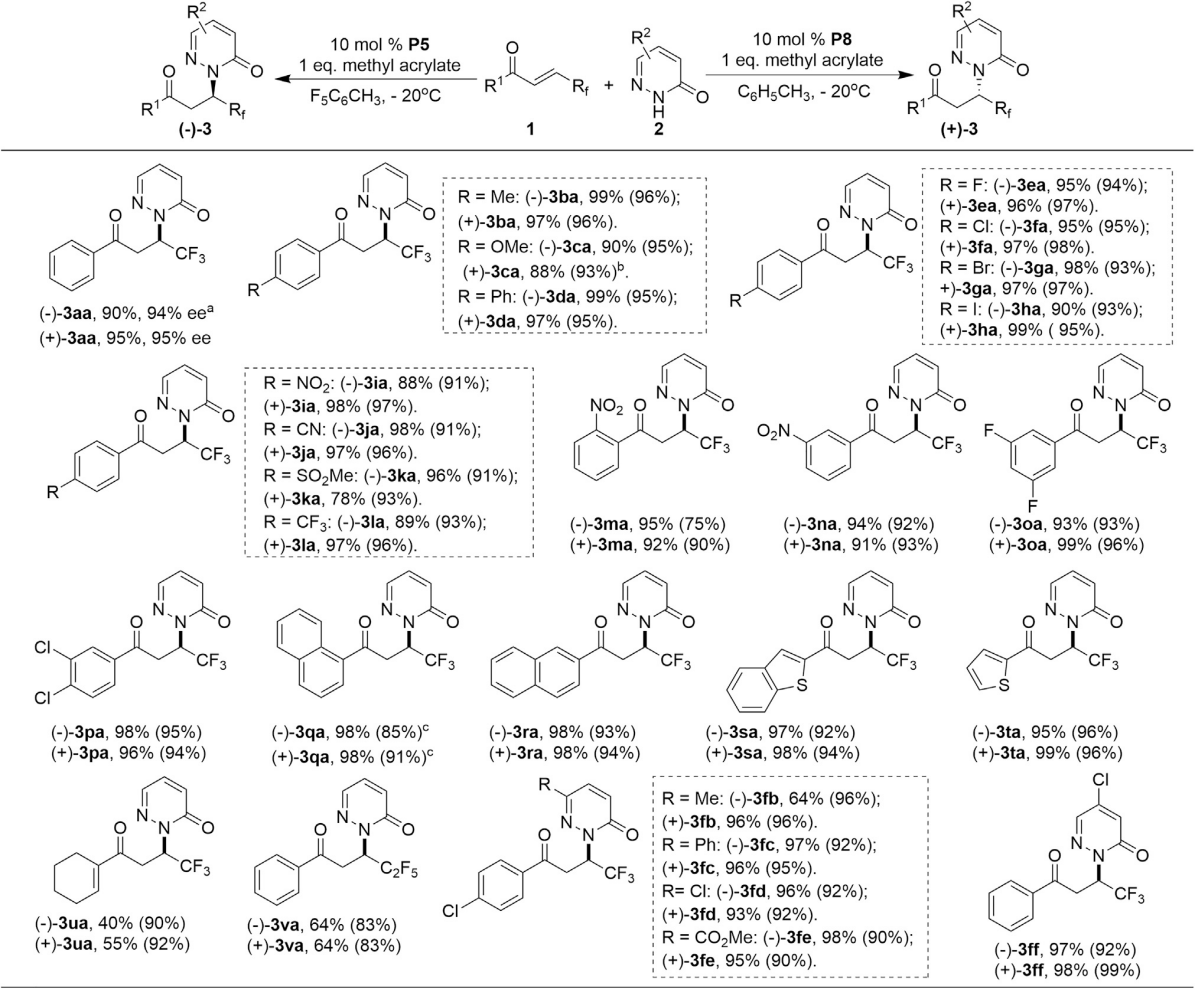
Substrate Study with Variation of *β*-Perfluoroalkyl-Substituted Enones **1** and Pyridazinones **2** ^a^Reactions were performed with **1** (0.1 mmol), **2** (0.2 mmol), methyl acrylate (0.1 mol); method A: **P5** (0.01 mmol) in F_5_PhCH_3_ (1.0 mL) at −20°C; method B: **P8** (0.01 mmol) in toluene (1.0 mL) at −20°C. Ee in parenthesis and determined by HPLC analysis on a chiral stationary phase. ^b^at −25°C. ^c^at −30°C.

The scope of this enantiodivergent hydroamination reaction was subsequently probed. Firstly, the scope of the enantioselective hydroamination reaction under the catalysis of **P8** in toluene was investigated (Scheme 4, Method B). Generally, *β-*trifluoromethyl enones with different substituents on the phenyl ring, regardless of the substitution patterns and electronic properties, afforded the corresponding products **(+)-3** in high yields with excellent ees (Scheme 4, **(+)-3aa-(+)-3pa**). The absolute configuration of **(+)-3da** was determined to be *S* by X-ray crystallographic analysis (see Supplemental Information) and the other products were analogously assigned. In addition, fused aromatic and hetero-aromatic group-substituted enones were also applicable to the reaction, delivering the desired hydroamination products in excellent yields (98%–99%) with 91%–-96% ee (Scheme 4, **(+)-3qa-(+)-3ta**). Enone **1u** with a cyclohexenyl substituent produced **(+)-3ua** in moderate yield with 92% ee (Scheme 4, Method B). Furthermore, the trifluoromethyl group could be replaced by perfluoroethyl, furnishing moderate yield of the desired product **(+)-3va** in 83% ee. Subsequently, the scope of the pyridazinone component **2** was investigated and all reactions proceeded well with no matter electron-donating or electron-withdrawing substituents (**2b-2f**) at different positions, providing **(+)-3fb-(+)-3ff** in 93%–98% yields with 90%–99% ees. Then, all the reactions mentioned above were then carried out under the catalysis of **P5** as the catalyst in CH_3_C_6_F_5_ at −20°C (Scheme 4). The scope of *β*-trifluoromethyl enone component is quite general, various aryl (**1a-1r**), heteroaryl (**1s-1t**), and cyclohexenyl (**1u**) substituents (Scheme 4, **(−)-3aa-(−)-3ua**) were compatible, delivering 75%–96% ees. What is more, *β*-pentafluoroethyl enone (**1v**) was also compatible to furnish good ee. Pyridazinones **2** with either electron-withdrawing or electron-donating substituents were also well tolerated delivering the desired products in good to excellent yields with excellent ees (**(−)-3fb-(−)-3ff**).

**Scheme 5:**
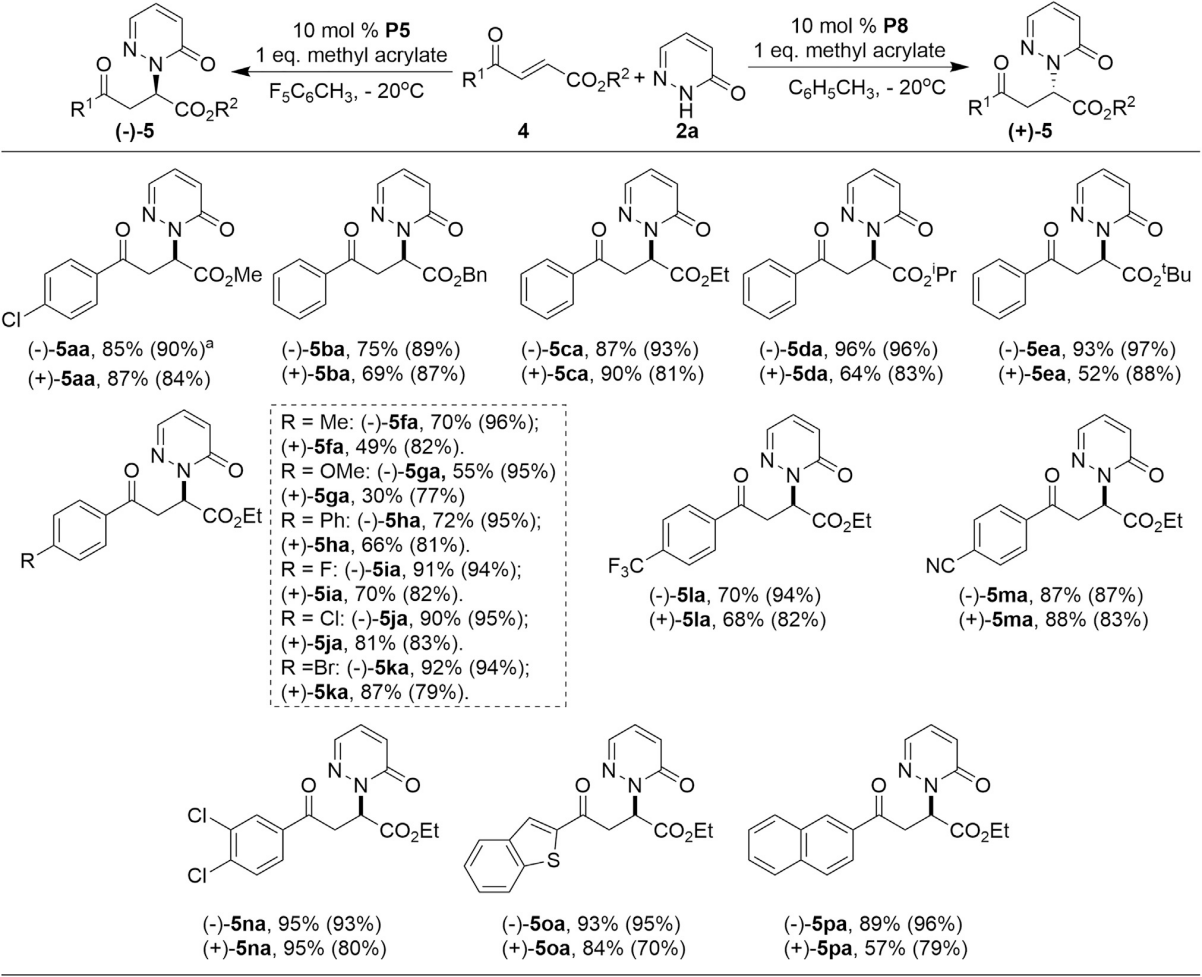
Substrate Study with Variation of 3-Aroyl Acrylates **4** and Pyridazinone **2a** ^a^Reactions were performed with **1** (0.1 mmol), **2** (0.2 mmol), methyl acrylate (0.1 mol); method A: **P5** (0.01 mmol) in F_5_PhCH_3_ (1.0 mL) at −20°C; method B: **P8** (0.01 mmol) in toluene (1.0 mL) at −20°C. Ee in parenthesis and determined by HPLC analysis on a chiral stationary phase.

The scope of 3-aroyl acrylates were then investigated (Scheme 5). In most cases, the desired products **(−)-5aa-(−)-5pa** were obtained in good yields with excellent enantioselectivity by using **P5** as the chiral catalyst (Method A). Substrates with various esters (**4a–4e**) and different aryl substituents (**4f–4p**) were all compatible, furnishing the corresponding products in 55%–97% yields and 87%–97% ees (**(−)-5aa-(−)-5pa**). Meanwhile, the reaction proceeded also well to afford the desired products **(+)-5aa-(+)-5pa** under the catalysis of **P8** (Method B). However, the reaction was found to be somewhat sensitive to the electronic nature of the substituents on the aromatic ring. Electron-donating substituents (**(+)-5fa-(+)-5ha**) led to the desired products in relatively lower yield compared with electron-withdrawing substituents (**(+)-5ia-(+)-5na**). The reaction of heteroaryl- (**4o**) and naphthyl- (**4p**) containing substrates proceeded smoothly to give the corresponding products in 57%–84% yields but with relatively lower enantioselectivities (**(+)-5oa-(+)-5pa**).

**Scheme 6:**
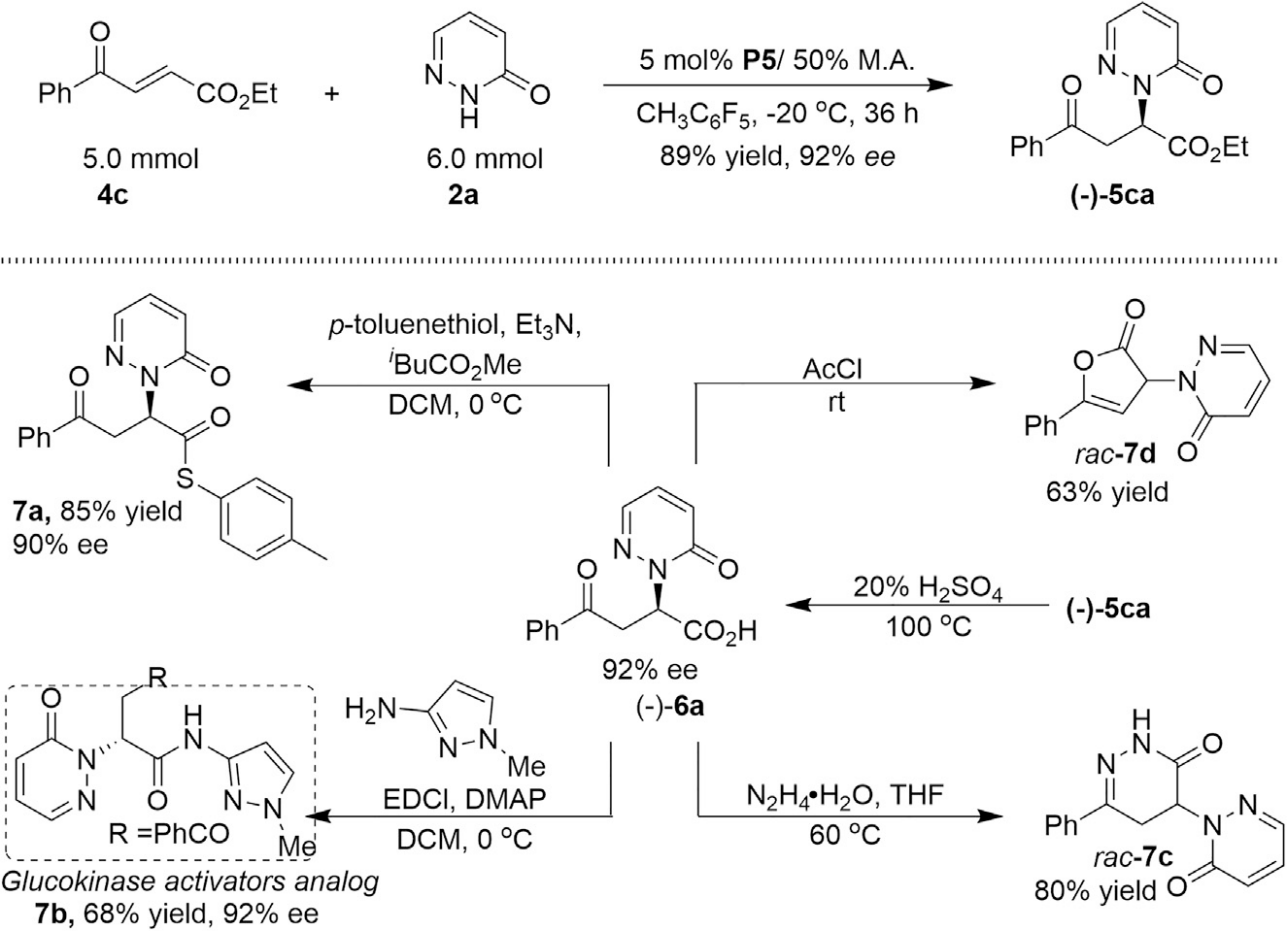
Scaled-Up Version of the Michael Addition and Transformation of the Products

To evaluate two chiral dipeptide phosphine catalytic systems on a large scale, 5.0 mmol of *β*-trifluoromethylated enone **1f** and 3-aroyl acrylate **4c** was used to perform the Michael addition reaction, providing the corresponding product **(+)-3fg** and **(−)-5ca** with excellent yields in 95% and 92% ees. The **(−)-5ca** could be hydrolyzed under acidic conditions, affording product **(−)-6a** in 95% yield with 92% ee. The thioester **7a** and glucokinase activators analog (Berthel et al., 2009, Allerton et al., 2009, Rathish et al., 2009) amide **7b** could be obtained in 85% and 68% yield, respectively from the compound **(−)-6a**. Racemic pyridazinone **7c** and lactone **7d** were both obtained in good yield by treating **(−)-6a** with either hydrazine hydrate in THF or acetyl chloride, respectively (Scheme 6).

### Mechanistic Study

**Scheme 7:**
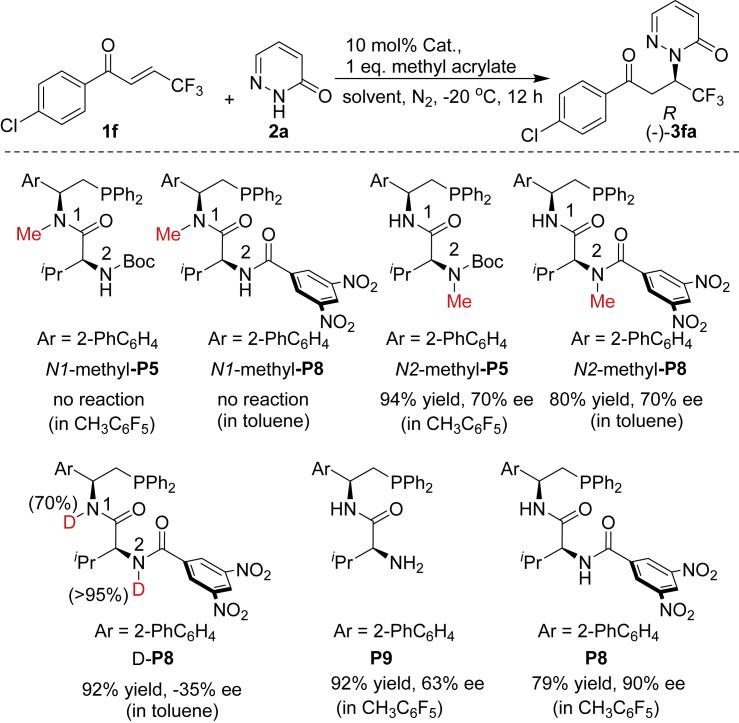
Control Experiments

To gain insight of the role of these two hydrogen-bonding interactions, N1-methyl-**P5**, N1-methyl-**P8**, N2-methyl-**P5**, N2-methyl-**P8**, deuterated **P8**, and **P9** with free terminal amine were then synthesized and subjected to the reaction, respectively (Scheme 7). It is interesting to find that N1-methyl-**P5** and N1-methyl-**P8** could not catalyze the reaction, indicating that the first N1-H is crucial to the catalytic activity. In addition, both N2-methyl-**P5** and N2-methyl-**P8** gave **(−)-3fa** in satisfactory yields with 70% ee. More interestingly, the deuterated catalyst **P8** could deliver **(+)-3fa** in 92% yield but with much lower enantioselectivity. Catalyst **P9** also gave **(−)-3fa** in satisfactory yields with 63% ee. Together, these observations clearly indicated that the second N2-H of **P8** is crucial to reverse the enantioselectivity. Subsequently, we wondered whether the stereoselectivities were enhanced by using the pentafluoro toluene. When **1f** and **2a** were carried out in CH_3_C_6_F_5_, the product **(+)-3fa** was obtained in 79% yield and slightly lower enantioselectivity (90% ee) compared with toluene (98% ee) as solvent. Simultaneously, we then conducted NMR titration experiments (see the SupplementalInformation for details) and observed that hydrogen bond interaction did not exist between pentafluoro toluene and pyridazinone or catalyst, implying the enantioselectivity was not significantly influenced by fluorinated solvent.

### Conclusion

In conclusion, we have developed two new chiral dipeptide phosphine catalysts, which showed good performance in enantioselective addition of pyridazinones with enones. The enantioselectivity could be switched by subtle variation of the amino moiety of chiral dipeptide phosphine catalyst without changing any stereocenter of the phosphine catalyst. Both enantiomers of *N*^*2*^-alkylated pyridazinones can be obtained in high yields (up to 99%) with good to excellent enantioselectivity (up to 99% ee) by the use of **P5** and **P8**, respectively. The results of control experiments suggest that a number of hydrogen-bonding interactions play a crucial role in determining the catalytic activity and enantioselectivity reversal (see the Supplemental Information for proposed transition states). The salient features of this work include readily available starting materials, mild reaction conditions, high efficiency, switchable enantioselectivity, and general substrate scope. Extensions of this concept with other important organic transformations and comprehensive theoretical studies into the reaction mechanism will also be reported in due course.

### Limitations of the Study

A brief examination showed that the present method is not compatible with chalcone and (E)-(2-nitrovinyl)benzene for the construction of corresponding *N*^*2*^-alkylated pyridazinones.

### Resource Availability

#### Lead Contact

Further information and requests for resources should be directed to and will be fulfilled by the Lead Contact, J. Zhang (junliangzhang@fudan.edu.cn).

#### Materials Availability

This study generated new unique reagents, include phosphine catalysts and *N*^*2*^-alkylated pyridazinones.

#### Data and Code Availability

The data for the X-ray crystallographic structure of (+)-**3da** has been deposited in the Cambridge Crystallographic DataCenter under accession numbers CCDC: 1839409.

## Methods

All methods can be found in the accompanying Transparent Methods supplemental file.

## Supplemental Information

Document S1. Transparent Methods, Figures S1–S4, Tables S1–S4, and Data S1 and S2

## Footnotes

Supplemental Information can be found online at https://doi.org/10.1016/j.isci.2020.101138.
