# Supplementary material for: Catalytic Enantiodivergent Michael Addition by Subtle Adjustment of Achiral Amino Moiety of Dipeptide Phosphines
Source: iScience. 2020 May 6;23(6):101138. doi: 10.1016/j.isci.2020.101138 (PMC7251764; doi:10.1016/j.isci.2020.101138)
Supplement: Document S1. Transparent Methods, Figures S1–S4, Tables S1–S4, and Data S1 and S2 [file mmc1.pdf]

iScience, Volume *23*

Supplemental Information

Catalytic Enantiodivergent Michael Addition by Subtle Adjustment of Achiral Amino Moiety of Dipeptide Phosphines

Huamin Wang, Xiuzheng Li, Youshao Tu, and Junliang Zhang

Transparent Methods

A. General Information

Unless otherwise noted, all reactions were carried out under a nitrogen atmosphere; materials obtained from commercial suppliers were used directly without further purification. The $[\alpha]_D$ was recorded using PolAAr 3005 High Accuracy Polarimeter. ^{1}H NMR spectra, ^{13}C NMR spectra, ^{31}P NMR spectra and ^{19}F NMR spectra were recorded on a Bruker 400 (300 or 500) MHz spectrometer in chloroform-d_3. Chemical shifts (in ppm) were referenced to tetramethylsilane (δ = 0 ppm) in $CDCl_3$ as an internal standard. ^{13}C NMR spectra were obtained by using the same NMR spectrometers and were calibrated with $CDCl_3$ (δ = 77.00 ppm). The data is being reported as (s = singlet, d = doublet, dd = doublet of doublet, t = triplet, m = multiplet or unresolved, br = broad signal, coupling constant(s) in Hz, integration). Noteworthy, splitting signals between ^{13}C nucleus and ^{13}P nucleus in some chiral phosphine catalysts were difficult to distinguish and these ^{13}C NMR signals were reported as singlet entirely.

Trichloromethane ($CHCl_3$), dichloromethane, dichloroethane and ethyl acetate were freshly distilled from CaH_2; tetrahydrofuran (THF), toluene and ether were dried with sodium benzophenone and distilled before use. Reactions were monitored by thin layer chromatography (TLC) using silicycle pre-coated silica gel plates. Flash column chromatography was performed on silica gel 60 (particle size 200-400 mesh ASTM, purchased from Yantai, China) and eluted with petroleum ether/ethyl acetate. The Substrates **1,** (Yamazaki et al., 2009; Daniel et al., 2013) and catalysts **P1-P9 and P11** were synthesized according to the reported methods. (Su et al., 2015; Zhou et al., 2015; Zhou et al., 2016; Chen et al., 2016; Wang et al, 2015; Wang et al, 2017) All reagents and solvents were used as received from commercial sources (*Energy Chemical, Adamas-beta*®) without further purification.

B. Experimental procedures

Typical Synthetic Procedure and Datas for Novel Chiral Phosphines Catalyst **P1-P8**.

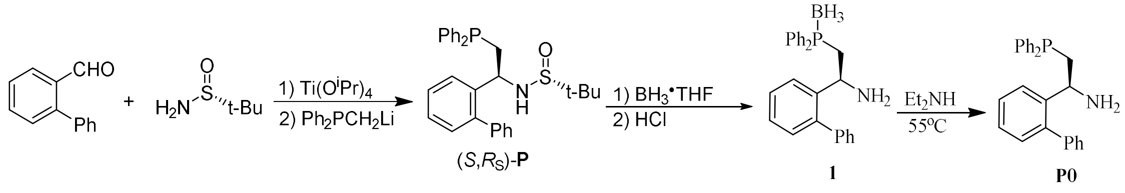

Step 1: to a flask containing a solution of [1,1'-biphenyl]-2-carbaldehyde (4.0 mmol) and *tert*-butylsulfinamide (6.0 mmol) was added $Ti(O^iPr)_4$ (8 mmol) and the mixture was stirred at 50ºC. Upon reaction completion, the reaction mixture was allowed to cool to room temperature, diluted with EtOAc, and poured to brine with rapid stirring. The resulting suspension was filtered through celite and washed with EtOAc. The combined organic phases were dried over $MgSO_4$ and the solvents were removed in vacuo. The residue was purified by silica gel chromatography using petroleum ether/EtOAc as the eluent to afford the desired chiral sulfinyl imines, isolated yield: 89%.

Step 2: A solution of diphenyl methyl phosphonic lithium (1.5 mmol) that containing TMEDA (1.5 mmol) in anhydrous THF was added to the solution of corresponding chiral sulfinyl imines (1.5 mmol chiral sulfinyl imines in 5 mL anhydrous THF) at room temperature. The mixture was stirred until completion of imine as indicated by TLC, followed by hydrolysis with 10 mL of water and diluted with EtOAc. The organic layer was separated, the aqueous phase was extracted three times with EtOAc (3X10 mL). The combined organic phases were dried over MgSO4 and the solvents were removed in vacuo. The residue was purified by silica gel chromatography using petroleum ether/EtOAc as the eluent to afford the desired ***(S,R_S)-P***, isolated yield: 51%, 5:1 *dr*.

Step 3: BH_3•THF (3.0 mmol) was added slowly to the solution of (*S*,*R_S*)-**P** (1.0 mmol) in dry THF at -30ºC and the reaction mixture was stirred for 2 h until completion of the material as indicated by TLC followed by adding 10 mL of water and 20 mL EtOAc. The aqueous phase was separated and extracted three times with 20 mL EtOAc. The combined organic phases were dried over $MgSO_4$ and the solvents were removed in vacuo.

Step 4: 6 M HCl (1 mL) was added to the above residue which disolved in MeOH (10 mL) and the reaction mixture was stirred at room temperature for 3 h until completion of material as indicated by TLC analysis, followed by washing with aq $NaHCO_3$ and 10 mL aq brine water. The organic layers was separated and extracted three times with 20 mL EtOAc. The combined organic phases were dried over $MgSO_4$ and the solvents were removed in vacuo.

Step 5: Et_2NH (5.0 mL) was added to the above residue and the mixture was stirred at 55°C for 6 h under the protection of N_2 until completion of material as indicated by TLC analysis. The solvent was then removed in vacuo and the resulting mixture **P0** was used directly for the next step.

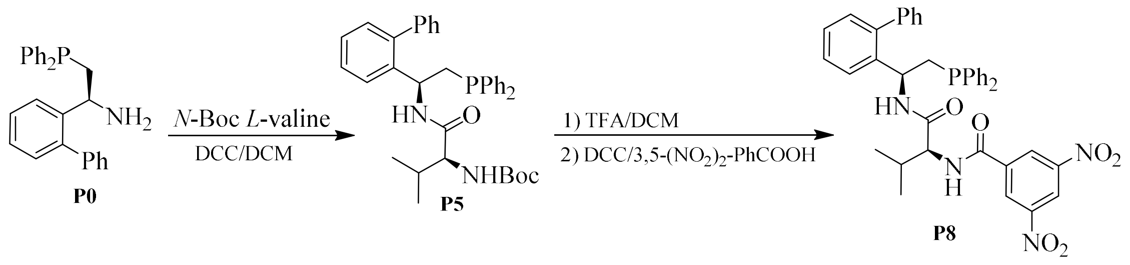

Step 1: To a stirred solution of *N*-Boc *L*-valine (434.6 mg, 2.0 mmol) in anhydrous CH_2Cl_2 (10 mL) was added DCC (226.8 mg, 1.1 mmol), and the resulting mixture was stirred at room temperature for 2 h. The solution was then cooled down to 0°C and the above residue and the mixture in CH_2Cl_2 (5 mL) was added dropwise over 2 minutes. The reaction mixture was further stirred for 1.0 h at 0°C and 1.0 h at room temperature. Water (10 mL) was added to quench the reaction, and the resulting mixture was extracted with dichloromethane several times (3 x 10 mL). The combined organic extracts were dried over sodium sulfate, filtered and concentrated, the residue was purified by column chromatography (hexane: ethyl acetate = 20:1) to afford **P5** (480 mg, 82%) as a white solid.

Step 2: To a stirred solution of **P5** (116 mg, 0.2 mmol) in anhydrous CH_2Cl_2 (2 mL) at room temperature was added TFA (0.4 mL), and the resulting mixture was stirred for 2 h. The reaction was then quenched with saturated aqueous $NaHCO_3$ (10 mL), and extracted with CH_2Cl_2 several times (3 × 10 mL). The combined organic extracts

were washed by brine (15 mL), and dried over Na_2SO_4, filtered and concentrated. The next operation is similar to above method which it afford **P8** (100 mg, 78%) as a yellow solid.

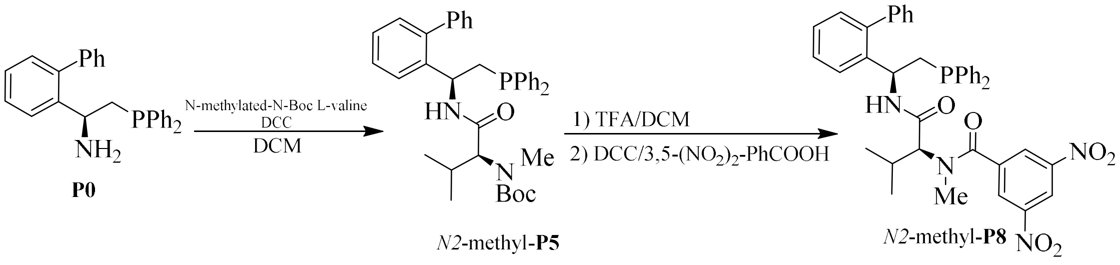

N2-methyl-**P5** were prepared according to the modified procedure of **P5**. To a stirred solution of *N2*-methyl-**P5** (180 mg, 0.3 mmol) in anhydrous CH_2Cl_2 (5 mL) at room temperature was added TFA (0.8 mL), and the resulting mixture was stirred for 2 h. The reaction was then quenched withsaturated aqueous $NaHCO_3$ (10 mL), and extracted with CH_2Cl_2 several times (3 × 10 mL). The combined organic extracts were washed by brine (15 mL), and dried over Na_2SO_4, filtered and concentrated. The next operation is similar to above method which it afford *N2*-methyl-**P8** (157 mg, 76%) as a light yellow solid.

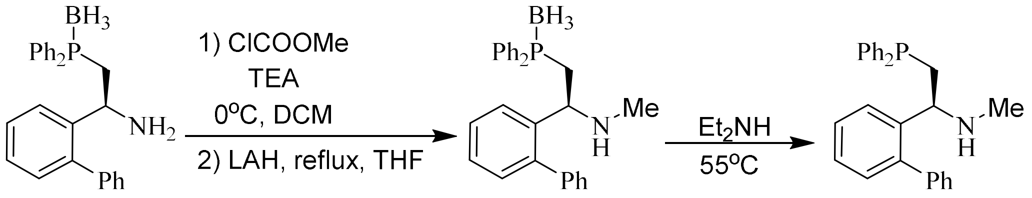

1 *N1*-methyl-**P0**

Step 1: To solution of amino phosphine **1** (3 mmol) and Et_3N (6.0 mmol) in dry CH_2Cl_2 (10 mL) at 0°C was added slowly ClCOOMe (4.5 mmol), and the resulting mixture was stirred at room temperature for 2h. Water (10 mL) was added and the organic layer was separated. The aqueous phase was extracted with CH_2Cl_2 (2 x 10 mL). The combined organic layers were washed with brine and dried over Na_2SO_4. Solvent was removed under reduced pressure, and the residue was used directly for the next step. To the solution of the carbamate intermediate in dry THF (10 mL) at 0 °C was added slowly LAH in THF (12 mmol), and the resulting mixture was refluxed

for 72 h. After cooling down to room temperature and further to 0ºC, the reaction mixture was quenched by addition of water and NaOH (1 M) solution. The insoluble slurry was filtrated off and washed with ethyl acetate. The filtrate was collected and the organic phase was separated. The aqueous layer was extracted with ethyl acetate (3 x 30 mL) several times, and the combined organic layers were washed with brine and dried over Na_2SO_4. Solvent was removed under reduced pressure, and the residue was used directly for the next step.

Step 2: Et_2NH (10.0 mL) was added to the above residue and the mixture was stirred at 55ºC for 6 h under the protection of N_2 until completion of material as indicated by TLC analysis. The solvent was then removed in vacuo and the resulting mixture *N1*-methyl-**P0** was used directly for the next step.

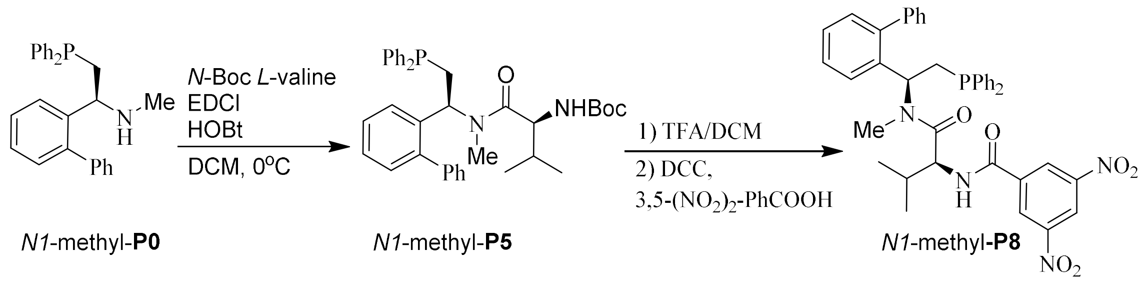

Step 1: To a solution of *N*-Boc-*L*-valine (3 mmol) in dry CH_2Cl_2 (10 mL) at 0 ºC under N_2 was added HOBt (3.6 mmol), *N,N*-diisopropylethylamine (3.6 mmol) and EDCl (3.6 mmol). After stirring for 10 min, crude product *N1*-methyl-**P0** in dry CH_2Cl_2 (10 mL) was introduced at the same temperature. The stirring was continued at 0ºC for 1 h and then at room temperature overnight. The mixture was diluted with CH_2Cl_2, washed with saturated aqueous NH_4Cl solution, and the organic layer was dried over Na_2SO_4. Solvent was removed under reduced pressure, and the residue was purified by column chromatography on silica gel (petroleum ether/EtOAc = 5/1) to afford *N1*-methyl-**P5** as a white solid (800 mg, 44% yield for three steps).

Step 2: To a stirred solution of *N1*-methyl-**P5** (0.3 mmol) in anhydrous CH_2Cl_2 (5 mL) at room temperature was added TFA (0.8 mL), and the resulting mixture was stirred for 2 h. The reaction was then quenched withsaturated aqueous $NaHCO_3$ (10 mL), and extracted with CH_2Cl_2 several times (3 × 10 mL). The combined organic

extracts were washed by brine (15 mL), and dried over Na_2SO_4, filtered and concentrated. The next operation is similar to above method which it afford *N1*-methyl-**P8** (125 mg, 60%) as a light yellow solid.

Typical Procedure for the Hydroamination Reactions, Related to Schem 4 and Scheme 5.

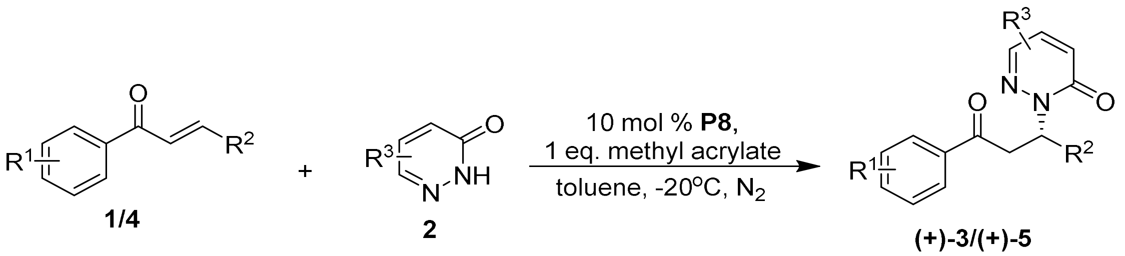

To a flame dried reaction tube with a magnetic stirring bar under N_2 at room temperature were added **P8** (0.01 mmol), pyridazinone **2** (0.2 mmol) and methyl acrylate (100 mol %), followed by the addition of anhydrous toluene (1.0 mL), and the mixture was stirred at -20°C for 10 min before the enones **1** / **4** (0.10 mmol) was added. When the reaction was finished (determined by TLC analysis), the crude mixture was purified by column chromatography on silica gel to afford the products (+)-**3**/(+)-**5**.

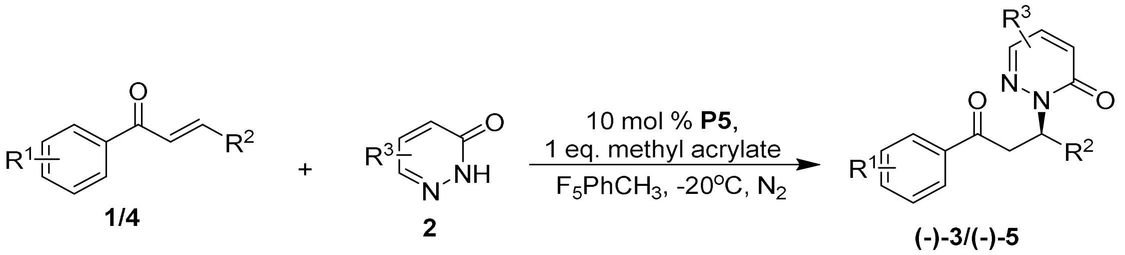

To a flame dried reaction tube with a magnetic stirring bar under N_2 at room temperature were added **P5** (0.01 mmol), pyridazinone **2** (0.2 mmol) and methyl acrylate (100 mol %), followed by the addition of pentafluoromethylbenzene (1.0 mL), and the mixture was stirred at -20°C for 10 min before the enones **1/4** (0.10 mmol) was added. When the reaction was finished (determined by TLC analysis), the crude mixture was purified by column chromatography on silica gel to afford the products

(-)-3/(-)-5.

Scaled-up Version of the Michael addition and Trans-formation of the Products, Related to Scheme 6

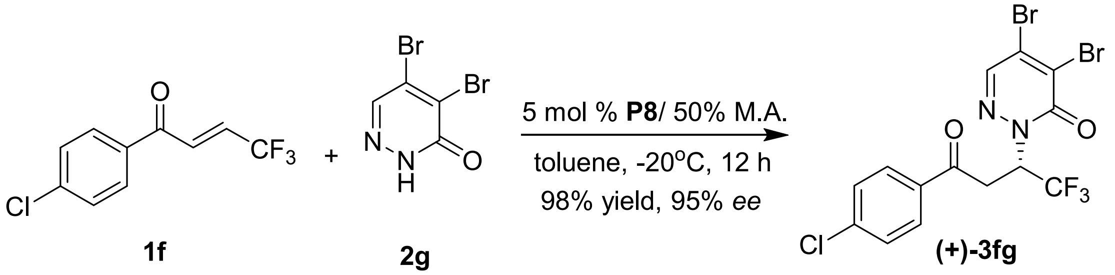

To a flame dried reaction tube with a magnetic stirring bar under N_2 at room temperature were added **P8** (0.25 mmol), 4,5-dibromopyridazin-3(2H)-one **2g** (6 mmol) and methyl acrylate (50 mol%), followed by the addition of anhydrous toluene (20.0 mL), and the mixture was stirred at -20°C for 10 min before the enones **1f** (5 mmol) was added. When the reaction was finished (determined by TLC analysis), the crude mixture was purified by column chromatography on silica gel to afford the product (+)-**3fg**, 2.4 g, 95% ee.

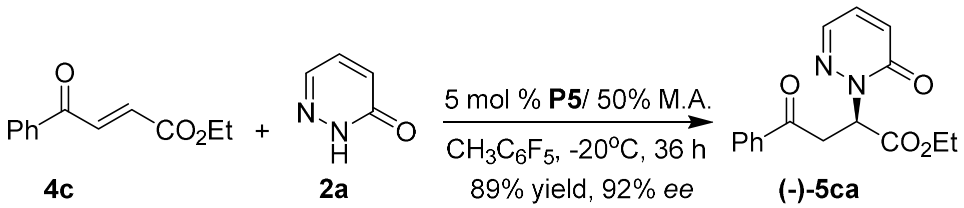

To a flame dried reaction tube with a magnetic stirring bar under N_2 at room temperature were added **P5** (0.25 mmol), pyridazinone **2a** (6 mmol) and methyl acrylate (50 mol %), followed by the addition of anhydrous 1,2,3,4,5-pentafluoro-6-methylbenzene (20.0 mL), and the mixture was stirred at -20°C for 10 min before the enones **4c** (5 mmol) was added. When the reaction was finished (determined by TLC analysis), the crude mixture was purified by column chromatography on silica gel to afford the product (-)-**5ca**, 1.3 g, 92% ee.

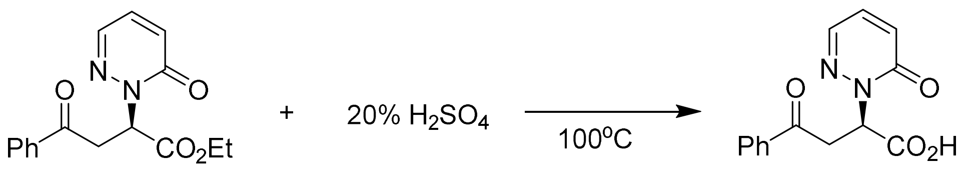

(-)-**5ca** (-)-6**a**

A mixture of (-)-**5ca** (2 mmol) and 20%H_2SO_4 (0.125 M, 16 mL) was heated at 100°C for 10 h and monitored by TLC. The reaction mixturewas poured onto ice/water with vigorously stirring and extracted with EA several times (3 × 10 mL). The combined organic extracts were washed by brine (15 mL), and dried over Na_2SO_4, filtered and concentrated. The crude mixture was purified by column chromatography on silica gel to afford the product (-)-**6a**, 517.4 mg, 95% yield, 92% ee.

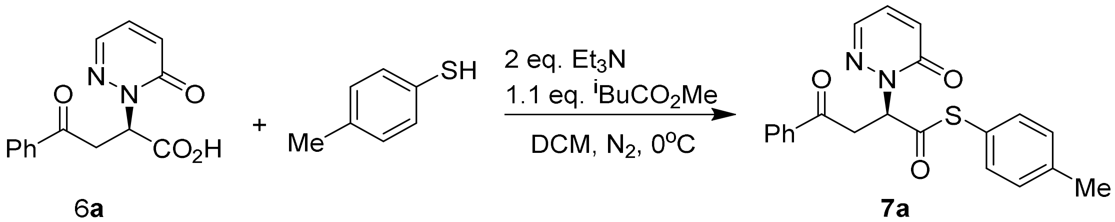

6**a** **7a**

A flame-dried flask was charged with **6a** (0.1 mmol, 1 equiv) and CH_2Cl_2 (1 mL). The reaction was cooled to 0°C and isobutyl chloroformate (0.11 mmol, 1.1 equiv) and Et_3N (0.1 mmol, 1 equiv) were added dropwise. The resulting mixture was stirred vigorously for 10 min under N_2, after which time Et_3N (0.1 mmol, 1 equiv) and thiophenol (0.22 mmol, 2.2 equiv) were added dropwise. The reaction was stirred at 0°C under N_2 for 1 h. The reaction was warmed to room temperature and washed with water, water, and brine. The combined aqueous layers were extracted with CH_2Cl_2. The combined organic layers were dried ($MgSO_4$), filtered, and concentrated. The crude residue was purified by column chromatography (PE/EA = 2/1) to afford the product **7a**, 32.2 mg, 85% yield, 90% ee.

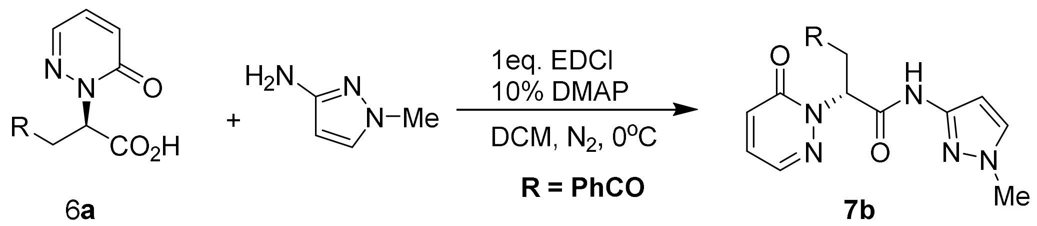

Add 1-ethyl-3-(3-dimethylaminopropyl) carbodiimide hydrochloride (1 equivalent), **6a** (0.1 mmol, 1 equivalent) and 4-dimethyl-aminopyridine (0.1 equivalent) to a stirred solution of 1-methyl-*1H*-pyrazol-3-amine (0.1 mmol, 1 equivalent) in methylene chloride at 0°C. Stir the reaction mixture at this temperature for 2 hours, during 2 hours the solution becomes homogeneous. After completion (TLC control using EA as eluent), wash the reaction mixture with water and brine. Dry the organic layer with Na_2SO_4. The crude residue was purified by column chromatography (EA) to afford the product **7b**, 23.9 mg, 68% yield, 93% ee.

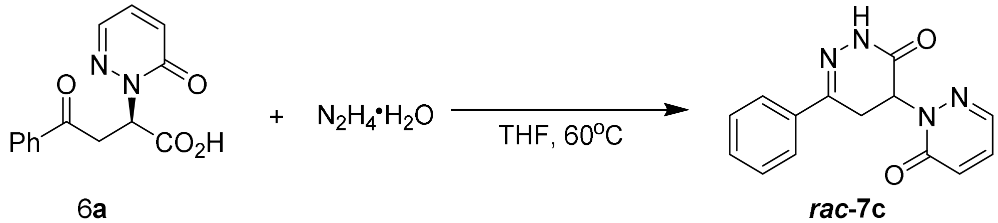

To the **6a** (0.1 mmol, 1 eq.) in THF (1 mL) was added $NHNH_2$•H_2O (0.2 mmol). The resulting mixture was stirred at 60°C for 1 h. The fltrate was concentrated to dryness under reduced pressure and the crude residue was then diluted in 1 M HCl and extracted with CH_2Cl_2. The organic layer was washed with water, dried over $MgSO_4$ and evaporated to dryness under reduced pressure. The crude residue was purified by column chromatography (PE/EA = 1/1) to afford the product *rac*-**7c**, 21.4 mg, 80% yield.

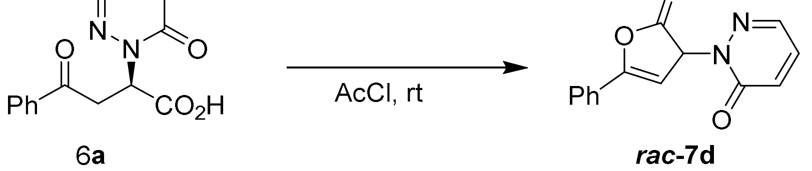

6a (0.1 mol), acetyl chloride (1 mL) was added and the mixture was stirred at room

temperature for 1 h. The acetyl chloride excess was removed in vacuo. The crude mixture was add H_2O (2 mL) to the reaction mixture and extract the organic layer with EtOAc (5 mL × 3). Evaporate the combined organic phases under reduced pressure. The crude residue was purified by column chromatography (PE/EA = 1/1) to afford the product *rac*-**7d**, 16.0 mg, 63% yield.

Synthesis of **d-P5** and **d-P8**, Related to Scheme 7.

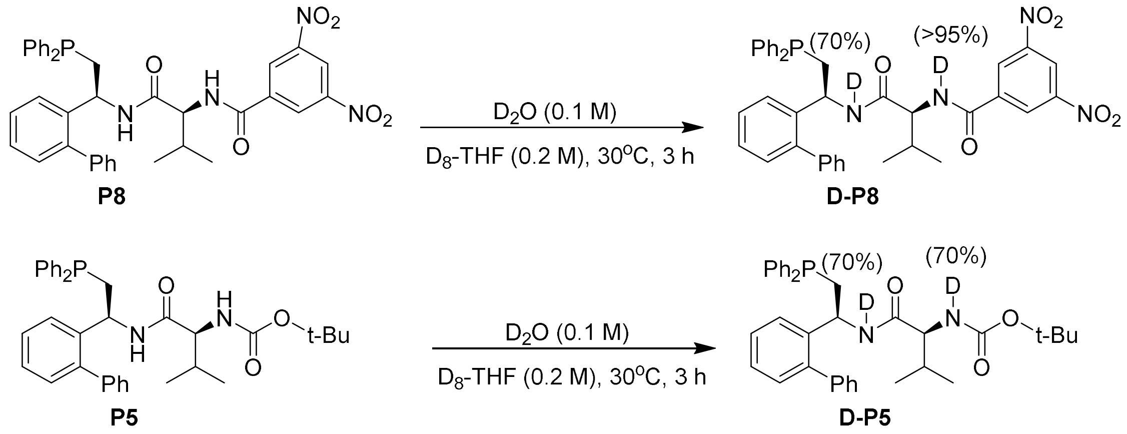

A flame-dried round bottom flask equipped with a magnetic stir bar under N_2 was charged with **P5** or **P8** (0.2 mmol) and d_8-THF (1.0 mL), followed by the addition of D_2O (2.0 mL). The reaction was then heated to 30°C for three hours. The reaction was then diluted with dry dichloromethane (5 mL), filtered through diatomite, dried over sodium sulfate and concentrated. ^1H NMR spectra was recorded on a Bruker300 (or 400) MHz spectrometer in DMSO-d_6.

Tables and Figures

Table S1. Asymmetric Michael Addition of pyridazinones to enones catalyzed by different chiral phosphines.[a] Related to Table 1.

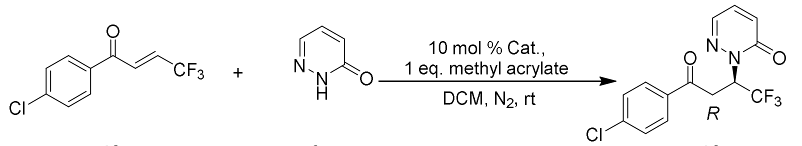

1f **2a** (-)-**3fa**

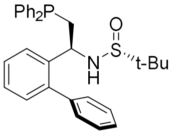

P1
trace

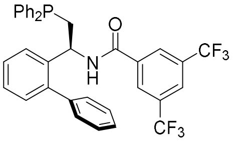

P2
88% yield, 16% ee

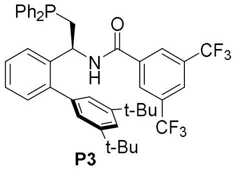

P3
90% yield, 17% ee

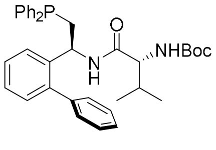

P4
90% yield, 31% ee

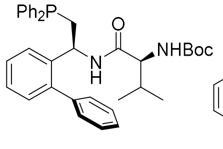

P5
98% yield, 51% ee

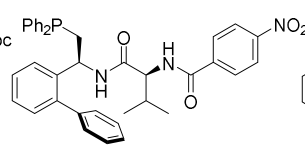

P6
96% yield, -26% ee

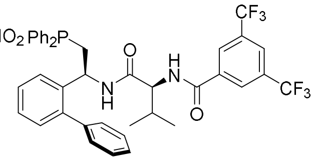

P7
99% yield, -25% ee

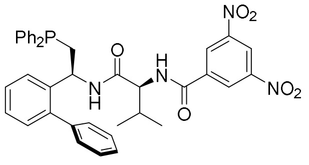

P8
99% yield, -57% ee

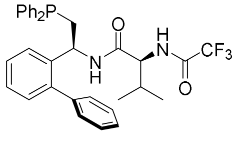

P10
85% yield, -2.4% ee

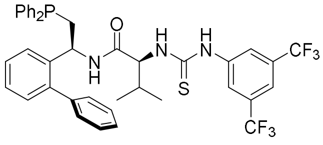

P11
97% yield, -47% ee

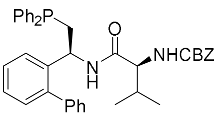

P12
97% yield, 40% ee

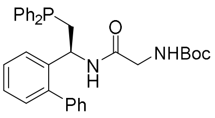

P13
93% yield, 21% ee

[a] Reaction conditions: **1f** (0.1 mmol), **2a** (0.2 mmol), methyl acrylate (0.1 mmol) and catlyst (0.01 mmol) in DCM (1 mL) at room temperature for 1 h. NMR yield with CH_2Br_2 as an internal standard. Determined by HPLC analysis on a chiral stationary phase.

Table S2. Optimization of Reaction Conditions Using Model Substrates.[a] Related to Table 1.

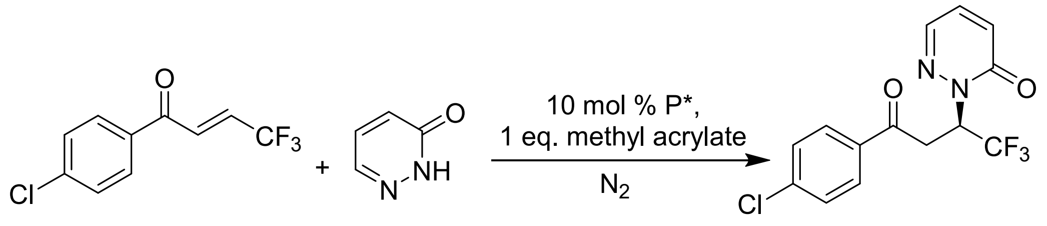

Entry	**Cat**	Temp. (^oC)	Solvent	Yield[b] (%)	Ee[c] (%)
1	**P8**	rt	$CHCl_3$	81	-67
2	**P8**	rt	THF	73	-62
3	**P8**	rt	Et_2O	95	-72
4	**P8**	rt	toluene	98	-81
5	**P8**	rt	$PhCF_3$	99	-73
6	**P8**	rt	*o*-xylene	98	-80
7	**P8**	rt	F_5PhCH_3	97	-79
8	**P6**	rt	toluene	97	-48
9	**P7**	rt	toluene	99	-67
10	**P8**	-10	toluene	98	-94
11	**P8**	-20	toluene	97	-98
12	**P5**	-20	toluene	95	86
13	**P6**	-20	toluene	95	-66
14	**P7**	-20	toluene	99	-90
15	**P5**	-20	F_5PhCH_3	98	95
26[d]	**P8**	-20	toluene	90	-98

[a] Reaction conditions: **1f** (0.1 mmol), **2a** (0.2 mmol), methyl acrylate (0.1 mmol) and the catalyst (0.01 mmol) in the solvent specified (1.0 mL) at room temperature for 1 h. [b] NMR yield with CH_2Br_2 as an internal standard. [c] Determined by HPLC analysis on a chiral stationary phase. [d] 50 mol % methyl acrylate was used.

Table S3. Optimization of Reaction Conditions Using Catalyst P5.[a] Related to Table 1.

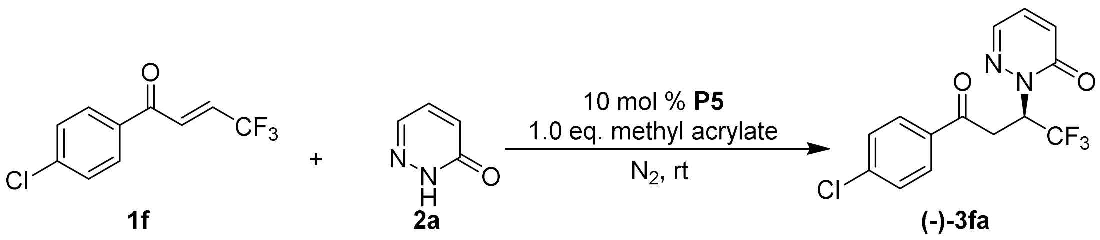

Entry	Solvent	Yield[b] (%)	Ee[c] (%)
1	$CHCl_3$	96	45
2	THF	86	55
3	Et_2O	94	64
4	toluene	95	64
5	$PhCF_3$	93	63
6	PhCl	95	60
7	F_5Ph	90	79
8	F_5PhCH_3	95	83
9	PhF	93	54
10	Mesitylene	95	71
11	*o*-xylene	92	69
12	*m*-xylene	94	69
13	*p*-xylene	NR	--
14	EA	96	47
15[d]	F_5Ph	94	91
16[d]	F_5PhCH_3	98	95
17[d]	$PhCF_3$	95	82
18[d]	toluene	95	86
19[d]	*o*-xylene	NR	--
20[d]	*m*-xylene	96	83
21[d]	mesitylene	98	86
22[d]	Et_2O	98	77

[a] Reaction conditions: **1f** (0.1 mmol), **2a** (0.2 mmol), methyl acrylate (0.1 mmol) and **P5** (0.01 mmol) in the solvent specified (1 mL) at room temperature for 1 h. [b] NMR yield with CH_2Br_2 as an internal standard. [c] Determined by HPLC analysis on a chiral stationary phase. [d] The reaction was performed at -20°C and the reaction time was 12 h.

Table S4. Nitrogen nucleophile survey.[a] Related to Scheme 4.

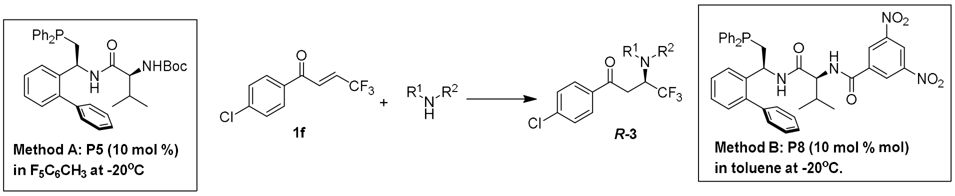

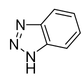	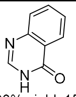	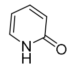	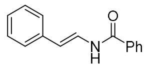
**Method A:** 80% yield, 39% ee<br>**Method B:** 89% yield, -85% ee	90% yield, 15% ee<br>98% yield, -65% ee	rt, 70% yield, 62% ee[b]<br>rt, 68% yield, -35% ee[b]	N.R.<br>N.R.

[a] Method A: **P5** (10 mol%) in $F_5C_6CH_3$ at -20°C; Method B: **P8** (10 mol% mmol) in toluene at -20°C. Determined by HPLC analysis on a chiral stationary phase. [b] 20 mol % 2-methyl-2-phenylpropionic acid as additive and the reaction was run at room temperture.

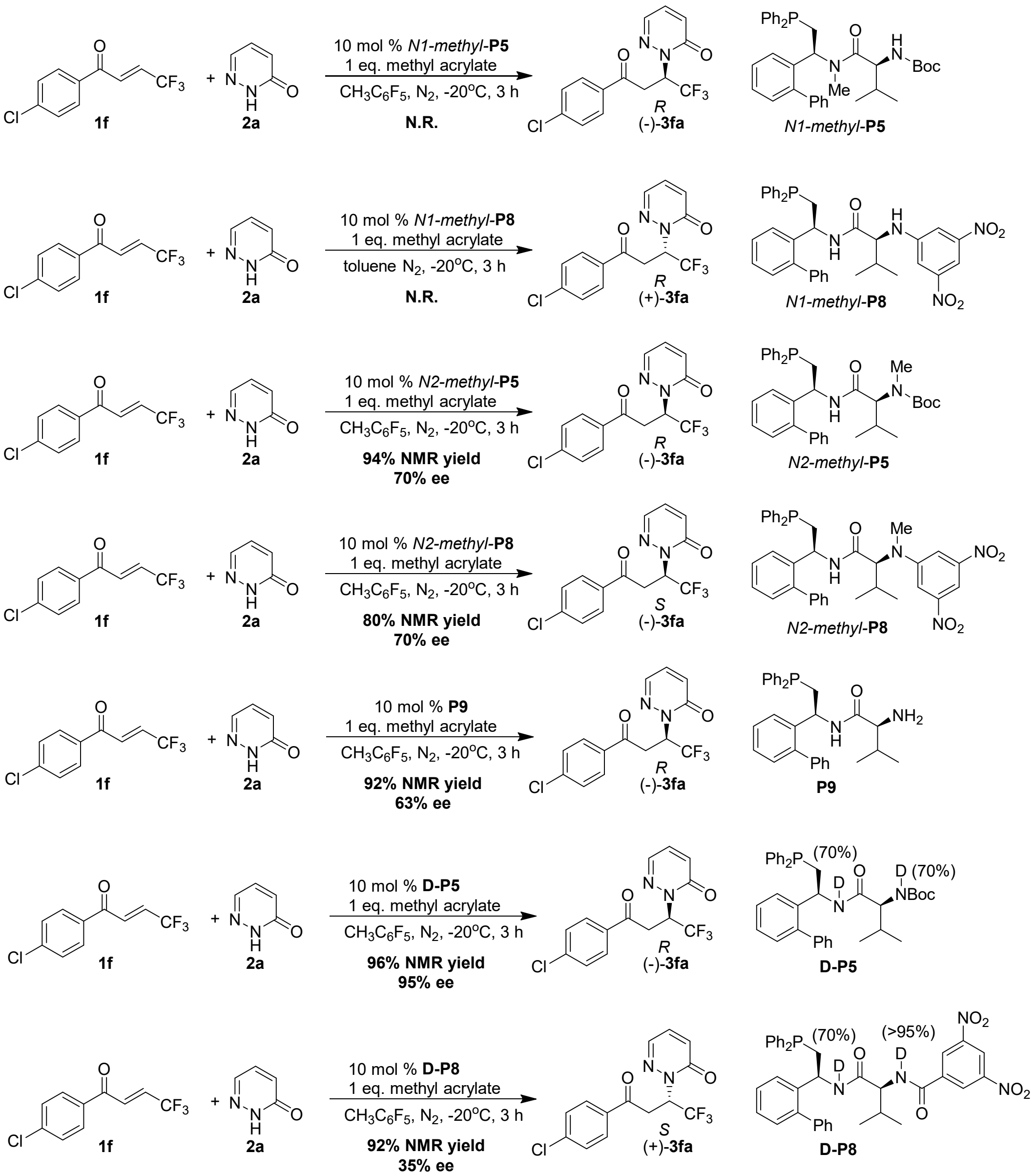

Figure S1. Some Control Experiments. Related to Scheme 7.

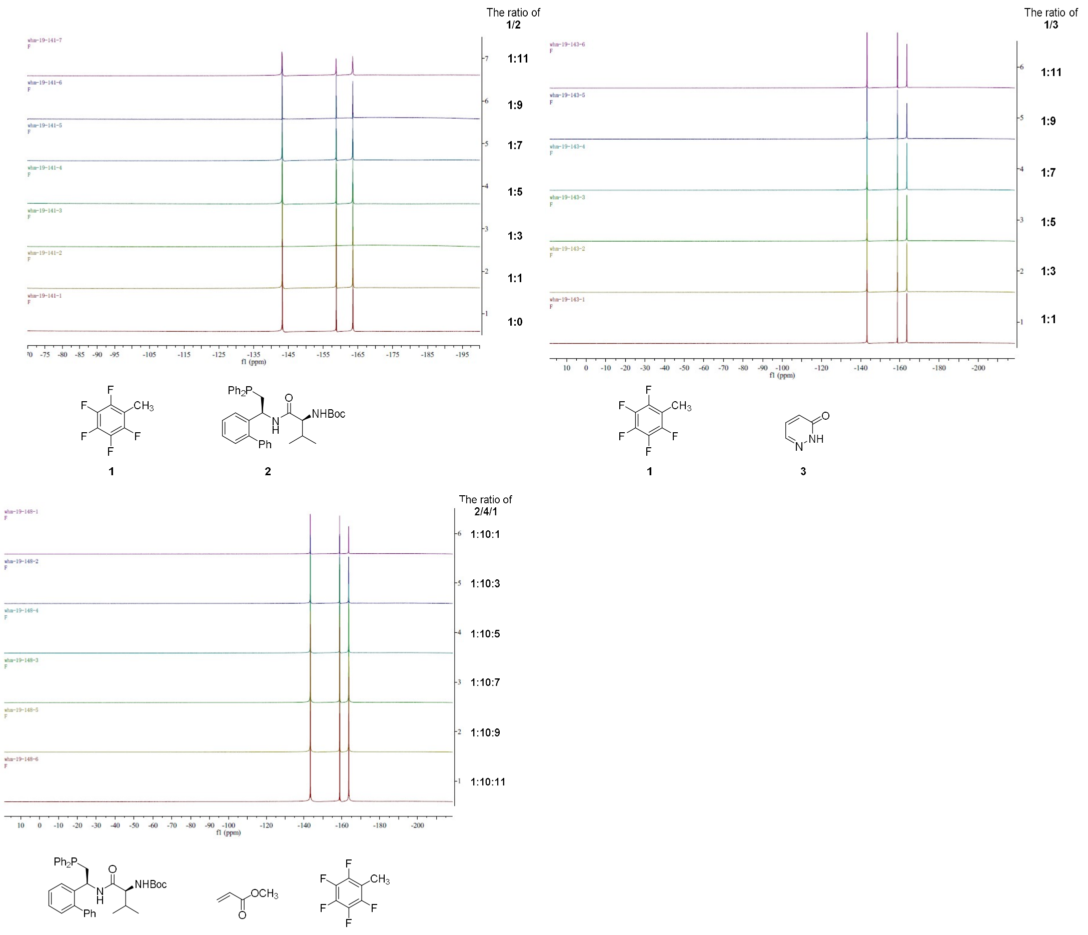

Figure S2. ^{19}**F-NMR titration experiments. Related to Scheme 7.**

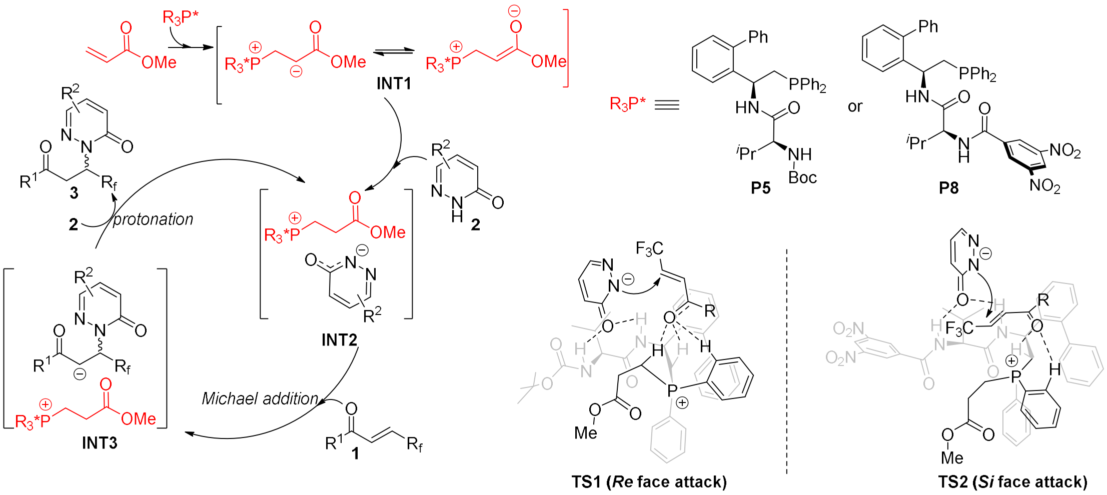

Figure S3. Proposed mechanism and transition states. Related to Scheme 7.

Data S1. Characterizations. Related to Scheme 3, Scheme 4, Scheme 5, Scheme 6 and Scheme 7.

The data of P4.

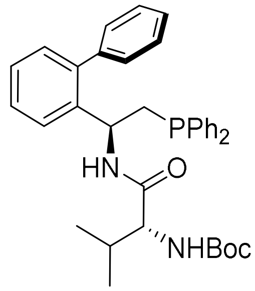

P4; white solid; yield: 70%; $[\alpha]_D^{20}$ = +34.4 (c = 1.0, $CHCl_3$); ^{1}H NMR (500 MHz, $CDCl_3$) δ 7.39-7.37 (m, 1H), 7.32 (dd, J = 7.1, 5.7 Hz, 6H), 7.28-7.22 (m, 7H), 7.15 (dd, J = 9.9, 3.8 Hz, 3H), 7.04 (t, J = 7.4 Hz, 2H), 5.25 (d, J = 6.4 Hz, 1H), 4.94 (s, 1H), 3.88 (dd, J = 8.1, 5.9 Hz, 1H), 2.89-2.80 (m, 2H), 2.32 (d, J = 6.1 Hz, 1H), 1.46 (s, 9H), 0.90 (d, J = 6.8 Hz, 3H), 0.82 (d, J = 6.8 Hz, 3H); ^{13}C NMR (126 MHz, $CDCl_3$) δ 170.57, 155.87, 141.04, 140.49, 140.25, 137.82 (d, J = 12.1 Hz), 132.60 (d, J = 19.4 Hz), 132.52 (d, J = 18.9 Hz), 130.52, 129.31, 128.66, 128.64, 128.58, 128.53, 128.52, 128.48, 128.34, 127.90, 127.17, 127.11, 125.07, 49.14, 48.74 (d, J = 14.7 Hz), 36.22, 36.09, 31.61, 30.89, 28.36, 22.67, 19.47, 17.55, 11.92; ^{31}P NMR (202 MHz, $CDCl_3$) δ -24.45; HRMS (ESI) m/z calcd. for $C_{36}H_{41}N_2NaO_3P$ $[M+Na]^+$ = 603.2747, found 603.2756.

The data of P5.

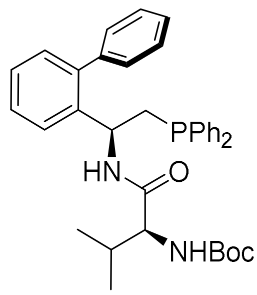

P5; white solid; yield: 82%; $[\alpha]_D^{20}$ = -6.0 (c = 0.33, $CHCl_3$); ^{1}H NMR (500 MHz, $CDCl_3$) δ 7.42 (d, J = 7.4 Hz, 1H), 7.32-7.19 (m, 13H), 7.12 (t, J = 6.5 Hz, 3H), 6.97 (t, J = 7.3 Hz, 2H), 6.71 (d, J = 5.0 Hz, 1H), 5.15 (dd, J = 10.1, 5.4 Hz, 1H), 5.02 (d, J

= 8.6 Hz, 1H), 3.85 (t, J = 7.9 Hz, 1H), 2.87-2.76 (m, 1H), 2.32 (dd, J = 13.8, 2.6 Hz, 1H), 2.26-2.20 (m, 1H), 2.03 (d, J = 6.3 Hz, 1H), 1.45 (s, 9H), 0.94-0.86 (m, 6H); ^{13}C NMR (126 MHz, $CDCl_3$) δ 170.88, 156.00, 140.80, 140.70, 140.65, 140.59, 138.06 (d, J = 11.9 Hz), 135.99 (d, J = 12.8 Hz), 132.82 (d, J = 19.9 Hz), 132.20 (d, J = 18.5 Hz), 130.51, 129.34, 128.82, 128.66, 128.60, 128.46, 128.44, 128.41, 128.01, 127.11, 127.06, 124.89, 79.82, 60.29, 49.05 (d, J = 6.4 Hz), 48.75 (d, J = 12.9 Hz), 36.19 (d, J = 17.0 Hz), 30.54, 28.38, 19.63, 18.11, 11.80 (d, J = 2.5 Hz); ^{31}P NMR (202 MHz, $CDCl_3$) δ -24.43 (s); HRMS (ESI) m/z calcd. for $C_{36}H_{42}N_2O_3P$ $[M+H]^+$ = 581.2928, found 581.2941.

The data of P6.

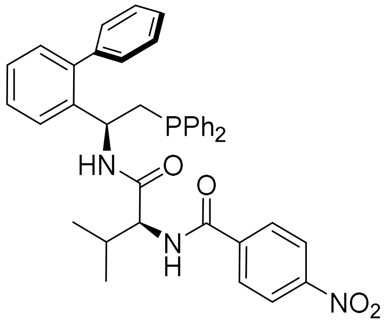

P6; pale yellow solid; yield: 75%; $[\alpha]_D^{20}$ = -14.7 (c = 0.33, $CHCl_3$); ^{1}H NMR (400 MHz, $CDCl_3$) δ 8.00 (d, J = 8.7 Hz, 2H), 7.80 (d, J = 8.7 Hz, 2H), 7.40-7.09 (m, 17H), 6.97 (t, J = 7.5 Hz, 2H), 5.27-5.20 (m, 1H), 4.53 (t, J = 7.7 Hz, 1H), 2.37-2.32 (m, 1H), 2.27-2.15 (m, 2H), 1.72 (s, 1H), 1.26 (s, 1H), 1.02 (dd, J = 18.4, 6.7 Hz, 6H); ^{13}C NMR (101 MHz, $CDCl_3$) δ 170.33, 165.49, 149.56, 140.62, 140.56, 140.45, 139.34, 137.82 (d, J = 11.5 Hz), 135.96 (d, J = 12.6 Hz), 132.70 (d, J = 19.8 Hz), 132.18 (d, J = 18.9 Hz), 130.58, 129.23, 128.86, 128.68, 128.61, 128.51, 128.43, 128.22, 128.12, 127.22 (d, J = 8.3 Hz), 124.76, 123.59, 59.47, 49.03 (d, J = 13.9 Hz), 36.31 (d, J = 17.4 Hz), 31.38, 19.10 (d, J = 122.1 Hz); ^{31}P NMR (162 MHz, $CDCl_3$) δ -24.42 (s); HRMS (ESI) m/z calcd. for $C_{38}H_{37}N_3O_4P$ $[M+H]^+$ = 630.2516, found 630.2529.

The data of P7.

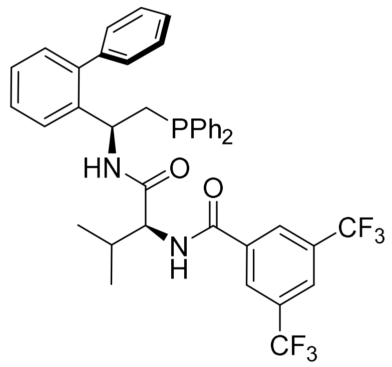

P7; white solid; yield: 84%; $[\alpha]_D^{20}$ = -12.0 (*c* = 0.33, $CHCl_3$); ^{1}H NMR (400 MHz, $CDCl_3$) δ 8.24 (s, 2H), 7.96 (s, 1H), 7.34-7.11 (m, 15H), 7.05 (d, *J* = 7.1 Hz, 1H), 6.98 (t, *J* = 7.2 Hz, 3H), 5.27-5.21 (m, 1H), 4.42 (t, *J* = 6.3 Hz, 1H), 2.35 (d, *J* = 12.4 Hz, 1H), 2.25-2.18 (m, 2H), 1.98 (s, 1H), 1.26 (s, 1H), 0.97 (dd, *J* = 17.2, 6.6 Hz, 6H); ^{13}C NMR (101 MHz, $CDCl_3$) δ167.41 (d, *J* = 537.3 Hz), 140.65, 140.39, 137.83 (d, *J* = 11.6 Hz), 136.12, 135.98, 132.66 (d, *J* = 19.8 Hz), 132.26, 132.07, 131.92, 131.58, 130.50, 129.23, 128.81, 128.61 (d, *J* = 7.0 Hz), 128.47 (d, *J* = 6.3 Hz), 128.37, 128.34, 127.98, 127.50 (d, *J* = 2.5 Hz), 127.12, 126.91, 124.74, 122.84 (q, *J* = 273.0 Hz), 59.76, 31.20, 48.90 (d, *J* = 13.8 Hz), 36.41 (d, *J* = 17.2 Hz), 18.97 (d, *J* = 114.3 Hz); ^{31}P NMR (162 MHz, $CDCl_3$) δ -24.37 (s); ^{19}F NMR (376 MHz, $CDCl_3$) δ -62.87 (s); HRMS (ESI) m/z calcd. for $C_{40}H_{36}F_6N_2O_2P$ $[M+H]^+$ = 721.2413, found 721.2421.

The data of P5.

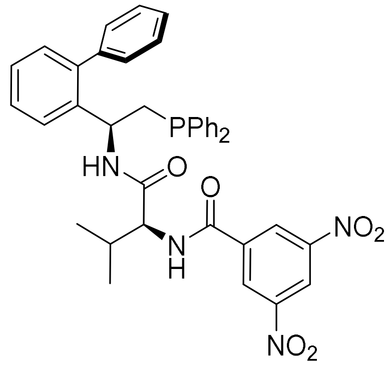

P8; yellow solid; yield: 78%; $[\alpha]_D^{20}$ = -29.1 (*c* = 0.33, $CHCl_3$); ^{1}H NMR (500 MHz, $CDCl_3$) δ 8.92 (s, 2H), 8.37 (s, 1H), 7.35-7.15 (m, 15H), 6.96 (dd, *J* = 25.1, 18.2 Hz, 4H), 5.17 (d, *J* = 4.1 Hz, 1H), 4.82 (s, 1H), 2.29 (d, *J* = 14.5 Hz, 2H), 2.16 (s, 1H), 1.91 (s, 1H), 1.11 (d, *J* = 5.5 Hz, 3H), 0.96 (d, *J* = 5.7 Hz, 3H); ^{13}C NMR (126 MHz, $CDCl_3$) δ 170.43, 163.38, 148.02, 140.40 (d, *J* = 5.7 Hz), 140.20 (d, *J* = 26.6 Hz),

137.80 (d, *J* = 11.5 Hz), 137.32, 135.45 (d, *J* = 12.6 Hz), 132.80 (d, *J* = 20.0 Hz), 131.96 (d, *J* = 18.4 Hz), 130.29, 129.09, 128.98, 128.54, 128.50, 128.46, 128.23, 128.70 (d, *J* = 7.4 Hz), 127.63, 127.20 (d, *J* = 17.4 Hz), 124.59, 121.04, 59.53, 48.86 (d, *J* = 12.6 Hz), 36.39 (d, *J* = 17.2 Hz), 32.18, 29.72, 29.38, 22.72, 14.15, 19.05 (d, *J* = 200.9 Hz); ^{31}P NMR (202 MHz, $CDCl_3$) δ -24.59 (s); HRMS (ESI) m/z calcd. for $C_{38}H_{36}N_4O_6P$ [M+H] $^+$ = 675.2367, found 675.2384.

The data of *N1*-methyl-P5.

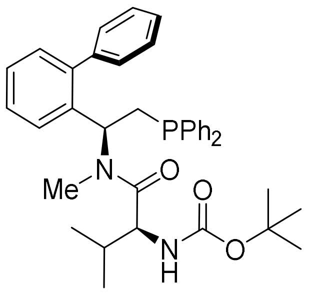

N1-methyl-**P5**; white solid; yield: 44% yield for three steps; $[\alpha]_D^{20}$ = +53.0 (*c* = 0.33, $CHCl_3$); ^{1}H NMR (500 MHz, $CDCl_3$) δ 7.57 (d, *J* = 7.7 Hz, 1H), 7.40-7.36 (m, 3H), 7.31 (dd, *J* = 5.8, 2.7 Hz, 4H), 7.29-7.19 (m, 8H), 7.13 (dd, *J* = 7.4, 1.0 Hz, 1H), 6.95 (d, *J* = 6.8 Hz, 2H), 5.69 (dd, *J* = 14.3, 7.6 Hz, 1H), 5.07 (d, *J* = 9.2 Hz, 1H), 4.24 (dd, *J* = 9.2, 5.7 Hz, 1H), 2.81 (s, 3H), 2.63 (dd, *J* = 13.8, 7.4 Hz, 1H), 2.50 (dd, *J* = 13.7, 9.2 Hz, 1H), 1.91-1.85 (m, 1H), 1.45 (s, 9H), 0.92 (d, *J* = 6.8 Hz, 3H), 0.81 (d, *J* = 6.7 Hz, 3H); ^{13}C NMR (126 MHz, $CDCl_3$) δ 171.17, 155.76, 143.08, 140.69, 138.28 (d, *J* = 14.5 Hz), 137.60 (d, *J* = 12.9 Hz), 136.69 (d, *J* = 5.4 Hz), 133.01, 132.86, 132.73, 132.58, 130.66, 128.77, 128.62, 128.55, 128.50, 128.16, 127.49, 127.77 (d, *J* = 3.2 Hz), 127.13 (d, *J* = 15.6 Hz), 79.13, 55.31, 52.62, 52.49, 31.59 (d, *J* = 3.8 Hz), 31.20, 31.14, 31.07, 28.44, 19.86, 17.20; ^{31}P NMR (202 MHz, $CDCl_3$) δ -22.75; HRMS (ESI) m/z calcd. for $C_{37}H_{43}N_2NaO_3P$ [M+Na] $^+$ = 617.2904, found 617.2908.

The data of *N1*-methyl-P8.

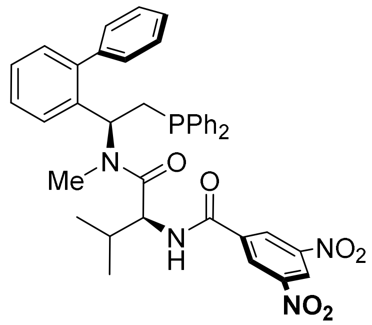

N1-methyl-**P8**; light yellow solid; yield: 60%; $[\alpha]_D^{20}$ = +55.3 (*c* = 1.0, $CHCl_3$); ^1H NMR (500 MHz, $CDCl_3$) δ 9.00-8.98 (m, 1H), 8.93 (d, *J* = 1.9 Hz, 2H), 7.46 (d, *J* = 5.8 Hz, 1H), 7.36-7.30 (m, 9H), 7.24 (dd, *J* = 10.1, 4.5 Hz, 2H), 7.12 (d, *J* = 6.8 Hz, 5H), 7.01 (s, 1H), 6.93 (d, *J* = 6.7 Hz, 2H), 5.74 (dd, *J* = 10.4, 5.4 Hz, 1H), 5.04 (d, *J* = 4.1 Hz, 1H), 3.01 (s, 3H), 2.63-2.60 (m, 1H), 2.48 (d, *J* = 9.4 Hz, 1H), 2.18-2.16 (m, 1H), 1.88-1.78 (m, 1H), 1.09 (d, *J* = 6.6 Hz, 3H), 0.84 (d, *J* = 6.6 Hz, 3H); ^{13}C NMR (126 MHz, $CDCl_3$) δ 170.94, 170.82, 162.71, 162.67, 153.34, 148.33, 148.28, 148.26, 142.27, 142.19, 140.70, 140.05, 137.84, 137.80, 137.30, 137.19, 132.80, 132.69, 132.64, 132.53, 130.89, 128.94, 128.79, 128.70, 128.65, 128.61, 128.56, 128.33, 127.58 (d, *J* = 5.2 Hz), 127.30, 126.94, 126.70, 121.08, 120.22, 60.46, 56.59, 55.11, 53.23 (d, *J* = 15.7 Hz), 50.40, 32.45, 31.77 (d, *J* = 17.0 Hz), 31.43, 31.10 (d, *J* = 7.2 Hz), 26.05, 25.22 (d, *J* = 5.7 Hz), 24.61, 21.09, 20.37, 17.23, 14.24; ^{31}P NMR (202 MHz, $CDCl_3$) δ -22.13; HRMS (ESI) m/z calcd. for $C_{39}H_{37}N_4NaO_6P$ $[M+Na]^+$ = 711.2343, found 711.2352.

The data of *N2*-methyl-P5.

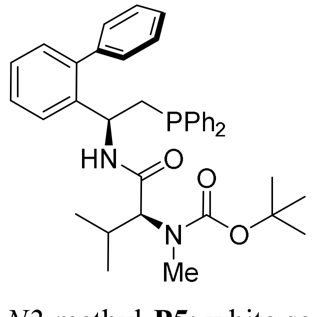

N2-methyl-**P5**; white solid; yield: 79%; $[\alpha]_D^{20}$ = -35.6 (*c* = 1.0, $CHCl_3$); ^1H NMR (400 MHz, $CDCl_3$) δ 7.32 (s, 6H), 7.24-7.18 (m, 8H), 7.12-7.09 (m, 3H), 6.93 (t, *J* = 7.5 Hz, 2H), 6.84 (d, *J* = 6.9 Hz, 1H), 5.21-5.16 (m, 1H), 4.07 (d, *J* = 11.2 Hz, 1H), 2.71 (s, 3H), 2.30-2.25 (m, 1H), 2.24-2.19 (m, 1H), 2.17-2.09 (m, 1H), 1.51 (s, 9H); ^{13}C NMR

(101 MHz, $CDCl_3$) δ 169.41, 57.18, 141.16 (d, *J* = 5.2 Hz), 140.86, 140.53, 138.42 (d, *J* = 12.3 Hz), 136.29 (d, *J* = 13.4 Hz), 132.81 (d, *J* = 20.0 Hz), 132.24 (d, *J* = 18.6 Hz), 130.47, 129.31, 128.67, 128.61, 128.54, 128.45, 128.40, 128.33, 127.88, 127.08, 126.90, 124.56, 80.16, 64.60, 47.93, 47.81, 36.48, 36.31, 29.94, 28.48, 25.78, 19.93, 18.74; ^{31}P NMR (202 MHz, $CDCl_3$) δ -23.75; HRMS (ESI) m/z calcd. for $C_{37}H_{43}N_2NaO_3P$ $[M+Na]^+$ = 617.2904, found 617.2897.

The data of *N1*-methyl-P8.

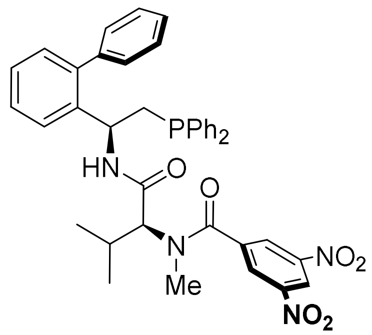

N2-methyl-**P8**; light yellow solid; yield: 76%; $[\alpha]_D^{20}$ = -39.5 (*c* = 0.33, $CHCl_3$); ^{1}H NMR (400 MHz, $CDCl_3$) δ 9.06 (s, 1H), 8.55 (d, *J* = 1.7 Hz, 2H), 7.46 (q, *J* = 7.7 Hz, 2H), 7.33-7.29 (m, 6H), 7.24-7.21 (m, 5H), 7.17-7.10 (m, 4H), 6.98 (t, *J* = 7.5 Hz, 2H), 5.29-5.22 (m, 1H), 4.55 (d, *J* = 11.3 Hz, 1H), 2.87 (s, 3H), 2.43-2.30 (m, 2H), 2.28-2.20(m, 1H), 1.05 (d, *J* = 6.4 Hz, 6H); ^{31}P NMR (122 MHz, $CDCl_3$) δ -23.57; ^{13}C NMR (101 MHz, $CDCl_3$) δ 168.18, 167.57, 148.56, 141.24 (d, *J* = 6.0 Hz), 140.62 (d, *J* = 59.0 Hz), 139.36, 138.31 (d, *J* = 11.9 Hz), 135.96 (d, *J* = 13.2 Hz), 132.85 (d, *J* = 20.0 Hz), 132.14 (d, *J* = 18.5 Hz), 130.79, 129.24, 128.91, 128.70 (d, *J* = 7.3 Hz), 128.55, 128.53, 128.50, 128.49, 127.99, 127.36, 127.25, 124.40, 119.95, 48.23, 48.11, 36.34, 36.17, 33.75, 25.64, 19.69, 19.02; HRMS (ESI) m/z calcd. for $C_{39}H_{37}N_4NaO_6P$ $[M+Na]^+$ = 711.2343, found 711.2356.

The data of P9.

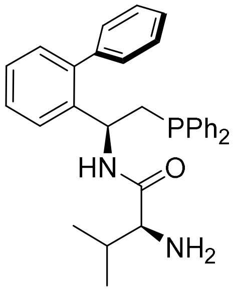

P9; white solid; yield: 80%; $[\alpha]_D^{20}$ = -1.6 (*c* = 0.33, $CHCl_3$); ^{1}H NMR (500 MHz, $CDCl_3$) δ 7.92 (d, *J* = 6.9 Hz, 1H), 7.41-7.38 (m, 3H), 7.31-7.28 (m, 4H), 7.26-7.20 (m, 7H), 7.15-7.11 (m, 3H), 7.00 (dd, *J* = 11.7, 4.1 Hz, 2H), 5.30-5.27 (m, 1H), 3.18 (d, *J* = 3.9 Hz, 1H), 2.32 (d, *J* = 7.4 Hz, 2H), 2.28-2.22 (m, 1H), 1.59 (s, 2H), 0.93 (d, *J* = 7.0 Hz, 3H), 0.76 (d, *J* = 6.9 Hz, 3H); ^{13}C NMR (126 MHz, $CDCl_3$) δ 173.45, 141.16 (d, *J* = 5.9 Hz), 140.94, 140.66, 138.25 (d, *J* = 11.9 Hz), 137.00 (d, *J* = 12.7 Hz), 132.64 (d, *J* = 6.4 Hz), 132.49 (d, *J* = 6.8 Hz),130.55, 129.48, 128.60, 128.54, 128.52, 128.47, 128.42, 128.37, 127.84, 125.12, 60.12, 48.28 (d, *J* = 14.4 Hz), 36.77 (d, *J* = 16.6 Hz) 30.88, 19.87, 16.21; ^{31}P NMR (202 MHz, $CDCl_3$) δ -23.64; HRMS (ESI) m/z calcd. for $C_{31}H_{34}N_2OP$ $[M+H]^+$ = 481.2403, found 481.2404.

The data of P10.

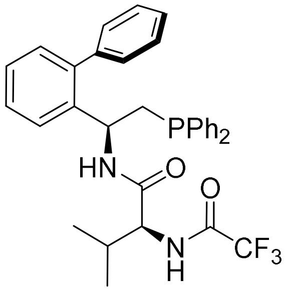

P10; white solid; $[\alpha]_D^{20}$ = -7.8 (*c* = 0.33, $CHCl_3$); ^{1}H NMR (500 MHz, $CDCl_3$) δ 7.35-7.22 (m, 14H), 7.17 (d, *J* = 7.4 Hz, 2H), 7.05 (d, *J* = 7.2 Hz, 1H), 7.01 (t, *J* = 7.4 Hz, 2H), 6.46 (s, 1H), 5.19 (d, *J* = 5.0 Hz, 1H), 4.29 (t, *J* = 6.9 Hz, 1H), 2.37 (d, *J* = 11.8 Hz, 1H), 2.30-2.24 (m, 1H), 2.09 (dd, *J* = 12.9, 6.5 Hz, 1H), 1.65 (s, 1H), 0.96 (d, *J* = 6.5 Hz, 3H), 0.92 (d, *J* = 6.6 Hz, 3H); ^{19}F NMR (376 MHz, $CDCl_3$) δ -75.65; ^{31}P NMR (202 MHz, $CDCl_3$) δ -24.45; ^{13}C NMR (126 MHz, $CDCl_3$) δ 168.30, 157.20 (q, *J* = 37.5 Hz), 140.76, 140.44, 140.02 (d, *J* = 5.6 Hz), 137.63 (d, *J* = 11.1 Hz), 135.82 (d, *J* = 13.1 Hz), 132.75 (d, *J* = 19.8 Hz), 132.21 (d, *J* = 18.7 Hz), 130.68, 129.24,

129.00, 128.75, 128.69, 128.68, 128.58, 128.53, 128.48, 128.17, 127.32 (d, *J* = 13.9 Hz), 115.77 (q, *J* = 287.7 Hz), 124.75, 58.66, 49.20 (d, *J* = 13.3 Hz), 36.20 (d, *J* = 17.1 Hz), 31.76, 21.53, 19.31, 17.89; HRMS (ESI) m/z calcd. for $C_{33}H_{33}F_3N_2O_2P$ [M+H] $^+$ = 577.2226, found 577.2229.

The data of P12.

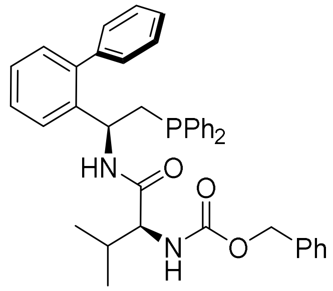

P12; white solid; $[\alpha]_D^{20}$ = -3.3 (*c* = 1.0, $CHCl_3$); ^{1}H NMR (300 MHz, $CDCl_3$) δ 7.31 (s, 11H), 7.23 (d, *J* = 10.3 Hz, 7H), 7.12 (dd, *J* = 9.4, 4.8 Hz, 3H), 6.98 (t, *J* = 7.6 Hz, 2H), 6.73 (d, *J* = 6.4 Hz, 1H), 5.40 (s, 1H), 5.17 (dd, *J* = 15.5, 6.5 Hz, 1H), 5.09 (s, 2H), 4.02 (d, *J* = 6.8 Hz, 1H), 2.80 (dd, *J* = 12.3, 5.5 Hz, 2H), 2.30-2.23 (m, 1H), 2.13-2.04 (m, 1H), 0.95 (d, *J* = 6.7 Hz, 3H), 0.89 (d, *J* = 6.6 Hz, 3H); ^{31}P NMR (162 MHz, $CDCl_3$) δ -24.34; ^{13}C NMR (101 MHz, $CDCl_3$) δ 170.56, 156.64, 140.80, 140.69, 140.60, 138.12 (d, *J* = 11.9 Hz),136.33, 136.26, 136.13, 132.83 (d, *J* = 19.8 Hz), 132.25 (d, *J* = 18.6 Hz), 130.54, 129.36, 128.85, 128.71, 128.64, 128.56, 128.51, 128.48, 128. 45, 128.16, 128.09, 128.01, 127.16, 127.13, 125.02, 67.02, 60.56, 48.95 (d, *J* = 3.4 Hz), 48.76 (d, *J* = 13.3 Hz), 36.35 (d, *J* = 16.9 Hz), 31.11, 21.57, 19.67, 17.98, 14.27, 11.69 (d, *J* = 4.0 Hz); HRMS (ESI) m/z calcd. for $C_{39}H_{40}N_2O_3P$ [M+H] $^+$ = 615.2771, found 615.2770.

The data of P13.

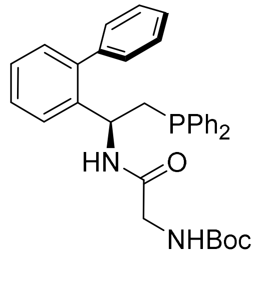

P13; white solid; $[\alpha]_D^{20}$ = +17.530 (*c* = 0.33, $CHCl_3$); ^{1}H NMR (500 MHz, $CDCl_3$) δ 7.39 (d, *J* = 7.7 Hz, 1H), 7.31-7.28 (m, 6H), 7.24-7.21 (m, 6H), 7.16-7.13 (m, 3H), 7.04 (t, *J* = 7.3 Hz, 2H), 6.75 (s, 1H), 5.28-5.25 (m, 1H), 5.13 (s, 1H), 3.68 (s, 2H), 2.32 (d, *J* = 7.3 Hz, 2H), 1.44 (s, 9H); ^{31}P NMR (202 MHz, $CDCl_3$) δ -23.96; ^{13}C NMR (126 MHz, $CDCl_3$) δ 168.31, 156.07, 140.74 (d, *J* = 46.8 Hz), 140.43 (d, *J* = 5.8 Hz), 138.00 (d, *J* = 12.1 Hz), 137.03 (d, *J* = 12.7 Hz), 132.72, 132.59, 132.57, 132.44, 130.54, 129.33, 128.67, 128.62, 128.59, 128.53, 128.52, 128.46, 128.37, 127.98, 127.18, 125.25, 60.44, 44.31, 48.73 (d, *J* = 14.9 Hz), 36.54 (d, *J* = 16.9 Hz), 28.38, 21.09, 14.24; HRMS (ESI) m/z calcd. for $C_{33}H_{36}N_2O_3P$ $[M+H]^+$ = 539.2458, found 539.2459.

(*S*)-2-(1,1,1-trifluoro-4-oxo-4-phenylbutan-2-yl)pyridazin-3(*2H*)-one

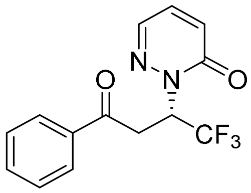

(+)-3aa; isolated yield: 28.1 mg (95%); colorless sticky oil; $[\alpha]_D^{20}$ = +291.9 (*c* = 1.0, $CHCl_3$); ^{1}H NMR (400 MHz, $CDCl_3$) δ 7.97-7.94 (m, 2H), 7.72 (dd, *J* = 3.7, 1.6 Hz, 1H), 7.60 (t, *J* = 7.4 Hz, 1H), 7.48 (t, *J* = 7.7 Hz, 2H), 7.14 (dd, *J* = 9.5, 3.7 Hz, 1H), 6.99 (dd, *J* = 9.5, 1.6 Hz, 1H), 6.47-6.38 (m, 1H), 4.29 (dd, *J* = 18.1, 10.9 Hz, 1H), 3.52 (dd, *J* = 18.1, 2.9 Hz, 1H); ^{13}C NMR (101 MHz, $CDCl_3$) δ 193.85, 160.11, 136.59, 135.79, 133.76, 131.04, 130.22, 128.74, 128.06, 124.41 (q, *J* = 282.8 Hz), 52.89 (q, *J* = 31.5 Hz), 35.30; ^{19}F NMR (376 MHz, $CDCl_3$) δ -73.16 (s); Enantiomeric excess: 95%, determined by HPLC (Chiralpak AD-H, hexane/*i*-PrOH = 80/20; flow rate 1.0 ml/min; 25 °C; 254 nm), first peak: t_R = 9.69 min, second peak: t_R = 13.64 min; HRMS (ESI) m/z calcd. for $C_{14}H_{11}F_3N_2NaO_2$ $[M+Na]^+$ = 319.0665, found 319.0667.

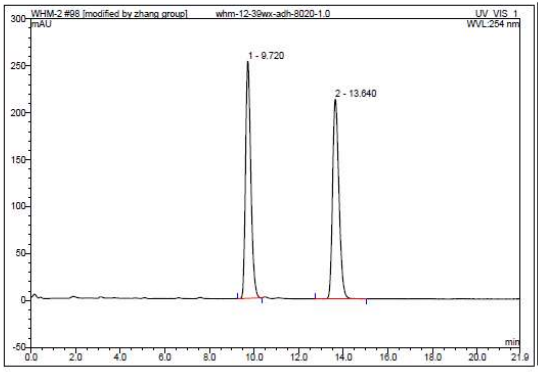

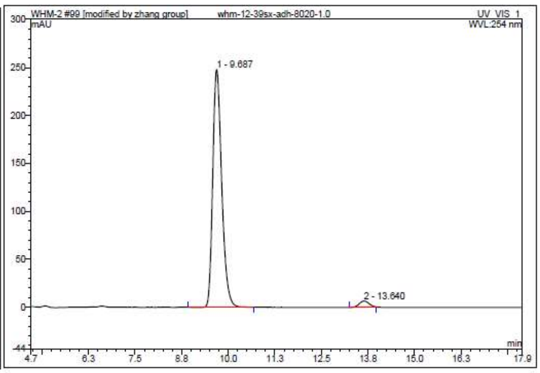

No.	Ret.Time min	Peak Name	Height mAU	Area mAU*min	Rel.Area %	Amount	Type
1	9.72	n.a.	252.603	69.783	49.93	n.a.	BMB*
2	13.64	n.a.	212.738	69.992	50.07	n.a.	BMB*
Total:			465.340	139.775	100.00	0.000	

No.	Ret.Time min	Peak Name	Height mAU	Area mAU*min	Rel.Area %	Amount	Type
1	9.69	n.a.	248.321	69.207	97.32	n.a.	BMB*
2	13.64	n.a.	6.384	1.904	2.68	n.a.	BMB*
Total:			254.705	71.111	100.00	0.000	

(*S*)-2-(1,1,1-trifluoro-4-(4-methoxyphenyl)-4-oxobutan-2-yl)pyridazin-3 (*2H*)-one

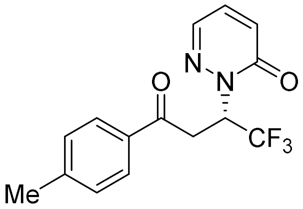

(+)-3ba; isolated yield: 30.0 mg (97%); colorless sticky oil; $[\alpha]_D^{20}$ = +230.3 (*c* = 1.0, $CHCl_3$); ^1H NMR (400 MHz, $CDCl_3$) δ 7.85 (d, *J* = 8.2 Hz, 2H), 7.71 (dd, *J* = 3.6, 1.6 Hz, 1H), 7.27 (d, *J* = 8.0 Hz, 2H), 7.13 (dd, *J* = 9.5, 3.7 Hz, 1H), 6.98 (dd, *J* = 9.5, 1.7 Hz, 1H), 6.64-6.37 (m, 1H), 4.25 (dd, *J* = 18.0, 10.9 Hz, 1H), 3.49 (dd, *J* = 18.0, 2.9 Hz, 1H), 2.41 (s, 3H); ^{13}C NMR (101 MHz, $CDCl_3$) δ 193.47, 160.16, 144.78, 136.59, 133.41, 131.06, 130.25, 129.45, 128.23, 124.49 (q, *J* = 282.8 Hz), 52.98 (q, *J* = 31.4 Hz), 35.20, 21.66 (q, *J* = 2.6 Hz); ^{19}F NMR (376 MHz, $CDCl_3$) δ -73.14 (s); Enantiomeric excess: 96%, determined by HPLC (Chiralpak AD-H, hexane/*i*-PrOH = 80/20; flow rate 1.0 ml/min; 25 °C; 254 nm), first peak: t_R = 10.29 min, second peak: t_R = 15.93 min; HRMS (ESI) m/z calcd. for $C_{15}H_{13}F_3N_2NaO_2$ $[M+Na]^+$ = 333.0821, found 333.0824.

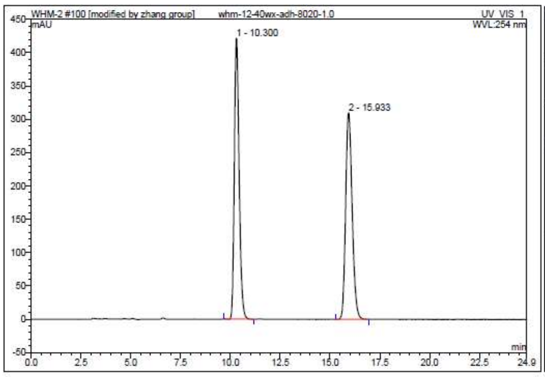

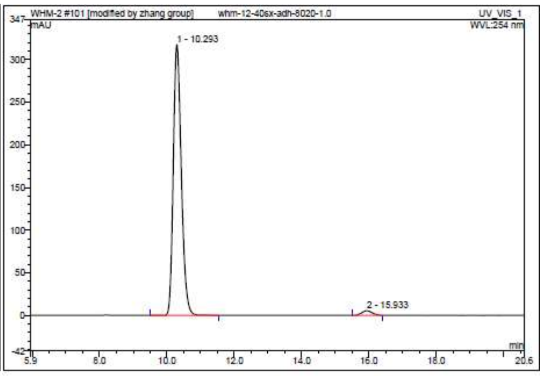

No.	Ret.Time min	Peak Name	Height mAU	Area mAU*min	Rel.Area %	Amount	Type
1	10.30	n.a.	421.575	117.395	49.97	n.a.	BMB*
2	15.93	n.a.	309.991	117.515	50.03	n.a.	BMB*
Total:			731.567	234.910	100.00	0.000	

No.	Ret.Time min	Peak Name	Height mAU	Area mAU*min	Rel.Area %	Amount	Type
1	10.29	n.a.	317.413	87.932	97.83	n.a.	BMB*
2	15.93	n.a.	5.412	1.946	2.17	n.a.	BMB*
Total:			322.825	89.879	100.00	0.000	

(*S*)-2-(1,1,1-trifluoro-4-oxo-4-(*p*-tolyl)butan-2-yl)pyridazin-3(*2H*)-one

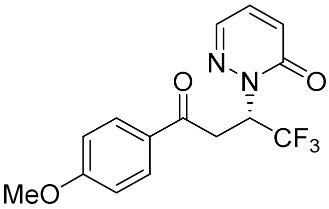

(+)-3ca; isolated yield: 28.7 mg (88%); colorless sticky oil; $[\alpha]_D^{20}$ = +355.6 (*c* = 1.0, $CHCl_3$); ^{1}H NMR (500 MHz, $CDCl_3$) δ 7.93 (d, *J* = 8.9 Hz, 2H), 7.72 (dd, *J* = 3.6, 1.6 Hz, 1H), 7.13 (dd, *J* = 9.5, 3.7 Hz, 1H), 7.00-6.93 (m, 3H), 6.45-6.38 (m, 1H), 4.23 (dd, *J* = 17.9, 11.0 Hz, 1H), 3.87 (s, 3H), 3.46 (dd, *J* = 17.9, 2.9 Hz, 1H); ^{13}C NMR (126 MHz, $CDCl_3$) δ 192.27, 164.00, 160.14, 136.55, 131.02, 130.41, 130.23, 128.88, 124.46 (q, *J* = 282.8 Hz), 113.89, 55.51, 52.95 (q, *J* = 31.3 Hz), 34.90; ^{19}F NMR (376 MHz, $CDCl_3$) δ -73.13 (s); Enantiomeric excess: 93%, determined by HPLC (Chiralpak AD-H, hexane/*i*-PrOH = 80/20; flow rate 1.0 ml/min; 25 °C; 254 nm), first peak: t_R = 14.74 min, second peak: t_R = 24.57 min; HRMS (ESI) m/z calcd. for $C_{15}H_{13}F_3N_2NaO_3$ $[M+Na]^+$ = 349.0770, found 349.0769.

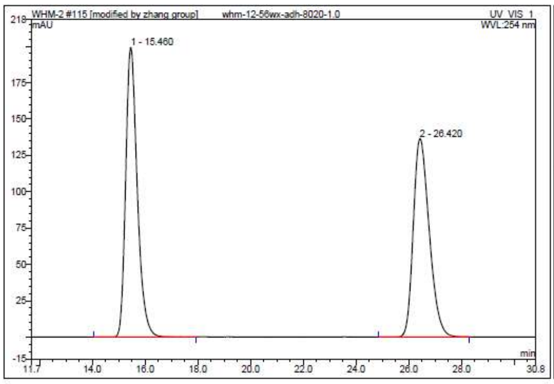

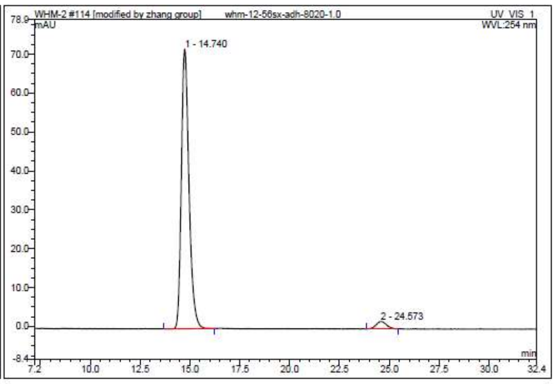

No.	Ret.Time min	Peak Name	Height mAU	Area mAU*min	Rel.Area %	Amount	Type
1	15.46	n.a.	199.091	101.056	51.51	n.a.	BMB*
2	26.42	n.a.	136.285	95.125	48.49	n.a.	BMB*
Total:			335.376	196.181	100.00	0.000	

No.	Ret.Time min	Peak Name	Height mAU	Area mAU*min	Rel.Area %	Amount	Type
1	14.74	n.a.	71.928	31.944	96.49	n.a.	BMB*
2	24.57	n.a.	1.915	1.163	3.51	n.a.	BMB*
Total:			73.843	33.107	100.00	0.000	

(*S*)-2-(4-([1,1'-biphenyl]-4-yl)-1,1,1-trifluoro-4-oxobutan-2-yl)pyridazin-3(*2H*)-one

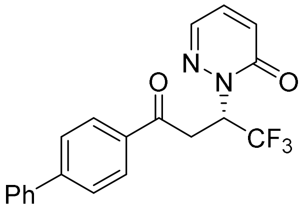

(+)-3da; isolated yield: 36.1 mg (97%); white solid; $[\alpha]_D^{20}$ = +321.9 (*c* = 1.0, $CHCl_3$); ^{1}H NMR (400 MHz, $CDCl_3$) δ 8.02 (d, *J* = 8.4 Hz, 2H), 7.75-7.64 (m, 3H), 7.62-7.60 (m, 2H), 7.51-7.39 (m, 3H), 7.13 (dd, *J* = 9.5, 3.7 Hz, 1H), 6.99 (dd, *J* = 9.6, 1.7 Hz, 1H), 6.49-6.40 (m, 1H), 4.32 (dd, *J* = 18.0, 10.9 Hz, 1H), 3.55 (dd, *J* = 18.0, 2.9 Hz, 1H); ^{13}C NMR (101 MHz, $CDCl_3$) δ 193.40, 160.12, 146.48, 139.55, 136.60, 134.47, 131.04, 130.24, 128.97, 128.68, 128.40, 127.34, 127.22, 124.44 (q, *J* = 282.8 Hz), 52.94 (q, *J* = 31.5 Hz), 35.32; ^{19}F NMR (376 MHz, $CDCl_3$) δ -73.09 (s); Enantiomeric excess: 95%, determined by HPLC (Chiralpak AD-H, hexane/*i*-PrOH = 80/20; flow rate 1.0 ml/min; 25 °C; 254 nm), first peak: t_R = 15.51 min, second peak: t_R = 27.28 min; HRMS (ESI) m/z calcd. for $C_{20}H_{15}F_3N_2NaO_2$ $[M+Na]^+$ = 395.0978, found = 395.0977.

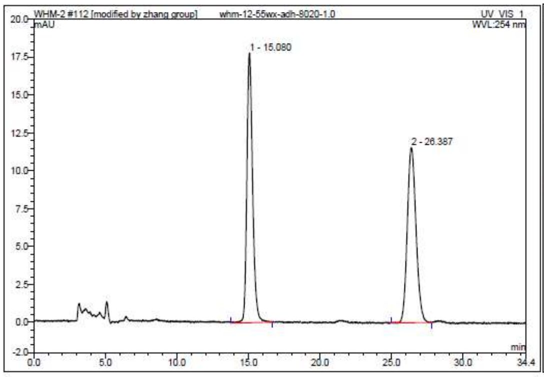

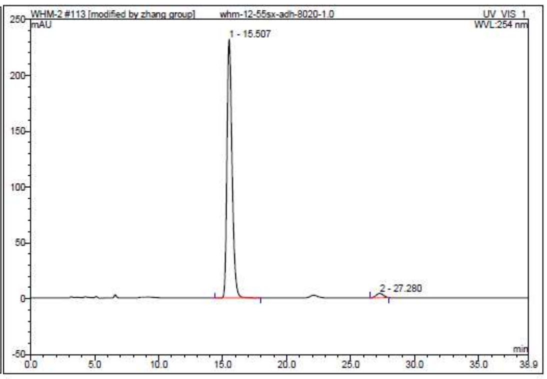

No.	Ret.Time min	Peak Name	Height mAU	Area mAU*min	Rel.Area %	Amount	Type
1	15.08	n.a.	17.807	8.303	50.44	n.a.	BMB*
2	26.39	n.a.	11.582	8.157	49.56	n.a.	BMB*
Total:			29.389	16.461	100.00	0.000	

No.	Ret.Time min	Peak Name	Height mAU	Area mAU*min	Rel.Area %	Amount	Type
1	15.51	n.a.	231.496	106.570	97.68	n.a.	BMB*
2	27.28	n.a.	3.799	2.533	2.32	n.a.	BMB*
Total:			235.295	109.103	100.00	0.000	

(*S*)-2-(1,1,1-trifluoro-4-(4-fluorophenyl)-4-oxobutan-2-yl)pyridazin-3(*2H*)-one

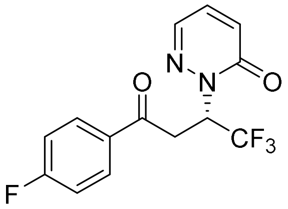

(+)-3ea; isolated yield: 30.1 mg (96%); colorless sticky oil; $[\alpha]_D^{20}$ = +269.9 (c = 1.0, $CHCl_3$); ^1H NMR (500 MHz, $CDCl_3$) δ 8.01-7.97 (m, 2H), 7.73-7.72 (m, 1H), 7.17-7.13 (m, 3H), 7.00 (dd, J = 9.5, 1.7 Hz, 1H), 6.43-6.39 (m, 1H), 4.26 (dd, J = 18.0, 10.9 Hz, 1H), 3.50 (dd, J = 18.0, 2.9 Hz, 1H); ^{13}C NMR (126 MHz, $CDCl_3$) δ 192.36, 166.16 (d, J = 256.1 Hz), 160.15, 136.71, 132.26 (d, J = 3.0 Hz), 131.15, 130.85 (d, J = 9.5 Hz), 130.31, 124.39 (q, J = 282.8 Hz), 115.99 (d, J = 22.0 Hz), 52.87 (q, J = 31.5 Hz), 35.25; ^{19}F NMR (376 MHz, $CDCl_3$) δ -73.17 (s), -103.67 (s);

Enantiomeric excess: 97%, determined by HPLC (Chiralpak AD-H, hexane/*i*-PrOH = 80/20; flow rate 1.0 ml/min; 25 °C; 254 nm), first peak: t_R = 10.19 min, second peak: t_R = 16.21 min; HRMS (ESI) m/z calcd. for $C_{14}H_{10}F_4N_2NaO_2$ $[M+Na]^+$ = 337.0571, found 337.0573.

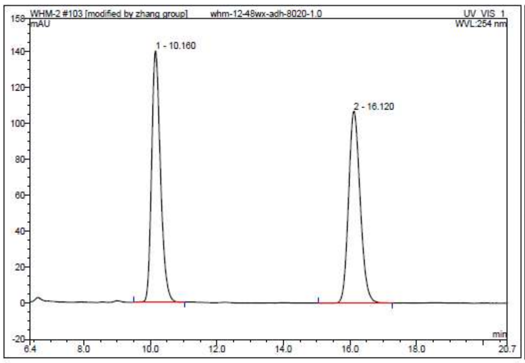

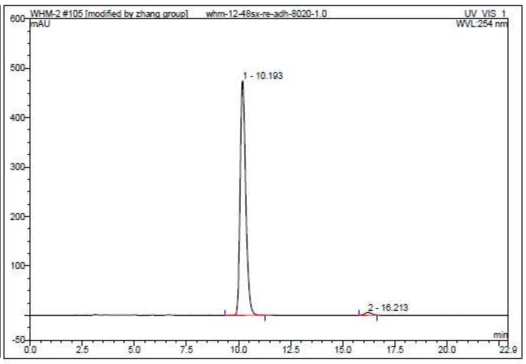

No.	Ret.Time min	Peak Name	Height mAU	Area mAU*min	Rel.Area %	Amount	Type
1	10.16	n.a.	139.790	43.183	50.07	n.a.	BMB*
2	16.12	n.a.	106.746	43.057	49.93	n.a.	BMB*
Total:			246.536	86.240	100.00	0.000	

No.	Ret.Time min	Peak Name	Height mAU	Area mAU*min	Rel.Area %	Amount	Type
1	10.19	n.a.	474.085	146.247	98.61	n.a.	BMB*
2	16.21	n.a.	5.559	2.062	1.39	n.a.	BMB*
Total:			479.645	148.308	100.00	0.000	

(*S*)-2-(4-(4-chlorophenyl)-1,1,1-trifluoro-4-oxobutan-2-yl)pyridazin-3(*2H*)- one

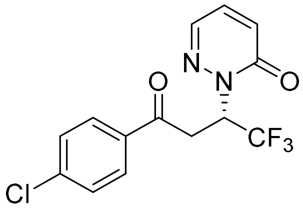

(+)-3fa; isolated yield: 32.0 mg (97%); colorless sticky oil; $[\alpha]_D^{20}$ = +235.2 (c = 1.0, $CHCl_3$); ^{1}H NMR (400 MHz, $CDCl_3$) δ 7.90 (d, J = 8.6 Hz, 2H), 7.73 (dd, J = 3.6, 1.6 Hz, 1H), 7.45 (d, J = 8.5 Hz, 2H), 7.16 (dd, J = 9.5, 3.7 Hz, 1H), 6.99 (dd, J = 9.5, 1.5 Hz, 1H), 6.45-6.36 (m, 1H), 4.25 (dd, J = 18.1, 10.9 Hz, 1H), 3.49 (dd, J = 18.1, 2.9 Hz, 1H); ^{13}C NMR (101 MHz, $CDCl_3$) δ 192.73, 160.08, 140.36, 136.66, 134.09, 131.10, 130.26, 129.49, 129.10, 124.32 (q, J = 282.9 Hz), 35.27, 52.83 (q, J = 31.5 Hz); ^{19}F NMR (376 MHz, $CDCl_3$) δ -73.17 (s). Enantiomeric excess: 98%, determined by HPLC (Chiralpak AD-H, hexane/*i*-PrOH = 80/20; flow rate 1.0 ml/min; 25 °C; 254 nm), first peak: t_R = 10.66 min, second peak: t_R = 17.35 min; HRMS (ESI) m/z calcd. for $C_{14}H_{10}ClF_3N_2NaO_2$ $[M+Na]^+$ = 353.0275, found 353.0280.

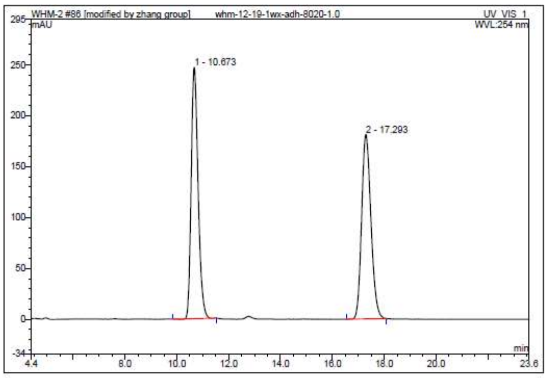

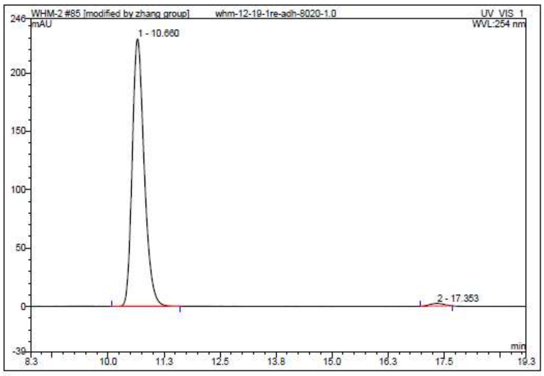

No.	Ret.Time min	Peak Name	Height mAU	Area mAU*min	Rel.Area %	Amount	Type
1	10.67	n.a.	246.832	76.155	50.24	n.a.	BMB*
2	17.29	n.a.	181.182	75.429	49.76	n.a.	BMB*
Total:			428.014	151.585	100.00	0.000	

No.	Ret.Time min	Peak Name	Height mAU	Area mAU*min	Rel.Area %	Amount	Type
1	10.66	n.a.	228.815	71.126	98.85	n.a.	BMB*
2	17.35	n.a.	2.329	0.828	1.15	n.a.	BMB*
Total:			231.144	71.954	100.00	0.000	

(*S*)-2-(4-(4-bromophenyl)-1,1,1-trifluoro-4-oxobutan-2-yl)pyridazin-3(*2H*)- one

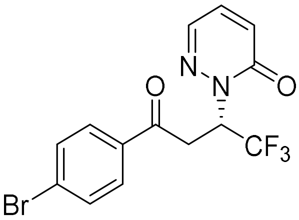

(+)-3ga; isolated yield: 36.4 mg (97%); colorless sticky oil; $[\alpha]_D^{20}$ = +226.1 (*c* = 1.0, $CHCl_3$); ^{1}H NMR (400 MHz, $CDCl_3$) δ 7.82 (d, *J* = 8.4 Hz, 2H), 7.73-7.72 (m, 1H), 7.63-7.61 (m, 2H), 7.15 (dd, *J* = 9.5, 3.7 Hz, 1H), 6.99 (d, *J* = 9.5 Hz, 1H), 6.44-6.36 (m, 1H), 4.24 (dd, *J* = 18.1, 10.9 Hz, 1H), 3.49 (dd, *J* = 18.1, 2.9 Hz, 1H); ^{13}C NMR (126 MHz, $CDCl_3$) δ 193.00, 160.15, 136.75, 134.49, 132.15, 131.19, 130.30, 129.62, 129.17, 124.36 (q, *J* = 282.8 Hz), 52.81 (q, *J* = 31.5 Hz), 35.29; ^{19}F NMR (376 MHz, $CDCl_3$) δ -73.16 (s); Enantiomeric excess: 97%, determined by HPLC (Chiralpak AD-H, hexane/*i*-PrOH = 80/20; flow rate 1.0 ml/min; 25 °C; 254 nm), first peak: t_R = 11.43 min, second peak: t_R = 18.57 min; HRMS (ESI) m/z calcd. for $C_{14}H_{10}BrF_3N_2NaO_2$ $[M+Na]^+$ = 396.9770, found 396.9773.

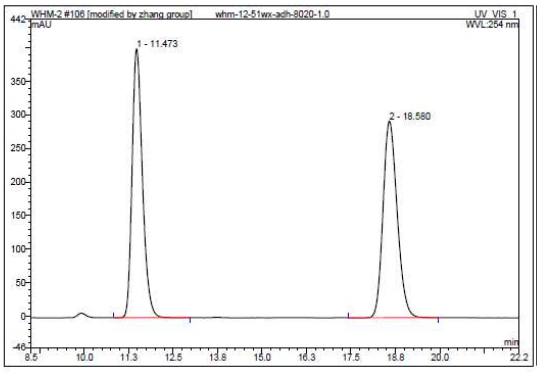

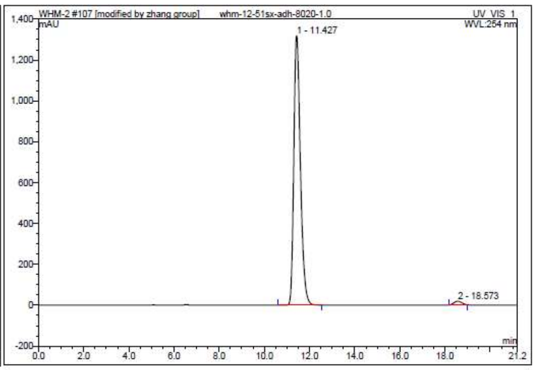

No.	Ret.Time min	Peak Name	Height mAU	Area mAU*min	Rel.Area %	Amount	Type
1	11.47	n.a.	400.487	134.896	49.98	n.a.	BMB*
2	18.58	n.a.	292.740	134.981	50.02	n.a.	BMB*
Total:			693.227	269.877	100.00	0.000	

No.	Ret.Time min	Peak Name	Height mAU	Area mAU*min	Rel.Area %	Amount	Type
1	11.43	n.a.	1317.814	445.903	98.43	n.a.	BMB*
2	18.57	n.a.	17.766	7.093	1.57	n.a.	BMB*
Total:			1335.580	452.997	100.00	0.000	

(*S*)-2-(1,1,1-trifluoro-4-(4-iodophenyl)-4-oxobutan-2-yl)pyridazin-3(*2H*)-one

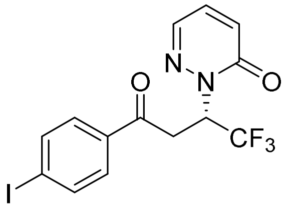

(+)-3ha; isolated yield: 41.8 mg (99%); white solid; $[\alpha]_D^{20}$ = +288.3 (*c* = 1.0, $CHCl_3$); ^{1}H NMR (400 MHz, $CDCl_3$) δ 7.84 (d, *J* = 8.5 Hz, 2H), 7.72 (dd, *J* = 3.6, 1.6 Hz, 1H), 7.66 (d, *J* = 8.5 Hz, 2H), 7.15 (dd, *J* = 9.5, 3.7 Hz, 1H), 6.99 (dd, *J* = 9.5, 1.6 Hz, 1H), 6.44-6.35 (m, 1H), 4.23 (dd, *J* = 18.1, 10.9 Hz, 1H), 3.47 (dd, *J* = 18.1, 3.0 Hz, 1H); ^{13}C NMR (126 MHz, $CDCl_3$) δ 193.25, 160.07, 138.08, 136.67, 134.94, 131.11, 130.24, 129.38, 124.29 (q, *J* = 282.8 Hz), 101.98, 52.73 (q, *J* = 31.5 Hz), 35.15; ^{19}F NMR (376 MHz, $CDCl_3$) δ -73.15 (s); Enantiomeric excess: 95%, determined by HPLC (Chiralpak AD-H, hexane/*i*-PrOH = 80/20; flow rate 1.0 ml/min; 25 °C; 254 nm), first peak: t_R = 12.58 min, second peak: t_R = 20.67 min; HRMS (ESI) m/z calcd. for $C_{14}H_{10}F_3IN_2NaO_2$ $[M+Na]^+$ = 444.9631, found 444.9631.

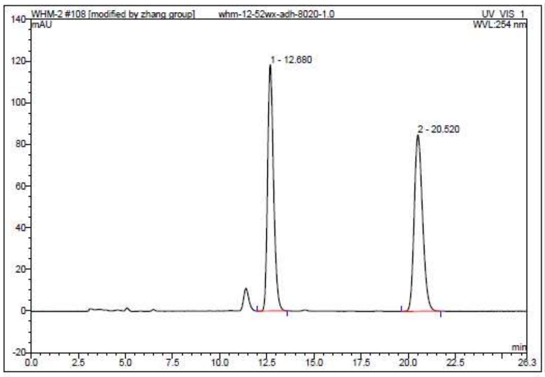

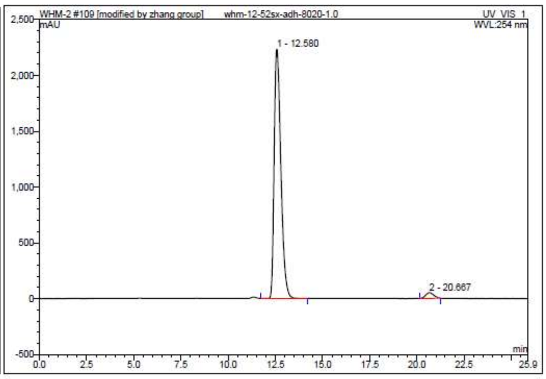

No.	Ret.Time min	Peak Name	Height mAU	Area mAU*min	Rel.Area %	Amount	Type
1	12.68	n.a.	118.159	43.297	49.84	n.a.	BMB*
2	20.52	n.a.	84.742	43.581	50.16	n.a.	BMB*
Total:			202.901	86.878	100.00	0.000	

No.	Ret.Time min	Peak Name	Height mAU	Area mAU*min	Rel.Area %	Amount	Type
1	12.58	n.a.	2234.596	923.252	97.35	n.a.	BMB*
2	20.67	n.a.	50.275	25.166	2.65	n.a.	BMB*
Total:			2284.871	948.417	100.00	0.000	

(*S*)-2-(1,1,1-trifluoro-4-(4-nitrophenyl)-4-oxobutan-2-yl)pyridazin-3(*2H*)-one

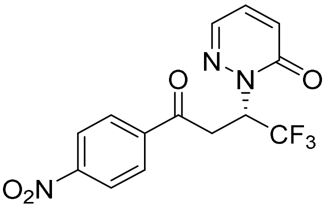

(+)-3ia; isolated yield: 33.4 mg (98%); colorless sticky oil; $[\alpha]_D^{20}$ = +288.2 (*c* = 1.0, $CHCl_3$); ^1H NMR (400 MHz, $CDCl_3$) δ 8.33 (d, *J* = 8.8 Hz, 2H), 8.14 (d, *J* = 8.8 Hz, 2H), 7.75 (dd, *J* = 3.6, 1.6 Hz, 1H), 7.19 (dd, *J* = 9.6, 3.7 Hz, 1H), 7.01 (dd, *J* = 9.6, 1.6 Hz, 1H), 6.46-6.38 (m, 1H), 4.33 (dd, *J* = 18.2, 10.8 Hz, 1H), 3.59 (dd, *J* = 18.3, 3.0 Hz, 1H); ^{13}C NMR (101 MHz, $CDCl_3$) δ 192.71, 160.07, 150.76, 140.07, 136.86, 131.26, 130.34, 129.24, 124.22 (q, *J* = 282.9 Hz), 124.02, 52.77 (q, *J* = 31.7 Hz), 35.89; ^{19}F NMR (376 MHz, $CDCl_3$) δ -73.19 (s); Enantiomeric excess: 97%, determined by HPLC (Chiralpak AD-H, hexane/*i*-PrOH = 80/20; flow rate 1.0 ml/min; 25 °C; 254 nm), first peak: t_R = 31.02 min, second peak: t_R = 32.74 min; HRMS (ESI) m/z calcd. for $C_{14}H_{10}F_3N_3NaO_4$ $[M+Na]^+$ = 364.0516, found 364.0515.

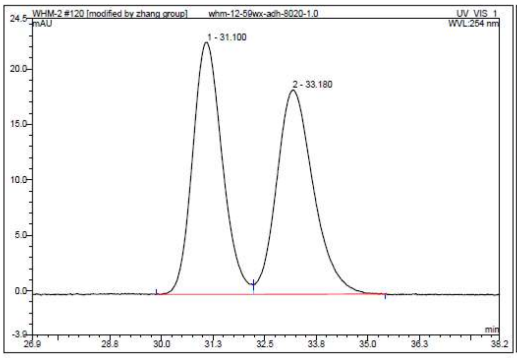

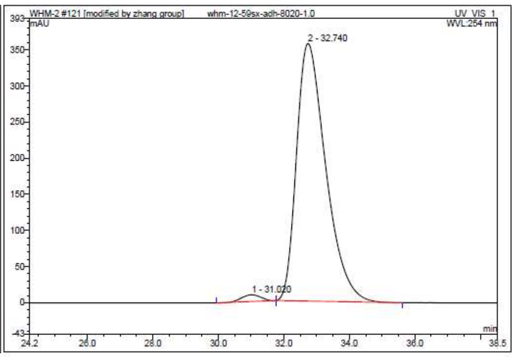

No.	Ret.Time min	Peak Name	Height mAU	Area mAU*min	Rel.Area %	Amount	Type
1	31.10	n.a.	22.728	18.935	49.92	n.a.	BM *
2	33.18	n.a.	18.434	18.998	50.08	n.a.	MB*
Total:			41.162	37.933	100.00	0.000	

No.	Ret.Time min	Peak Name	Height mAU	Area mAU*min	Rel.Area %	Amount	Type
1	31.02	n.a.	9.264	6.155	1.64	n.a.	BMb*
2	32.74	n.a.	356.223	368.278	98.36	n.a.	bMB*
Total:			365.487	374.433	100.00	0.000	

(*S*)-4-(4,4,4-trifluoro-3-(6-oxopyridazin-1(*6H*)-yl)butanoyl)benzonitrile

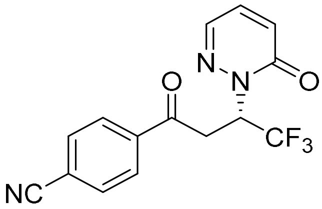

(+)-3ja; isolated yield: 31.1 mg (97%); colorless sticky oil; $[\alpha]_D^{20}$ = +276.8 (*c* = 1.0, $CHCl_3$); ^1H NMR (400 MHz, $CDCl_3$) δ 8.06 (d, *J* = 8.5 Hz, 2H), 7.80 (d, *J* = 8.5 Hz, 2H), 7.74 (dd, *J* = 3.6, 1.6 Hz, 1H), 7.18 (dd, *J* = 9.6, 3.7 Hz, 1H), 7.01 (dd, *J* = 9.6, 1.6 Hz, 1H), 6.45-6.36 (m, 1H), 4.29 (dd, *J* = 18.2, 10.8 Hz, 1H), 3.55 (dd, *J* = 18.2, 3.0 Hz, 1H); ^{13}C NMR (101 MHz, $CDCl_3$) δ 192.87, 160.07, 138.62, 136.83, 132.67, 131.25, 130.33, 128.57, 124.23 (q, *J* = 282.9 Hz), 117.65, 117.12, 52.76 (q, *J* = 31.6 Hz), 35.68; ^{19}F NMR (376 MHz, $CDCl_3$) δ -73.19 (s); Enantiomeric excess: 96%, determined by HPLC (Chiralpak AD-H, hexane/*i*-PrOH = 80/20; flow rate 1.0 ml/min; 25 °C; 254 nm), first peak: t_R = 23.54 min, second peak: t_R = 26.24 min; HRMS (ESI) m/z calcd. for $C_{15}H_{10}F_3N_3NaO_2$ $[M+Na]^+$ = 344.0617, found 344.0622.

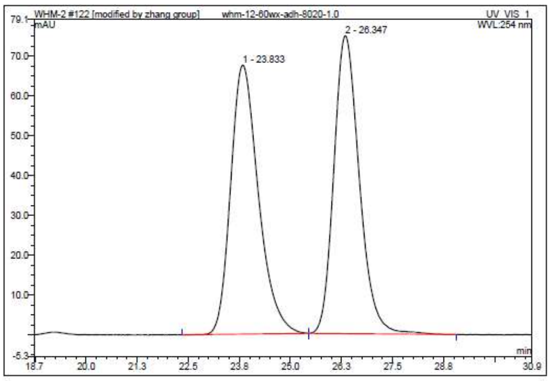

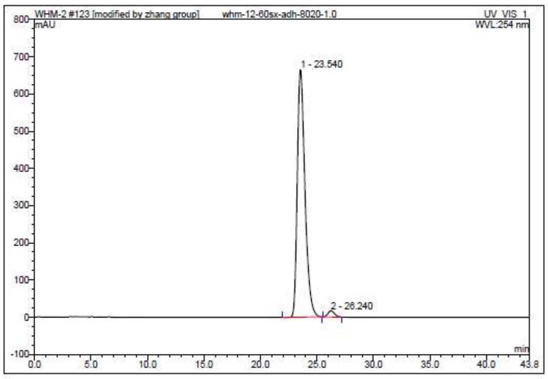

No.	Ret.Time min	Peak Name	Height mAU	Area mAU*min	Rel.Area %	Amount	Type
1	23.83	n.a.	67.435	53.011	49.57	n.a.	BMb*
2	26.35	n.a.	74.674	53.921	50.43	n.a.	bMB*
Total:			142.108	106.931	100.00	0.000	

No.	Ret.Time min	Peak Name	Height mAU	Area mAU*min	Rel.Area %	Amount	Type
1	23.54	n.a.	664.559	521.095	97.93	n.a.	BMB*
2	26.24	n.a.	16.364	11.029	2.07	n.a.	BMB*
Total:			680.923	532.124	100.00	0.000	

(*S*)-2-(1,1,1-trifluoro-4-(4-(methylsulfonyl)phenyl)-4-oxobutan-2-yl) pyridaz-in-3(*2H*)-one

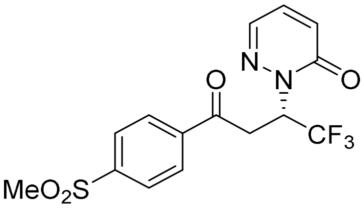

(+)-3ka; isolated yield: 29.2 mg (78%); white solid; $[\alpha]_D^{20}$ = +176.2 (*c* = 1.0, $CHCl_3$); ^{1}H NMR (400 MHz, $CDCl_3$) δ 8.14 (d, *J* = 8.5 Hz, 2H), 8.07 (d, *J* = 8.5 Hz, 2H), 7.73 (dd, *J* = 3.6, 1.6 Hz, 1H), 7.17 (dd, *J* = 9.6, 3.7 Hz, 1H), 7.01 (dd, *J* = 9.6, 1.6 Hz, 1H), 6.46-6.37 (m, 1H), 4.31 (dd, *J* = 18.2, 10.8 Hz, 1H), 3.56 (dd, *J* = 18.2, 3.0 Hz, 1H), 3.08 (s, 1H); ^{13}C NMR (101 MHz, $CDCl_3$) δ 192.95, 160.08, 144.94, 139.61, 136.81, 131.21, 130.37, 129.02, 127.99, 124.23 (q, *J* = 282.8 Hz), 52.81 (q, *J* = 31.7 Hz), 44.26, 35.83; ^{19}F NMR (376 MHz, $CDCl_3$) δ -73.17 (s); Enantiomeric excess: 93%, determined by HPLC (Chiralpak AD-H, hexane/*i*-PrOH = 90/10; flow rate 1.0 ml/min; 25 °C; 254 nm), first peak: t_R = 39.94 min, second peak: t_R = 50.88 min; HRMS (ESI) m/z calcd. for $C_{15}H_{13}F_3N_2NaO_4S$ $[M+Na]^+$ = 397.0440, found 397.0445.

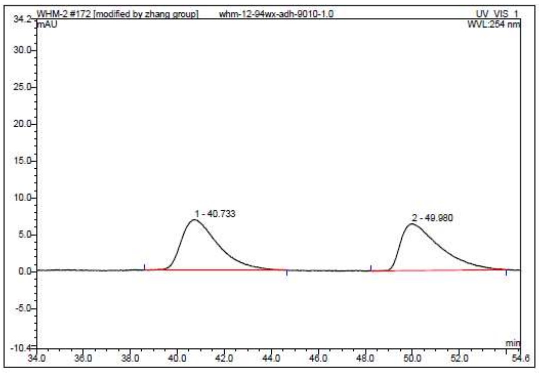

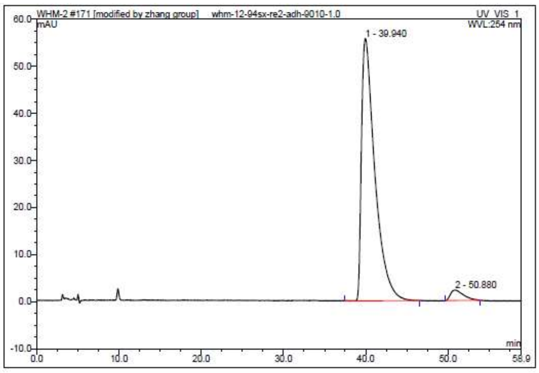

No.	Ret.Time min	Peak Name	Height mAU	Area mAU*min	Rel.Area %	Amount	Type
1	40.73	n.a.	6.824	12.324	50.24	n.a.	BMB*
2	49.98	n.a.	6.339	12.207	49.76	n.a.	BMB*
Total:			13.163	24.532	100.00	0.000	

No.	Ret.Time min	Peak Name	Height mAU	Area mAU*min	Rel.Area %	Amount	Type
1	39.94	n.a.	55.722	104.841	96.24	n.a.	BMB*
2	50.88	n.a.	2.252	4.097	3.76	n.a.	BMB*
Total:			57.974	108.938	100.00	0.000	

(*S*)-2-(1,1,1-trifluoro-4-oxo-4-(4-(trifluoromethyl)phenyl)butan-2-yl) pyridazin-3(*2H*)-one

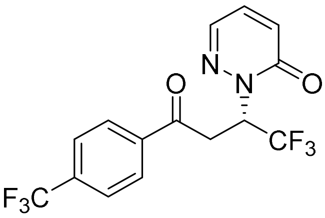

(+)-3la; isolated yield: 35.3 mg (97%); colorless sticky oil; $[\alpha]_D^{20}$ = +180.2 (*c* = 1.0, $CHCl_3$); ^{1}H NMR (500 MHz, $CDCl_3$) δ 8.08 (d, *J* = 8.1 Hz, 2H), 7.76-7.73 (m, 3H), 7.20-7.16 (m, 1H), 7.01 (dd, *J* = 9.6, 1.7 Hz, 1H), 6.46-6.39 (m, 1H), 4.32 (dd, *J* = 18.2, 10.9 Hz, 1H), 3.55 (dd, *J* = 18.2, 3.0 Hz, 1H); ^{13}C NMR (126 MHz, $CDCl_3$) δ 193.10, 160.09, 138.31, 136.76, 135.04 (q, *J* = 32.8 Hz), 131.18, 130.28, 128.47, 125.85 (q, *J* = 3.7 Hz), 124.25 (q, *J* = 282.8 Hz), 123.38 (q, *J* = 272.8 Hz), 52.72 (q, *J* = 31.6 Hz), 35.58; ^{19}F NMR (376 MHz, $CDCl_3$) δ -63.27 (s), -73.21 (s); Enantiomeric excess: 96%, determined by HPLC (Chiralpak AD-H, hexane/*i*-PrOH = 80/20; flow rate 1.0 ml/min; 25 °C; 254 nm), first peak: t_R = 9.23 min, second peak: t_R = 13.33 min; HRMS (ESI) m/z calcd. for $C_{15}H_{10}F_6N_2NaO_2$ [M+Na] $^+$ = 387.0539, found 387.0544.

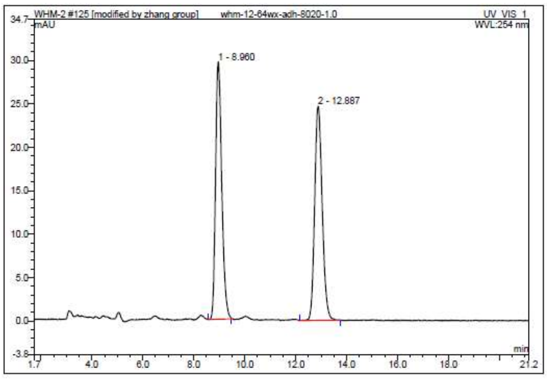

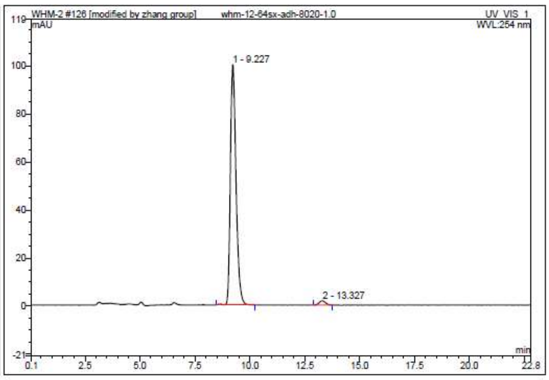

No.	Ret.Time min	Peak Name	Height mAU	Area mAU*min	Rel.Area %	Amount	Type
1	8.96	n.a.	29.624	8.352	49.71	n.a.	BMB*
2	12.89	n.a.	24.624	8.450	50.29	n.a.	BMB*
Total:			54.248	16.802	100.00	0.000	

No.	Ret.Time min	Peak Name	Height mAU	Area mAU*min	Rel.Area %	Amount	Type
1	9.23	n.a.	100.182	29.994	98.11	n.a.	BMB*
2	13.33	n.a.	1.765	0.579	1.89	n.a.	BMB*
Total:			101.947	30.574	100.00	0.000	

(*S*)-2-(1,1,1-trifluoro-4-(2-nitrophenyl)-4-oxobutan-2-yl)pyridazin-3(*2H*)-one

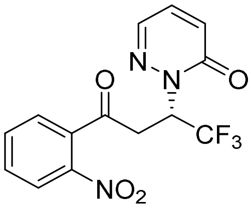

(+)-3ma; isolated yield: 31.4 mg (92%); white soild; $[\alpha]_D^{20}$ = +140,9 (*c* = 1.0, $CHCl_3$); ^{1}H NMR (500 MHz, $CDCl_3$) δ 8.12 (dd, *J* = 8.2, 0.6 Hz, 1H), 7.84 (dd, *J* = 3.7, 1.6 Hz, 1H), 7.76-7.73 (m, 1H), 7.66-7.63 (m, 1H), 7.40 (dd, *J* = 7.6, 1.2 Hz, 1H), 7.22 (dd, *J* = 9.5, 3.7 Hz, 1H), 7.00 (dd, *J* = 9.5, 1.6 Hz, 1H), 6.47-6.40 (m, 1H), 4.03 (dd, *J* = 18.3, 10.8 Hz, 1H), 3.47 (dd, *J* = 18.3, 2.8 Hz, 1H); ^{13}C NMR (126 MHz, $CDCl_3$) δ 196.41, 160.14, 145.58, 136.96, 136.34, 134.41, 131.40, 131.17, 130.25, 124.07 (q, *J* = 282.9 Hz), 127.26, 124.65, 52.50 (q, *J* = 31.9 Hz), 39.44; ^{19}F NMR (376 MHz, $CDCl_3$) δ -73.14 (s); Enantiomeric excess: 90%, determined by HPLC (Chiralpak AD-H, hexane/*i*-PrOH = 90/10; flow rate 1.0 ml/min; 25 °C; 254 nm), first peak: t_R = 12.75 min, second peak: t_R = 15.98 min; HRMS (ESI) m/z calcd. for $C_{14}H_{10}F_3N_3NaO_4$ $[M+Na]^+$ = 364.0516, found 364.0520.

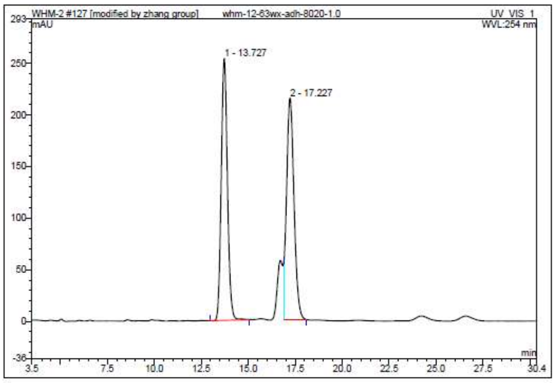

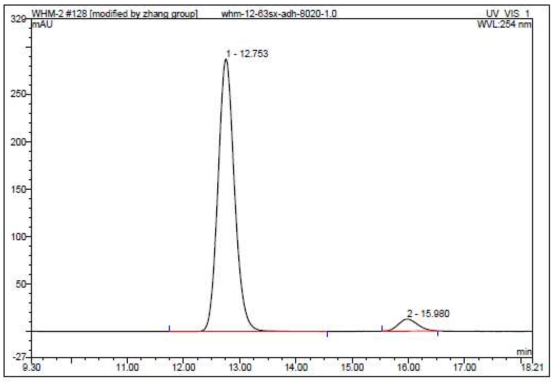

No.	Ret.Time min	Peak Name	Height mAU	Area mAU*min	Rel.Area %	Amount	Type
1	13.73	n.a.	253.462	96.431	49.17	n.a.	BMB*
2	17.23	n.a.	214.298	99.701	50.83	n.a.	MB*
Total:			467.760	196.131	100.00	0.000	

No.	Ret.Time min	Peak Name	Height mAU	Area mAU*min	Rel.Area %	Amount	Type
1	12.75	n.a.	286.762	97.340	95.13	n.a.	BMB*
2	15.98	n.a.	12.549	4.984	4.87	n.a.	BMB*
Total:			299.311	102.324	100.00	0.000	

(*S*)-2-(1,1,1-trifluoro-4-(3-nitrophenyl)-4-oxobutan-2-yl)pyridazin-3(*2H*)-one

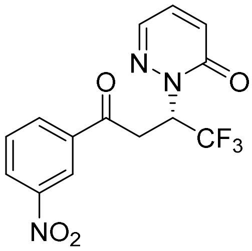

(+)-3na; isolated yield: 31.0 mg (91%); colorless sticky oil; $[\alpha]_D^{20}$ = +273.3 (*c* = 1.0, $CHCl_3$); ^{1}H NMR (400 MHz, $CDCl_3$) δ 8.78 (t, *J* = 1.7 Hz, 1H), 8.47-8.45 (m, 1H), 8.30 (d, *J* = 7.8 Hz, 1H), 7.76-7.71 (m, 2H), 7.19 (dd, *J* = 9.6, 3.7 Hz, 1H), 7.01 (dd, *J* = 9.6, 1.6 Hz, 1H), 6.48-6.39 (m, 1H), 4.35 (dd, *J* = 18.2, 10.8 Hz, 1H), 3.60 (dd, *J* = 18.2, 3.0 Hz, 1H); ^{13}C NMR (101 MHz, $CDCl_3$) δ 192.06, 160.03, 148.49, 136.94, 136.83, 133.57, 131.22, 130.28, 130.16, 127.99, 124.18 (q, *J* = 282.9 Hz), 122.96, 52.72 (q, *J* = 31.5 Hz), 35.63; ^{19}F NMR (376 MHz, $CDCl_3$) δ -73.17 (s); Enantiomeric excess: 93%, determined by HPLC (Chiralpak AD-H, hexane/*i*-PrOH = 80/20; flow rate 1.0 ml/min; 25 °C; 254 nm), first peak: t_R = 15.22 min, second peak: t_R = 23.71 min; HRMS (ESI) m/z calcd. for $C_{14}H_{10}F_3N_3NaO_4$ $[M+Na]^+$ = 364.0516, found 364.0522.

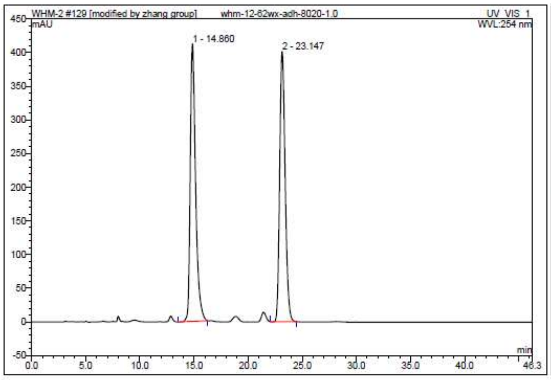

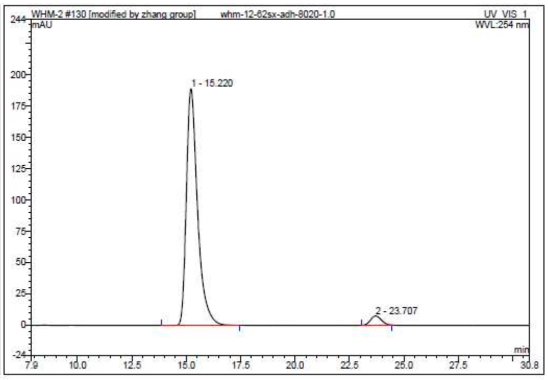

No.	Ret.Time min	Peak Name	Height mAU	Area mAU*min	Rel.Area %	Amount	Type
1	14.86	n.a.	412.457	236.584	50.13	n.a.	BMB*
2	23.15	n.a.	401.490	235.401	49.87	n.a.	BMB*
Total:			813.947	471.985	100.00	0.000	

No.	Ret.Time min	Peak Name	Height mAU	Area mAU*min	Rel.Area %	Amount	Type
1	15.22	n.a.	189.248	112.781	96.32	n.a.	BMB*
2	23.71	n.a.	7.471	4.309	3.68	n.a.	BMB*
Total:			196.719	117.090	100.00	0.000	

(*S*)-2-(4-(3,5-difluorophenyl)-1,1,1-trifluoro-4-oxobutan-2-yl)pyridazin-3(*2H*)-one

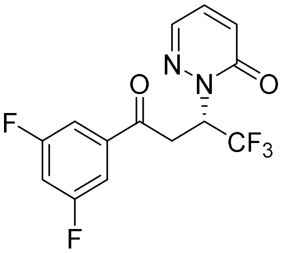

(+)-3oa; isolated yield: 32.9 mg (99%); colorless sticky oil; $[\alpha]_D^{20}$ = +188.3 (*c* = 1.0, $CHCl_3$); ^1H NMR (500 MHz, $CDCl_3$) δ 7.74 (dd, *J* = 3.6, 1.6 Hz, 1H), 7.49-7.44 (m, 2H), 7.17 (dd, *J* = 9.6, 3.7 Hz, 1H), 7.06 (tt, *J* = 8.3, 2.3 Hz, 1H), 7.01 (dd, *J* = 9.6, 1.6 Hz, 1H), 6.43-6.36 (m, 1H), 4.23 (dd, *J* = 18.2, 10.9 Hz, 1H), 3.48 (dd, *J* = 18.2, 2.9 Hz, 1H); ^{19}F NMR (376 MHz, $CDCl_3$) δ -73.24, -107.29; ^{13}C NMR (126 MHz, $CDCl_3$) δ 191.69, 164.12 (d, *J* = 11.7 Hz), 162.11 (d, *J* = 11.7 Hz), 160.10, 136.82, 131.22, 130.35, 138.51 (t, *J* = 7.6 Hz), 124.22 (q, *J* = 282.8 Hz), 52.71 (q, *J* = 31.5 Hz), 35.56; Enantiomeric excess: 96%, determined by HPLC (Chiralpak AD-H, hexane/*i*-PrOH = 80/20; flow rate 1.0 ml/min; 25 °C; 254 nm), first peak: t_R = 7.987 min, second peak: t_R = 12.556 min; HRMS (ESI) m/z calcd. for $C_{14}H_{10}F_5N_2O_2$ $[M+H]^+$ = 333.0657, found = 333.0652.

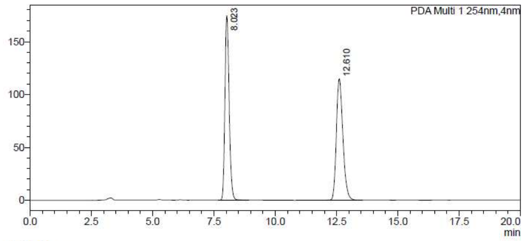

<Peak Table>
PDA Ch1 254nm

Peak#	Ret. Time	Area	Height	Area/Height	Height%	Area%
1	8.023	2096341	174597	12.007	60.319	49.686
2	12.610	2122830	114858	18.482	39.681	50.314
Total		4219171	289455		100.000	100.000

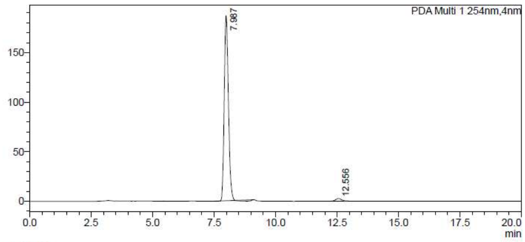

<Peak Table>
PDA Ch1 254nm

Peak#	Ret. Time	Area	Height	Area/Height	Height%	Area%
1	7.987	2172669	186810	11.630	98.623	97.967
2	12.556	45081	2609	17.279	1.377	2.033
Total		2217749	189419		100.000	100.000

(*S*)-2-(4-(3,4-dichlorophenyl)-1,1,1-trifluoro-4-oxobutan-2-yl)pyridazin-3(*2H*)-one

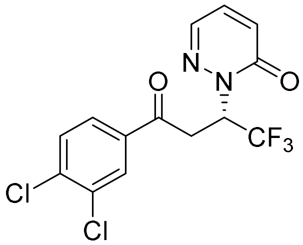

(+)-3pa; isolated yield: 35.0 mg (96%); colorless sticky oil; $[\alpha]_D^{20}$ = +480.8 (*c* = 1.0, $CHCl_3$); ^{1}H NMR (400 MHz, $CDCl_3$) δ 8.02 (d, *J* = 1.9 Hz, 1H), 7.79 (dd, *J* = 8.4, 2.0 Hz, 1H), 7.73 (dd, *J* = 3.6, 1.5 Hz, 1H), 7.57 (d, *J* = 8.4 Hz, 1H), 7.16 (dd, *J* = 9.5, 3.7 Hz, 1H), 7.00 (dd, *J* = 9.5, 1.5 Hz, 1H), 6.44-6.35 (m, 1H), 4.23 (dd, *J* = 18.1, 10.9 Hz, 1H), 3.48 (dd, *J* = 18.1, 3.0 Hz, 1H); ^{13}C NMR (101 MHz, $CDCl_3$) δ 191.86, 160.03, 138.52, 136.73, 135.26, 133.57, 131.14, 130.92, 130.28, 130.08, 127.07, 124.23 (q, *J* = 282.9 Hz), 52.78 (q, *J* = 31.6 Hz), 35.35; ^{19}F NMR (376 MHz, $CDCl_3$) δ -73.17 (s); Enantiomeric excess: 94%, determined by HPLC (Chiralpak AD-H, hexane/*i*-PrOH = 80/20; flow rate 1.0 ml/min; 25 °C; 254 nm), first peak: t_R = 9.46 min, second peak: t_R = 12.75 min; HRMS (ESI) m/z calcd. for $C_{14}H_9Cl_2F_3N_2NaO_2$ $[M+Na]^+$ = 386.9885, found = 386.9889.

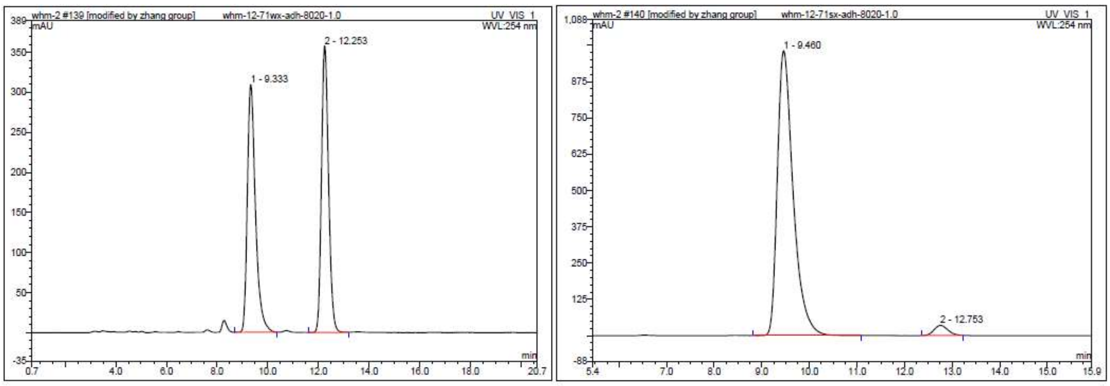

No.	Ret.Time min	Peak Name	Height mAU	Area mAU*min	Rel.Area %	Amount	Type
1	9.33	n.a.	309.037	115.009	49.93	n.a.	BMB*
2	12.25	n.a.	357.468	115.347	50.07	n.a.	BMB*
Total:			666.505	230.356	100.00	0.000	

No.	Ret.Time min	Peak Name	Height mAU	Area mAU*min	Rel.Area %	Amount	Type
1	9.46	n.a.	981.364	376.911	97.02	n.a.	BMB*
2	12.75	n.a.	34.547	11.563	2.98	n.a.	BMB*
Total:			1015.912	388.473	100.00	0.000	

(*S*)-2-(1,1,1-trifluoro-4-(naphthalen-1-yl)-4-oxobutan-2-yl)pyridazin-3(*2H*) -one

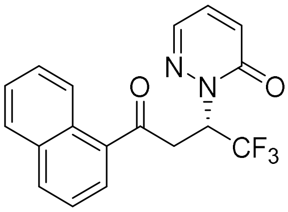

(+)-3qa; isolated yield: 33.9 mg (98%); colorless sticky oil; $[\alpha]_D^{20}$ = +148.4 (*c* = 1.0, $CHCl_3$); ^1H NMR (400 MHz, $CDCl_3$) δ 8.57-8.55 (m, 1H), 8.01 (t, *J* = 7.3 Hz, 2H), 7.85 (dd, *J* = 7.0, 2.3 Hz, 1H), 7.73 (dd, *J* = 3.6, 1.6 Hz, 1H), 7.56-7.49 (m, 3H), 7.13 (dd, *J* = 9.5, 3.7 Hz, 1H), 6.99 (dd, *J* = 9.5, 1.6 Hz, 1H), 6.54-6.45 (m, 1H), 4.39 (dd, *J* = 17.9, 11.0 Hz, 1H), 3.59 (dd, *J* = 17.9, 3.2 Hz, 1H); ^{13}C NMR (101 MHz, $CDCl_3$) δ 197.33, 160.22, 136.70, 133.97, 133.76, 131.11, 130.32, 130.12, 128.49, 128.48, 128.36, 126.69, 125.64, 124.48 (q, *J* = 282.9 Hz), 124.29, 53.35 (q, *J* = 31.5 Hz), 38.21, 29.69; ^{19}F NMR (376 MHz, $CDCl_3$) δ -73.03 (s); Enantiomeric excess: 91%, determined by HPLC (Chiralpak AD-H, hexane/*i*-PrOH = 90/10; flow rate 1.0 ml/min; 25 °C; 254 nm), first peak: t_R = 9.86 min, second peak: t_R = 11.99 min; HRMS (ESI) m/z calcd. for $C_{18}H_{13}F_3N_2NaO_2$ $[M+Na]^+$ = 369.0821, found 369.0819.

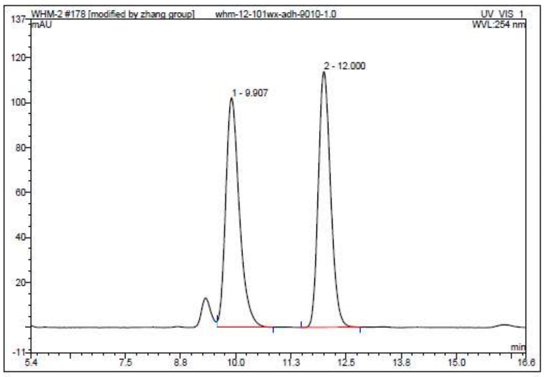

No.	Ret.Time min	Peak Name	Height mAU	Area mAU*min	Rel.Area %	Amount	Type
1	9.91	n.a.	102.073	36.523	49.79	n.a.	MB*
2	12.00	n.a.	113.816	36.831	50.21	n.a.	BMB*
Total:			215.888	73.354	100.00	0.000	

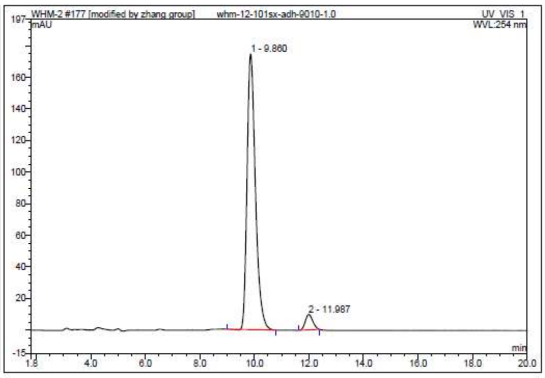

No.	Ret.Time min	Peak Name	Height mAU	Area mAU*min	Rel.Area %	Amount	Type
1	9.86	n.a.	174.491	61.731	95.24	n.a.	BMB*
2	11.99	n.a.	9.752	3.085	4.76	n.a.	BMB*
Total:			184.243	64.816	100.00	0.000	

(*S*)-2-(1,1,1-trifluoro-4-(naphthalen-2-yl)-4-oxobutan-2-yl)pyridazin-3(*2H*)-one

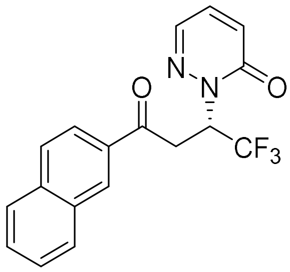

(+)-3ra; isolated yield: 33.9 mg (98%); colorless sticky oil; $[\alpha]_D^{20}$ = +450.6 (*c* = 0.33, $CHCl_3$); ^1H NMR (400 MHz, $CDCl_3$) δ 8.48 (s, 1H), 7.97 (d, *J* = 8.5 Hz, 2H), 7.87 (dd, *J* = 8.3, 4.2 Hz, 2H), 7.71 (dd, *J* = 3.6, 1.6 Hz, 1H), 7.63-7.55 (m, 2H), 7.12 (dd, *J* = 9.5, 3.7 Hz, 1H), 6.99 (dd, *J* = 9.5, 1.6 Hz, 1H), 6.53-6.44 (m, 1H), 4.43 (dd, *J* = 18.0, 10.9 Hz, 1H), 3.64 (dd, *J* = 18.0, 2.9 Hz, 1H); ^{13}C NMR (101 MHz, $CDCl_3$) δ 193.81, 160.20, 136.67, 135.87, 133.18, 132.41, 131.12, 130.28, 130.07, 129.63, 128.91, 128.71, 127.83, 127.06, 124.53 (q, *J* = 282.8 Hz), 123.51, 53.08 (q, *J* = 31.3 Hz), 35.39. ^{19}F NMR (376 MHz, $CDCl_3$) δ -73.03 (s); Enantiomeric excess: 94%, determined by HPLC (Chiralpak AD-H, hexane/*i*-PrOH = 80/20; flow rate 1.0 ml/min; 25 °C; 254 nm), first peak: t_R = 11.61 min, second peak: t_R = 15.35 min; HRMS (ESI) m/z calcd. for $C_{18}H_{13}F_3N_2NaO_2$ [M+Na] $^+$ = 369.0821, found 369.0822.

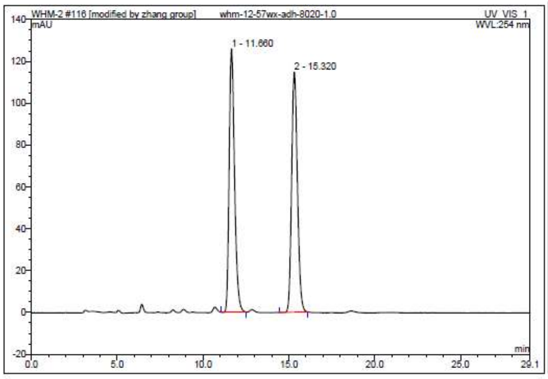

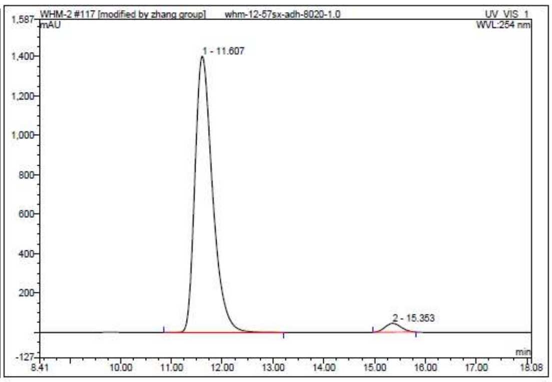

No.	Ret.Time min	Peak Name	Height mAU	Area mAU*min	Rel.Area %	Amount	Type
1	11.66	n.a.	125.797	45.418	50.33	n.a.	BMB*
2	15.32	n.a.	114.766	44.828	49.67	n.a.	BMB*
Total:			240.563	90.246	100.00	0.000	

No.	Ret.Time min	Peak Name	Height mAU	Area mAU*min	Rel.Area %	Amount	Type
1	11.61	n.a.	1401.567	563.560	97.16	n.a.	BMB*
2	15.35	n.a.	44.777	16.483	2.84	n.a.	BMB*
Total:			1446.344	580.042	100.00	0.000	

(*S*)-2-(4-(benzo[b]thiophen-2-yl)-1,1,1-trifluoro-4-oxobutan-2-yl)pyridazin -3(*2H*)-one

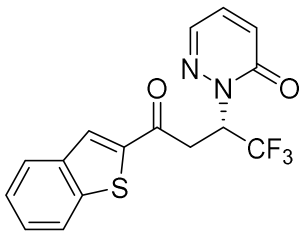

(+)-3sa; isolated yield: 34.5 mg (98%); colorless sticky oil; $[\alpha]_D^{20}$ = +395.1 (*c* = 1.0, $CHCl_3$); ^{1}H NMR (400 MHz, $CDCl_3$) δ 8.05 (s, 1H), 7.91 (d, *J* = 7.9 Hz, 1H), 7.85 (d, *J* = 8.0 Hz, 1H), 7.73 (dd, *J* = 3.5, 1.5 Hz, 1H), 7.49-7.40 (m, 2H), 7.13 (dd, *J* = 9.6, 3.7 Hz, 1H), 6.97 (dd, *J* = 9.5, 1.5 Hz, 1H), 6.47-6.39 (m, 1H), 4.31 (dd, *J* = 17.7, 10.9 Hz, 1H), 3.58 (dd, *J* = 17.7, 3.1 Hz, 1H); ^{13}C NMR (126 MHz, $CDCl_3$) δ 188.22, 160.02, 142.59, 142.07, 138.81, 136.75, 131.15, 130.24, 129.85, 127.84, 126.08, 125.21, 124.23 (q, *J* = 282.9 Hz), 122.92, 52.76 (q, *J* = 31.6 Hz), 35.70; ^{19}F NMR (376 MHz, $CDCl_3$) δ -73.11 (s); Enantiomeric excess: 94%, determined by HPLC (Chiralpak AD-H, hexane/*i*-PrOH = 80/20; flow rate 1.0 ml/min; 25 °C; 254 nm), first peak: t_R = 13.97 min, second peak: t_R = 19.45 min; HRMS (ESI) m/z calcd. for $C_{16}H_{11}F_3N_2NaO_2S$ $[M+Na]^+$ = 375.0386, found 375.0383.

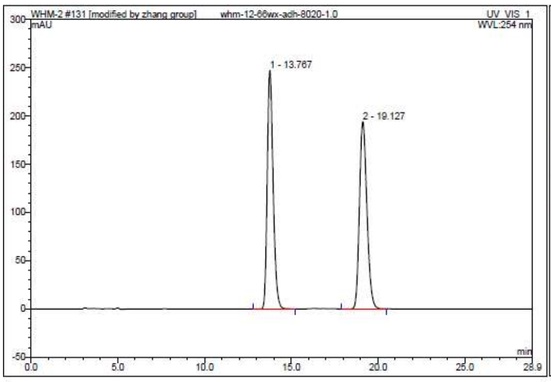

No.	Ret.Time min	Peak Name	Height mAU	Area mAU*min	Rel.Area %	Amount	Type
1	13.77	n.a.	247.420	97.428	50.09	n.a.	BMB*
2	19.13	n.a.	194.256	97.096	49.91	n.a.	BMB*
Total:			441.675	194.524	100.00	0.000	

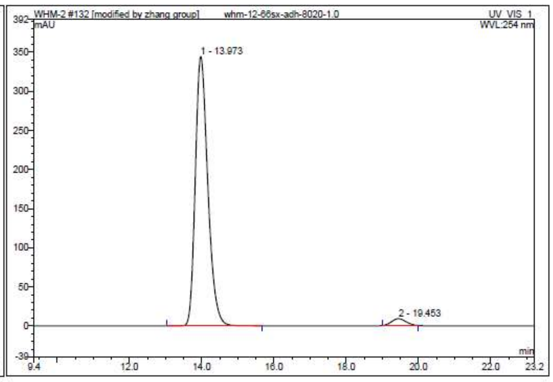

No.	Ret.Time min	Peak Name	Height mAU	Area mAU*min	Rel.Area %	Amount	Type
1	13.97	n.a.	344.437	138.213	97.17	n.a.	BMB*
2	19.45	n.a.	8.768	4.020	2.83	n.a.	BMB*
Total:			353.206	142.233	100.00	0.000	

(*S*)-2-(1,1,1-trifluoro-4-oxo-4-(thiophen-2-yl)butan-2-yl)pyridazin-3(*2H*)-one

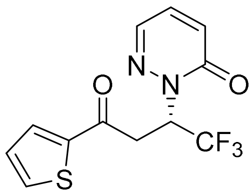

(+)-3ta; isolated yield: 29.9 mg (99%); colorless sticky oil; $[\alpha]_D^{20}$ = +205.9 (*c* = 1.0, $CHCl_3$); ^{1}H NMR (400 MHz, $CDCl_3$) δ 7.80 (dd, *J* = 3.8, 0.9 Hz, 1H), 7.74 (dd, *J* = 3.6, 1.6 Hz, 1H), 7.68 (dd, *J* = 4.9, 0.9 Hz, 1H), 7.17-7.13 (m, 2H), 6.97 (dd, *J* = 9.5, 1.6 Hz, 1H), 6.44-6.35 (m, 1H), 4.18 (dd, *J* = 17.6, 10.9 Hz, 1H), 3.49 (dd, *J* = 17.6, 3.1 Hz, 1H); ^{13}C NMR (126 MHz, $CDCl_3$) δ 186.63, 160.02, 142.72, 136.70, 134.55, 132.54, 131.13, 130.21, 128.26, 124.23 (q, *J* = 282.9 Hz), 52.73 (q, *J* = 31.5 Hz), 35.76; ^{19}F NMR (376 MHz, $CDCl_3$) δ -73.15 (s); Enantiomeric excess: 96%, determined by HPLC (Chiralpak AD-H, hexane/*i*-PrOH = 80/20; flow rate 1.0 ml/min; 25 °C; 254 nm), first peak: t_R = 11.51 min, second peak: t_R = 16.85 min; HRMS (ESI) m/z calcd. for $C_{12}H_9F_3N_2NaO_2S$ $[M+Na]^+$ = 325.0229, found 325.0229.

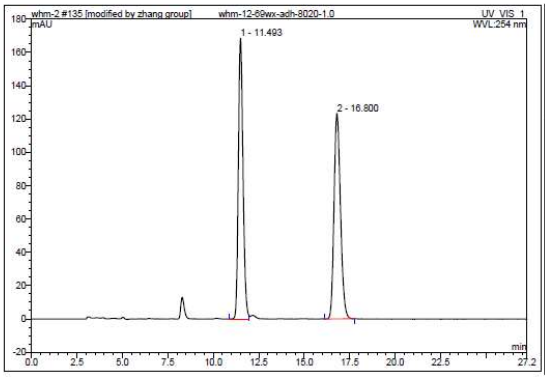

No.	Ret.Time min	Peak Name	Height mAU	Area mAU*min	Rel.Area %	Amount	Type
1	11.49	n.a.	168.477	50.132	49.74	n.a.	BM *
2	16.80	n.a.	123.385	50.653	50.26	n.a.	BMB*
Total:			291.862	100.785	100.00	0.000	

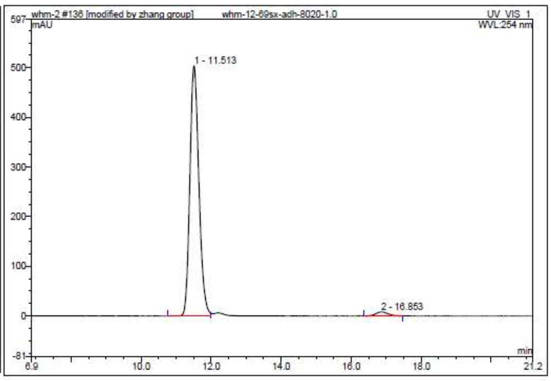

No.	Ret.Time min	Peak Name	Height mAU	Area mAU*min	Rel.Area %	Amount	Type
1	11.51	n.a.	503.754	150.280	97.89	n.a.	BM *
2	16.85	n.a.	8.013	3.231	2.11	n.a.	BMB*
Total:			511.766	153.512	100.00	0.000	

(*S*)-2-(4-(cyclohex-1-en-1-yl)-1,1,1-trifluoro-4-oxobutan-2-yl)pyridazin-3(*2H*) -one

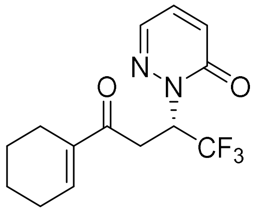

(+)-3ua; isolated yield: 16.5 mg (55%); colorless sticky oil; $[\alpha]_D^{20}$ = +249.6 (c = 0.33, $CHCl_3$); ^{1}H NMR (500 MHz, $CDCl_3$) δ 7.75 (dd, J = 3.7, 1.6 Hz, 1H), 7.16-7.13 (m, 1H), 7.00-6.96 (m, 2H), 6.30-6.23 (m, 1H), 3.91 (dd, J = 17.7, 11.0 Hz, 1H), 3.21 (dd, J = 17.7, 2.9 Hz, 1H), 2.27 (dd, J = 3.6, 2.2 Hz, 2H), 2.20-2.12 (m, 2H); ^{13}C NMR (126 MHz, $CDCl_3$) δ 194.64, 160.16, 141.39, 138.78, 136.51, 131.06, 131.01, 130.26, 124.49 (q, J = 282.7 Hz), 52.99 (q, J = 31.1 Hz), 33.90, 26.12, 22.92, 21.55 (d, J = 40.9 Hz); ^{19}F NMR (376 MHz, $CDCl_3$) δ -73.19 (s); Enantiomeric excess: 92%, determined by HPLC (Chiralpak AD-H, hexane/*i*-PrOH = 80/20; flow rate 1.0 ml/min; 25 °C; 254 nm), first peak: t_R = 8.09 min, second peak: t_R = 10.87 min; HRMS (ESI) m/z calcd. for $C_{14}H_{15}F_3N_2NaO_2$ $[M+Na]^+$ = 323.09787, found 323.0982.

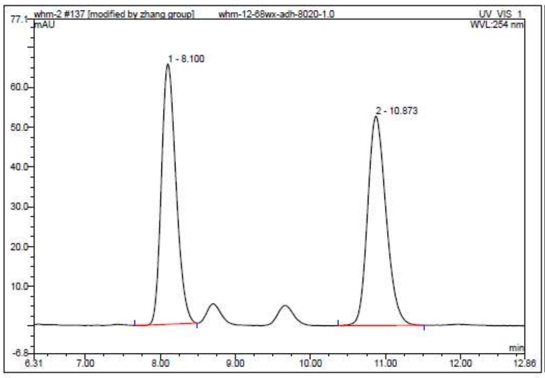

No.	Ret.Time min	Peak Name	Height mAU	Area mAU*min	Rel.Area %	Amount	Type
1	8.10	n.a.	65.432	14.770	49.54	n.a.	BMB*
2	10.87	n.a.	52.600	15.047	50.46	n.a.	BMB*
Total:			118.032	29.817	100.00	0.000	

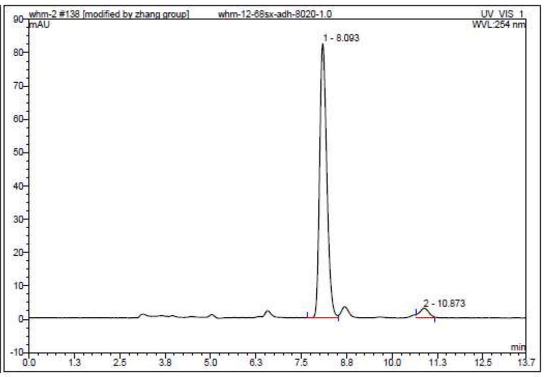

No.	Ret.Time min	Peak Name	Height mAU	Area mAU*min	Rel.Area %	Amount	Type
1	8.09	n.a.	82.214	18.745	96.01	n.a.	BM *
2	10.87	n.a.	2.708	0.780	3.99	n.a.	MB*
Total:			84.923	19.525	100.00	0.000	

(*S*)-2-(1,1,1,2,2-pentafluoro-5-oxo-5-phenylpentan-3-yl)pyridazin-3(*2H*)-One

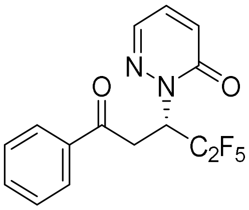

(+)-3va; isolated yield: 22.0 mg (64%); colorless sticky oil; $[\alpha]_D^{20}$ = +94.1 (*c* = 1.0, $CHCl_3$); ^1H NMR (400 MHz, $CDCl_3$) δ 7.96-7.94 (m, 2H), 7.72 (dd, *J* = 3.6, 1.6 Hz, 1H), 7.60 (t, *J* = 7.4 Hz, 1H), 7.47 (t, *J* = 7.7 Hz, 2H), 7.12 (dd, *J* = 9.6, 3.7 Hz, 1H), 6.97 (dd, *J* = 9.6, 1.7 Hz, 1H), 6.61-6.65 (m, 1H), 4.32 (dd, *J* = 18.1, 10.8 Hz, 1H), 3.55 (dd, *J* = 18.1, 2.3 Hz, 1H); ^{13}C NMR (126 MHz, $CDCl_3$) δ 193.90, 160.09, 136.80, 135.82, 133.85, 131.04, 130.13, 128.81, 128.14, 119.84 (t, *J* = 35.5 Hz), 117.56 (t, *J* = 35.4 Hz), 51.09 (t, J = 23.3 Hz), 35.21; ^{19}F NMR (376 MHz, CDCl3) δ -82.53 (s), -120.25 (dd, J = 1710.6, 275.5 Hz).; Enantiomeric excess: 83%, determined by HPLC (Chiralpak AD-H, hexane/*i*-PrOH = 80/20; flow rate 1.0 ml/min; 25 °C; 254 nm), first peak: t_R = 8.32 min, second peak: t_R = 8.97 min; HRMS (ESI) m/z calcd. for $C_{15}H_{11}F_5N_2NaO_2$ $[M+Na]^+$ = 369.0633, found 369.0628.

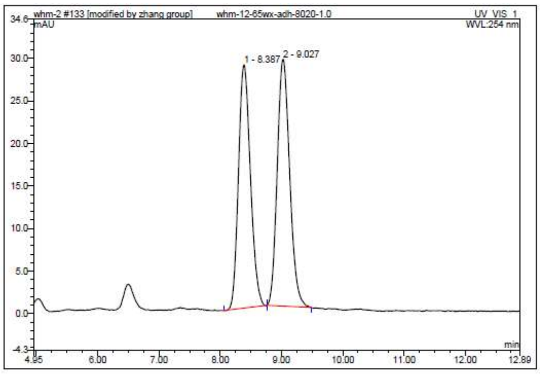

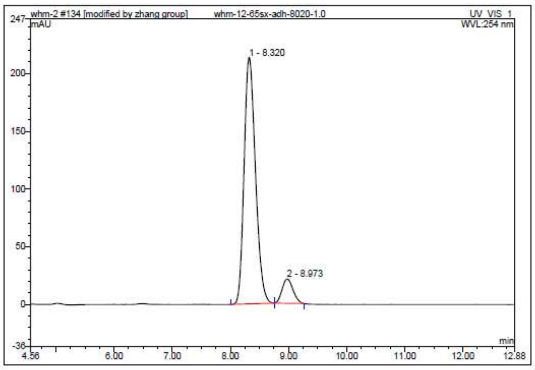

No.	Ret.Time min	Peak Name	Height mAU	Area mAU*min	Rel.Area %	Amount	Type
1	8.39	n.a.	28.610	6.459	48.56	n.a.	BMb*
2	9.03	n.a.	29.027	6.842	51.44	n.a.	bMB*
Total:			57.636	13.301	100.00	0.000	

No.	Ret.Time min	Peak Name	Height mAU	Area mAU*min	Rel.Area %	Amount	Type
1	8.32	n.a.	213.462	48.246	91.34	n.a.	BMb*
2	8.97	n.a.	21.126	4.571	8.66	n.a.	bMB*
Total:			234.588	52.817	100.00	0.000	

(*S*)-2-(4-(4-chlorophenyl)-1,1,1-trifluoro-4-oxobutan-2-yl)-6-methylpyri Dazin-3(*2H*)-one

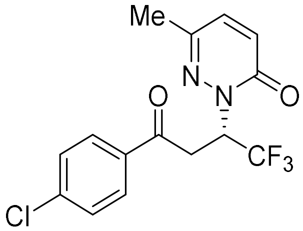

(+)-3fb; isolated yield: 33.1 mg (96%); colorless sticky oil; $[\alpha]_D^{20}$ = +254.6 (*c* = 1.0, $CHCl_3$); ^{1}H NMR (400 MHz, $CDCl_3$) δ 7.91 (d, *J* = 8.6 Hz, 1H), 7.45 (d, *J* = 8.6 Hz, 1H), 7.03 (d, *J* = 9.6 Hz, 1H), 6.91 (d, *J* = 9.6 Hz, 1H), 6.40-6.31 (m, 1H), 4.24 (dd, *J* = 17.9, 10.8 Hz, 1H), 3.45 (dd, *J* = 17.9, 3.0 Hz, 1H), 2.24 (s, 1H); ^{13}C NMR (126 MHz, $CDCl_3$) δ 192.95, 159.44, 144.97, 140.27, 134.23, 133.30, 129.97, 129.50, 129.08, 124.38 (q, *J* = 283.0 Hz), 52.63 (q, *J* = 31.4 Hz), 35.17, 20.89. ^{19}F NMR (376 MHz, $CDCl_3$) δ -73.10 (s); Enantiomeric excess: 96%, determined by HPLC (Chiralpak AD-H, hexane/*i*-PrOH = 80/20; flow rate 1.0 ml/min; 25 °C; 254 nm), first peak: t_R = 9.63 min, second peak: t_R = 12.43 min; HRMS (ESI) m/z calcd. for $C_{15}H_{12}ClF_3N_2NaO_2$ $[M+Na]^+$ = 367.0432, found 367.0435.

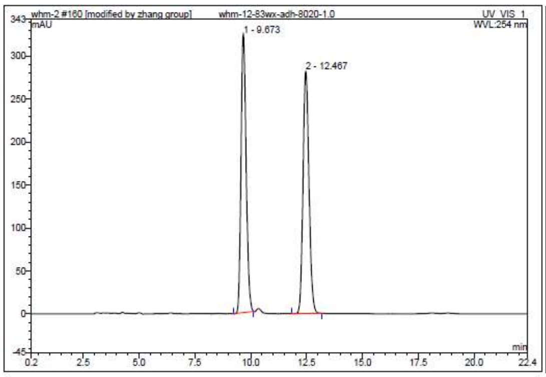

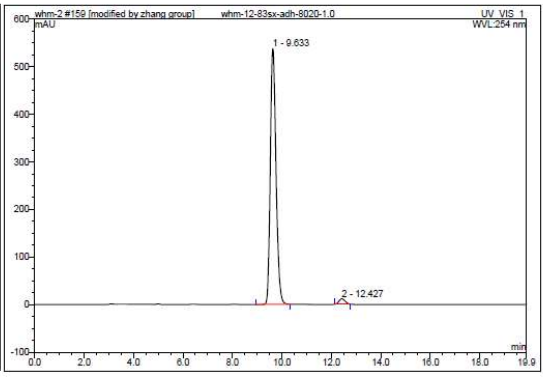

No.	Ret.Time min	Peak Name	Height mAU	Area mAU*min	Rel.Area %	Amount	Type
1	9.67	n.a.	325.255	86.363	49.70	n.a.	BMB*
2	12.47	n.a.	282.076	87.403	50.30	n.a.	BMB*
Total:			607.330	173.766	100.00	0.000	

No.	Ret.Time min	Peak Name	Height mAU	Area mAU*min	Rel.Area %	Amount	Type
1	9.63	n.a.	537.167	143.415	97.80	n.a.	BMB*
2	12.43	n.a.	11.214	3.231	2.20	n.a.	BMB*
Total:			548.381	146.646	100.00	0.000	

(*S*)-2-(4-(4-chlorophenyl)-1,1,1-trifluoro-4-oxobutan-2-yl)-6-phenylpyridazin-3(*2H*)-one

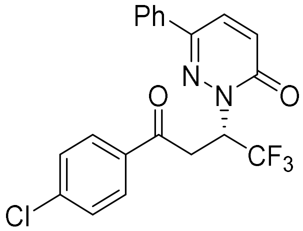

(+)-3fc; isolated yield: 39.1 mg (96%); colorless sticky oil; $[\alpha]_D^{20}$ = +50.3 (*c* = 1.0, $CHCl_3$); ^{1}H NMR (400 MHz, $CDCl_3$) δ 7.89 (d, *J* = 8.6 Hz, 2H), 7.66-7.63 (m, 3H), 7.42-7.40 (m, 5H), 7.08 (d, *J* = 9.8 Hz, 1H), 6.52-6.44 (m, 1H), 4.31 (dd, *J* = 17.9, 10.8 Hz, 1H), 3.55 (dd, *J* = 17.9, 3.0 Hz, 1H); ^{13}C NMR (126 MHz, $CDCl_3$) δ 192.89, 159.44, 145.04, 140.39, 134.30, 134.16, 130.45, 130.44, 129.83, 129.56, 129.14, 128.99, 125.95, 124.44 (q, *J* = 283.0 Hz), 53.13 (q, *J* = 31.4 Hz), 35.51; ^{19}F NMR (376 MHz, $CDCl_3$) δ -72.96 (s); Enantiomeric excess: 95%, determined by HPLC (Chiralpak AD-H, hexane/*i*-PrOH = 80/20; flow rate 1.0 ml/min; 25 °C; 254 nm), first peak: t_R = 10.19 min, second peak: t_R = 12.50 min; HRMS (ESI) m/z calcd. for $C_{20}H_{14}ClF_3N_2NaO_2$ $[M+Na]^+$ = 429.0588, found = 429.0592.

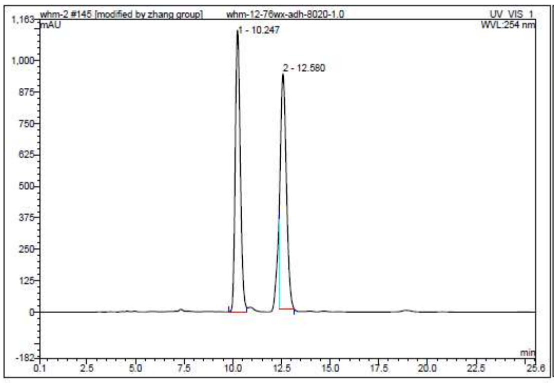

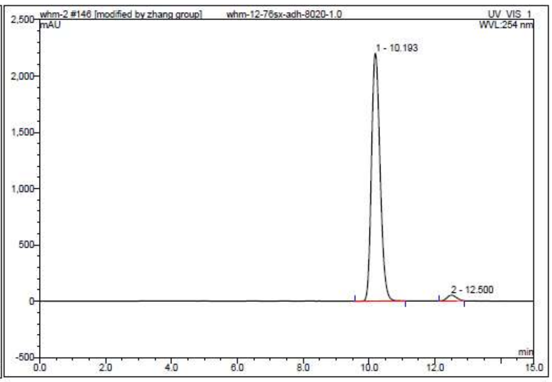

No.	Ret.Time min	Peak Name	Height mAU	Area mAU*min	Rel.Area %	Amount	Type
1	10.25	n.a.	1118.454	335.634	49.97	n.a.	BM *
2	12.58	n.a.	936.273	336.075	50.03	n.a.	MB*
Total:			2054.727	671.709	100.00	0.000	

No.	Ret.Time min	Peak Name	Height mAU	Area mAU*min	Rel.Area %	Amount	Type
1	10.19	n.a.	2201.054	664.655	97.52	n.a.	BMB*
2	12.50	n.a.	50.926	16.913	2.48	n.a.	BMB*
Total:			2251.980	681.567	100.00	0.000	

(*S*)-6-chloro-2-(4-(4-chlorophenyl)-1,1,1-trifluoro-4-oxobutan-2-yl)pyridaz-in-3(*2H*)-one

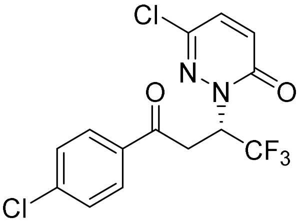

(+)-3fd; isolated yield: 33.9 mg (93%); colorless sticky oil; $[\alpha]_D^{20}$ = +178.8 (*c* = 1.0, $CHCl_3$); ^1H NMR (500 MHz, $CDCl_3$) δ 7.92-7.89 (m, 2H), 7.48-7.45 (m, 2H), 7.16-7.14 (m, 1H), 6.99 (d, *J* = 9.8 Hz, 1H), 6.33-6.26 (m, 1H), 4.18 (dd, *J* = 18.2, 10.9 Hz, 1H), 3.49 (dd, *J* = 18.2, 2.8 Hz, 1H); ^{13}C NMR (126 MHz, $CDCl_3$) δ 192.70, 158.48, 140.53, 138.33, 133.95, 133.90, 132.15, 129.54, 129.16, 124.00 (q, *J* = 282.9 Hz), 53.09 (q, *J* = 31.8 Hz), 35.11; ^{19}F NMR (376 MHz, $CDCl_3$) δ -73.22 (s); Enantiomeric excess: 92%, determined by HPLC (Chiralpak AD-H, hexane/*i*-PrOH = 80/20; flow rate 1.0 ml/min; 25 °C; 254 nm), first peak: t_R = 9.12 min, second peak: t_R = 11.43 min; HRMS (ESI) m/z calcd. for $C_{14}H_9Cl_2F_3N_2NaO_2$ $[M+Na]^+$ = 386.9885, found 386.9890.

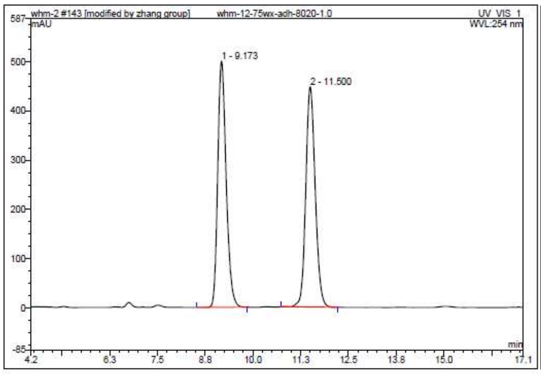

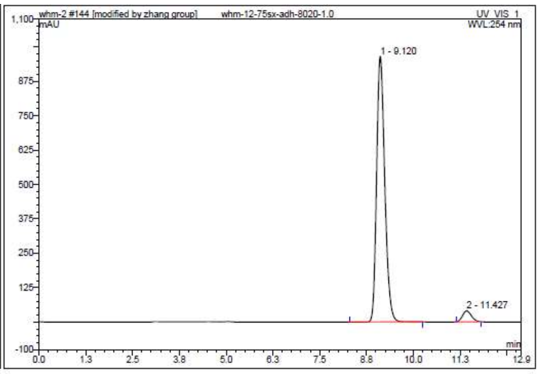

No.	Ret.Time min	Peak Name	Height mAU	Area mAU*min	Rel.Area %	Amount	Type
1	9.17	n.a.	499.984	127.851	49.94	n.a.	BMB*
2	11.50	n.a.	447.271	128.178	50.06	n.a.	BMB*
Total:			947.255	256.030	100.00	0.000	

No.	Ret.Time min	Peak Name	Height mAU	Area mAU*min	Rel.Area %	Amount	Type
1	9.12	n.a.	964.667	243.205	95.78	n.a.	BMB*
2	11.43	n.a.	39.708	10.725	4.22	n.a.	BMB*
Total:			1004.376	253.930	100.00	0.000	

Methyl (*S*)-1-(4-(4-chlorophenyl)-1,1,1-trifluoro-4-oxobutan-2-yl)-6-oxo-1,6-dihydropyridazine-3-carboxylate

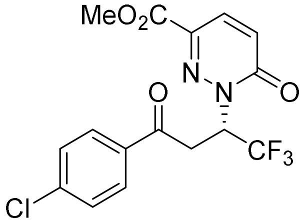

(+)-3fe; isolated yield: 37.0 mg (95%); white solid; $[\alpha]_D^{20}$ = +153.3 (*c* = 1.0, $CHCl_3$); ^{1}H NMR (400 MHz, $CDCl_3$) δ 7.91 (d, *J* = 8.6 Hz, 2H), 7.79 (d, *J* = 9.8 Hz, 1H), 7.46 (d, *J* = 8.6 Hz, 2H), 7.03 (d, *J* = 9.8 Hz, 1H), 6.46-6.37 (m, 1H), 4.33 (dd, *J* = 18.1, 11.1 Hz, 1H), 3.88 (s, 3H), 3.52 (dd, *J* = 18.1, 2.8 Hz, 1H); ^{13}C NMR (101 MHz, $CDCl_3$) δ 192.94, 162.10, 159.37, 140.53, 136.74, 134.09, 131.19, 129.63, 129.14, 124.06 (q, *J* = 282.9 Hz), 53.79 (q, *J* = 31.8 Hz), 35.32, 29.68; ^{19}F NMR (376 MHz, $CDCl_3$) δ -73.05 (s); Enantiomeric excess: 90%, determined by HPLC (Chiralpak AD-H, hexane/*i*-PrOH = 90/10; flow rate 1.0 ml/min; 25 °C; 254 nm), first peak: t_R = 10.91 min, second peak: t_R = 14.53 min; HRMS (ESI) m/z calcd. for $C_{16}H_{12}ClF_3N_2NaO_4$ $[M+Na]^+$ = 411.0330, found 411.0330.

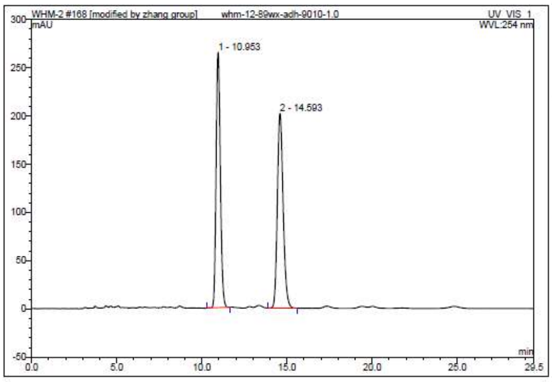

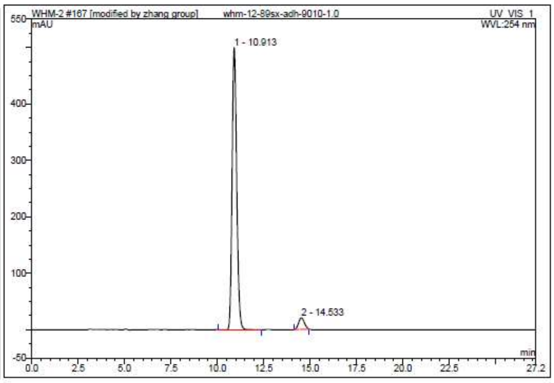

No.	Ret.Time min	Peak Name	Height mAU	Area mAU*min	Rel.Area %	Amount	Type
1	10.95	n.a.	264.911	76.646	49.11	n.a.	BMB*
2	14.59	n.a.	202.013	79.427	50.89	n.a.	BMB*
Total:			466.924	156.073	100.00	0.000	

No.	Ret.Time min	Peak Name	Height mAU	Area mAU*min	Rel.Area %	Amount	Type
1	10.91	n.a.	499.378	143.894	95.18	n.a.	BMB*
2	14.53	n.a.	20.427	7.285	4.82	n.a.	BMB*
Total:			519.805	151.179	100.00	0.000	

(*S*)-5-chloro-2-(4-(4-chlorophenyl)-1,1,1-trifluoro-4-oxobutan-2-yl)pyridaz-in-3(*2H*)-one

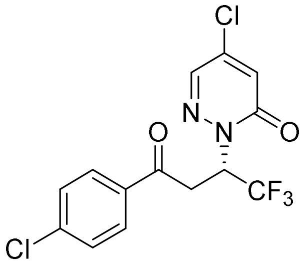

(+)-3ff; isolated yield: 35.8 mg (98%); colorless sticky oil; $[\alpha]_D^{20}$ = +240.5 (*c* = 1.0, $CHCl_3$); ^{1}H NMR (400 MHz, $CDCl_3$) δ 7.91-7.88 (m, 2H), 7.68 (d, *J* = 2.4 Hz, 1H), 7.47-7.45 (m, 2H), 7.04 (d, *J* = 2.4 Hz, 1H), 6.35-6.26 (m, 1H), 4.21 (dd, *J* = 18.2, 11.1 Hz, 1H), 3.51 (dd, *J* = 18.2, 2.9 Hz, 1H); ^{13}C NMR (101 MHz, $CDCl_3$) δ 192.56, 158.74, 140.53, 139.56, 136.99, 133.91, 129.48, 129.16, 127.52, 124.10 (d, *J* = 282.7 Hz), 52.95 (q, *J* = 31.7 Hz), 35.14; ^{19}F NMR (376 MHz, $CDCl_3$) δ -73.20 (s); Enantiomeric excess: 99%, determined by HPLC (Chiralpak AD-H, hexane/*i*-PrOH = 80/20; flow rate 1.0 ml/min; 25 °C; 254 nm), first peak: t_R = 9.19 min, second peak: t_R = 16.77 min; HRMS (ESI) m/z calcd. for $C_{14}H_9Cl_2F_3N_2NaO_2$ [M+Na] $^+$ = 386.9885, found 386.9888.

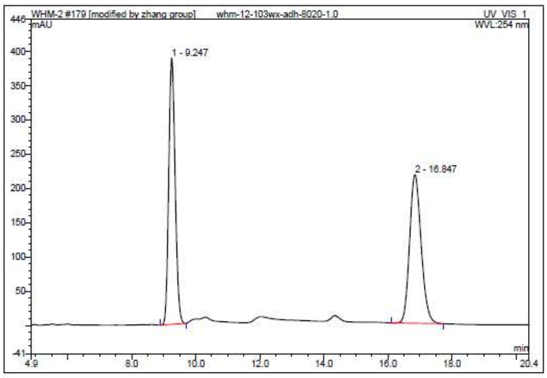

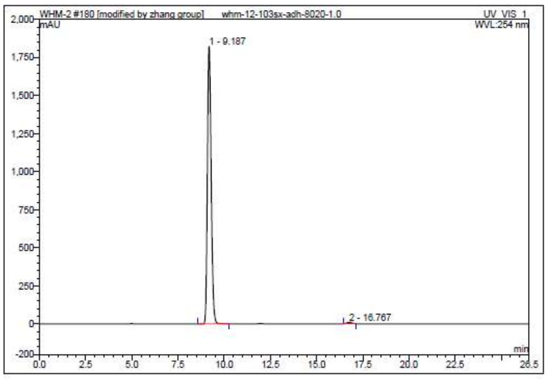

No.	Ret.Time min	Peak Name	Height mAU	Area mAU*min	Rel.Area %	Amount	Type
1	9.25	n.a.	388.099	89.899	49.85	n.a.	BMB*
2	16.85	n.a.	216.742	90.434	50.15	n.a.	BMB*
Total:			604.842	180.332	100.00	0.000	

No.	Ret.Time min	Peak Name	Height mAU	Area mAU*min	Rel.Area %	Amount	Type
1	9.19	n.a.	1821.961	431.870	99.23	n.a.	BMB*
2	16.77	n.a.	9.289	3.343	0.77	n.a.	BMB*
Total:			1831.250	435.213	100.00	0.000	

(*S*)-4,5-dibromo-2-(4-(4-chlorophenyl)-1,1,1-trifluoro-4-oxobutan-2-yl)pyridazin-3(*2H*)-one

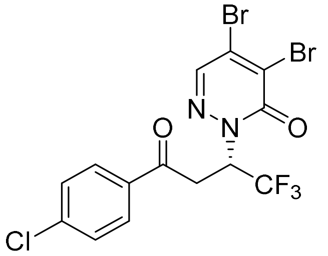

(+)-3fg; isolated yield: 47.8 mg (98%); white solid; $[\alpha]_D^{20}$ = +223.7 (*c* = 1.0, $CHCl_3$); ^{1}H NMR (500 MHz, $CDCl_3$) δ 7.90-7.88 (m, 2H), 7.75 (s, 1H), 7.46 (d, *J* = 8.5 Hz, 2H), 6.32-6.25 (m, 1H), 4.22 (dd, *J* = 18.2, 11.1 Hz, 1H), 3.52 (dd, *J* = 18.2, 2.9 Hz, 1H); ^{13}C NMR (126 MHz, $CDCl_3$) δ 192.47, 156.57, 140.60, 137.92, 133.78, 130.98, 130.92, 129.49, 129.18, 123.94 (q, *J* = 282.9 Hz), 54.57 (q, *J* = 31.7 Hz), 35.19; ^{19}F NMR (376 MHz, $CDCl_3$) δ -73.06 (s); Enantiomeric excess: 94%, determined by HPLC (Chiralpak AD-H, hexane/*i*-PrOH = 90/10; flow rate 1.0 ml/min; 25 °C; 254 nm), first peak: t_R = 20.53 min, second peak: t_R = 26.39 min; HRMS (ESI) m/z calcd. for $C_{14}H_8Br_2ClF_3N_2NaO_2$ [M+Na] $^+$ = 508.8485, found 508.8486.

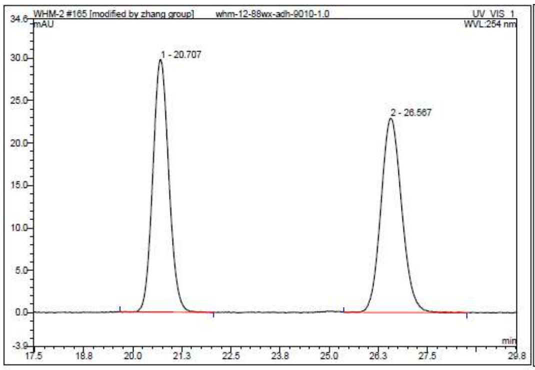

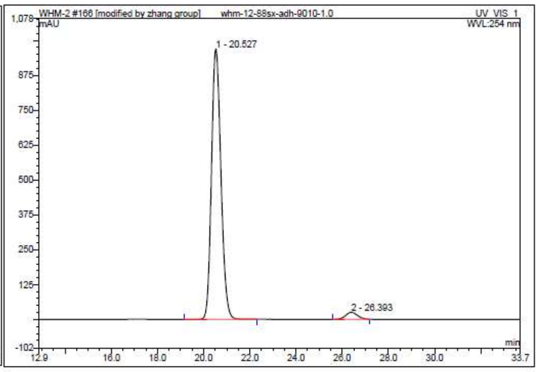

No.	Ret.Time min	Peak Name	Height mAU	Area mAU*min	Rel.Area %	Amount	Type
1	20.71	n.a.	29.768	14.019	49.88	n.a.	BMB*
2	26.57	n.a.	22.873	14.086	50.12	n.a.	BMB*
Total:			52.641	28.105	100.00	0.000	

No.	Ret.Time min	Peak Name	Height mAU	Area mAU*min	Rel.Area %	Amount	Type
1	20.53	n.a.	968.503	458.248	96.88	n.a.	BMB*
2	26.39	n.a.	24.904	14.739	3.12	n.a.	BMB*
Total:			993.407	472.987	100.00	0.000	

Methyl (*S*)-4-(4-chlorophenyl)-4-oxo-2-(6-oxopyridazin-1(*6H*)-yl)butanoate

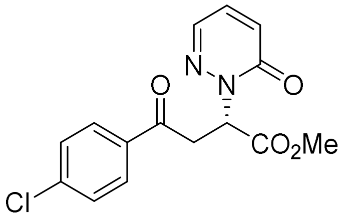

(+)-5aa; isolated yield: 27.9 mg (87%); colorless sticky oil; $[\alpha]_D^{20}$ = +16.0 (*c* = 1.0, $CHCl_3$); ^1H NMR (400 MHz, $CDCl_3$) δ 7.92 (d, *J* = 8.6 Hz, 2H), 7.74 (dd, *J* = 3.7, 1.6 Hz, 1H), 7.44 (d, *J* = 8.5 Hz, 2H), 7.20 (dd, *J* = 9.5, 3.8 Hz, 1H), 6.97 (dd, *J* = 9.5, 1.6 Hz, 1H), 6.11 (dd, *J* = 7.8, 5.6 Hz, 1H), 3.91 (dd, *J* = 17.7, 5.6 Hz, 1H), 3.81 (d, *J* = 7.9 Hz, 1H), 3.76 (s, 3H); ^{13}C NMR (101 MHz, $CDCl_3$) δ 194.41, 169.50, 160.11, 139.97, 136.32, 134.59, 131.46, 130.10, 129.59, 129.01, 58.57, 52.96, 38.13; Enantiomeric excess: 84%, determined by HPLC (Chiralpak AD-H, hexane/*i*-PrOH = 70/30; flow rate 1.0 ml/min; 25 °C; 254 nm), first peak: t_R = 17.62 min, second peak: t_R = 21.70 min; HRMS (ESI) m/z calcd. for $C_{15}H_{13}ClN_2NaO_4$ [M+Na] $^+$ = 343.0456, found 343.0453.

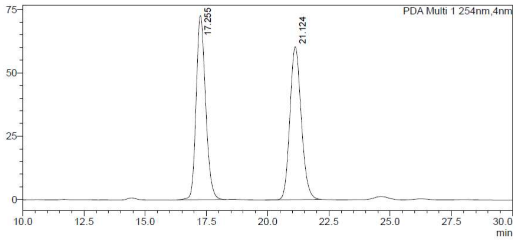

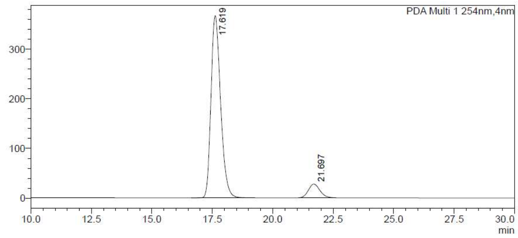

<Peak Table>
PDA Ch1 254nm

Peak#	Ret. Time	Area	Height	Area/Height	Height%	Area%
1	17.255	1947641	72727	26.780	54.630	49.988
2	21.124	1948553	60399	32.261	45.370	50.012
Total		3896194	133126		100.000	100.000

<Peak Table>
PDA Ch1 254nm

Peak#	Ret. Time	Area	Height	Area/Height	Height%	Area%
1	17.619	10114273	366634	27.587	93.040	91.900
2	21.697	891436	27426	32.503	6.960	8.100
Total		11005709	394060		100.000	100.000

Benzyl (*S*)-4-oxo-2-(6-oxopyridazin-1(*6H*)-yl)-4-phenylbutanoate

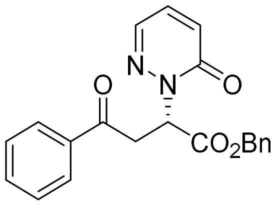

(+)-5ba; isolated yield: 25.0 mg (69%); colorless sticky oil; $[\alpha]_D^{20}$ = +7.4 (*c* = 1.0, $CHCl_3$); ^{1}H NMR (400 MHz, $CDCl_3$) δ 7.96 (d, *J* = 7.6 Hz, 2H), 7.71-7.70 (m, 1H), 7.58 (t, *J* = 7.4 Hz, 1H), 7.46 (t, *J* = 7.7 Hz, 2H), 7.34-7.26 (m, 5H), 7.17 (dd, *J* = 9.5, 3.8 Hz, 1H), 6.97 (dd, *J* = 9.4, 1.3 Hz, 1H), 6.20 (t, *J* = 6.7 Hz, 1H), 5.25-5.18 (m, 2H), 3.91 (d, *J* = 6.5 Hz, 2H); ^{13}C NMR (101 MHz, $CDCl_3$) δ 195.51, 169.14, 160.20, 136.23, 136.20, 135.19, 133.52, 131.43, 130.06, 128.69, 128.54, 128.32, 128.20, 128.03, 67.59, 58.62, 38.11; Enantiomeric excess: 87%, determined by HPLC (Chiralpak AD-H, hexane/*i*-PrOH = 70/30; flow rate 1.0 ml/min; 25 °C; 254 nm), first peak: t_R = 21.07 min, second peak: t_R = 27.84 min; HRMS (ESI) m/z calcd. for $C_{21}H_{18}N_2NaO_4$ [M+Na]$^+$ = 385.1159, found 385.1164.

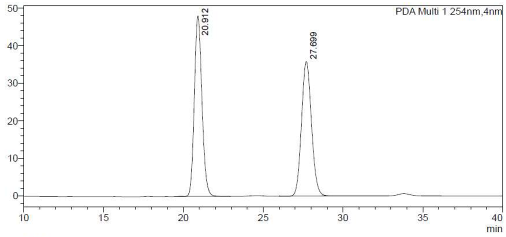

<Peak Table>

PDA Ch1 254nm

Peak#	Ret. Time	Area	Height	Area/Height	Height%	Area%
1	20.912	1593094	48044	33.159	57.275	50.539
2	27.699	1559090	35839	43.503	42.725	49.461
Total		3152184	83883		100.000	100.000

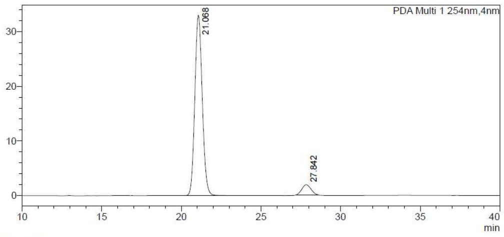

<Peak Table>

PDA Ch1 254nm

Peak#	Ret. Time	Area	Height	Area/Height	Height%	Area%
1	21.068	1070397	33004	32.432	94.673	93.619
2	27.842	72957	1857	39.286	5.327	6.381
Total		1143354	34861		100.000	100.000

Ethyl (*S*)-4-oxo-2-(6-oxopyridazin-1(*6H*)-yl)-4-phenylbutanoate

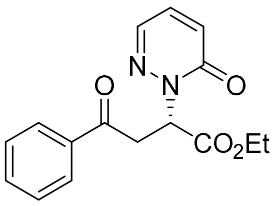

(+)-5ca; isolated yield: 27.0 mg (90%); colorless sticky oil; $[\alpha]_D^{20}$ = +1.9 (*c* = 1.0, $CHCl_3$); ^{1}H NMR (400 MHz, $CDCl_3$) δ 7.99 (d, *J* = 7.5 Hz, 2H), 7.74 (d, *J* = 2.3 Hz, 1H), 7.59 (t, *J* = 7.3 Hz, 1H), 7.48 (t, *J* = 7.6 Hz, 2H), 7.19 (dd, *J* = 9.5, 3.8 Hz, 1H), 6.98 (d, *J* = 9.4 Hz, 1H), 6.12 (dd, *J* = 7.3, 6.1 Hz, 1H), 4.23 (q, *J* = 6.8 Hz, 2H), 3.90 (t, *J* = 6.0 Hz, 2H), 1.24 (t, *J* = 7.1 Hz, 3H); ^{13}C NMR (101 MHz, $CDCl_3$) δ 195.63,

169.21, 160.20, 136.24, 136.21, 133.50, 131.42, 130.05, 128.69, 128.19, 62.07, 58.66, 38.13, 14.05; Enantiomeric excess: 81%, determined by HPLC (Chiralpak AD-H, hexane/*i*-PrOH = 70/30; flow rate 1.0 ml/min; 25 °C; 254 nm), first peak: t_R = 11.57 min, second peak: t_R = 13.84 min; HRMS (ESI) m/z calcd. for $C_{16}H_{16}N_2NaO_4$ [M+Na] $^+$ = 323.1002, found 323.1006.

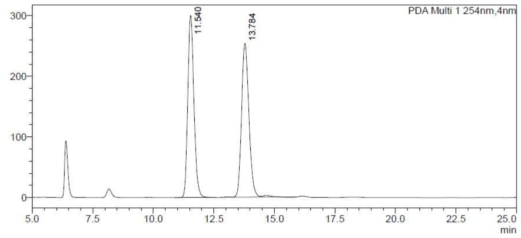

<Peak Table>

PDA Ch1 254nm

Peak#	Ret. Time	Area	Height	Area/Height	Height%	Area%
1	11.540	5298874	300798	17.616	54.217	49.463
2	13.784	5413830	254006	21.314	45.783	50.537
Total		10712704	554803		100.000	100.000

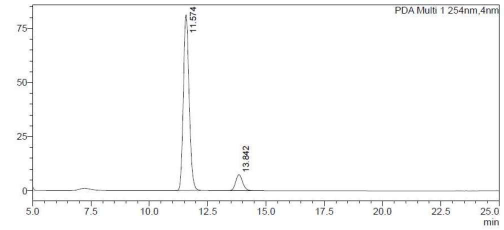

<Peak Table>

PDA Ch1 254nm

Peak#	Ret. Time	Area	Height	Area/Height	Height%	Area%
1	11.574	1400703	81203	17.249	91.652	90.206
2	13.842	152079	7396	20.563	8.348	9.794
Total		1552782	88599		100.000	100.000

isopropyl (*S*)-4-oxo-2-(6-oxopyridazin-1(*6H*)-yl)-4-phenylbutanoate

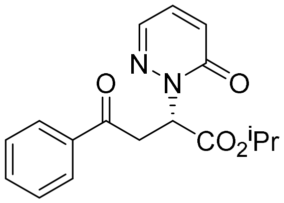

(+)-5da; isolated yield: 20.0 mg (64%); colorless sticky oil; $[\alpha]_D^{20}$ = +2.3 (*c* = 1.0, $CHCl_3$); ^{1}H NMR (500 MHz, $CDCl_3$) δ 8.00-7.98 (m, 2H), 7.74 (dd, *J* = 3.6, 1.6 Hz, 1H), 7.59 (dd, *J* = 10.6, 4.2 Hz, 1H), 7.48 (dd, *J* = 11.0, 4.4 Hz, 2H), 7.19 (dd, *J* = 9.5, 3.8 Hz, 1H), 6.97 (dd, *J* = 9.5, 1.7 Hz, 1H), 6.09 (dd, *J* = 7.7, 5.8 Hz, 1H), 5.11-5.06 (m, 1H), 3.92-3.82 (m, 2H), 1.24 (d, *J* = 6.3 Hz, 3H), 1.20 (d, *J* = 6.2 Hz, 3H); ^{13}C NMR (126 MHz, $CDCl_3$) δ 195.72, 168.71, 160.20, 136.26, 136.15, 133.50, 131.43, 130.01, 128.70, 128.20, 69.89, 58.83, 38.07, 21.69, 21.60; Enantiomeric excess: 83%, determined by HPLC (Chiralpak AD-H, hexane/*i*-PrOH = 70/30; flow rate 1.0 ml/min; 25 °C; 254 nm), first peak: t_R = 9.25 min, second peak: t_R = 10.60 min; HRMS (ESI) m/z calcd. for $C_{17}H_{18}N_2NaO_4$ [M+Na] $^+$ = 337.1159, found 337.1156.

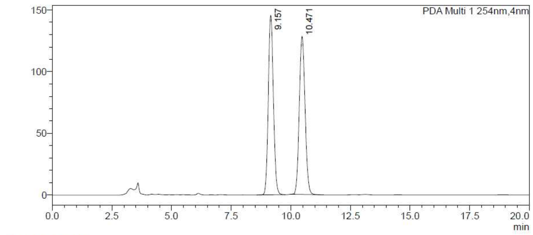

<Peak Table>
PDA Ch1 254nm

Peak#	Ret. Time	Area	Height	Area/Height	Height%	Area%
1	9.157	2167877	145440	14.906	53.129	50.026
2	10.471	2165655	128310	16.878	46.871	49.974
Total		4333531	273750		100.000	100.000

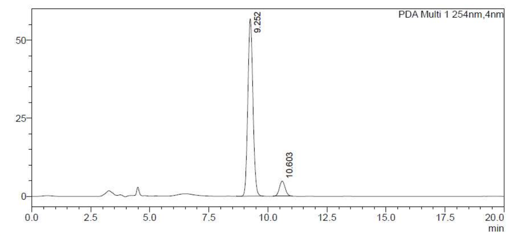

<Peak Table>
PDA Ch1 254nm

Peak#	Ret. Time	Area	Height	Area/Height	Height%	Area%
1	9.252	854654	56767	15.055	92.148	91.226
2	10.603	82195	4837	16.993	7.852	8.774
Total		936849	61604		100.000	100.000

tert-butyl (S)-4-oxo-2-(6-oxopyridazin-1(*6H*)-yl)-4-phenylbutanoate

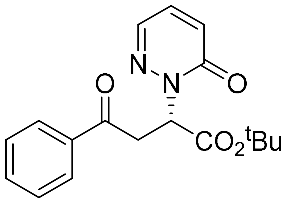

(+)-5ea; isolated yield: 17.1 mg (52%); colorless sticky oil; $[\alpha]_D^{20}$ = +2.5 (*c* = 1.0, $CHCl_3$); ^1H NMR (500 MHz, $CDCl_3$) δ 7.99 (d, *J* = 7.8 Hz, 2H), 7.73 (d, *J* = 3.6 Hz, 1H), 7.58 (t, *J* = 7.3 Hz, 1H), 7.47 (t, *J* = 7.6 Hz, 2H), 7.18 (dd, *J* = 9.5, 3.8 Hz, 1H), 6.96 (d, *J* = 9.4 Hz, 1H), 6.03 (t, *J* = 6.8 Hz, 1H), 3.85 (d, *J* = 6.8 Hz, 2H), 1.44 (s, 9H); ^{13}C NMR (126 MHz, $CDCl_3$) δ 195.81, 168.26, 160.23, 136.32, 135.99, 133.45, 131.30, 130.00, 128.68, 128.21, 82.80, 59.33, 38.06, 27.89; Enantiomeric excess: 88%, determined by HPLC (Chiralpak AD-H, hexane/*i*-PrOH = 70/30; flow rate 1.0 ml/min; 25 °C; 254 nm), first peak: t_R = 7.16 min, second peak: t_R = 9.37 min; HRMS (ESI) m/z calcd. for $C_{18}H_{20}N_2NaO_4$ $[M+Na]^+$ = 351.1315, found 351.1320.

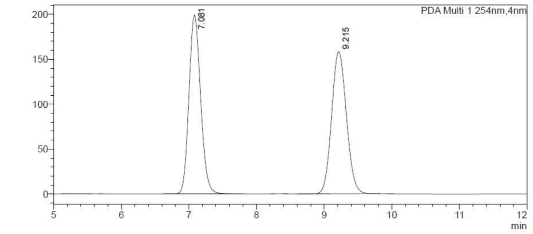

<Peak Table>
PDA Ch1 254nm

Peak#	Ret. Time	Area	Height	Area/Height	Height%	Area%
1	7.081	2391225	198890	12.023	55.695	49.873
2	9.215	2403425	158217	15.191	44.305	50.127
Total		4794650	357107		100.000	100.000

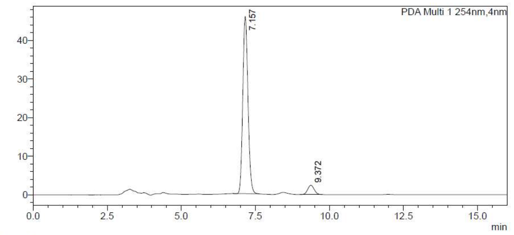

<Peak Table>
PDA Ch1 254nm

Peak#	Ret. Time	Area	Height	Area/Height	Height%	Area%
1	7.157	558142	45953	12.146	94.993	93.791
2	9.372	36948	2422	15.255	5.007	6.209
Total		595090	48375		100.000	100.000

Ethyl (*S*)-4-oxo-2-(6-oxopyridazin-1(*6H*)-yl)-4-(p-tolyl)butanoate

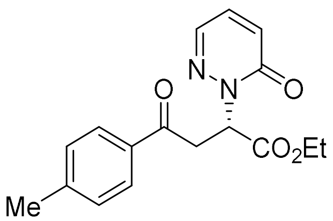

(+)-5fa; isolated yield: 15.4 mg (49%); colorless sticky oil; $[\alpha]_D^{20}$ = +12.5 (*c* = 1.0, $CHCl_3$); ^{1}H NMR (400 MHz, $CDCl_3$) δ 7.88 (d, *J* = 8.2 Hz, 2H), 7.72 (dd, *J* = 3.7, 1.5 Hz, 1H), 7.26 (d, *J* = 7.9 Hz, 2H), 7.18 (dd, *J* = 9.5, 3.8 Hz, 1H), 6.96 (dd, *J* = 9.5, 1.5 Hz, 1H), 6.11 (dd, *J* = 7.5, 6.0 Hz, 1H), 4.23 (qd, *J* = 7.1, 1.3 Hz, 2H), 3.86 (dd, *J* = 6.7, 3.7 Hz, 2H), 2.41 (s, 3H), 1.23 (t, *J* = 7.1 Hz, 3H); ^{13}C NMR (101 MHz, $CDCl_3$) δ 195.17, 169.24, 160.17, 144.31, 136.09, 133.85, 131.32, 130.02, 129.34, 128.30, 61.99, 58.74, 38.00, 21.67, 14.03; Enantiomeric excess: 82%, determined by HPLC (Chiralpak AD-H, hexane/*i*-PrOH = 70/30; flow rate 1.0 ml/min; 25 °C; 254 nm), first peak: t_R = 12.81 min, second peak: t_R = 16.17 min; HRMS (ESI) m/z calcd. for $C_{17}H_{18}N_2NaO_4$ $[M+Na]^+$ = 337.1159, found 337.1158.

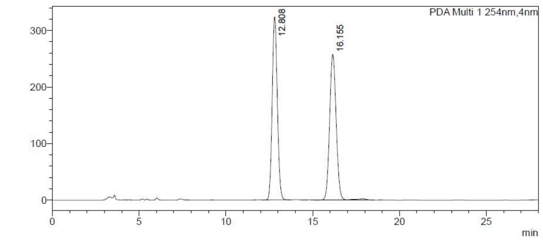

<Peak Table>
PDA Ch1 254nm

Peak#	Ret. Time	Area	Height	Area/Height	Height%	Area%
1	12.808	6652490	324038	20.530	55.695	49.388
2	16.155	6817283	257767	26.447	44.305	50.612
Total		13469774	581805		100.000	100.000

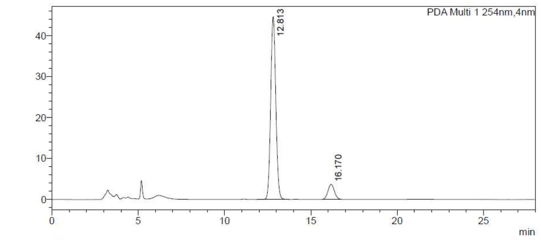

<Peak Table>
PDA Ch1 254nm

Peak#	Ret. Time	Area	Height	Area/Height	Height%	Area%
1	12.813	915999	44626	20.526	92.431	90.763
2	16.170	93226	3654	25.510	7.569	9.237
Total		1009225	48280		100.000	100.000

Ethyl (*S*)-4-(4-methoxyphenyl)-4-oxo-2-(6-oxopyridazin-1(6H)-yl)butanoate

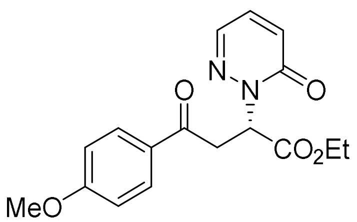

(+)-5ga; isolated yield: 10 mg (30%); white solid; $[\alpha]_D^{20}$ = +20.2 (*c* = 0.33, $CHCl_3$); ^{1}H NMR (500 MHz, $CDCl_3$) δ 7.97 (d, *J* = 8.6 Hz, 2H), 7.74 (d, *J* = 3.6 Hz, 1H), 7.18 (dd, *J* = 9.4, 3.7 Hz, 1H), 6.95 (dd, *J* = 12.2, 9.2 Hz, 3H), 6.11 (t, *J* = 6.7 Hz, 1H), 4.25-4.21 (m, 2H), 3.87 (s, 3H), 3.85-3.83 (m, 2H), 1.24 (t, *J* = 7.1 Hz, 3H); ^{13}C NMR

(126 MHz, $CDCl_3$) δ 194.07, 169.36, 163.76, 160.21, 136.16, 131.38, 130.51, 130.04, 129.33, 113.81, 62.03, 58.80, 55.54, 37.74, 14.07; Enantiomeric excess: 77%, determined by HPLC (Chiralpak AD-H, hexane/*i*-PrOH = 70/30; flow rate 1.0 ml/min; 25 °C; 254 nm), first peak: t_R = 17.79 min, second peak: t_R = 24.43 min; HRMS (ESI) m/z calcd. for $C_{17}H_{18}N_2NaO_5$ $[M+Na]^+$ = 353.1108, found 353.1113.

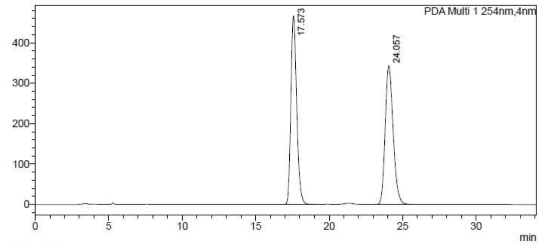

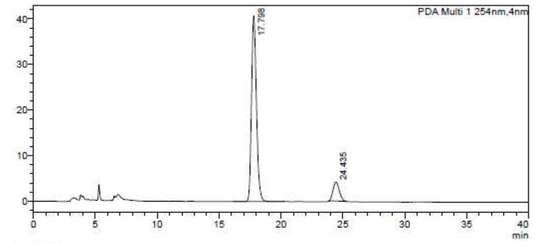

<Peak Table>

PDA Ch1 254nm

Peak#	Ret. Time	Area	Height	Area/Height	Height%	Area%
1	17.573	12868033	466398	27.590	57.618	49.889
2	24.057	12925246	343063	37.676	42.382	50.111
Total		25793279	809461		100.000	100.000

<Peak Table>

PDA Ch1 254nm

Peak#	Ret. Time	Area	Height	Area/Height	Height%	Area%
1	17.798	1123499	40773	27.555	90.744	88.402
2	24.435	147397	4159	35.440	9.256	11.598
Total		1270896	44932		100.000	100.000

Ethyl (*S*)-4-([1,1'-biphenyl]-4-yl)-4-oxo-2-(6-oxopyridazin-1(*6H*)-yl)-Butanoate

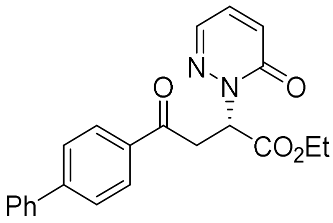

(+)-5ha; isolated yield: 24.8 mg (66%); colorless sticky oil; $[\alpha]_D^{20}$ = +44.2 (*c* = 1.0, $CHCl_3$); ^1H NMR (400 MHz, $CDCl_3$) δ 8.06 (d, *J* = 8.4 Hz, 2H), 7.74 (dd, *J* = 3.7, 1.5 Hz, 1H), 7.69 (d, *J* = 8.4 Hz, 2H), 7.62 (d, *J* = 7.3 Hz, 2H), 7.47 (t, *J* = 7.4 Hz, 2H), 7.40 (t, *J* = 7.3 Hz, 1H), 7.18 (dd, *J* = 9.5, 3.8 Hz, 1H), 6.97 (dd, *J* = 9.5, 1.5 Hz, 1H), 6.14 (dd, *J* = 7.7, 5.8 Hz, 1H), 4.27-4.21 (m, 2H), 3.98-3.86 (m, 2H), 1.25 (t, *J* = 7.1 Hz, 3H); ^{13}C NMR (101 MHz, $CDCl_3$) δ 195.18, 169.21, 160.19, 146.14, 139.77, 136.16, 135.01, 131.37, 130.06, 128.99, 128.80, 128.33, 127.30, 127.29, 62.05, 58.78, 38.15, 14.06; Enantiomeric excess: 81%, determined by HPLC (Chiralpak AD-H, hexane/*i*-PrOH = 70/30; flow rate 1.0 ml/min; 25 °C; 254 nm), first peak: t_R = 18.93 min, second peak: t_R = 34.60 min; HRMS (ESI) m/z calcd. for $C_{22}H_{20}N_2NaO_4$ $[M+Na]^+$ = 399.1315, found 399.1314.

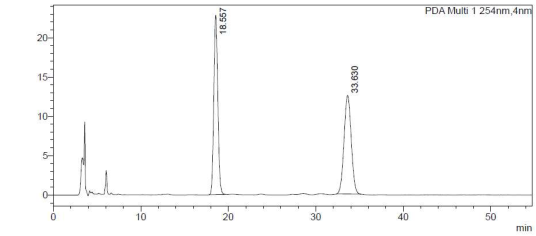

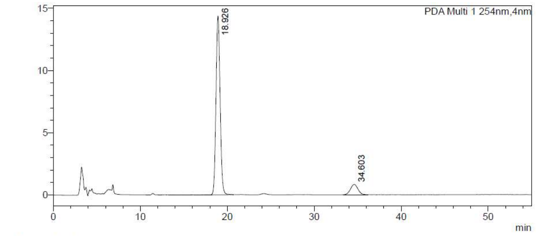

<Peak Table>

PDA Ch1 254nm

Peak#	Ret. Time	Area	Height	Area/Height	Height%	Area%
1	18.557	721314	22798	31.639	64.559	50.233
2	33.630	714612	12515	57.099	35.441	49.767
Total		1435925	35313		100.000	100.000

<Peak Table>

PDA Ch1 254nm

Peak#	Ret. Time	Area	Height	Area/Height	Height%	Area%
1	18.926	472663	14372	32.889	94.564	90.584
2	34.603	49131	826	59.465	5.436	9.416
Total		521794	15198		100.000	100.000

Ethyl (*S*)-4-(4-fluorophenyl)-4-oxo-2-(6-oxopyridazin-1(*6H*)-yl)butanoate

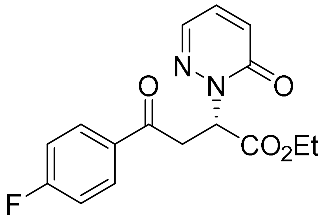

(+)-5ia; isolated yield: 22.3 mg (70%); colorless sticky oil; $[\alpha]_D^{20}$ = +1.7 (c = 1.0, $CHCl_3$); ^{1}H NMR (500 MHz, $CDCl_3$) δ 8.03-8.00 (m, 2H), 7.74 (dd, J = 3.7, 1.6 Hz, 1H), 7.20 (dd, J = 9.5, 3.8 Hz, 1H), 7.14 (t, J = 8.6 Hz, 2H), 6.97 (dd, J = 9.5, 1.6 Hz, 1H), 6.10 (dd, J = 7.9, 5.6 Hz, 1H), 4.26-4.20 (m, 2H), 3.90 (dd, J = 17.6, 5.6 Hz, 1H), 3.80 (dd, J = 17.6, 7.9 Hz, 1H), 1.23 (t, J = 7.1 Hz, 3H); ^{19}F NMR (376 MHz, $CDCl_3$) δ -104.43; ^{13}C NMR (126 MHz, $CDCl_3$) δ 194.09, 169.06, 165.95 (d, J = 255.3 Hz), 160.14, 136.22, 132.75 (d, J = 2.9 Hz), 131.43, 130.86 (d, J = 9.4 Hz), 130.06, 62.08, 115.81 (d, J = 21.9 Hz), 58.75, 38.03, 14.02; Enantiomeric excess: 82%, determined by HPLC (Chiralpak AD-H, hexane/*i*-PrOH = 70/30; flow rate 1.0 ml/min; 25 °C; 254 nm), first peak: t_R = 12.11 min, second peak: t_R = 14.38 min; HRMS (ESI) m/z calcd. for $C_{16}H_{15}FN_2NaO_4$ $[M+Na]^+$ = 341.0908, found 341.0905.

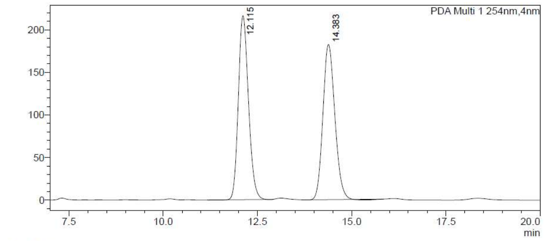

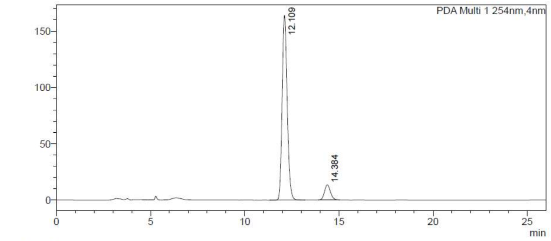

<Peak Table>

PDA Ch1 254nm

Peak#	Ret. Time	Area	Height	Area/Height	Height%	Area%
1	12.115	4033504	216040	18.670	54.237	50.185
2	14.383	4003817	182283	21.965	45.763	49.815
Total		8037321	398323		100.000	100.000

<Peak Table>

PDA Ch1 254nm

Peak#	Ret. Time	Area	Height	Area/Height	Height%	Area%
1	12.109	3064112	164095	18.673	92.388	91.169
2	14.384	296815	13519	21.955	7.612	8.831
Total		3360927	177614		100.000	100.000

Ethyl (*S*)-4-(4-chlorophenyl)-4-oxo-2-(6-oxopyridazin-1(*6H*)-yl)butanoate

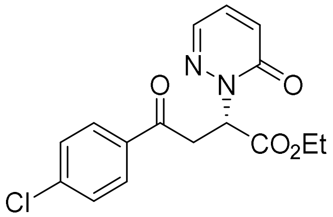

(+)-5ja; isolated yield: 27.1 mg (81%); colorless sticky oil; $[\alpha]_D^{20}$ = +19.6 (*c* = 1.0, $CHCl_3$); ^{1}H NMR (500 MHz, $CDCl_3$) δ 7.93 (d, *J* = 8.6 Hz, 2H), 7.75 (dd, *J* = 3.7, 1.5 Hz, 1H), 7.45 (d, *J* = 8.6 Hz, 2H), 7.21 (dd, *J* = 9.5, 3.8 Hz, 1H), 6.98 (dd, *J* = 9.5, 1.5 Hz, 1H), 6.10 (dd, *J* = 7.9, 5.6 Hz, 1H), 4.26-4.20 (m, 2H), 3.90 (dd, *J* = 17.7, 5.5 Hz, 1H), 3.81 (dd, *J* = 17.7, 8.0 Hz, 1H), 1.24 (t, *J* = 7.1 Hz, 3H); ^{13}C NMR (126 MHz, $CDCl_3$) δ 194.53, 169.07, 160.17, 139.98, 136.32, 134.53, 131.52, 130.10, 129.64, 129.03, 62.17, 58.67, 38.07, 14.07; Enantiomeric excess: 83%, determined by HPLC (Chiralpak AD-H, hexane/*i*-PrOH = 70/30; flow rate 1.0 ml/min; 25 °C; 254 nm), first peak: t_R = 14.48 min, second peak: t_R = 17.87 min; HRMS (ESI) m/z calcd. for $C_{16}H_{15}ClN_2NaO_4$ $[M+Na]^+$ = 357.0613, found 357.0608.

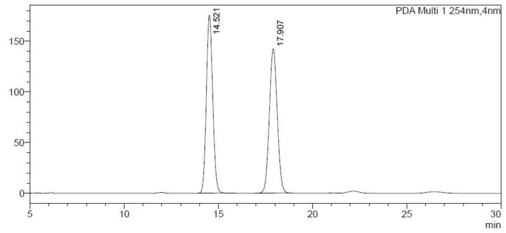

<Peak Table>

PDA Ch1 254nm

Peak#	Ret. Time	Area	Height	Area/Height	Height%	Area%
1	14.521	4126845	175637	23.496	55.233	49.879
2	17.907	4146926	142354	29.131	44.767	50.121
Total		8273770	317991		100.000	100.000

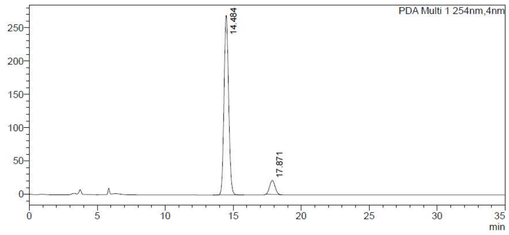

<Peak Table>

PDA Ch1 254nm

Peak#	Ret. Time	Area	Height	Area/Height	Height%	Area%
1	14.484	6327574	269665	23.465	92.790	91.598
2	17.871	580427	20955	27.699	7.210	8.402
Total		6908001	290620		100.000	100.000

Ethyl (*S*)-4-(4-bromophenyl)-4-oxo-2-(6-oxopyridazin-1(*6H*)-yl)butanoate

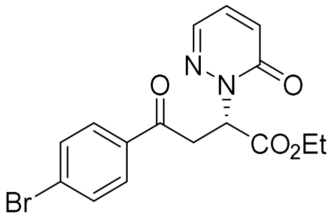

(+)-5ka; isolated yield: 33.0 mg (87%); colorless sticky oil; $[\alpha]_D^{20}$ = +20.3 (*c* = 1.0, $CHCl_3$); ^{1}H NMR (500 MHz, $CDCl_3$) δ 7.85 (d, *J* = 8.5 Hz, 2H), 7.73 (dd, *J* = 3.7, 1.6 Hz, 1H), 7.61 (d, *J* = 8.5 Hz, 2H), 7.19 (dd, *J* = 9.5, 3.8 Hz, 1H), 6.96 (dd, *J* = 9.5, 1.6 Hz, 1H), 6.09 (dd, *J* = 7.9, 5.6 Hz, 1H), 4.26-4.19 (m, 2H), 3.89 (dd, *J* = 17.7, 5.6 Hz,

1H), 3.78 (dd, J = 17.7, 7.9 Hz, 1H), 1.23 (t, J = 7.1 Hz, 3H); ^{13}C NMR (126 MHz, $CDCl_3$) δ 194.71, 168.99, 160.12, 136.25, 135.02, 132.00, 131.46, 130.06, 129.70, 128.69, 62.10, 58.70, 38.05, 14.04; Enantiomeric excess: 79%, determined by HPLC (Chiralpak AD-H, hexane/*i*-PrOH = 70/30; flow rate 1.0 ml/min; 25 °C; 254 nm), first peak: t_R = 14.46 min, second peak: t_R = 17.99 min; HRMS (ESI) m/z calcd. for $C_{16}H_{15}BrN_2NaO_4$ $[M+Na]^+$ = 401.0107, found 401.0101.

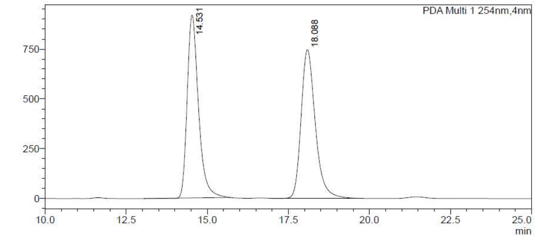

<Peak Table>

PDA Ch1 254nm

Peak#	Ret. Time	Area	Height	Area/Height	Height%	Area%
1	14.531	22196624	917336	24.197	55.141	49.511
2	18.088	22634920	746288	30.330	44.859	50.489
Total		44831544	1663624		100.000	100.000

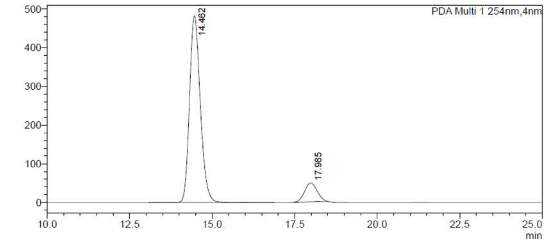

<Peak Table>

PDA Ch1 254nm

Peak#	Ret. Time	Area	Height	Area/Height	Height%	Area%
1	14.462	10954210	482325	22.711	90.794	89.452
2	17.985	1291722	48903	26.414	9.206	10.548
Total		12245932	531228		100.000	100.000

Ethyl (*S*)-4-oxo-2-(6-oxopyridazin-1(*6H*)-yl)-4-(4-(trifluoromethyl)phenyl)-Butanoate

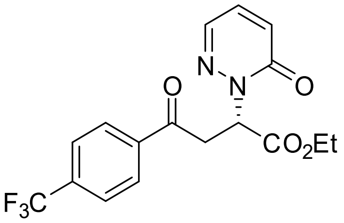

(+)-5la; isolated yield: 25.0 mg (68%); colorless sticky oil; $[\alpha]_D^{20}$ = +1.8 (c = 1.0, $CHCl_3$); ^{1}H NMR (500 MHz, $CDCl_3$) δ 8.10 (d, J = 8.2 Hz, 2H), 7.75 (d, J = 7.3 Hz, 3H), 7.21 (dd, J = 9.5, 3.8 Hz, 1H), 6.99 (dd, J = 9.5, 1.4 Hz, 1H), 6.11 (dd, J = 7.8, 5.7 Hz, 1H), 4.27-4.21 (m, 2H), 3.96 (dd, J = 17.7, 5.6 Hz, 1H), 3.83 (dd, J = 17.7, 7.9 Hz, 1H), 1.24 (t, J = 7.1 Hz, 3H); ^{19}F NMR (282 MHz, $CDCl_3$) δ -63.14; ^{13}C NMR (126 MHz, $CDCl_3$) δ 194.88, 168.94, 160.16, 138.88, 136.36, 134.59 (t, J = 32.7 Hz), 131.54, 130.14, 128.57, 125.80 (q, J = 3.7 Hz), 123.53 (q, J = 272.7 Hz), 62.23, 58.67, 38.38, 14.05; Enantiomeric excess: 82%, determined by HPLC (Chiralpak AD-H, hexane/*i*-PrOH = 70/30; flow rate 1.0 ml/min; 25 °C; 254 nm), first peak: t_R = 11.00 min, second peak: t_R = 14.45 min; HRMS (ESI) m/z calcd. for $C_{17}H_{15}F_3N_2NaO_4$ $[M+Na]^+$ = 391.0876, found 391.0875.

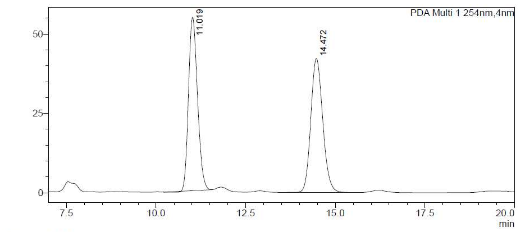

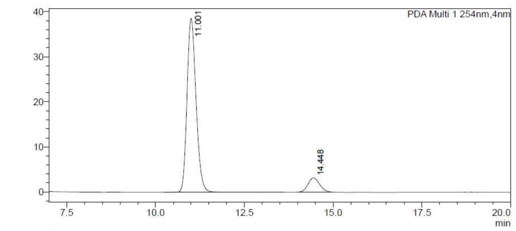

<Peak Table>
PDA Ch1 254nm

Peak#	Ret. Time	Area	Height	Area/Height	Height%	Area%
1	11.019	948396	54657	17.352	56.415	49.215
2	14.472	978661	42226	23.177	43.585	50.785
Total		1927057	96883		100.000	100.000

<Peak Table>
PDA Ch1 254nm

Peak#	Ret. Time	Area	Height	Area/Height	Height%	Area%
1	11.001	677286	38527	17.579	92.499	90.737
2	14.448	69138	3124	22.128	7.501	9.263
Total		746424	41652		100.000	100.000

Ethyl (*S*)-4-(4-cyanophenyl)-4-oxo-2-(6-oxopyridazin-1(*6H*)-yl)butanoate

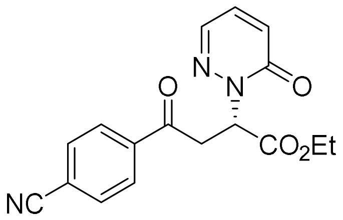

(+)-5ma; isolated yield: 28.6 mg (88%); colorless sticky oil; $[\alpha]_D^{20}$ = +15.1 (*c* = 1.0, $CHCl_3$); ^{1}H NMR (500 MHz, $CDCl_3$) δ 8.09 (d, *J* = 8.4 Hz, 2H), 7.80 (d, *J* = 8.4 Hz, 2H), 7.75 (dd, *J* = 3.8, 1.6 Hz, 1H), 7.23 (dd, *J* = 9.5, 3.8 Hz, 1H), 6.99 (dd, *J* = 9.5, 1.6 Hz, 1H), 6.09 (dd, *J* = 7.6, 5.8 Hz, 1H), 4.27-4.20 (m, 2H), 3.97 (dd, *J* = 17.7, 5.8 Hz, 1H), 3.79 (dd, *J* = 17.7, 7.7 Hz, 1H), 1.24 (t, *J* = 7.1 Hz, 3H); ^{13}C NMR (126 MHz, $CDCl_3$) δ 194.62, 168.81, 160.11, 139.18, 136.43, 132.62, 131.61, 130.15, 128.65, 117.89, 116.69, 62.27, 58.67, 38.39, 14.04; Enantiomeric excess: 83%, determined by HPLC (Chiralpak AD-H, hexane/*i*-PrOH = 70/30; flow rate 1.0 ml/min; 25 °C; 254 nm), first peak: t_R = 16.64 min, second peak: t_R = 19.78 min; HRMS (ESI) m/z calcd. for $C_{17}H_{15}N_3NaO_4$ $[M+Na]^+$ = 348.0955, found 348.0960.

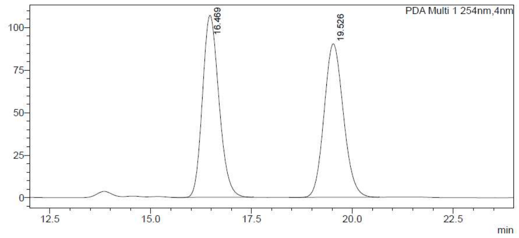

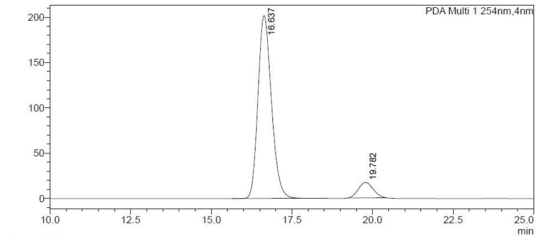

<Peak Table>
PDA Ch1 254nm

Peak#	Ret. Time	Area	Height	Area/Height	Height%	Area%
1	16.469	3046865	106996	28.476	54.268	50.295
2	19.526	3011165	90168	33.395	45.732	49.705
Total		6058030	197164		100.000	100.000

<Peak Table>
PDA Ch1 254nm

Peak#	Ret. Time	Area	Height	Area/Height	Height%	Area%
1	16.637	5848919	201714	28.996	92.211	91.452
2	19.782	546718	17039	32.087	7.789	8.548
Total		6395638	218752		100.000	100.000

Ethyl (*S*)-4-(3,4-dichlorophenyl)-4-oxo-2-(6-oxopyridazin-1(*6H*)-yl)-

Butanoate

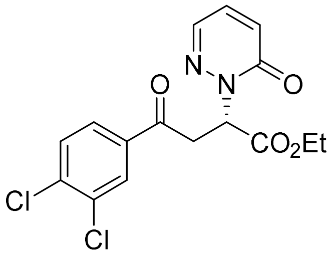

(+)-5na; isolated yield: 35.0 mg (95%); colorless sticky oil; $[\alpha]_D^{20}$ = +13.4 (*c* = 1.0, $CHCl_3$); ^{1}H NMR (500 MHz, $CDCl_3$) δ 8.05 (s, 1H), 7.81 (dd, *J* = 8.4, 1.0 Hz, 1H), 7.75 (dd, *J* = 2.2, 1.5 Hz, 1H), 7.57-7.55 (m, 1H), 7.21 (dd, *J* = 9.5, 3.8 Hz, 1H), 6.97 (d, *J* = 9.5 Hz, 1H), 6.07 (t, *J* = 6.7 Hz, 1H), 4.26-4.20 (m, 2H), 3.89 (dd, *J* = 17.7, 5.6 Hz, 1H), 3.75 (dd, *J* = 17.7, 7.8 Hz, 1H), 1.25-1.22 (m, 3H); ^{13}C NMR (126 MHz, $CDCl_3$) δ 193.63, 168.88, 160.13, 138.09, 136.38, 135.74, 133.39, 131.57, 130.86, 130.20, 130.11, 127.24, 62.22, 58.65, 38.11, 14.05; Enantiomeric excess: 80%, determined by HPLC (Chiralpak OD-H to OD-H, hexane/*i*-PrOH = 60/40; flow rate 0.5 ml/min; 25 °C; 254 nm), first peak: t_R = 69.93 min, second peak: t_R = 74.21 min; HRMS (ESI) m/z calcd. for $C_{16}H_{14}Cl_2N_2NaO_4$ $[M+Na]^+$ = 391.0223, found 391.0220.

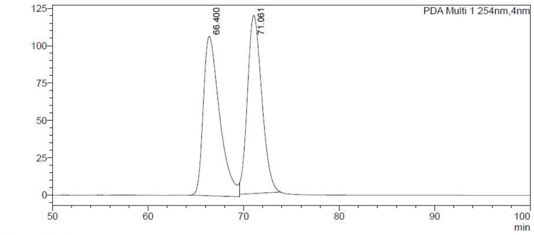

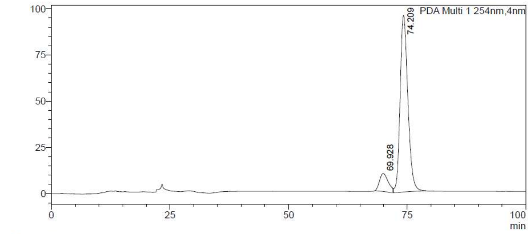

<Peak Table>

PDA Ch1 254nm

Peak#	Ret. Time	Area	Height	Area/Height	Height%	Area%
1	66.400	12675493	106808	118.676	47.245	50.394
2	71.061	12477177	119267	104.616	52.755	49.606
Total		25152671	226074		100.000	100.000

<Peak Table>

PDA Ch1 254nm

Peak#	Ret. Time	Area	Height	Area/Height	Height%	Area%
1	69.928	1223945	9833	124.477	9.323	10.124
2	74.209	10865410	95638	113.610	90.677	89.876
Total		12089355	105470		100.000	100.000

Ethyl (*S*)-4-(benzo[b]thiophen-2-yl)-4-oxo-2-(6-oxopyridazin-1(*6H*)-yl)but-anoate

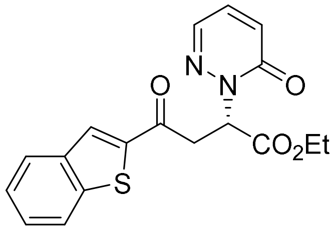

(+)-5oa; isolated yield: 29.9 mg (84%); colorless sticky oil; $[\alpha]_D^{20}$ = +33.1 (*c* = 1.0, $CHCl_3$); ^{1}H NMR (400 MHz, $CDCl_3$) δ 8.05 (s, 1H), 7.88 (dd, *J* = 13.8, 7.9 Hz, 2H), 7.74 (dd, *J* = 3.7, 1.6 Hz, 1H), 7.49-7.39 (m, 2H), 7.19 (dd, *J* = 9.5, 3.8 Hz, 1H), 6.96

(dd, J = 9.5, 1.5 Hz, 1H), 6.10 (dd, J = 8.0, 5.7 Hz, 1H), 4.28-4.20 (m, 2H), 4.00-3.86 (m, 2H), 1.24 (t, J = 7.1 Hz, 3H); ^{13}C NMR (101 MHz, $CDCl_3$) δ 190.01, 168.89, 160.16, 142.74, 142.60, 139.01, 136.33, 131.53, 130.07, 129.67, 127.66, 126.08, 125.12, 123.00, 62.17, 58.84, 38.54, 14.05; Enantiomeric excess: 70%, determined by HPLC (Chiralpak AD-H, hexane/*i*-PrOH = 70/30; flow rate 1.0 ml/min; 25 °C; 254 nm), first peak: t_R = 15.02 min, second peak: t_R = 17.21 min; HRMS (ESI) m/z calcd. for $C_{18}H_{16}N_2NaO_4S$ $[M+Na]^+$ = 379.0723, found 379.0723.

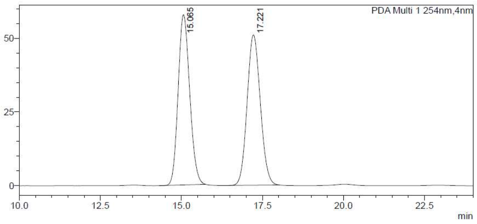

<Peak Table>
PDA Ch1 254nm

Peak#	Ret. Time	Area	Height	Area/Height	Height%	Area%
1	15.065	1435781	57772	24.853	53.087	49.780
2	17.221	1448465	51052	28.372	46.913	50.220
Total		2884246	108824		100.000	100.000

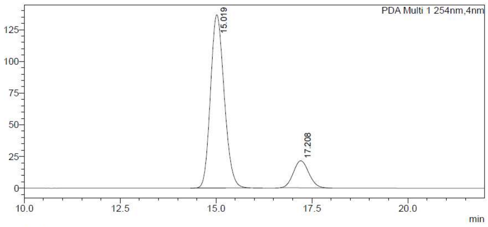

<Peak Table>
PDA Ch1 254nm

Peak#	Ret. Time	Area	Height	Area/Height	Height%	Area%
1	15.019	3404298	136628	24.917	86.472	85.014
2	17.208	600095	21374	28.075	13.528	14.986
Total		4004393	158003		100.000	100.000

Ethyl (*S*)-4-(naphthalen-2-yl)-4-oxo-2-(6-oxopyridazin-1(*6H*)-yl)butanoate

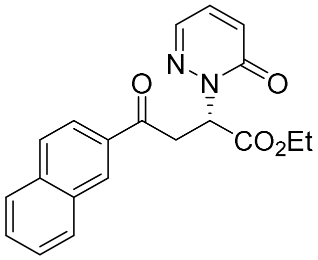

(+)-5pa; isolated yield: 20.0 mg (57%); colorless sticky oil; $[\alpha]_D^{20}$ = +47.1 (c = 1.0, $CHCl_3$); ^{1}H NMR (400 MHz, $CDCl_3$) δ 8.52 (s, 1H), 8.03 (dd, J = 8.6, 1.6 Hz, 1H), 7.96 (d, J = 8.0 Hz, 1H), 7.90-7.86 (m, 2H), 7.74 (dd, J = 3.7, 1.6 Hz, 1H), 7.63-7.54 (m, 2H), 7.18 (dd, J = 9.5, 3.8 Hz, 1H), 6.97 (dd, J = 9.5, 1.6 Hz, 1H), 6.17 (dd, J = 7.7, 5.8 Hz, 1H), 4.29-4.21 (m, 2H), 4.09-3.97 (m, 2H), 1.25 (t, J = 7.1 Hz, 3H); ^{13}C NMR (101 MHz, $CDCl_3$) δ 195.53, 169.26, 160.23, 136.23, 135.74, 133.60, 132.45, 131.45, 130.07, 129.63, 128.70, 128.56, 127.81, 126.91, 123.75, 62.09, 58.91, 38.16, 14.08; Enantiomeric excess: 79%, determined by HPLC (Chiralpak AD-H to AD-H, hexane/*i*-PrOH = 60/40; flow rate 0.5 ml/min; 25 °C; 254 nm), first peak: t_R = 39.78 min, second peak: t_R = 41.53 min; HRMS (ESI) m/z calcd. for $C_{20}H_{18}N_2NaO_4$ $[M+Na]^+$ = 373.1159, found 373.1151.

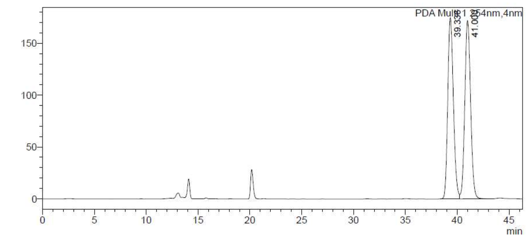

<Peak Table>
PDA Ch1 254nm

Peak#	Ret. Time	Area	Height	Area/Height	Height%	Area%
1	39.336	6587284	174643	37.719	50.417	49.607
2	41.000	6691619	171755	38.960	49.583	50.393
Total		13278903	346398		100.000	100.000

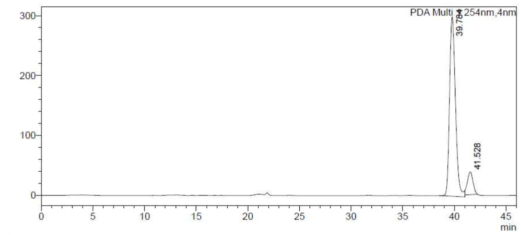

<Peak Table>
PDA Ch1 254nm

Peak#	Ret. Time	Area	Height	Area/Height	Height%	Area%
1	39.784	11896876	299441	39.730	88.719	89.228
2	41.528	1436254	38074	37.722	11.281	10.772
Total		13333129	337515		100.000	100.000

(*R*)-2-(1,1,1-trifluoro-4-oxo-4-phenylbutan-2-yl)pyridazin-3(*2H*)-one

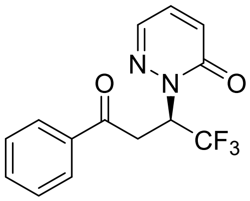

(-)-3aa; isolated yield: 26.6 mg (90%); colorless sticky oil; $[\alpha]_D^{20}$ = -286.7 (*c* = 1.0, $CHCl_3$); Enantiomeric excess: 94%, determined by HPLC (Chiralpak AD-H, hexane/*i*-PrOH = 80/20; flow rate 1.0 ml/min; 25 °C; 254 nm), first peak: t_R = 8.388 min, second peak: t_R = 12.096 min.

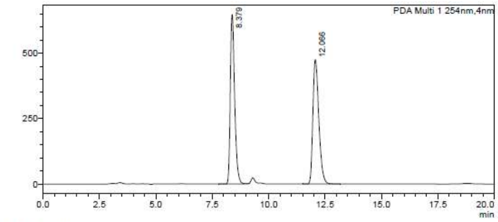

<Peak Table>
PDA Ch1 254nm

Peak#	Ret. Time	Area	Height	Height%	Area%
1	8.379	8674606	646927	57.665	49.950
2	12.066	8691860	474943	42.335	50.050
Total		17366466	1121870	100.000	100.000

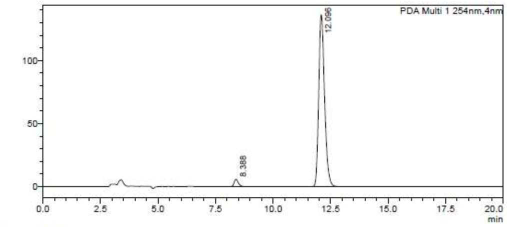

<Peak Table>
PDA Ch1 254nm

Peak#	Ret. Time	Area	Height	Height%	Area%
1	8.388	72287	5786	4.071	2.906
2	12.096	2415473	136352	95.929	97.094
Total		2487760	142138	100.000	100.000

(*R*)-2-(1,1,1-trifluoro-4-oxo-4-(p-tolyl)butan-2-yl)pyridazin-3(*2H*)-one

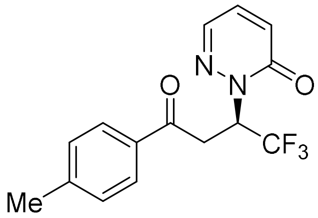

(-)-3ba; isolated yield: 30.7 mg (99%); colorless sticky oil; $[\alpha]_D^{20}$ = -298.4 (*c* = 1.0, $CHCl_3$); Enantiomeric excess: 96%, determined by HPLC (Chiralpak AD-H, hexane/*i*-PrOH = 80/20; flow rate 1.0 ml/min; 25 °C; 254 nm), first peak: t_R = 9.286 min, second peak: t_R = 14.768 min.

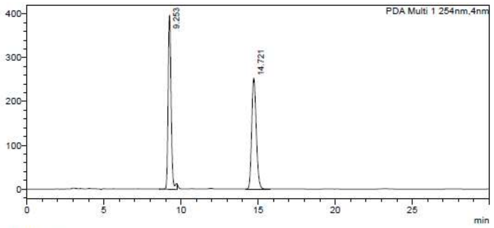

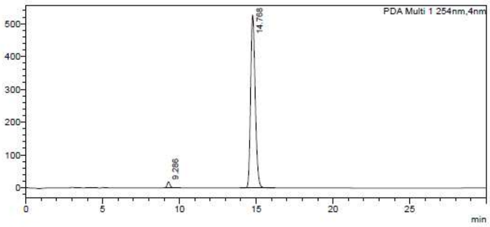

<Peak Table>
PDA Ch1 254nm

Peak#	Ret. Time	Area	Height	Height%	Area%
1	9.253	5343459	398081	61.118	50.668
2	14.721	5202519	253254	38.882	49.332
Total		10545978	651336	100.000	100.000

<Peak Table>
PDA Ch1 254nm

Peak#	Ret. Time	Area	Height	Height%	Area%
1	9.286	222868	17505	3.218	1.984
2	14.768	11009439	526486	96.782	98.016
Total		11232307	543991	100.000	100.000

(*R*)-2-(1,1,1-trifluoro-4-(4-methoxyphenyl)-4-oxobutan-2-yl)pyridazin-3(*2H*)-one

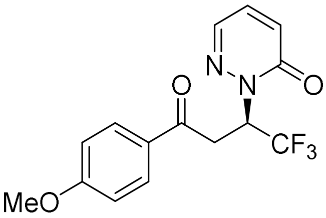

(-)-3ca; isolated yield: 29.3 mg (90%); colorless sticky oil; $[\alpha]_D^{20}$ = -320.6 (*c* = 1.0, $CHCl_3$); Enantiomeric excess: 95%, determined by HPLC (Chiralpak AD-H, hexane/*i*-PrOH = 80/20; flow rate 1.0 ml/min; 25 °C; 254 nm), first peak: t_R = 12.734 min, second peak: t_R = 21.611 min.

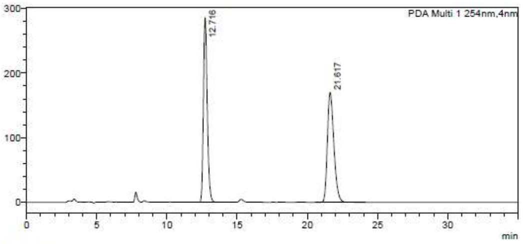

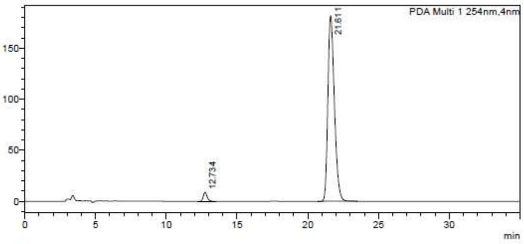

<Peak Table>
PDA Ch1 254nm

Peak#	Ret. Time	Area	Height	Height%	Area%
1	12.716	5421723	285553	62.697	49.806
2	21.617	5463996	169899	37.303	50.194
Total		10885719	455452	100.000	100.000

<Peak Table>
PDA Ch1 254nm

Peak#	Ret. Time	Area	Height	Height%	Area%
1	12.734	164347	8764	4.599	2.740
2	21.611	5833443	181812	95.401	97.260
Total		5997790	190576	100.000	100.000

(*R*)-2-(4-([1,1'-biphenyl]-4-yl)-1,1,1-trifluoro-4-oxobutan-2-yl)pyridazin-3(*2H*)-one

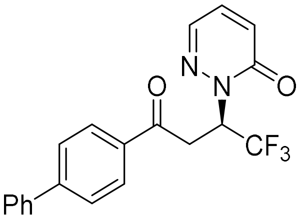

(-)-3da; isolated yield: 36.8 mg (99%); white solid; $[\alpha]_D^{20}$ = -256.1 (*c* = 1.0, $CHCl_3$);

Enantiomeric excess: 95%, determined by HPLC (Chiralpak AD-H, hexane/*i*-PrOH = 80/20; flow rate 1.0 ml/min; 25 °C; 254 nm), first peak: t_R = 13.774 min, second peak: t_R = 24.216 min.

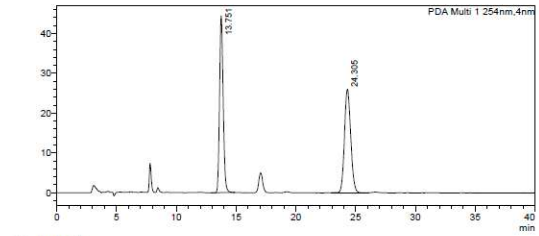

<Peak Table>
PDA Ch1 254nm

Peak#	Ret. Time	Area	Height	Height%	Area%
1	13.751	941345	44466	63.110	49.852
2	24.305	946942	25992	36.890	50.148
Total		1888287	70458	100.000	100.000

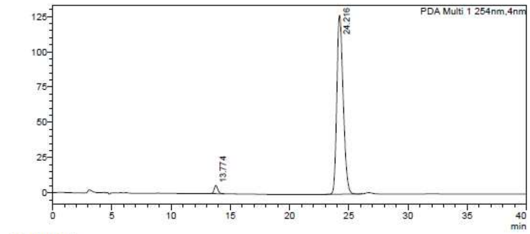

<Peak Table>
PDA Ch1 254nm

Peak#	Ret. Time	Area	Height	Height%	Area%
1	13.774	125345	5855	4.410	2.569
2	24.216	4754448	126891	95.590	97.431
Total		4879793	132746	100.000	100.000

(*R*)-2-(1,1,1-trifluoro-4-(4-fluorophenyl)-4-oxobutan-2-yl)pyridazin-3(*2H*) -one

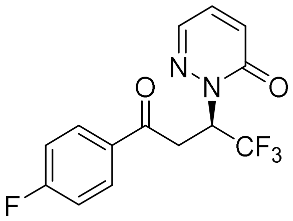

(-)-3ea; isolated yield: 29.8 mg (95%); colorless sticky oil; $[\alpha]_D^{20}$ = -243.1 (*c* = 1.0, $CHCl_3$); Enantiomeric excess: 94%, determined by HPLC (Chiralpak AD-H, hexane/*i*-PrOH = 90/10; flow rate 1.0 ml/min; 25 °C; 254 nm), first peak: t_R = 8.954 min, second peak: t_R = 14.645 min.

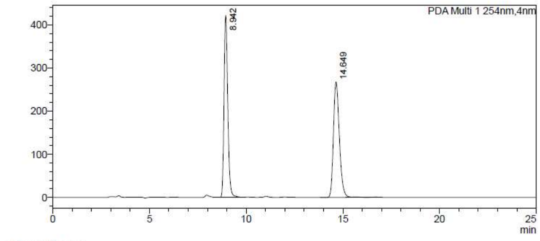

<Peak Table>
PDA Ch1 254nm

Peak#	Ret. Time	Area	Height	Height%	Area%
1	8.942	5637774	421701	61.184	49.782
2	14.649	5687222	267537	38.816	50.218
Total		11324996	689238	100.000	100.000

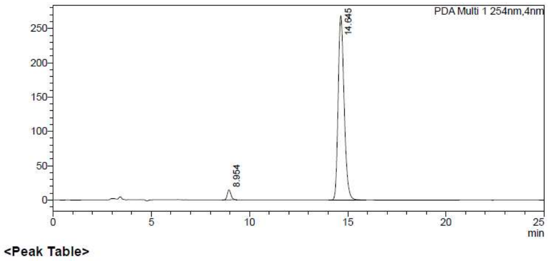

<Peak Table>
PDA Ch1 254nm

Peak#	Ret. Time	Area	Height	Height%	Area%
1	8.954	189568	14624	5.164	3.221
2	14.645	5695967	268596	94.836	96.779
Total		5885535	283220	100.000	100.000

(*R*)-2-(4-(4-chlorophenyl)-1,1,1-trifluoro-4-oxobutan-2-yl)pyridazin-3(*2H*) -one

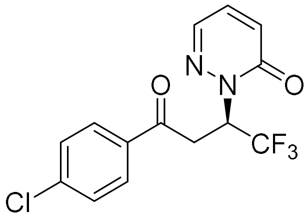

(-)-3fa; isolated yield: 31.2 mg (95%); colorless sticky oil; $[\alpha]_D^{20}$ = -284.5 (*c* = 1.0, $CHCl_3$); Enantiomeric excess: 95%, determined by HPLC (Chiralpak AD-H, hexane/*i*-PrOH = 80/20; flow rate 1.0 ml/min; 25 °C; 254 nm), first peak: t_R = 9.908 min, second peak: t_R = 16.340 min.

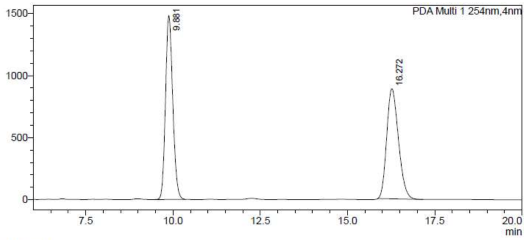

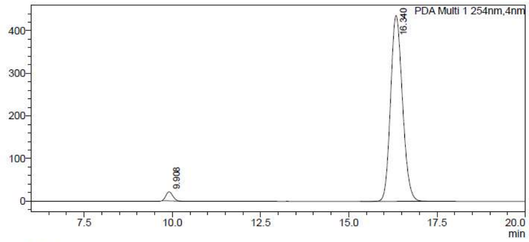

<Peak Table>

PDA Ch1 254nm

Peak#	Ret. Time	Area	Height	Height%	Area%
1	9.881	21404177	1480557	62.563	50.818
2	16.272	20715070	885967	37.437	49.182
Total		42119247	2366524	100.000	100.000

<Peak Table>

PDA Ch1 254nm

Ret. Time	Area	Height	Unit	Peak End	Mark	Height%	Area%
9.908	287121	21195		10.293	M	4.633	2.722
16.340	10261400	436234		17.963	M	95.367	97.278
	10548521	457429				100.000	100.000

(*R*)-2-(4-(4-bromophenyl)-1,1,1-trifluoro-4-oxobutan-2-yl)pyridazin-3(*2H*)-one

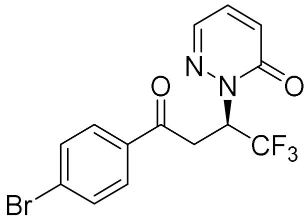

(-)-3ga; isolated yield: 36.8 mg (98%); colorless sticky oil; $[\alpha]_D^{20}$ = -307.4 (*c* = 1.0, $CHCl_3$); Enantiomeric excess: 93%, determined by HPLC (Chiralpak AD-H, hexane/*i*-PrOH = 80/20; flow rate 1.0 ml/min; 25 °C; 254 nm), first peak: t_R = 10.333 min, second peak: t_R = 17.299 min.

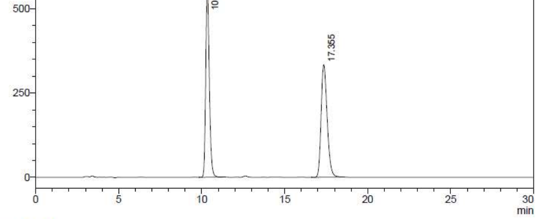

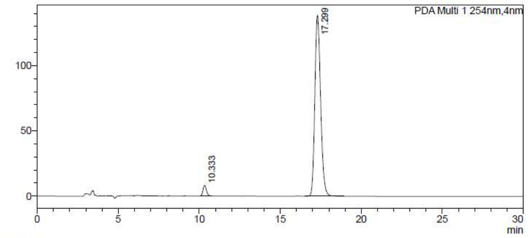

<Peak Table>

PDA Ch1 254nm

Peak#	Ret. Time	Area	Height	Height%	Area%
1	10.341	8433742	554871	62.425	50.001
2	17.355	8433254	333992	37.575	49.999
Total		16866996	888863	100.000	100.000

<Peak Table>

PDA Ch1 254nm

Peak#	Ret. Time	Area	Height	Height%	Area%
1	10.333	118429	8093	5.515	3.329
2	17.299	3439484	138659	94.485	96.671
Total		3557912	146752	100.000	100.000

(*R*)-2-(1,1,1-trifluoro-4-(4-iodophenyl)-4-oxobutan-2-yl)pyridazin-3(*2H*)-one

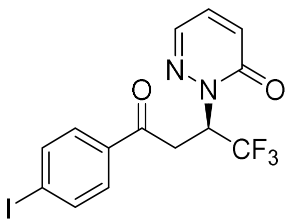

(-)-3ha; isolated yield: 38.0 mg (90%); colorless sticky oil; $[\alpha]_D^{20}$ = -272.9 (*c* = 1.0, $CHCl_3$); Enantiomeric excess: 93%, determined by HPLC (Chiralpak AD-H, hexane/*i*-PrOH = 80/20; flow rate 1.0 ml/min; 25 °C; 254 nm), first peak: t_R = 11.318 min, second peak: t_R = 18.690 min.

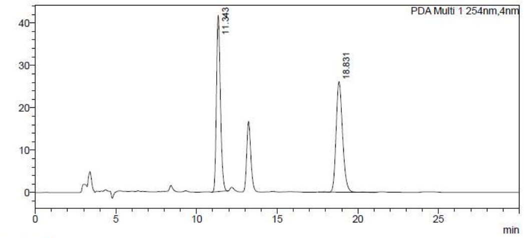

<Peak Table>

PDA Ch1 254nm

Peak#	Ret. Time	Area	Height	Height%	Area%
1	11.343	709379	41554	61.446	49.403
2	18.831	726531	26073	38.554	50.597
Total		1435910	67627	100.000	100.000

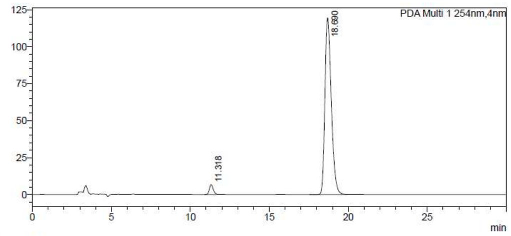

<Peak Table>

PDA Ch1 254nm

Peak#	Ret. Time	Area	Height	Height%	Area%
1	11.318	114602	6683	5.294	3.304
2	18.690	3354515	119547	94.706	96.696
Total		3469117	126230	100.000	100.000

(*R*)-2-(1,1,1-trifluoro-4-(4-nitrophenyl)-4-oxobutan-2-yl)pyridazin-3(*2H*)-one

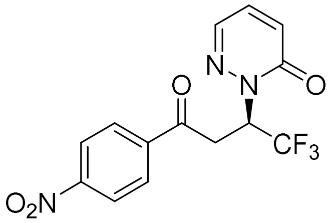

(-)-3ia; isolated yield: 30.0 mg (88%); colorless sticky oil; $[\alpha]_D^{20}$ = -284.1 (*c* = 1.0, $CHCl_3$); Enantiomeric excess: 91%, determined by HPLC (Chiralpak AD-H, hexane/*i*-PrOH = 70/30; flow rate 1.0 ml/min; 25 °C; 254 nm), first peak: t_R = 18.031 min, second peak: t_R = 40.589 min.

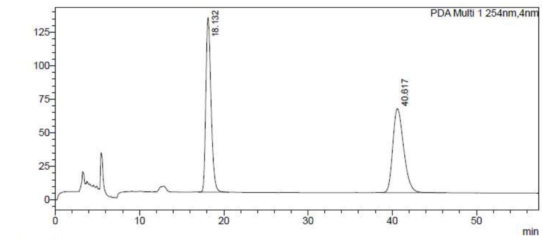

<Peak Table>
PDA Ch1 254nm

Peak#	Ret. Time	Area	Height	Height%	Area%
1	18.132	5580524	130076	67.472	49.800
2	40.617	5625426	62710	32.528	50.200
Total		11205950	192787	100.000	100.000

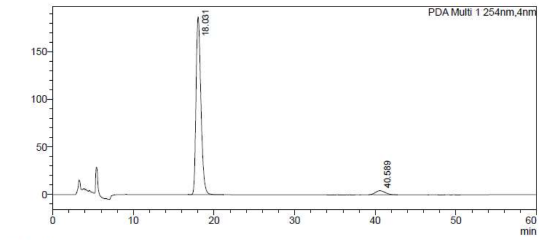

<Peak Table>
PDA Ch1 254nm

Peak#	Ret. Time	Area	Height	Height%	Area%
1	18.031	7865804	186805	97.766	95.641
2	40.589	358520	4269	2.234	4.359
Total		8224324	191073	100.000	100.000

(*R*)-4-(4,4,4-trifluoro-3-(6-oxopyridazin-1(*6H*)-yl)butanoyl)benzonitrile

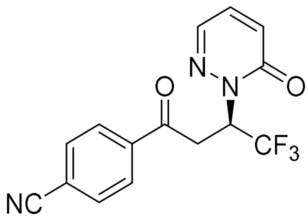

(-)-3ja; isolated yield: 31.5 mg (98%); colorless sticky oil; $[\alpha]_D^{20}$ = -299.2 (*c* = 1.0, $CHCl_3$); Enantiomeric excess: 91%, determined by HPLC (Chiralpak AD-H, hexane/*i*-PrOH = 80/20; flow rate 1.0 ml/min; 25 °C; 254 nm), first peak: t_R = 18.918 min, second peak: t_R = 22.761 min.

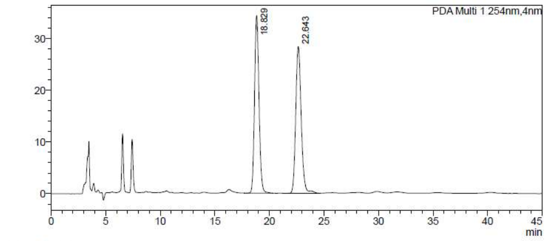

<Peak Table>
PDA Ch1 254nm

Peak#	Ret. Time	Area	Height	Height%	Area%
1	18.829	977187	34363	54.738	49.597
2	22.643	993070	28413	45.262	50.403
Total		1970257	62776	100.000	100.000

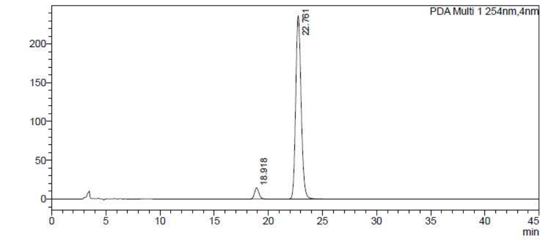

<Peak Table>
PDA Ch1 254nm

Peak#	Ret. Time	Area	Height	Height%	Area%
1	18.918	402851	14308	5.713	4.677
2	22.761	8211087	236121	94.287	95.323
Total		8613938	250429	100.000	100.000

(*R*)-2-(1,1,1-trifluoro-4-(4-(methylsulfonyl)phenyl)-4-oxobutan-2-yl)pyrida-zin-3(*2H*)-one

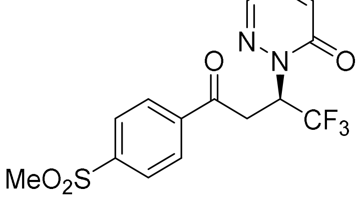

(-)-3ka; isolated yield: 35.9 mg (96%); yellow oil; $[\alpha]_D^{20}$ = -220.1 (*c* = 1.0, $CHCl_3$); Enantiomeric excess: 91%, determined by HPLC (Chiralpak AD-H, hexane/*i*-PrOH =

90/10; flow rate 1.0 ml/min; 25 °C; 254 nm), first peak: t_R = 32.345 min, second peak: t_R = 41.491 min.

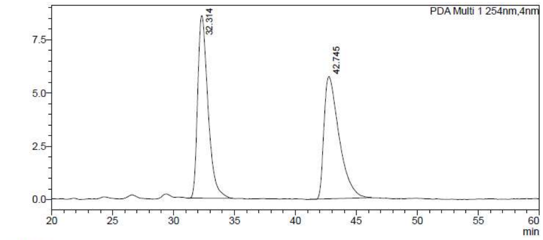

<Peak Table>
PDA Ch1 254nm

Peak#	Ret. Time	Area	Height	Height%	Area%
1	32.314	503991	8567	59.901	50.498
2	42.745	494050	5735	40.099	49.502
Total		998042	14302	100.000	100.000

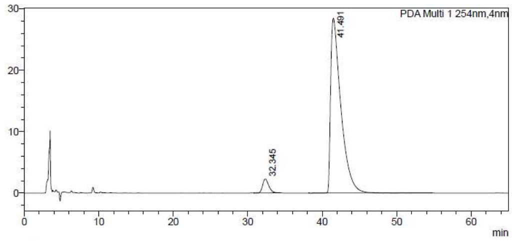

<Peak Table>
PDA Ch1 254nm

Peak#	Ret. Time	Area	Height	Height%	Area%
1	32.345	128812	2279	7.413	4.516
2	41.491	2723663	28465	92.587	95.484
Total		2852475	30744	100.000	100.000

(*R*)-2-(1,1,1-trifluoro-4-oxo-4-(4-(trifluoromethyl)phenyl)butan-2-yl) pyridazin-3(*2H*)-one

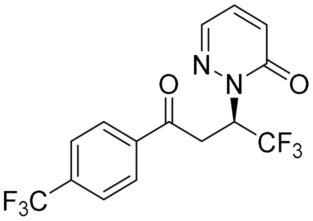

(-)-3la; isolated yield: 32.4 mg (89%); yellow oil; $[\alpha]_D^{20}$ = -278.9 (*c* = 1.0, $CHCl_3$); Enantiomeric excess: 93%, determined by HPLC (Chiralpak AD-H, hexane/*i*-PrOH = 80/20; flow rate 1.0 ml/min; 25 °C; 254 nm), first peak: t_R = 7.929 min, second peak: t_R = 11.515 min.

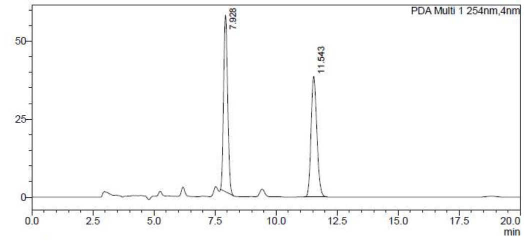

<Peak Table>
PDA Ch1 254nm

Peak#	Ret. Time	Area	Height	Area/Height	Height%	Area%
1	7.928	653609	56606	11.547	59.446	50.594
2	11.543	638265	38616	16.528	40.554	49.406
Total		1291875	95223		100.000	100.000

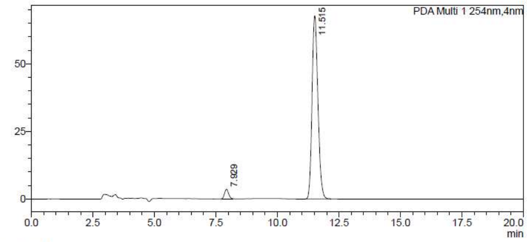

<Peak Table>
PDA Ch1 254nm

Peak#	Ret. Time	Area	Height	Height%	Area%
1	7.929	39319	3487	4.896	3.375
2	11.515	1125750	67733	95.104	96.625
Total		1165069	71220	100.000	100.000

(*R*)-2-(1,1,1-trifluoro-4-(2-nitrophenyl)-4-oxobutan-2-yl)pyridazin-3(*2H*)-one

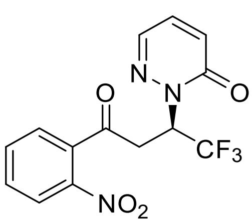

(-)-3ma; isolated yield: 32.4 mg (95%); yellow oil; $[\alpha]_D^{20}$ = -127.3 (*c* = 1.0, $CHCl_3$); Enantiomeric excess: 75%, determined by HPLC (Chiralpak AD-H, hexane/*i*-PrOH = 80/20; flow rate 1.0 ml/min; 25 °C; 254 nm), first peak: t_R = 11.818 min, second peak: t_R = 15.631 min.

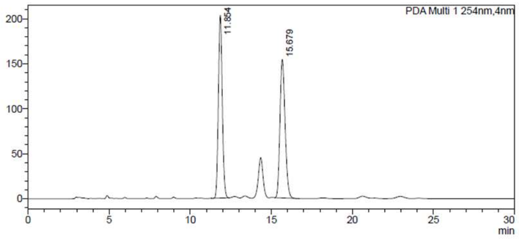

<Peak Table>

PDA Ch1 254nm

Peak#	Ret. Time	Area	Height	Height%	Area/Height	Area%
1	11.854	3402219	202885	56.885	16.769	50.109
2	15.679	3387396	153775	43.115	22.028	49.891
Total		6789615	356660	100.000		100.000

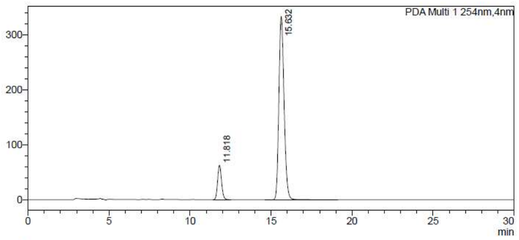

<Peak Table>

PDA Ch1 254nm

Peak#	Ret. Time	Area	Height	Height%	Area/Height	Area%
1	11.818	1051494	62491	15.790	16.826	12.376
2	15.632	7444716	333282	84.210	22.338	87.624
Total		8496210	395773	100.000		100.000

(*R*)-2-(1,1,1-trifluoro-4-(3-nitrophenyl)-4-oxobutan-2-yl)pyridazin-3(*2H*)-one

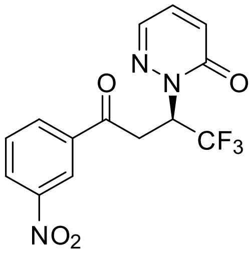

(-)-3na; isolated yield: 32.1 mg (94%); colorless sticky oil; $[\alpha]_D^{20}$ = -263.0 (*c* = 1.0, $CHCl_3$); Enantiomeric excess: 92%, determined by HPLC (Chiralpak AD-H, hexane/*i*-PrOH = 80/20; flow rate 1.0 ml/min; 25 °C; 254 nm), first peak: t_R = 13.409 min, second peak: t_R = 23.927 min.

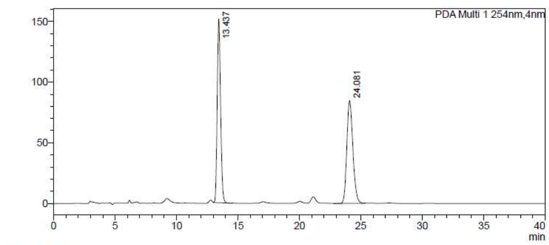

<Peak Table>
PDA Ch1 254nm

Peak#	Ret. Time	Area	Height	Height%	Area%
1	13.437	2878185	151400	64.134	49.842
2	24.081	2896407	84668	35.866	50.158
Total		5774591	236067	100.000	100.000

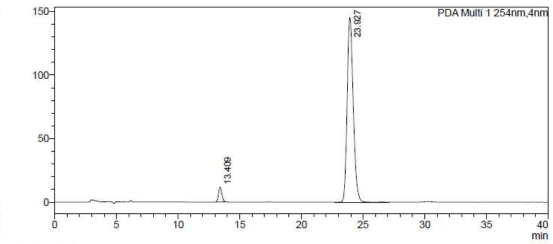

<Peak Table>
PDA Ch1 254nm

Peak#	Ret. Time	Area	Height	Height%	Area%
1	13.409	216195	11719	7.460	4.157
2	23.927	4984462	145364	92.540	95.843
Total		5200657	157083	100.000	100.000

(*R*)-2-(4-(3,5-difluorophenyl)-1,1,1-trifluoro-4-oxobutan-2-yl)pyridazin-3(*2H*)-one

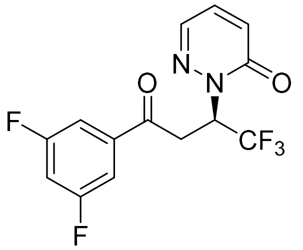

(-)-3oa; isolated yield: 31.0 mg (93%); colorless sticky oil; $[\alpha]_D^{20}$ = -209.6 (*c* = 1.0, $CHCl_3$); Enantiomeric excess: 93%, determined by HPLC (Chiralpak AD-H, hexane/*i*-PrOH = 80/20; flow rate 1.0 ml/min; 25 °C; 254 nm), first peak: t_R = 7.975 min, second peak: t_R = 12.459 min.

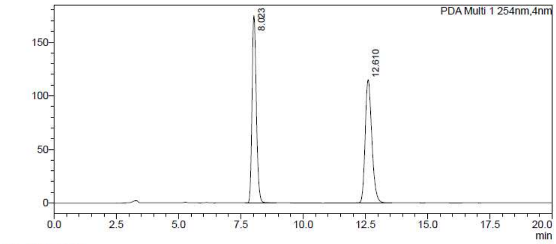

<Peak Table>
PDA Ch1 254nm

Peak#	Ret. Time	Area	Height	Area/Height	Height%	Area%
1	8.023	2096341	174597	12.007	60.319	49.686
2	12.610	2122830	114858	18.482	39.681	50.314
Total		4219171	289455		100.000	100.000

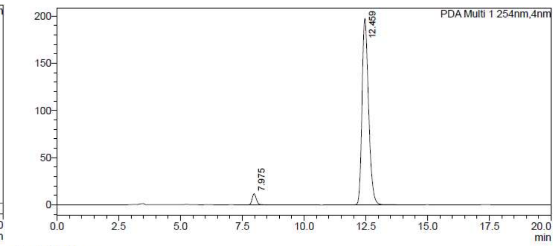

<Peak Table>
PDA Ch1 254nm

Peak#	Ret. Time	Area	Height	Area/Height	Height%	Area%
1	7.975	136017	11737	11.589	5.616	3.606
2	12.459	3635827	197258	18.432	94.384	96.394
Total		3771844	208995		100.000	100.000

(*R*)-2-(4-(3,4-dichlorophenyl)-1,1,1-trifluoro-4-oxobutan-2-yl)pyridazin-3(*2H*)-one

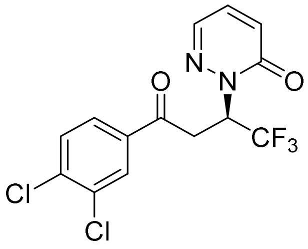

(-)-3pa; isolated yield: 35.8 mg (98%); white solid; $[\alpha]_D^{20}$ = -286.6 (*c* = 1.0, $CHCl_3$); Enantiomeric excess: 95%, determined by HPLC (Chiralpak AD-H, hexane/*i*-PrOH = 80/20; flow rate 1.0 ml/min; 25 °C; 254 nm), first peak: t_R = 7.905 min, second peak: t_R = 11.792 min.

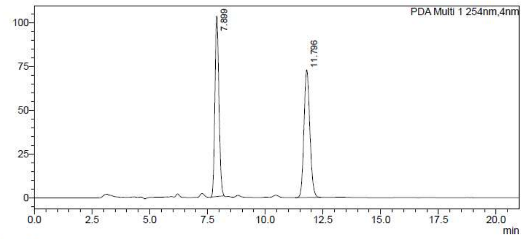

<Peak Table>
PDA Ch1 254nm

Peak#	Ret. Time	Area	Height	Height%	Area%
1	7.899	1244077	102870	58.540	49.086
2	11.796	1290405	72855	41.460	50.914
Total		2534482	175725	100.000	100.000

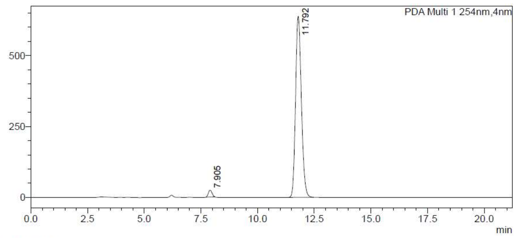

<Peak Table>
PDA Ch1 254nm

Peak#	Ret. Time	Area	Height	Height%	Area%
1	7.905	271124	24203	3.657	2.325
2	11.792	11391847	637666	96.343	97.675
Total		11662971	661869	100.000	100.000

(*R*)-2-(1,1,1-trifluoro-4-(naphthalen-1-yl)-4-oxobutan-2-yl)pyridazin-3(*2H*)-one

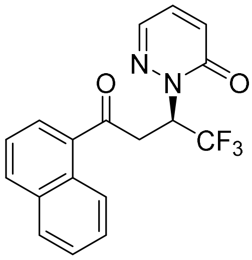

(-)-3qa; isolated yield: 33.9 mg (98%); colorless sticky oil; $[\alpha]_D^{20}$ = -181.2 (*c* = 1.0, $CHCl_3$); Enantiomeric excess: 85%, determined by HPLC (Chiralpak AD-H, hexane/*i*-PrOH = 80/20; flow rate 1.0 ml/min; 25 °C; 254 nm), first peak: t_R = 8.340 min, second peak: t_R = 10.875 min.

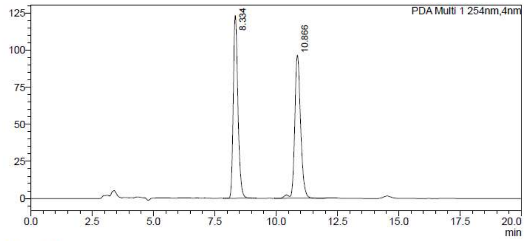

<Peak Table>
PDA Ch1 254nm

Peak#	Ret. Time	Area	Height	Height%	Area%
1	8.334	1629785	123371	56.137	50.464
2	10.866	1599783	96398	43.863	49.536
Total		3229569	219768	100.000	100.000

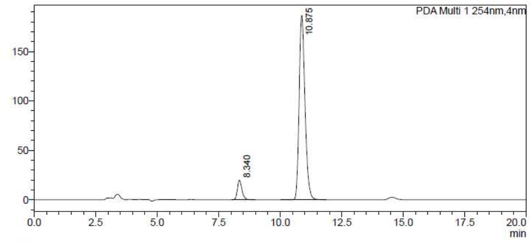

<Peak Table>
PDA Ch1 254nm

Peak#	Ret. Time	Area	Height	Height%	Area%
1	8.340	254968	19802	9.608	7.627
2	10.875	3088022	186307	90.392	92.373
Total		3342990	206109	100.000	100.000

(*R*)-2-(1,1,1-trifluoro-4-(naphthalen-2-yl)-4-oxobutan-2-yl)pyridazin-3(*2H*)

-one

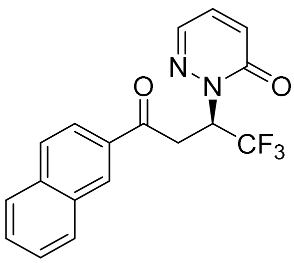

(-)-3ra; isolated yield: 33.9 mg (98%); colorless sticky oil; $[\alpha]_D^{20}$ = -422.3 (*c* = 1.0, $CHCl_3$); Enantiomeric excess: 93%, determined by HPLC (Chiralpak AD-H, hexane/*i*-PrOH = 80/20; flow rate 1.0 ml/min; 25 °C; 254 nm), first peak: t_R = 10.523 min, second peak: t_R = 14.709 min.

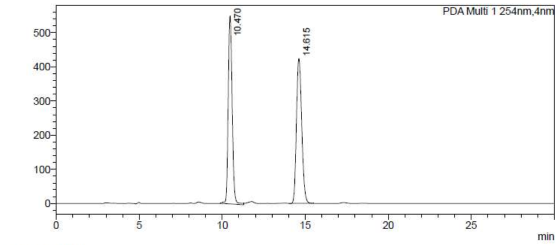

<Peak Table>
PDA Ch1 254nm

Peak#	Ret. Time	Area	Height	Height%	Area%
1	10.470	9085732	550426	56.460	49.226
2	14.615	9371508	424476	43.540	50.774
Total		18457239	974902	100.000	100.000

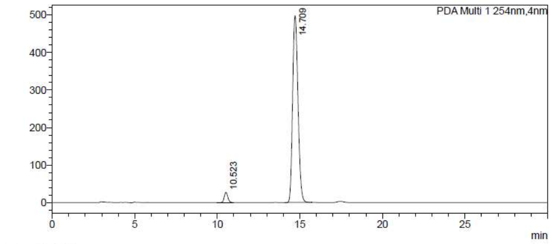

<Peak Table>
PDA Ch1 254nm

Peak#	Ret. Time	Area	Height	Height%	Area%
1	10.523	404213	27164	5.169	3.464
2	14.709	11265039	498373	94.831	96.536
Total		11669252	525537	100.000	100.000

(*R*)-2-(4-(benzo[b]thiophen-2-yl)-1,1,1-trifluoro-4-oxobutan-2-yl)pyridazin-3(*2H*)-one

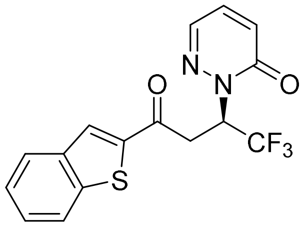

(-)-3sa; isolated yield: 34.1 mg (97%); colorless sticky oil; $[\alpha]_D^{20}$ = -384.1 (*c* = 1.0, $CHCl_3$); Enantiomeric excess: 92%, determined by HPLC (Chiralpak AD-H, hexane/*i*-PrOH = 80/20; flow rate 1.0 ml/min; 25 °C; 254 nm), first peak: t_R = 12.571 min, second peak: t_R = 17.941 min.

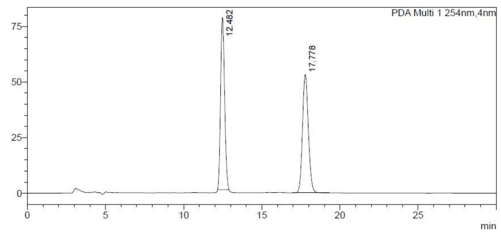

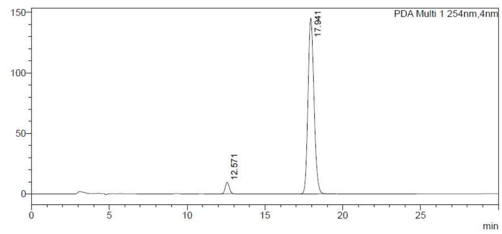

<Peak Table>

PDA Ch1 254nm

Peak#	Ret. Time	Area	Height	Height%	Area%
1	12.482	1387564	77414	59.260	50.025
2	17.778	1386179	53221	40.740	49.975
Total		2773743	130635	100.000	100.000

<Peak Table>

PDA Ch1 254nm

Peak#	Ret. Time	Area	Height	Height%	Area%
1	12.571	171555	9469	6.120	4.217
2	17.941	3896939	145247	93.880	95.783
Total		4068495	154716	100.000	100.000

(*R*)-2-(1,1,1-trifluoro-4-oxo-4-(thiophen-2-yl)butan-2-yl)pyridazin-3(*2H*)-one

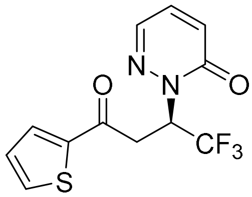

(-)-3ta; isolated yield: 28.7 mg (95%); yellow oil; $[\alpha]_D^{20}$ = -278.5 (*c* = 1.0, $CHCl_3$); Enantiomeric excess: 96%, determined by HPLC (Chiralpak AD-H, hexane/*i*-PrOH = 80/20; flow rate 1.0 ml/min; 25 °C; 254 nm), first peak: t_R = 10.632 min, second peak: t_R = 15.932 min.

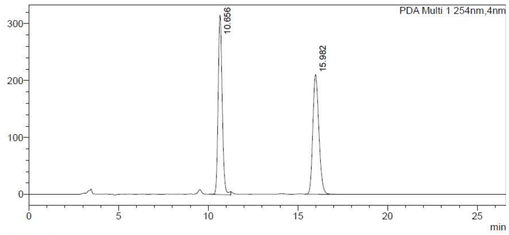

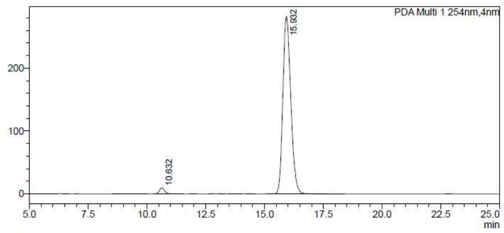

<Peak Table>

PDA Ch1 254nm

Peak#	Ret. Time	Area	Height	Height%	Area%
1	10.656	4960293	315319	60.001	50.181
2	15.982	4924586	210205	39.999	49.819
Total		9884879	525524	100.000	100.000

<Peak Table>

PDA Ch1 254nm

Peak#	Ret. Time	Area	Height	Height%	Area%
1	10.632	142083	9392	3.222	2.104
2	15.932	6610519	282115	96.778	97.896
Total		6752602	291507	100.000	100.000

(*R*)-2-(4-(cyclohex-1-en-1-yl)-1,1,1-trifluoro-4-oxobutan-2-yl)pyridazin-3(*2H*)-one

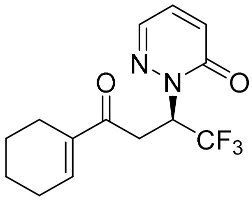

(-)-3ua; isolated yield: 12.0 mg (40%); colorless sticky oil; $[\alpha]_D^{20}$ = -235.5 (*c* = 0.33, $CHCl_3$); Enantiomeric excess: 90%, determined by HPLC (Chiralpak AD-H,

hexane/*i*-PrOH = 80/20; flow rate 1.0 ml/min; 25 °C; 254 nm), first peak: t_R = 7.266 min, second peak: t_R = 9.892 min.

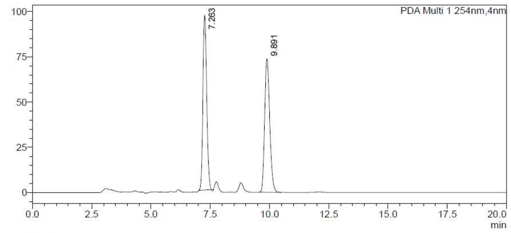

<Peak Table>
PDA Ch1 254nm

Peak#	Ret. Time	Area	Height	Conc.	Height%	Area%
1	7.263	1066238	96603	0.000	56.676	49.164
2	9.891	1102485	73844	0.000	43.324	50.836
Total		2168723	170447		100.000	100.000

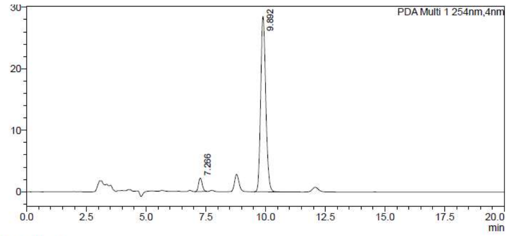

<Peak Table>
PDA Ch1 254nm

Peak#	Ret. Time	Area	Height	Height%	Area%
1	7.266	23549	2194	7.143	5.274
2	9.892	422933	28516	92.857	94.726
Total		446482	30710	100.000	100.000

(*R*)-2-(1,1,1,2,2-pentafluoro-5-oxo-5-phenylpentan-3-yl)pyridazin-3(*2H*)-One

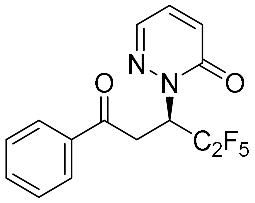

(-)-3ua; isolated yield: 22.0 mg (64%); colorless sticky oil; Enantiomeric excess: 83%, determined by HPLC (Chiralpak AD-H, hexane/*i*-PrOH = 80/20; flow rate 1.0 ml/min; 25 °C; 254 nm), first peak: t_R = 7.857 min, second peak: t_R = 8.661 min.

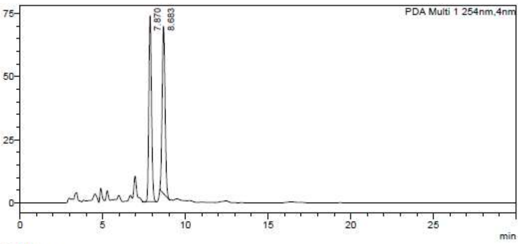

<Peak Table>
PDA Ch1 254nm

Peak#	Ret. Time	Area	Height	Height%	Area%
1	7.870	831648	73570	52.563	50.333
2	8.683	820645	66395	47.437	49.667
Total		1652293	139966	100.000	100.000

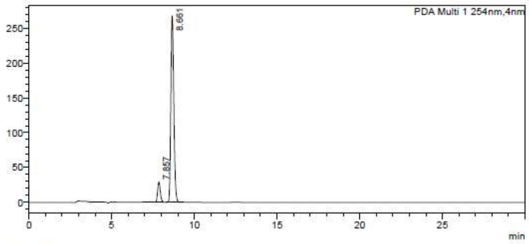

<Peak Table>
PDA Ch1 254nm

Peak#	Ret. Time	Area	Height	Height%	Area%
1	7.857	317239	28776	9.712	8.660
2	8.661	3345826	267520	90.288	91.340
Total		3663065	296297	100.000	100.000

(*R*)-2-(4-(4-chlorophenyl)-1,1,1-trifluoro-4-oxobutan-2-yl)-6-methylpyrida-zin-3(*2H*)-one

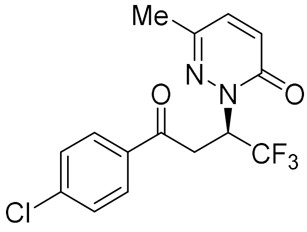

(-)-3fb; isolated yield: 22.0 mg (64%); white solid; $[\alpha]_D^{20}$ = -222.3 (*c* = 1.0, $CHCl_3$);

Enantiomeric excess: 96%, determined by HPLC (Chiralpak AD-H, hexane/*i*-PrOH = 80/20; flow rate 1.0 ml/min; 25 °C; 254 nm), first peak: t_R = 9.167 min, second peak: t_R = 12.150 min.

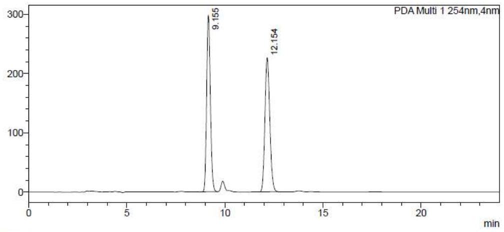

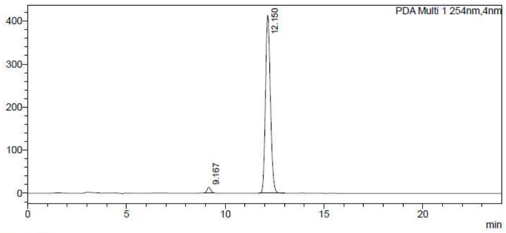

<Peak Table>

PDA Ch1 254nm

Peak#	Ret. Time	Area	Height	Conc.	Height%	Area%
1	9.155	3938897	298276	0.000	56.830	50.091
2	12.154	3924609	226581	0.000	43.170	49.909
Total		7863506	524858		100.000	100.000

<Peak Table>

PDA Ch1 254nm

Peak#	Ret. Time	Area	Height	Height%	Area%
1	9.167	160719	12842	3.013	2.182
2	12.150	7204111	413440	96.987	97.818
Total		7364830	426282	100.000	100.000

(*R*)-2-(4-(4-chlorophenyl)-1,1,1-trifluoro-4-oxobutan-2-yl)-6-phenylpyrida-zin-3(*2H*)-one

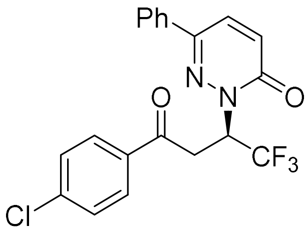

(-)-3fc; isolated yield: 39.5 mg (97%); colorless sticky oil; $[\alpha]_D^{20}$ = -76.6 (*c* = 1.0, $CHCl_3$); Enantiomeric excess: 92%, determined by HPLC (Chiralpak AD-H, hexane/*i*-PrOH = 80/20; flow rate 1.0 ml/min; 25 °C; 254 nm), first peak: t_R = 9.624 min, second peak: t_R = 11.733 min.

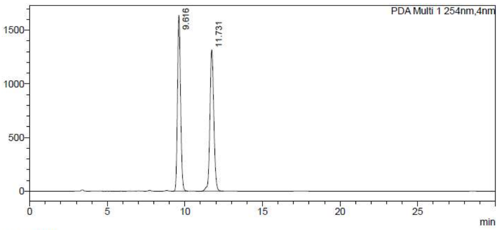

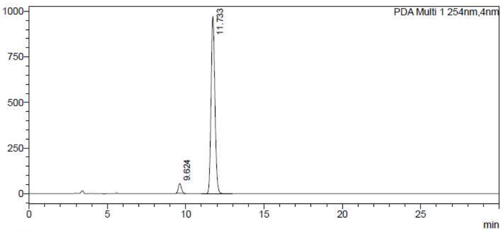

<Peak Table>

PDA Ch1 254nm

Peak#	Ret. Time	Area	Height	Height%	Area%
1	9.616	22583185	1636563	55.399	49.497
2	11.731	23041728	1317565	44.601	50.503
Total		45624912	2954127	100.000	100.000

<Peak Table>

PDA Ch1 254nm

Peak#	Ret. Time	Area	Height	Height%	Area%
1	9.624	718152	53996	5.259	4.132
2	11.733	16662682	972673	94.741	95.868
Total		17380833	1026669	100.000	100.000

(*R*)-6-chloro-2-(4-(4-chlorophenyl)-1,1,1-trifluoro-4-oxobutan-2-yl)pyrida-zin-3(*2H*)-one

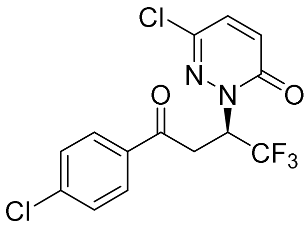

(-)-3fd; isolated yield: 35.0 mg (96%); white solid; $[\alpha]_D^{20}$ = -188.3 (c = 1.0, $CHCl_3$); Enantiomeric excess: 92%, determined by HPLC (Chiralpak AD-H, hexane/*i*-PrOH = 80/20; flow rate 1.0 ml/min; 25 °C; 254 nm), first peak: t_R = 8.770 min, second peak: t_R = 11.389 min.

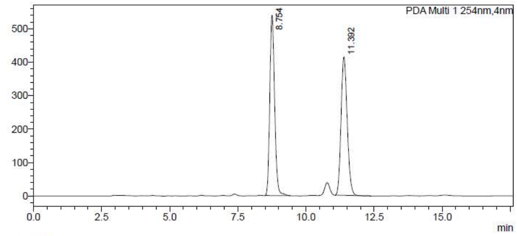

<Peak Table>

PDA Ch1 254nm

Peak#	Ret. Time	Area	Height	Height%	Area%
1	8.754	6649519	539803	56.581	50.508
2	11.392	6515838	414239	43.419	49.492
Total		13165356	954042	100.000	100.000

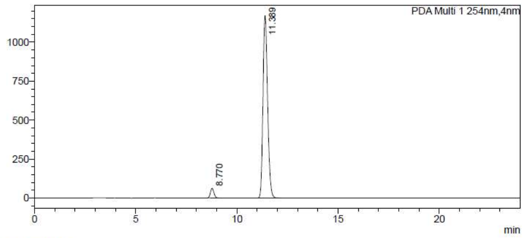

<Peak Table>

PDA Ch1 254nm

Peak#	Ret. Time	Area	Height	Height%	Area%
1	8.770	745483	62341	5.056	3.858
2	11.389	18577460	1170550	94.944	96.142
Total		19322943	1232890	100.000	100.000

Methyl (*R*)-1-(4-(4-chlorophenyl)-1,1,1-trifluoro-4-oxobutan-2-yl)-6-oxo-1,6-dihydropyridazine-3-carboxylate

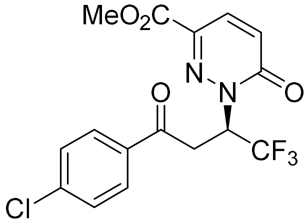

(-)-3fe; isolated yield: 38.1 mg (98%); white solid; $[\alpha]_D^{20}$ = -228.9 (c = 1.0, $CHCl_3$); Enantiomeric excess: 90%, determined by HPLC (Chiralpak AD-H, hexane/*i*-PrOH = 80/20; flow rate 1.0 ml/min; 25 °C; 254 nm), first peak: t_R = 10.047 min, second peak: t_R = 13.182 min.

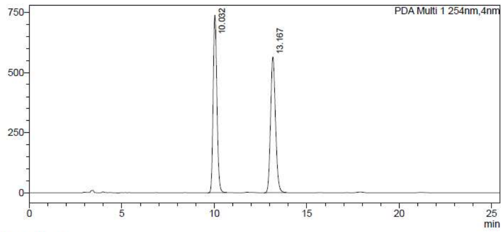

<Peak Table>

PDA Ch1 254nm

Peak#	Ret. Time	Area	Height	Height%	Area%
1	10.032	10447672	738152	56.626	49.879
2	13.167	10498273	565407	43.374	50.121
Total		20945945	1303558	100.000	100.000

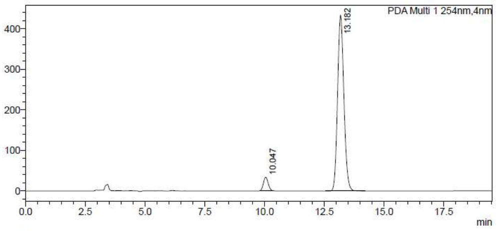

<Peak Table>

PDA Ch1 254nm

Peak#	Ret. Time	Area	Height	Height%	Area%
1	10.047	442801	32935	7.057	5.219
2	13.182	8042208	433798	92.943	94.781
Total		8485009	466733	100.000	100.000

(*R*)-5-chloro-2-(4-(4-chlorophenyl)-1,1,1-trifluoro-4-oxobutan-2-yl)pyridazin-3(*2H*)-one

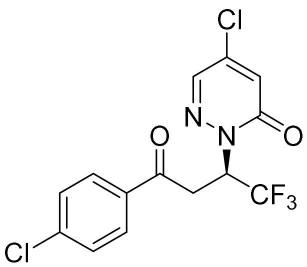

(-)-3ff; isolated yield: 35.4 mg (97%); colorless sticky oil; $[\alpha]_D^{20}$ = -257.0 (*c* = 1.0, $CHCl_3$); Enantiomeric excess: 92%, determined by HPLC (Chiralpak AD-H, hexane/*i*-PrOH = 80/20; flow rate 1.0 ml/min; 25 °C; 254 nm), first peak: t_R = 8.833 min, second peak: t_R = 16.246 min.

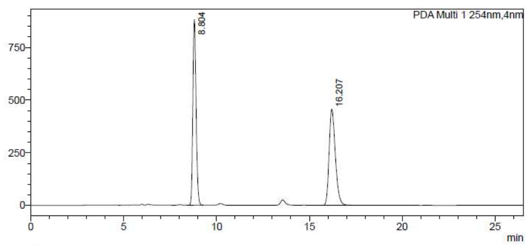

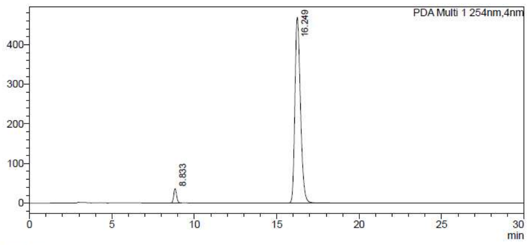

<Peak Table>
PDA Ch1 254nm

Peak#	Ret. Time	Area	Height	Height%	Area%
1	8.804	10629799	881642	65.894	49.965
2	16.207	10644802	456333	34.106	50.035
Total		21274601	1337975	100.000	100.000

<Peak Table>
PDA Ch1 254nm

Peak#	Ret. Time	Area	Height	Height%	Area%
1	8.833	433556	36293	7.184	3.792
2	16.249	11000964	468926	92.816	96.208
Total		11434520	505218	100.000	100.000

Methyl (*R*)-4-(4-chlorophenyl)-4-oxo-2-(6-oxopyridazin-1(*6H*)-yl)butanoate

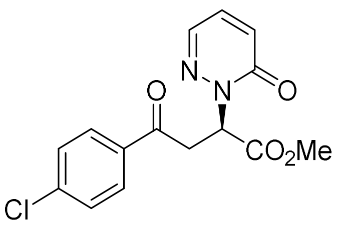

(-)-5aa; isolated yield: 27.2 mg (85%); colorless sticky oil; $[\alpha]_D^{20}$ = -17.1 (*c* = 1.0, $CHCl_3$); Enantiomeric excess: 90%, determined by HPLC (Chiralpak AD-H, hexane/*i*-PrOH = 70/30; flow rate 1.0 ml/min; 25 °C; 254 nm), first peak: t_R = 17.67 min, second peak: t_R = 21.60 min.

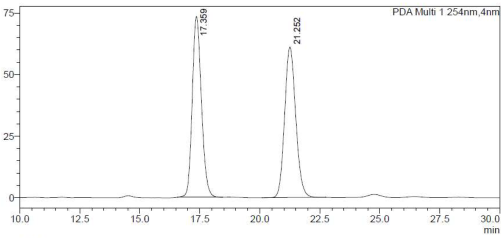

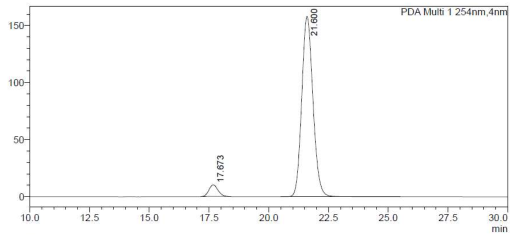

<Peak Table>
PDA Ch1 254nm

Peak#	Ret. Time	Area	Height	Area/Height	Height%	Area%
1	17.359	1949809	73412	26.560	54.558	49.767
2	21.252	1968097	61146	32.187	45.442	50.233
Total		3917906	134558		100.000	100.000

<Peak Table>
PDA Ch1 254nm

Peak#	Ret. Time	Area	Height	Area/Height	Height%	Area%
1	17.673	268458	10238	26.222	6.089	4.950
2	21.600	5154489	157897	32.645	93.911	95.050
Total		5422947	168135		100.000	100.000

Benzyl (*R*)-4-oxo-2-(6-oxopyridazin-1(*6H*)-yl)-4-phenylbutanoate

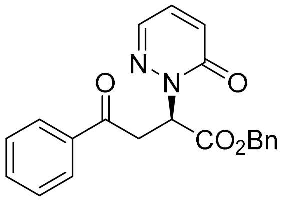

(-)-5ba; isolated yield: 27.2 mg (75%); colorless sticky oil; $[\alpha]_D^{20}$ = -6.2 (*c* = 1.0, $CHCl_3$); Enantiomeric excess: 89%, determined by HPLC (Chiralpak AD-H, hexane/*i*-PrOH = 70/30; flow rate 1.0 ml/min; 25 °C; 254 nm), first peak: t_R = 20.88min, second peak: t_R = 27.51 min.

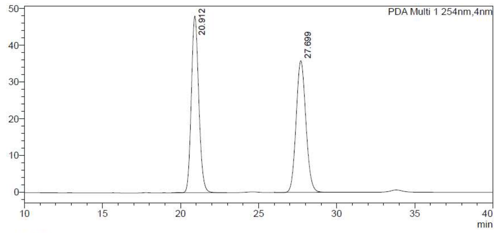

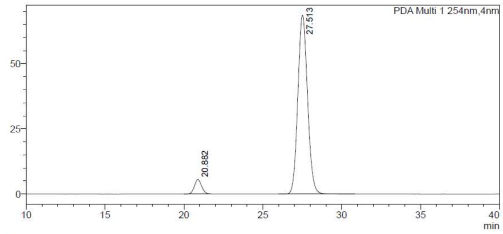

<Peak Table>
PDA Ch1 254nm

Peak#	Ret. Time	Area	Height	Area/Height	Height%	Area%
1	20.912	1593094	48044	33.159	57.275	50.539
2	27.699	1559090	35839	43.503	42.725	49.461
Total		3152184	83883		100.000	100.000

<Peak Table>
PDA Ch1 254nm

Peak#	Ret. Time	Area	Height	Area/Height	Height%	Area%
1	20.882	176181	5523	31.902	7.451	5.670
2	27.513	2930902	68595	42.728	92.549	94.330
Total		3107083	74117		100.000	100.000

Ethyl (*R*)-4-oxo-2-(6-oxopyridazin-1(*6H*)-yl)-4-phenylbutanoate

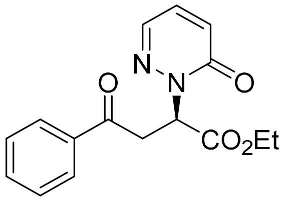

(-)-5ca; isolated yield: 26.1 mg (87%); colorless sticky oil; $[\alpha]_D^{20}$ = -9.0 (*c* = 1.0, $CHCl_3$); Enantiomeric excess: 93%, determined by HPLC (Chiralpak AD-H, hexane/*i*-PrOH = 70/30; flow rate 1.0 ml/min; 25 °C; 254 nm), first peak: t_R = 11.60

min, second peak: t_R = 13.86 min.

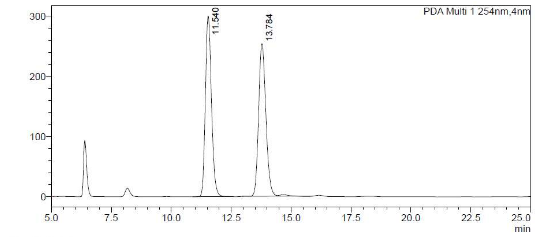

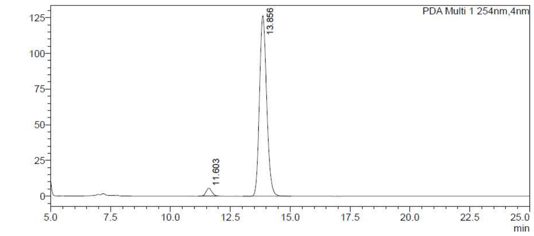

<Peak Table>
PDA Ch1 254nm

Peak#	Ret. Time	Area	Height	Area/Height	Height%	Area%
1	11.540	5298874	300798	17.616	54.217	49.463
2	13.784	5413830	254006	21.314	45.783	50.537
Total		10712704	554803		100.000	100.000

<Peak Table>
PDA Ch1 254nm

Peak#	Ret. Time	Area	Height	Area/Height	Height%	Area%
1	11.603	94143	5552	16.958	4.205	3.429
2	13.856	2651535	126474	20.965	95.795	96.571
Total		2745678	132026		100.000	100.000

isopropyl (*R*)-4-oxo-2-(6-oxopyridazin-1(*6H*)-yl)-4-phenylbutanoate

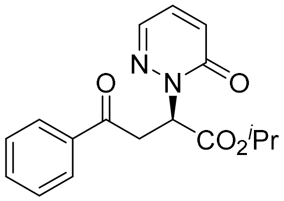

(-)-5da; isolated yield: 30.1 mg (96%); colorless sticky oil; $[\alpha]_D^{20}$ = -11.7 (*c* = 1.0, $CHCl_3$); Enantiomeric excess: 96%, determined by HPLC (Chiralpak AD-H, hexane/*i*-PrOH = 70/30; flow rate 1.0 ml/min; 25 °C; 254 nm), first peak: t_R = 11.30 min, second peak: t_R = 13.18 min.

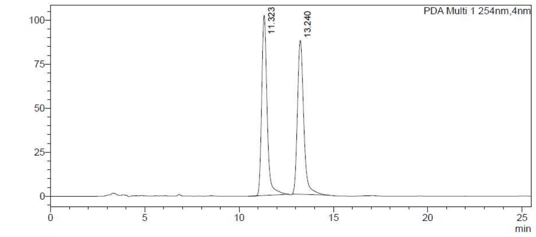

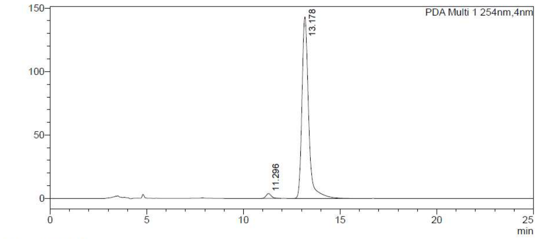

<Peak Table>
PDA Ch1 254nm

Peak#	Ret. Time	Area	Height	Area/Height	Height%	Area%
1	11.323	2039585	102219	19.953	53.903	50.167
2	13.240	2025991	87415	23.177	46.097	49.833
Total		4065575	189635		100.000	100.000

<Peak Table>
PDA Ch1 254nm

Peak#	Ret. Time	Area	Height	Area/Height	Height%	Area%
1	11.296	71074	3797	18.719	2.584	2.037
2	13.178	3418452	143141	23.882	97.416	97.963
Total		3489526	146938		100.000	100.000

tert-butyl (*R*)-4-oxo-2-(6-oxopyridazin-1(*6H*)-yl)-4-phenylbutanoate

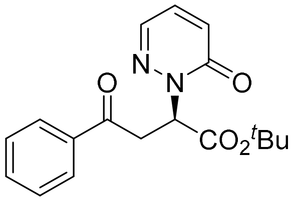

(-)-5ea; isolated yield: 30.5 mg (93%); colorless sticky oil; $[\alpha]_D^{20}$ = -6.5 (*c* = 1.0, $CHCl_3$); Enantiomeric excess: 97%, determined by HPLC (Chiralpak AD-H, hexane/*i*-PrOH = 70/30; flow rate 1.0 ml/min; 25 °C; 254 nm), first peak: t_R = 8.35

min, second peak: t_R = 11.40 min.

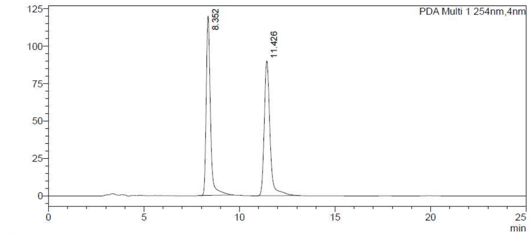

<Peak Table>
PDA Ch1 254nm

Peak#	Ret. Time	Area	Height	Area/Height	Height%	Area%
1	8.352	1849900	119895	15.429	57.096	49.434
2	11.426	1892233	90094	21.003	42.904	50.566
Total		3742132	209989		100.000	100.000

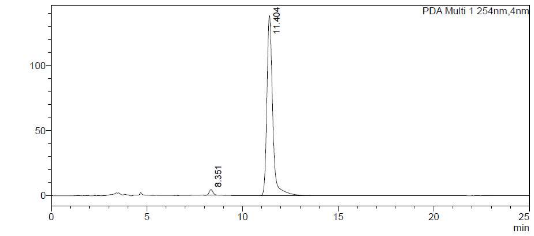

<Peak Table>
PDA Ch1 254nm

Peak#	Ret. Time	Area	Height	Area/Height	Height%	Area%
1	8.351	53239	4167	12.776	2.925	1.798
2	11.404	2907588	138315	21.021	97.075	98.202
Total		2960827	142482		100.000	100.000

Ethyl (*R*)-4-oxo-2-(6-oxopyridazin-1(*6H*)-yl)-4-(*p*-tolyl)butanoate

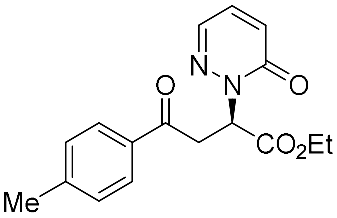

(-)-5fa; isolated yield: 22.0 mg (70%); colorless sticky oil; $[\alpha]_D^{20}$ = -22.5 (c = 1.0, $CHCl_3$); Enantiomeric excess: 96%, determined by HPLC (Chiralpak AD-H, hexane/*i*-PrOH = 70/30; flow rate 1.0 ml/min; 25 °C; 254 nm), first peak: t_R = 12.88 min, second peak: t_R = 16.06 min.

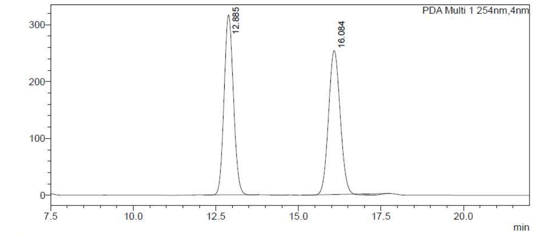

<Peak Table>
PDA Ch1 254nm

Peak#	Ret. Time	Area	Height	Area/Height	Height%	Area%
1	12.885	6229300	316534	19.680	55.546	50.425
2	16.084	6124251	253322	24.176	44.454	49.575
Total		12353551	569856		100.000	100.000

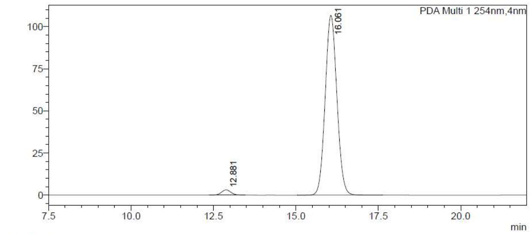

<Peak Table>
PDA Ch1 254nm

Peak#	Ret. Time	Area	Height	Area/Height	Height%	Area%
1	12.881	60934	3087	19.740	2.811	2.262
2	16.061	2633005	106713	24.674	97.189	97.738
Total		2693939	109800		100.000	100.000

Ethyl (*R*)-4-(4-methoxyphenyl)-4-oxo-2-(6-oxopyridazin-1(6H)-yl)butanoate

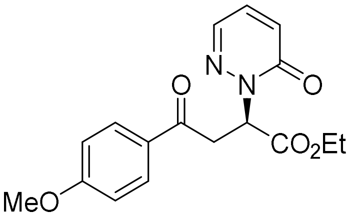

(-)-5ga; isolated yield: 18 mg (55%); colorless sticky oil; $[\alpha]_D^{20}$ = -35.5 (c = 0.33,

$CHCl_3$); Enantiomeric excess: 95%, determined by HPLC (Chiralpak AD-H, hexane/*i*-PrOH = 70/30; flow rate 1.0 ml/min; 25 °C; 254 nm), first peak: t_R = 17.26 min, second peak: t_R = 24.46 min.

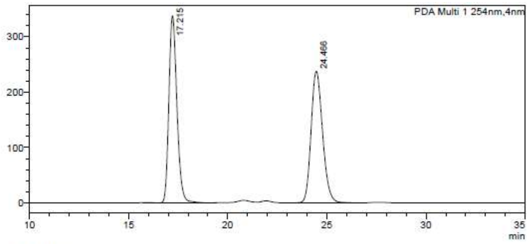

<Peak Table>
PDA Ch1 254nm

Peak#	Ret. Time	Area	Height	Area/Height	Height%	Area%
1	17.215	9470247	337391	28.069	58.673	49.784
2	24.466	9552595	237641	40.198	41.327	50.216
Total		19022842	575032		100.000	100.000

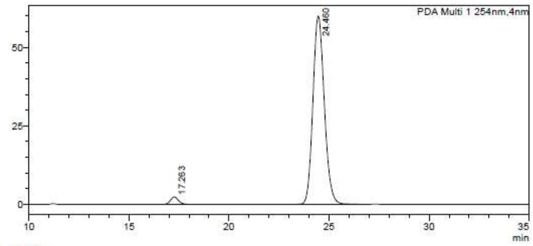

<Peak Table>
PDA Ch1 254nm

Peak#	Ret. Time	Area	Height	Area/Height	Height%	Area%
1	17.263	63552	2330	27.270	3.739	2.602
2	24.460	2378459	60007	39.636	96.261	97.398
Total		2442011	62338		100.000	100.000

Ethyl (*R*)-4-([1,1'-biphenyl]-4-yl)-4-oxo-2-(6-oxopyridazin-1(*6H*)-yl)-butanoate

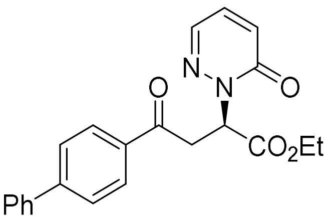

(-)-5ha; isolated yield: 27.1 mg (72%); colorless sticky oil; $[\alpha]_D^{20}$ = -56.9 (*c* = 1.0, $CHCl_3$); Enantiomeric excess: 95%, determined by HPLC (Chiralpak AD-H, hexane/*i*-PrOH = 70/30; flow rate 1.0 ml/min; 25 °C; 254 nm), first peak: t_R = 18.81 min, second peak: t_R = 33.07 min.

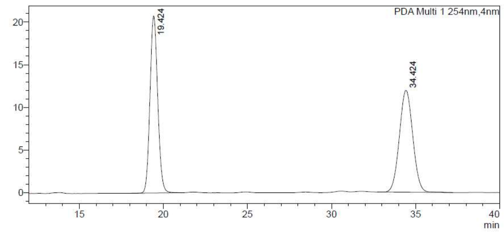

<Peak Table>
PDA Ch1 254nm

Peak#	Ret. Time	Area	Height	Area/Height	Height%	Area%
1	19.424	661400	20750	31.874	63.457	49.943
2	34.424	662921	11950	55.476	36.543	50.057
Total		1324321	32700		100.000	100.000

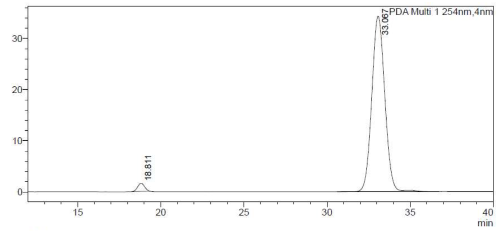

<Peak Table>
PDA Ch1 254nm

Peak#	Ret. Time	Area	Height	Area/Height	Height%	Area%
1	18.811	45763	1587	28.838	4.420	2.394
2	33.067	1865830	34314	54.375	95.580	97.606
Total		1911593	35901		100.000	100.000

Ethyl (*R*)-4-(4-fluorophenyl)-4-oxo-2-(6-oxopyridazin-1(*6H*)-yl)butanoate

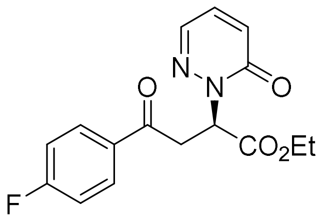

(-)-5ia; isolated yield: 28.9 mg (91%); colorless sticky oil; $[\alpha]_D^{20}$ = -2.0 (*c* = 1.0, $CHCl_3$); Enantiomeric excess: 94%, determined by HPLC (Chiralpak AD-H, hexane/*i*-PrOH = 70/30; flow rate 1.0 ml/min; 25 °C; 254 nm), first peak: t_R = 14.33 min, second peak: t_R = 17.68 min.

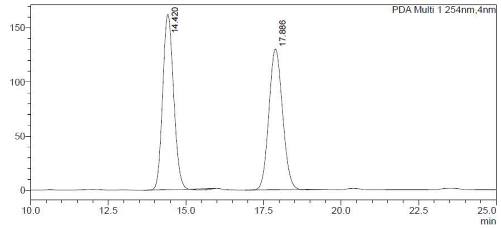

<Peak Table>
PDA Ch1 254nm

Peak#	Ret. Time	Area	Height	Area/Height	Height%	Area%
1	14.420	3933710	161873	24.301	55.449	49.761
2	17.886	3971482	130057	30.537	44.551	50.239
Total		7905192	291930		100.000	100.000

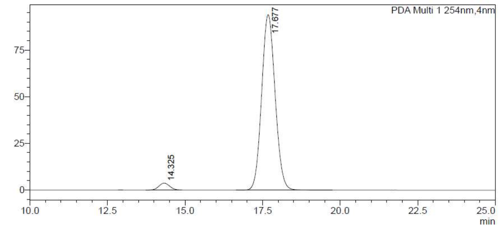

<Peak Table>
PDA Ch1 254nm

Peak#	Ret. Time	Area	Height	Area/Height	Height%	Area%
1	14.325	89363	3724	23.996	3.811	3.053
2	17.677	2837663	93986	30.192	96.189	96.947
Total		2927025	97710		100.000	100.000

Ethyl (*R*)-4-(4-chlorophenyl)-4-oxo-2-(6-oxopyridazin-1(*6H*)-yl)butanoate

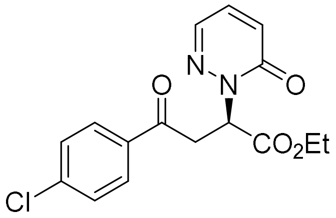

(-)-5ja; isolated yield: 30.2 mg (90%); colorless sticky oil; $[\alpha]_D^{20}$ = -20.6 (*c* = 1.0, $CHCl_3$); Enantiomeric excess: 95%, determined by HPLC (Chiralpak AD-H, hexane/*i*-PrOH = 70/30; flow rate 1.0 ml/min; 25 °C; 254 nm), first peak: t_R = 17.85 min, second peak: t_R = 22.48 min.

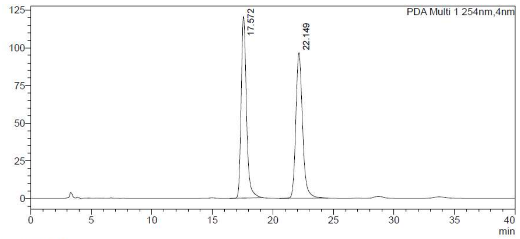

<Peak Table>
PDA Ch1 254nm

Peak#	Ret. Time	Area	Height	Area/Height	Height%	Area%
1	17.572	3668373	120666	30.401	55.526	49.628
2	22.149	3723406	96647	38.526	44.474	50.372
Total		7391779	217313		100.000	100.000

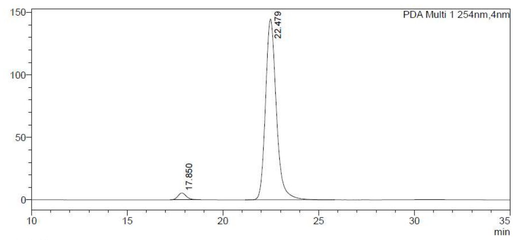

<Peak Table>
PDA Ch1 254nm

Peak#	Ret. Time	Area	Height	Area/Height	Height%	Area%
1	17.850	159502	5394	29.570	3.597	2.732
2	22.479	5679614	144580	39.283	96.403	97.268
Total		5839116	149975		100.000	100.000

Ethyl (*R*)-4-(4-bromophenyl)-4-oxo-2-(6-oxopyridazin-1(*6H*)-yl)butanoate

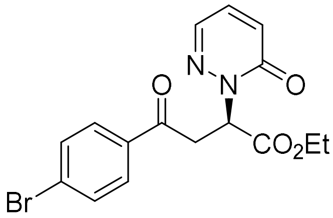

(-)-5ka; isolated yield: 34.9 mg (92%); colorless sticky oil; $[\alpha]_D^{20}$ = -25.4 (*c* = 1.0, $CHCl_3$); Enantiomeric excess: 94%, determined by HPLC (Chiralpak AD-H, hexane/*i*-PrOH = 70/30; flow rate 1.0 ml/min; 25 °C; 254 nm), first peak: t_R = 16.58 min, second peak: t_R = 21.28 min.

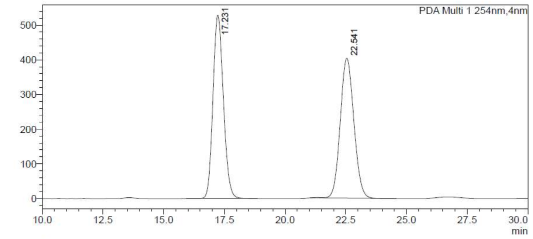

<Peak Table>
PDA Ch1 254nm

Peak#	Ret. Time	Area	Height	Area/Height	Height%	Area%
1	17.231	15749531	528339	29.810	56.716	50.009
2	22.541	15743581	403209	39.046	43.284	49.991
Total		31493112	931548		100.000	100.000

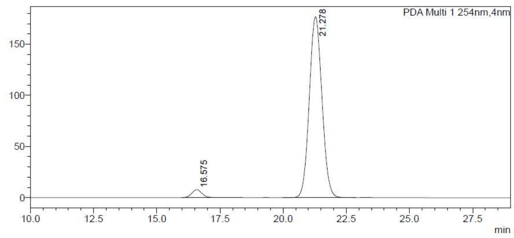

<Peak Table>
PDA Ch1 254nm

Peak#	Ret. Time	Area	Height	Area/Height	Height%	Area%
1	16.575	207670	7651	27.144	4.147	3.259
2	21.278	6164614	176819	34.864	95.853	96.741
Total		6372284	184469		100.000	100.000

Ethyl (*R*)-4-oxo-2-(6-oxopyridazin-1(*6H*)-yl)-4-(4-(trifluoromethyl)phenyl)-butanoate

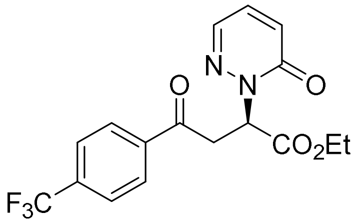

(-)-5la; isolated yield: 25.8 mg (70%); colorless sticky oil; $[\alpha]_D^{20}$ = -3.4 (*c* = 1.0, $CHCl_3$); Enantiomeric excess: 94%, determined by HPLC (Chiralpak AD-H, hexane/*i*-PrOH = 70/30; flow rate 1.0 ml/min; 25 °C; 254 nm), first peak: t_R = 8.79 min, second peak: t_R = 11.59 min.

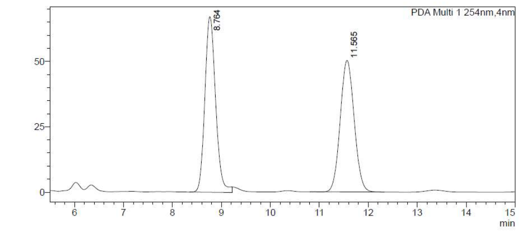

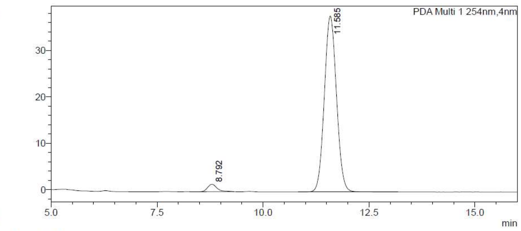

<Peak Table>
PDA Ch1 254nm

Peak#	Ret. Time	Area	Height	Area/Height	Height%	Area%
1	8.764	1025531	67071	15.290	57.168	50.749
2	11.565	995275	50253	19.805	42.832	49.251
Total		2020805	117323		100.000	100.000

<Peak Table>
PDA Ch1 254nm

Peak#	Ret. Time	Area	Height	Area/Height	Height%	Area%
1	8.792	24501	1588	15.426	4.022	3.146
2	11.585	754384	37906	19.901	95.978	96.854
Total		778885	39494		100.000	100.000

Ethyl (*R*)-4-(4-cyanophenyl)-4-oxo-2-(6-oxopyridazin-1(*6H*)-yl)butanoate

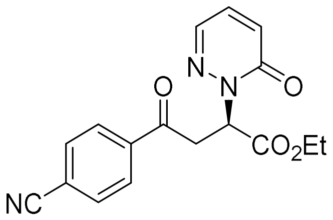

(-)-5ma; isolated yield: 28.3 mg (87%); colorless sticky oil; $[\alpha]_D^{20}$ = -19.1 (*c* = 1.0, $CHCl_3$); Enantiomeric excess: 87%, determined by HPLC (Chiralpak AD-H, hexane/*i*-PrOH = 70/30; flow rate 1.0 ml/min; 25 °C; 254 nm), first peak: t_R = 15.97 min, second peak: t_R = 18.99 min.

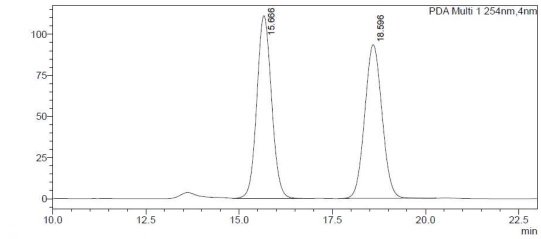

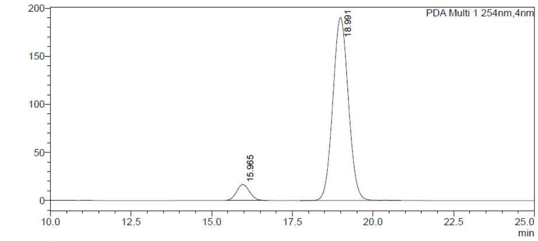

<Peak Table>
PDA Ch1 254nm

Peak#	Ret. Time	Area	Height	Area/Height	Height%	Area%
1	15.666	3043467	111032	27.411	54.301	50.246
2	18.596	3013651	93443	32.251	45.699	49.754
Total		6057118	204475		100.000	100.000

<Peak Table>
PDA Ch1 254nm

Peak#	Ret. Time	Area	Height	Area/Height	Height%	Area%
1	15.965	453028	16424	27.583	7.951	6.633
2	18.991	6376801	190145	33.536	92.049	93.367
Total		6829828	206569		100.000	100.000

Ethyl (*R*)-4-(3,4-dichlorophenyl)-4-oxo-2-(6-oxopyridazin-1(*6H*)-yl)-butanoate

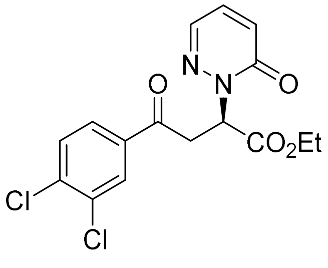

(-)-5na; isolated yield: 35.1 mg (95%); colorless sticky oil; $[\alpha]_D^{20}$ = -23.1 (*c* = 1.0,

$CHCl_3$); Enantiomeric excess: 93%, determined by HPLC (Chiralpak OD-H to OD-H, hexane/*i*-PrOH = 60/40; flow rate 0.5 ml/min; 25 °C; 254 nm), first peak: t_R = 68.16 min, second peak: t_R = 73.29 min.

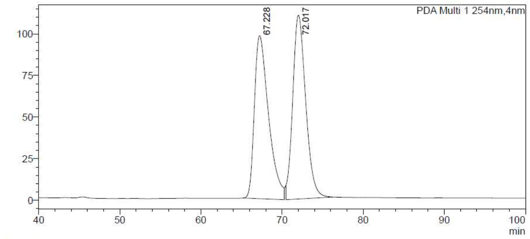

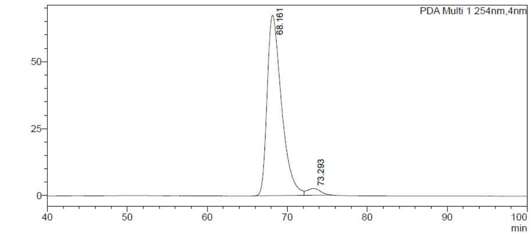

<Peak Table>
PDA Ch1 254nm

Peak#	Ret. Time	Area	Height	Area/Height	Height%	Area%
1	67.228	11868215	98011	121.091	47.006	49.135
2	72.017	12286237	110496	111.192	52.994	50.865
Total		24154452	208507		100.000	100.000

<Peak Table>
PDA Ch1 254nm

Peak#	Ret. Time	Area	Height	Area/Height	Height%	Area%
1	68.161	8425920	67380	125.051	96.364	96.539
2	73.293	302034	2543	118.790	3.636	3.461
Total		8727954	69922		100.000	100.000

Ethyl (*R*)-4-(benzo[b]thiophen-2-yl)-4-oxo-2-(6-oxopyridazin-1(*6H*)-yl)-butanoate

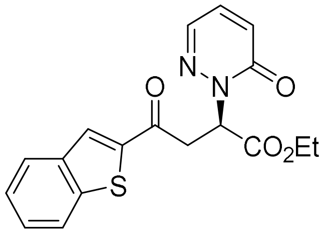

(-)-5oa; isolated yield: 33.1 mg (93%); colorless sticky oil; $[\alpha]_D^{20}$ = -55.8 (*c* = 1.0, $CHCl_3$); Enantiomeric excess: 95%, determined by HPLC (Chiralpak AD-H, hexane/*i*-PrOH = 70/30; flow rate 1.0 ml/min; 25 °C; 254 nm), first peak: t_R = 16.57 min, second peak: t_R = 18.92 min.

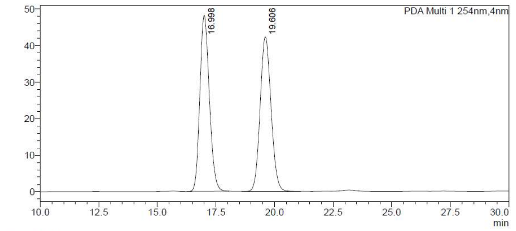

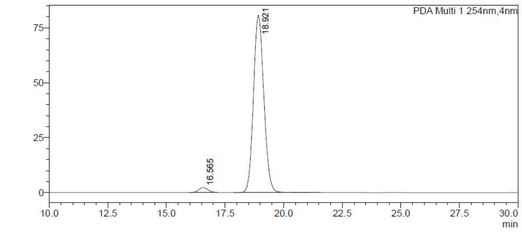

<Peak Table>
PDA Ch1 254nm

Peak#	Ret. Time	Area	Height	Area/Height	Height%	Area%
1	16.998	1358995	48155	28.221	53.232	49.835
2	19.606	1368010	42307	32.335	46.768	50.165
Total		2727005	90462		100.000	100.000

<Peak Table>
PDA Ch1 254nm

Peak#	Ret. Time	Area	Height	Area/Height	Height%	Area%
1	16.565	58316	2219	26.280	2.678	2.300
2	18.921	2477646	80644	30.723	97.322	97.700
Total		2535961	82863		100.000	100.000

Ethyl (*R*)-4-(naphthalen-2-yl)-4-oxo-2-(6-oxopyridazin-1(*6H*)-yl)butanoate

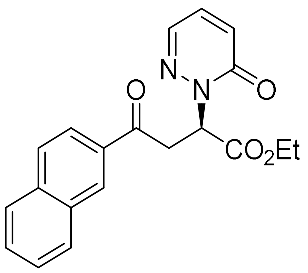

(-)-5pa; isolated yield: 31.2 mg (89%); colorless sticky oil; $[\alpha]_D^{20}$ = -77.8 (*c* = 1.0, $CHCl_3$); Enantiomeric excess: 96%, determined by HPLC (Chiralpak AD-H to AD-H, hexane/*i*-PrOH = 60/40; flow rate 0.5 ml/min; 25 °C; 254 nm), first peak: t_R = 41.05 min, second peak: t_R = 42.63 min.

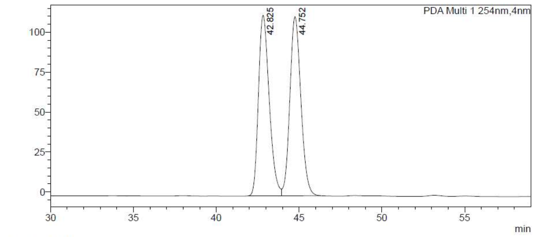

<Peak Table>
PDA Ch1 254nm

Peak#	Ret. Time	Area	Height	Area/Height	Height%	Area%
1	42.825	5132839	113101	45.383	50.218	49.691
2	44.752	5196585	112121	46.348	49.782	50.309
Total		10329424	225222		100.000	100.000

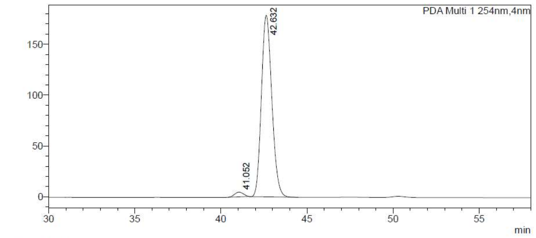

<Peak Table>
PDA Ch1 254nm

Peak#	Ret. Time	Area	Height	Area/Height	Height%	Area%
1	41.052	162069	4632	34.986	2.529	2.103
2	42.632	7544344	178568	42.249	97.471	97.897
Total		7706413	183201		100.000	100.000

(*R*)-4-oxo-2-(6-oxopyridazin-1(*6H*)-yl)-4-phenylbutanoic acid

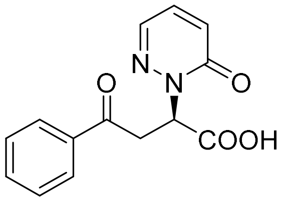

(-)-6a; white solid; The enantiomeric excess of **6a** as determined by chiral HPLC analysis on Chiralpak AD-H after esterification with $TMSCH_2N_2$, (hexanes:2-propanol = 70:30, flow rate 1.0 mL/min; 25 °C; 254 nm); minor enantiomer t_R =12.15 min, major enantiomer t_R = 14.19 min; $[\alpha]_D^{20}$ = +2.8 (*c* = 1.0, $CHCl_3$); ^{1}H NMR (500 MHz, MeOD) δ 8.00 (d, *J* = 7.6 Hz, 2H), 7.87 (d, *J* = 2.6 Hz, 1H), 7.62 (t, *J* = 7.4 Hz, 1H), 7.50 (t, *J* = 7.6 Hz, 2H), 7.40 (dd, *J* = 9.4, 3.7 Hz, 1H), 7.02 (d, *J* = 9.2 Hz, 1H), 6.09 (dd, *J* = 8.2, 5.4 Hz, 1H), 4.01-3.90 (m, 2H); ^{13}C NMR (126 MHz, MeOD) δ 196.38, 137.16, 136.32, 133.29, 132.50, 129.00, 128.47, 128.43, 127.89, 127.84, 58.15, 48.19, 48.02, 37.80; HRMS (ESI) m/z calcd. for $C_{14}H_{12}N_2NaO_4$ $[M+Na]^+$ = 295.0689, found 295.0685.

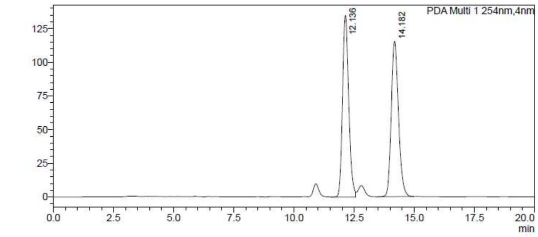

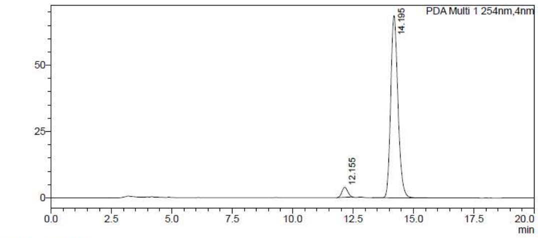

<Peak Table>
PDA Ch1 254nm

Peak#	Ret. Time	Area	Height	Area/Height	Height%	Area%
1	12.136	2416541	134955	17.906	53.934	50.034
2	14.182	2413235	115267	20.936	46.066	49.966
Total		4829776	250222		100.000	100.000

<Peak Table>
PDA Ch1 254nm

Peak#	Ret. Time	Area	Height	Area/Height	Height%	Area%
1	12.155	62438	3768	16.572	5.201	4.175
2	14.195	1433052	68679	20.866	94.799	95.825
Total		1495489	72447		100.000	100.000

S-(*p*-tolyl) (*R*)-4-oxo-2-(6-oxopyridazin-1(*6H*)-yl)-4-phenylbutanethioate

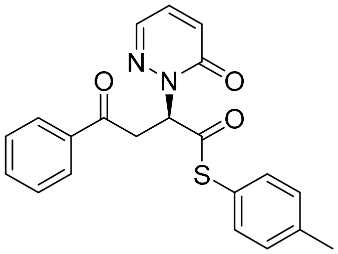

7a, red solid; $[\alpha]_D^{20}$ = +125.9 (*c* = 1.0, $CHCl_3$); ^{1}H NMR (500 MHz, $CDCl_3$) δ 7.97-7.95 (m, 2H), 7.79 (dd, *J* = 3.7, 1.6 Hz, 1H), 7.57 (dd, *J* = 10.5, 4.3 Hz, 1H), 7.45 (t, *J* = 7.7 Hz, 2H), 7.29 (d, *J* = 8.1 Hz, 2H), 7.22-7.19 (m, 3H), 7.01 (dd, *J* = 9.5, 1.7 Hz, 1H), 6.41 (dd, *J* = 8.2, 5.4 Hz, 1H), 4.00-3.90 (m, 2H), 2.35 (s, 3H); ^{13}C NMR (126 MHz, $CDCl_3$) δ 195.63, 195.25, 160.35, 140.06, 136.71, 136.16, 134.69, 133.55, 131.53, 130.23, 130.14, 128.70, 128.21, 122.76, 64.09, 38.51, 21.35; Enantiomeric excess: 90%, determined by HPLC (Chiralpak AD-H, hexane/*i*-PrOH = 70/30; flow rate 1.0 ml/min; 25 °C; 254 nm), first peak: t_R = 21.61 min, second peak: t_R = 22.84 min; HRMS (ESI) m/z calcd. for $C_{21}H_{18}N_2NaO_3S$ $[M+Na]^+$ = 401.0930, found 401.0929.

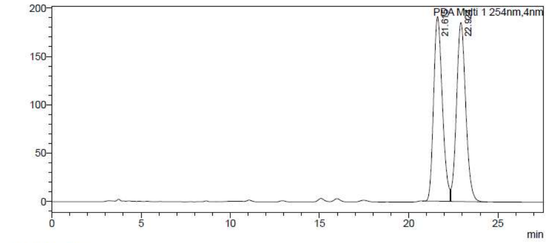

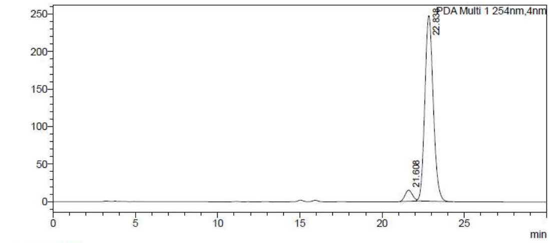

<Peak Table>
PDA Ch1 254nm

Peak#	Ret. Time	Area	Height	Area/Height	Height%	Area%
1	21.615	6493722	190622	34.066	50.741	49.458
2	22.924	6636123	185056	35.860	49.259	50.542
Total		13129845	375678		100.000	100.000

<Peak Table>
PDA Ch1 254nm

Peak#	Ret. Time	Area	Height	Area/Height	Height%	Area%
1	21.608	459796	14752	31.169	5.629	4.965
2	22.838	8800482	247304	35.586	94.371	95.035
Total		9260278	262056		100.000	100.000

(*R*)-N-(1-methyl-*1H*-pyrazol-3-yl)-4-oxo-2-(6-oxopyridazin-1(*6H*)-yl)-4-

Phenylbutanamide

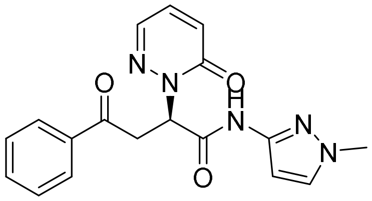

7b, yellow solid; $[\alpha]_D^{20}$ = +49.0 (*c* = 1.0, $CHCl_3$); ^1H NMR (400 MHz, $CDCl_3$) δ 9.53 (s, 1H), 8.00 (d, *J* = 7.6 Hz, 2H), 7.78 (d, *J* = 2.5 Hz, 1H), 7.55 (d, *J* = 7.3 Hz, 1H), 7.45 (t, *J* = 7.6 Hz, 2H), 7.18-7.15 (m, 2H), 7.00-6.97 (m, 1H), 6.58 (d, *J* = 1.7 Hz, 1H), 6.30 (t, *J* = 6.8 Hz, 1H), 3.99-3.97 (m, 2H), 3.77 (s, 3H); ^{13}C NMR (101 MHz, $CDCl_3$) δ 196.22, 165.83, 160.96, 146.44, 136.87, 136.26, 133.48, 131.39, 130.76, 129.83, 128.65, 128.22, 125.73, 97.61, 57.95, 38.17; Enantiomeric excess: 92%, determined by HPLC (Chiralpak AS-H, hexane/*i*-PrOH = 50/50; flow rate 1.0 ml/min; 25 °C; 254 nm), first peak: t_R = 22.650 min, second peak: t_R = 45.612 min; HRMS (ESI) m/z calcd. for $C_{18}H_{17}N_5NaO_3$ $[M+Na]^+$ = 374.1224, found 374.1220.

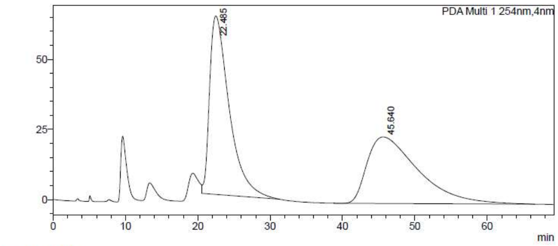

<Peak Table>
PDA Ch1 254nm

Peak#	Ret. Time	Area	Height	Area/Height	Height%	Area%
1	22.485	12016442	63636	188.830	72.855	50.767
2	45.640	11653194	23711	491.474	27.145	49.233
Total		23669636	87347		100.000	100.000

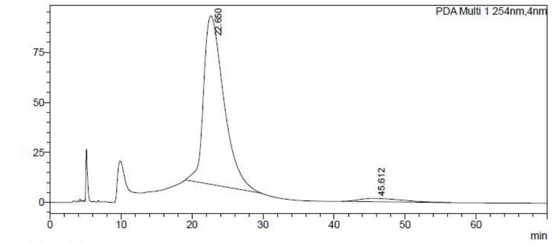

<Peak Table>
PDA Ch1 254nm

Peak#	Ret. Time	Area	Height	Area/Height	Height%	Area%
1	22.650	16765958	84182	199.163	98.088	96.104
2	45.612	679631	1641	414.105	1.912	3.896
Total		17445589	85823		100.000	100.000

6'-phenyl-4',5'-dihydro-*6H*-[1,4'-bipyridazine]-3',6(*2'H*)-dione

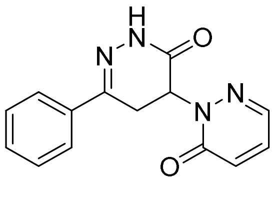

7c, white solid; ^1H NMR (500 MHz, $CDCl_3$) δ 8.77 (s, 1H), 7.87 (d, *J* = 2.2 Hz, 1H), 7.72-7.70 (m, 2H), 7.43-7.41 (m, 2H), 7.23 (dd, *J* = 9.5, 3.7 Hz, 1H), 7.00 (d, *J* = 9.3 Hz, 1H), 5.96 (dd, *J* = 13.4, 7.4 Hz, 1H), 3.60 (dd, *J* = 16.4, 13.6 Hz, 1H), 3.39 (dd, *J* = 16.6, 7.4 Hz, 1H); ^{13}C NMR (126 MHz, $CDCl_3$) δ 163.41, 160.35, 150.44, 137.26, 135.09, 131.53, 130.22, 129.98, 128.74, 125.89, 53.02, 28.28; HRMS (ESI) m/z calcd. for $C_{14}H_{12}N_4NaO_2$ $[M+Na]^+$ = 291.0852, found 291.0849.

2-(2-oxo-5-phenyl-2,3-dihydrofuran-3-yl)pyridazin-3(*2H*)-one

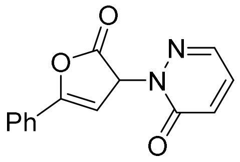

7d, white solid; ^{1}H NMR (500 MHz, $CDCl_3$) δ 7.80 (dd, J = 3.8, 1.6 Hz, 1H), 7.68-7.67 (m, 2H), 7.44-7.43 (m, 3H), 7.23 (dd, J = 9.5, 3.8 Hz, 1H), 6.98 (dd, J = 9.5, 1.6 Hz, 1H), 6.29 (d, J = 2.6 Hz, 1H), 5.88 (d, J = 2.7 Hz, 1H); ^{13}C NMR (126 MHz, $CDCl_3$) δ 171.06, 159.52, 156.55, 137.40, 131.96, 130.62, 130.14, 128.78, 127.48, 125.42, 98.03, 62.57; HRMS (ESI) m/z calcd. for $C_{14}H_{10}N_2NaO_3$ $[M+Na]^+$ = 277.0584, found 277.0584.

X-Ray Crystallographic Analysis

Determination of the Absolute Configurations of the Product (+)-3da

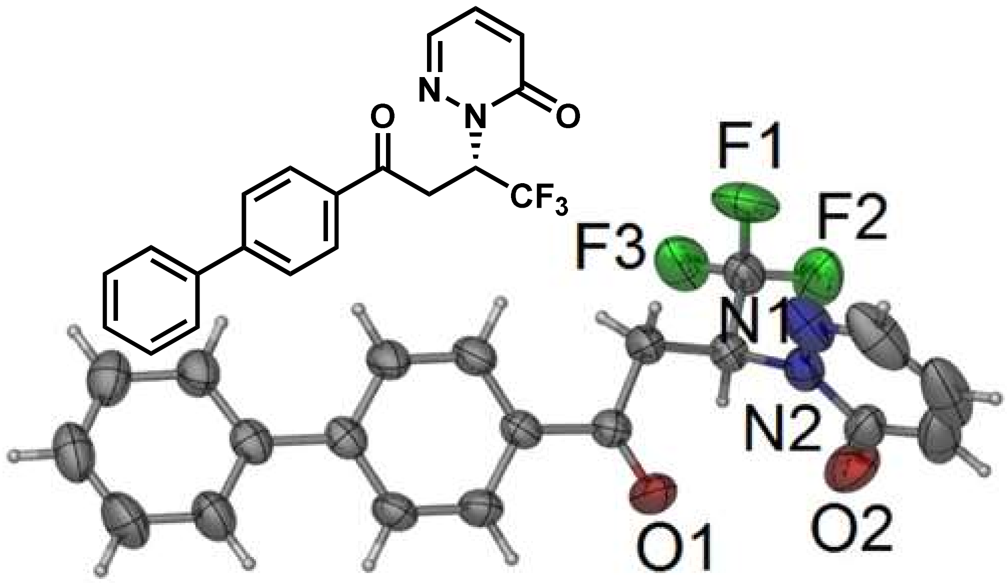

Figure S4. X ray structure of (+)-3da (CCDC 1839409). Related to Scheme 4.

Data S2. **Spectra of Products. Realated to Scheme 3, Scheme 4, Scheme 5, Scheme 6 and Scheme 7.**

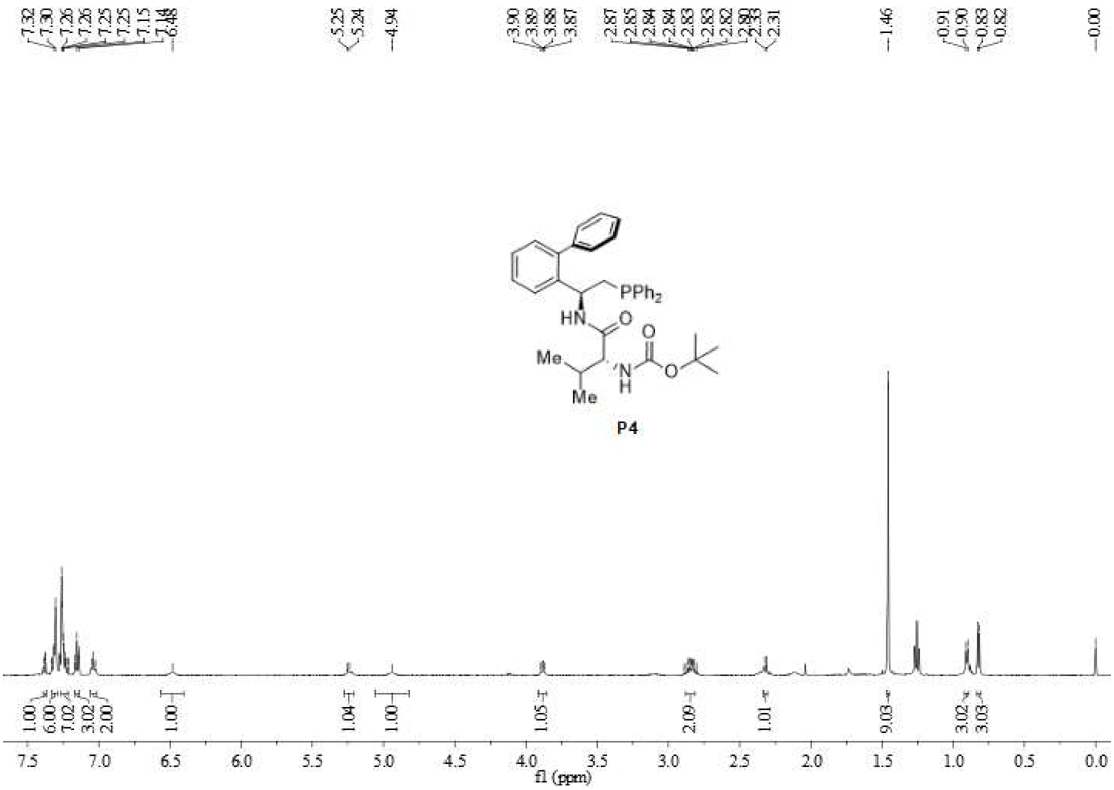

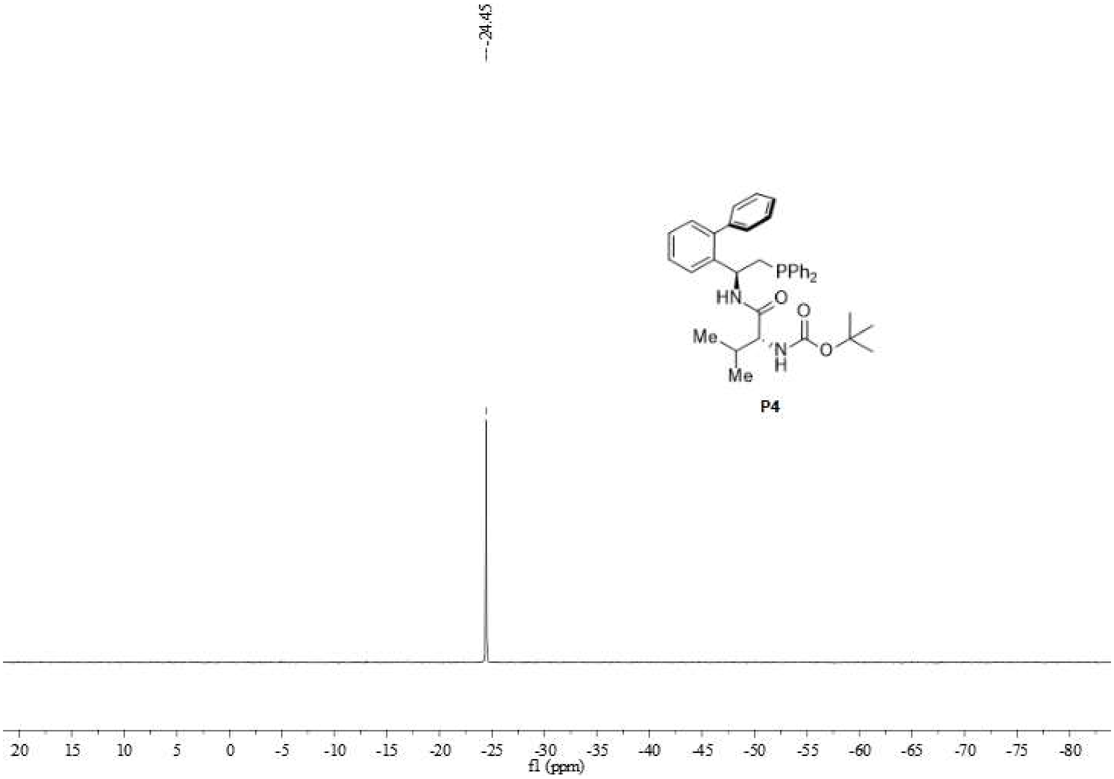

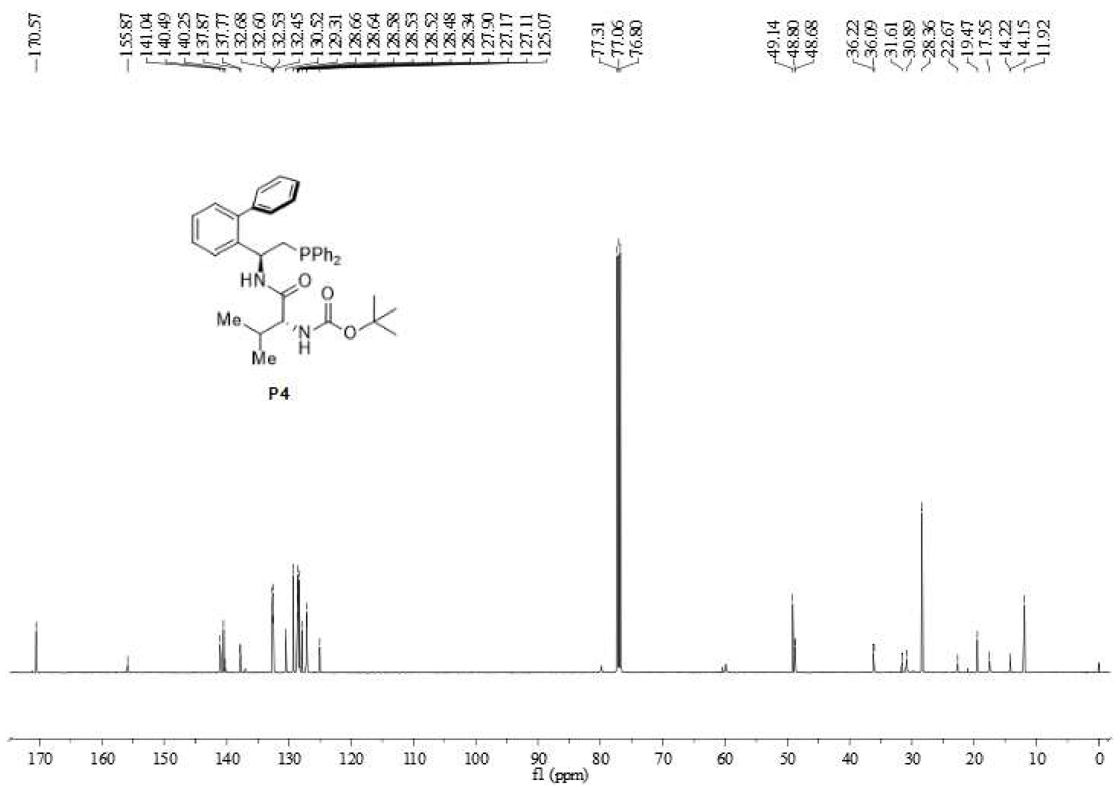
170.57
155.87
141.04
140.49
140.25
137.87
137.77
132.68
132.60
132.53
132.45
130.52
129.31
128.66
128.64
128.58
128.53
128.52
128.48
128.34
127.90
127.17
127.11
125.07
77.31
77.06
76.80
49.14
48.80
48.68
36.22
36.09
31.61
30.89
28.36
22.67
19.47
17.55
14.22
14.15
11.92
PPh2
HN
O
O
Me
N
H
O
Me
P4
170 160 150 140 130 120 110 100 90 80 70 60 50 40 30 20 10 0
f1 (ppm)

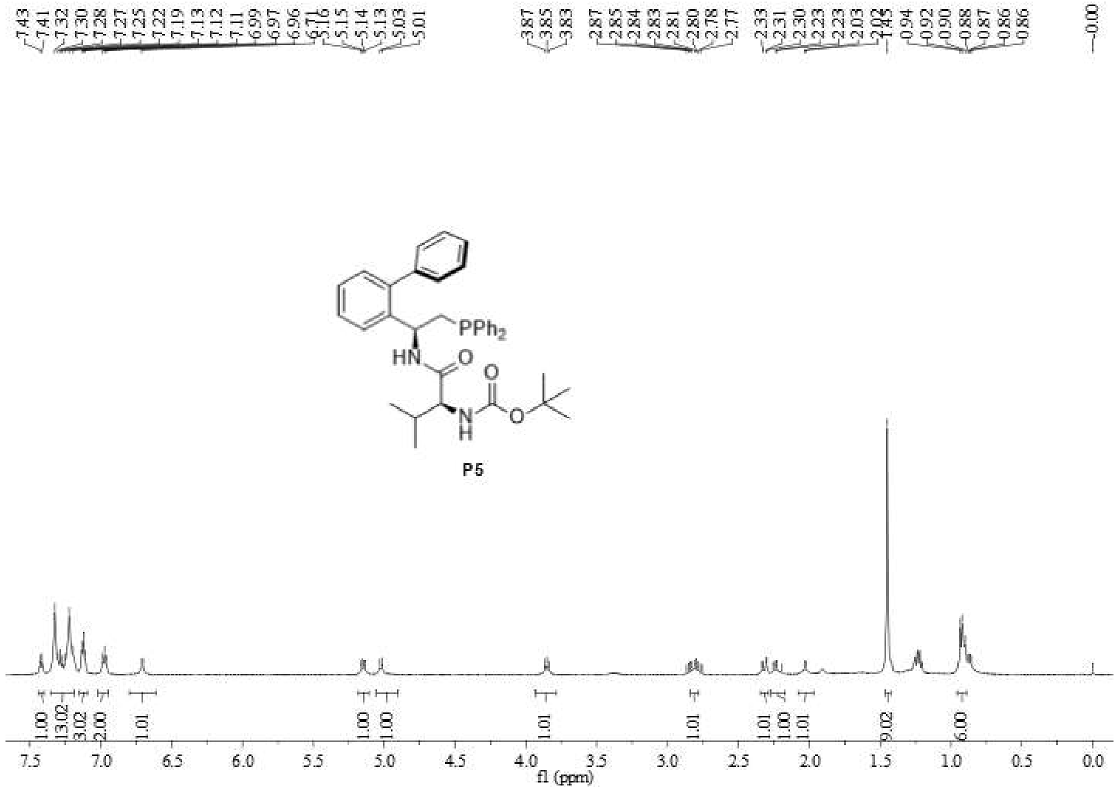
7.43
7.41
7.32
7.30
7.28
7.27
7.25
7.22
7.19
7.13
7.12
7.11
6.99
6.97
6.96
6.71
5.16
5.15
5.14
5.13
5.03
5.01
3.87
3.85
3.83
2.87
2.85
2.84
2.83
2.81
2.80
2.78
2.77
2.33
2.31
2.30
2.23
2.23
2.03
2.02
1.45
0.94
0.92
0.90
0.88
0.87
0.86
0.86
-0.00
PPh2
HN
O
O
N
H
O
P5
1.00
13.02
3.02
2.00
1.01
1.00
1.00
1.01
1.01
1.01
1.00
1.01
9.02
6.00
7.5 7.0 6.5 6.0 5.5 5.0 4.5 4.0 3.5 3.0 2.5 2.0 1.5 1.0 0.5 0.0
f1 (ppm)

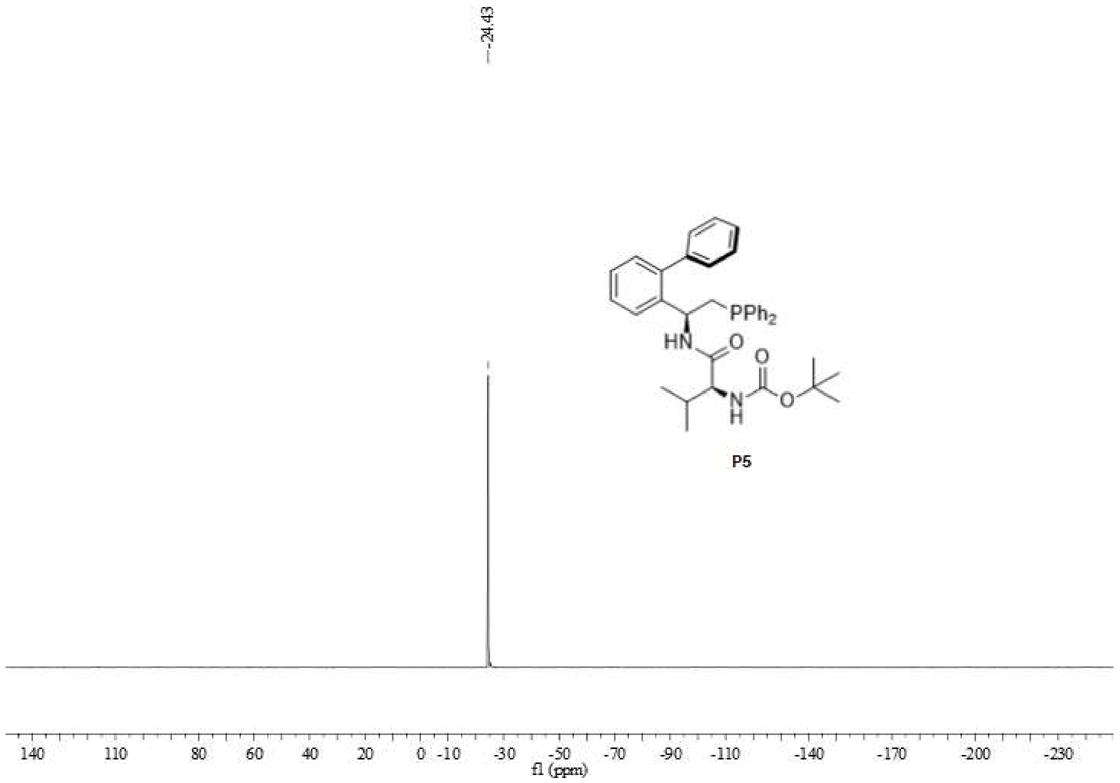
-24.43
PPh2
HN
O
O
N
H
O
P5
140
110
80
60
40
20
0
-10
-30
-50
-70
-90
-110
-140
-170
-200
-230
f1 (ppm)

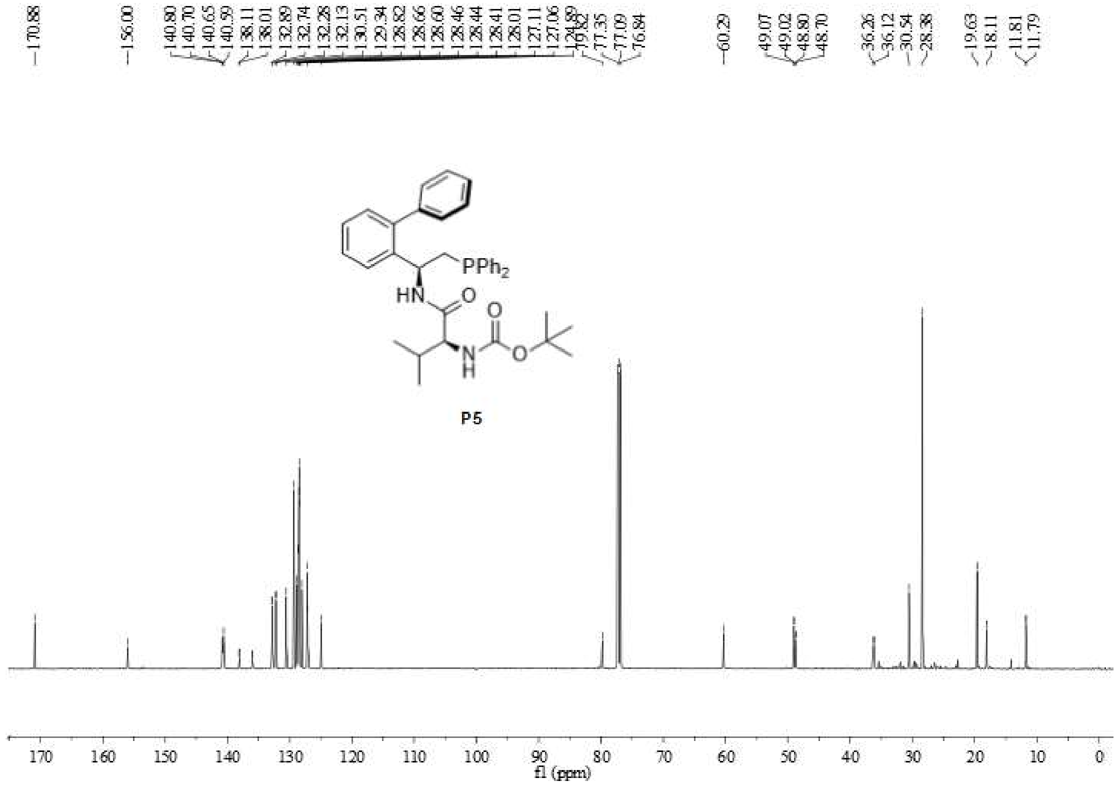
170.88
156.00
140.80
140.70
140.65
140.59
138.11
138.01
132.89
132.74
132.28
132.13
130.51
129.34
128.82
128.66
128.60
128.46
128.44
128.41
128.01
127.11
127.06
124.89
79.82
77.35
77.09
76.84
60.29
49.07
49.02
48.80
48.70
36.26
36.12
30.54
28.38
19.63
18.11
11.81
11.79
PPh2
HN
O
O
N
H
O
P5
170
160
150
140
130
120
110
100
90
80
70
60
50
40
30
20
10
0
f1 (ppm)

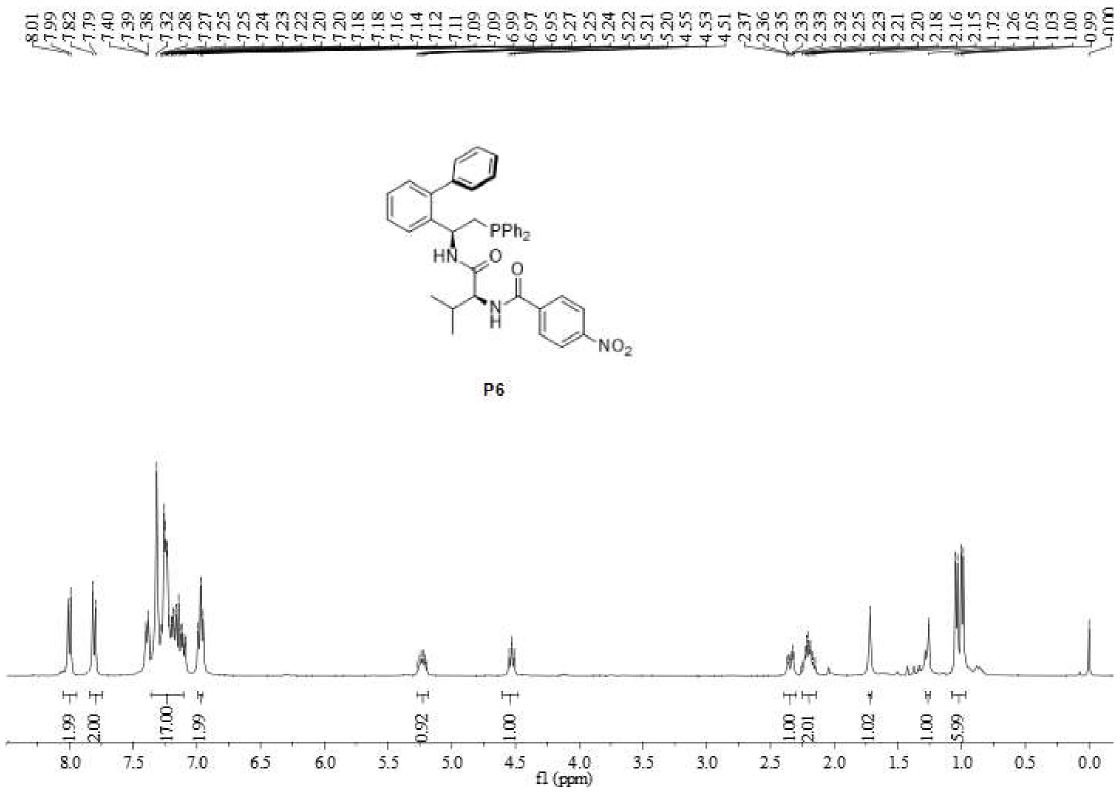
8.01 7.99 7.82 7.79 7.40 7.39 7.38 7.32 7.28 7.27 7.25 7.25 7.24 7.23 7.22 7.20 7.20 7.18 7.16 7.14 7.12 7.11 7.09 7.09 6.99 6.97 6.95 5.27 5.25 5.24 5.22 5.21 5.20 4.55 4.53 4.51 2.37 2.36 2.35 2.33 2.33 2.33 2.32 2.25 2.23 2.21 2.20 2.18 2.16 2.15 1.72 1.26 1.05 1.03 1.00 0.99 0.00
PPh2
HN
O
H
N
NO2
P6
1.99
2.00
17.00
1.99
0.92
1.00
1.00
2.01
1.02
1.00
5.99
f1 (ppm)

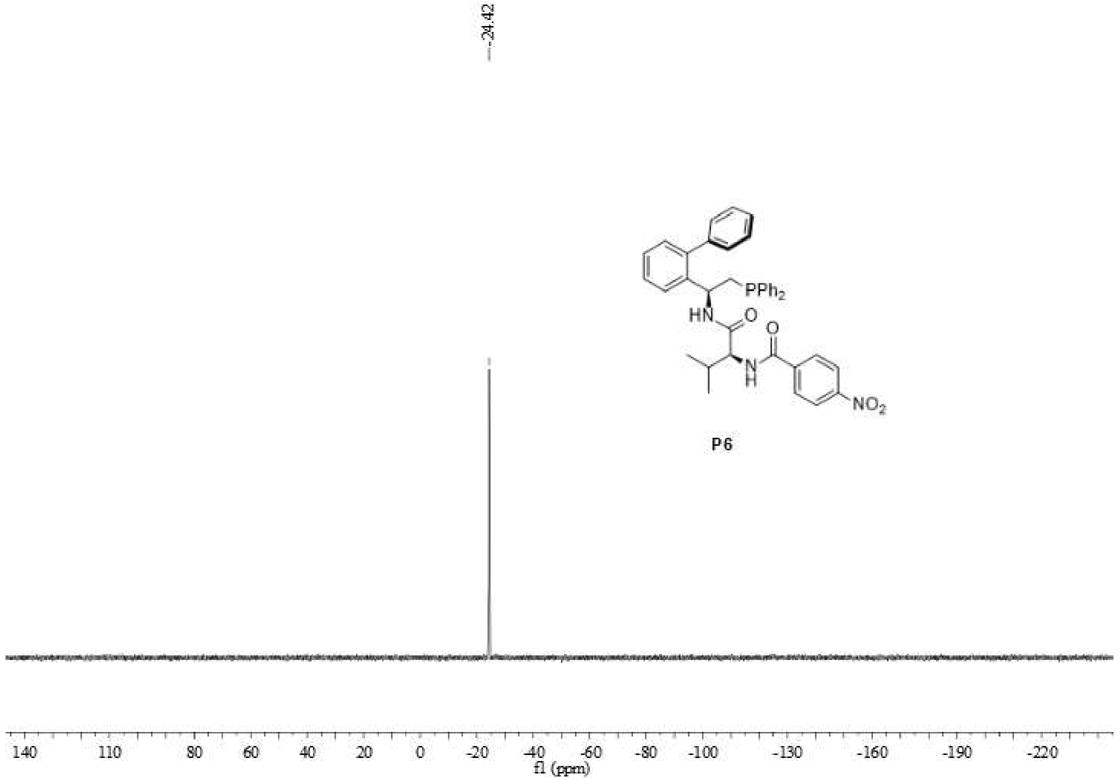
-24.42
PPh2
HN
O
H
N
NO2
P6
f1 (ppm)

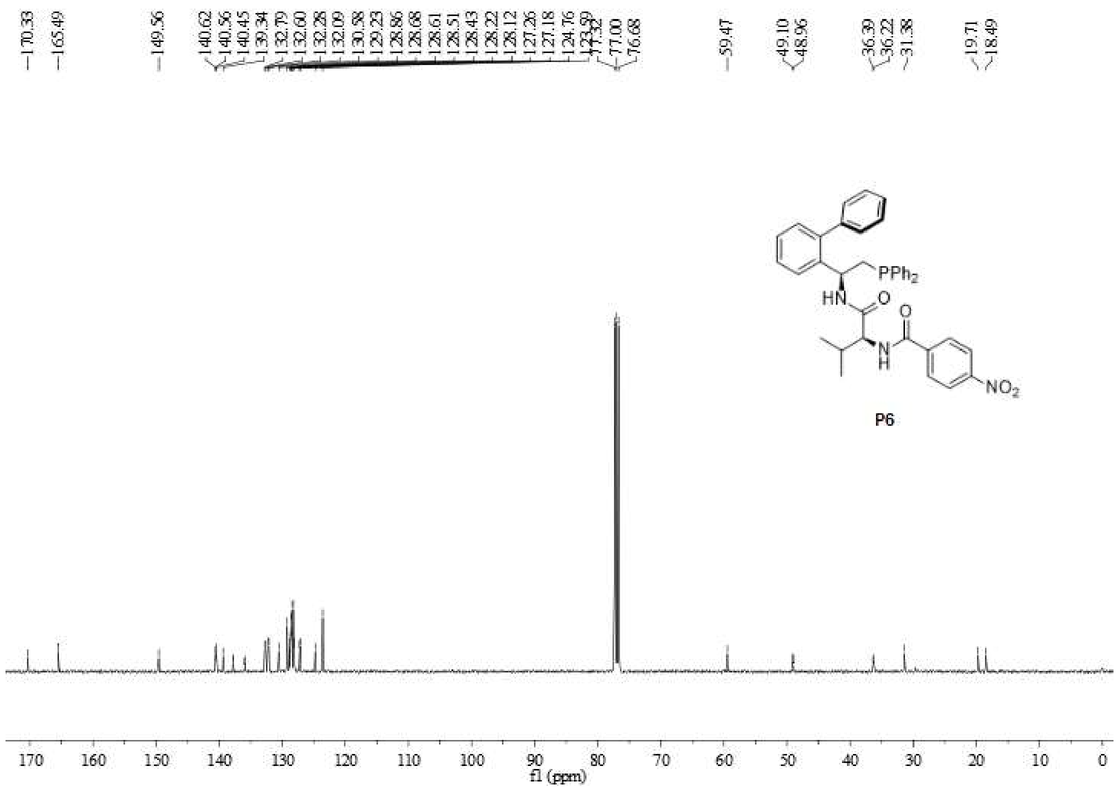
170.33
165.49
149.56
140.62
140.56
140.45
139.34
132.79
132.60
132.28
132.09
130.58
129.23
128.86
128.68
128.61
128.51
128.43
128.22
128.12
127.26
127.18
124.76
123.59
77.32
77.00
76.68
59.47
49.10
48.96
36.39
36.22
31.38
19.71
18.49
PPh2
HN
O
O
N
H
NO2
P6
f1 (ppm)

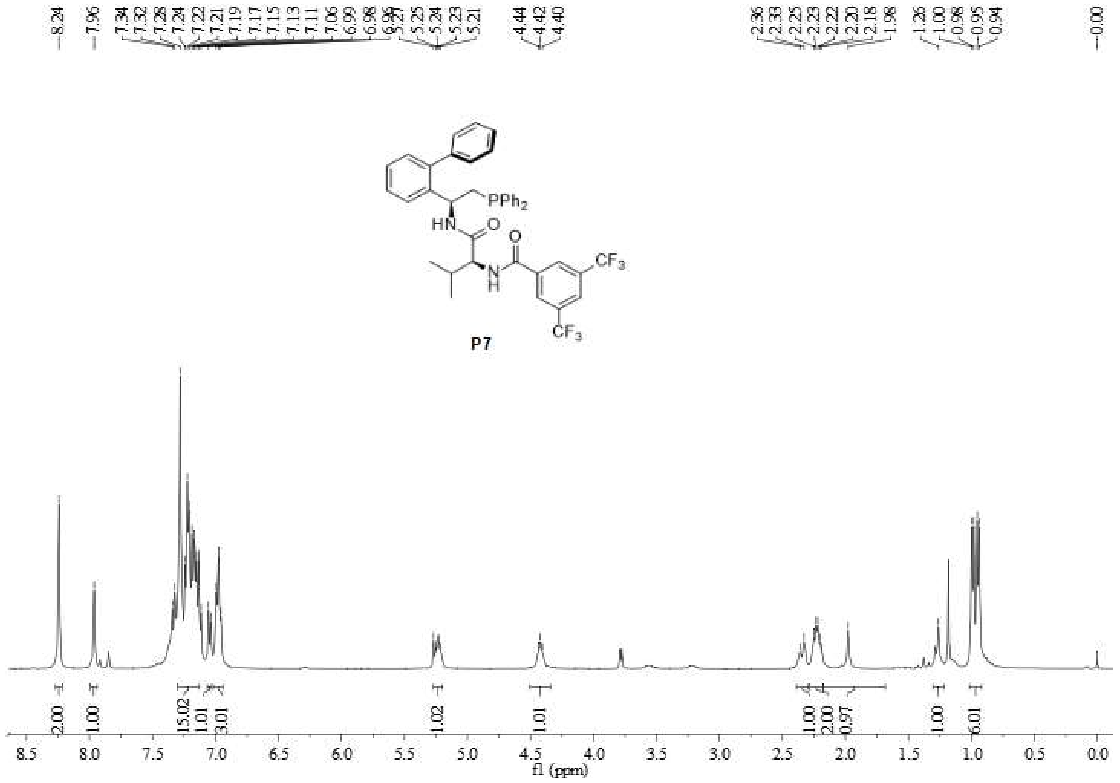
8.24
7.96
7.34
7.32
7.28
7.24
7.22
7.21
7.19
7.17
7.15
7.13
7.11
7.06
6.99
6.98
6.96
5.27
5.25
5.24
5.23
5.21
4.44
4.42
4.40
2.36
2.33
2.25
2.23
2.22
2.20
2.18
1.98
1.26
1.00
0.98
0.95
0.94
0.00
PPh2
HN
O
O
N
H
CF3
CF3
P7
2.00
1.00
15.02
1.01
3.01
1.02
1.01
1.00
2.00
0.97
1.00
6.01
f1 (ppm)

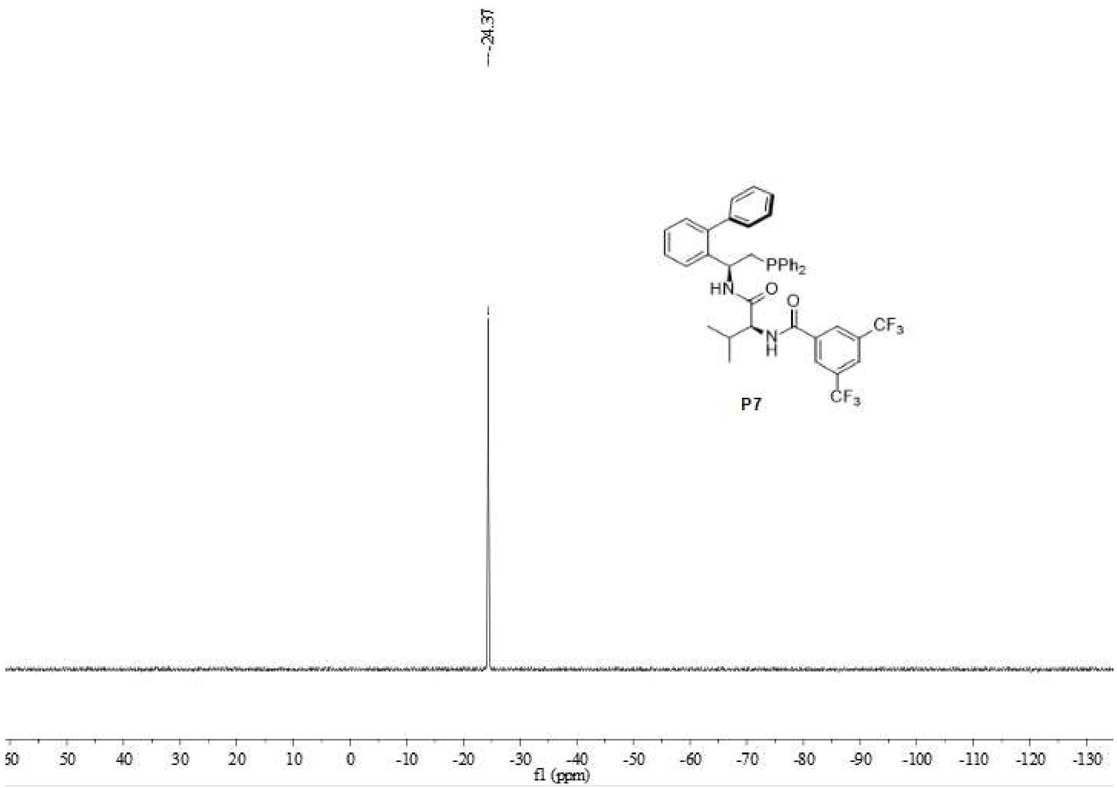

-24.37
PPh2
HN
O
N
H
CF3
CF3
P7
60 50 40 30 20 10 0 -10 -20 -30 -40 -50 -60 -70 -80 -90 -100 -110 -120 -130
f1 (ppm)

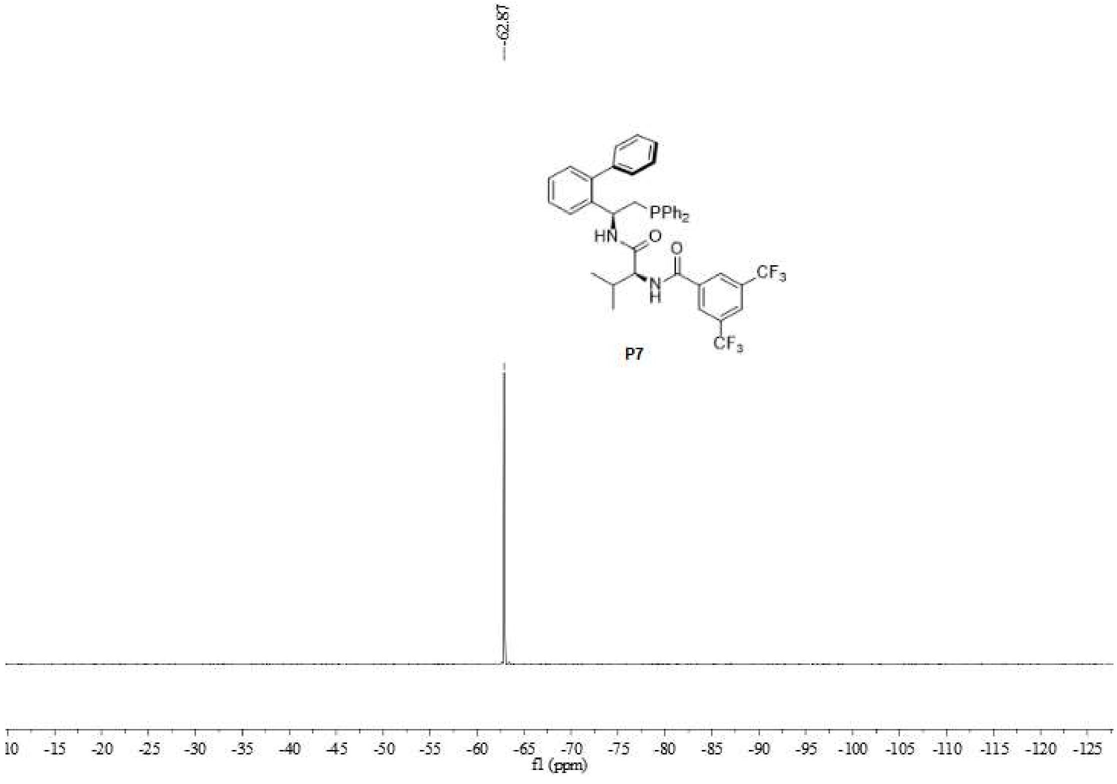

-62.87
PPh2
HN
O
N
H
CF3
CF3
P7
10 -15 -20 -25 -30 -35 -40 -45 -50 -55 -60 -65 -70 -75 -80 -85 -90 -95 -100 -105 -110 -115 -120 -125
f1 (ppm)

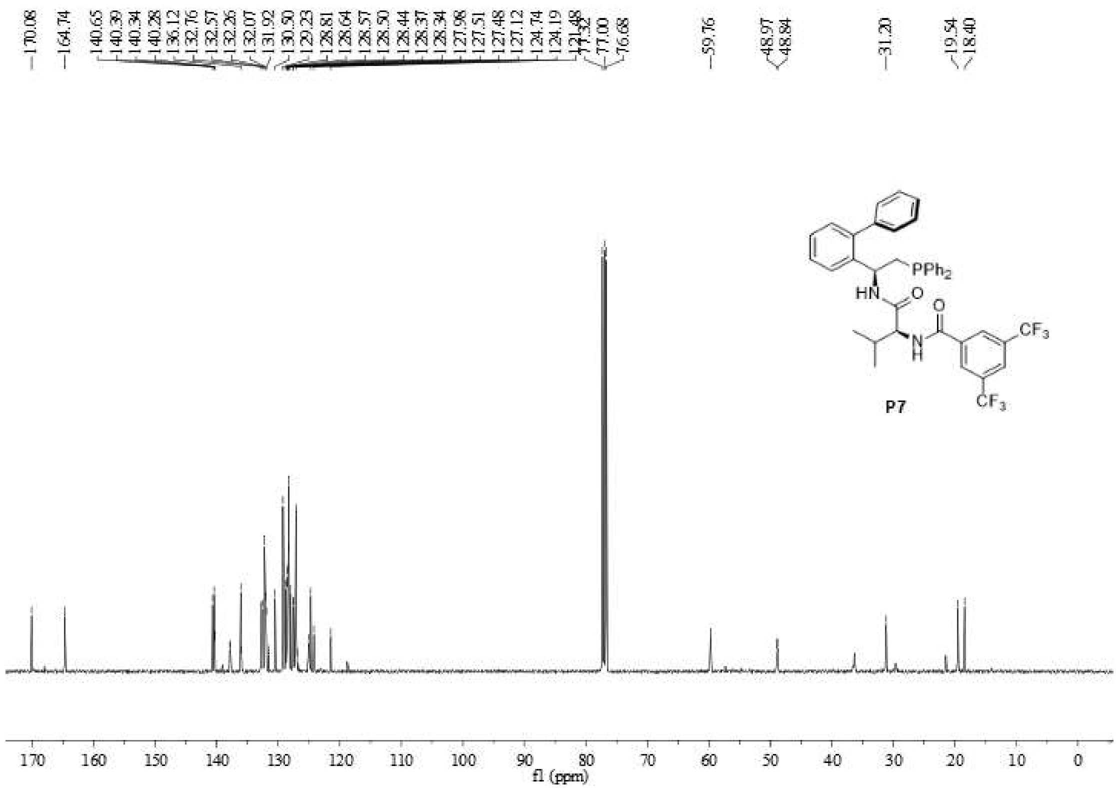
170.08
164.74
140.65
140.39
140.34
140.28
136.12
132.76
132.57
132.26
132.07
131.92
130.50
129.23
128.81
128.64
128.57
128.50
128.44
128.37
128.34
127.98
127.51
127.48
127.12
124.74
124.19
121.48
77.32
77.00
76.68
59.76
48.97
48.84
31.20
19.54
18.40
PPh2
HN
O
O
N
H
CF3
CF3
P7
170 160 150 140 130 120 110 100 90 80 70 60 50 40 30 20 10 0
f1 (ppm)

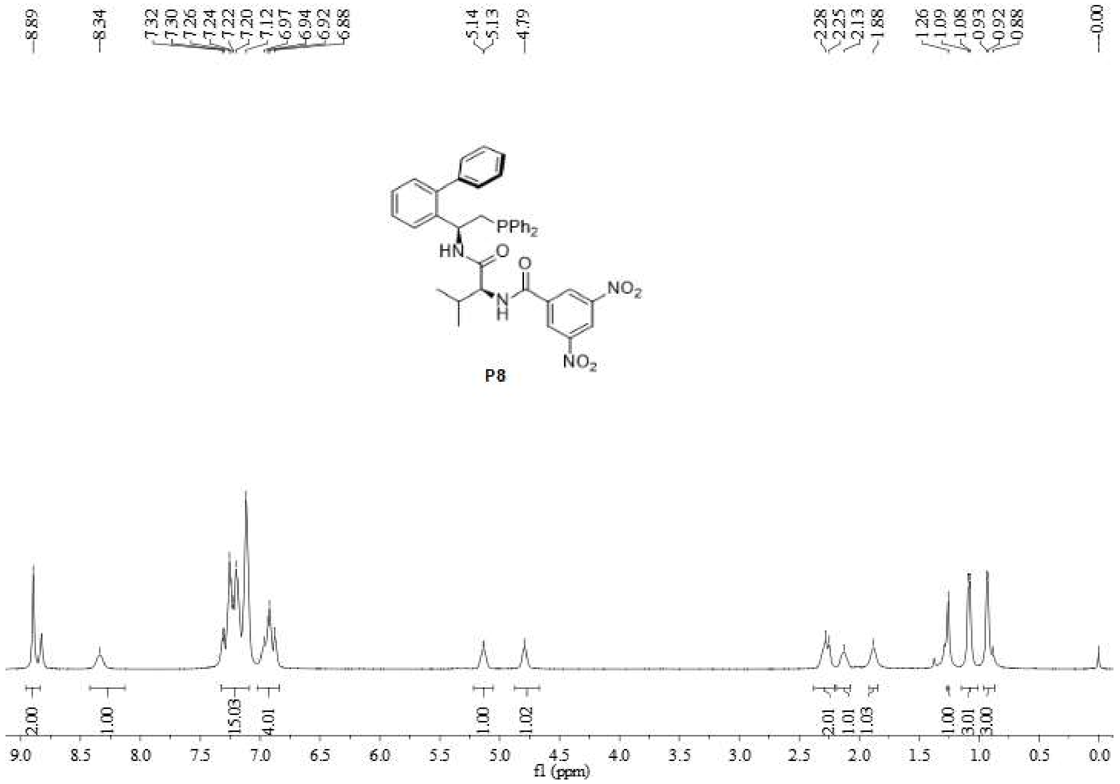
8.89
8.34
7.32
7.30
7.26
7.24
7.22
7.20
7.12
6.97
6.94
6.92
6.88
5.14
5.13
4.79
2.28
2.25
2.13
1.88
1.26
1.09
1.08
0.93
0.92
0.88
0.00
PPh2
HN
O
O
N
H
NO2
NO2
P8
2.00
1.00
15.03
4.01
1.00
1.02
2.01
1.01
1.03
1.00
3.01
3.00
9.0 8.5 8.0 7.5 7.0 6.5 6.0 5.5 5.0 4.5 4.0 3.5 3.0 2.5 2.0 1.5 1.0 0.5 0.0
f1 (ppm)

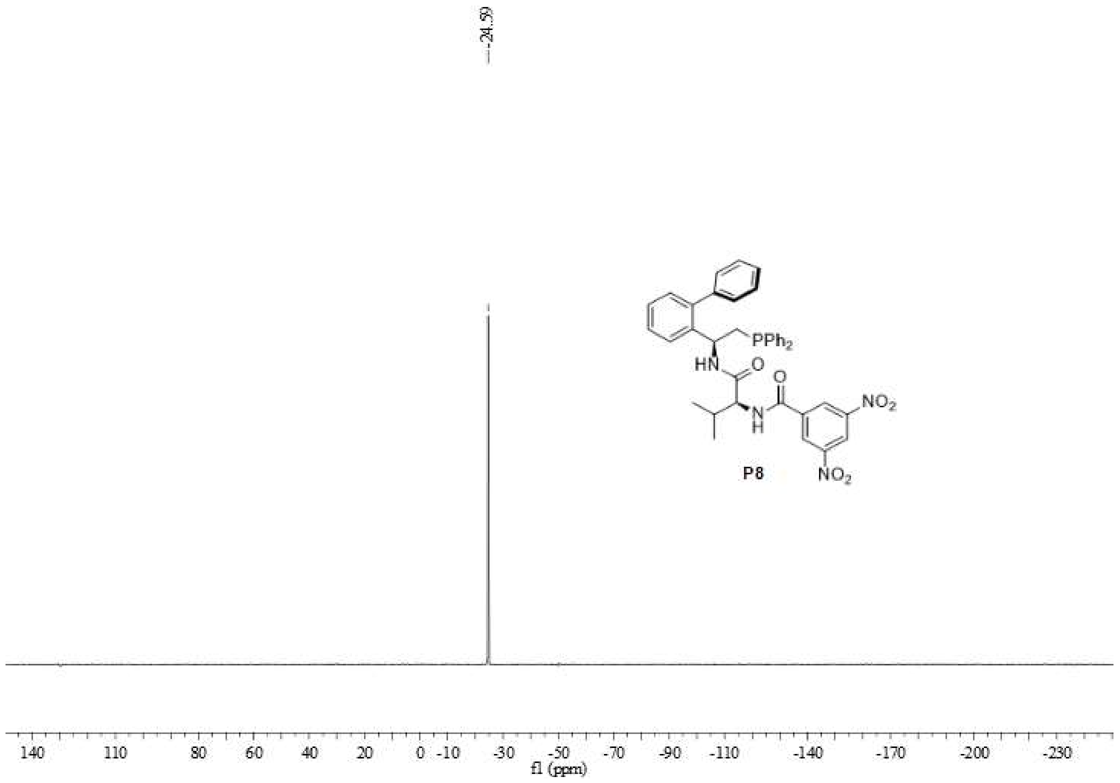

-24.59
PPh2
HN
O
NO2
N
H
NO2
P8
140 110 80 60 40 20 0 -10 -30 -50 -70 -90 -110 -140 -170 -200 -230
f1 (ppm)

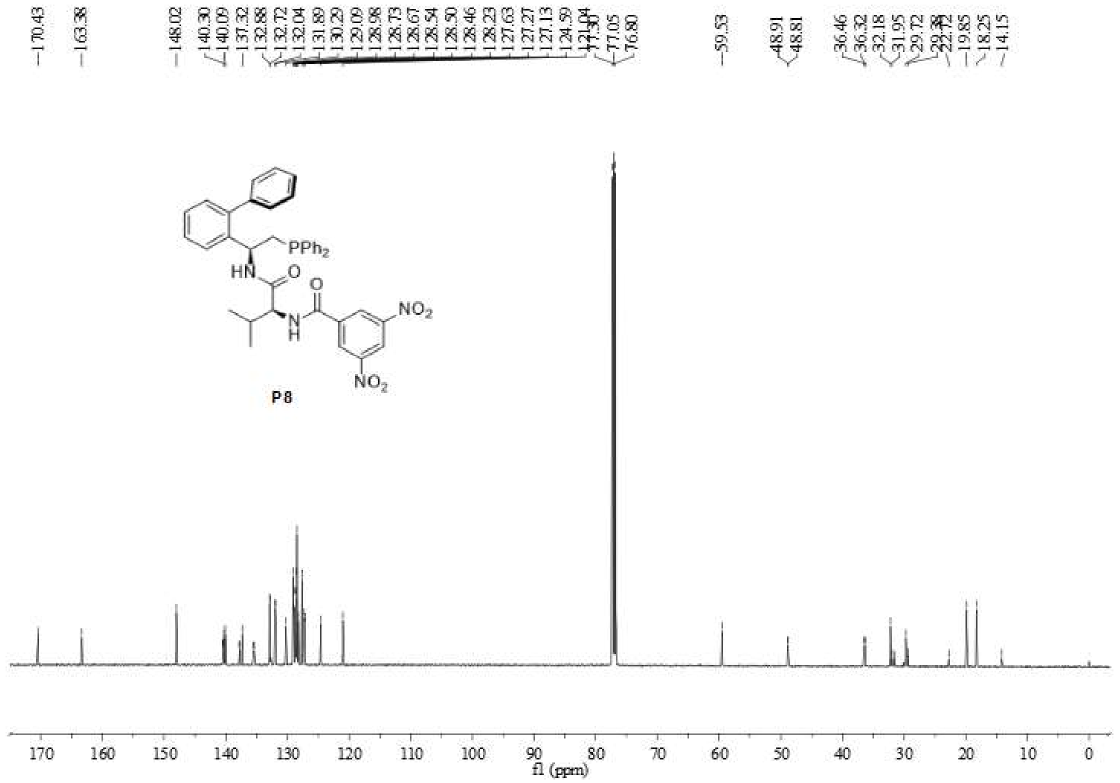

170.43
163.38
148.02
140.30
140.09
137.32
132.88
132.72
132.04
131.89
130.29
129.09
128.98
128.73
128.67
128.54
128.50
128.46
128.23
127.63
127.27
127.13
124.59
121.04
77.30
77.05
76.80
59.53
48.91
48.81
36.46
36.32
32.18
31.95
29.72
22.72
19.85
18.25
14.15
PPh2
HN
O
NO2
N
H
NO2
P8
170 160 150 140 130 120 110 100 90 80 70 60 50 40 30 20 10 0
f1 (ppm)

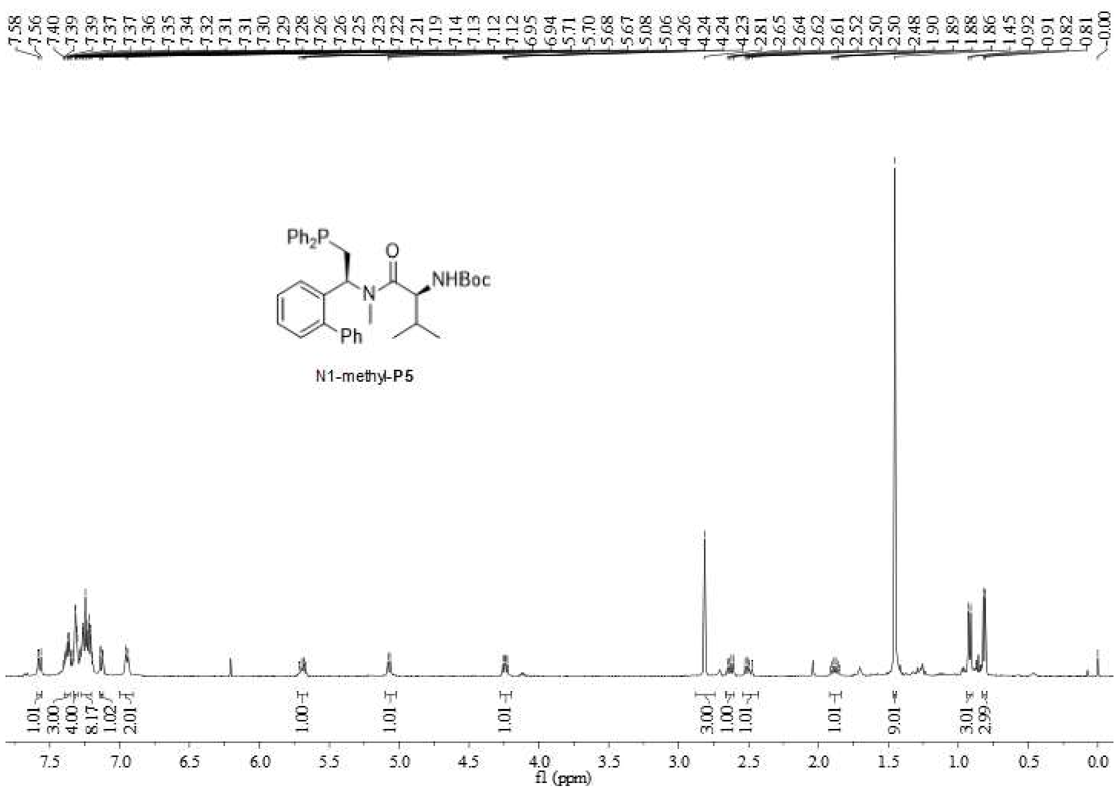
Ph2P
NHBoc
Ph
N1-methyl-P5
f1 (ppm)

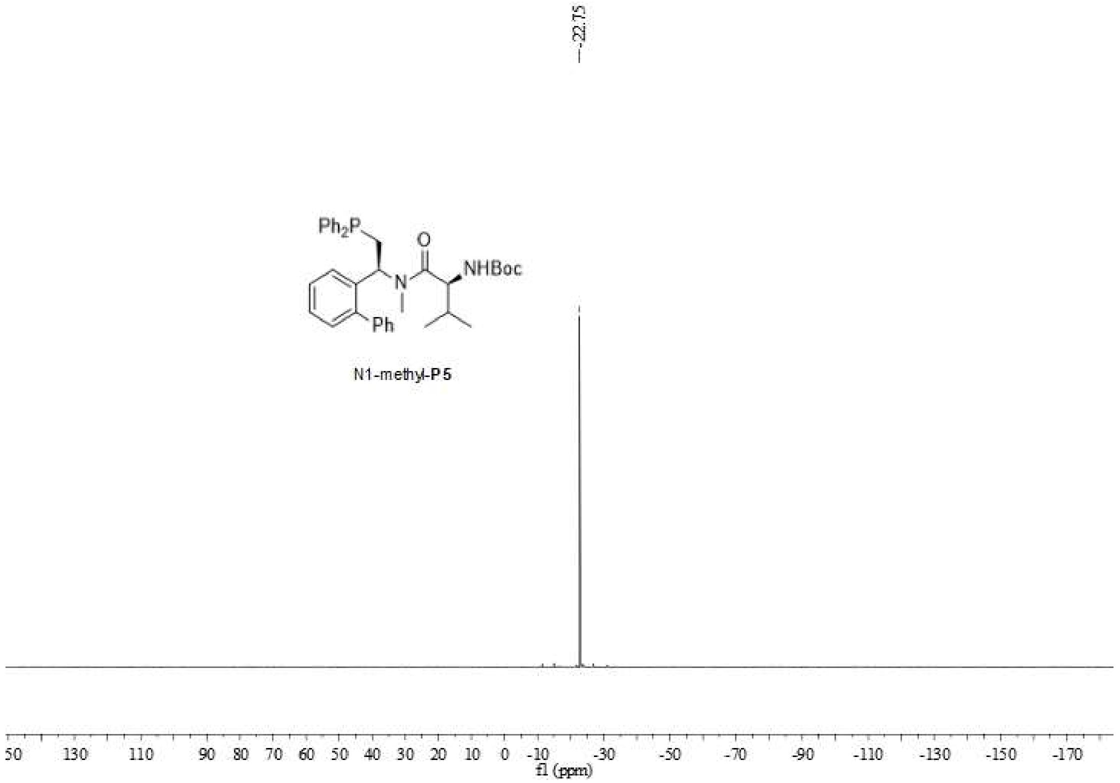
-22.75
Ph2P
NHBoc
Ph
N1-methyl-P5
f1 (ppm)

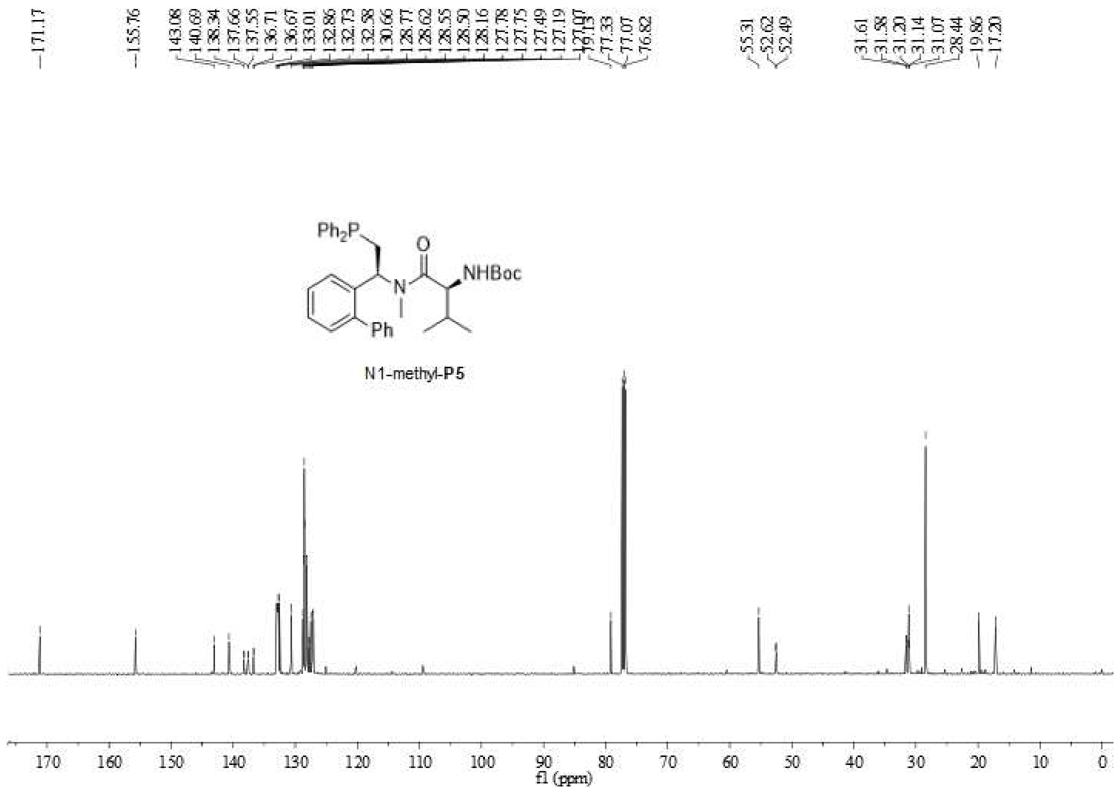
N1-methyl-P5
f1 (ppm)

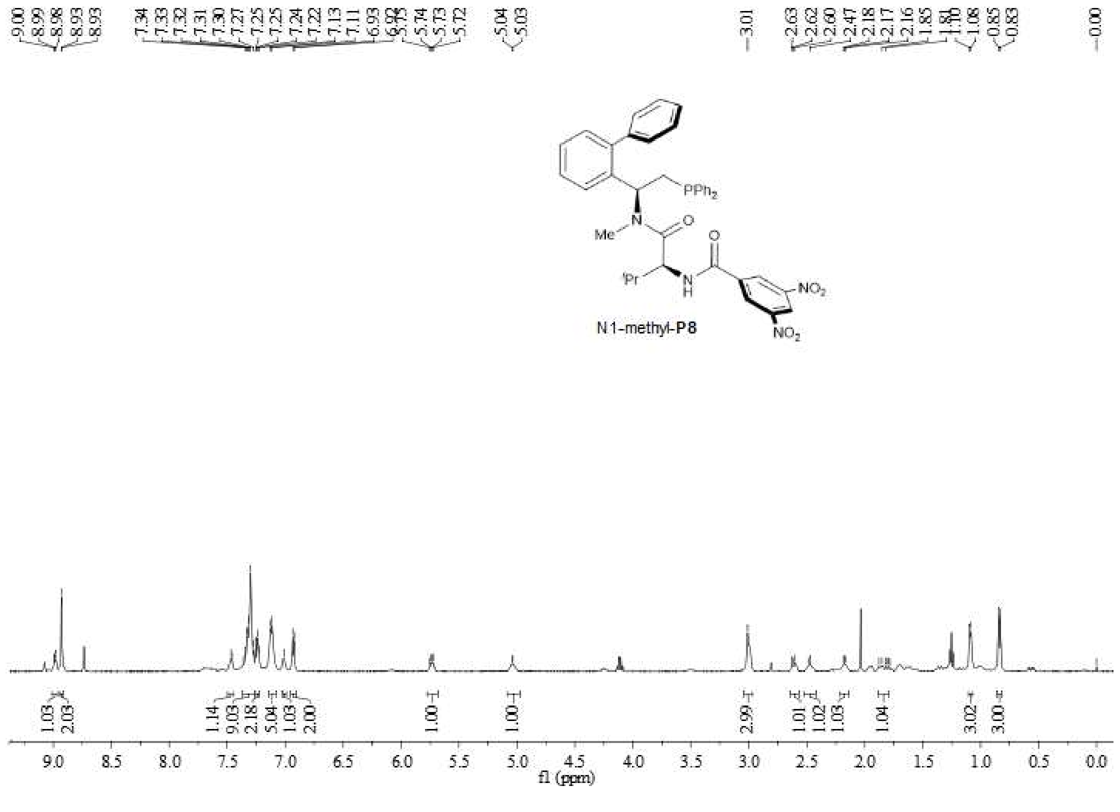
N1-methyl-P8
f1 (ppm)

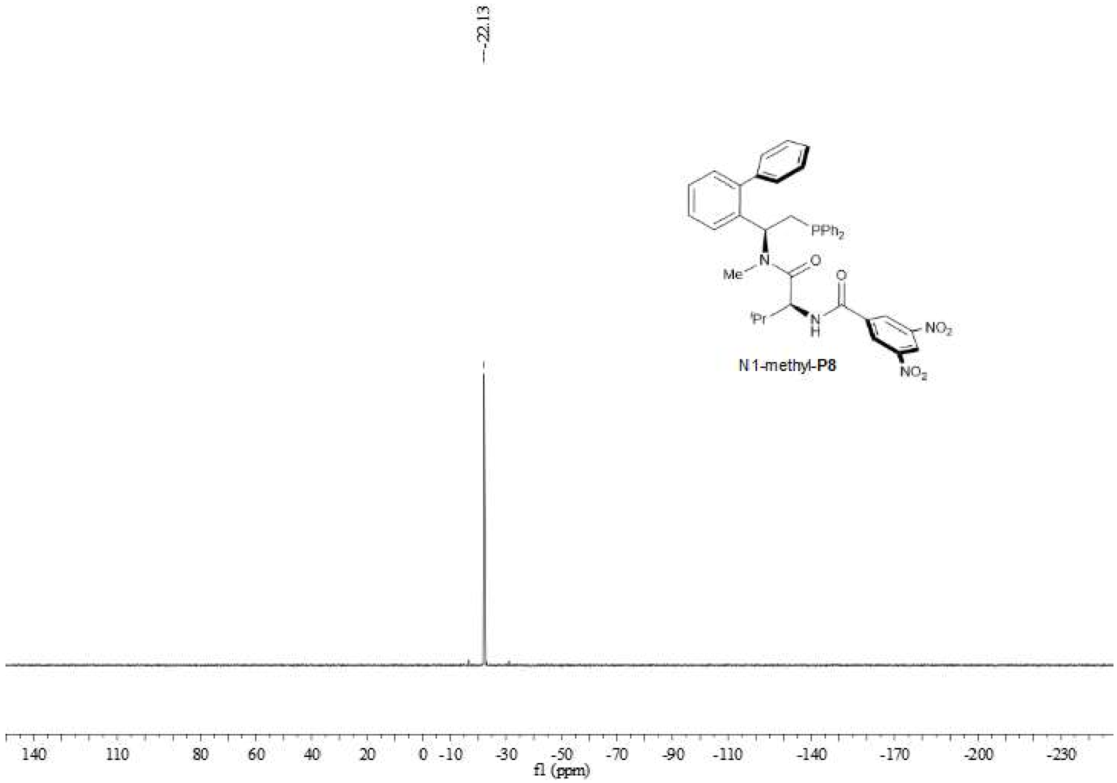

-22.13
PPh2
Me
N
O
O
iPr
N
H
NO2
NO2
N1-methyl-P8
140
110
80
60
40
20
0
-10
-30
-50
-70
-90
-110
-140
-170
-200
-230
f1 (ppm)

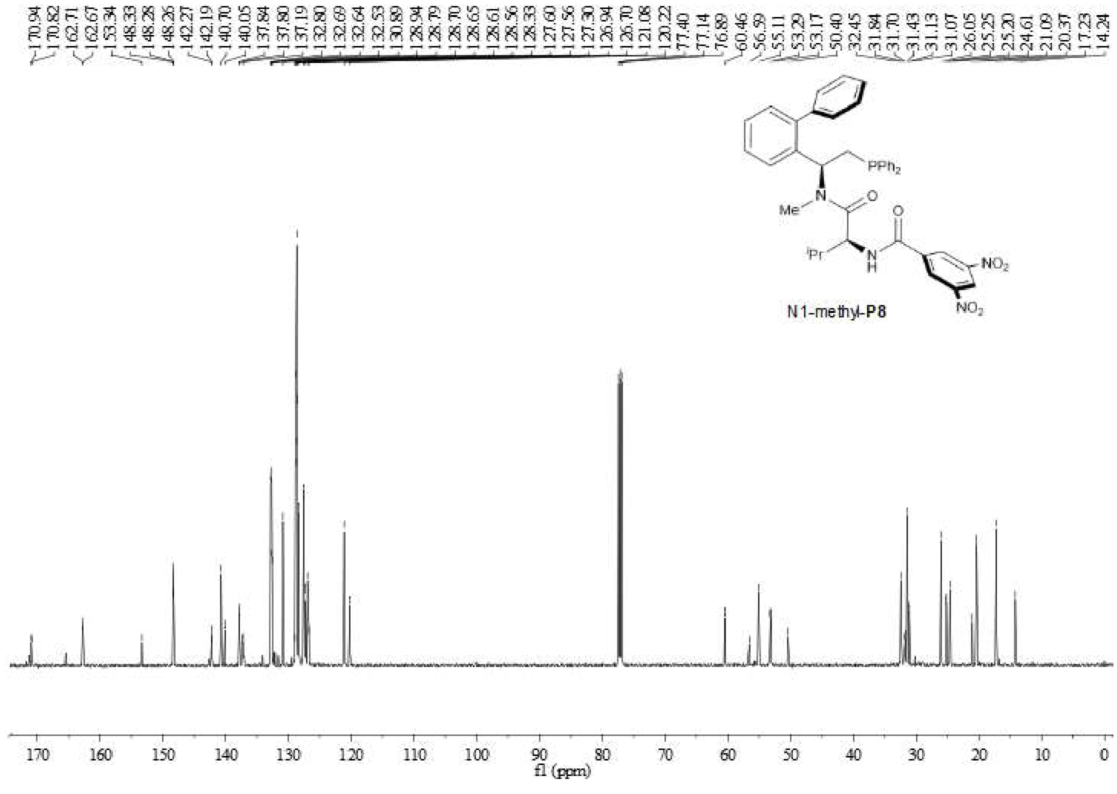

170.94
170.82
162.71
162.67
153.34
148.33
148.28
148.26
142.27
142.19
140.70
140.05
137.84
137.80
137.19
132.80
132.69
132.64
132.53
130.89
128.94
128.79
128.70
128.65
128.61
128.56
128.33
127.60
127.56
127.30
126.94
126.70
121.08
120.22
77.40
77.14
76.89
60.46
56.59
55.11
53.29
53.17
50.40
32.45
31.84
31.70
31.43
31.13
31.07
26.05
25.25
25.20
24.61
21.09
20.37
17.23
14.24
PPh2
Me
N
O
O
iPr
N
H
NO2
NO2
N1-methyl-P8
170
160
150
140
130
120
110
100
90
80
70
60
50
40
30
20
10
0
f1 (ppm)

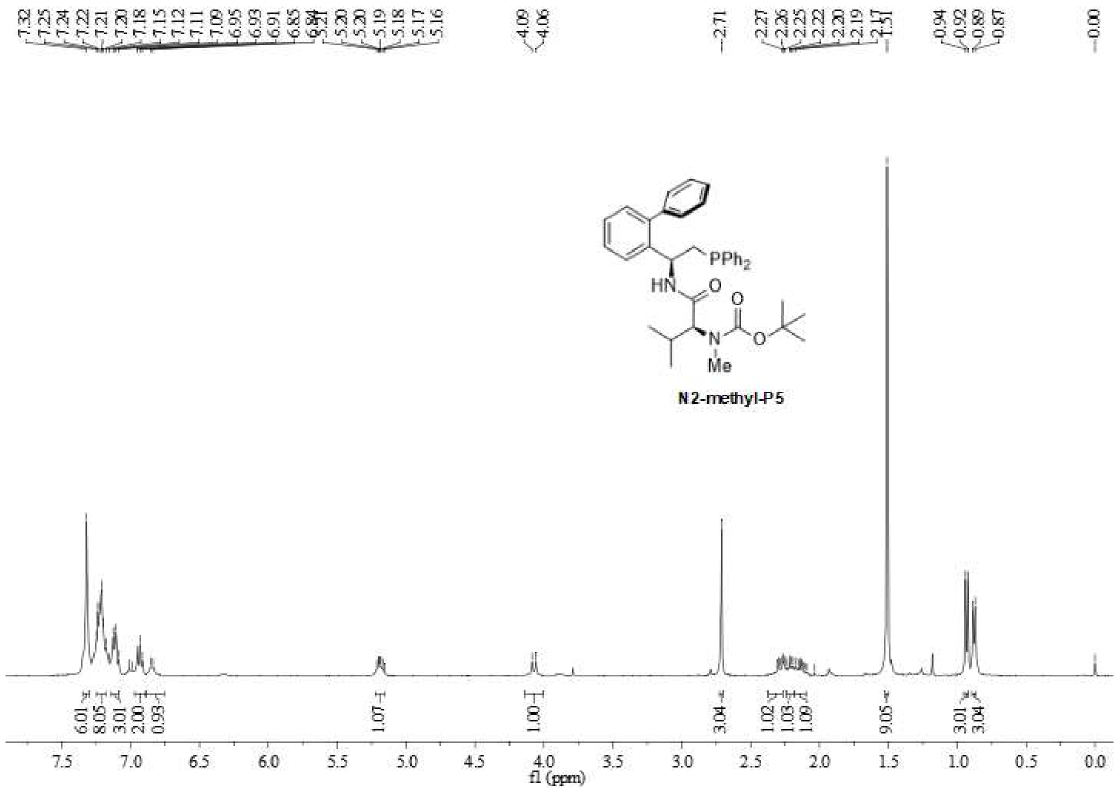
PPh2
HN
O
O
N
O
Me
N2-methyl-P5
fl (ppm)

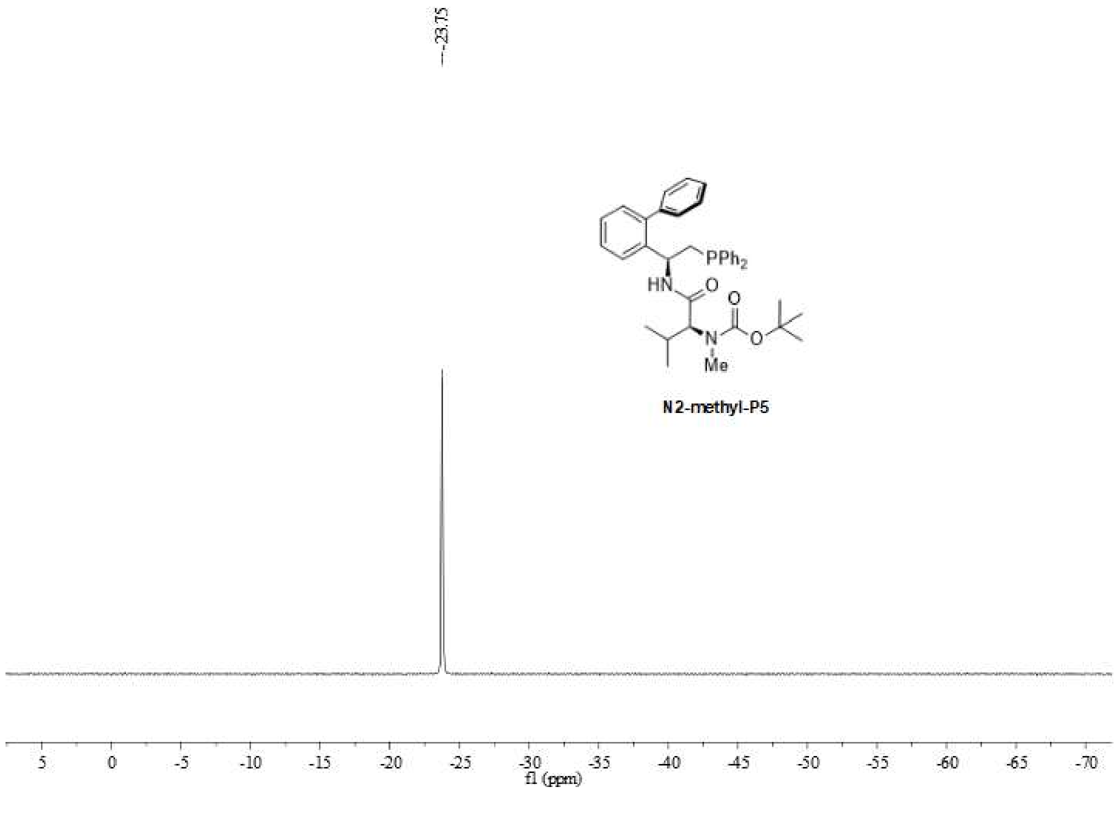
-23.75
PPh2
HN
O
O
N
O
Me
N2-methyl-P5
fl (ppm)

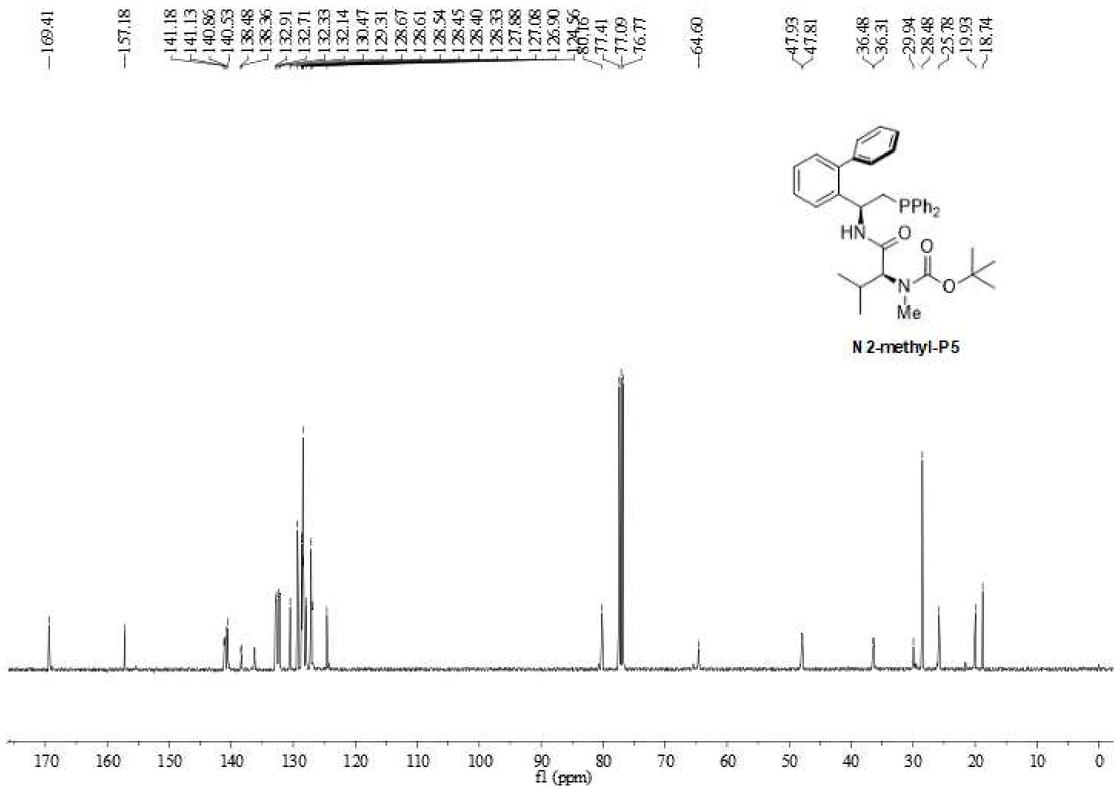
169.41
157.18
141.18
141.13
140.86
140.53
138.48
138.36
132.91
132.71
132.33
132.14
130.47
129.31
128.67
128.61
128.54
128.45
128.40
128.33
127.88
127.08
126.90
124.56
80.16
77.41
77.09
76.77
64.60
47.93
47.81
36.48
36.31
29.94
28.48
25.78
19.93
18.74
PPh2
HN
O
O
N
O
Me
N 2-methyl-P5
f1 (ppm)

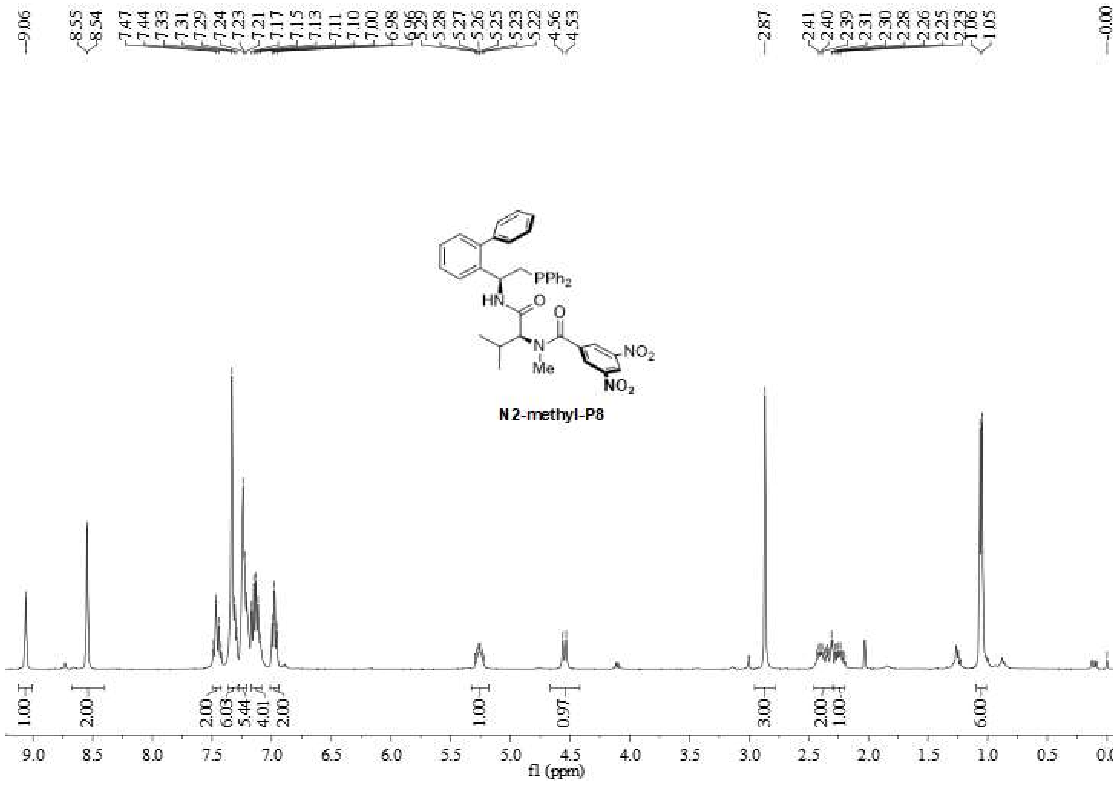
9.06
8.55
8.54
7.47
7.44
7.33
7.31
7.29
7.24
7.23
7.21
7.17
7.15
7.13
7.11
7.10
7.00
6.98
6.96
5.29
5.28
5.27
5.26
5.25
5.23
5.22
4.56
4.53
2.87
2.41
2.40
2.39
2.31
2.30
2.28
2.26
2.25
2.23
1.06
1.05
0.00
PPh2
HN
O
O
N
Me
NO2
NO2
N 2-methyl-P8
1.00
2.00
2.00
6.03
5.44
4.01
2.00
1.00
0.97
3.00
2.00
1.00
6.00
f1 (ppm)

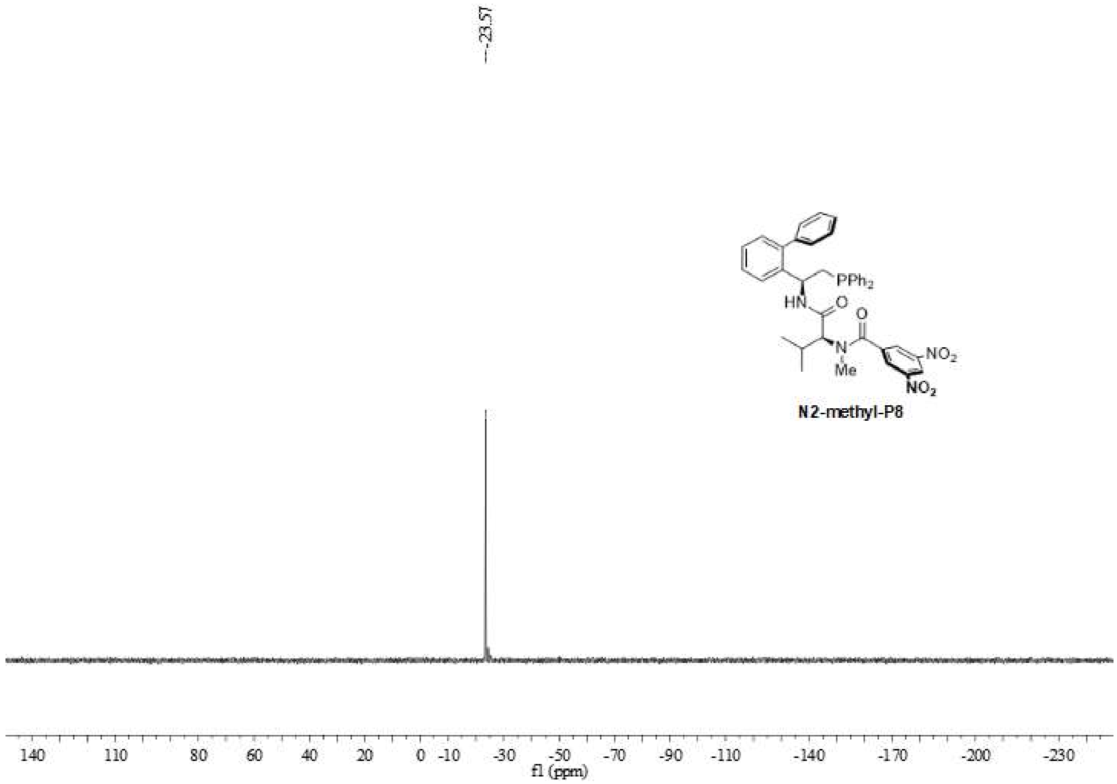
--23.57
PPh2
HN
O
O
N
Me
NO2
NO2
N2-methyl-P8
140
110
80
60
40
20
0
-10
-30
-50
-70
-90
-110
-140
-170
-200
-230
f1 (ppm)

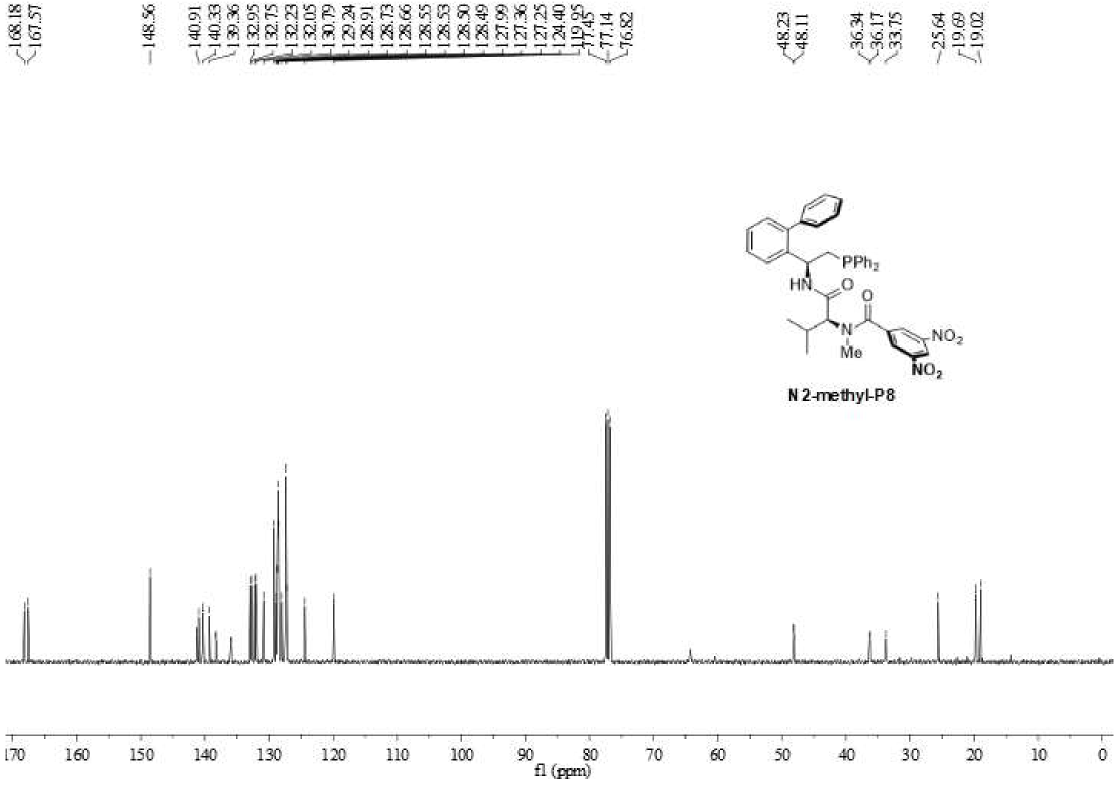
168.18
167.57
148.56
140.91
140.33
139.36
132.95
132.75
132.23
132.05
130.79
129.24
128.91
128.73
128.66
128.55
128.53
128.50
128.49
127.99
127.36
127.25
124.40
119.95
77.45
77.14
76.82
48.23
48.11
36.34
36.17
33.75
25.64
19.69
19.02
PPh2
HN
O
O
N
Me
NO2
NO2
N2-methyl-P8
170
160
150
140
130
120
110
100
90
80
70
60
50
40
30
20
10
0
f1 (ppm)

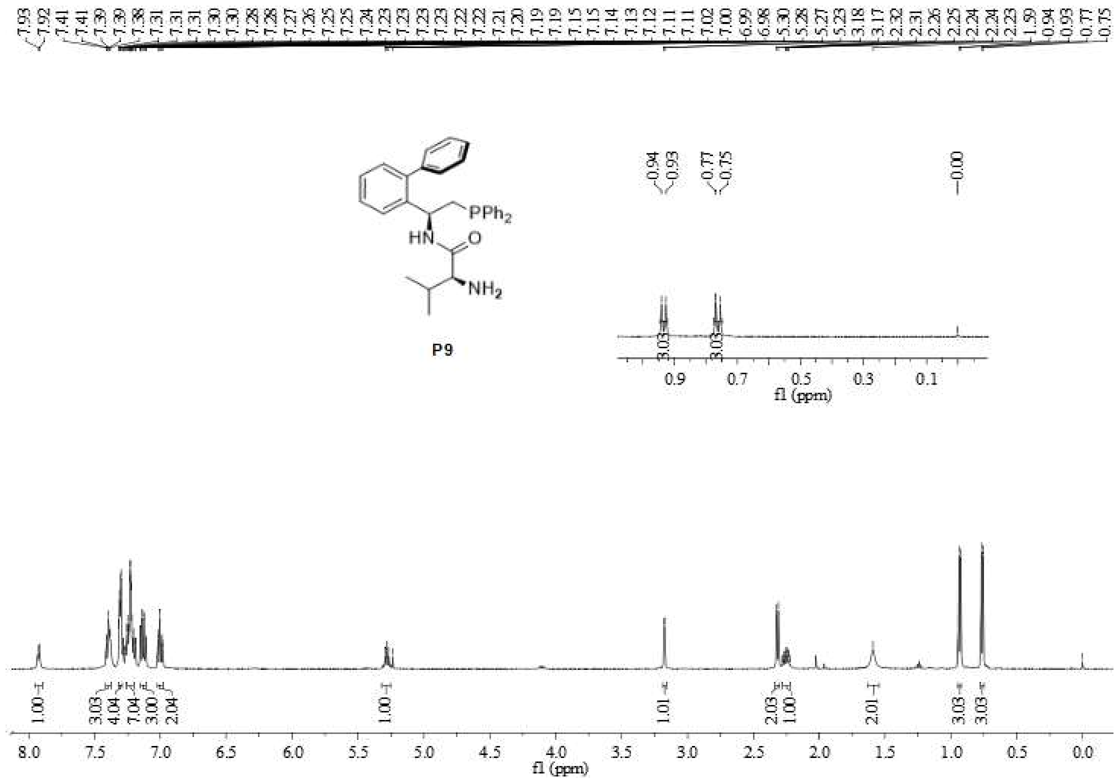
7.93
7.92
7.41
7.41
7.39
7.39
7.38
7.31
7.31
7.30
7.30
7.28
7.28
7.27
7.26
7.25
7.25
7.24
7.23
7.23
7.23
7.23
7.22
7.22
7.21
7.20
7.19
7.19
7.15
7.15
7.14
7.13
7.12
7.11
7.11
7.02
7.00
6.99
6.98
5.30
5.28
5.27
5.23
3.18
3.17
2.32
2.31
2.26
2.25
2.24
2.24
2.23
1.59
0.94
0.93
0.77
0.75
PPh2
HN
O
NH2
P9
0.94
0.93
0.77
0.75
0.00
3.03
3.03
0.9
0.7
0.5
0.3
0.1
f1 (ppm)
1.00
3.03
4.04
7.04
3.00
2.04
1.00
1.01
2.03
1.00
2.01
3.03
3.03
8.0
7.5
7.0
6.5
6.0
5.5
5.0
4.5
4.0
3.5
3.0
2.5
2.0
1.5
1.0
0.5
0.0
f1 (ppm)

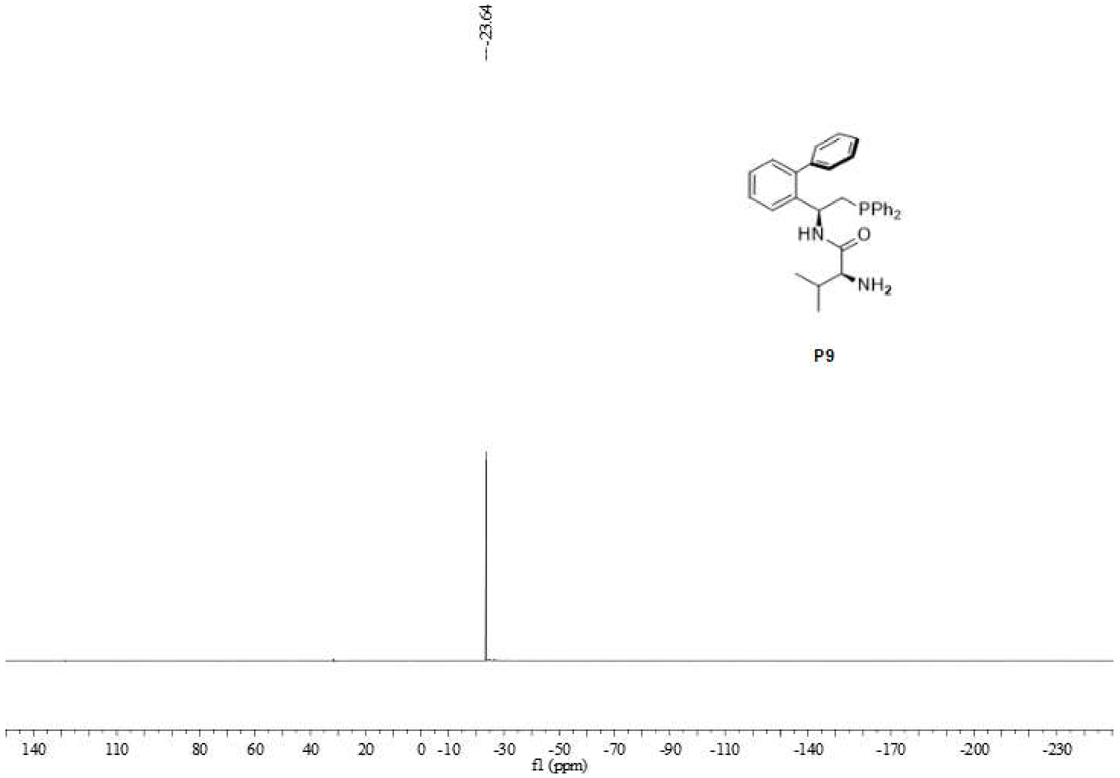
-23.64
PPh2
HN
O
NH2
P9
140
110
80
60
40
20
0
-10
-30
-50
-70
-90
-110
-140
-170
-200
-230
f1 (ppm)

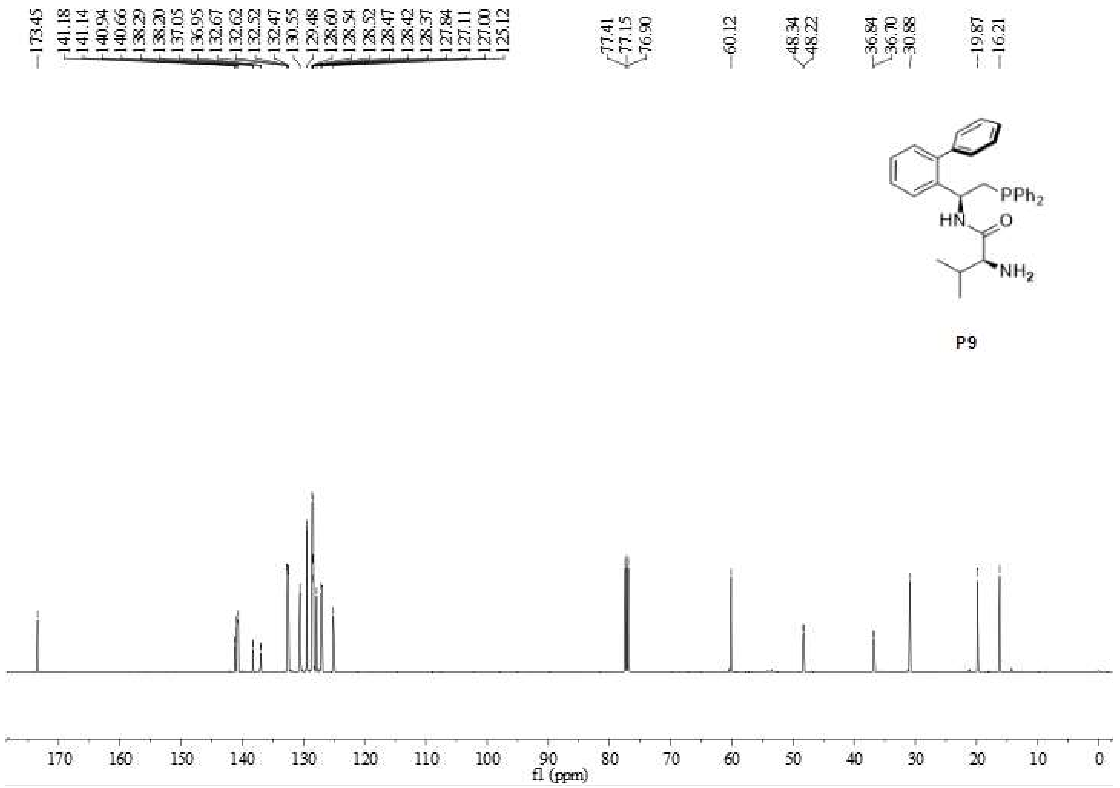
173.45
141.18
141.14
140.94
140.66
138.29
138.20
137.05
136.95
132.67
132.62
132.52
132.47
130.55
129.48
128.60
128.54
128.52
128.47
128.42
128.37
127.84
127.11
127.00
125.12
77.41
77.15
76.90
60.12
48.34
48.22
36.84
36.70
30.88
19.87
16.21
PPh2
HN
O
NH2
P9
f1 (ppm)

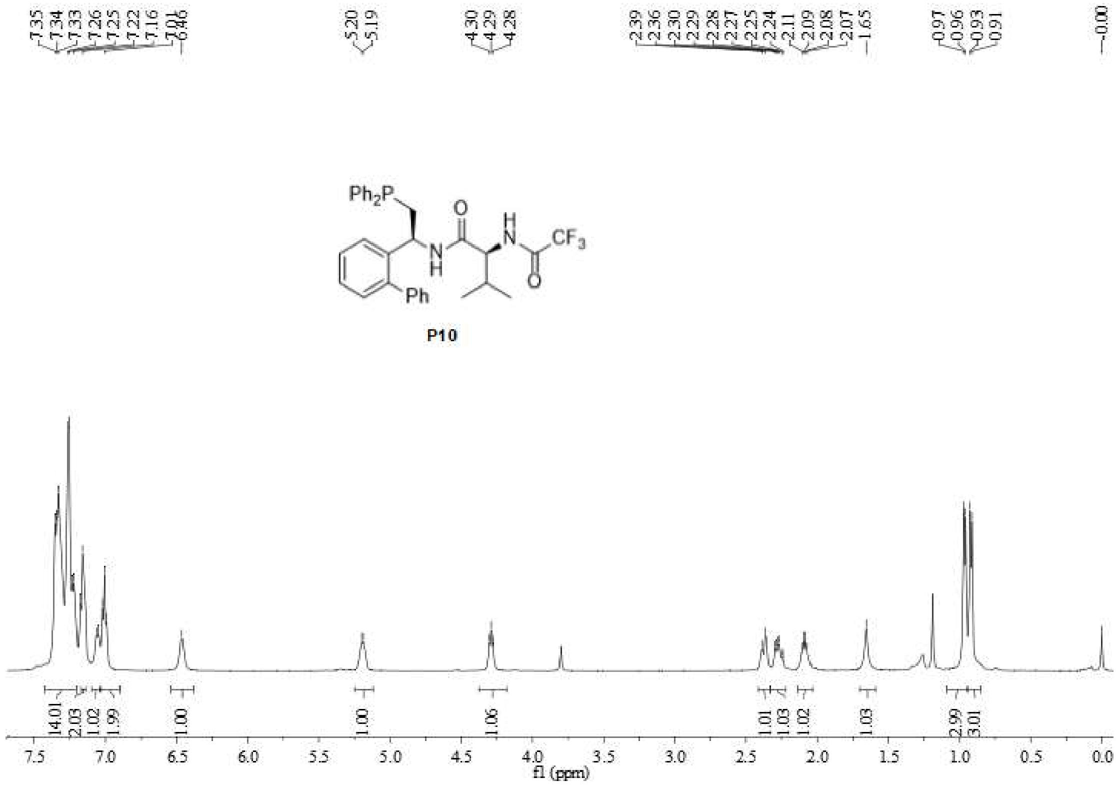
7.35
7.34
7.33
7.26
7.25
7.22
7.16
7.01
6.46
5.20
5.19
4.30
4.29
4.28
2.39
2.36
2.30
2.29
2.28
2.27
2.25
2.24
2.11
2.09
2.08
2.07
1.65
0.97
0.96
0.93
0.91
-0.00
Ph2P
O
H
N
CF3
N
H
O
Ph
P10
14.01
2.03
1.02
1.99
1.00
1.00
1.06
1.01
1.03
1.02
1.03
2.99
3.01
f1 (ppm)

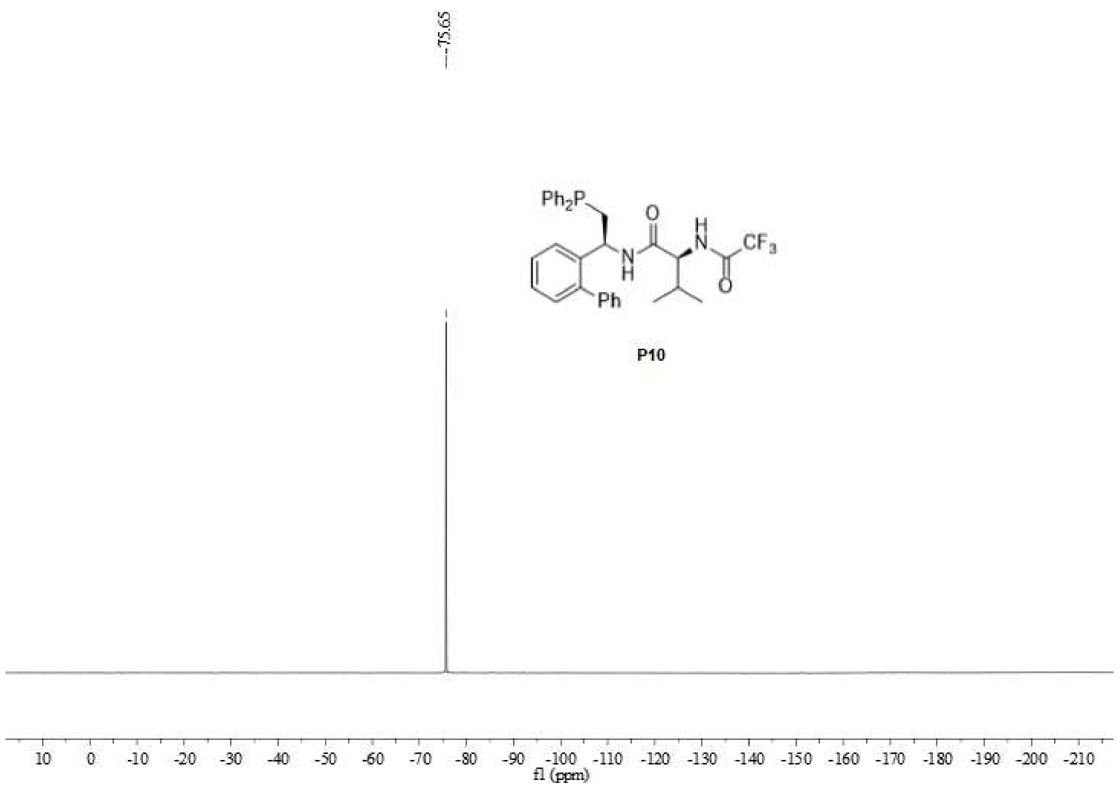
-75.65
Ph2P
CF3
Ph
P10
10 0 -10 -20 -30 -40 -50 -60 -70 -80 -90 -100 -110 -120 -130 -140 -150 -160 -170 -180 -190 -200 -210
f1 (ppm)

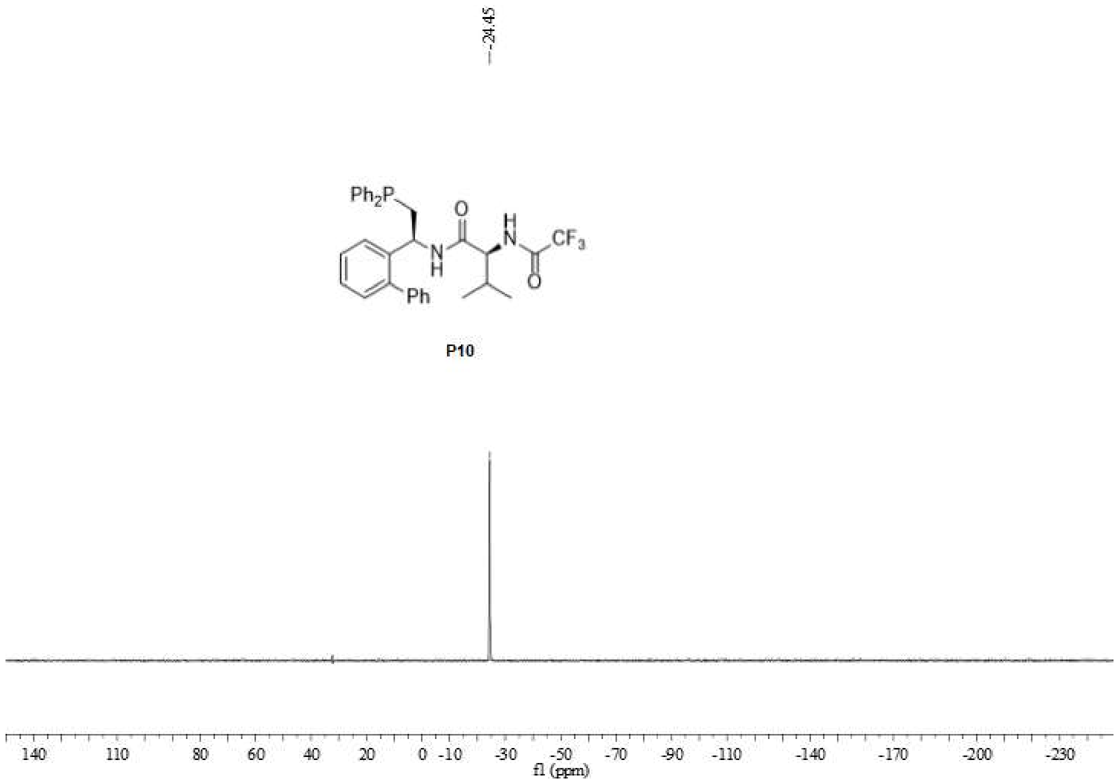
-24.45
Ph2P
CF3
Ph
P10
140 110 80 60 40 20 0 -10 -30 -50 -70 -90 -110 -140 -170 -200 -230
f1 (ppm)

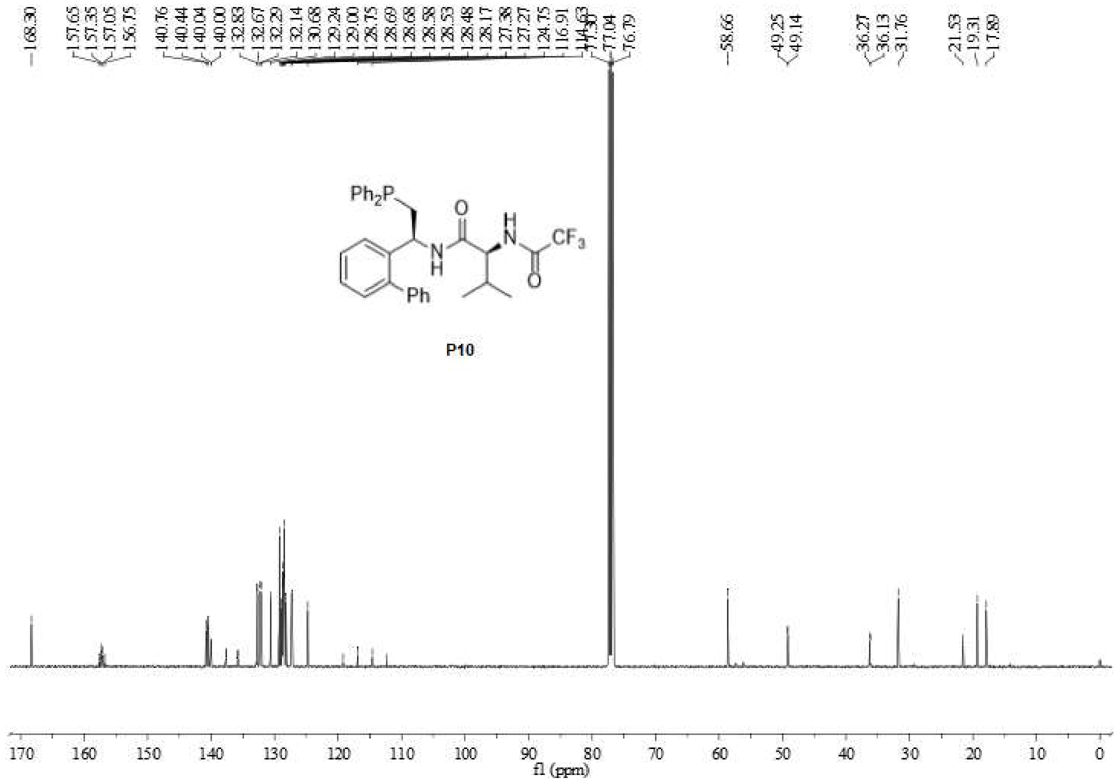
168.30
157.65
157.35
157.05
156.75
140.76
140.44
140.04
140.00
132.83
132.67
132.29
132.14
130.68
129.24
129.00
128.75
128.69
128.68
128.58
128.53
128.48
128.17
127.38
127.27
124.75
116.91
114.63
77.30
77.04
76.79
58.66
49.25
49.14
36.27
36.13
31.76
21.53
19.31
17.89
Ph2P
N
H
O
H
N
CF3
O
Ph
P10
170 160 150 140 130 120 110 100 90 80 70 60 50 40 30 20 10 0
f1 (ppm)

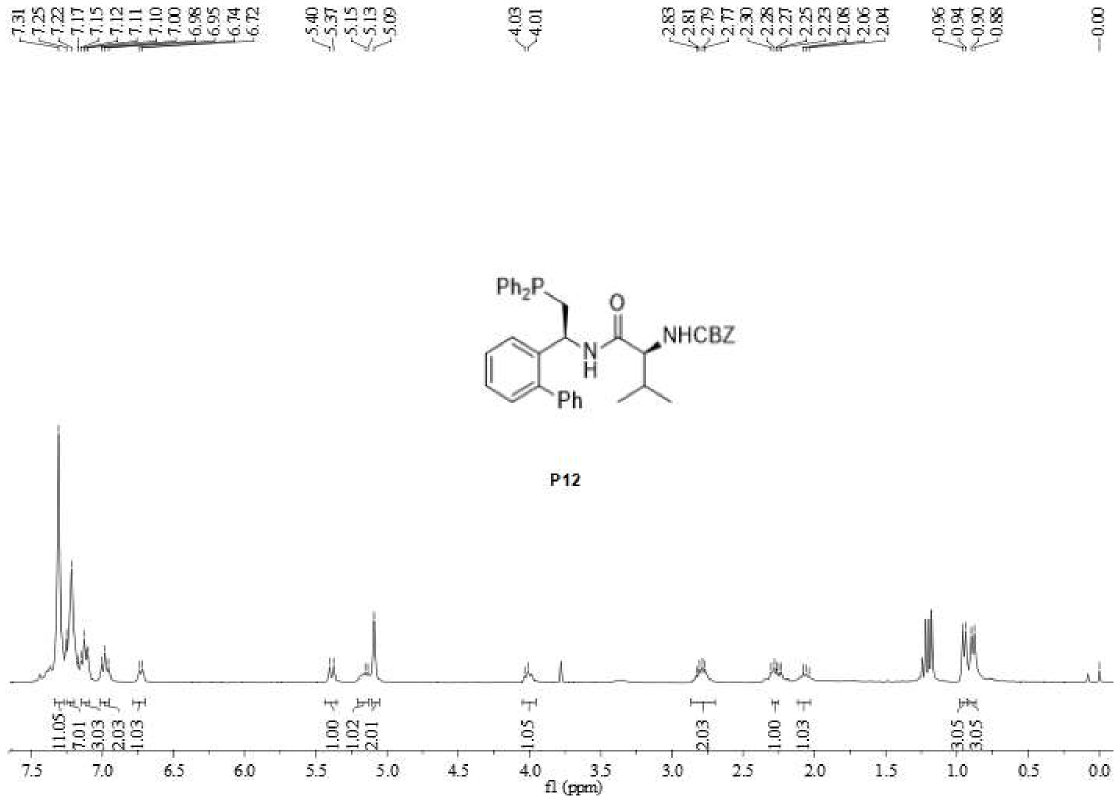
7.31
7.25
7.22
7.17
7.15
7.12
7.11
7.10
7.00
6.98
6.95
6.74
6.72
5.40
5.37
5.15
5.13
5.09
4.03
4.01
2.83
2.81
2.79
2.77
2.30
2.28
2.27
2.25
2.23
2.08
2.06
2.04
0.96
0.94
0.90
0.88
0.00
Ph2P
N
H
O
NHCBZ
Ph
P12
11.05
7.01
3.03
2.03
1.03
1.00
1.02
2.01
1.05
2.03
1.00
1.03
3.05
3.05
7.5 7.0 6.5 6.0 5.5 5.0 4.5 4.0 3.5 3.0 2.5 2.0 1.5 1.0 0.5 0.0
f1 (ppm)

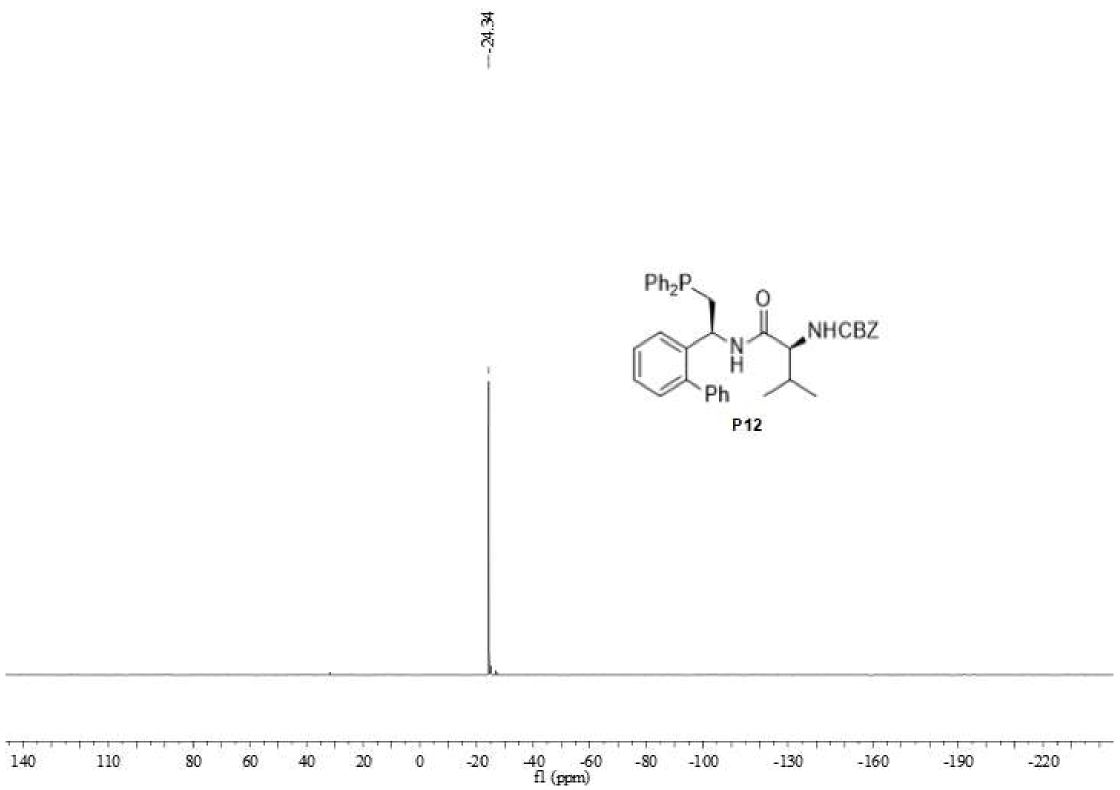

--24.34
Ph2P
O
NHCBZ
N
H
Ph
P12
140
110
80
60
40
20
0
-20
-40
-60
-80
-100
-130
-160
-190
-220
f1 (ppm)

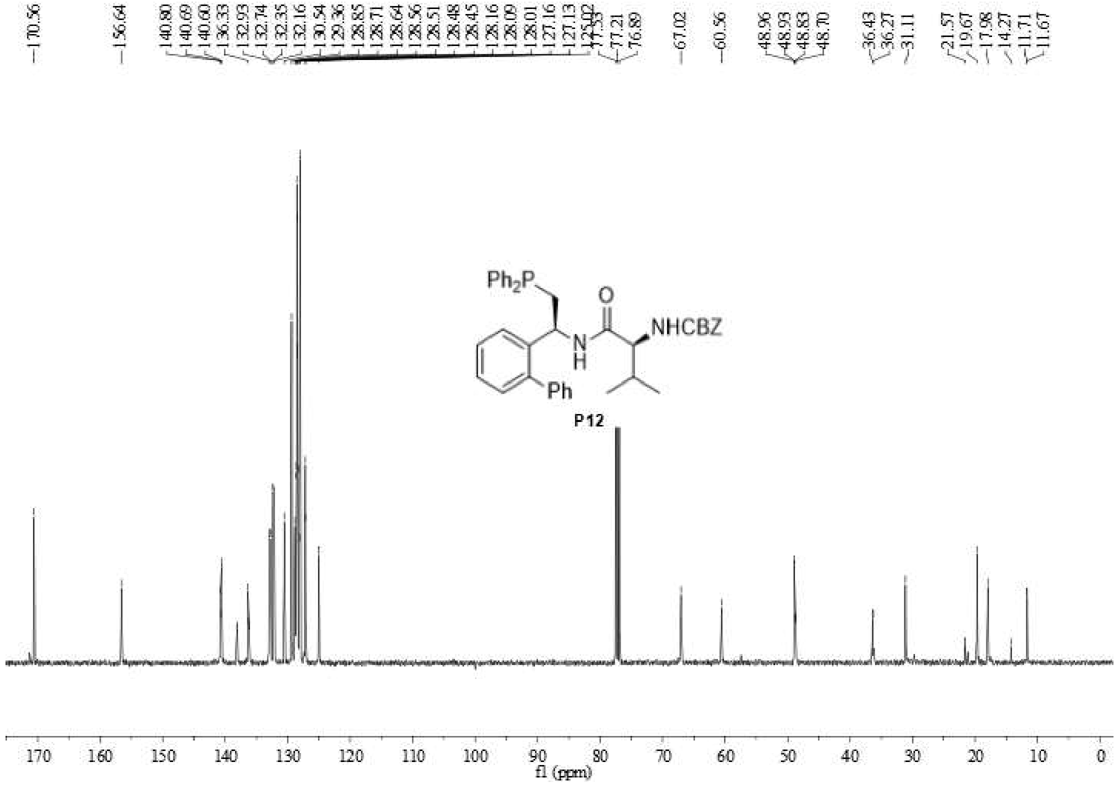

170.56
156.64
140.80
140.69
140.60
136.33
132.93
132.74
132.35
132.16
130.54
129.36
128.85
128.71
128.64
128.56
128.51
128.48
128.45
128.16
128.09
128.01
127.16
127.13
125.02
77.53
77.21
76.89
67.02
60.56
48.96
48.93
48.83
48.70
36.43
36.27
31.11
21.57
19.67
17.98
14.27
11.71
11.67
Ph2P
O
NHCBZ
N
H
Ph
P12
170
160
150
140
130
120
110
100
90
80
70
60
50
40
30
20
10
0
f1 (ppm)

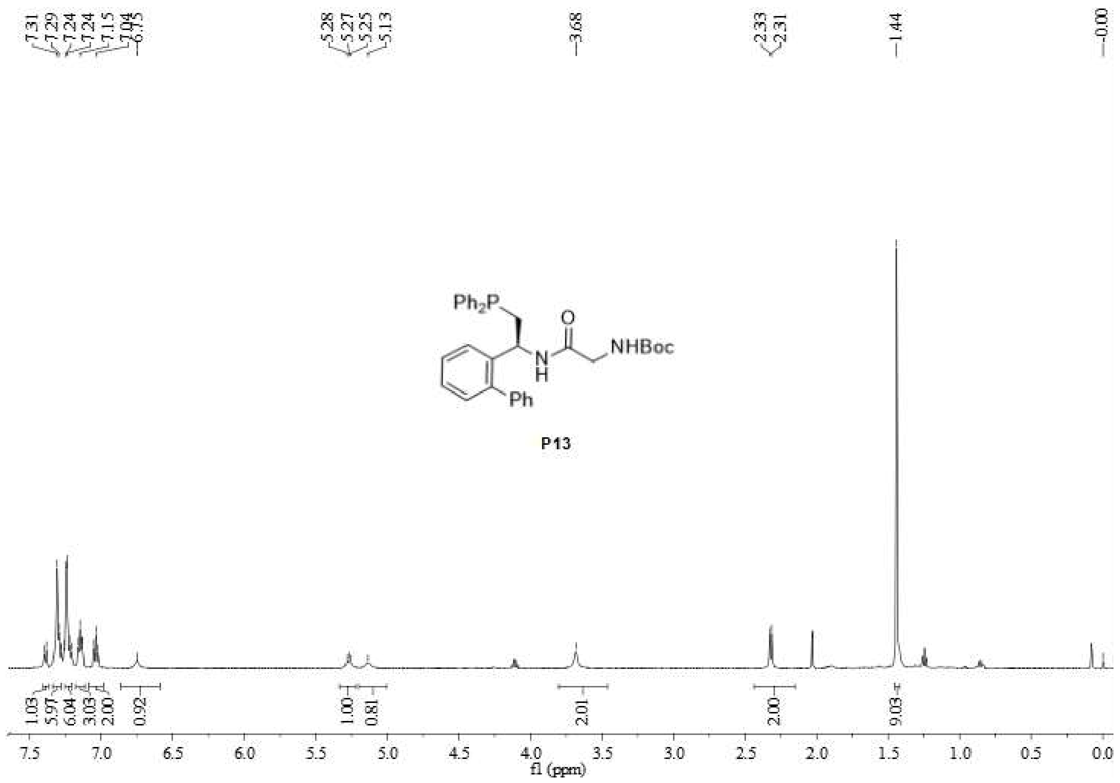

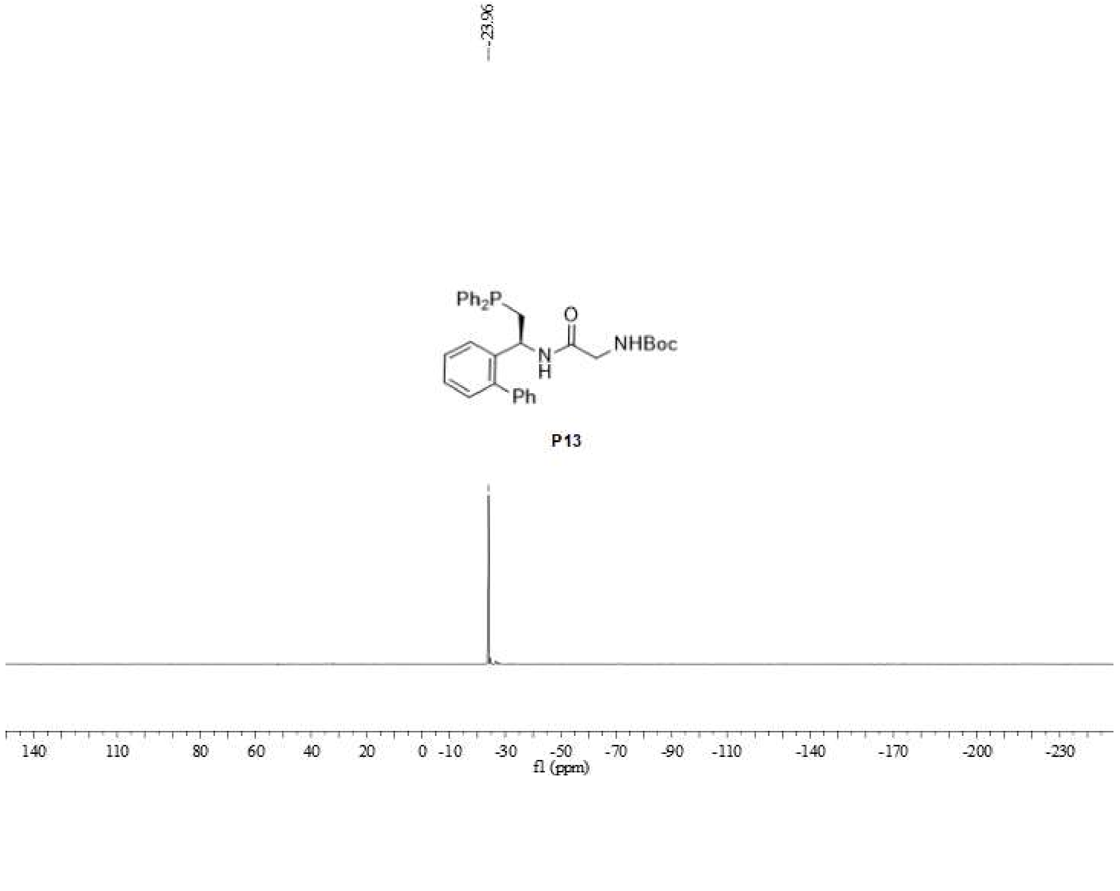

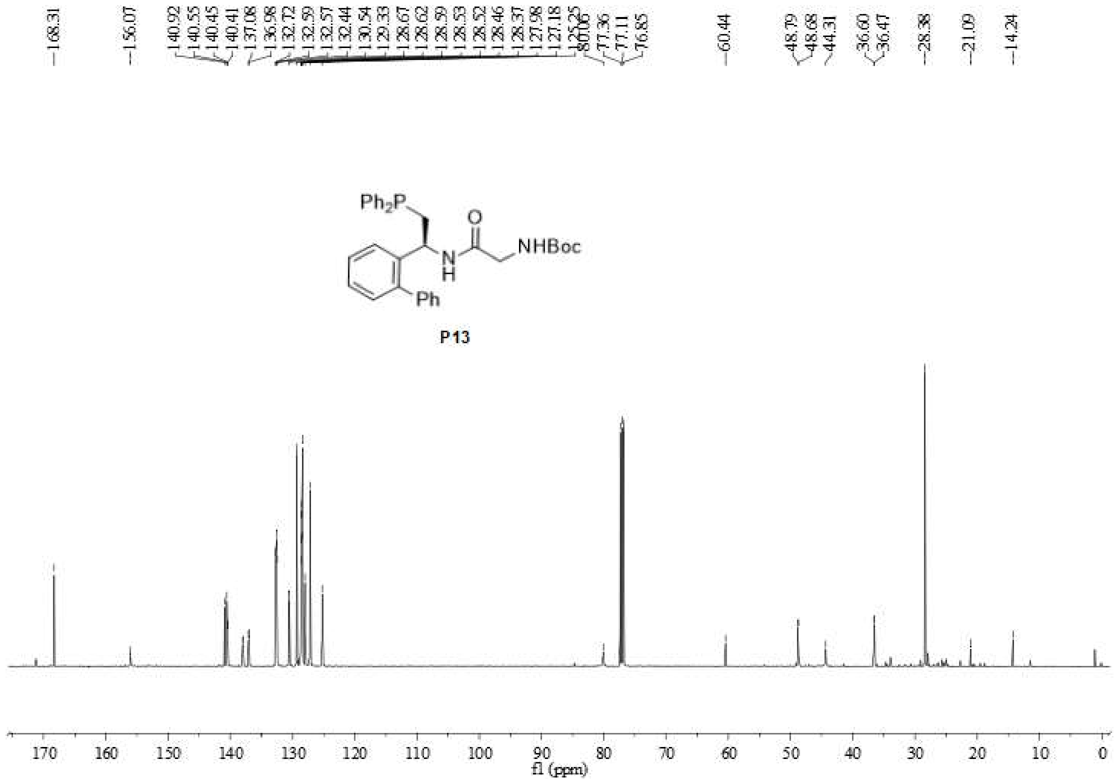
168.31
156.07
140.92
140.55
140.45
140.41
137.08
136.98
132.72
132.59
132.57
132.44
130.54
129.33
128.67
128.62
128.59
128.53
128.52
128.46
128.37
127.98
127.18
125.25
80.06
77.36
77.11
76.85
60.44
48.79
48.68
44.31
36.60
36.47
28.38
21.09
14.24
Ph2P
O
N
H
NHBoc
Ph
f1 (ppm)

P13

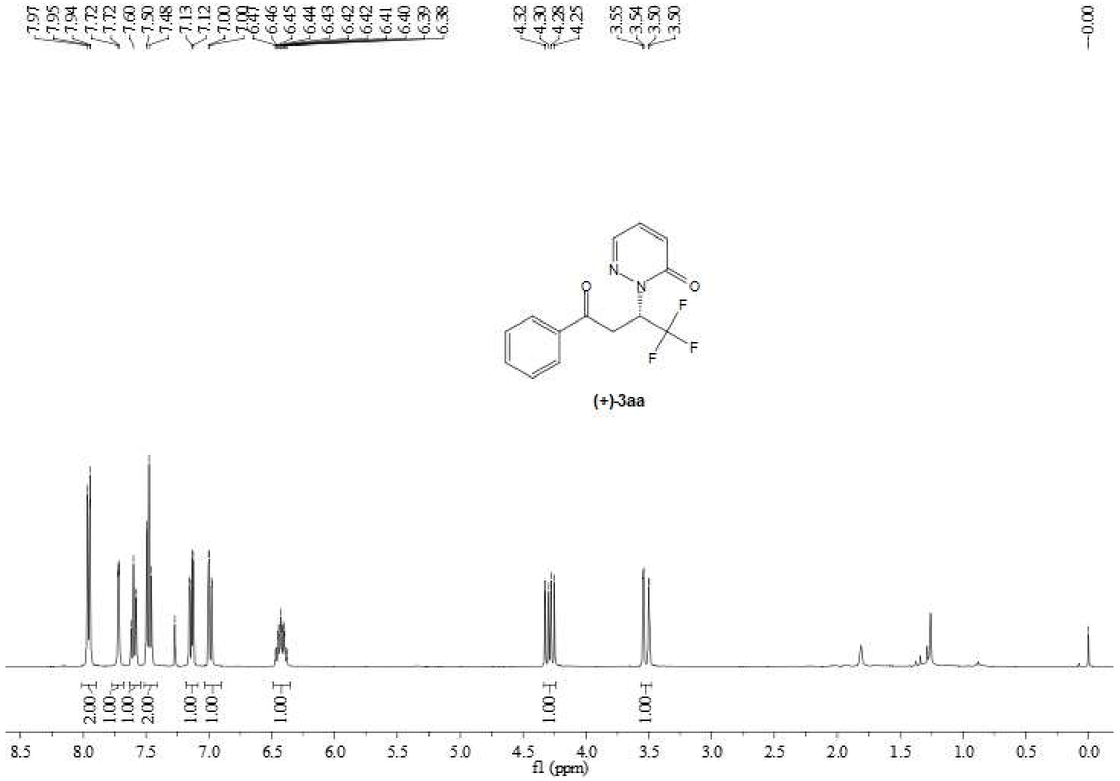
7.97
7.95
7.94
7.72
7.72
7.60
7.50
7.48
7.13
7.12
7.00
7.00
6.47
6.46
6.45
6.44
6.43
6.42
6.42
6.41
6.40
6.39
6.38
4.32
4.30
4.28
4.25
3.55
3.54
3.50
3.50
0.00
N
N
O
O
F
F
F
2.00
1.00
1.00
2.00
1.00
1.00
1.00
1.00
1.00
f1 (ppm)

(+)-3aa

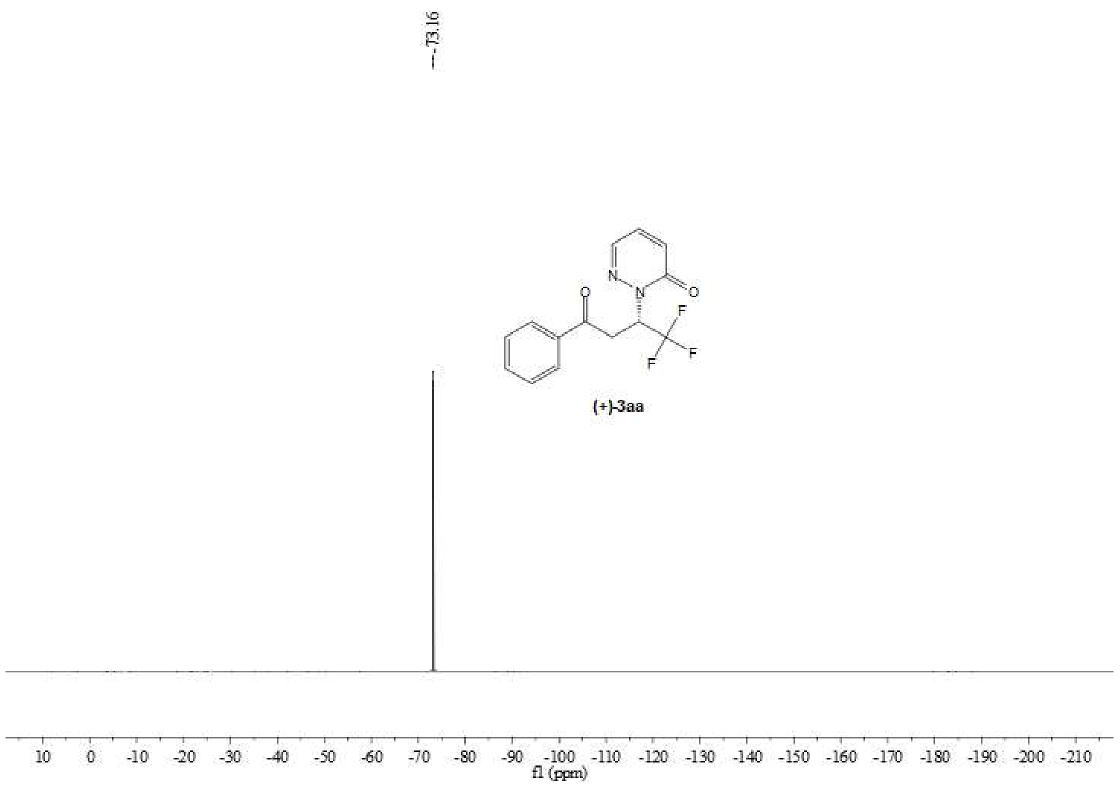
-73.16
(+)-3aa
10 0 -10 -20 -30 -40 -50 -60 -70 -80 -90 -100 -110 -120 -130 -140 -150 -160 -170 -180 -190 -200 -210
f1 (ppm)

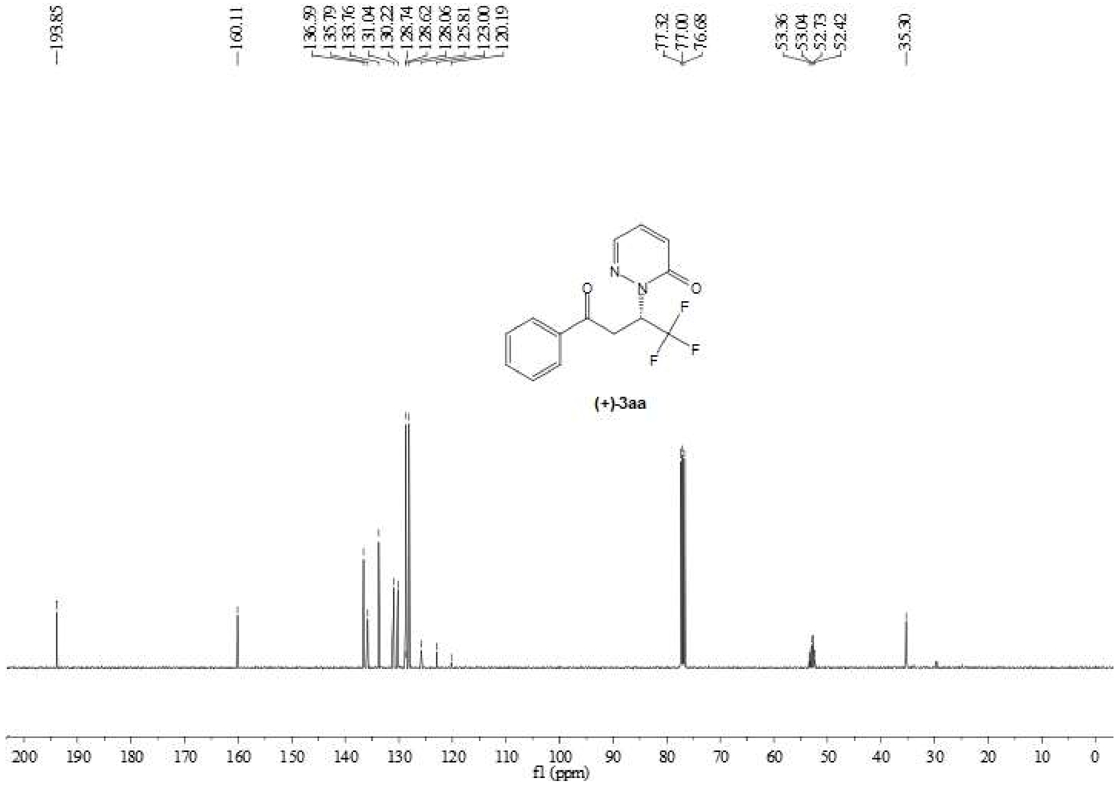
193.85
160.11
136.59
135.79
133.76
131.04
130.22
128.74
128.62
128.06
125.81
123.00
120.19
77.32
77.00
76.68
53.36
53.04
52.73
52.42
35.30
(+)-3aa
200 190 180 170 160 150 140 130 120 110 100 90 80 70 60 50 40 30 20 10 0
f1 (ppm)

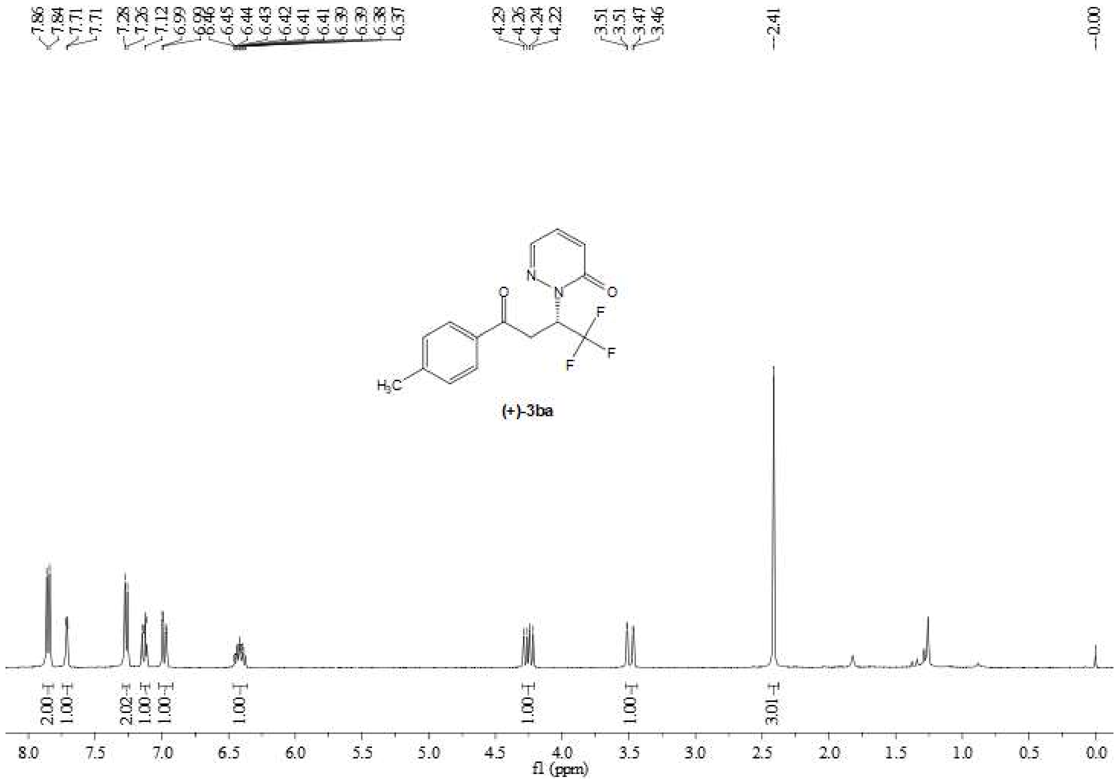
7.86
7.84
7.71
7.71
7.28
7.26
7.12
6.99
6.46
6.45
6.44
6.43
6.42
6.41
6.41
6.39
6.39
6.38
6.37
4.29
4.26
4.24
4.22
3.51
3.51
3.47
3.46
2.41
0.00
(+)-3ba
2.00
1.00
2.02
1.00
1.00
1.00
1.00
1.00
3.01
8.0 7.5 7.0 6.5 6.0 5.5 5.0 4.5 4.0 3.5 3.0 2.5 2.0 1.5 1.0 0.5 0.0
f1 (ppm)

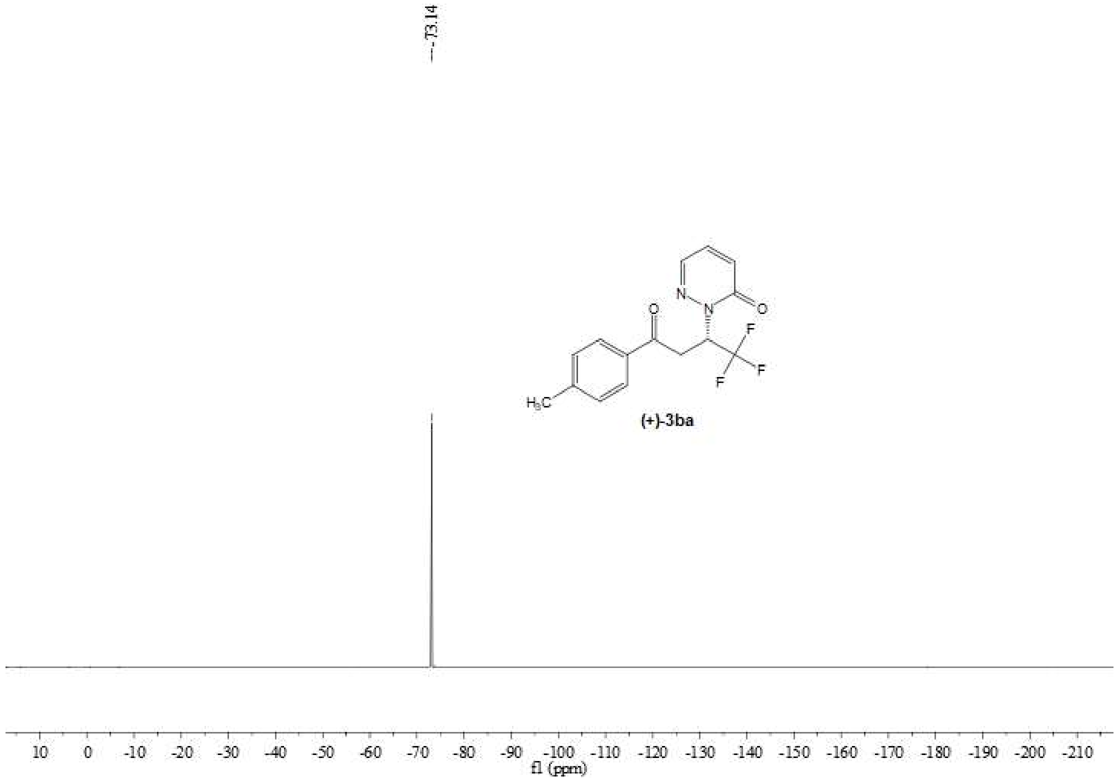
-73.14
(+)-3ba
10 0 -10 -20 -30 -40 -50 -60 -70 -80 -90 -100 -110 -120 -130 -140 -150 -160 -170 -180 -190 -200 -210
f1 (ppm)

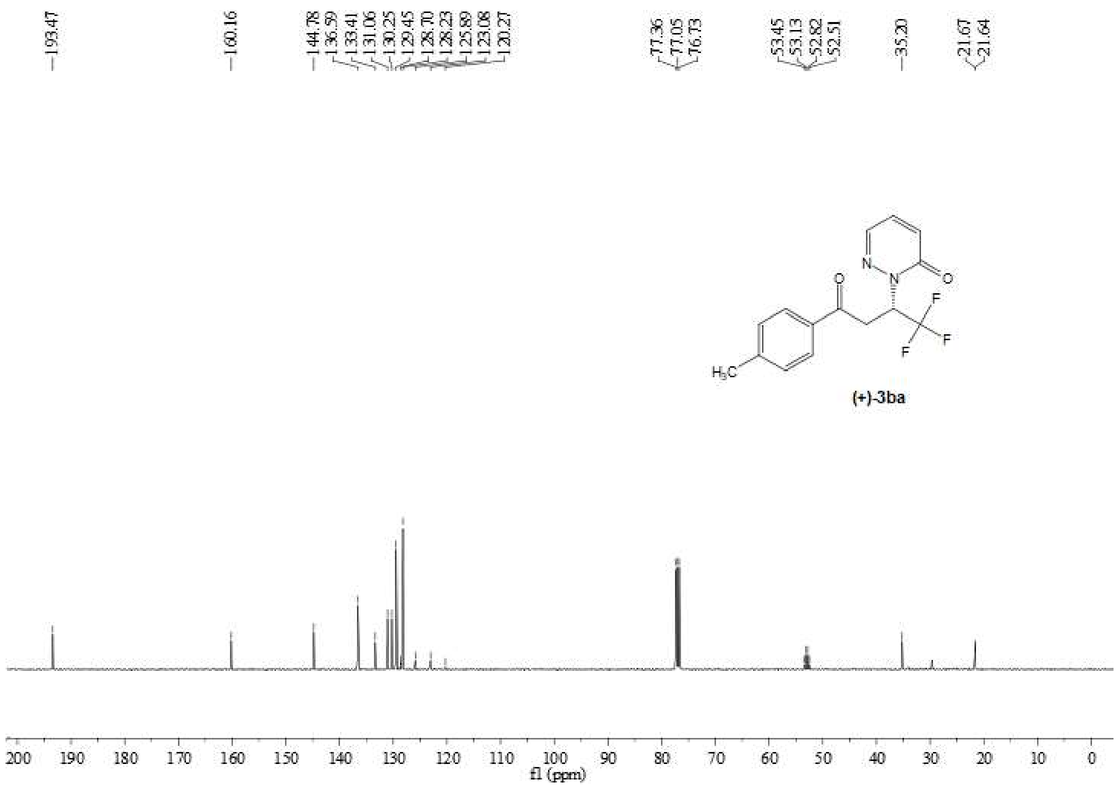
193.47
160.16
144.78
136.59
133.41
131.06
130.25
129.45
128.70
128.23
125.89
123.08
120.27
77.36
77.05
76.73
53.45
53.13
52.82
52.51
35.20
21.67
21.64
(+)-3ba
fl (ppm)

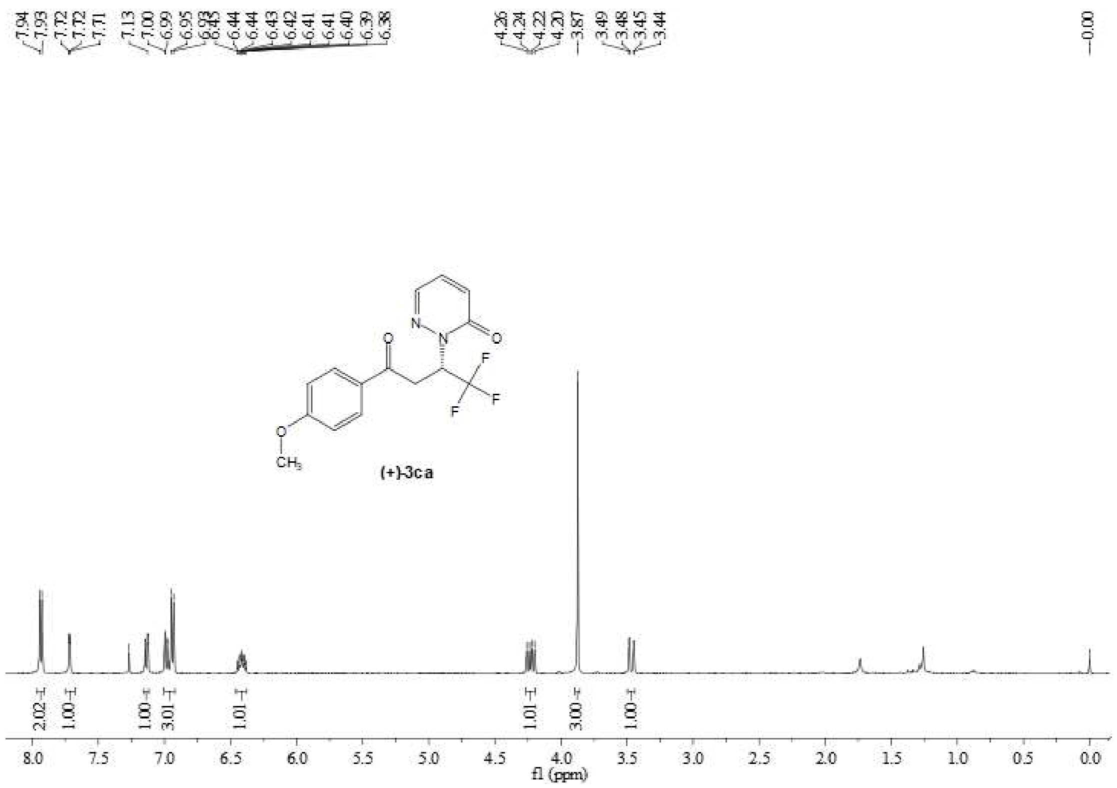
7.94
7.93
7.72
7.72
7.71
7.13
7.00
6.99
6.95
6.93
6.45
6.44
6.44
6.43
6.42
6.41
6.40
6.39
6.38
4.26
4.24
4.22
4.20
3.87
3.49
3.48
3.45
3.44
0.00
(+)-3ca
2.02
1.00
1.00
3.01
1.01
1.01
3.00
1.00
fl (ppm)

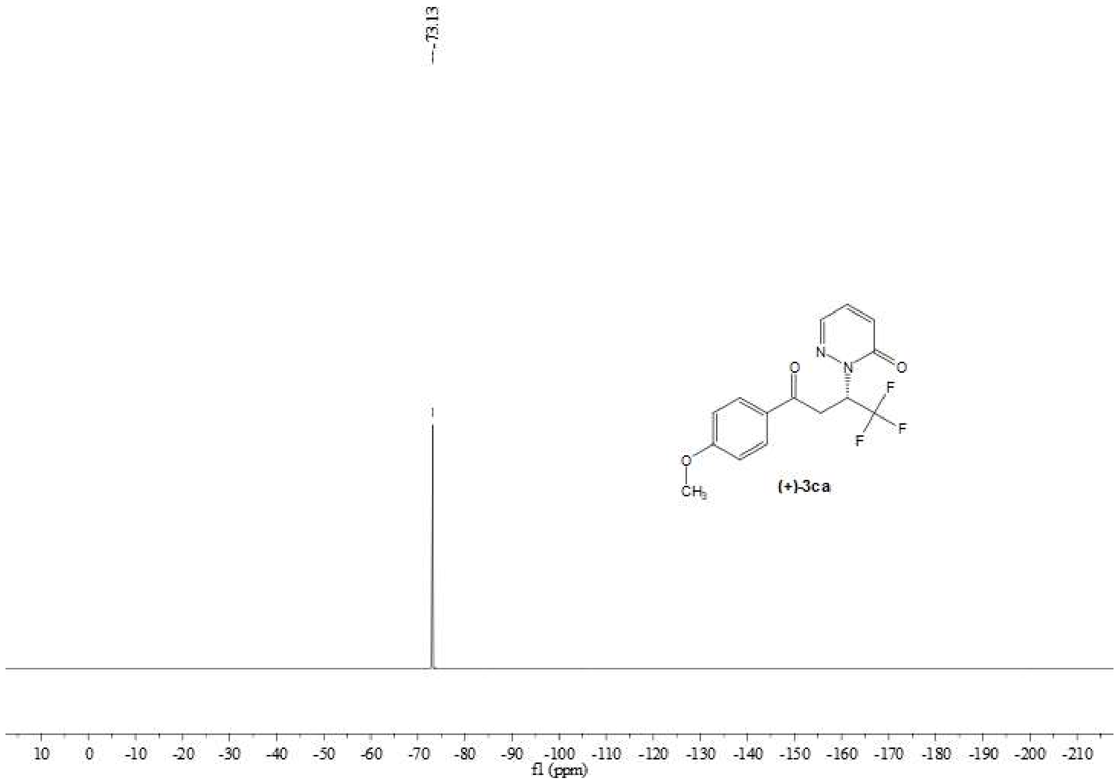

--73.13
(+)-3ca
10 0 -10 -20 -30 -40 -50 -60 -70 -80 -90 -100 -110 -120 -130 -140 -150 -160 -170 -180 -190 -200 -210
f1 (ppm)

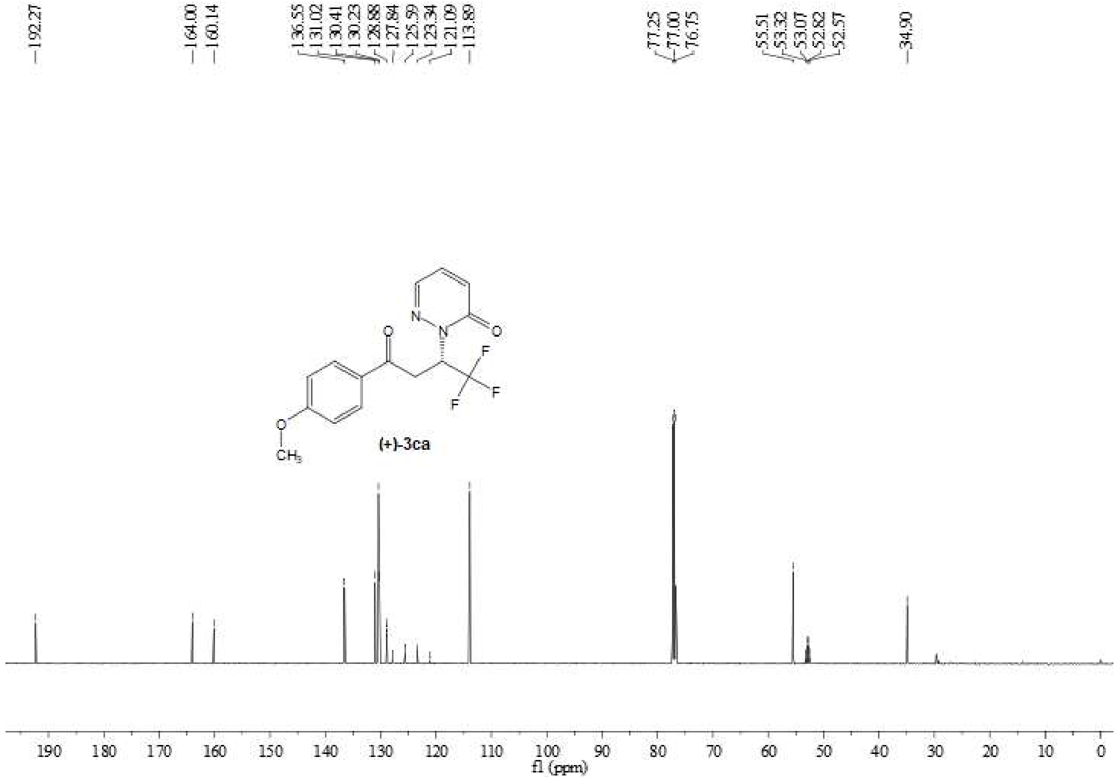

192.27
164.00
160.14
136.55
131.02
130.41
130.23
128.88
127.84
125.59
123.34
121.09
113.89
77.25
77.00
76.75
55.51
53.32
53.07
52.82
52.57
34.90
(+)-3ca
190 180 170 160 150 140 130 120 110 100 90 80 70 60 50 40 30 20 10 0
f1 (ppm)

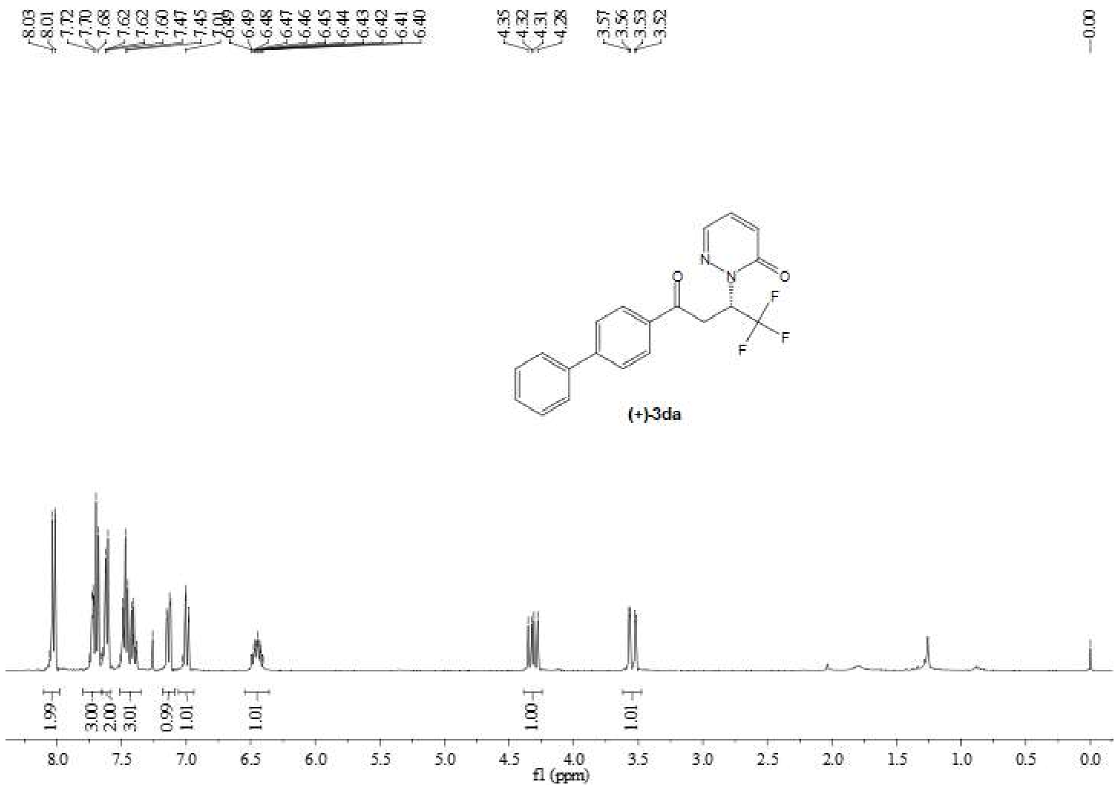
8.03
8.01
7.72
7.70
7.68
7.62
7.62
7.60
7.47
7.45
7.01
6.49
6.48
6.47
6.46
6.45
6.44
6.43
6.42
6.41
6.40
4.35
4.32
4.31
4.28
3.57
3.56
3.53
3.52
0.00
(+)-3da
1.99
3.00
2.00
3.01
0.99
1.01
1.01
1.00
1.01
fl (ppm)

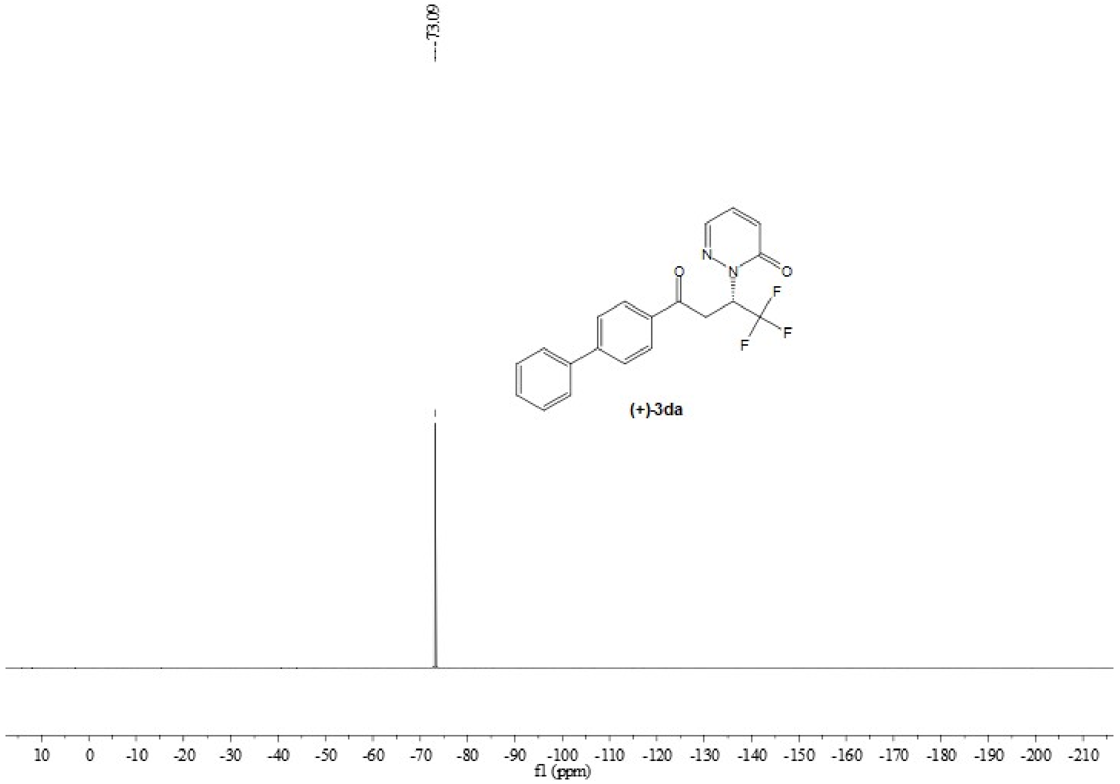
-73.09
(+)-3da
fl (ppm)

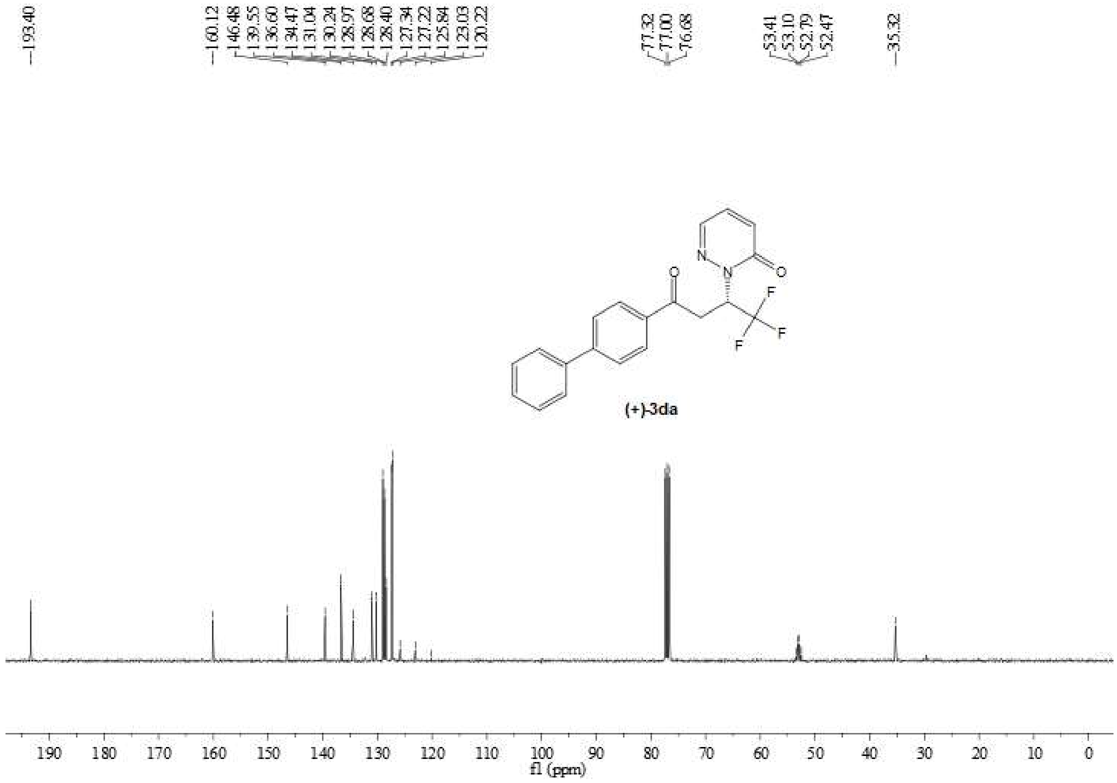
193.40
160.12
146.48
139.55
136.60
134.47
131.04
130.24
128.97
128.68
128.40
127.34
127.22
125.84
123.03
120.22
77.32
77.00
76.68
53.41
53.10
52.79
52.47
35.32
(+)-3da
190 180 170 160 150 140 130 120 110 100 90 80 70 60 50 40 30 20 10 0
f1 (ppm)

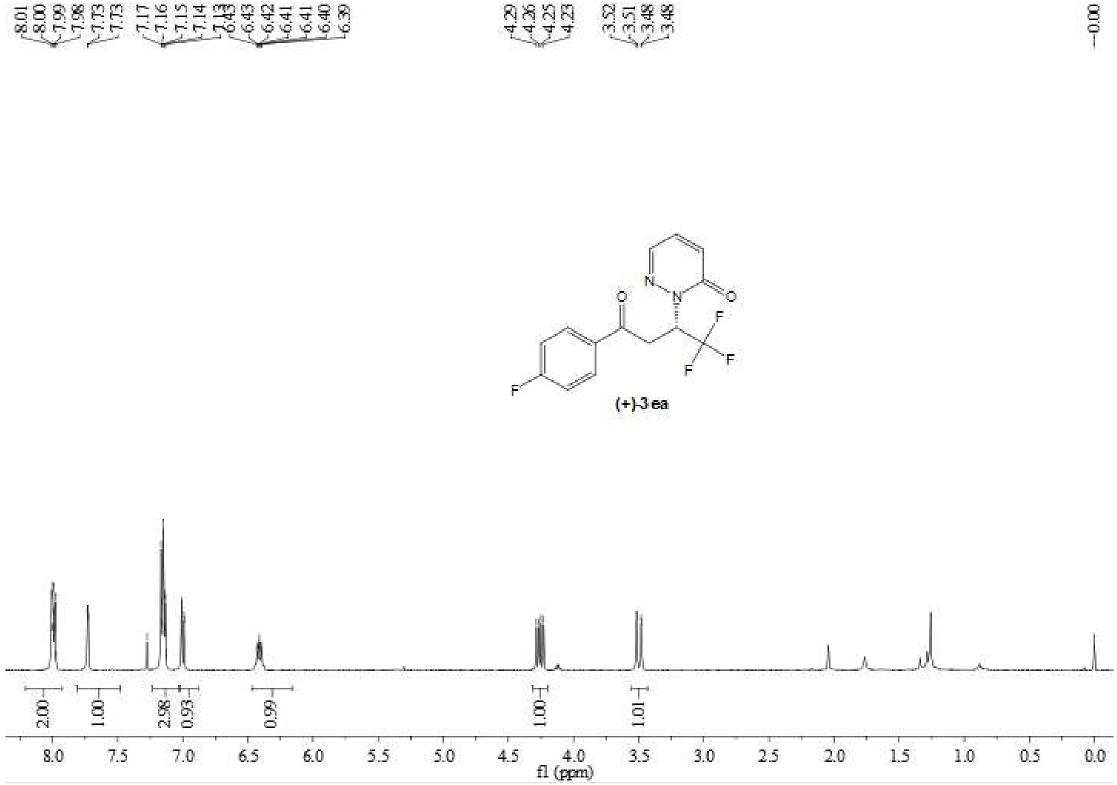
8.01
8.00
7.99
7.98
7.73
7.73
7.17
7.16
7.15
7.14
7.13
6.43
6.43
6.42
6.41
6.41
6.40
6.39
4.29
4.26
4.25
4.23
3.52
3.51
3.48
3.48
0.00
(+)-3 ea
2.00
1.00
2.98
0.93
0.99
1.00
1.01
8.0 7.5 7.0 6.5 6.0 5.5 5.0 4.5 4.0 3.5 3.0 2.5 2.0 1.5 1.0 0.5 0.0
f1 (ppm)

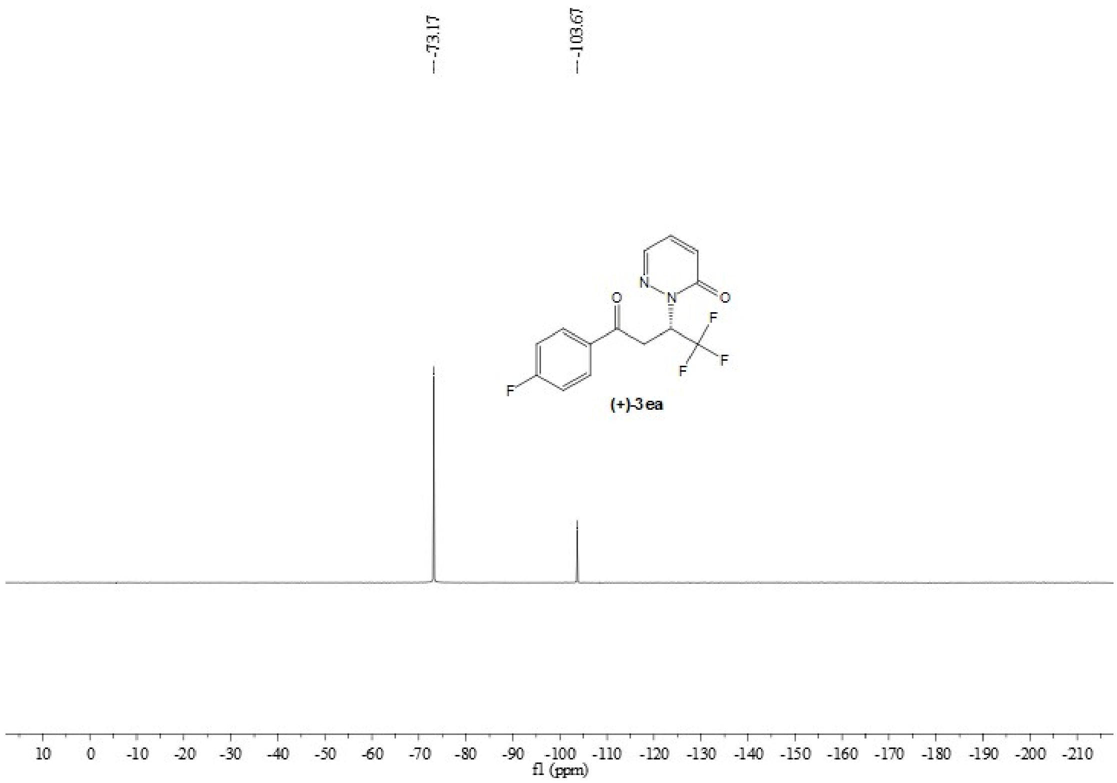
-73.17
-103.67
(+)-3ea
fl (ppm)

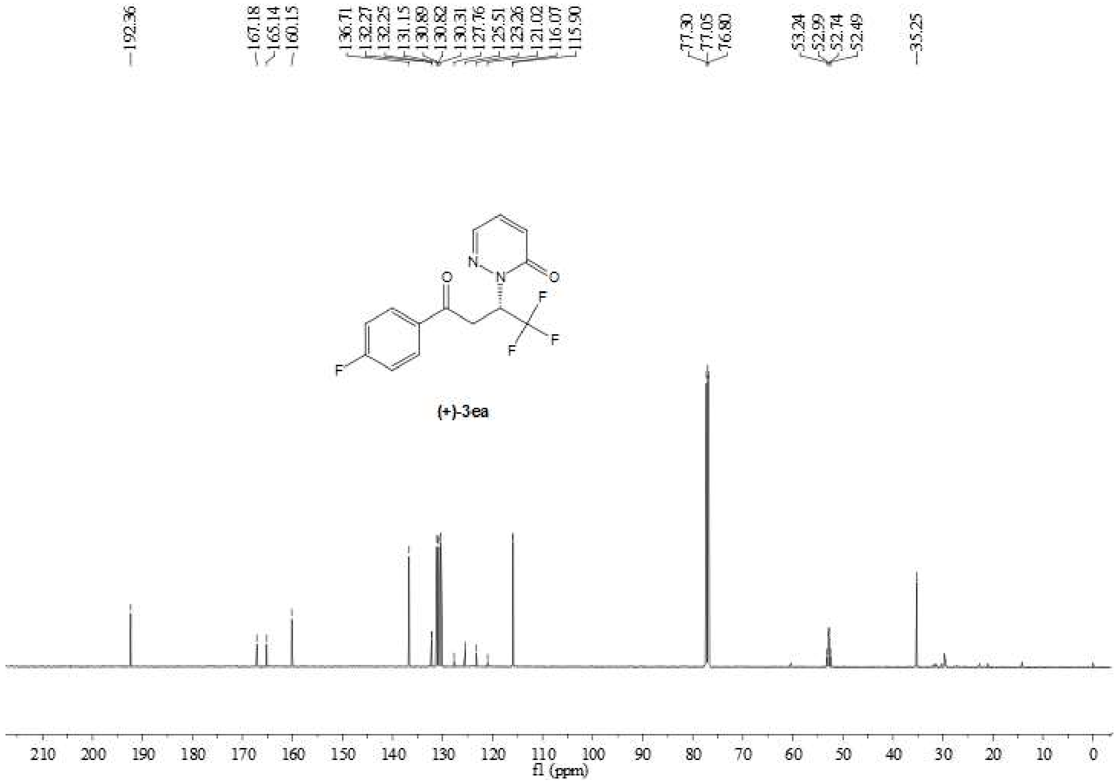
192.36
167.18
165.14
160.15
136.71
132.27
132.25
131.15
130.89
130.82
130.31
127.76
125.51
123.26
121.02
116.07
115.90
77.30
77.05
76.80
53.24
52.99
52.74
52.49
35.25
(+)-3ea
fl (ppm)

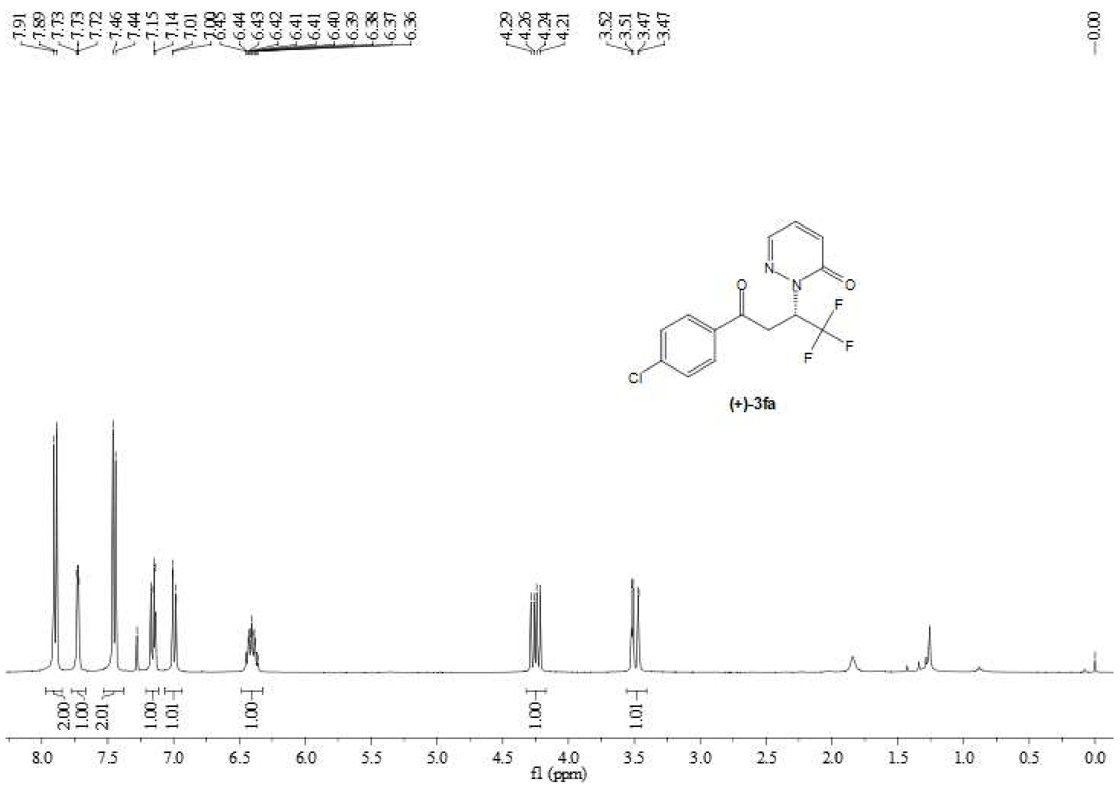
7.91
7.89
7.73
7.73
7.72
7.46
7.44
7.15
7.14
7.01
7.00
6.45
6.44
6.43
6.42
6.41
6.41
6.40
6.39
6.38
6.37
6.36
4.29
4.26
4.24
4.21
3.52
3.51
3.47
3.47
0.00
(+)-3fa
2.00
1.00
2.01
1.00
1.01
1.00
1.00
1.01
f1 (ppm)

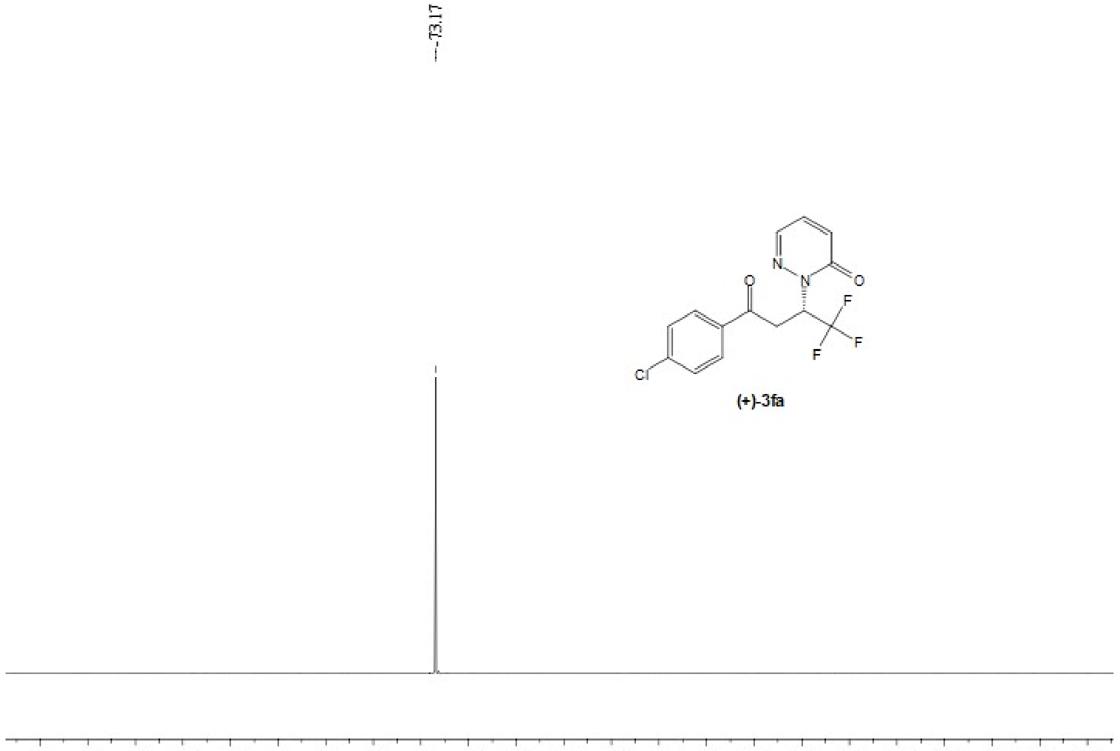
-73.17
(+)-3fa
f1 (ppm)

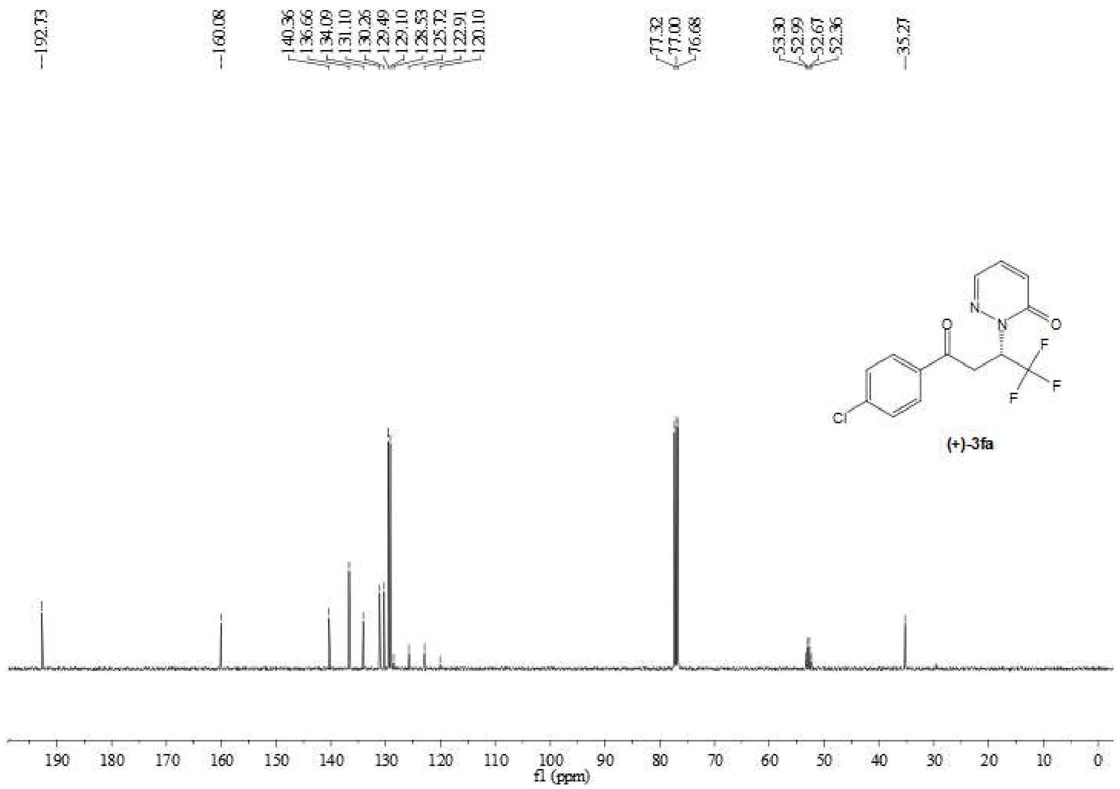
192.73
160.08
140.36
136.66
134.09
131.10
130.26
129.49
129.10
128.53
125.72
122.91
120.10
77.32
77.00
76.68
53.30
52.99
52.67
52.36
35.27
(+)-3fa
190 180 170 160 150 140 130 120 110 100 90 80 70 60 50 40 30 20 10 0
f1 (ppm)

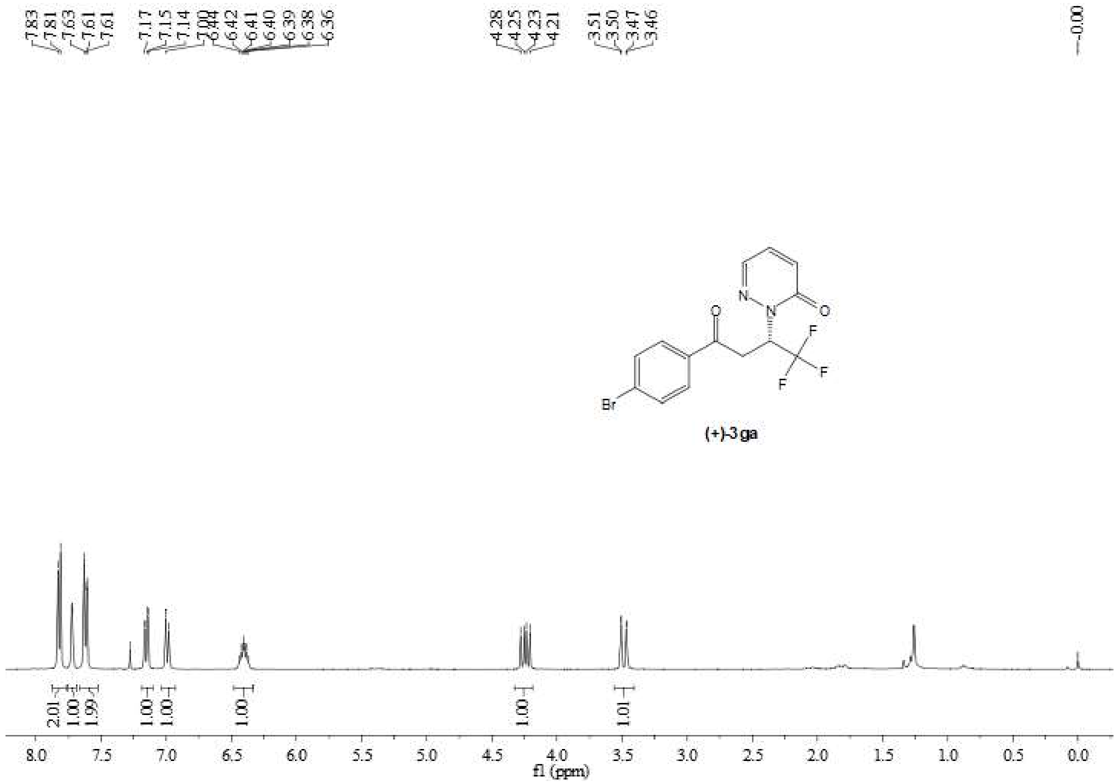
7.83
7.81
7.63
7.61
7.61
7.17
7.15
7.14
7.00
6.99
6.42
6.41
6.40
6.39
6.38
6.36
4.28
4.25
4.23
4.21
3.51
3.50
3.47
3.46
0.00
(+)-3ga
2.01
1.00
1.99
1.00
1.00
1.00
1.00
1.01
8.0 7.5 7.0 6.5 6.0 5.5 5.0 4.5 4.0 3.5 3.0 2.5 2.0 1.5 1.0 0.5 0.0
f1 (ppm)

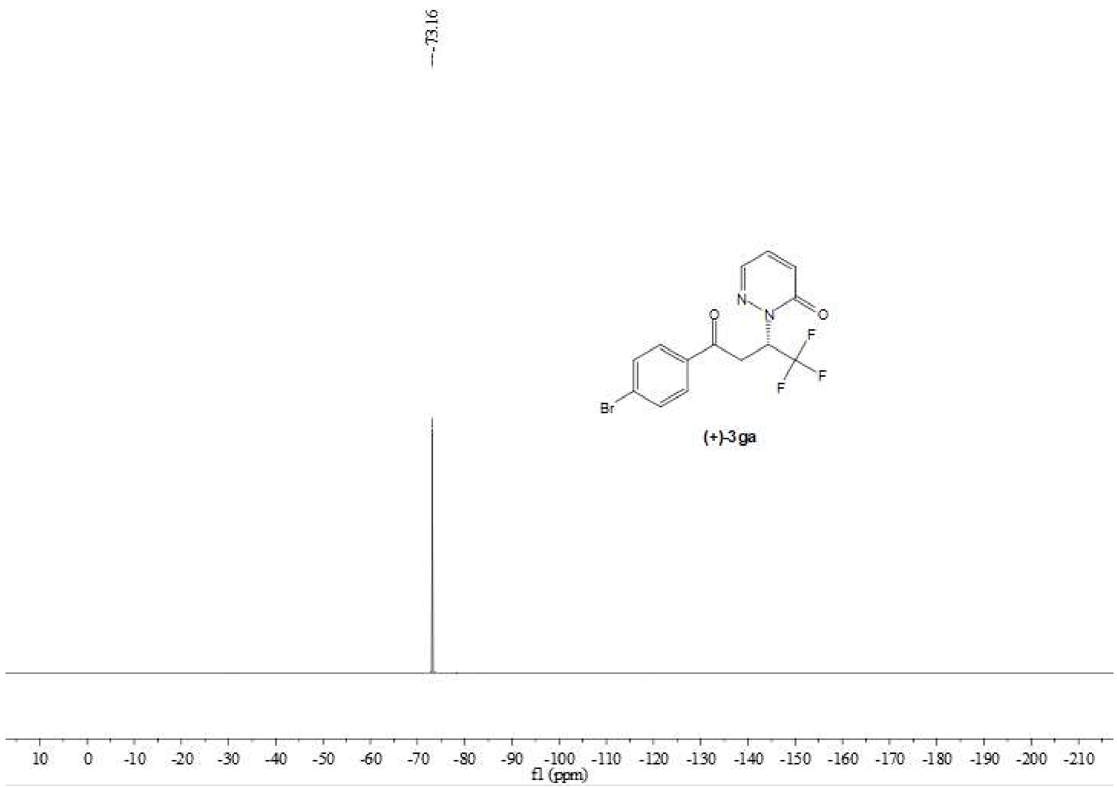
-73.16
(+)-3ga
10 0 -10 -20 -30 -40 -50 -60 -70 -80 -90 -100 -110 -120 -130 -140 -150 -160 -170 -180 -190 -200 -210
f1 (ppm)

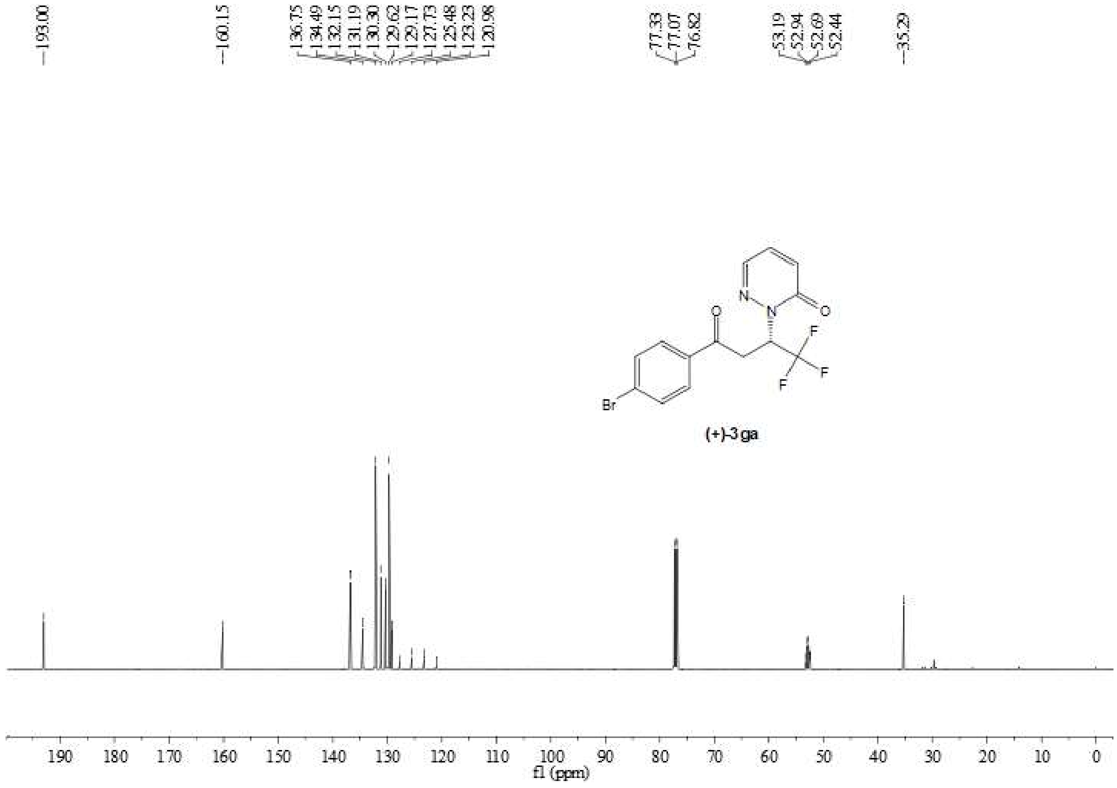
193.00
160.15
136.75
134.49
132.15
131.19
130.30
129.62
129.17
127.73
125.48
123.23
120.98
77.33
77.07
76.82
53.19
52.94
52.69
52.44
35.29
(+)-3ga
190 180 170 160 150 140 130 120 110 100 90 80 70 60 50 40 30 20 10 0
f1 (ppm)

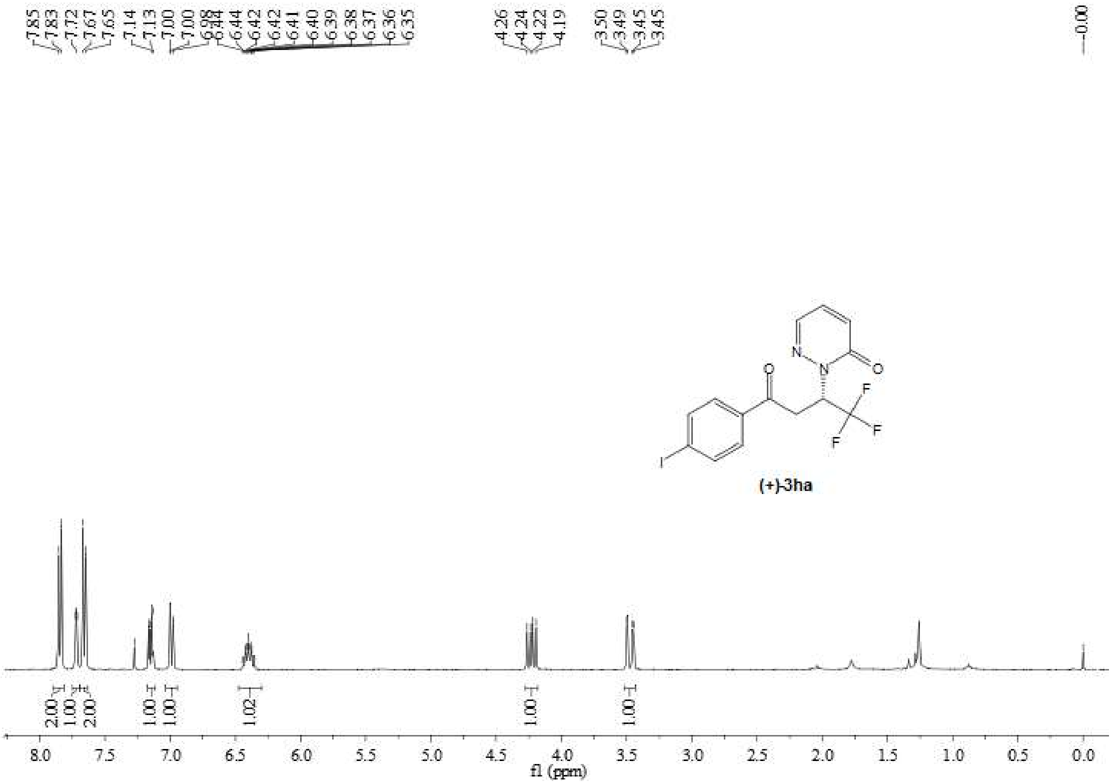
7.85
7.83
7.72
7.67
7.65
7.14
7.13
7.00
7.00
6.98
6.44
6.44
6.42
6.41
6.40
6.39
6.38
6.37
6.36
6.35
4.26
4.24
4.22
4.19
3.50
3.49
3.45
3.45
0.00
(+)-3ha
f1 (ppm)

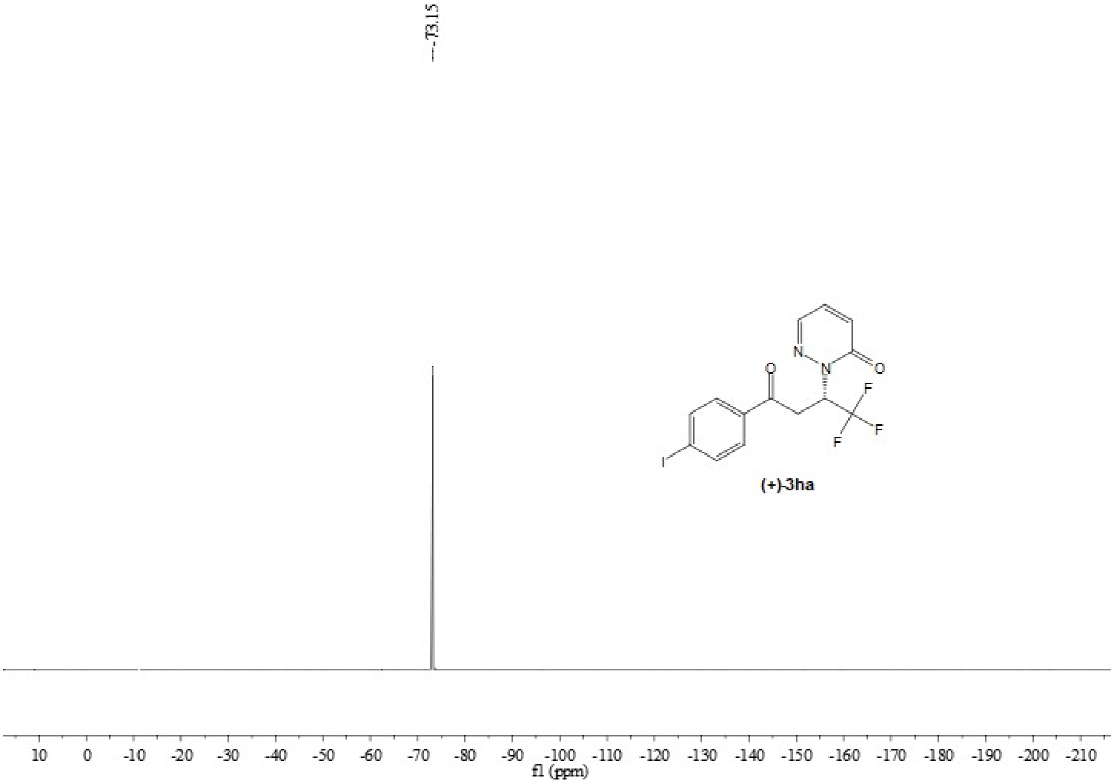
-73.15
(+)-3ha
f1 (ppm)

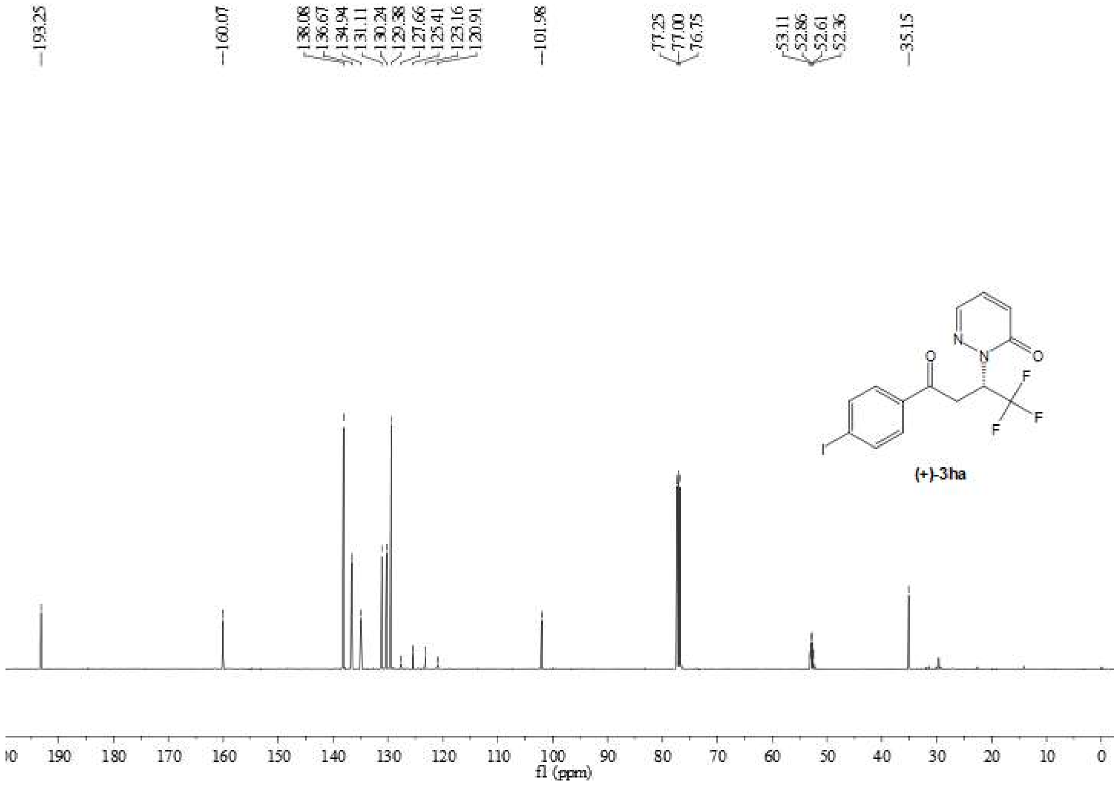
193.25
160.07
138.08
136.67
134.94
131.11
130.24
129.38
127.66
125.41
123.16
120.91
101.98
77.25
77.00
76.75
53.11
52.86
52.61
52.36
35.15
(+)-3ha
fl (ppm)

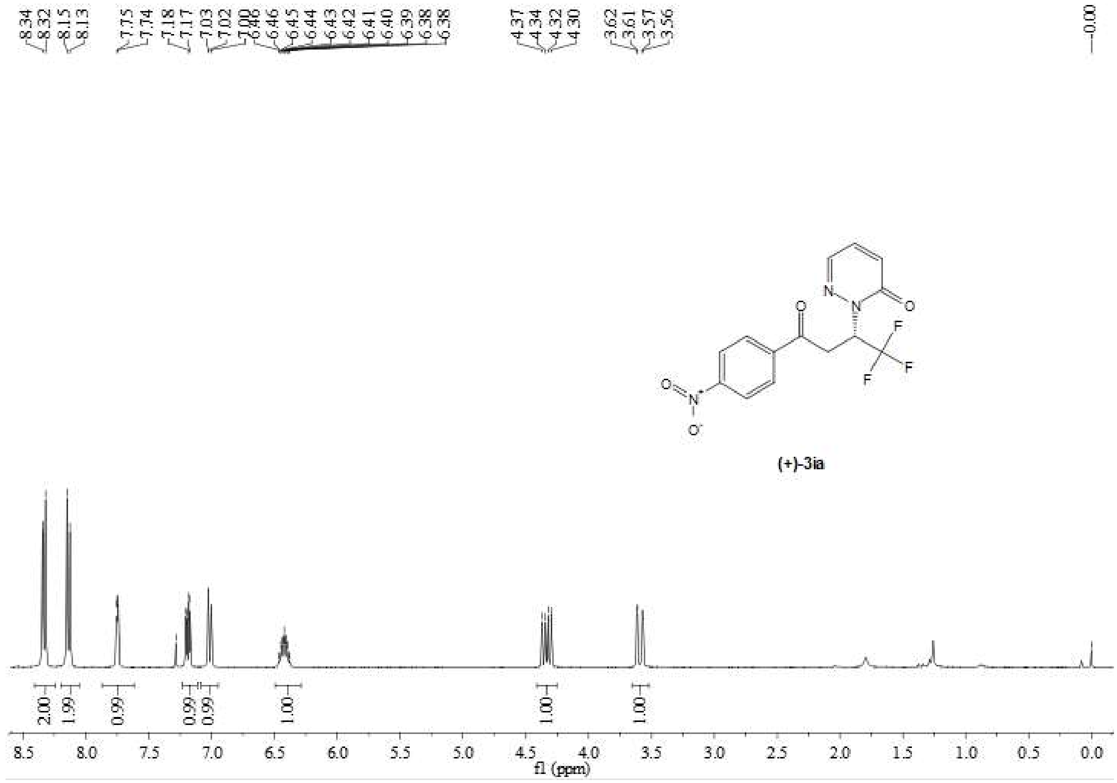
8.34
8.32
8.15
8.13
7.75
7.74
7.18
7.17
7.03
7.02
7.00
6.46
6.45
6.44
6.43
6.42
6.41
6.40
6.39
6.38
6.38
4.37
4.34
4.32
4.30
3.62
3.61
3.57
3.56
0.00
(+)-3ia
2.00
1.99
0.99
0.99
0.99
1.00
1.00
1.00
fl (ppm)

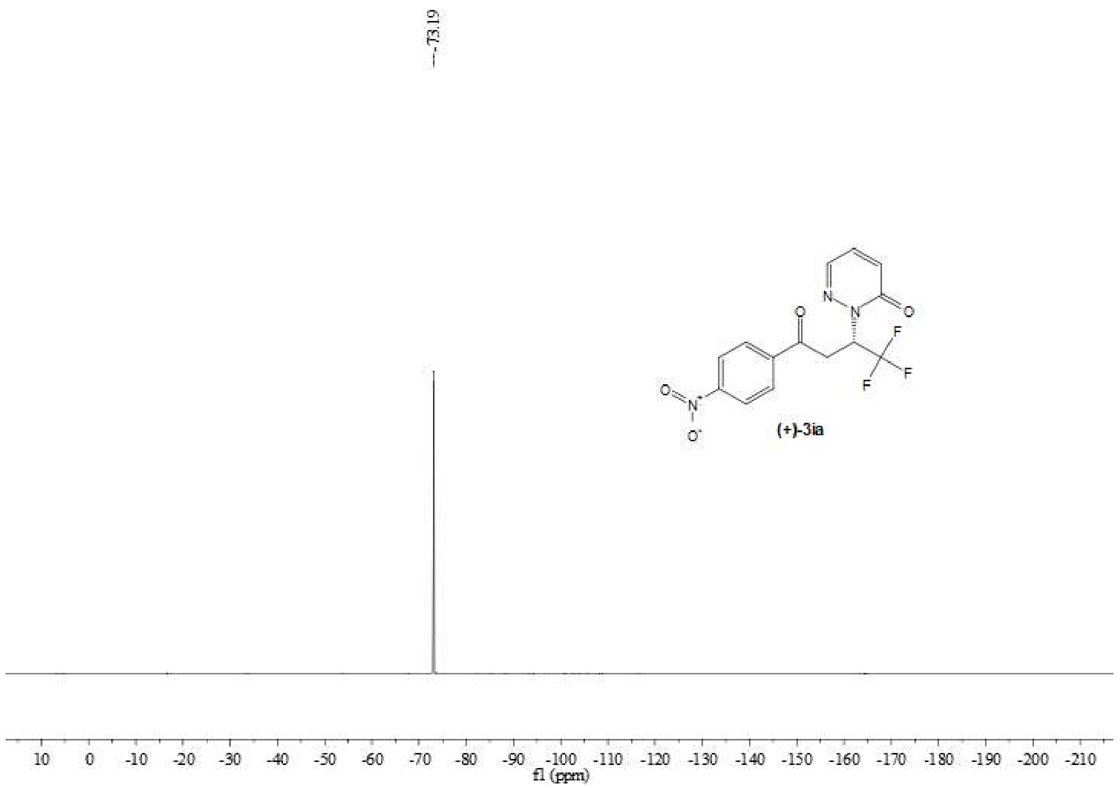
-73.19
(+)-3ia
10 0 -10 -20 -30 -40 -50 -60 -70 -80 -90 -100 -110 -120 -130 -140 -150 -160 -170 -180 -190 -200 -210
f1 (ppm)

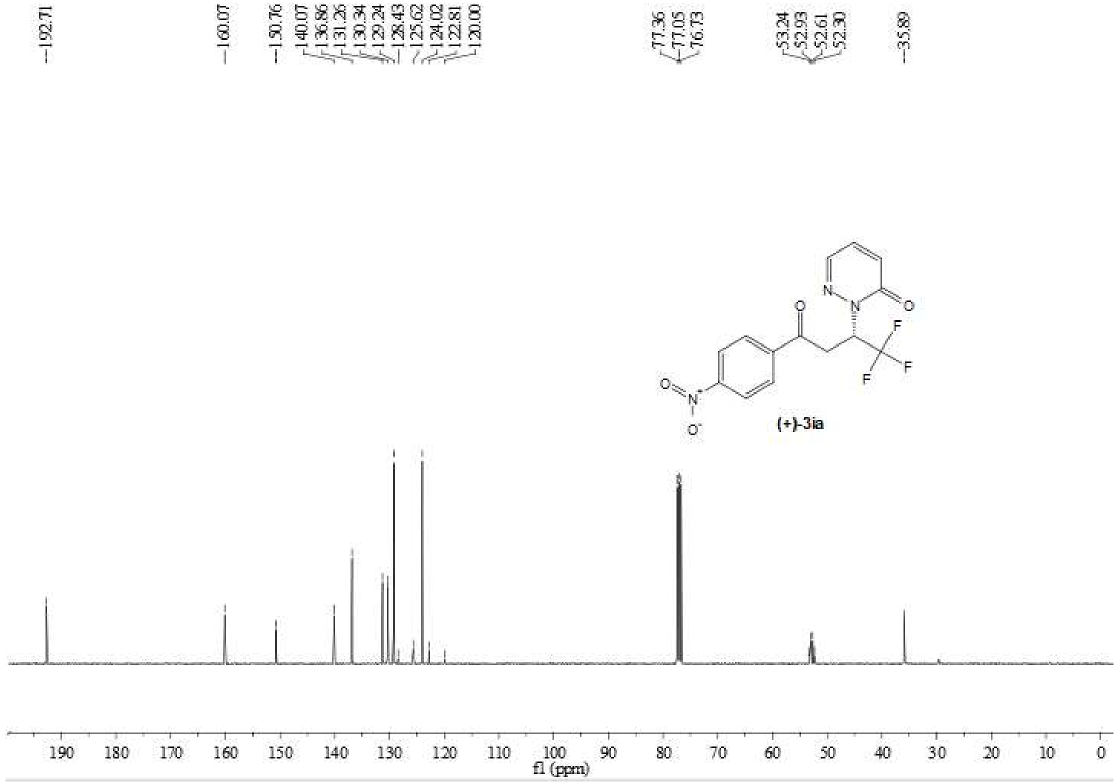
192.71
160.07
150.76
140.07
136.86
131.26
130.34
129.24
128.43
125.62
124.02
122.81
120.00
77.36
77.05
76.73
53.24
52.93
52.61
52.30
35.89
(+)-3ia
190 180 170 160 150 140 130 120 110 100 90 80 70 60 50 40 30 20 10 0
f1 (ppm)

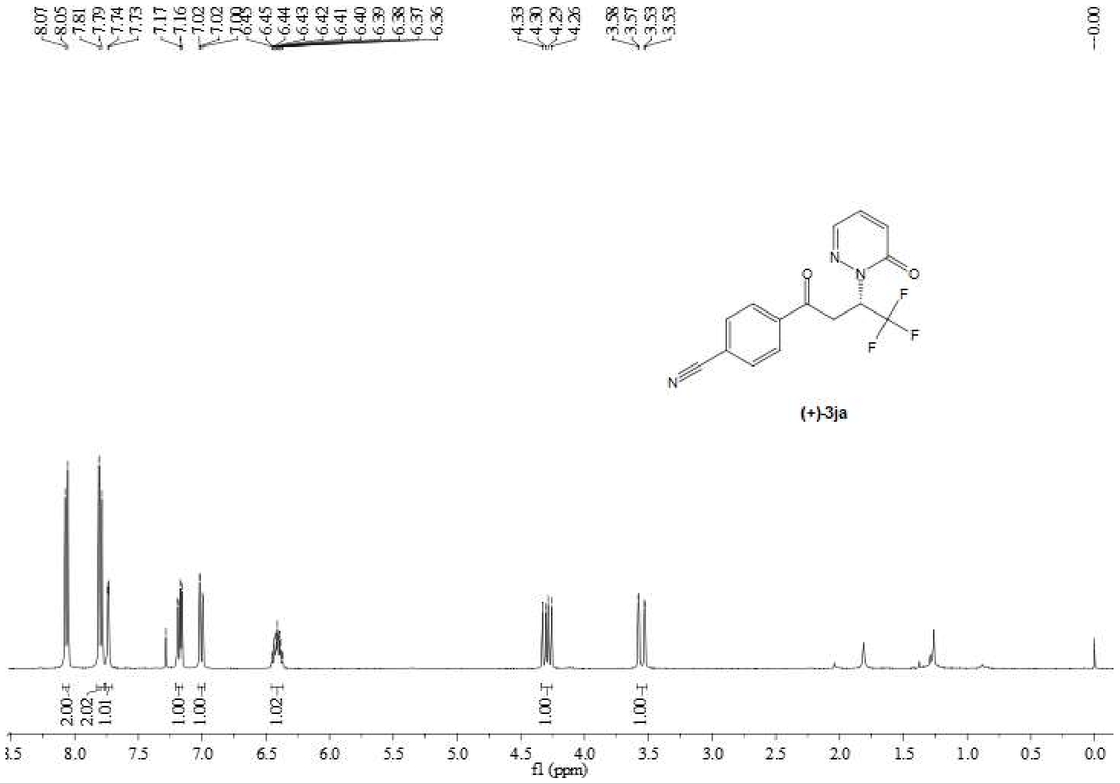

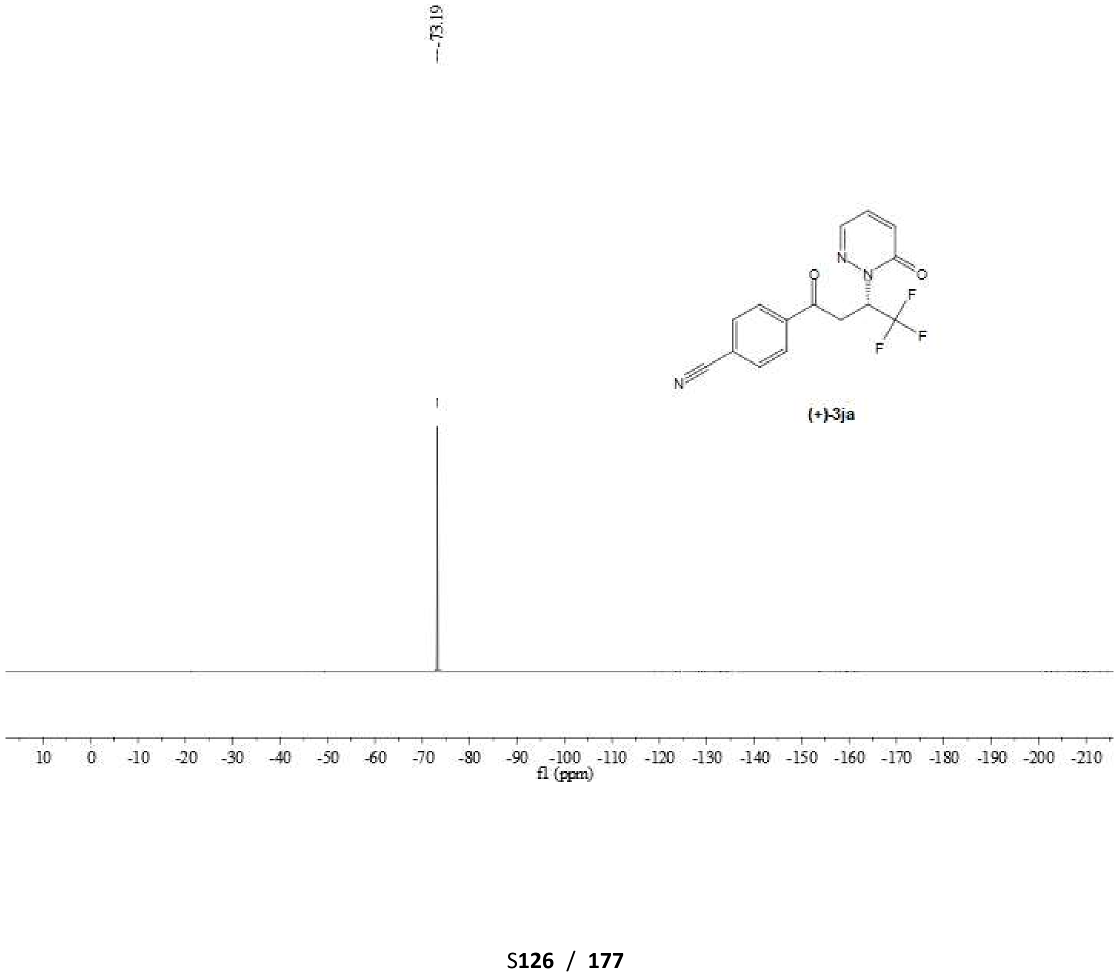

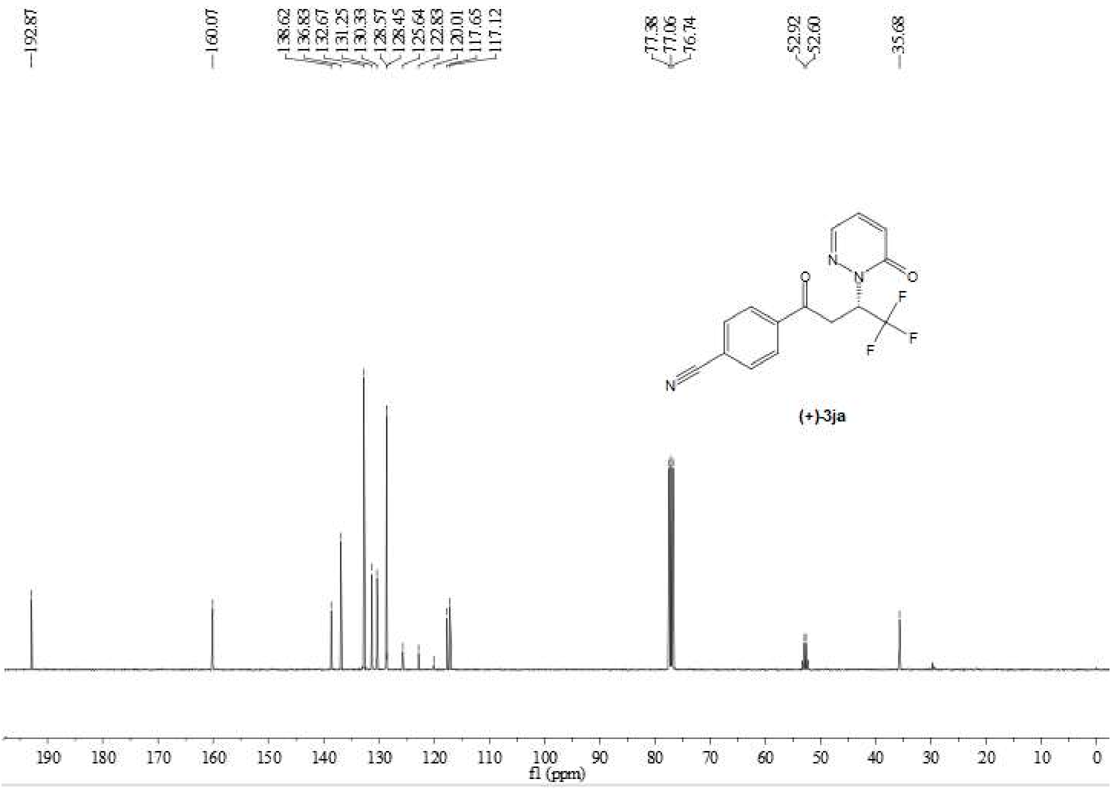
(+)-3ja
f1 (ppm)

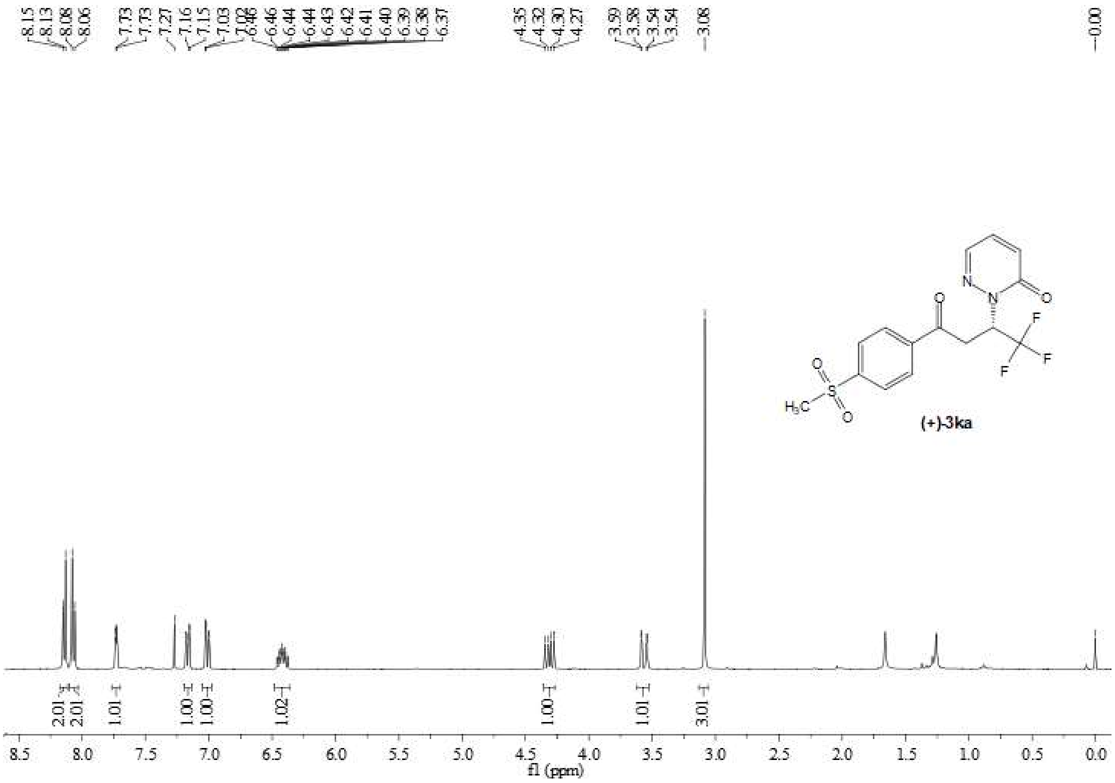
(+)-3ka
f1 (ppm)

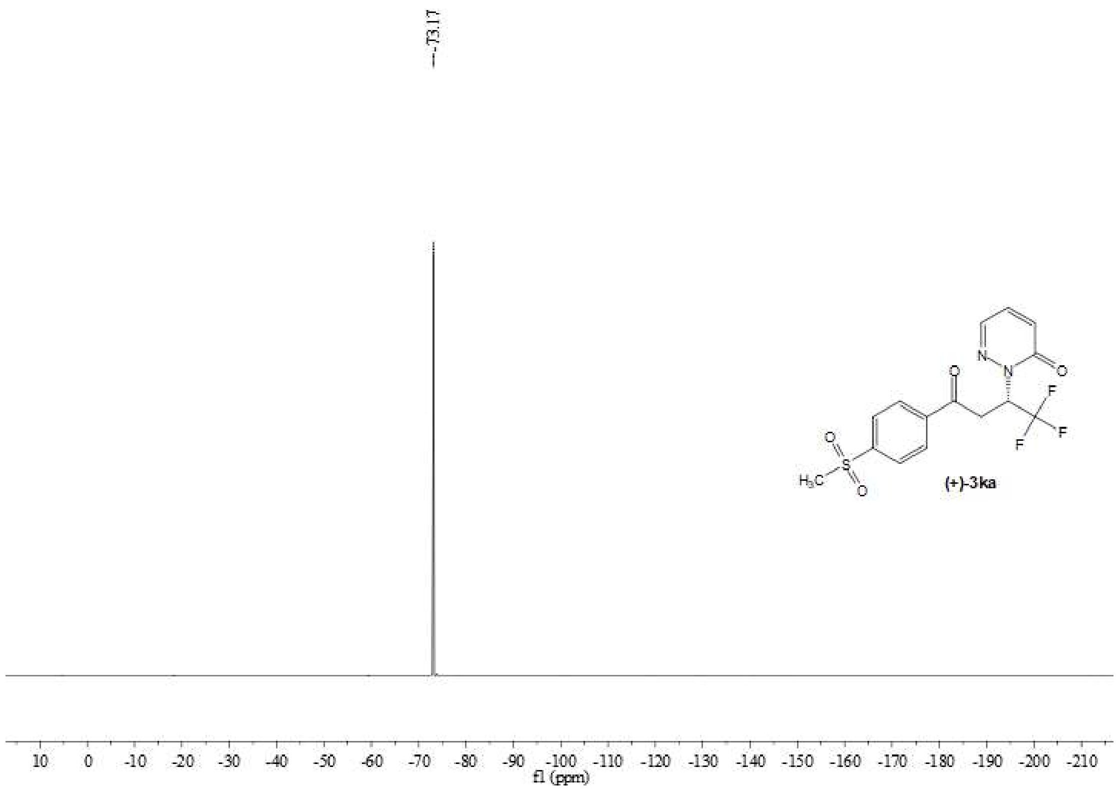
-73.17
(+)-3ka
10 0 -10 -20 -30 -40 -50 -60 -70 -80 -90 -100 -110 -120 -130 -140 -150 -160 -170 -180 -190 -200 -210
f1 (ppm)

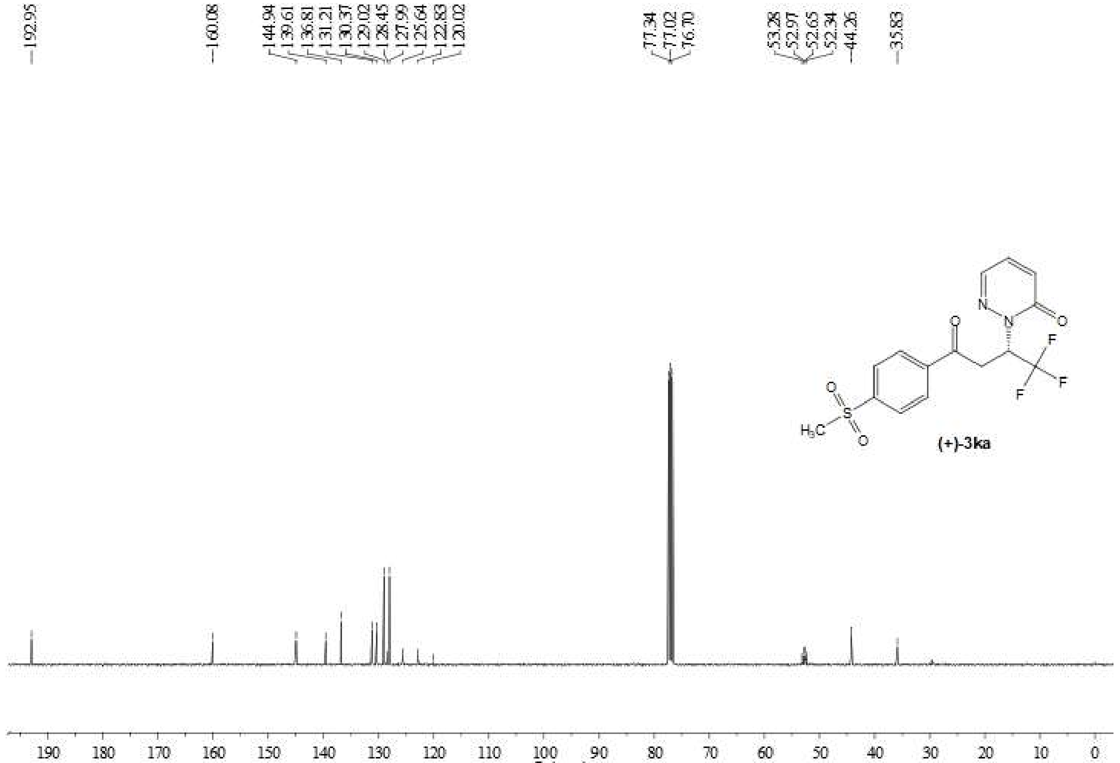
192.95
160.08
144.94
139.61
136.81
131.21
130.37
129.02
128.45
127.99
125.64
122.83
120.02
77.34
77.02
76.70
53.28
52.97
52.65
52.34
44.26
35.83
(+)-3ka
190 180 170 160 150 140 130 120 110 100 90 80 70 60 50 40 30 20 10 0
f1 (ppm)

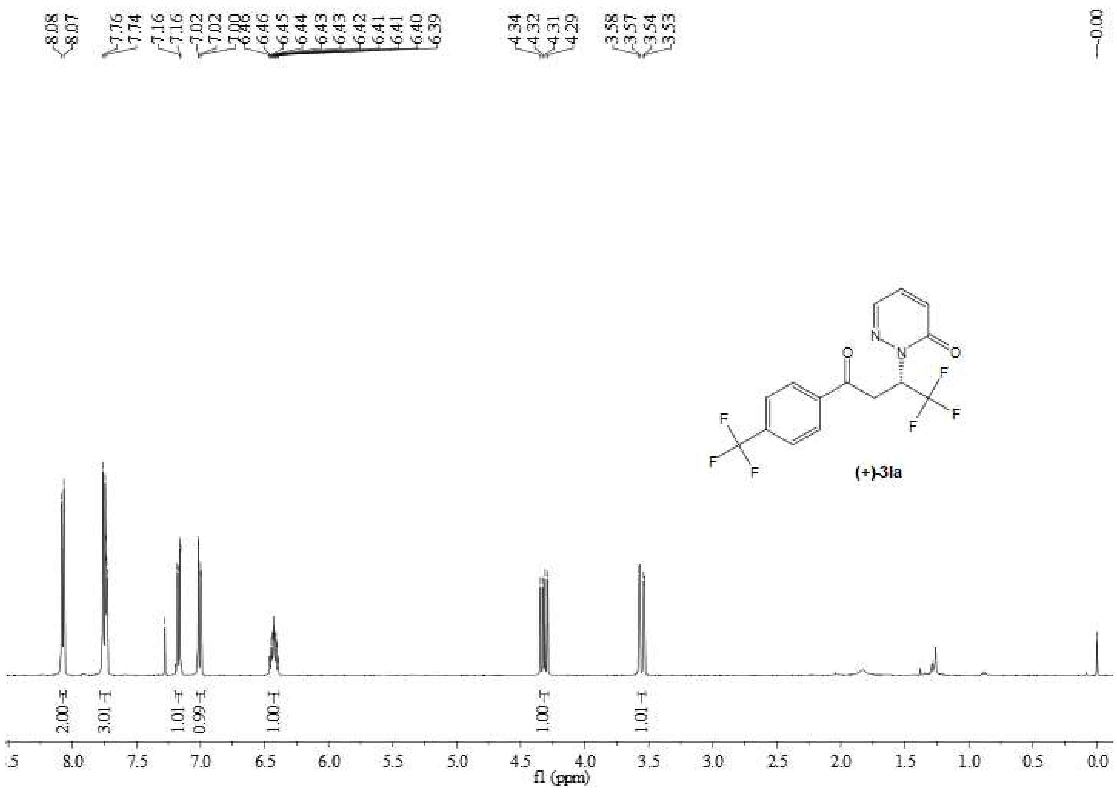
8.08
8.07
7.76
7.74
7.16
7.16
7.02
7.02
7.00
6.46
6.45
6.44
6.43
6.43
6.42
6.41
6.41
6.40
6.39
4.34
4.32
4.31
4.29
3.58
3.57
3.54
3.53
0.00
(+)-3la
2.00
3.01
1.01
0.99
1.00
1.00
1.01
fl (ppm)

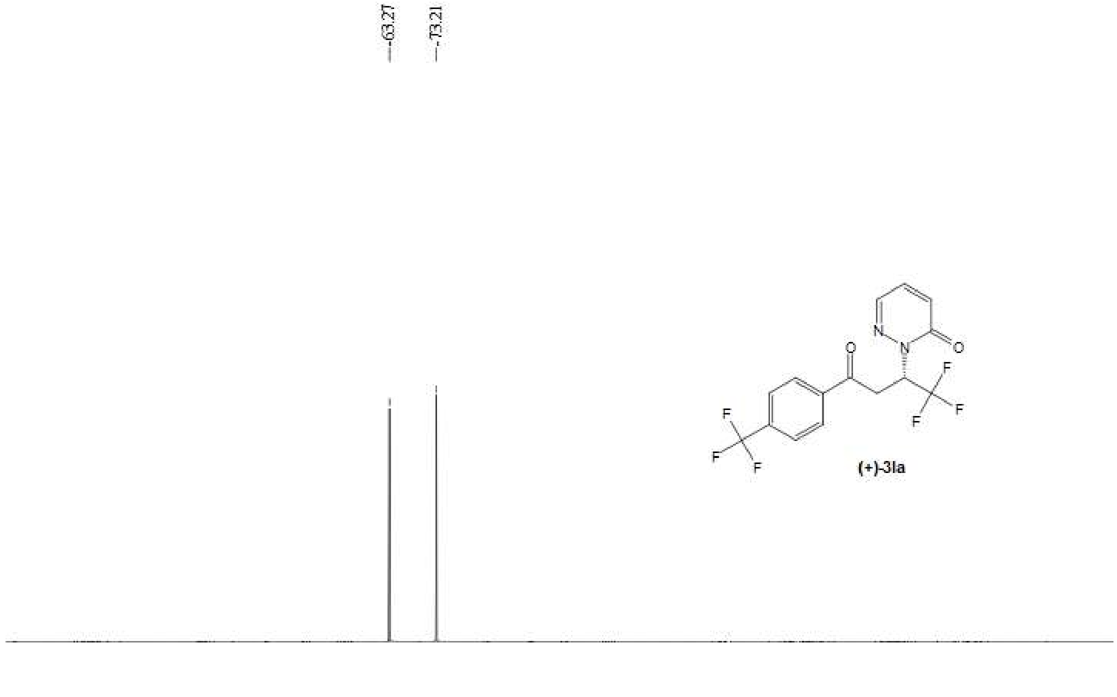
-63.27
-73.21
(+)-3la
fl (ppm)

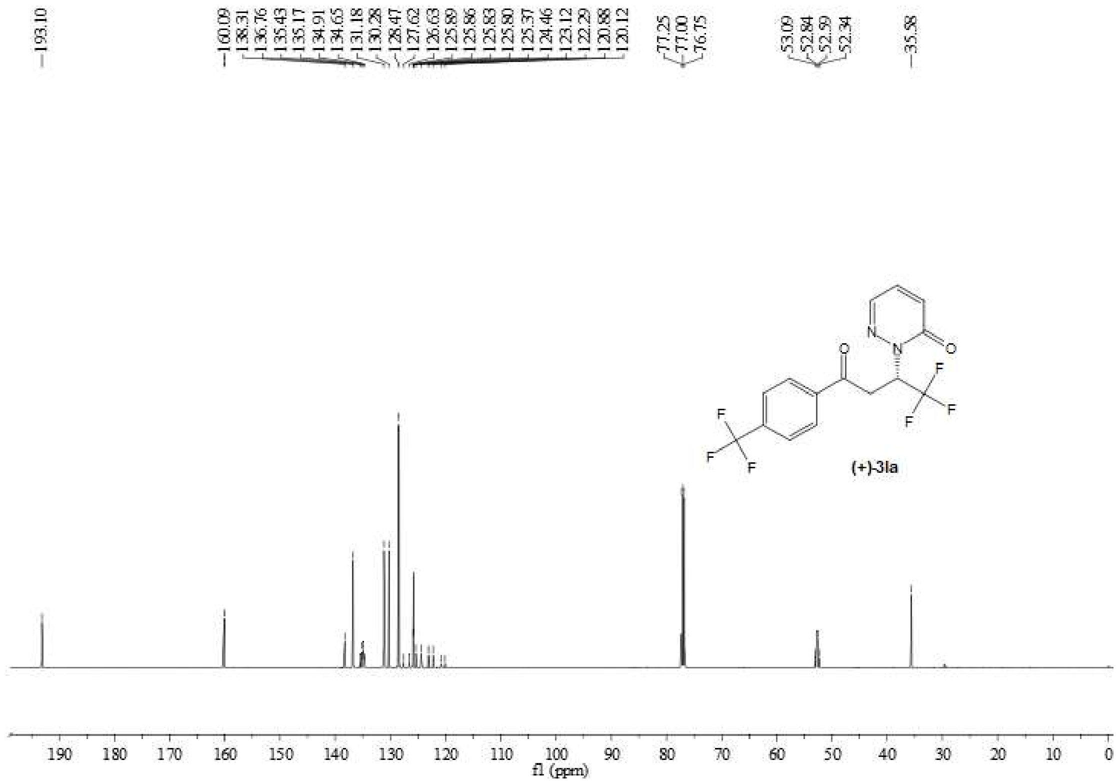
193.10
160.09
138.31
136.76
135.43
135.17
134.91
134.65
131.18
130.28
128.47
127.62
126.63
125.89
125.86
125.83
125.80
125.37
124.46
123.12
122.29
120.88
120.12
77.25
77.00
76.75
53.09
52.84
52.59
52.34
35.58
(+)-3la
f1 (ppm)

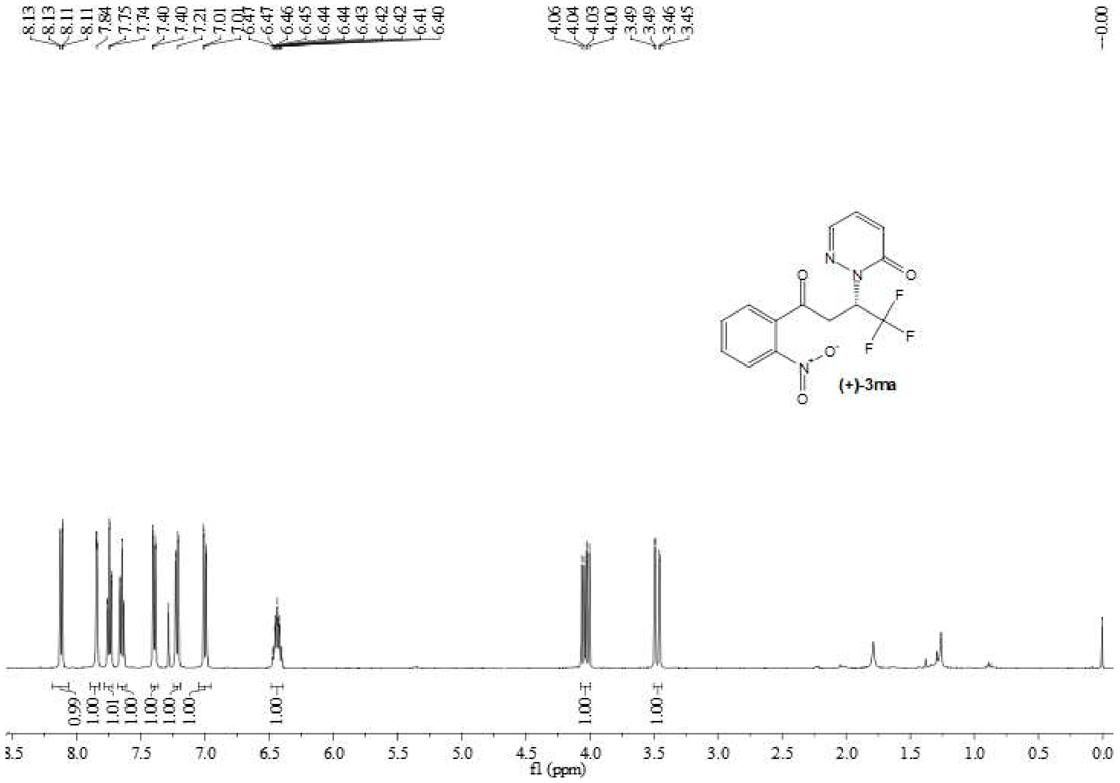
8.13
8.13
8.11
8.11
7.84
7.75
7.74
7.40
7.40
7.21
7.01
7.01
6.47
6.47
6.46
6.45
6.44
6.44
6.43
6.42
6.42
6.41
6.40
4.06
4.04
4.03
4.00
3.49
3.49
3.46
3.45
0.00
(+)-3ma
0.99
1.00
1.01
1.00
1.00
1.00
1.00
1.00
1.00
1.00
f1 (ppm)

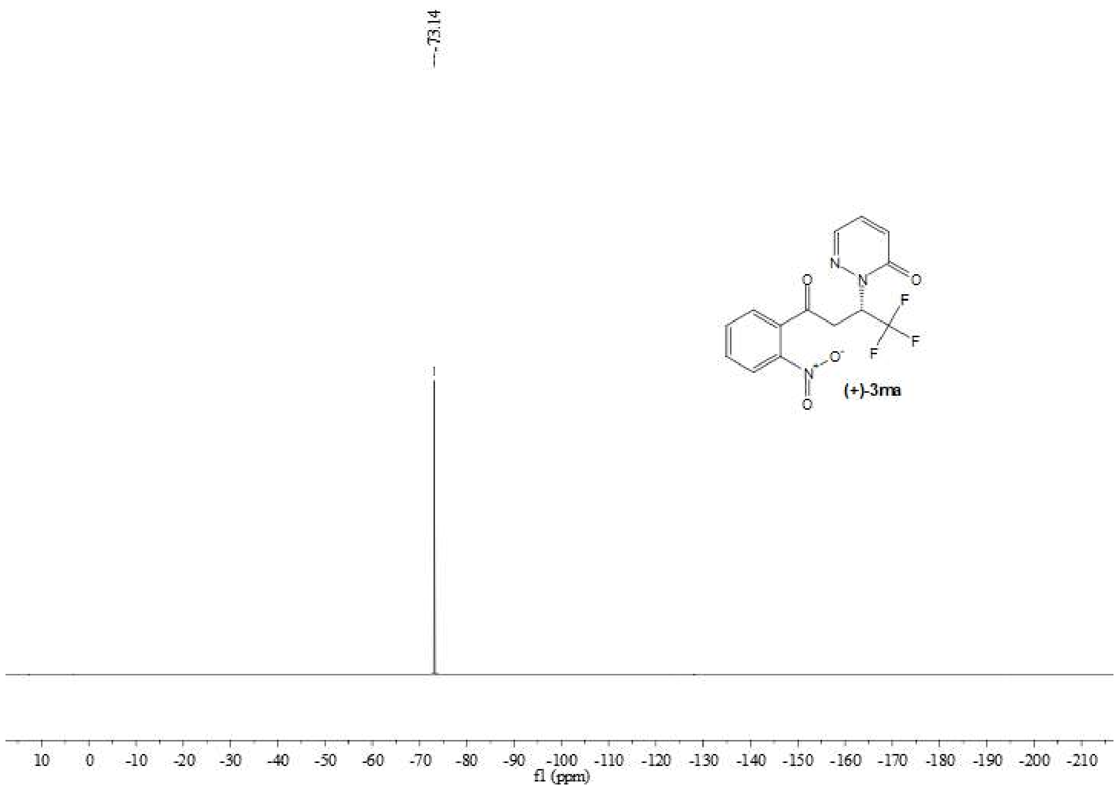
-73.14
(+)-3ma
10 0 -10 -20 -30 -40 -50 -60 -70 -80 -90 -100 -110 -120 -130 -140 -150 -160 -170 -180 -190 -200 -210
f1 (ppm)

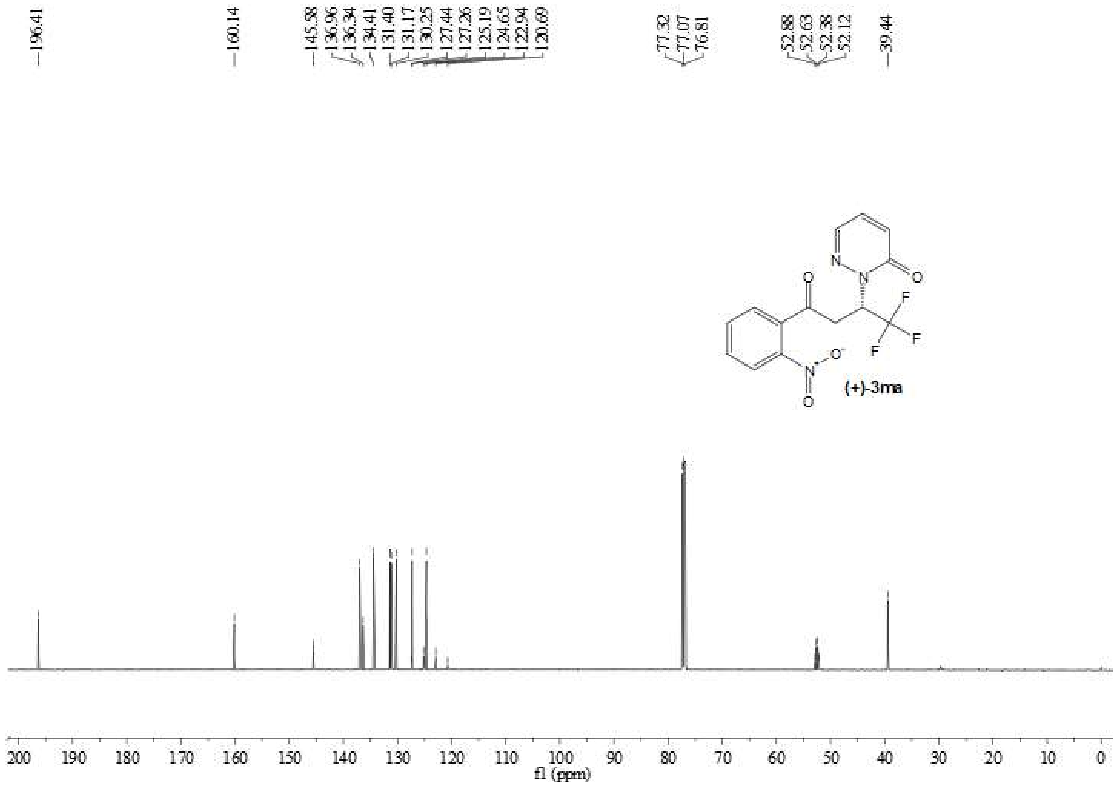
196.41
160.14
145.58 136.96 136.34 134.41 131.40 131.17 130.25 127.44 127.26 125.19 124.65 122.94 120.69
77.32 77.07 76.81
52.88 52.63 52.38 52.12
39.44
(+)-3ma
200 190 180 170 160 150 140 130 120 110 100 90 80 70 60 50 40 30 20 10 0
f1 (ppm)

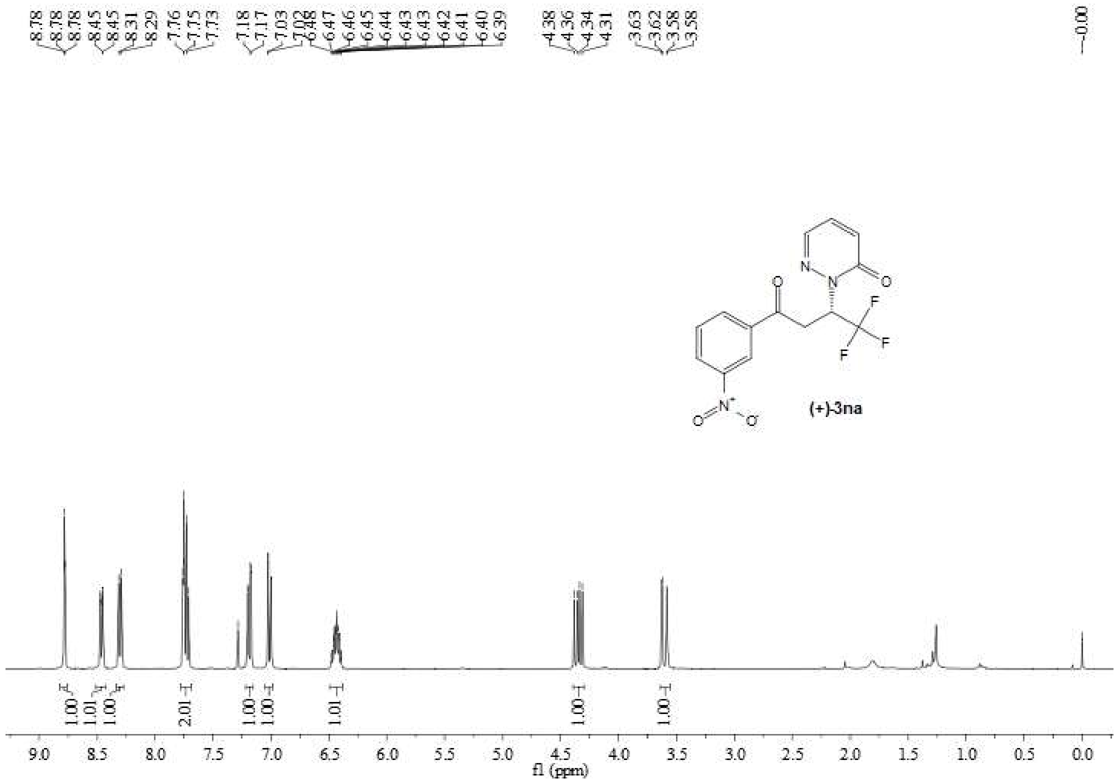
(+)-3na
f1 (ppm)

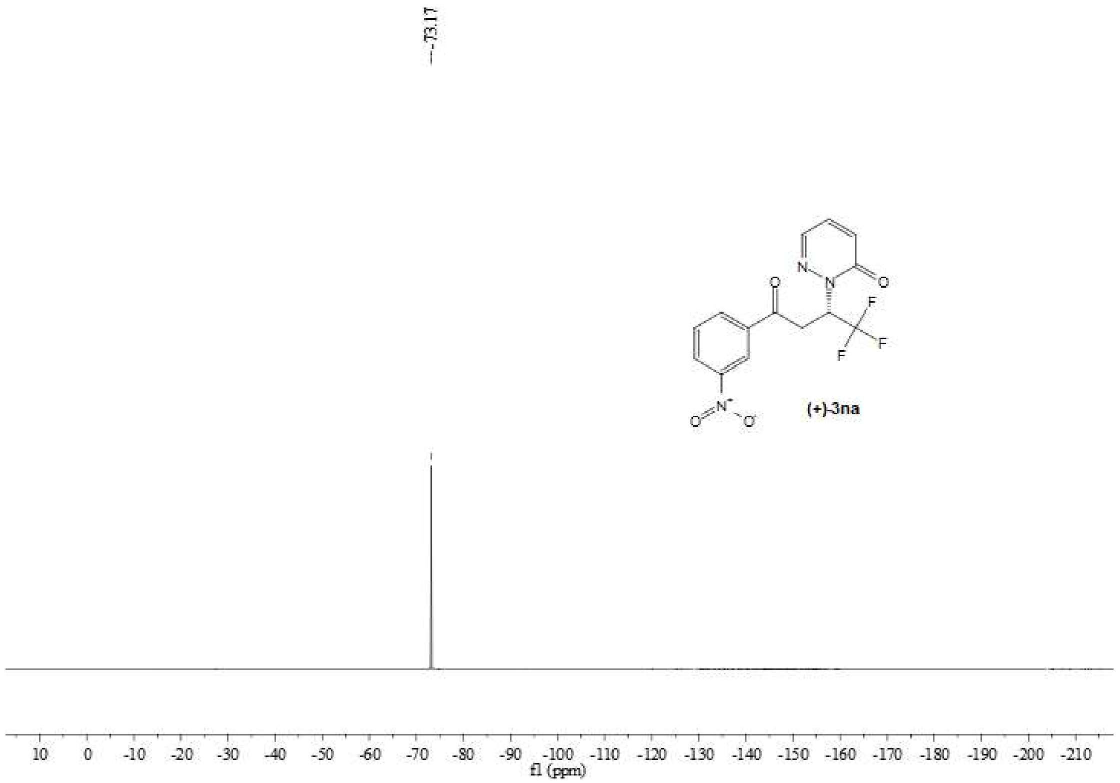
-73.17
(+)-3na
f1 (ppm)

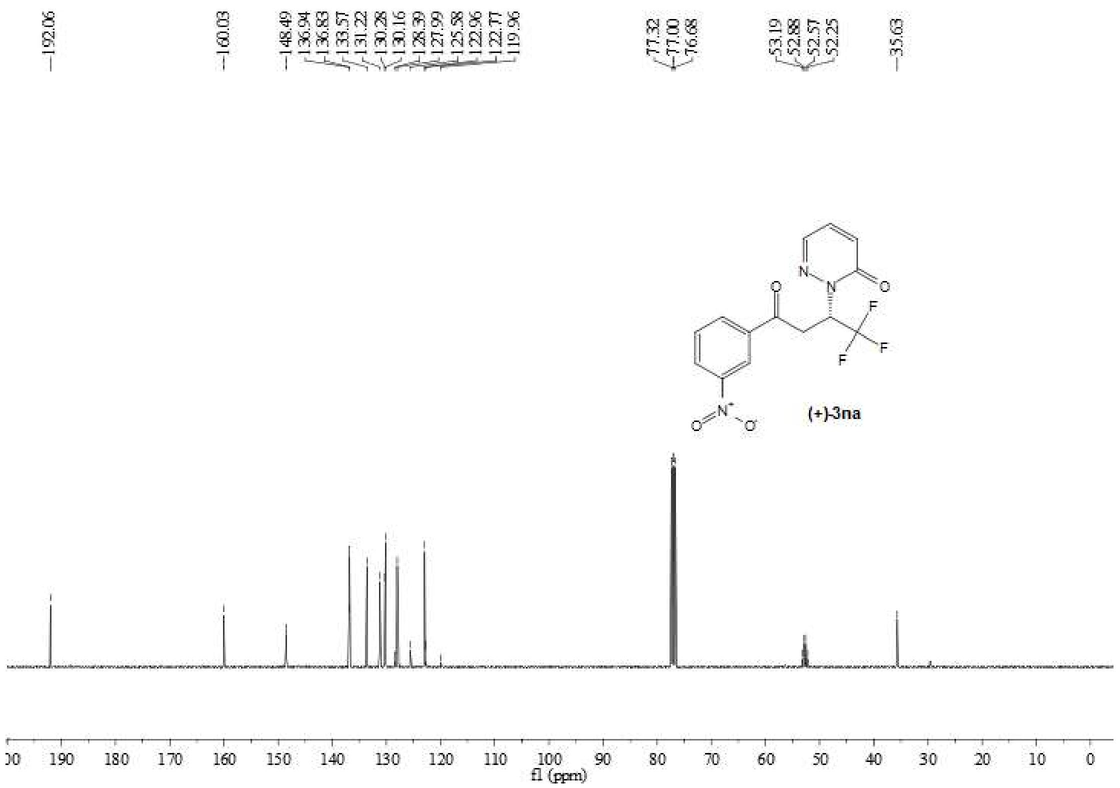
192.06
160.03
148.49
136.94
136.83
133.57
131.22
130.28
130.16
128.39
127.99
125.58
122.96
122.77
119.96
77.32
77.00
76.68
53.19
52.88
52.57
52.25
35.63
(+)-3na
fl (ppm)

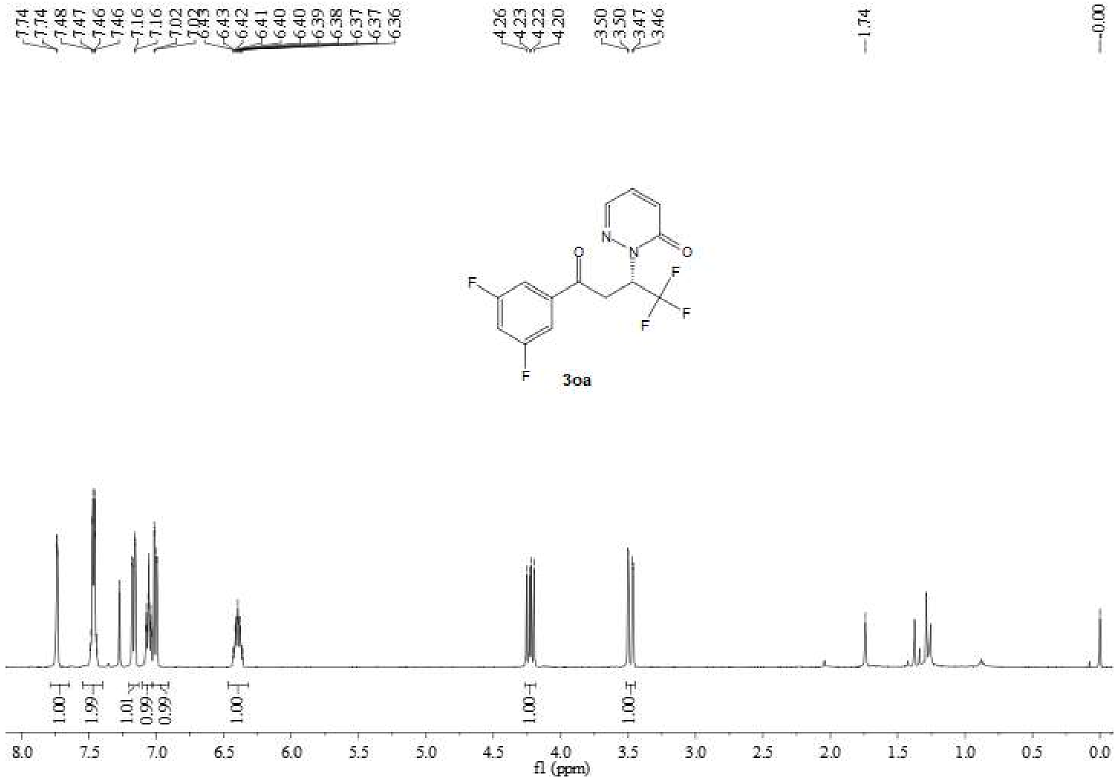
7.74
7.74
7.48
7.47
7.46
7.46
7.16
7.16
7.02
7.02
6.43
6.42
6.41
6.40
6.39
6.38
6.37
6.37
6.36
4.26
4.23
4.22
4.20
3.50
3.50
3.47
3.46
1.74
0.00
3oa
1.00
1.99
1.01
0.99
0.99
1.00
1.00
1.00
fl (ppm)

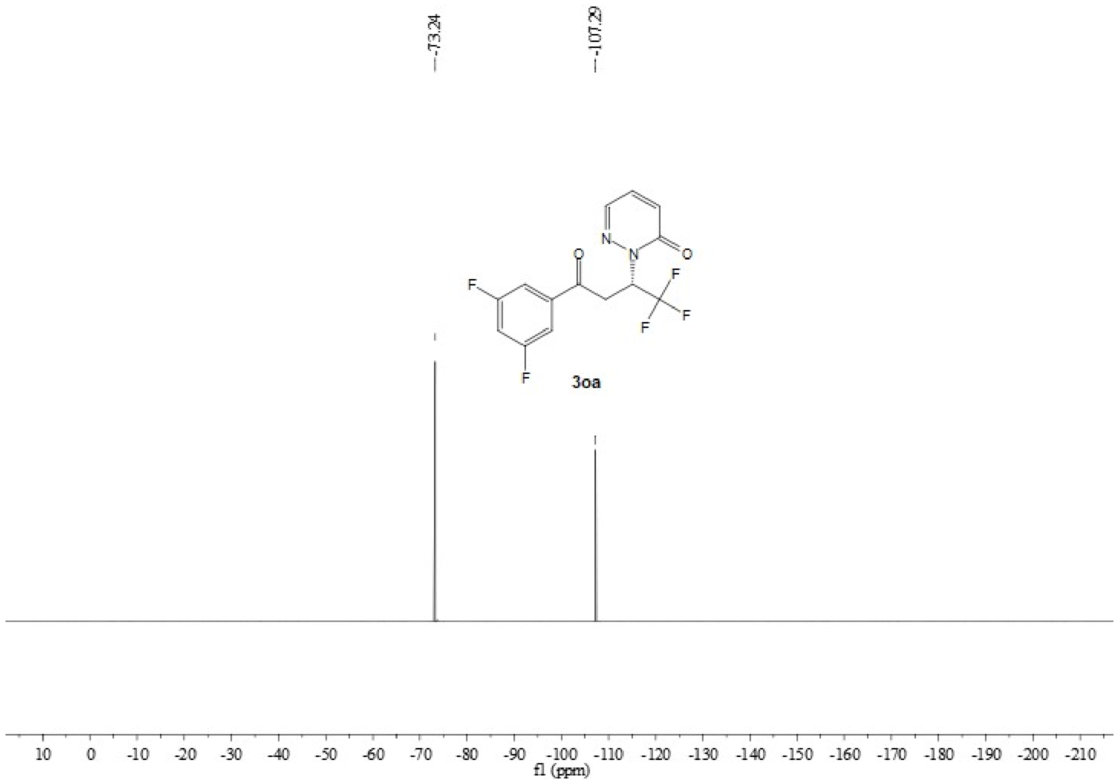

-73.24
-107.29
F
F
O
N
N
O
F
F
F
3oa
10 0 -10 -20 -30 -40 -50 -60 -70 -80 -90 -100 -110 -120 -130 -140 -150 -160 -170 -180 -190 -200 -210
f1 (ppm)

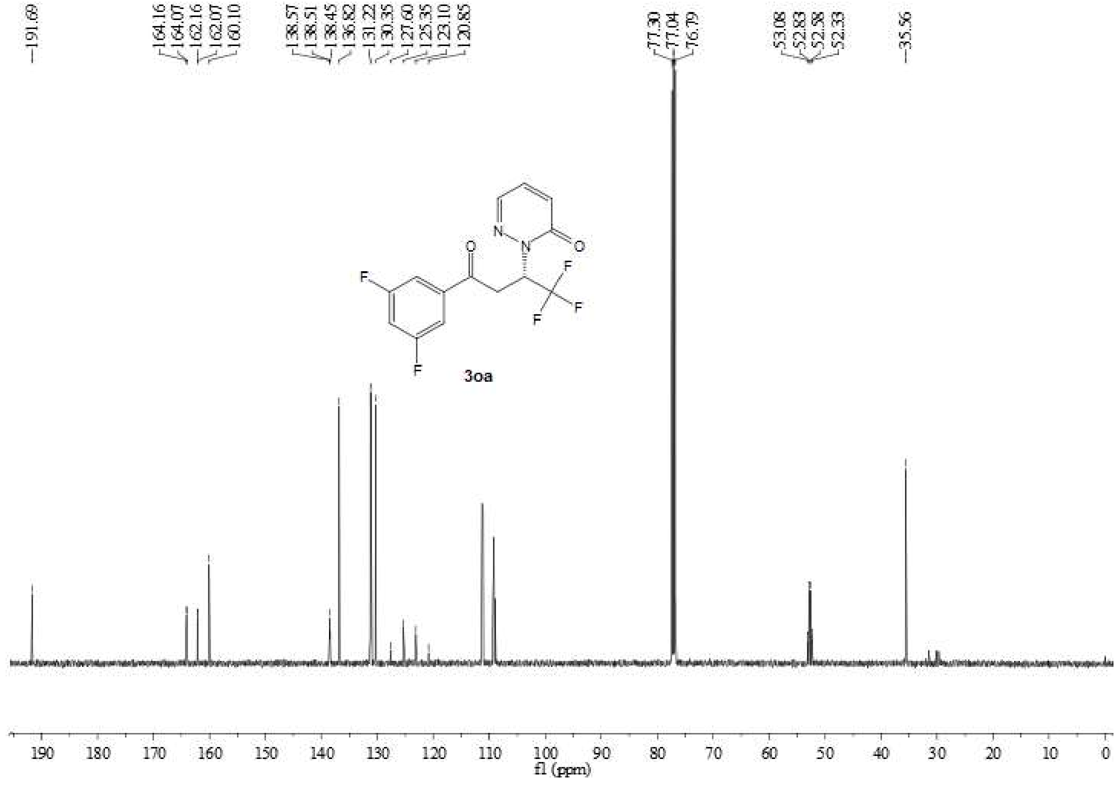

191.69
164.16
164.07
162.16
162.07
160.10
138.57
138.51
138.45
136.82
131.22
130.35
127.60
125.35
123.10
120.85
77.30
77.04
76.79
53.08
52.83
52.58
52.33
35.56
F
F
O
N
N
O
F
F
F
3oa
190 180 170 160 150 140 130 120 110 100 90 80 70 60 50 40 30 20 10 0
f1 (ppm)

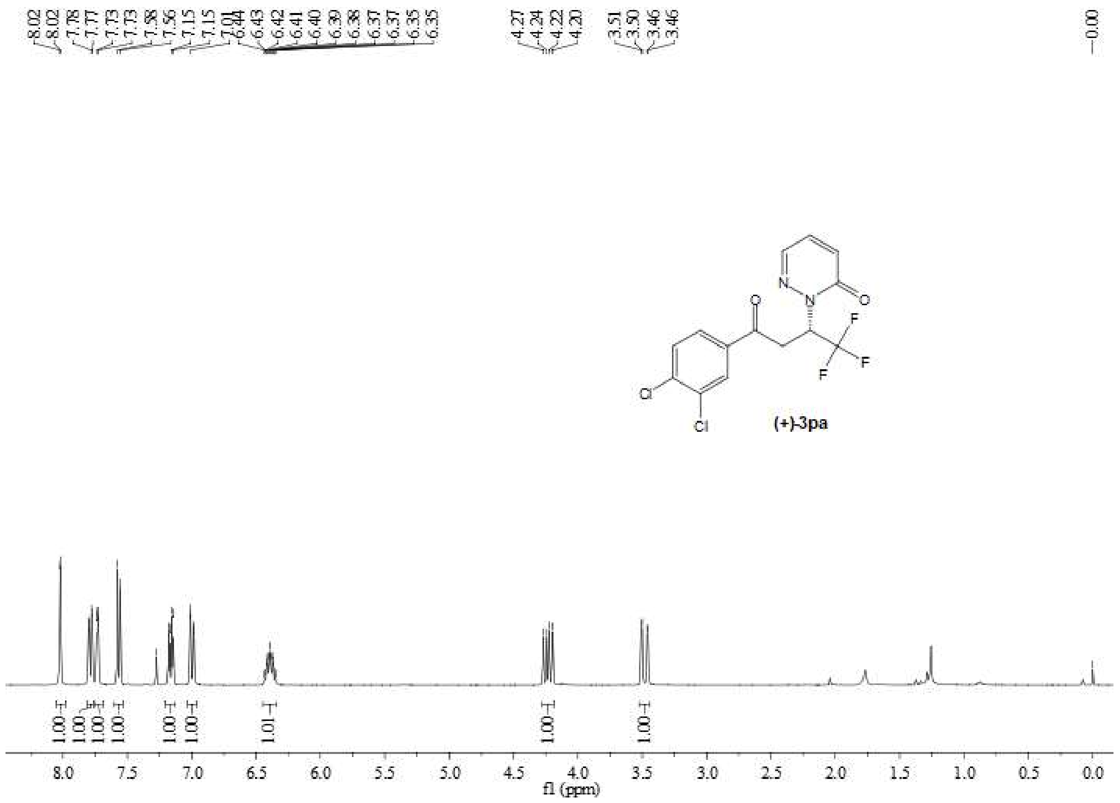
8.02 8.02 7.78 7.77 7.73 7.73 7.58 7.56 7.15 7.15 7.01 6.44 6.43 6.42 6.41 6.40 6.39 6.38 6.37 6.37 6.35 6.35
4.27 4.24 4.22 4.20
3.51 3.50 3.46 3.46
0.00
O
N
N
O
F
F
F
Cl
Cl
(+)-3pa
1.00 1.00 1.00 1.00 1.00 1.00 1.01 1.00 1.00
fl (ppm)

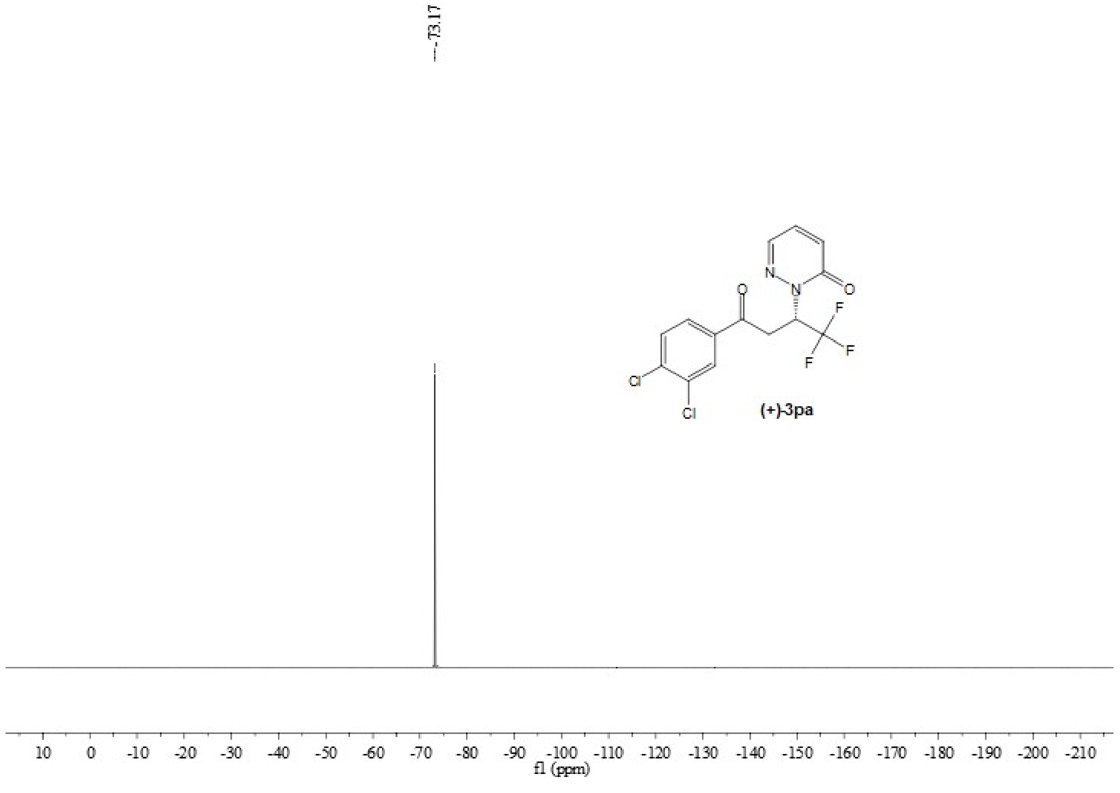
-73.17
O
N
N
O
F
F
F
Cl
Cl
(+)-3pa
fl (ppm)

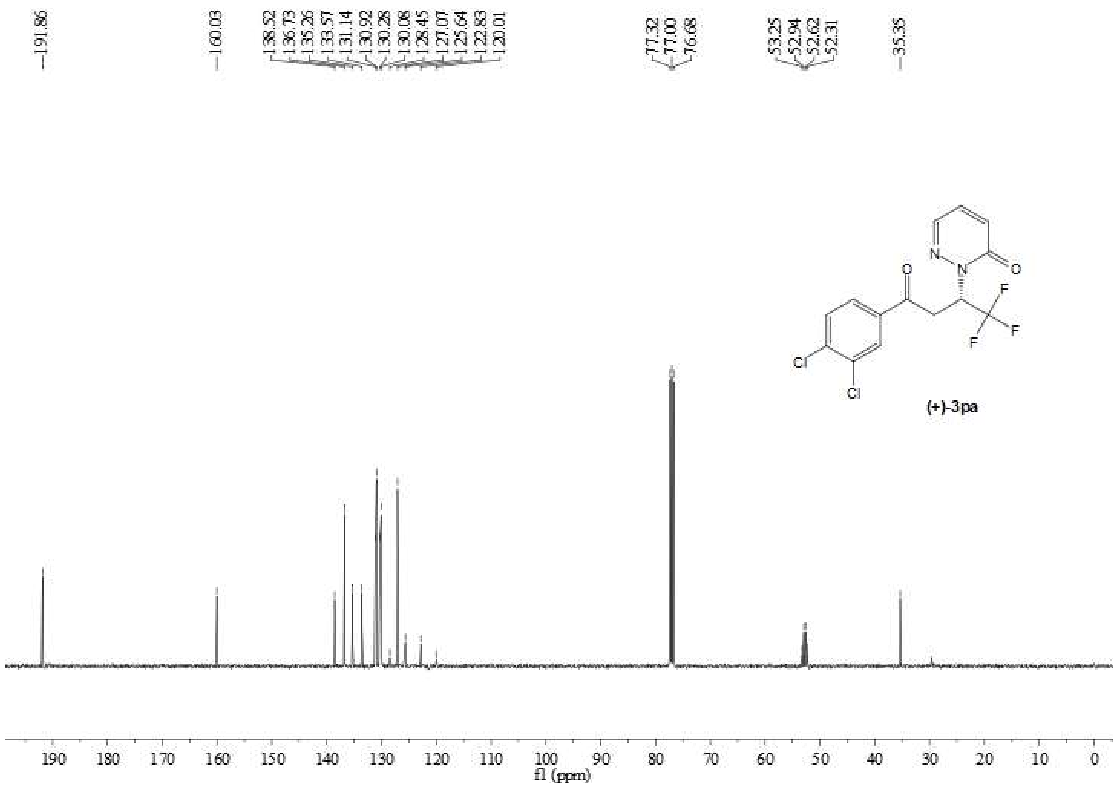
191.86
160.03
138.52
136.73
135.26
133.57
131.14
130.92
130.28
130.08
128.45
127.07
125.64
122.83
120.01
77.32
77.00
76.68
53.25
52.94
52.62
52.31
35.35
(+)-3pa
f1 (ppm)

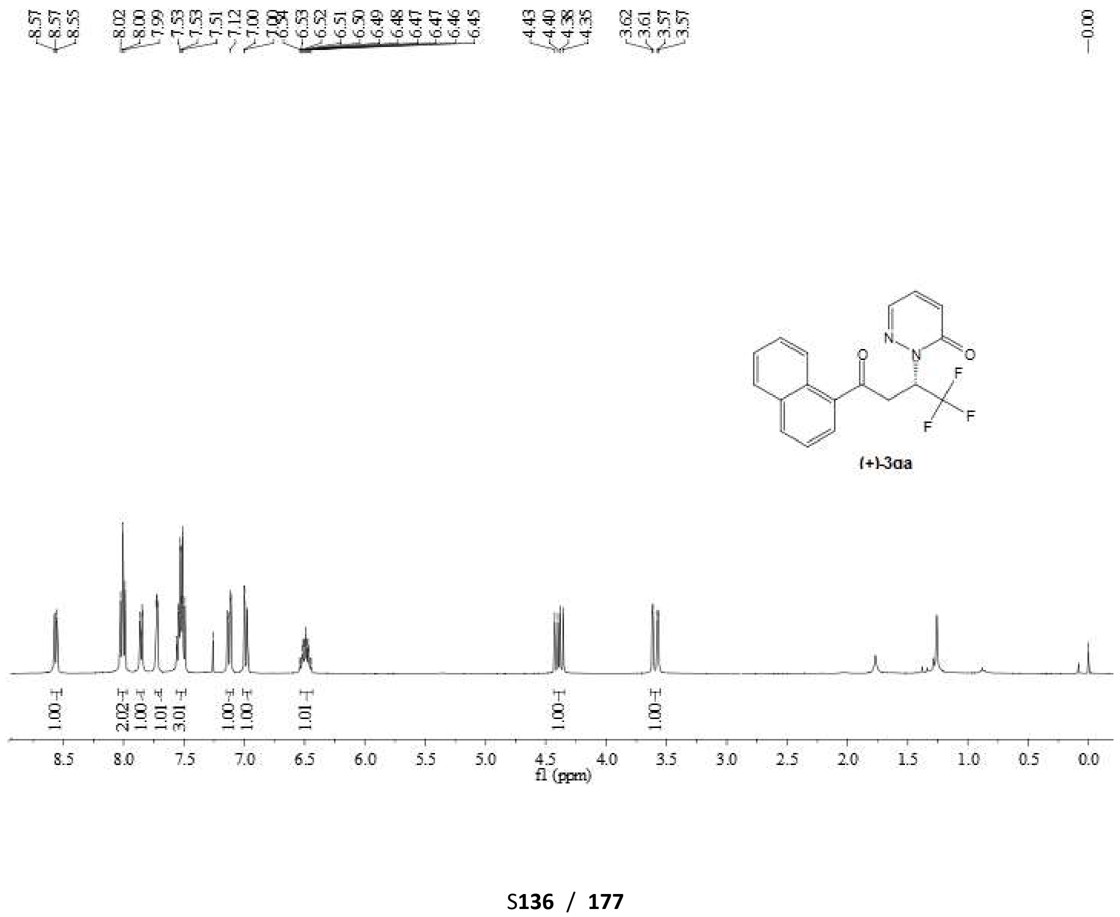
8.57
8.57
8.55
8.02
8.00
7.99
7.53
7.53
7.51
7.12
7.00
6.53
6.52
6.51
6.50
6.49
6.48
6.47
6.46
6.45
4.43
4.40
4.38
4.35
3.62
3.61
3.57
3.57
0.00
(+)-3qa
1.00
2.02
1.00
1.01
3.01
1.00
1.00
1.01
1.00
1.00
f1 (ppm)

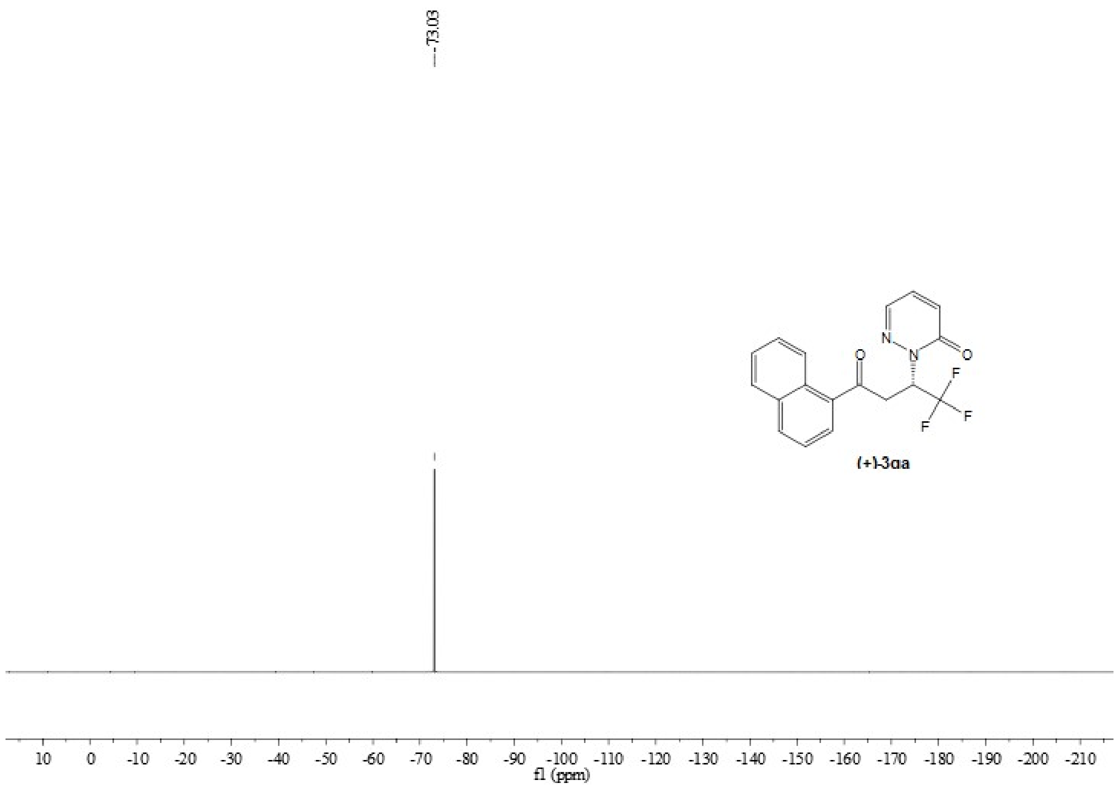
-73.03
(+)-3qa
10 0 -10 -20 -30 -40 -50 -60 -70 -80 -90 -100 -110 -120 -130 -140 -150 -160 -170 -180 -190 -200 -210
f1 (ppm)

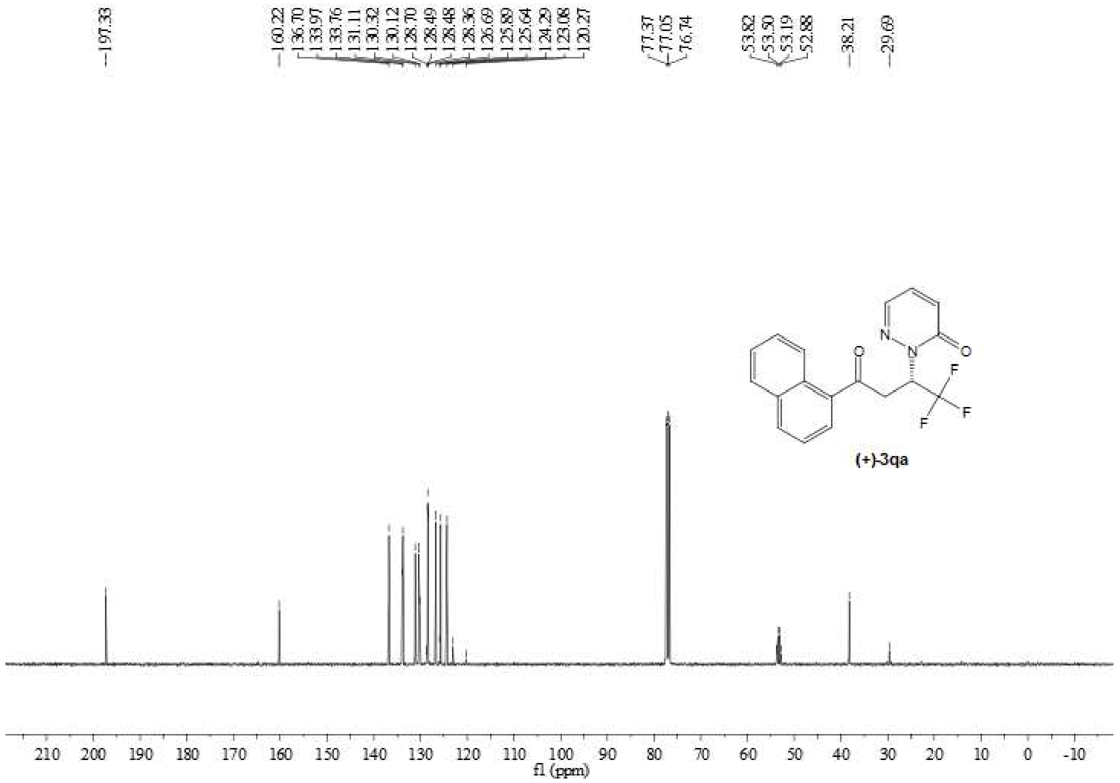
197.33
160.22 136.70 133.97 133.76 131.11 130.32 130.12 128.70 128.49 128.48 128.36 126.69 125.89 125.64 124.29 123.08 120.27
77.37 77.05 76.74
53.82 53.50 53.19 52.88
38.21
29.69
(+)-3qa
210 200 190 180 170 160 150 140 130 120 110 100 90 80 70 60 50 40 30 20 10 0 -10
f1 (ppm)

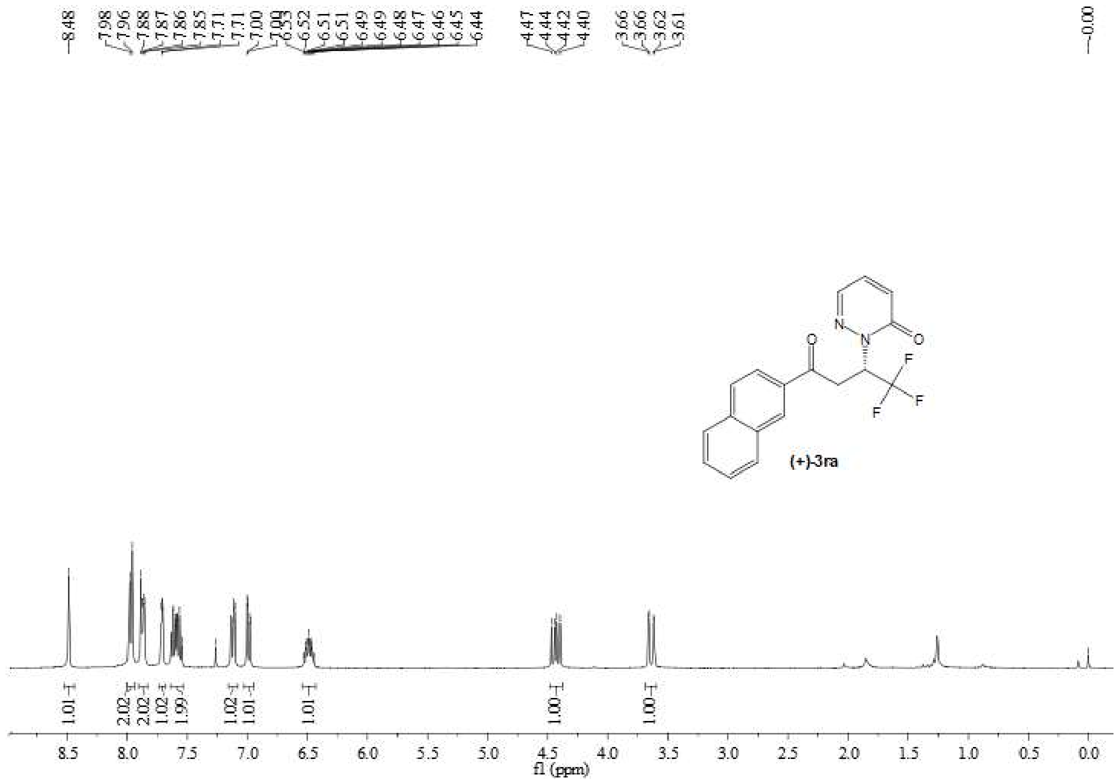
8.48
7.98
7.96
7.88
7.87
7.86
7.85
7.71
7.71
7.00
6.53
6.52
6.51
6.51
6.49
6.49
6.48
6.47
6.46
6.45
6.44
4.47
4.44
4.42
4.40
3.66
3.66
3.62
3.61
0.00
(+)-3ra
1.01
2.02
2.02
1.02
1.99
1.02
1.01
1.01
1.00
1.00
fl (ppm)

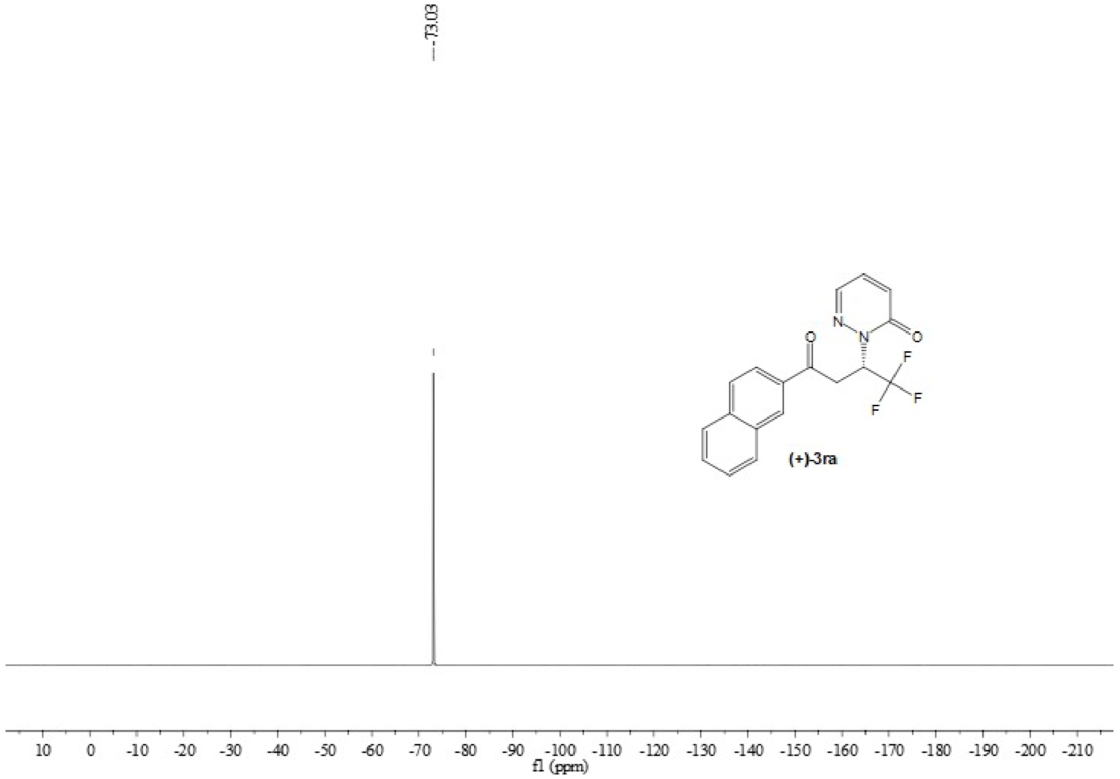
-73.03
(+)-3ra
fl (ppm)

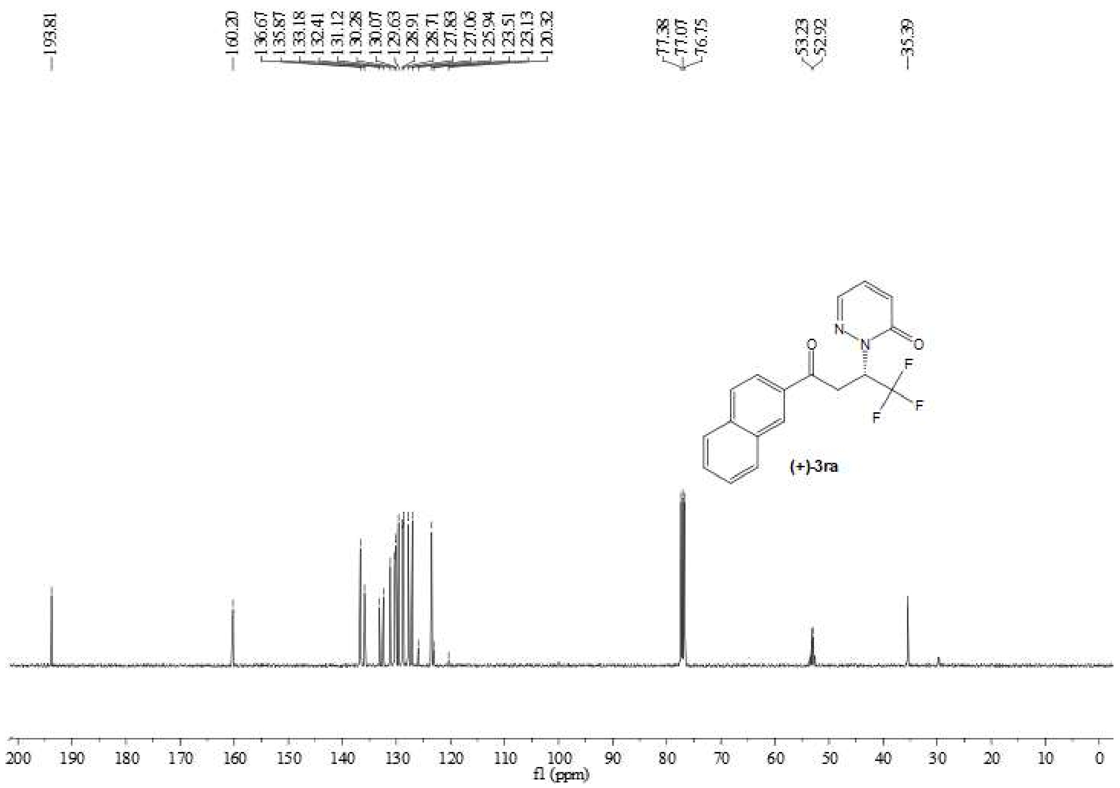
193.81
160.20
136.67
135.87
133.18
132.41
131.12
130.28
130.07
129.63
128.91
128.71
127.83
127.06
125.94
123.51
123.13
120.32
77.38
77.07
76.75
53.23
52.92
35.39
(+)-3ra
fl (ppm)

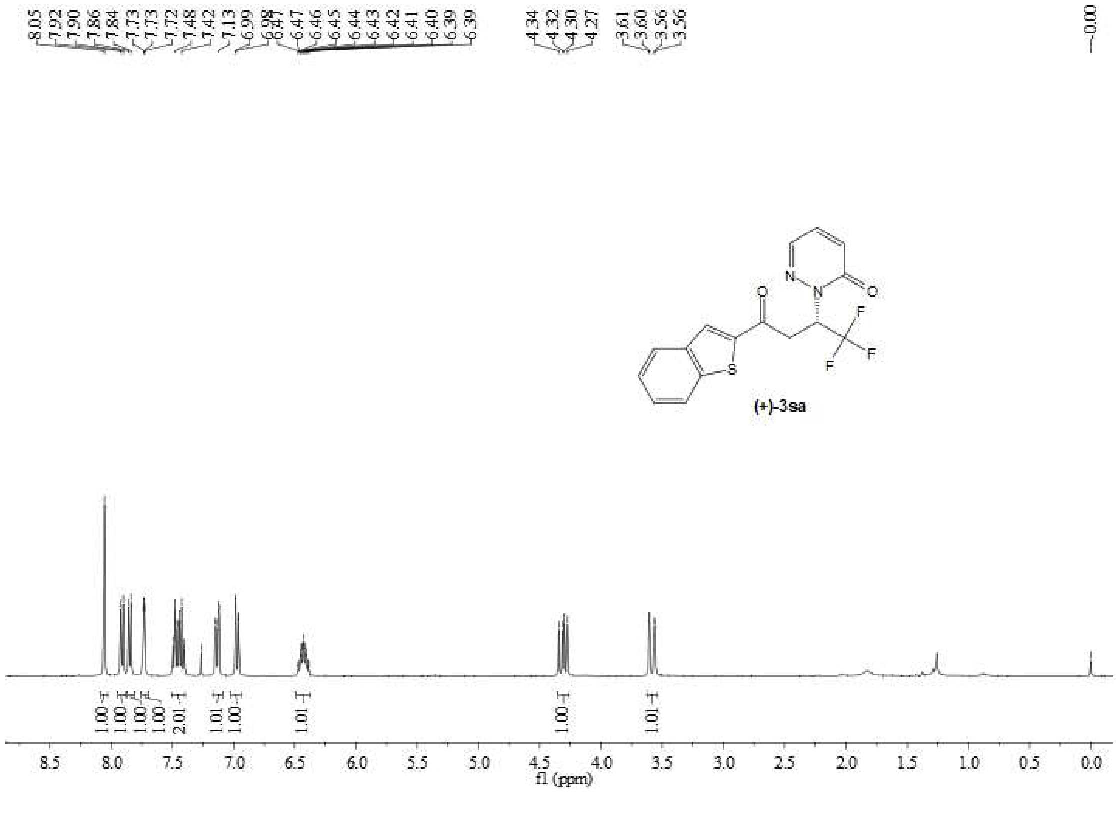
8.05
7.92
7.90
7.86
7.84
7.73
7.73
7.72
7.48
7.42
7.13
6.99
6.98
6.47
6.47
6.46
6.45
6.44
6.43
6.42
6.41
6.40
6.39
6.39
4.34
4.32
4.30
4.27
3.61
3.60
3.56
3.56
0.00
(+)-3sa
1.00
1.00
1.00
1.00
2.01
1.01
1.00
1.01
1.00
1.01
fl (ppm)

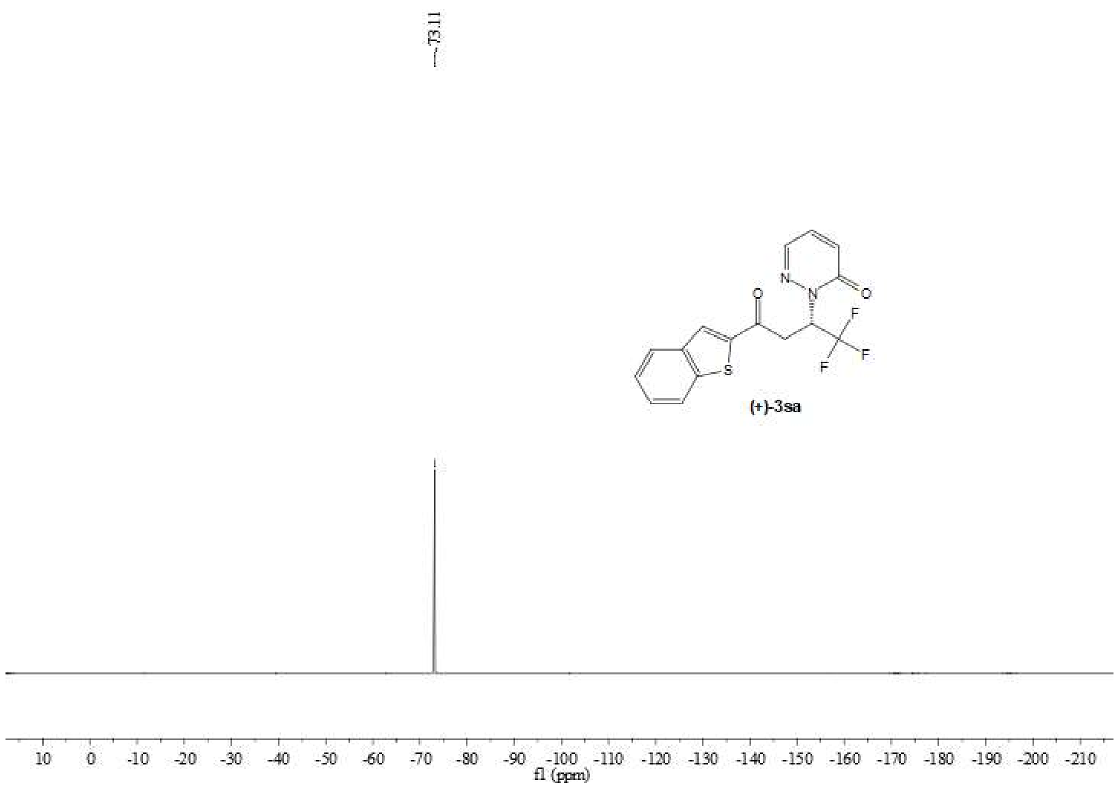
-73.11
(+)-3sa
fl (ppm)

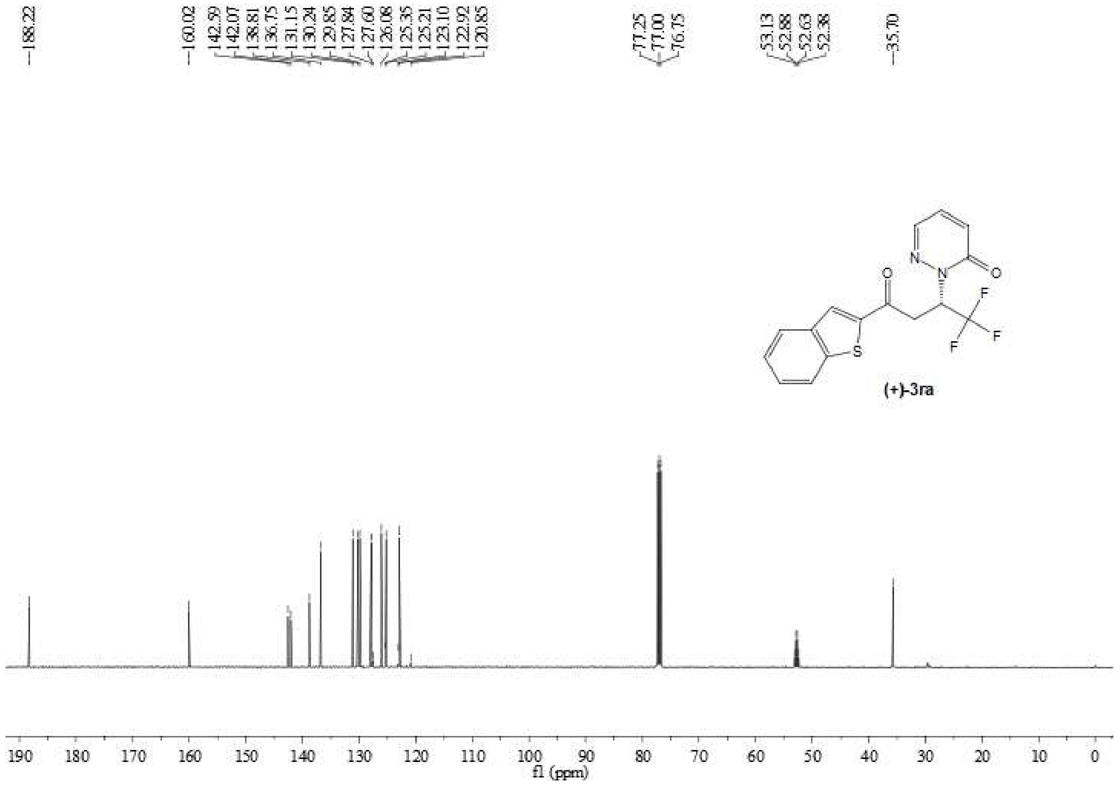
188.22
160.02
142.59
142.07
138.81
136.75
131.15
130.24
129.85
127.84
127.60
126.08
125.35
125.21
123.10
122.92
120.85
77.25
77.00
76.75
53.13
52.88
52.63
52.38
35.70
(+)-3ra
fl (ppm)

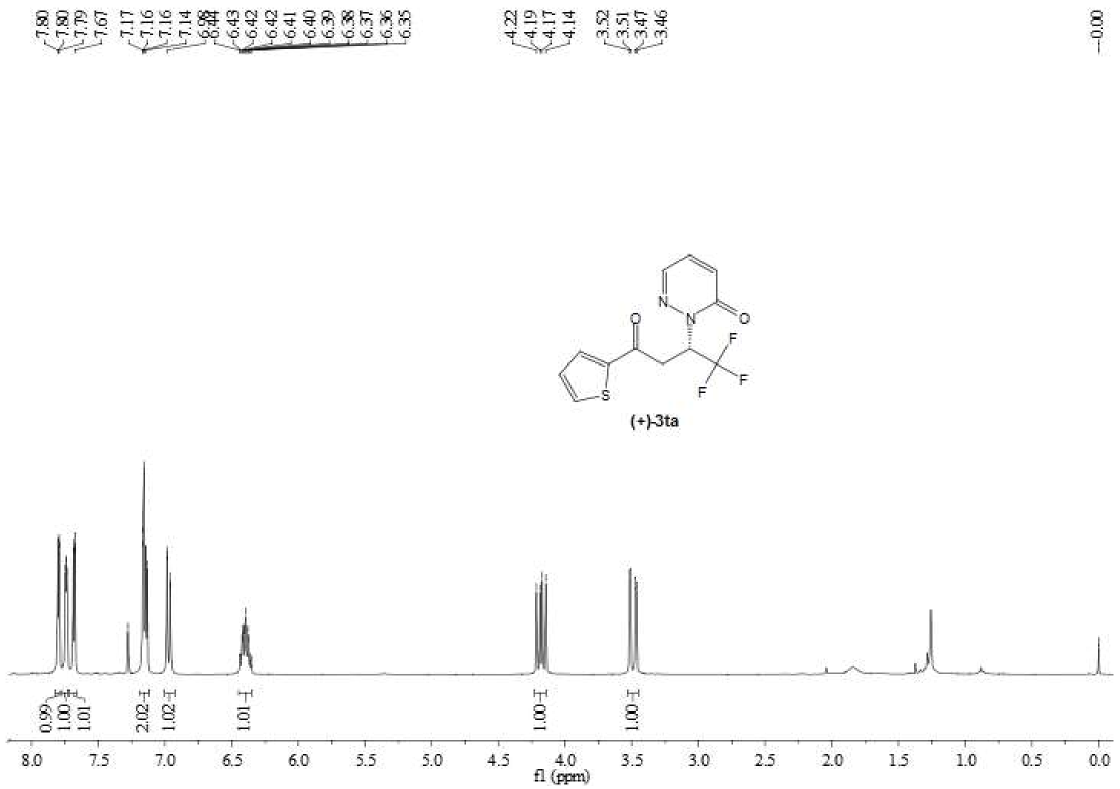

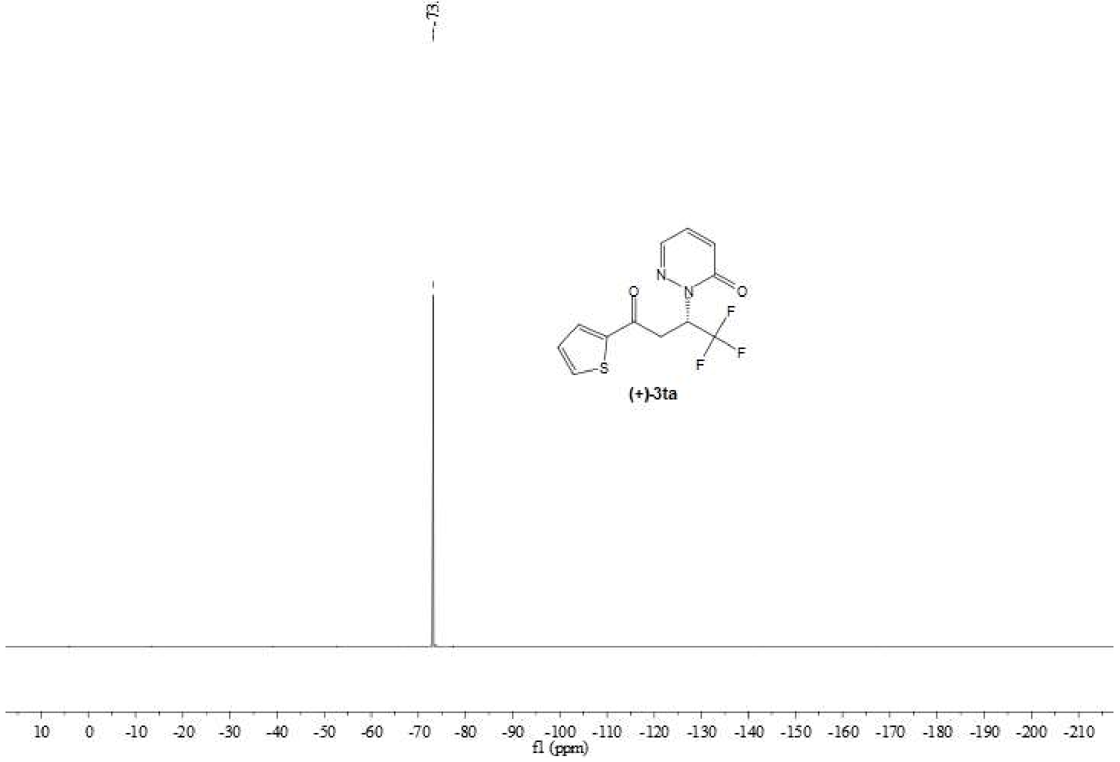

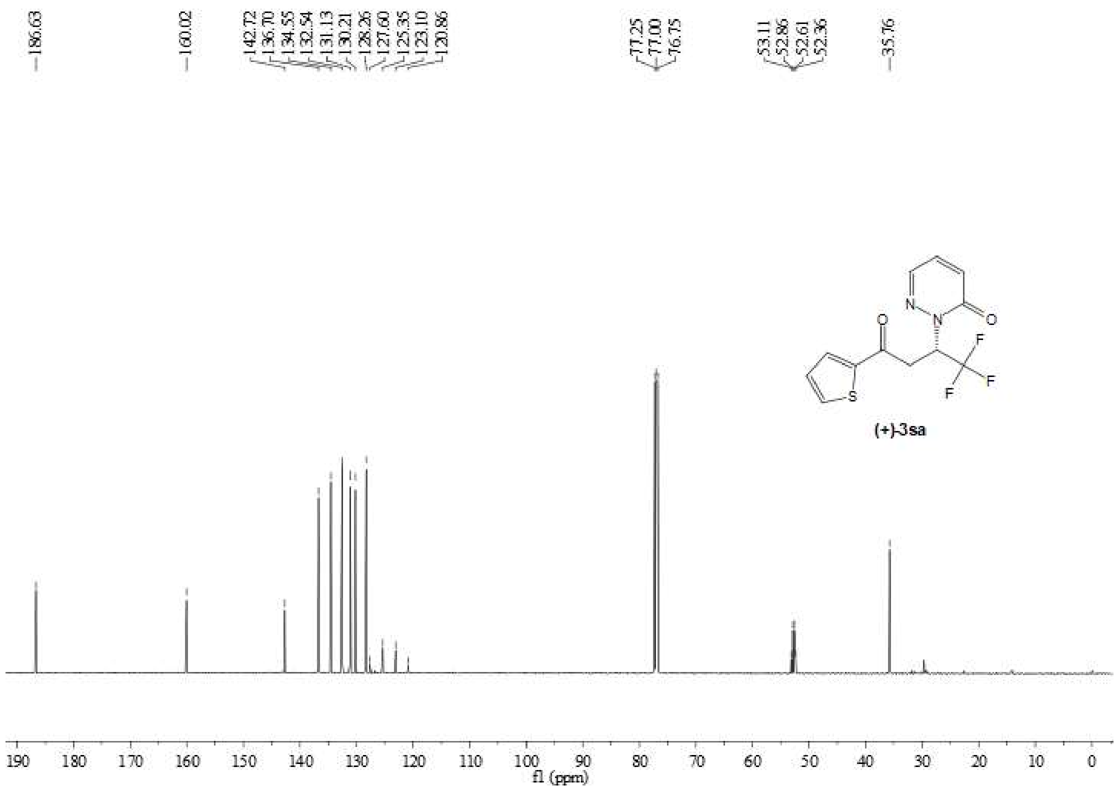
186.63
160.02
142.72
136.70
134.55
132.54
131.13
130.21
128.26
127.60
125.35
123.10
120.86
77.25
77.00
76.75
53.11
52.86
52.61
52.36
35.76
(+)-3sa
f1 (ppm)

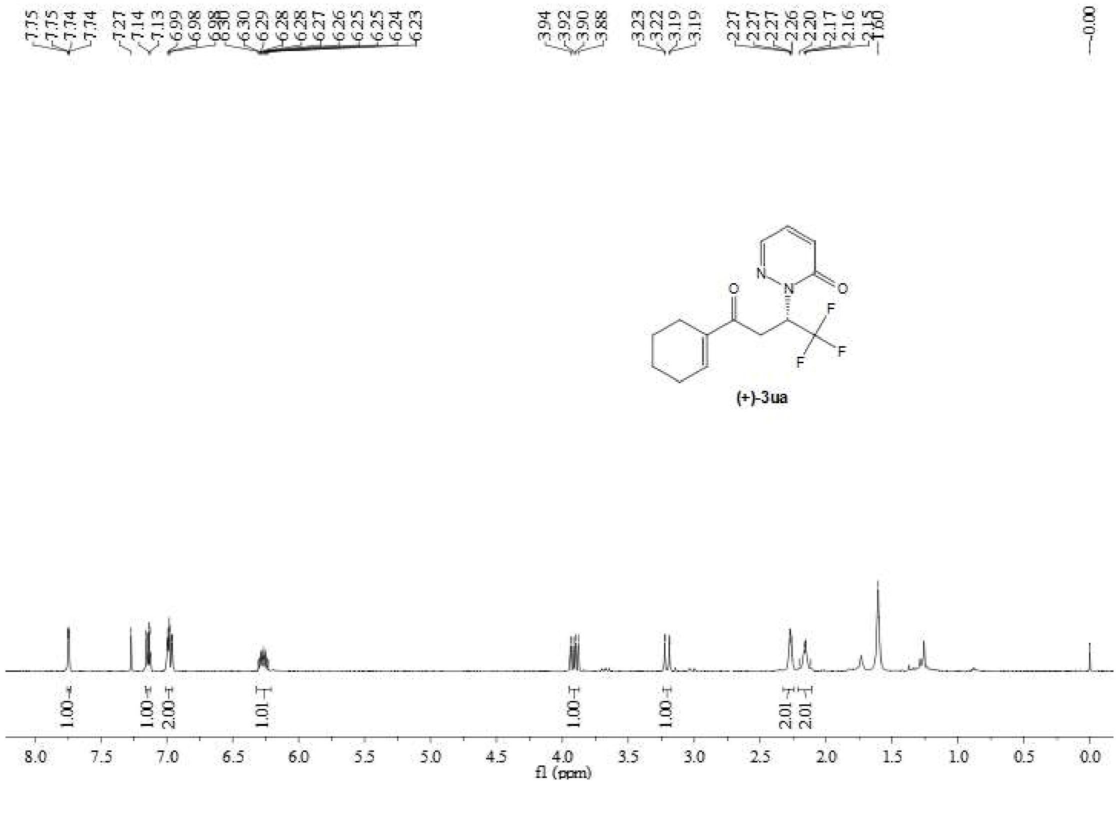
7.75
7.75
7.74
7.74
7.27
7.14
7.13
6.99
6.98
6.30
6.29
6.28
6.28
6.27
6.26
6.25
6.25
6.24
6.23
3.94
3.92
3.90
3.88
3.23
3.22
3.19
3.19
2.27
2.27
2.27
2.26
2.20
2.17
2.16
2.15
1.60
0.00
(+)-3ua
1.00
1.00
2.00
1.01
1.00
1.00
2.01
2.01
f1 (ppm)

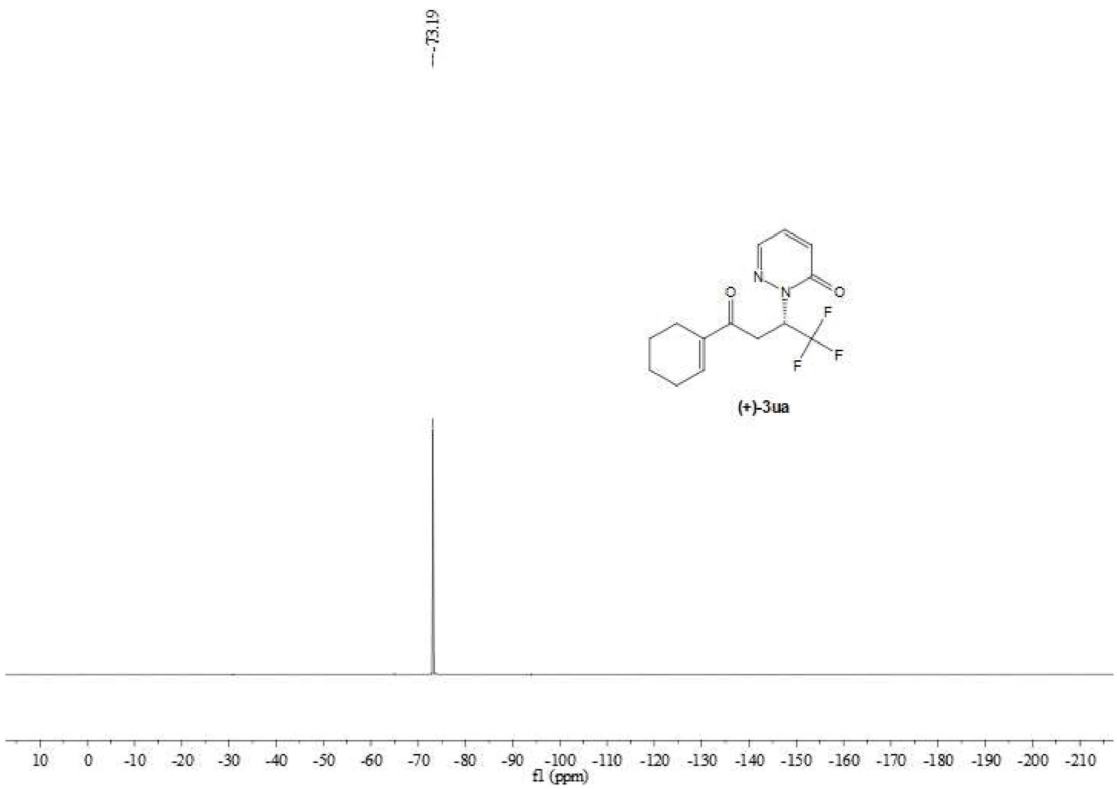
-73.19
N
N
O
O
F
F
F
(+)-3ua
10 0 -10 -20 -30 -40 -50 -60 -70 -80 -90 -100 -110 -120 -130 -140 -150 -160 -170 -180 -190 -200 -210
f1 (ppm)

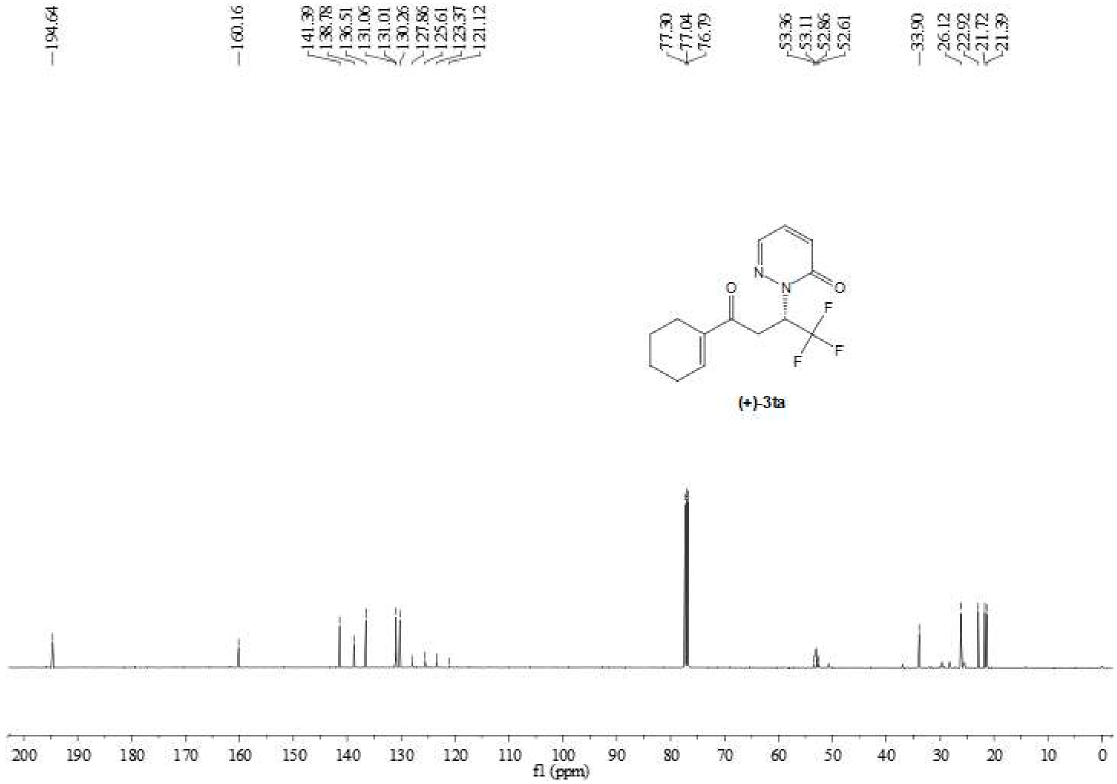
194.64
160.16
141.39
138.78
136.51
131.06
131.01
130.26
127.86
125.61
123.37
121.12
77.30
77.04
76.79
53.36
53.11
52.86
52.61
33.90
26.12
22.92
21.72
21.39
N
N
O
O
F
F
F
(+)-3ta
200 190 180 170 160 150 140 130 120 110 100 90 80 70 60 50 40 30 20 10 0
f1 (ppm)

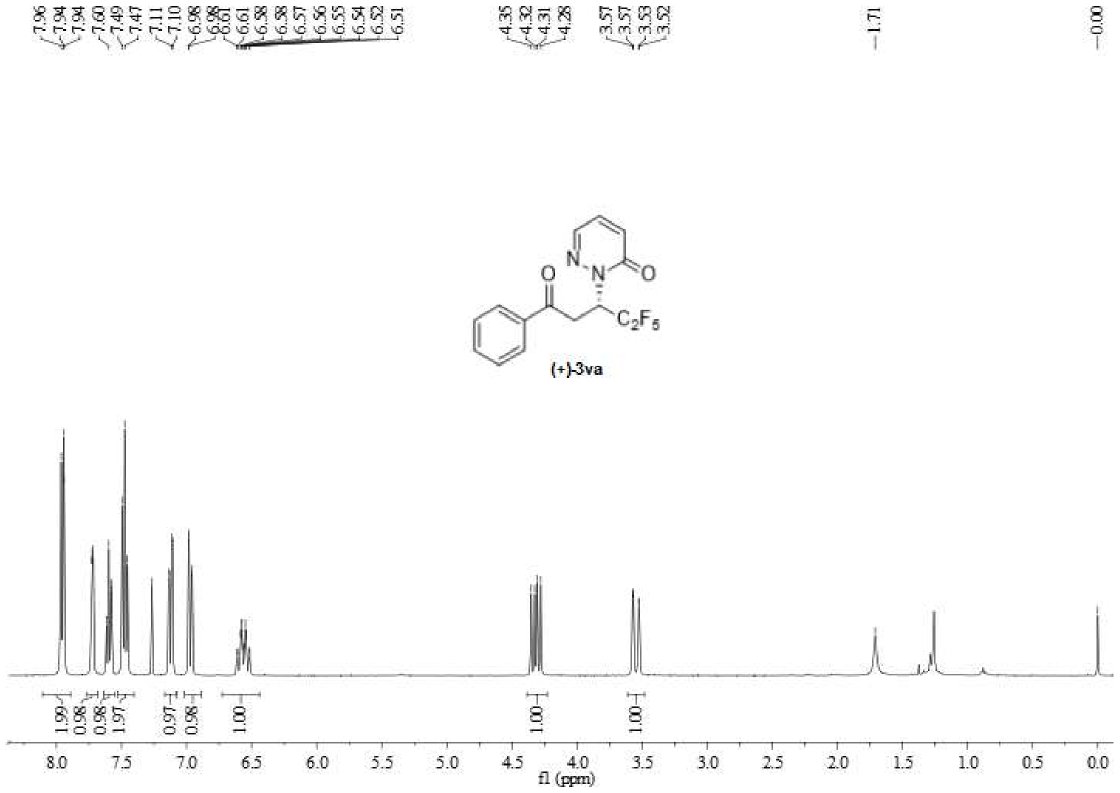

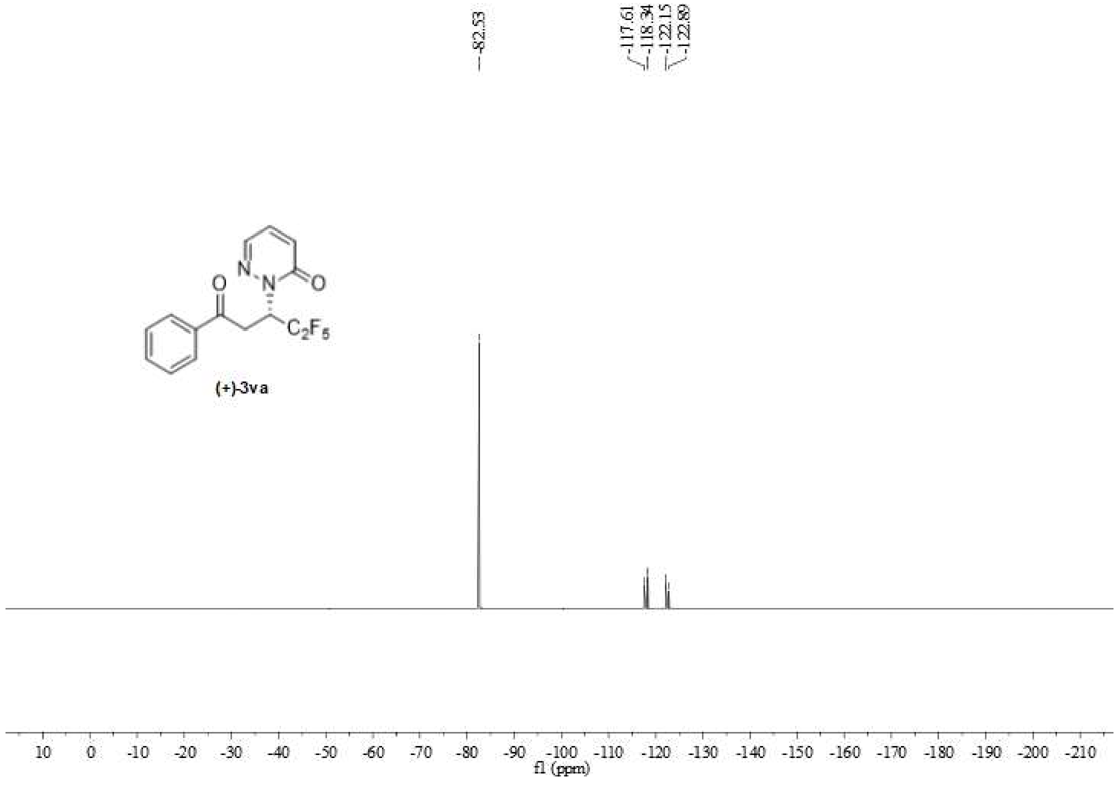

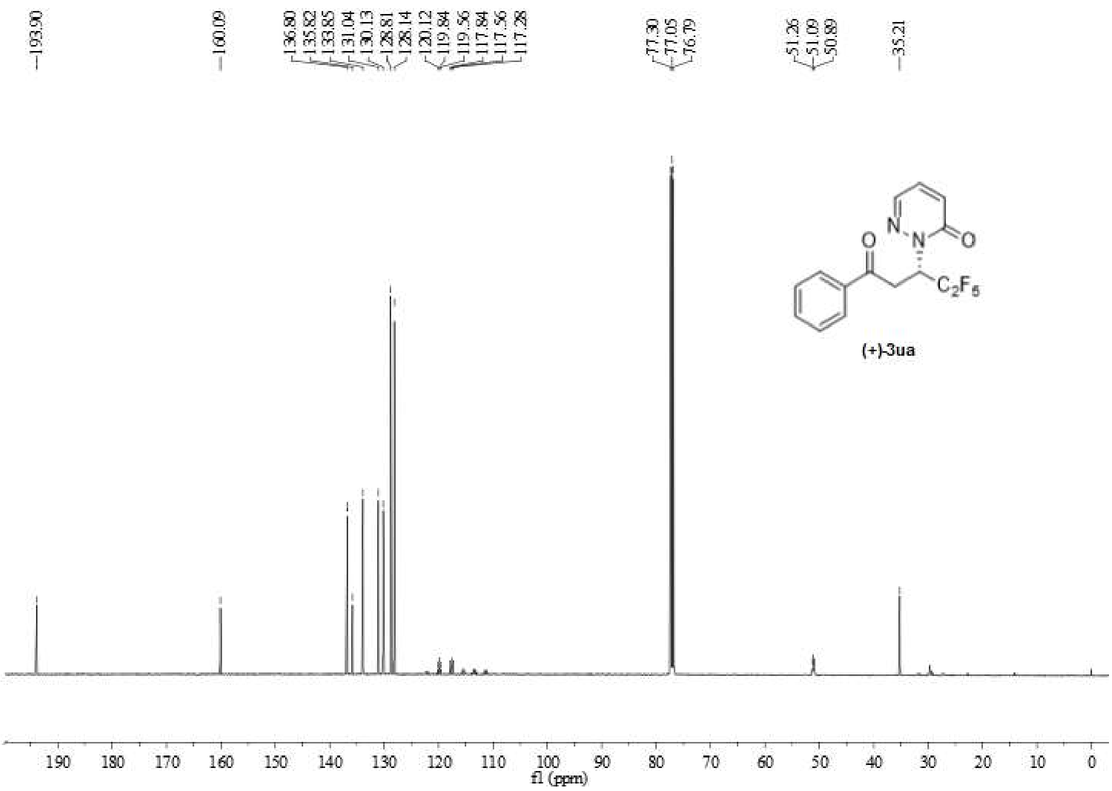
193.90
160.09
136.80
135.82
133.85
131.04
130.13
128.81
128.14
120.12
119.84
119.56
117.84
117.56
117.28
77.30
77.05
76.79
51.26
51.09
50.89
35.21
(+)-3ua
f1 (ppm)

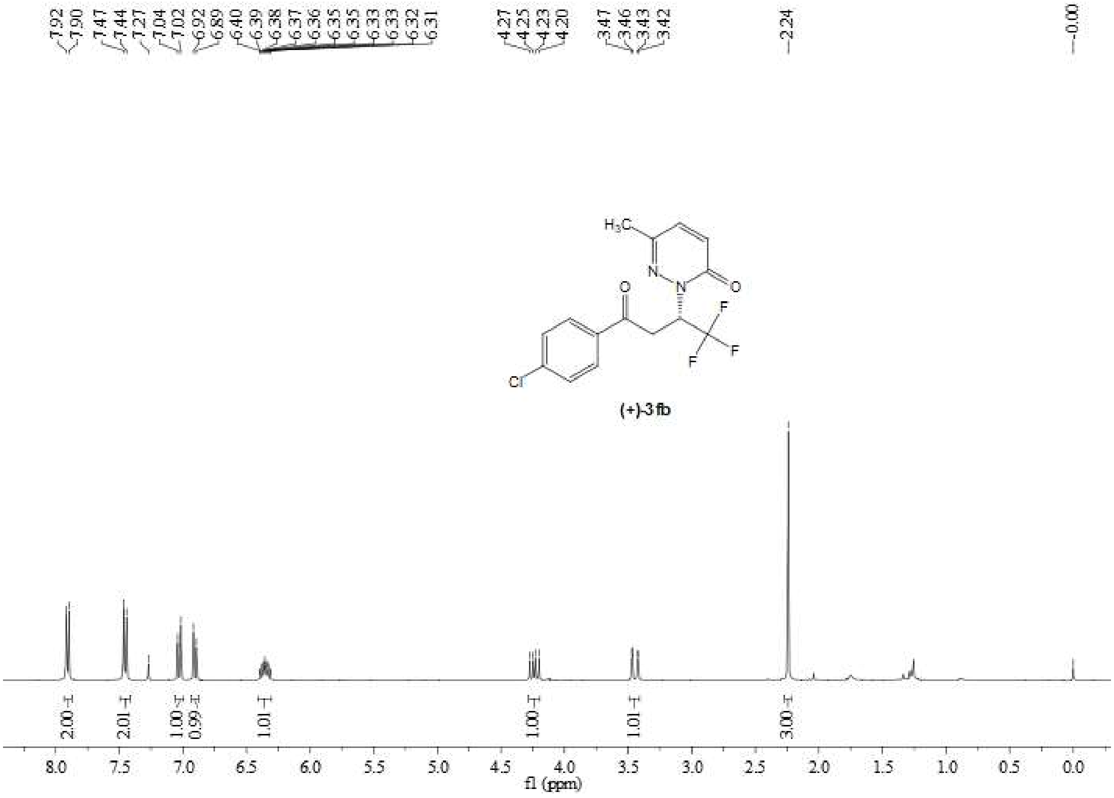
7.92
7.90
7.47
7.44
7.27
7.04
7.02
6.92
6.89
6.40
6.39
6.38
6.37
6.36
6.35
6.35
6.33
6.33
6.32
6.31
4.27
4.25
4.23
4.20
3.47
3.46
3.43
3.42
2.24
-0.00
(+)-3fb
2.00
2.01
1.00
0.99
1.01
1.00
1.01
3.00
f1 (ppm)

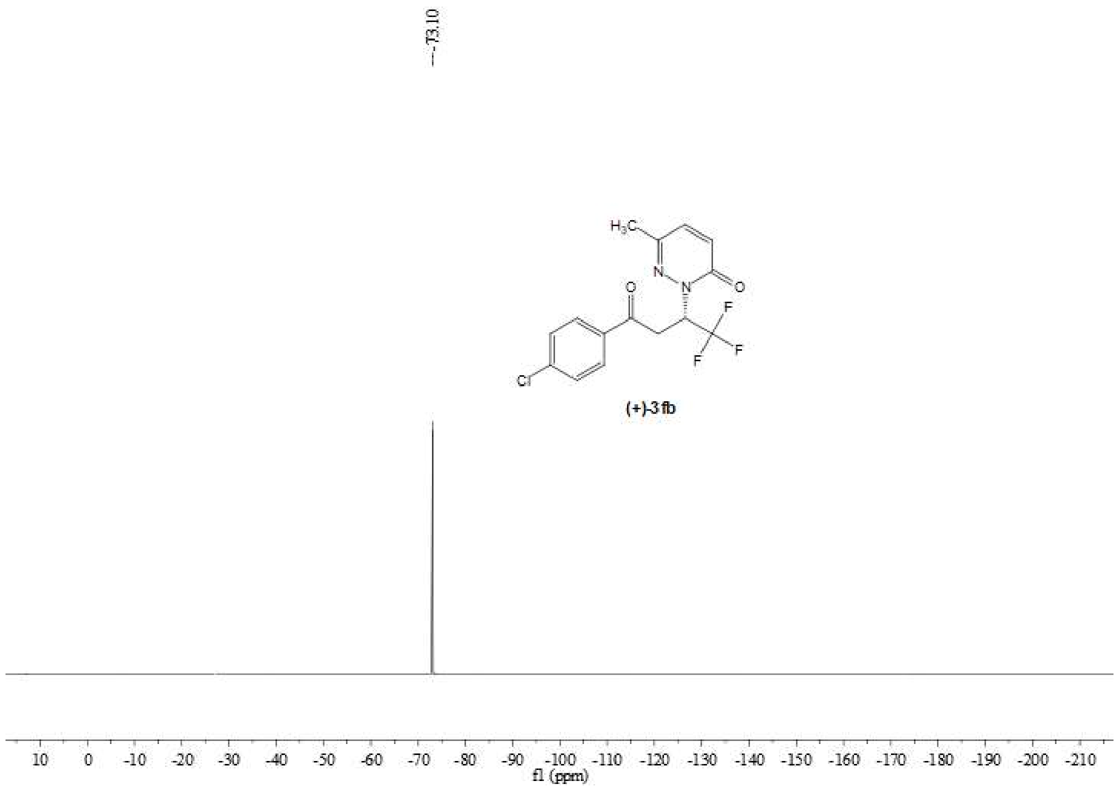

--73.10
H3C
N
N
O
O
F
F
F
Cl
(+)-3fb
10 0 -10 -20 -30 -40 -50 -60 -70 -80 -90 -100 -110 -120 -130 -140 -150 -160 -170 -180 -190 -200 -210
f1 (ppm)

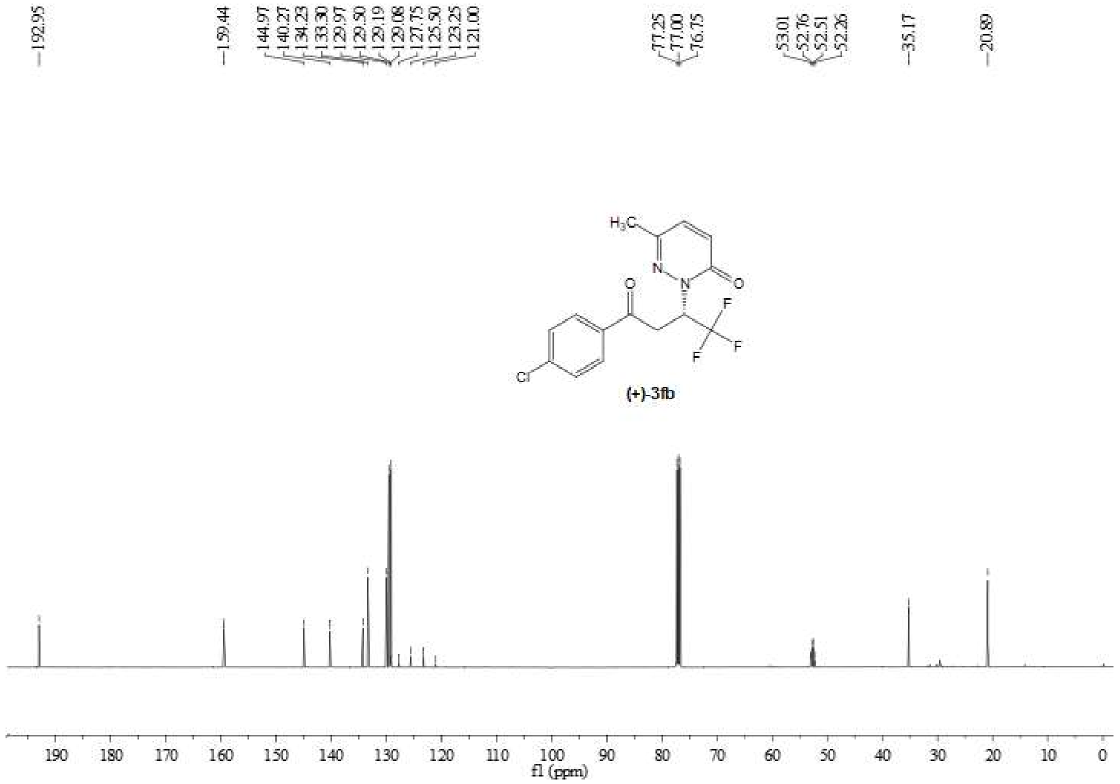

192.95
159.44
144.97 140.27 134.23 133.30 129.97 129.50 129.19 129.08 127.75 125.50 123.25 121.00
77.25 77.00 76.75
53.01 52.76 52.51 52.26
35.17
20.89
H3C
N
N
O
O
F
F
F
Cl
(+)-3fb
190 180 170 160 150 140 130 120 110 100 90 80 70 60 50 40 30 20 10 0
f1 (ppm)

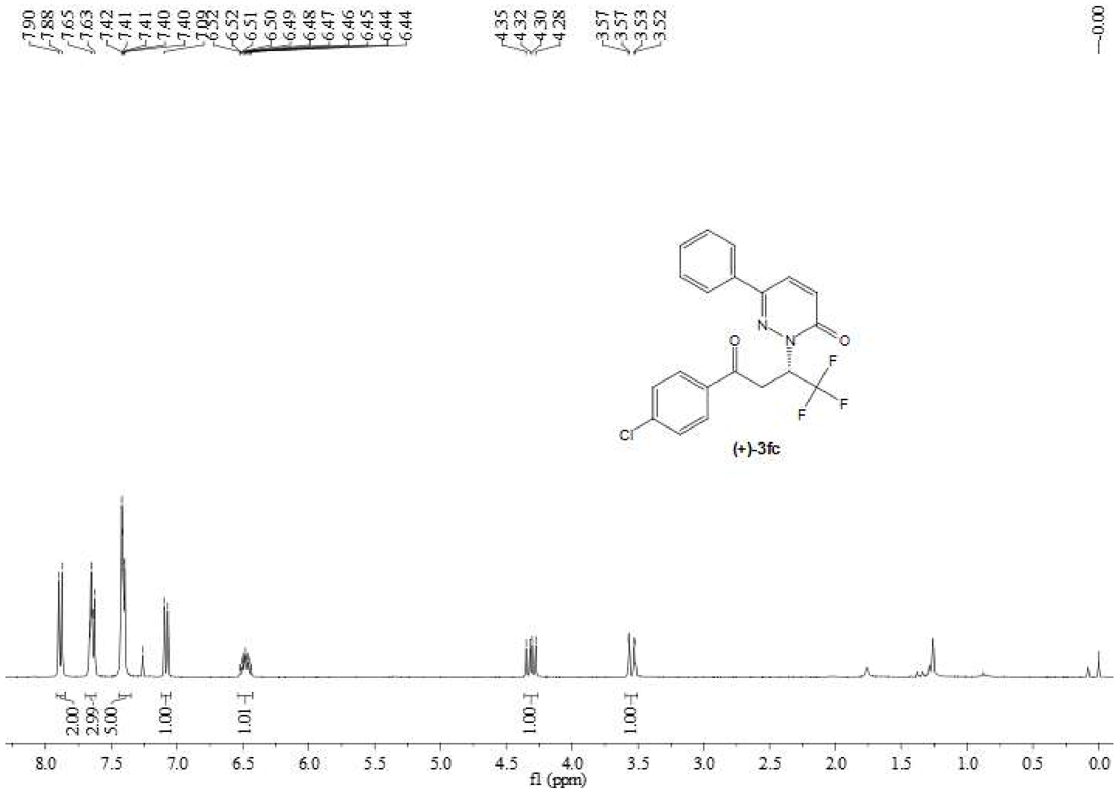
7.90
7.88
7.65
7.63
7.42
7.41
7.40
7.40
7.09
6.52
6.51
6.50
6.49
6.48
6.47
6.46
6.45
6.44
6.44
4.35
4.32
4.30
4.28
3.57
3.57
3.53
3.52
0.00
N
N
O
O
F
F
F
Cl
(+)-3fc
2.00
2.99
5.00
1.00
1.01
1.00
1.00
8.0 7.5 7.0 6.5 6.0 5.5 5.0 4.5 4.0 3.5 3.0 2.5 2.0 1.5 1.0 0.5 0.0
f1 (ppm)

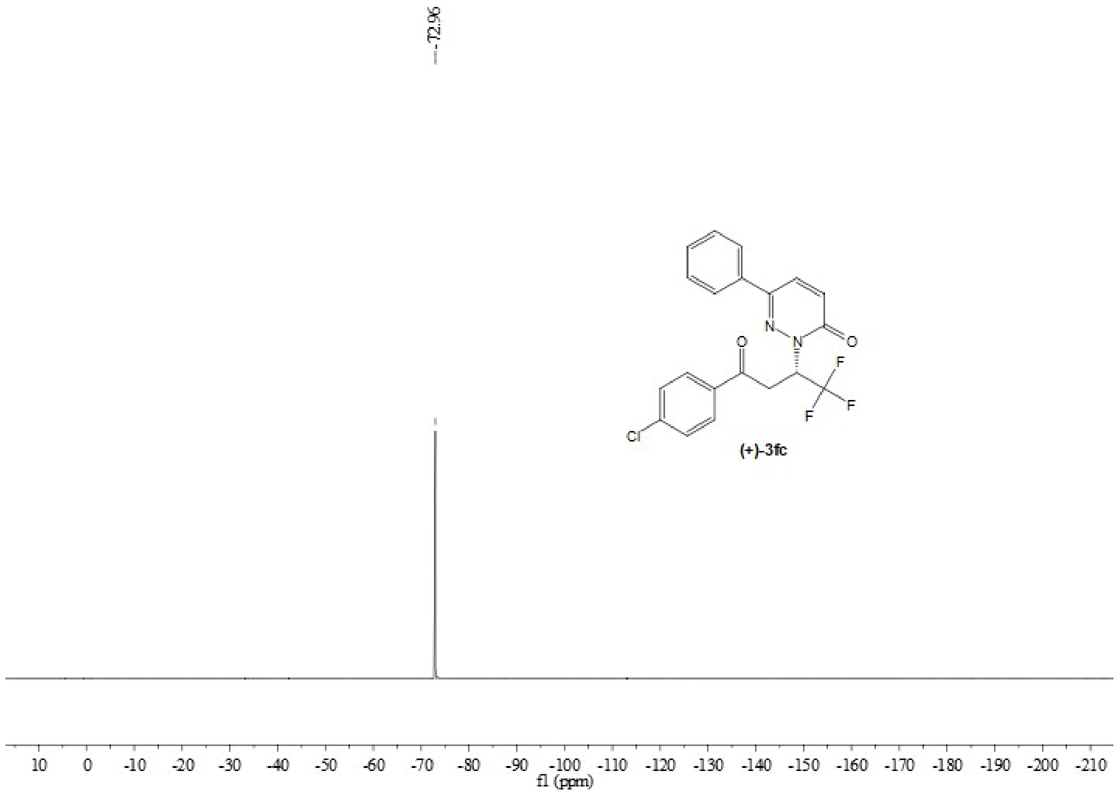
-72.96
N
N
O
O
F
F
F
Cl
(+)-3fc
10 0 -10 -20 -30 -40 -50 -60 -70 -80 -90 -100 -110 -120 -130 -140 -150 -160 -170 -180 -190 -200 -210
f1 (ppm)

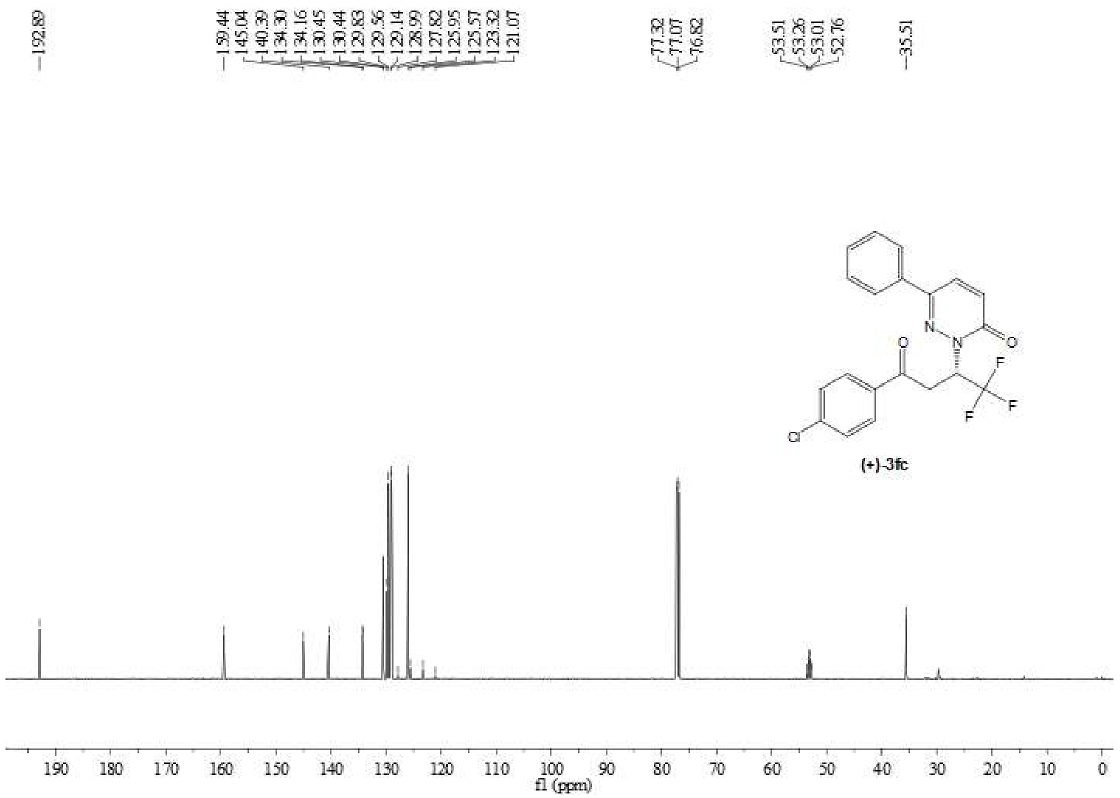
192.89
159.44
145.04
140.39
134.30
134.16
130.45
130.44
129.83
129.56
129.14
128.99
127.82
125.95
125.57
123.32
121.07
77.32
77.07
76.82
53.51
53.26
53.01
52.76
35.51
N
N
O
O
F
F
F
Cl
(+)-3fc
190 180 170 160 150 140 130 120 110 100 90 80 70 60 50 40 30 20 10 0
f1 (ppm)

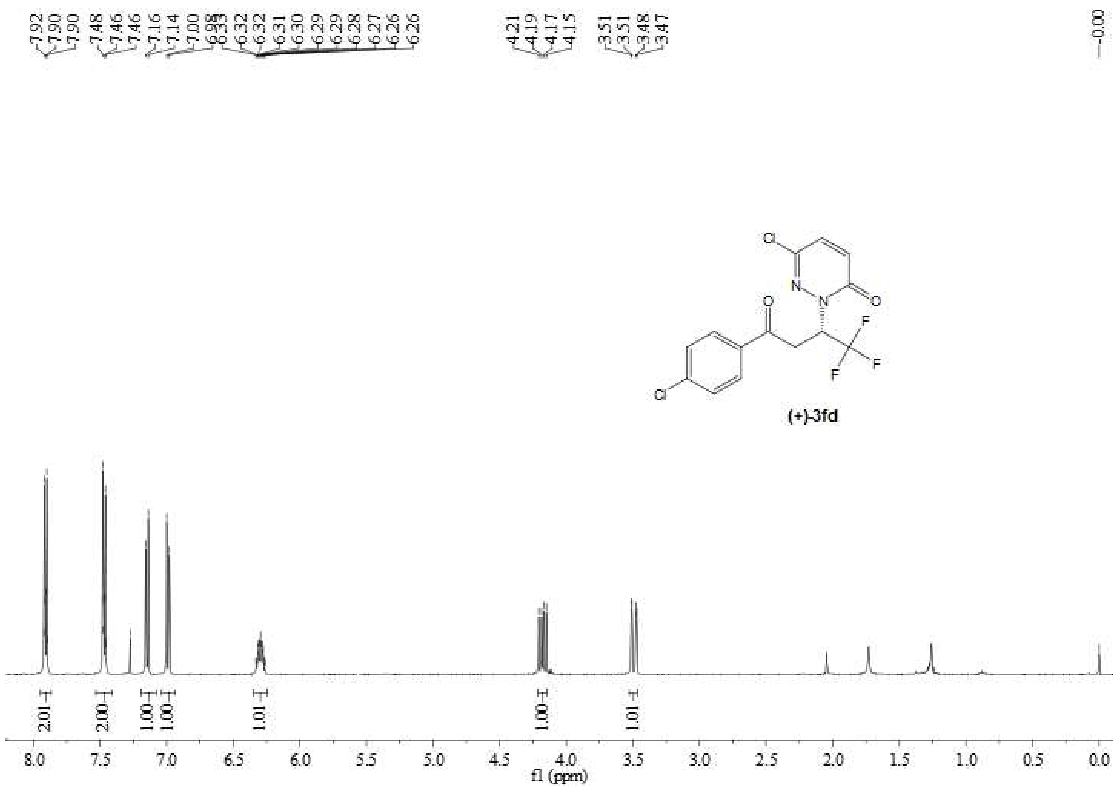
7.92
7.90
7.90
7.48
7.46
7.46
7.16
7.14
7.00
6.98
6.33
6.32
6.32
6.31
6.30
6.29
6.29
6.28
6.27
6.26
6.26
4.21
4.19
4.17
4.15
3.51
3.51
3.48
3.47
0.00
Cl
N
N
O
O
F
F
F
Cl
(+)-3fd
2.01
2.00
1.00
1.00
1.01
1.00
1.01
8.0 7.5 7.0 6.5 6.0 5.5 5.0 4.5 4.0 3.5 3.0 2.5 2.0 1.5 1.0 0.5 0.0
f1 (ppm)

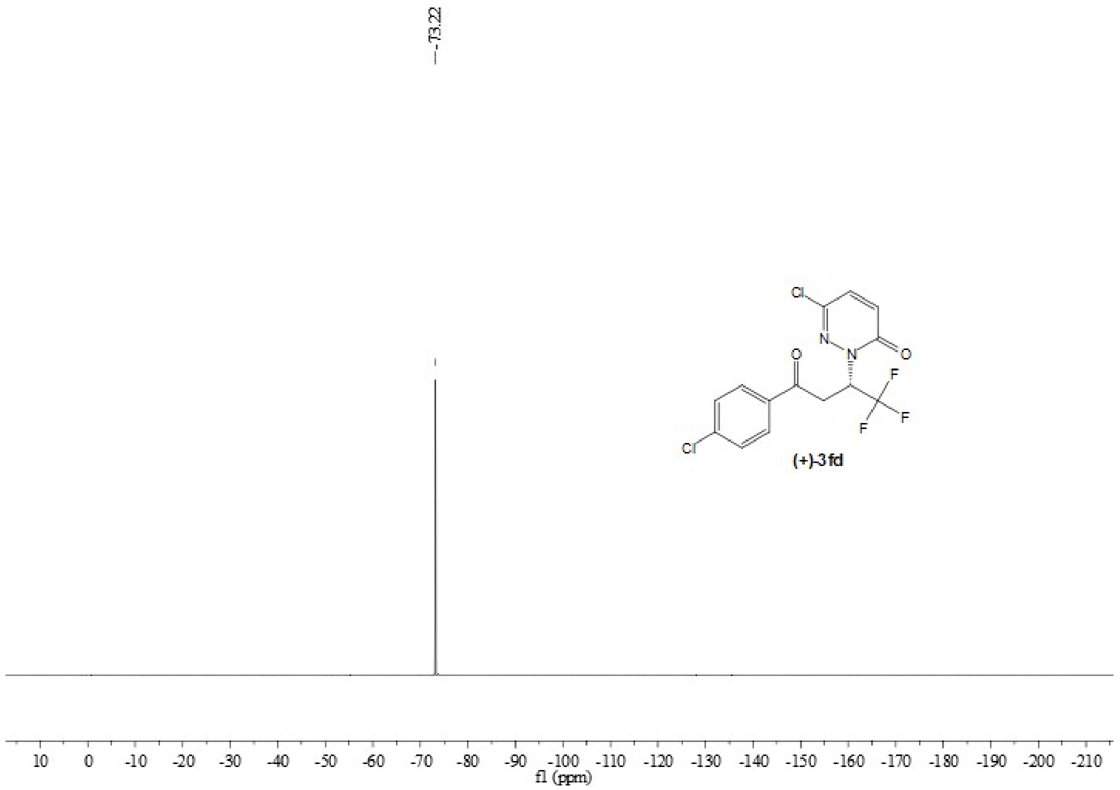

-73.22
Cl
N
N
O
O
F
F
F
Cl
(+)-3fd
f1 (ppm)

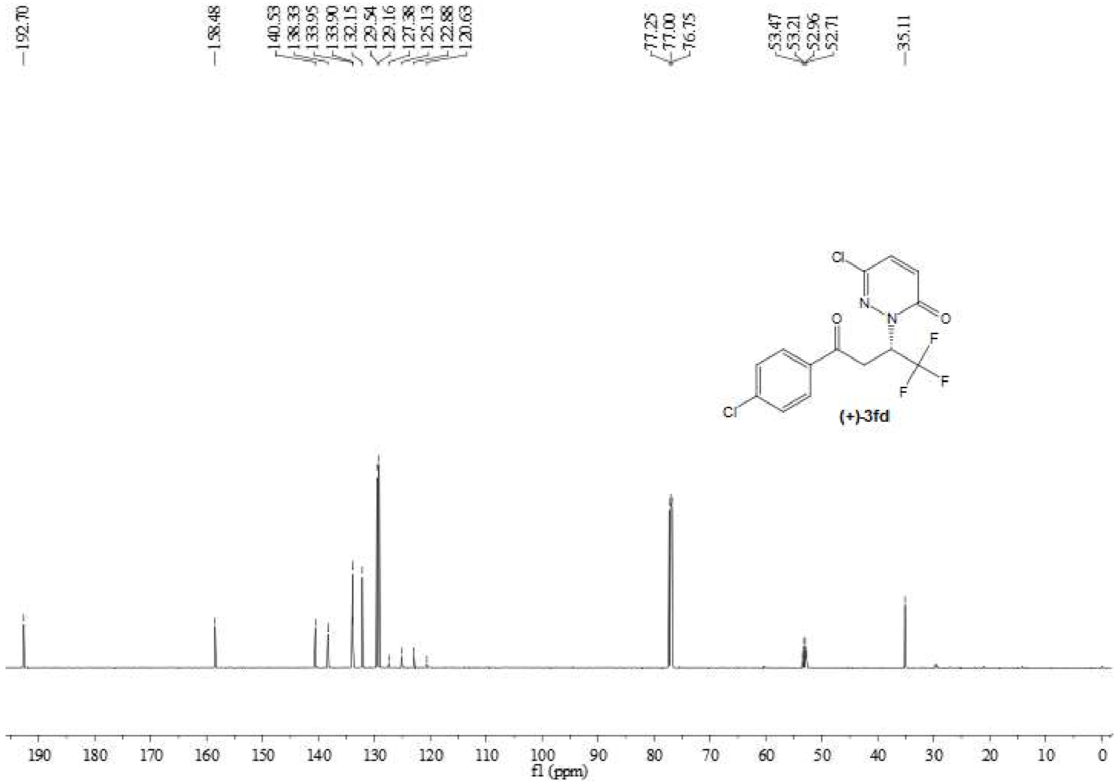

192.70
158.48
140.53
138.33
133.95
133.90
132.15
129.54
129.16
127.38
125.13
122.88
120.63
77.25
77.00
76.75
53.47
53.21
52.96
52.71
35.11
Cl
N
N
O
O
F
F
F
Cl
(+)-3fd
f1 (ppm)

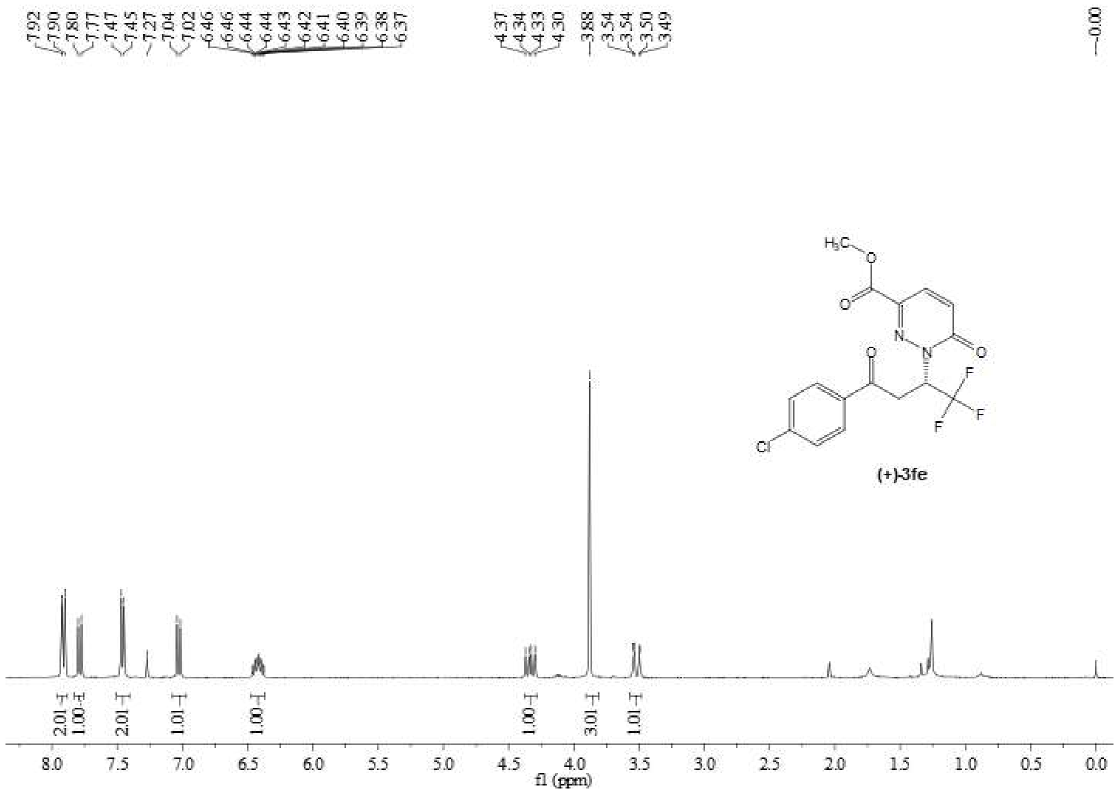
(+)-3fe
f1 (ppm)

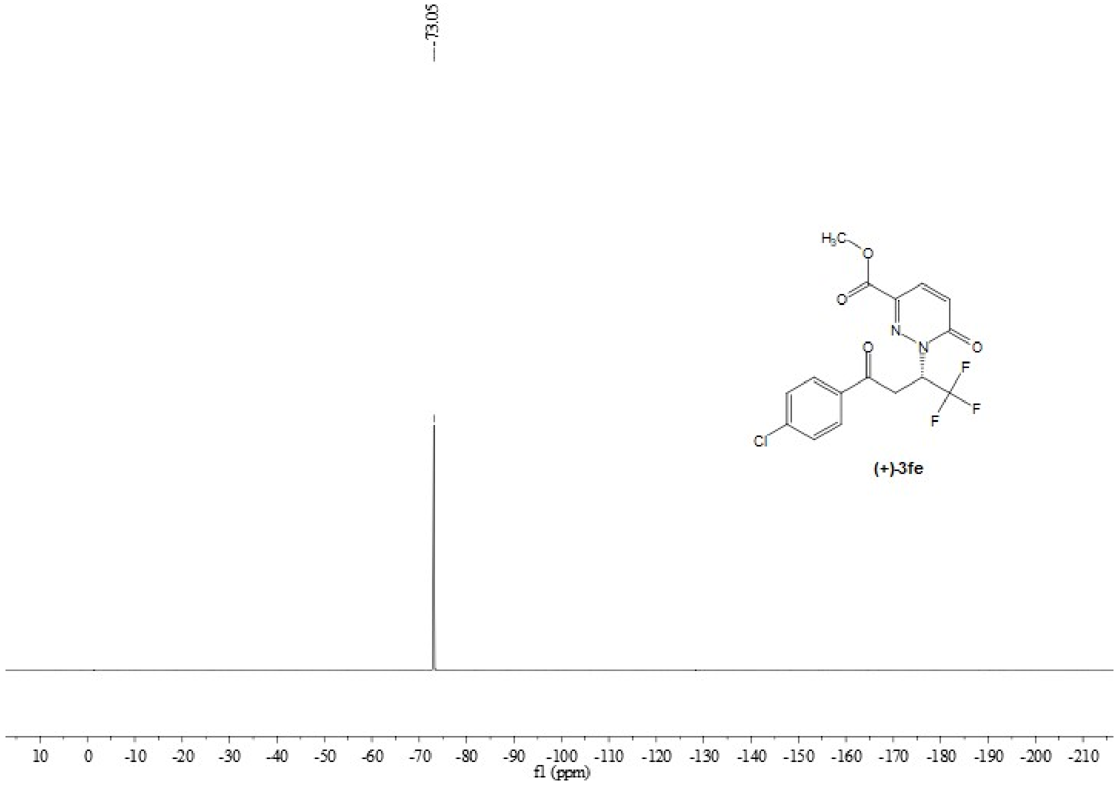
-73.05
(+)-3fe
f1 (ppm)

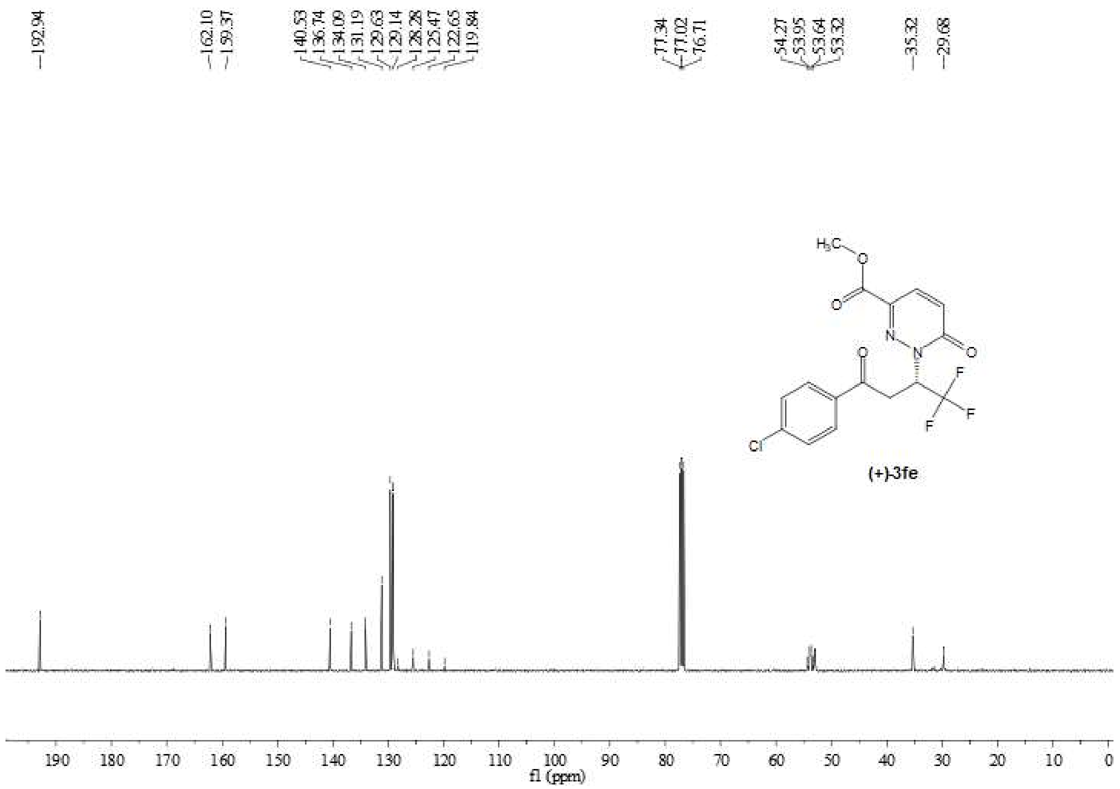
192.94
162.10
159.37
140.53
136.74
134.09
131.19
129.63
129.14
128.28
125.47
122.65
119.84
77.34
77.02
76.71
54.27
53.95
53.64
53.32
35.32
29.68
(+)-3fe
f1 (ppm)

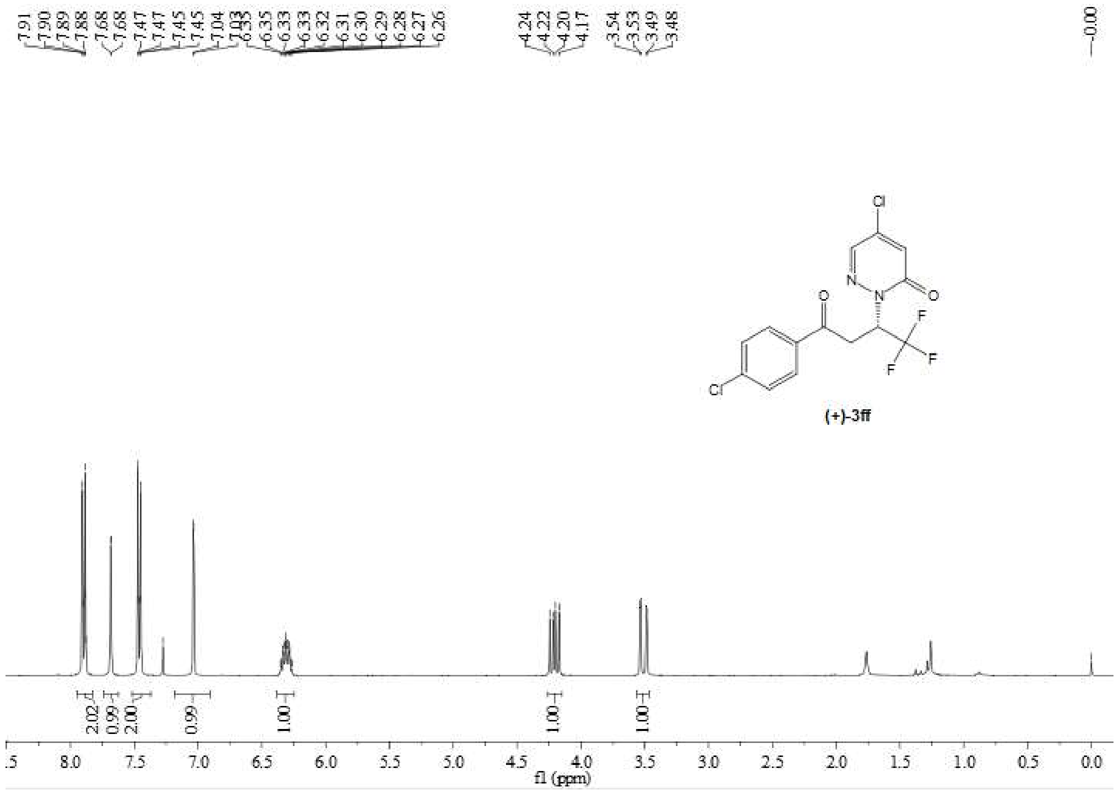
7.91
7.90
7.89
7.88
7.68
7.68
7.47
7.47
7.45
7.45
7.04
7.03
6.35
6.33
6.33
6.32
6.31
6.30
6.29
6.28
6.27
6.26
4.24
4.22
4.20
4.17
3.54
3.53
3.49
3.48
0.00
(+)-3ff
2.02
0.99
2.00
0.99
1.00
1.00
1.00
f1 (ppm)

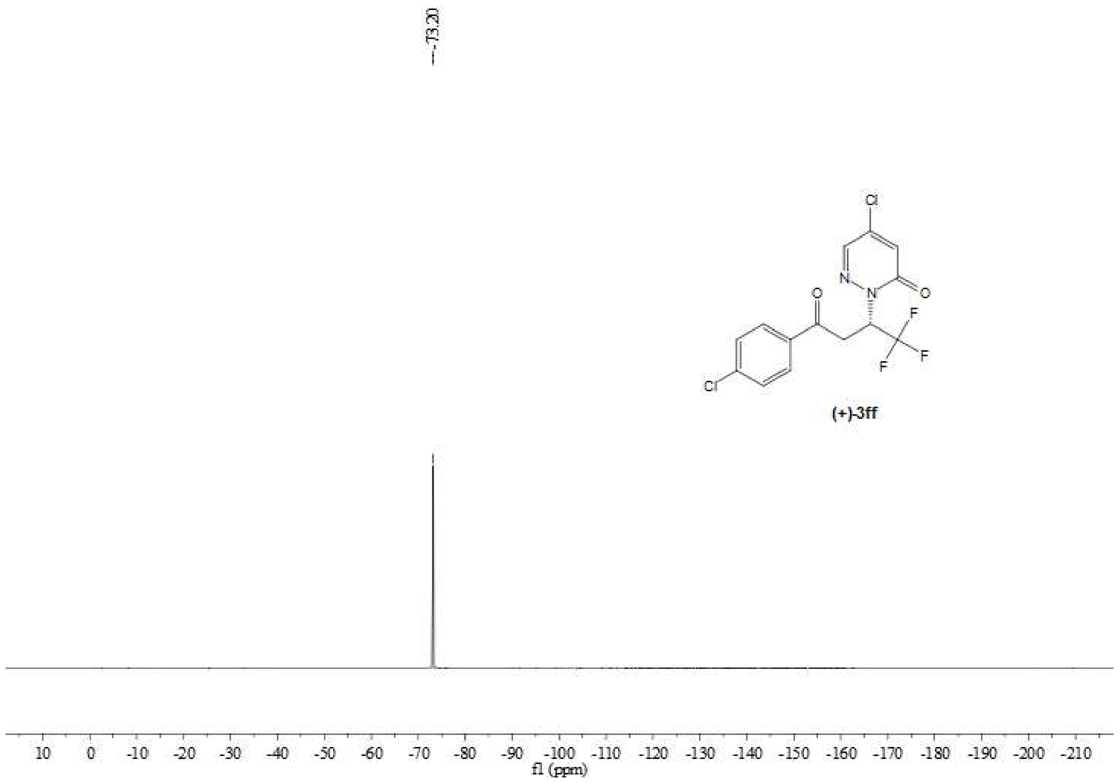
-73.20
Cl
N
N
O
O
F
F
F
Cl
(+)-3ff
10 0 -10 -20 -30 -40 -50 -60 -70 -80 -90 -100 -110 -120 -130 -140 -150 -160 -170 -180 -190 -200 -210
f1 (ppm)

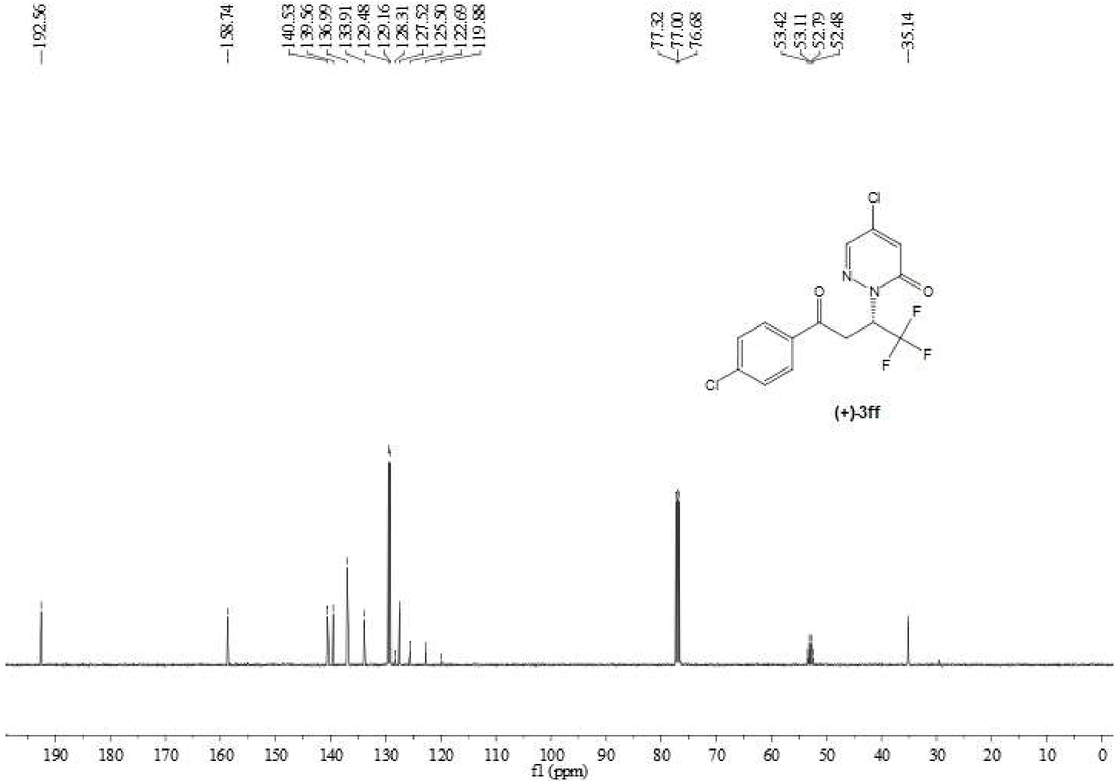
192.56
158.74
140.53
139.56
136.99
133.91
129.48
129.16
128.31
127.52
125.50
122.69
119.88
77.32
77.00
76.68
53.42
53.11
52.79
52.48
35.14
Cl
N
N
O
O
F
F
F
Cl
(+)-3ff
190 180 170 160 150 140 130 120 110 100 90 80 70 60 50 40 30 20 10 0
f1 (ppm)

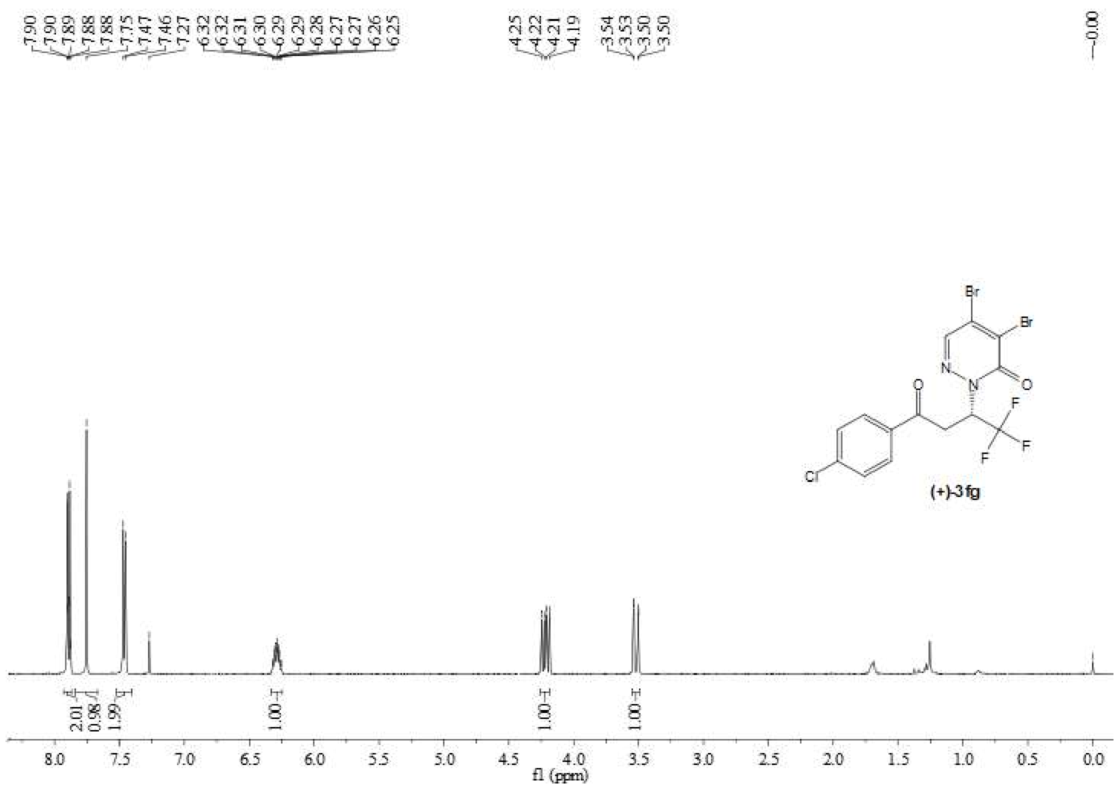

(+)-3fg
f1 (ppm)

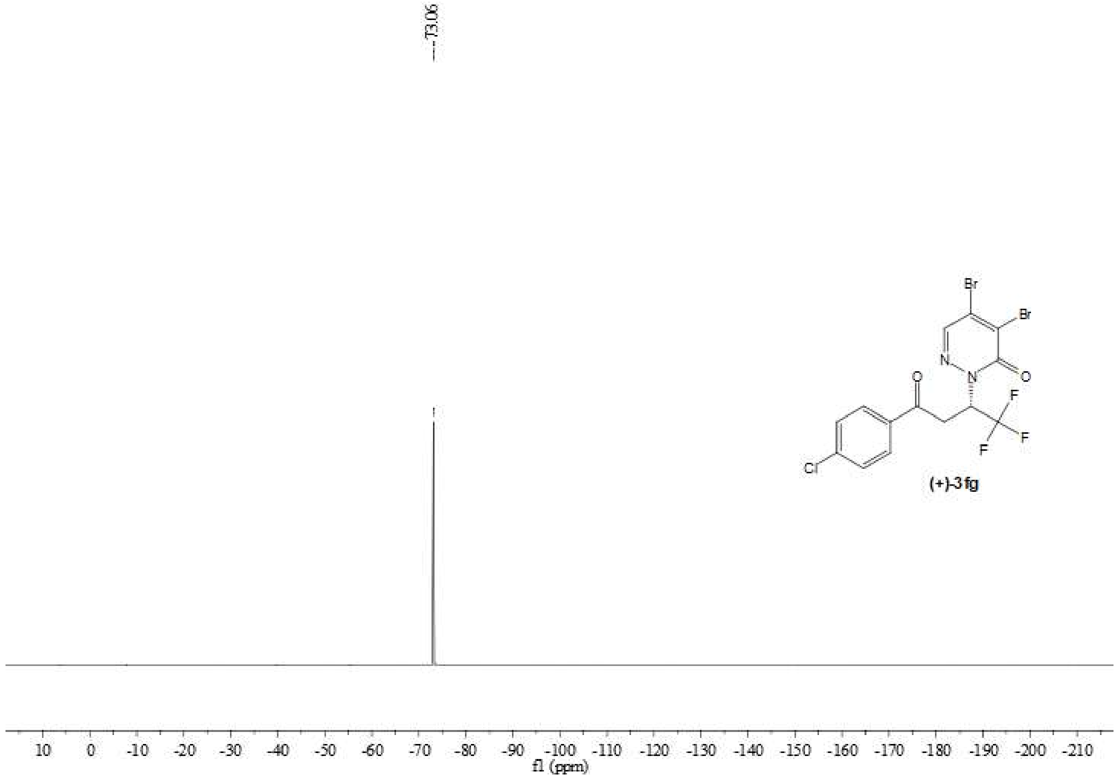

(+)-3fg
-73.06
f1 (ppm)

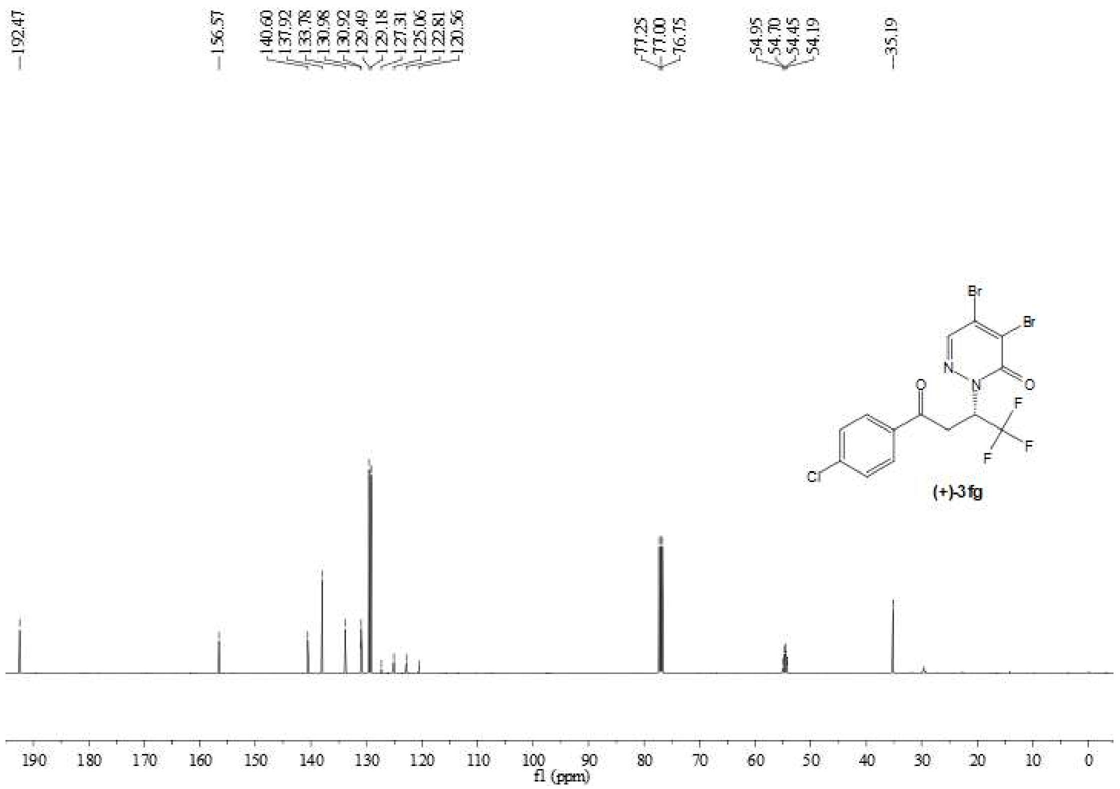
192.47
156.57
140.60
137.92
133.78
130.98
130.92
129.49
129.18
127.31
125.06
122.81
120.56
77.25
77.00
76.75
54.95
54.70
54.45
54.19
35.19
Br
Br
N
N
O
O
F
F
F
Cl
(+)-3fg
f1 (ppm)

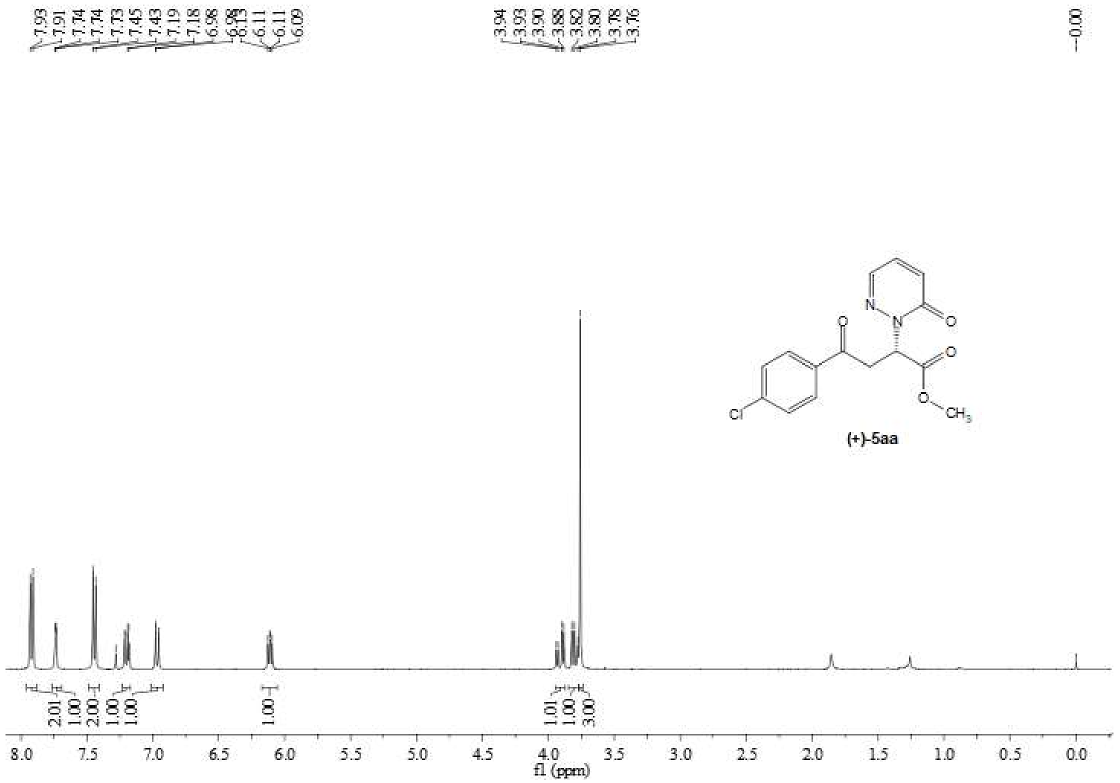
7.93
7.91
7.74
7.74
7.73
7.45
7.43
7.19
7.18
6.98
6.11
6.11
6.09
3.94
3.93
3.90
3.88
3.82
3.80
3.78
3.76
0.00
N
N
O
O
O
O
CH3
Cl
(+)-5aa
2.01
1.00
2.00
1.00
1.00
1.00
1.01
1.00
3.00
f1 (ppm)

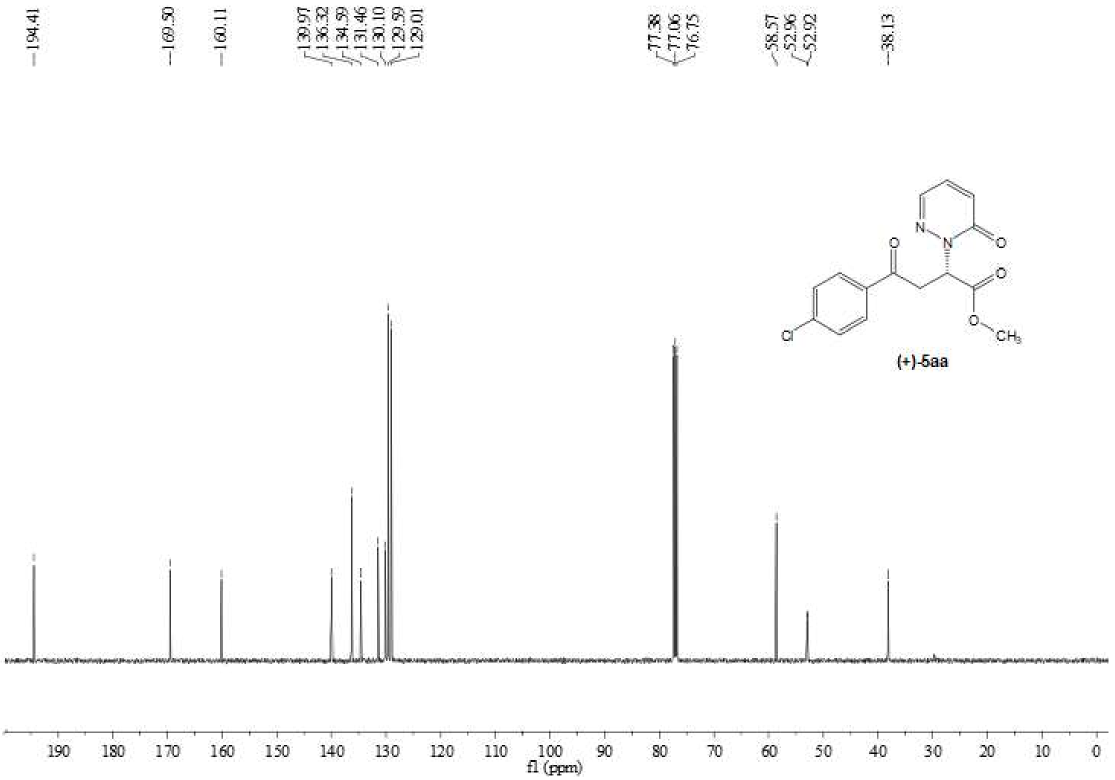

194.41
169.50
160.11
139.97
136.32
134.59
131.46
130.10
129.59
129.01
77.38
77.06
76.75
58.57
52.96
52.92
38.13
N
N
O
O
O
O
Cl
CH3
(+)-5aa
190 180 170 160 150 140 130 120 110 100 90 80 70 60 50 40 30 20 10 0
f1 (ppm)

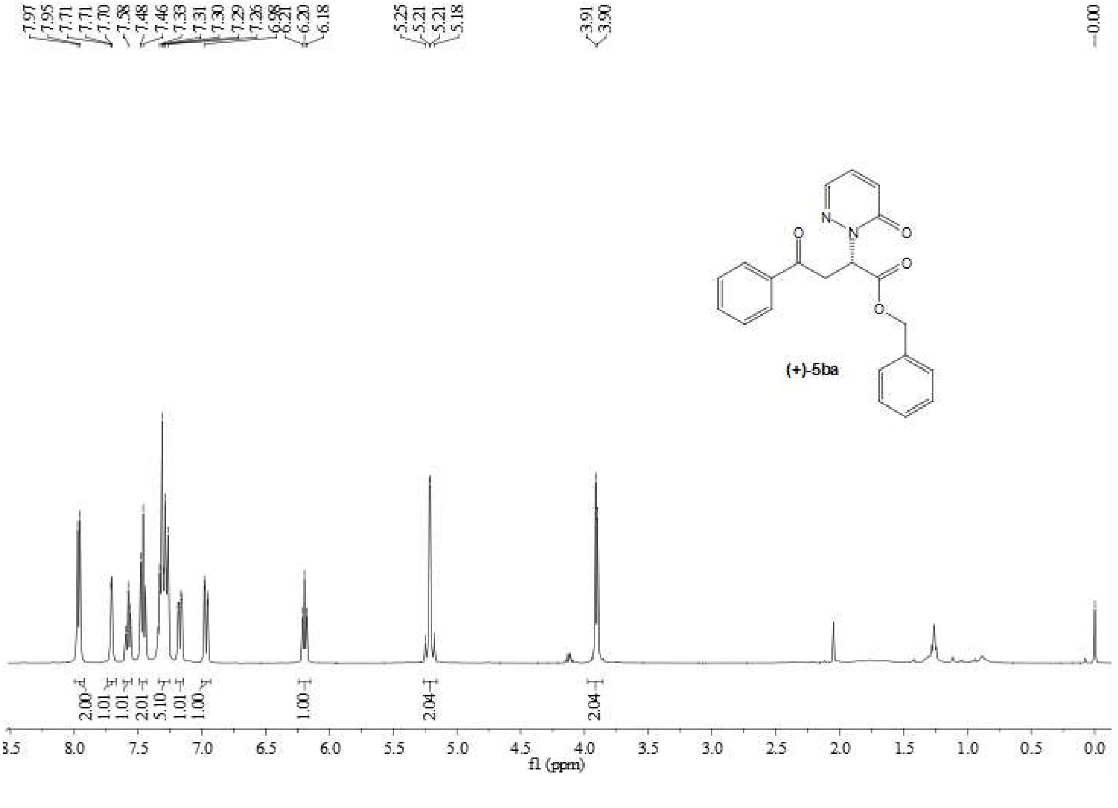

7.97
7.95
7.71
7.71
7.70
7.58
7.48
7.46
7.33
7.31
7.30
7.29
7.26
6.98
6.21
6.20
6.18
5.25
5.21
5.21
5.18
3.91
3.90
0.00
N
N
O
O
O
O
(+)-5ba
2.00
1.01
1.01
2.01
5.10
1.01
1.00
1.00
2.04
2.04
8.5 8.0 7.5 7.0 6.5 6.0 5.5 5.0 4.5 4.0 3.5 3.0 2.5 2.0 1.5 1.0 0.5 0.0
f1 (ppm)

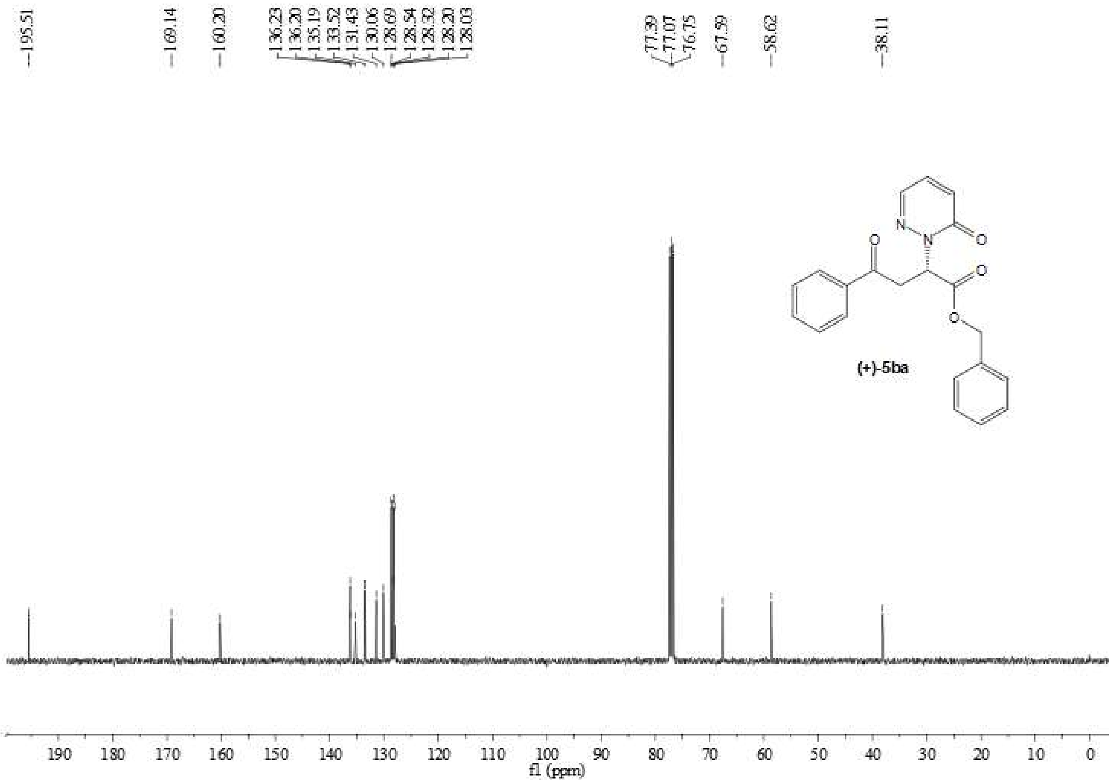
195.51
169.14
160.20
136.23
136.20
135.19
133.52
131.43
130.06
128.69
128.54
128.32
128.20
128.03
77.39
77.07
76.75
67.59
58.62
38.11
(+)-5ba
190 180 170 160 150 140 130 120 110 100 90 80 70 60 50 40 30 20 10 0
f1 (ppm)

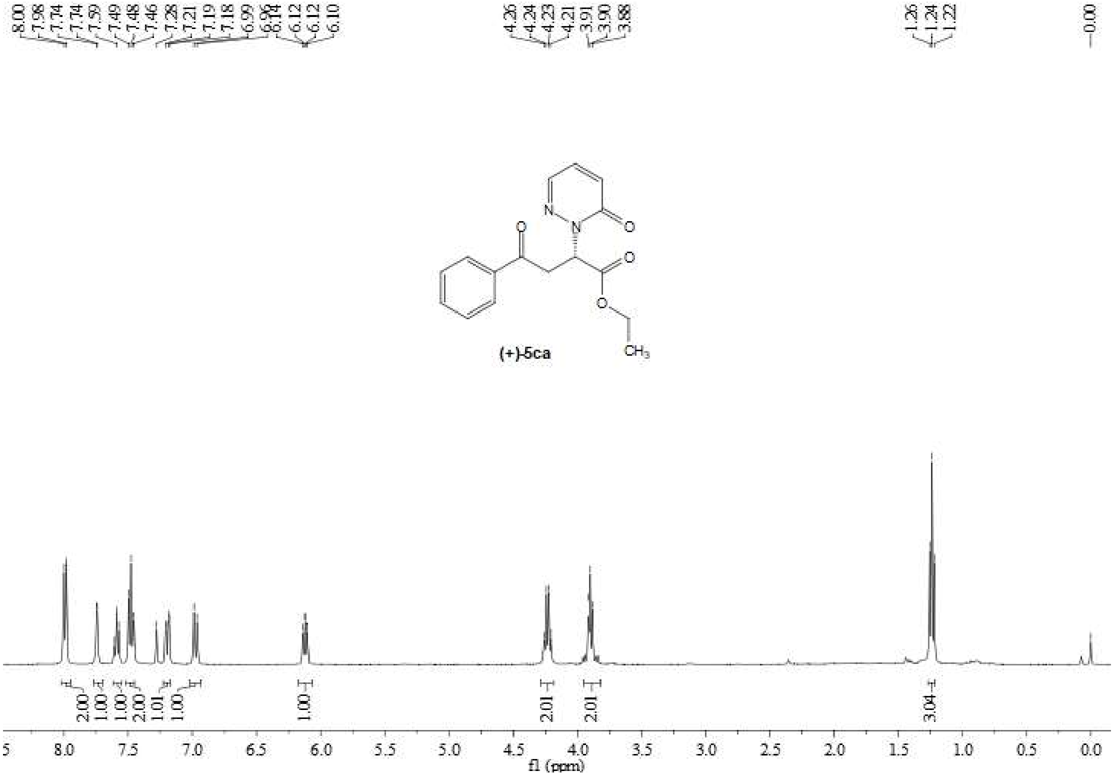
8.00
7.98
7.74
7.74
7.59
7.49
7.48
7.46
7.28
7.21
7.19
7.18
6.99
6.96
6.14
6.12
6.12
6.10
4.26
4.24
4.23
4.21
3.91
3.90
3.88
1.26
1.24
1.22
0.00
(+)-5ca
CH3
2.00
1.00
1.00
2.00
1.01
1.00
1.00
2.01
2.01
3.04
8.0 7.5 7.0 6.5 6.0 5.5 5.0 4.5 4.0 3.5 3.0 2.5 2.0 1.5 1.0 0.5 0.0
f1 (ppm)

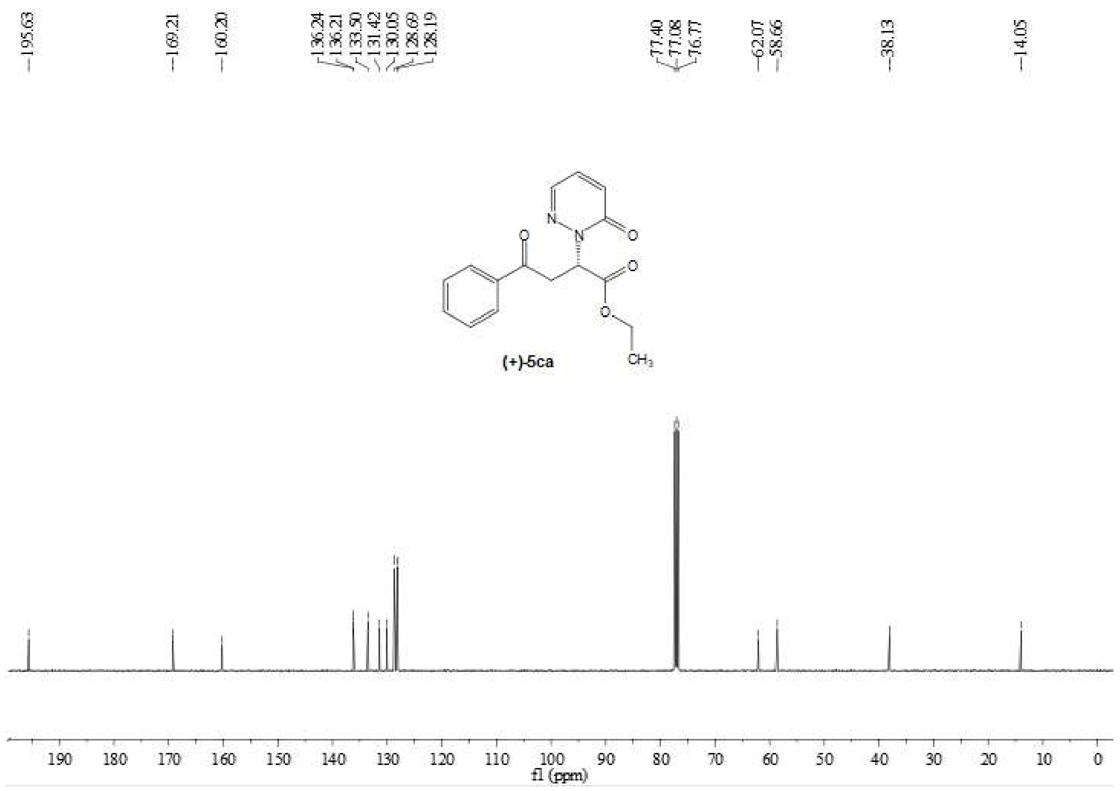
(+)-5ca
f1 (ppm)

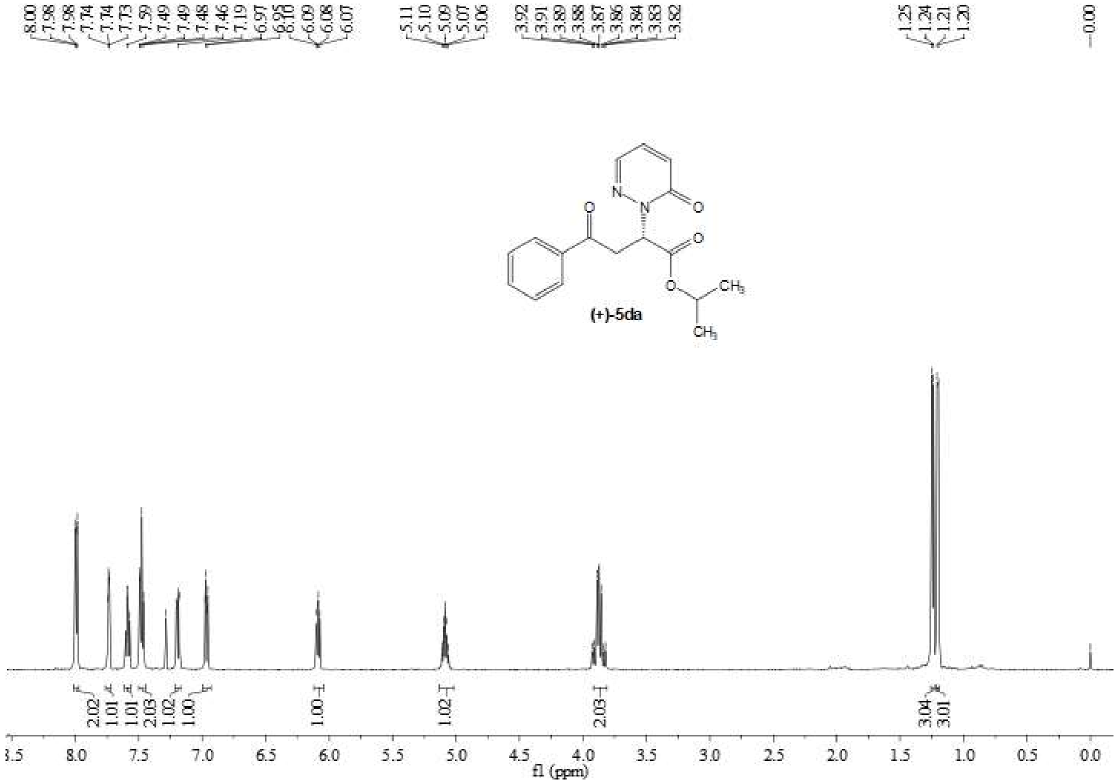
(+)-5da
f1 (ppm)

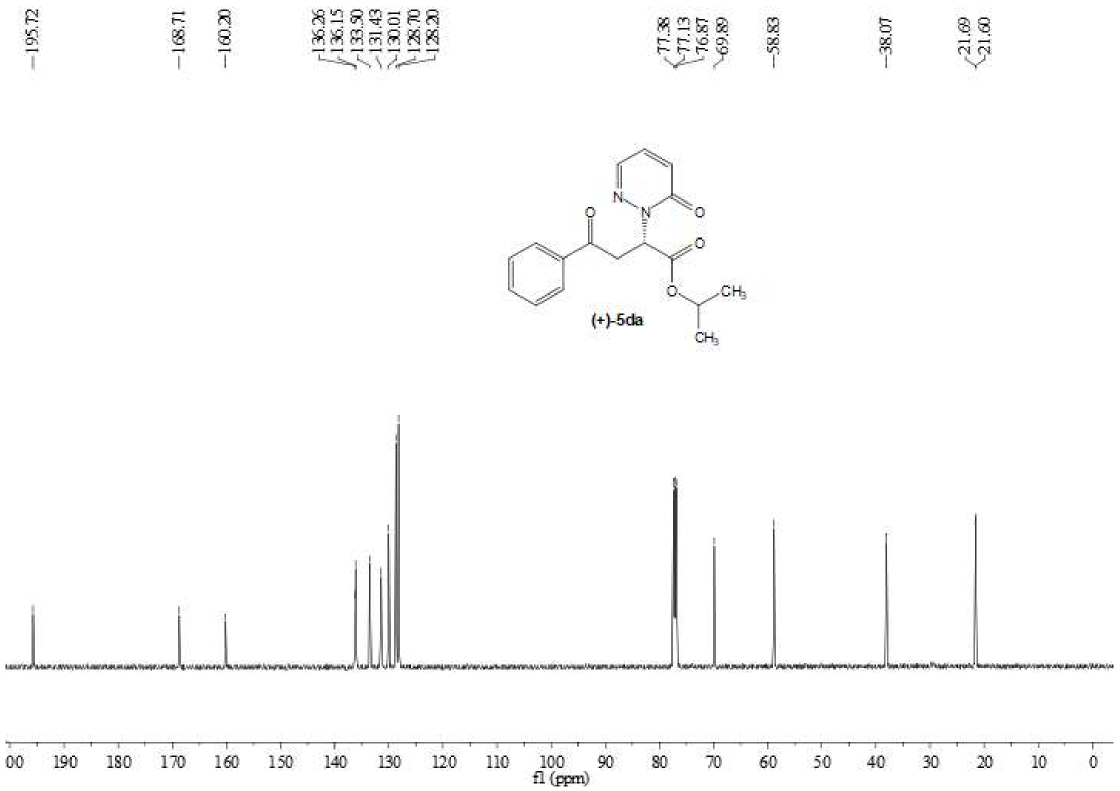
195.72
168.71
160.20
136.26
136.15
133.50
131.43
130.01
128.70
128.20
77.38
77.13
76.87
69.89
58.83
38.07
21.69
21.60
(+)-5da
f1 (ppm)

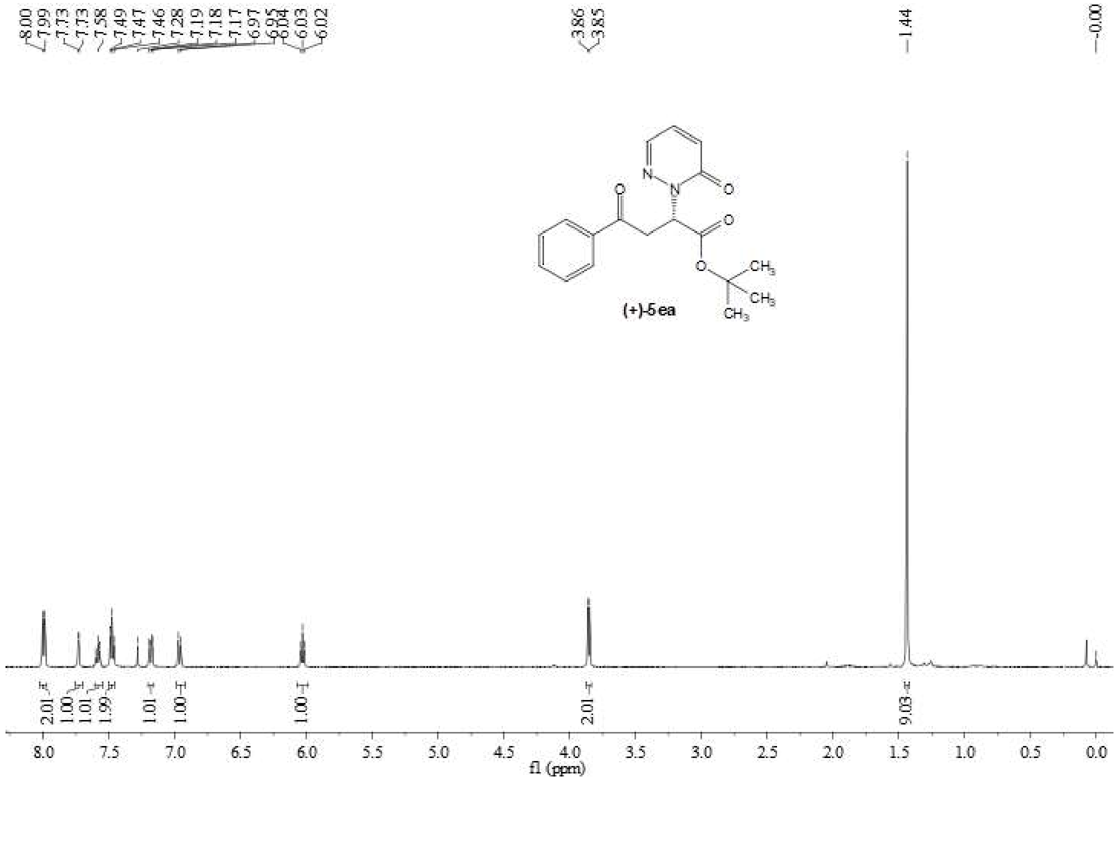
8.00
7.99
7.73
7.73
7.58
7.49
7.47
7.46
7.28
7.19
7.18
7.17
6.97
6.95
6.04
6.03
6.02
3.86
3.85
1.44
-0.00
(+)-5ea
2.01
1.00
1.01
1.99
1.01
1.00
1.00
2.01
9.03
f1 (ppm)

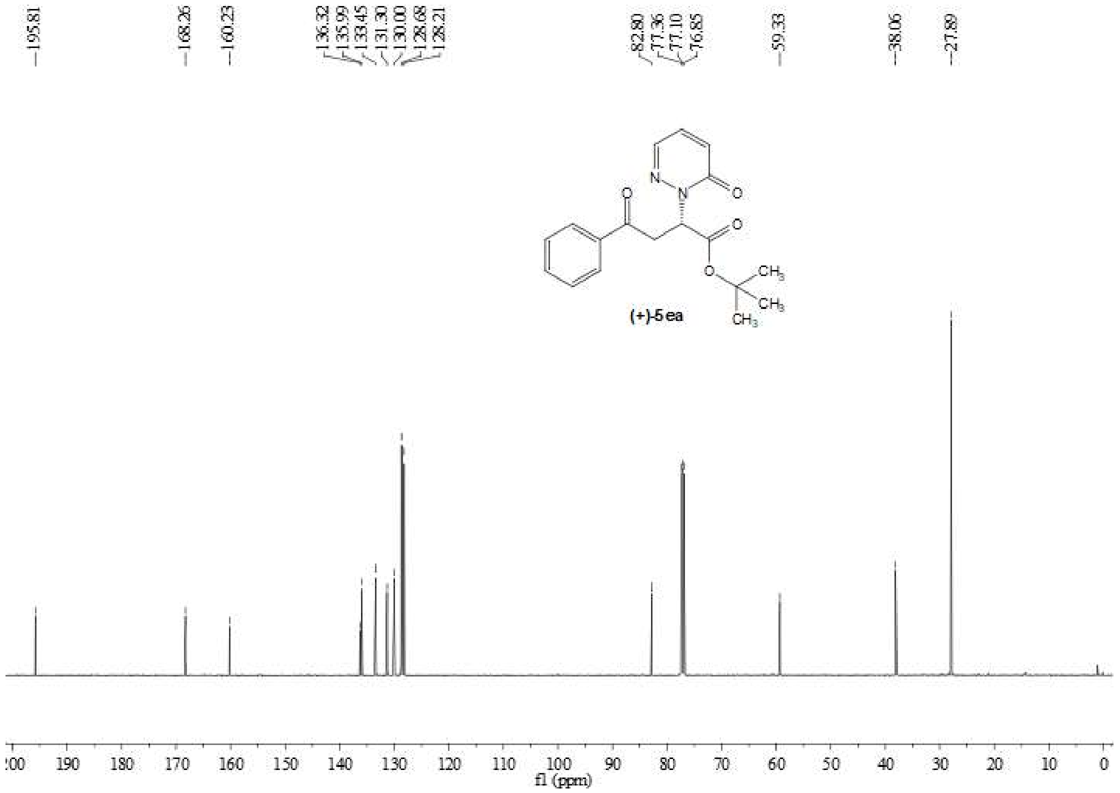
195.81
168.26
160.23
136.32
135.99
133.45
131.30
130.00
128.68
128.21
82.80
77.36
77.10
76.85
59.33
38.06
27.89
N
N
O
O
O
O
CH_3
CH_3
CH_3
(+)-5ea
f1 (ppm)

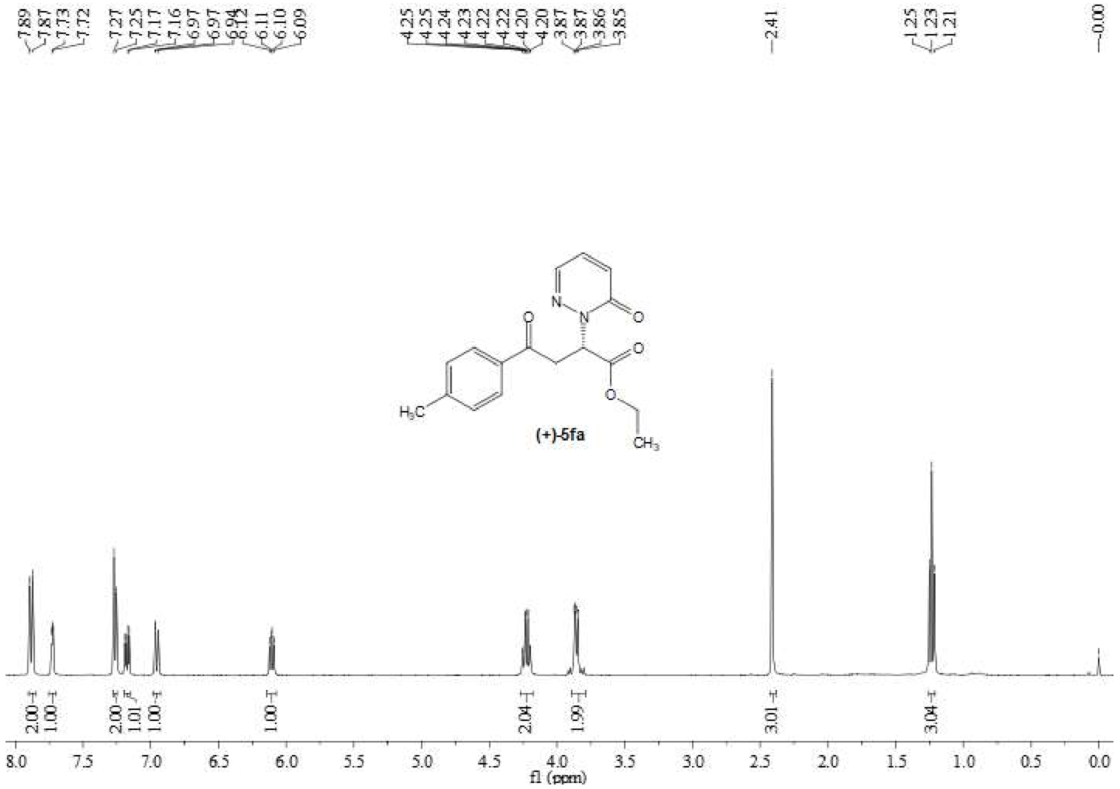
7.89
7.87
7.73
7.72
7.27
7.25
7.17
7.16
6.97
6.97
6.94
6.12
6.11
6.10
6.09
4.25
4.25
4.24
4.23
4.22
4.22
4.20
4.20
3.87
3.87
3.86
3.85
2.41
1.25
1.23
1.21
0.00
N
N
O
O
O
O
H_3C
CH_3
(+)-5fa
2.00
1.00
2.00
1.01
1.00
1.00
2.04
1.99
3.01
3.04
f1 (ppm)

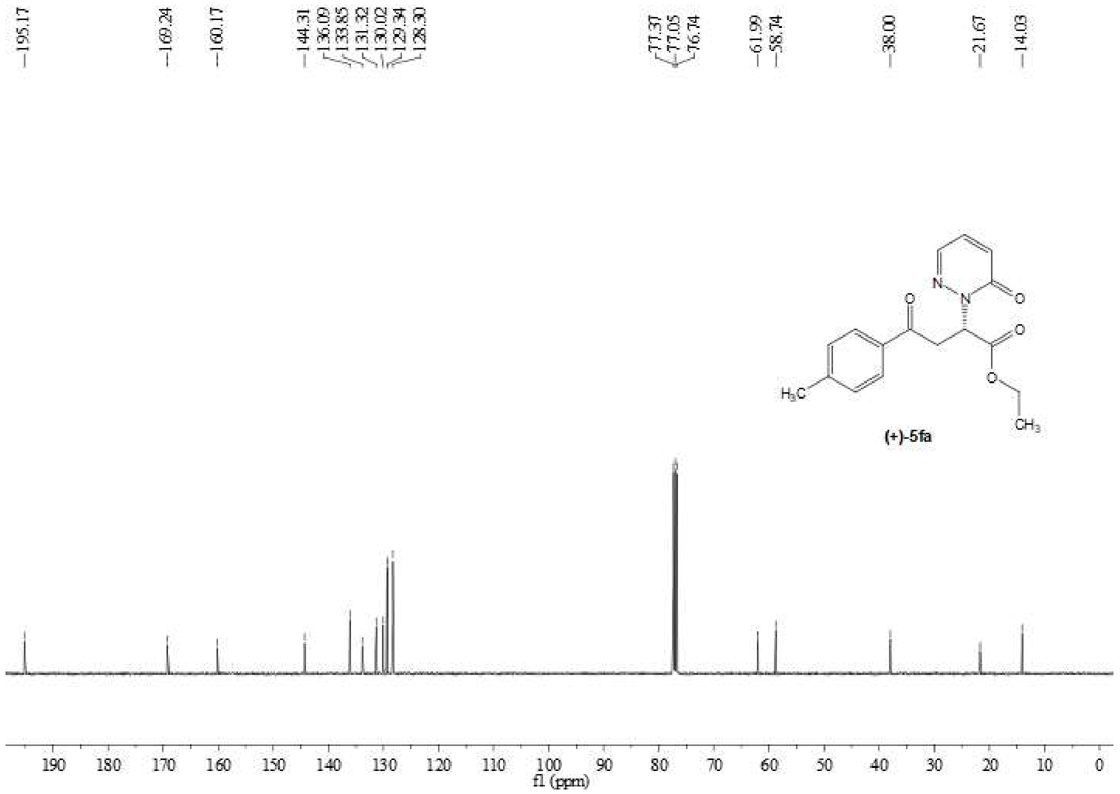
195.17
169.24
160.17
144.31
136.09
133.85
131.32
130.02
129.34
128.30
77.37
77.05
76.74
61.99
58.74
38.00
21.67
14.03
(+)-5fa
f1 (ppm)

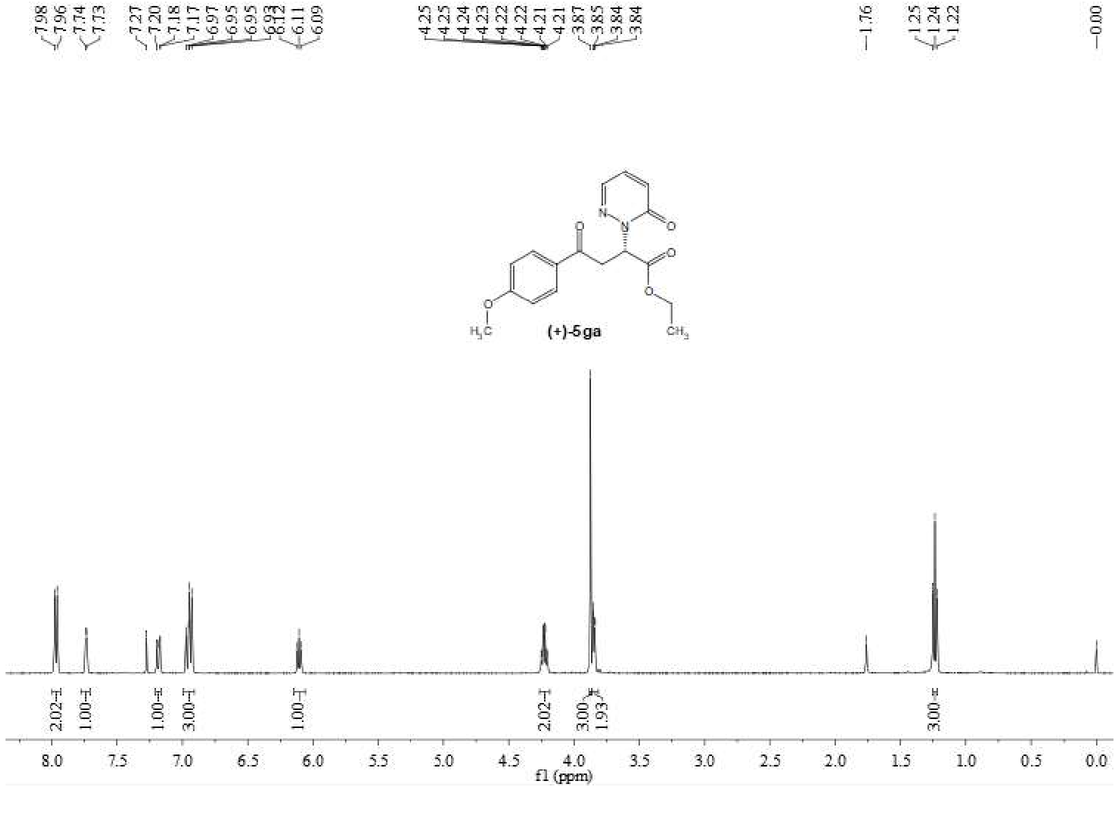
7.98
7.96
7.74
7.73
7.27
7.20
7.18
7.17
6.97
6.95
6.95
6.93
6.12
6.11
6.09
4.25
4.25
4.24
4.23
4.22
4.22
4.21
4.21
3.87
3.85
3.84
3.84
1.76
1.25
1.24
1.22
0.00
(+)-5ga
2.02
1.00
1.00
3.00
1.00
2.02
3.00
1.93
3.00
f1 (ppm)

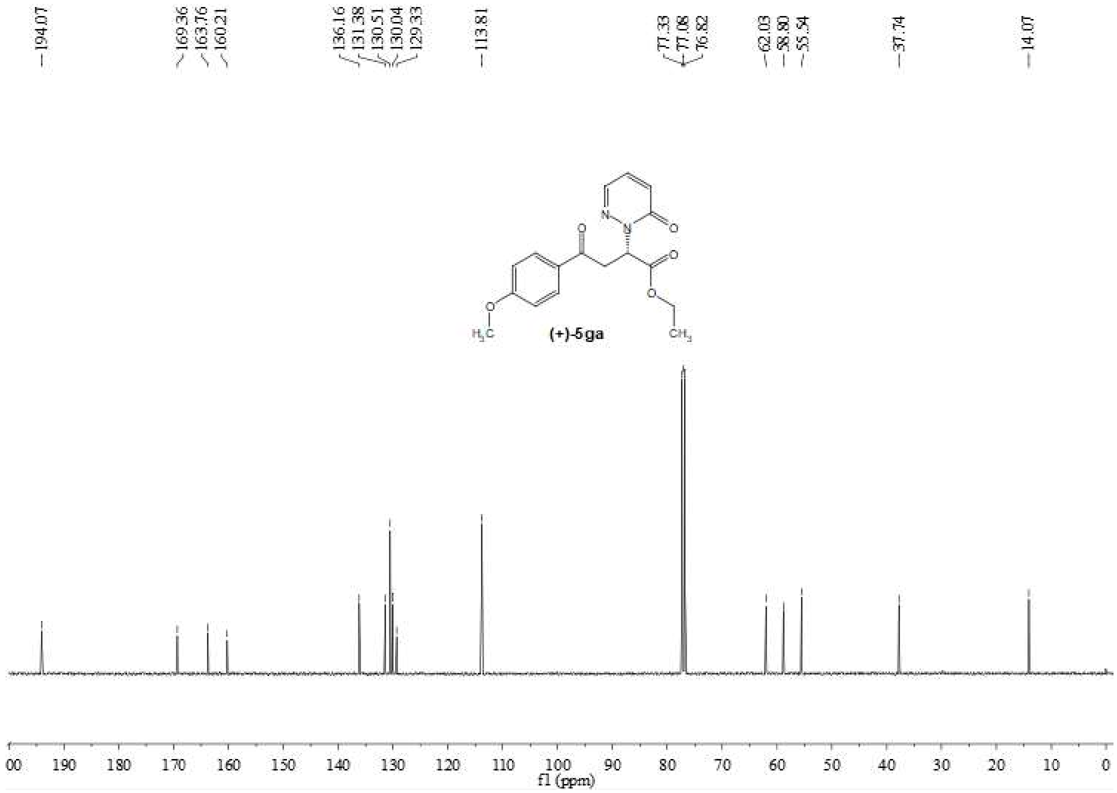
194.07
169.36
163.76
160.21
136.16
131.38
130.51
130.04
129.33
113.81
77.33
77.08
76.82
62.03
58.80
55.54
37.74
14.07
(+)-5ga
f1 (ppm)

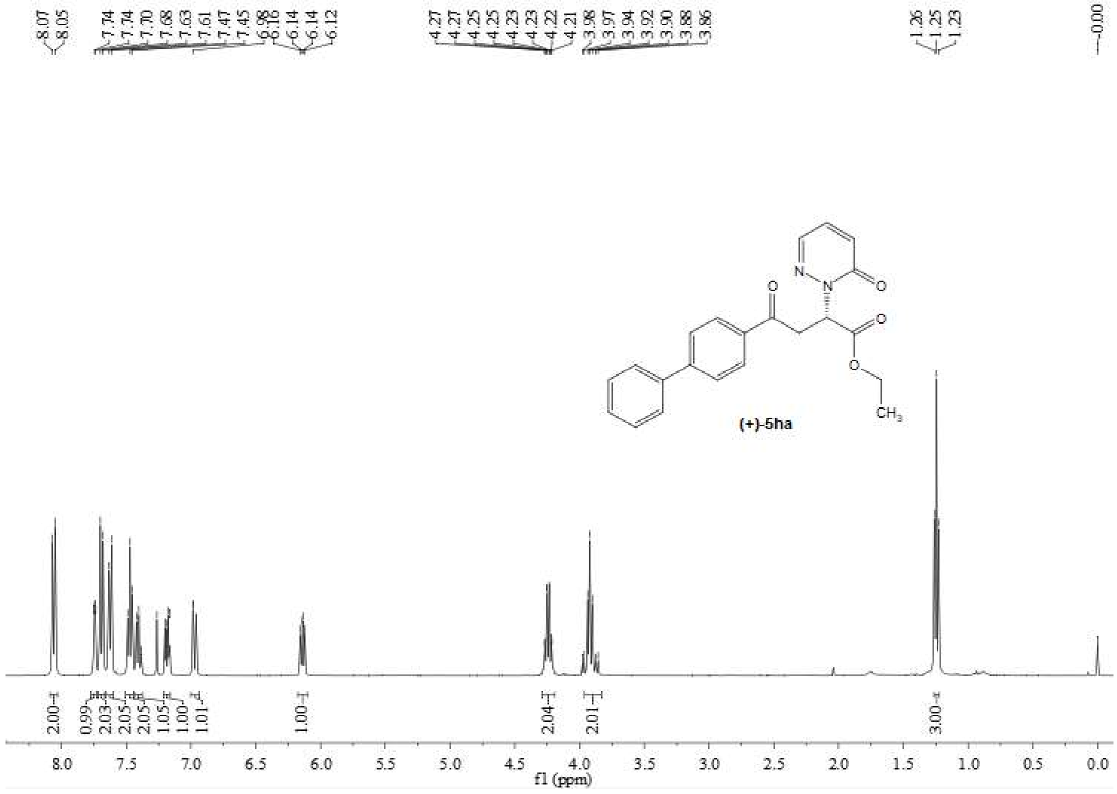
8.07
8.05
7.74
7.74
7.70
7.68
7.63
7.61
7.47
7.45
6.98
6.16
6.14
6.14
6.12
4.27
4.27
4.25
4.25
4.23
4.23
4.22
4.21
3.98
3.97
3.94
3.92
3.90
3.88
3.86
1.26
1.25
1.23
-0.00
(+)-5ha
2.00
0.99
2.03
2.05
2.05
1.05
1.00
1.01
1.00
2.04
2.01
3.00
f1 (ppm)

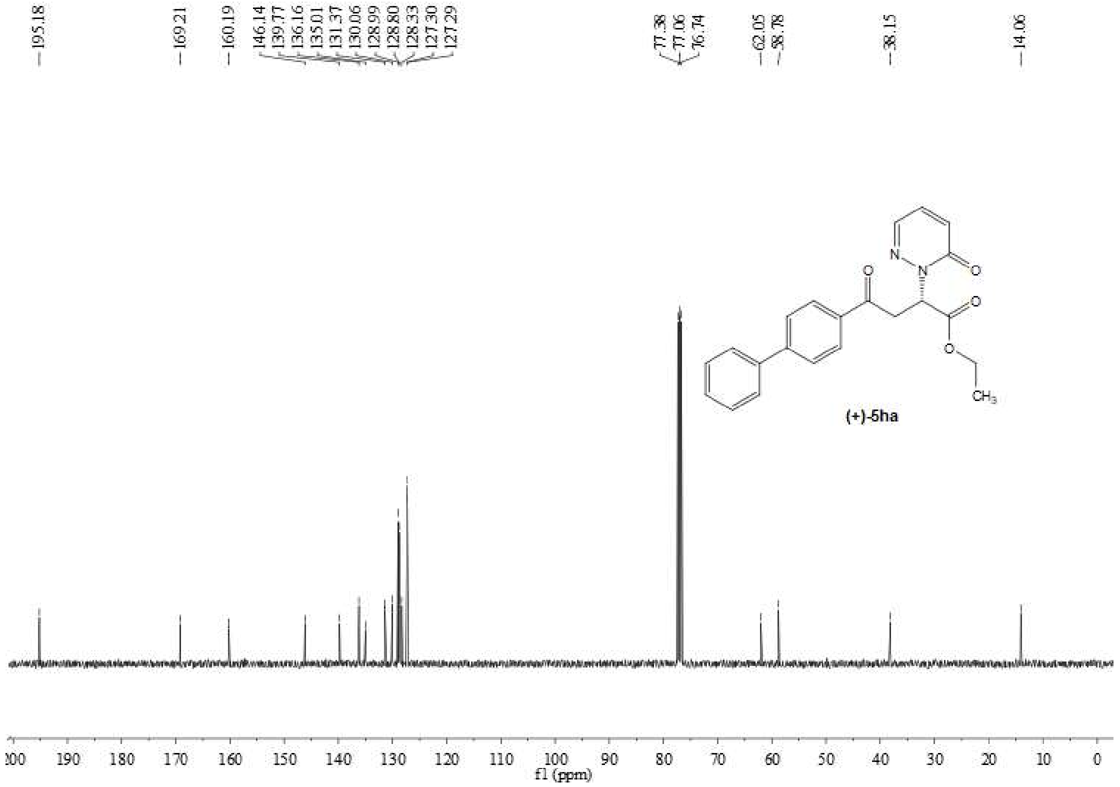

195.18
169.21
160.19
146.14
139.77
136.16
135.01
131.37
130.06
128.99
128.80
128.33
127.30
127.29
77.38
77.06
76.74
62.05
58.78
38.15
14.06
(+)-5ha
f1 (ppm)

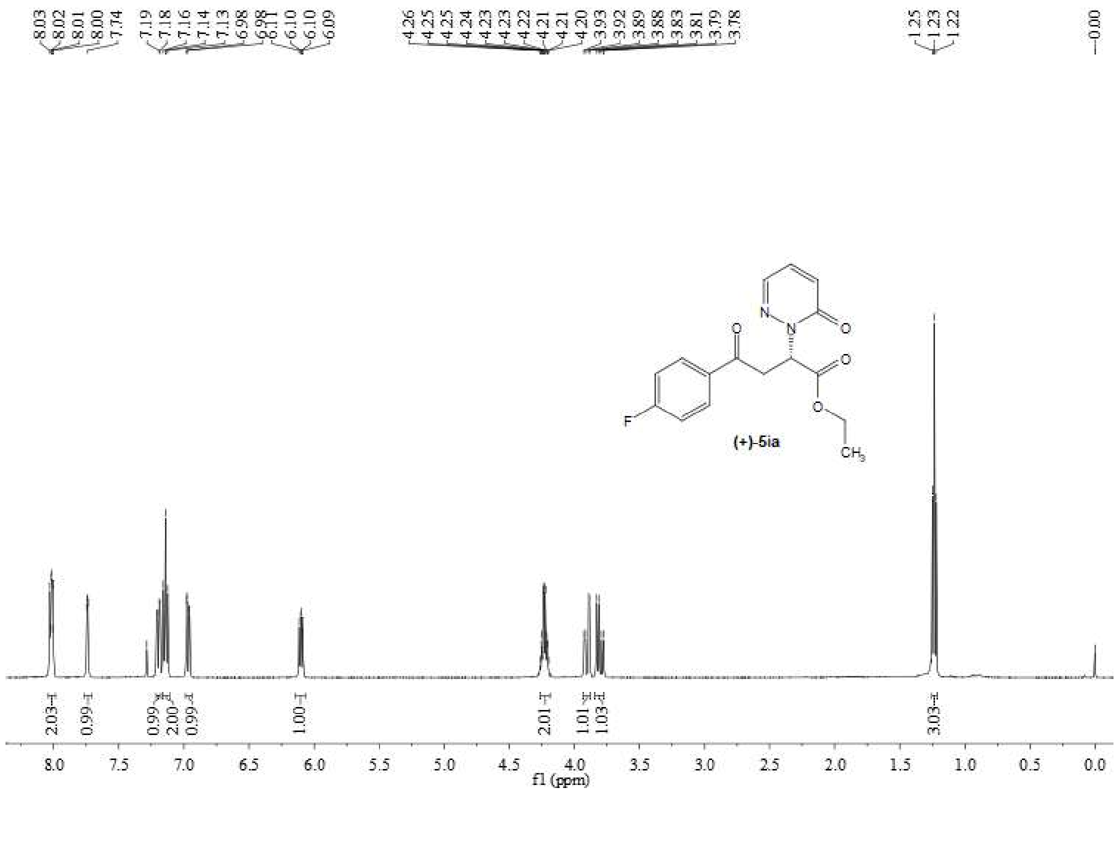

8.03
8.02
8.01
8.00
7.74
7.19
7.18
7.16
7.14
7.13
6.98
6.98
6.11
6.10
6.10
6.09
4.26
4.25
4.25
4.24
4.23
4.23
4.22
4.21
4.21
4.20
3.93
3.92
3.89
3.88
3.83
3.81
3.79
3.78
1.25
1.23
1.22
0.00
(+)-5ia
2.03
0.99
0.99
2.00
0.99
1.00
2.01
1.01
1.03
3.03
f1 (ppm)

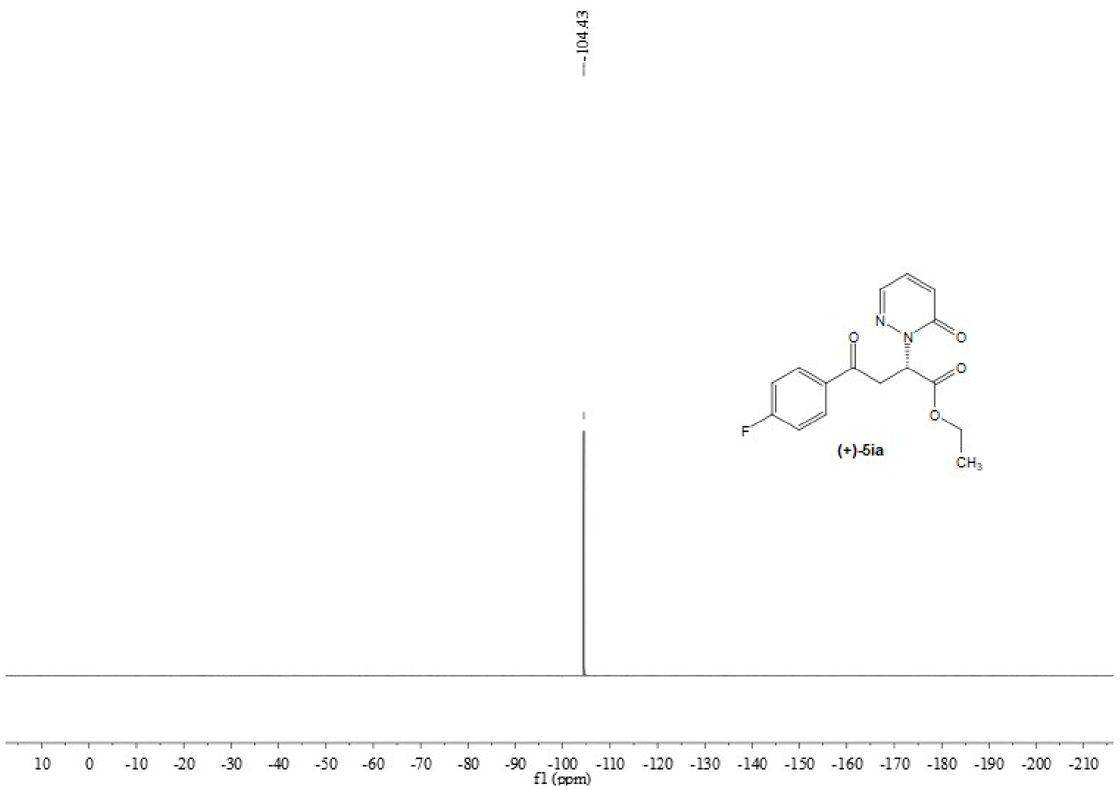

--104.43
(+)-5ia
F
O
N
CH3
10 0 -10 -20 -30 -40 -50 -60 -70 -80 -90 -100 -110 -120 -130 -140 -150 -160 -170 -180 -190 -200 -210
f1 (ppm)

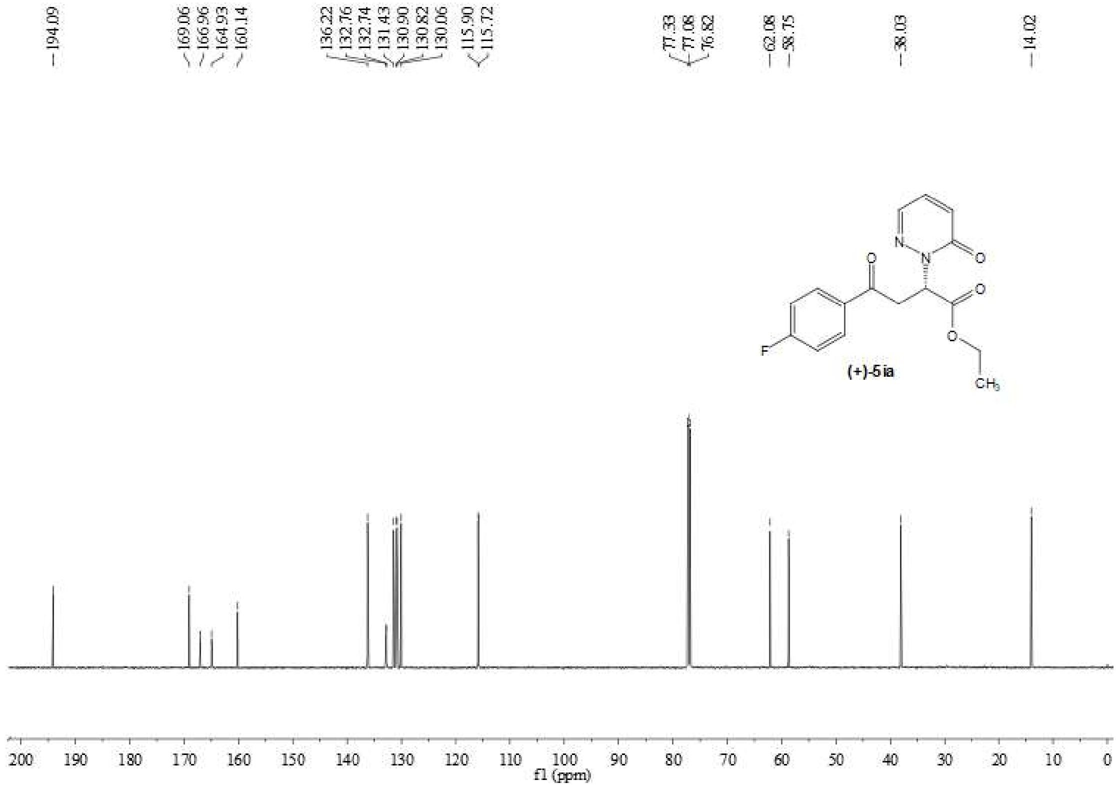

194.09
169.06
166.96
164.93
160.14
136.22
132.76
132.74
131.43
130.90
130.82
130.06
115.90
115.72
77.33
77.08
76.82
62.08
58.75
38.03
14.02
(+)-5ia
F
O
N
CH3
200 190 180 170 160 150 140 130 120 110 100 90 80 70 60 50 40 30 20 10 0
f1 (ppm)

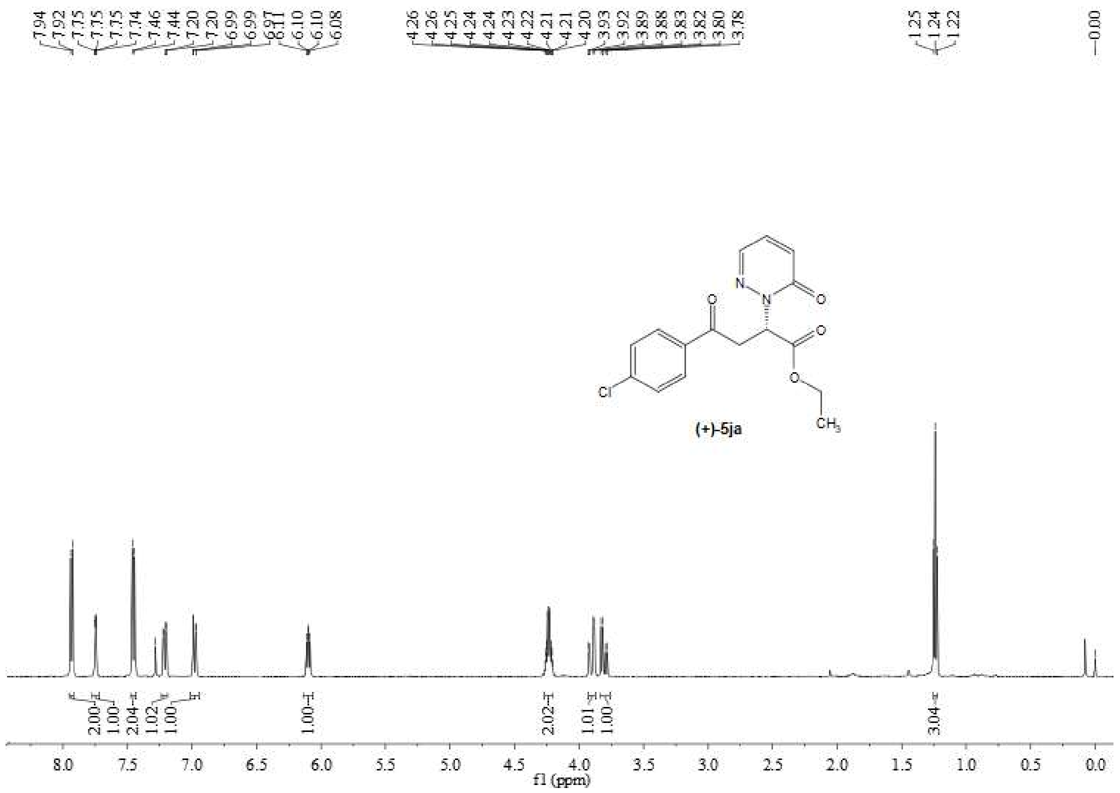
7.94
7.92
7.75
7.75
7.75
7.74
7.46
7.44
7.20
7.20
6.99
6.99
6.97
6.11
6.10
6.10
6.08
4.26
4.26
4.25
4.24
4.24
4.23
4.22
4.21
4.21
4.20
3.93
3.92
3.89
3.88
3.83
3.82
3.80
3.78
1.25
1.24
1.22
0.00
N
N
O
O
O
O
Cl
CH3
(+)-5ja
2.00
1.00
2.04
1.02
1.00
1.00
2.02
1.01
1.00
3.04
f1 (ppm)

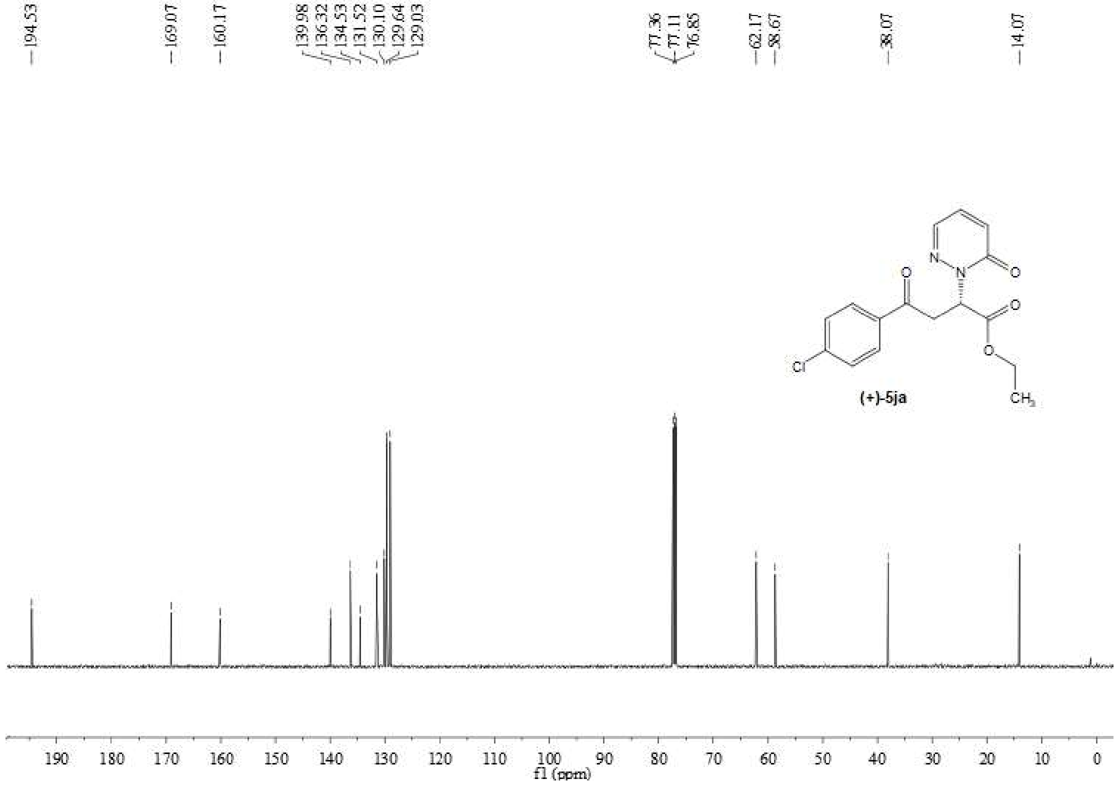
194.53
169.07
160.17
139.98
136.32
134.53
131.52
130.10
129.64
129.03
77.36
77.11
76.85
62.17
58.67
38.07
14.07
N
N
O
O
O
O
Cl
CH3
(+)-5ja
f1 (ppm)

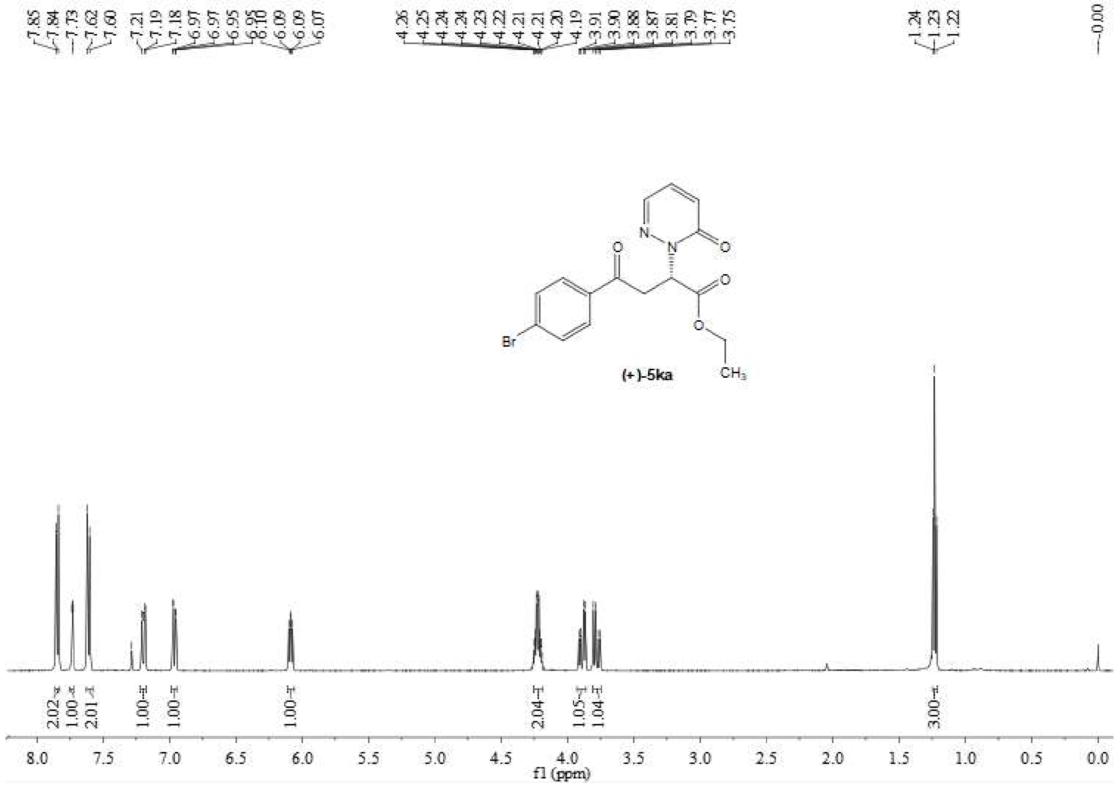

7.85 7.84 7.73 7.62 7.60 7.21 7.19 7.18 6.97 6.97 6.95 6.95 6.10 6.09 6.09 6.07
4.26 4.25 4.24 4.24 4.23 4.22 4.21 4.21 4.20 4.19 3.91 3.90 3.88 3.87 3.81 3.79 3.77 3.75
1.24 1.23 1.22
0.00
N
O
O
O
O
Br
CH3
(+)-5ka
2.02 1.00 2.01 1.00 1.00 1.00 2.04 1.05 1.04 3.00
8.0 7.5 7.0 6.5 6.0 5.5 5.0 4.5 4.0 3.5 3.0 2.5 2.0 1.5 1.0 0.5 0.0
f1 (ppm)

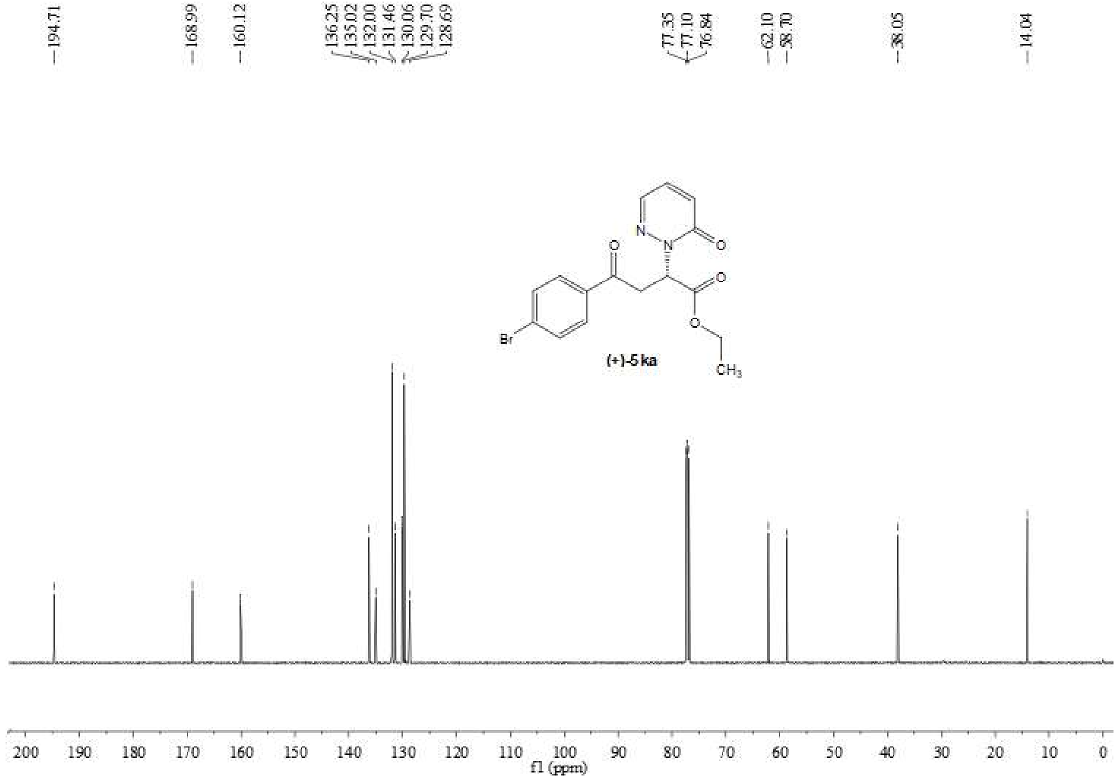

194.71
168.99
160.12
136.25 135.02 132.00 131.46 130.06 129.70 128.69
77.35 77.10 76.84
62.10 58.70
38.05
14.04
N
O
O
O
O
Br
CH3
(+)-5ka
200 190 180 170 160 150 140 130 120 110 100 90 80 70 60 50 40 30 20 10 0
f1 (ppm)

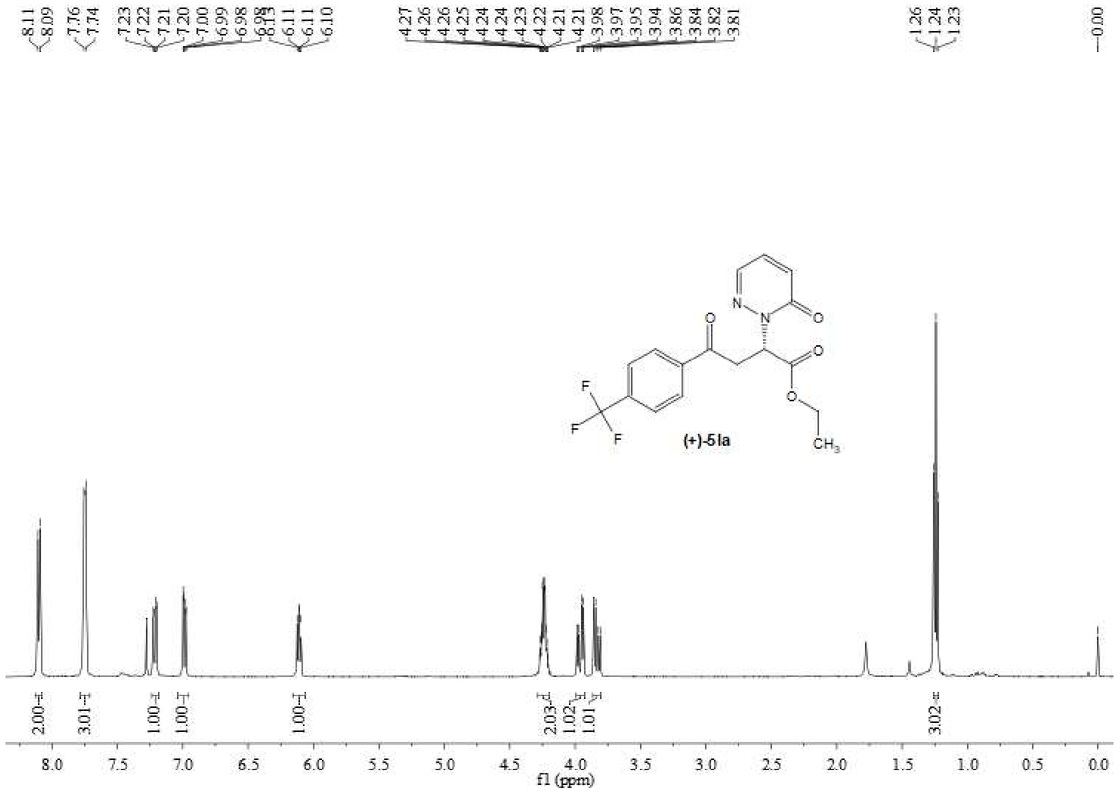

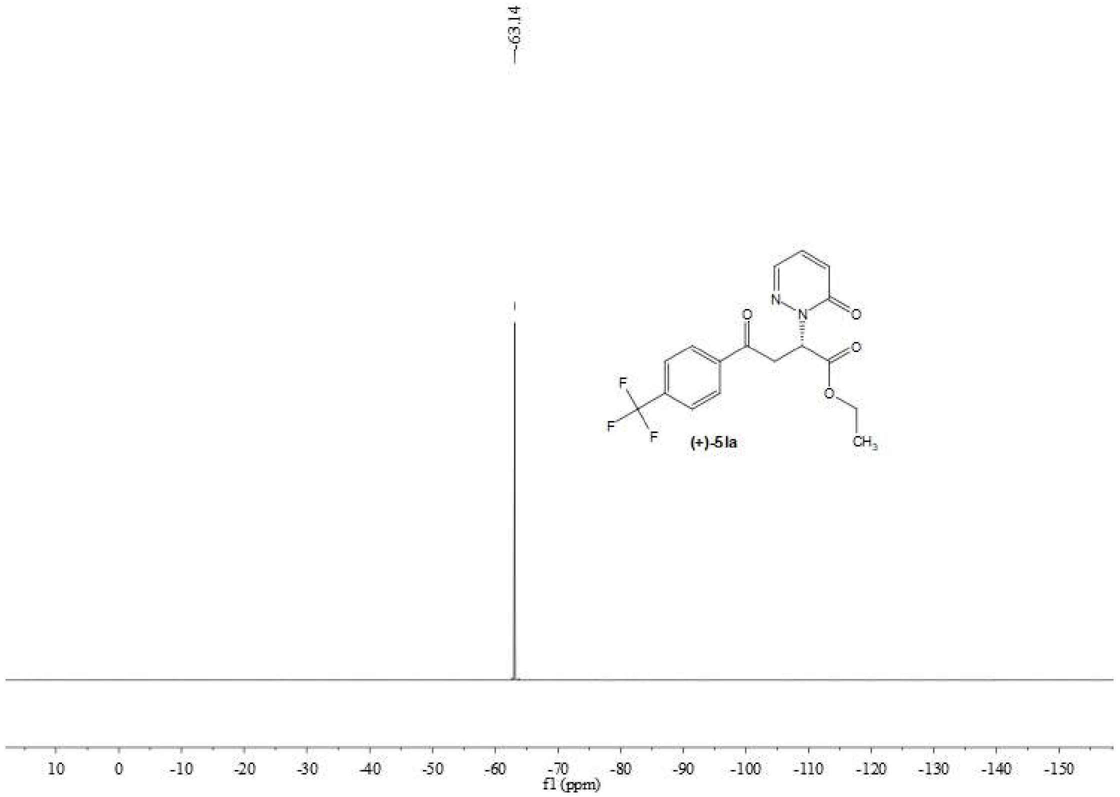

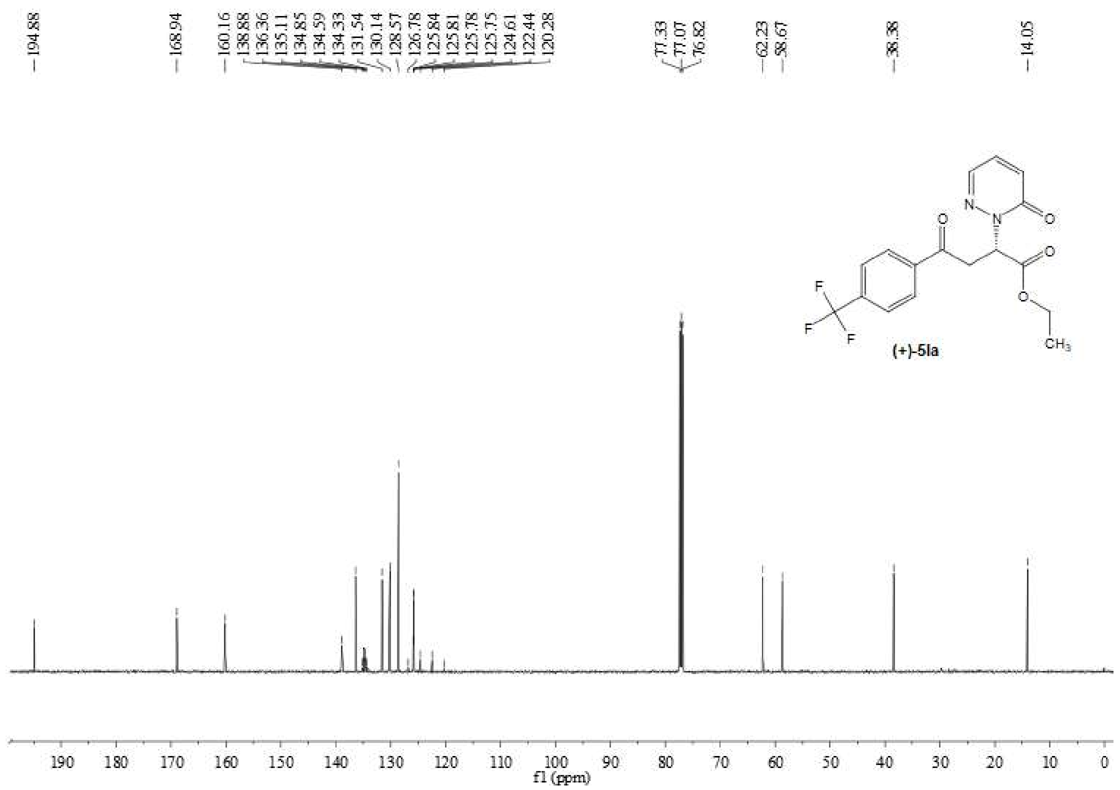
194.88
168.94
160.16
138.88
136.36
135.11
134.85
134.59
134.33
131.54
130.14
128.57
126.78
125.84
125.81
125.78
125.75
124.61
122.44
120.28
77.33
77.07
76.82
62.23
58.67
38.38
14.05
F
F
F
N
N
O
O
O
O
CH3
(+)-5la
f1 (ppm)

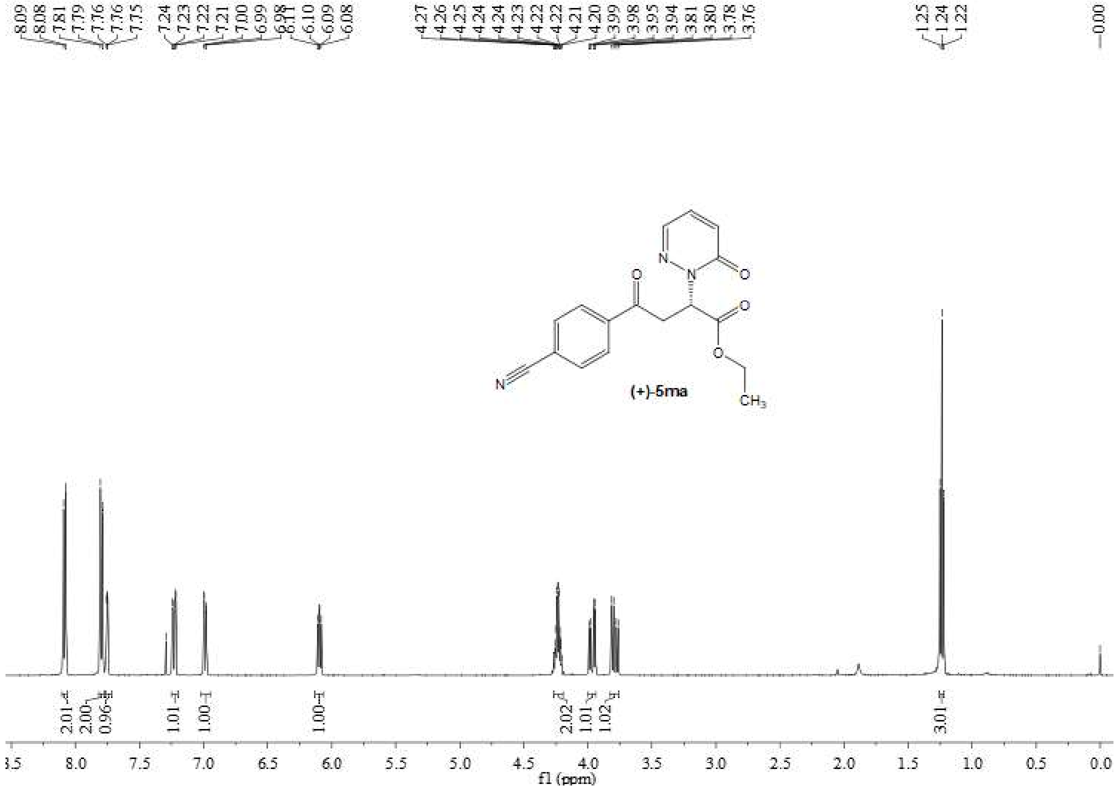
8.09
8.08
7.81
7.79
7.76
7.76
7.75
7.24
7.23
7.22
7.21
7.00
6.99
6.98
6.11
6.10
6.09
6.08
4.27
4.26
4.25
4.24
4.24
4.23
4.22
4.22
4.21
4.20
3.99
3.98
3.95
3.94
3.81
3.80
3.78
3.76
1.25
1.24
1.22
0.00
N
N
O
O
O
O
N
CH3
(+)-5ma
2.01
2.00
0.96
1.01
1.00
1.00
2.02
1.01
1.02
3.01
f1 (ppm)

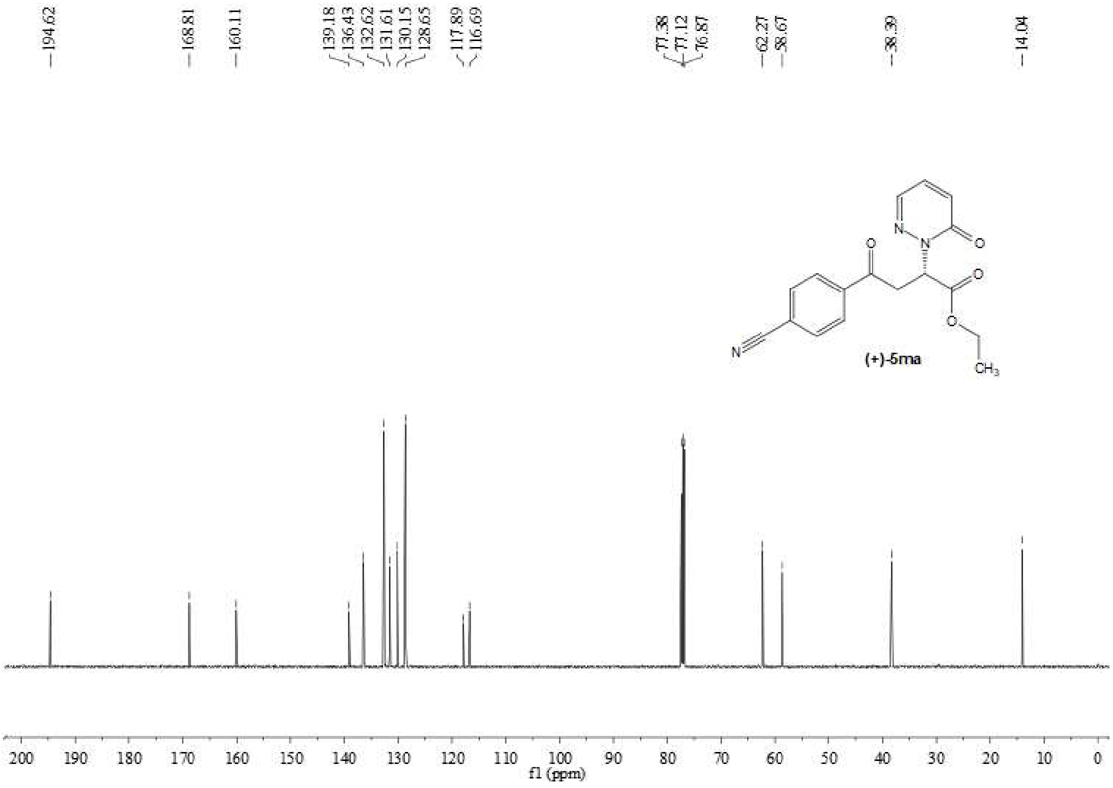
194.62
168.81
160.11
139.18
136.43
132.62
131.61
130.15
128.65
117.89
116.69
77.38
77.12
76.87
62.27
58.67
38.39
14.04
(+)-5ma
f1 (ppm)

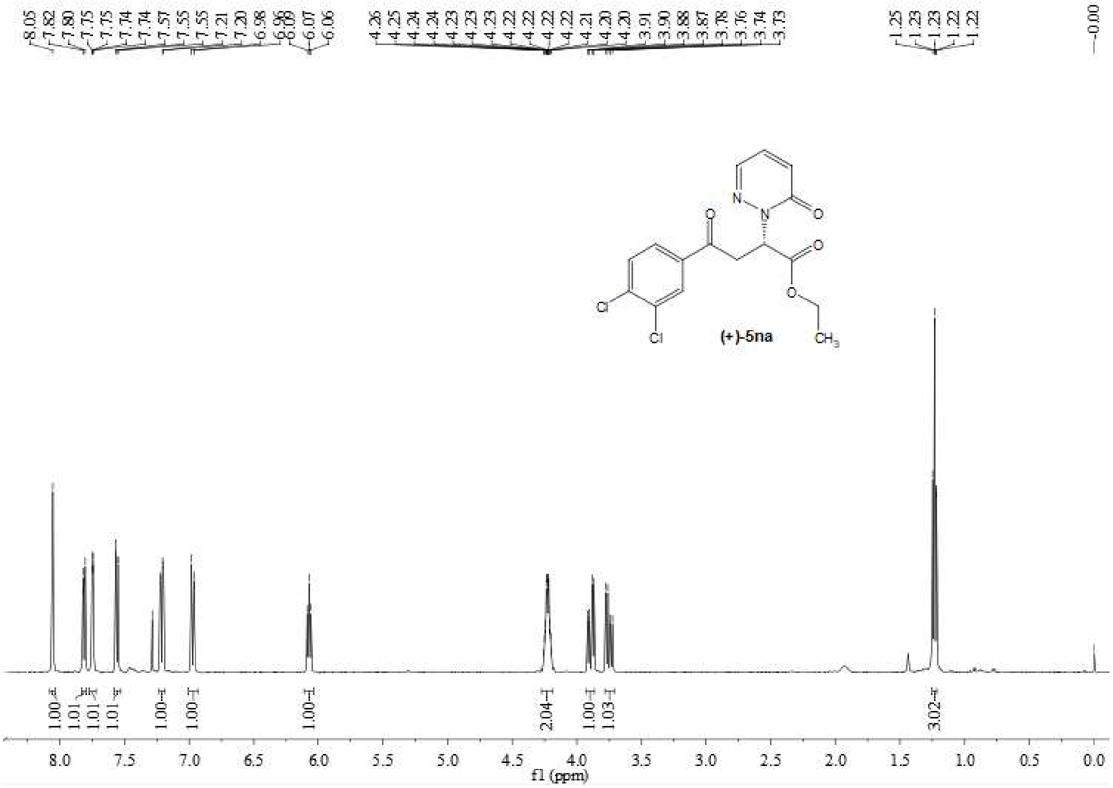
8.05
7.82
7.80
7.75
7.75
7.74
7.74
7.57
7.55
7.55
7.21
7.20
6.98
6.96
6.08
6.07
6.06
4.26
4.25
4.24
4.24
4.23
4.23
4.23
4.22
4.22
4.22
4.22
4.22
4.21
4.20
4.20
3.91
3.90
3.88
3.87
3.78
3.76
3.74
3.73
1.25
1.23
1.23
1.22
1.22
0.00
(+)-5na
1.00
1.01
1.01
1.01
1.00
1.00
1.00
2.04
1.00
1.03
3.02
f1 (ppm)

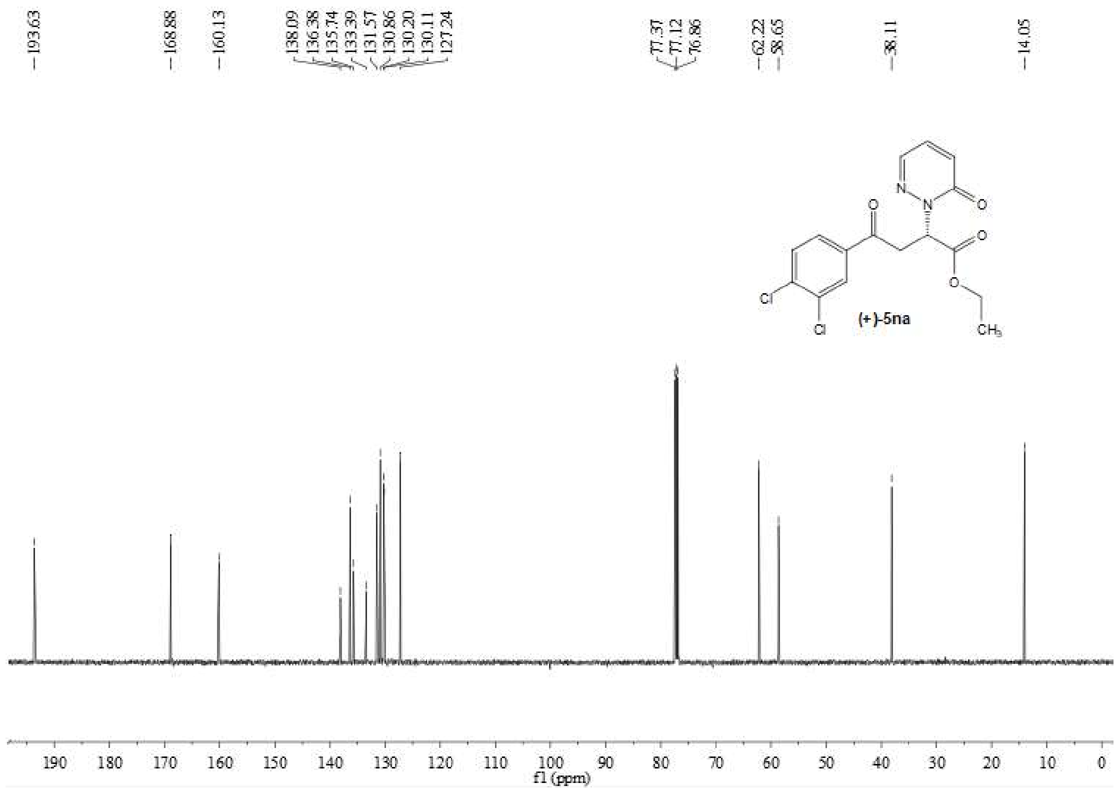
(+)-5na
f1 (ppm)

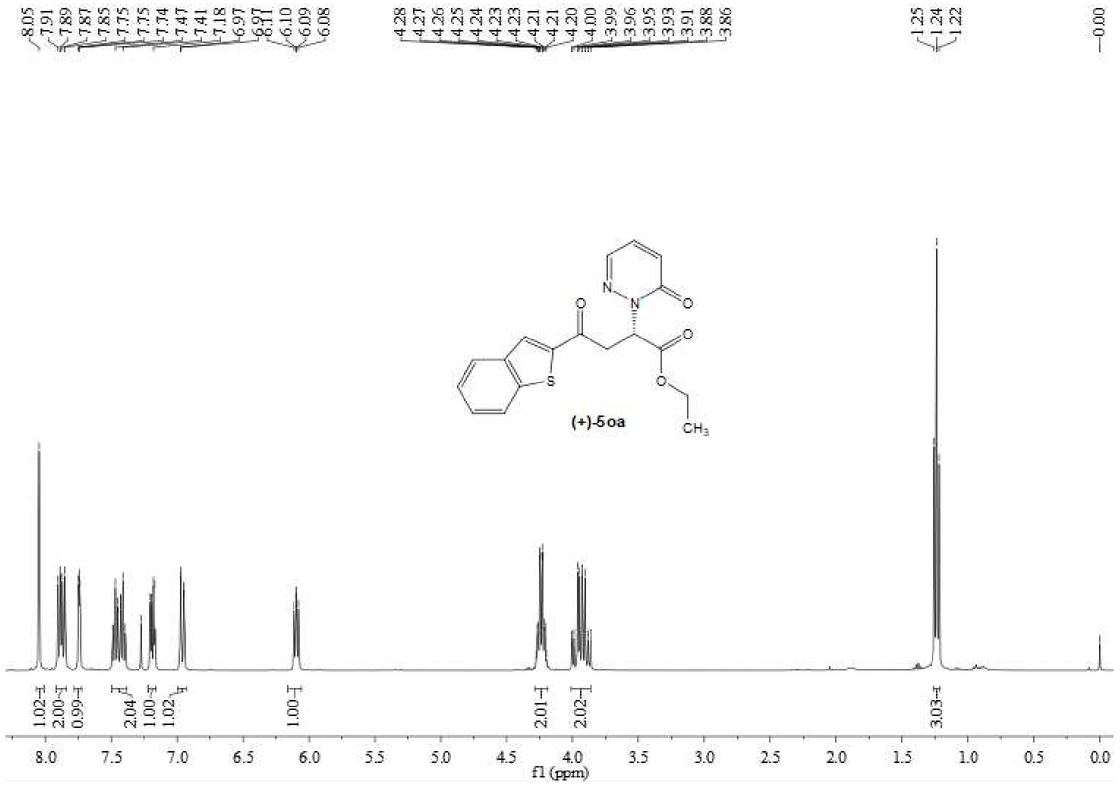
(+)-5oa
f1 (ppm)

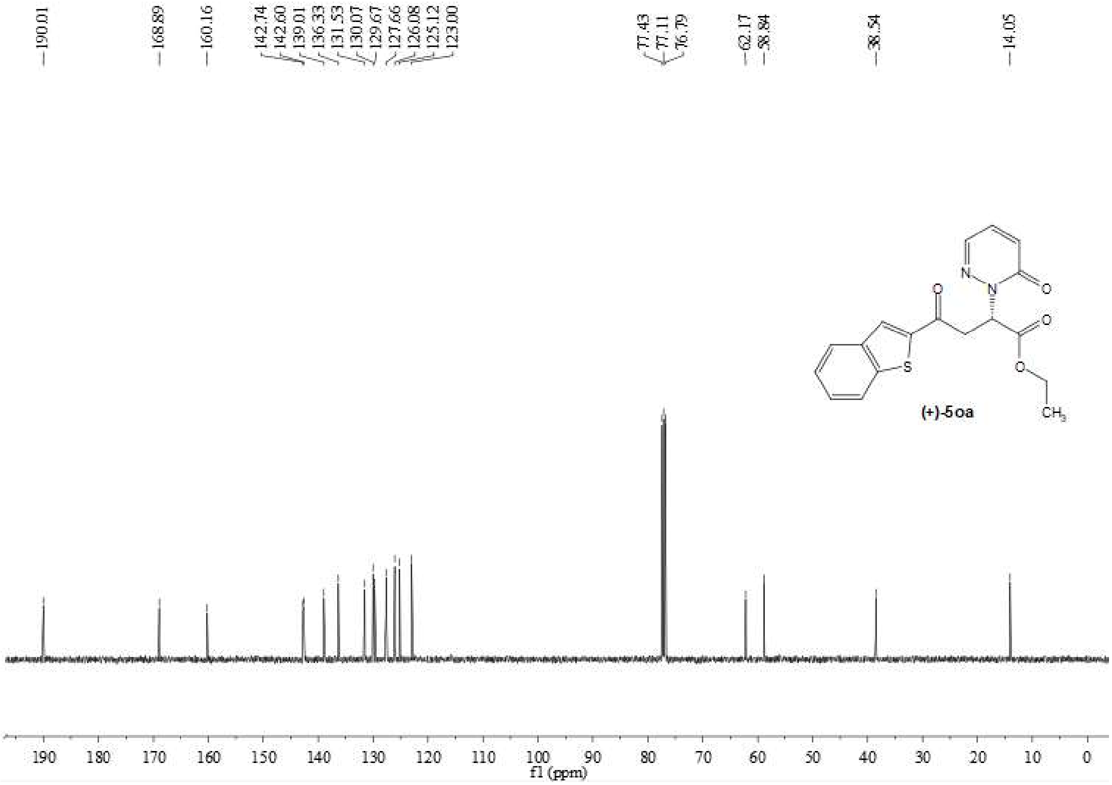
190.01
168.89
160.16
142.74
142.60
139.01
136.33
131.53
130.07
129.67
127.66
126.08
125.12
123.00
77.43
77.11
76.79
62.17
58.84
38.54
14.05
(+)-5oa
f1 (ppm)

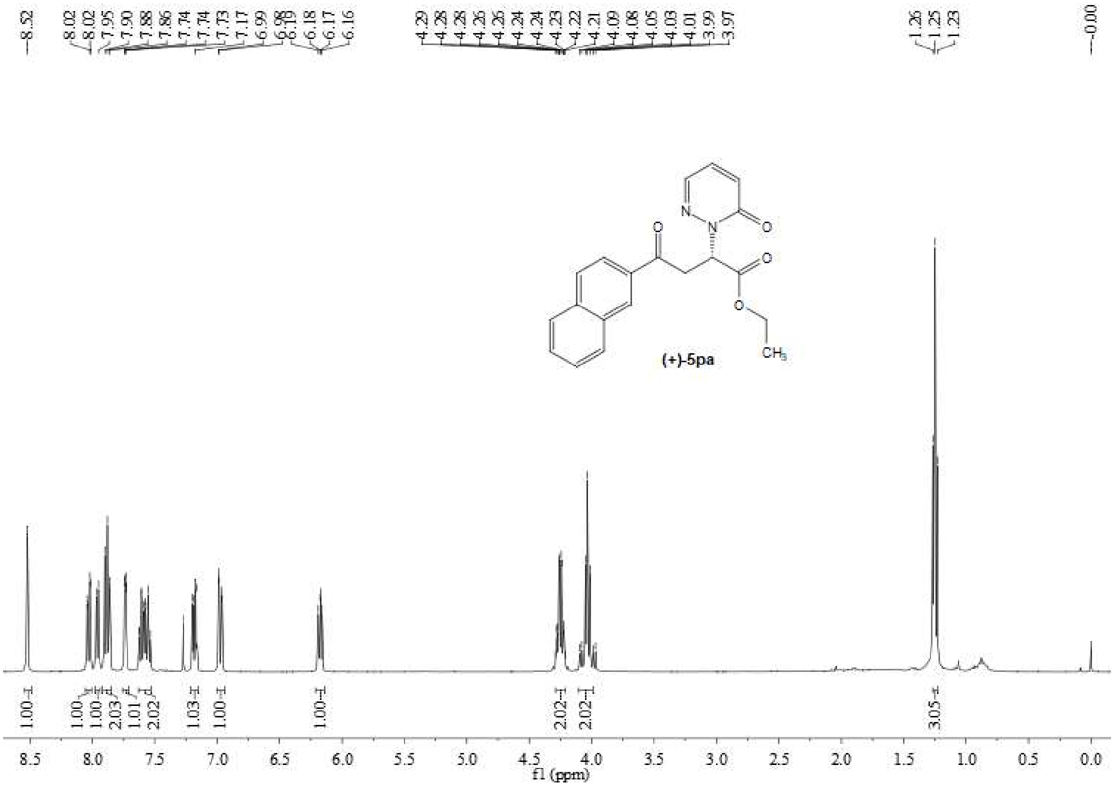
8.52
8.02
8.02
7.95
7.90
7.88
7.86
7.74
7.74
7.73
7.17
6.99
6.98
6.19
6.18
6.17
6.16
4.29
4.28
4.28
4.26
4.26
4.24
4.24
4.23
4.22
4.21
4.09
4.08
4.05
4.03
4.01
3.99
3.97
1.26
1.25
1.23
0.00
(+)-5pa
f1 (ppm)

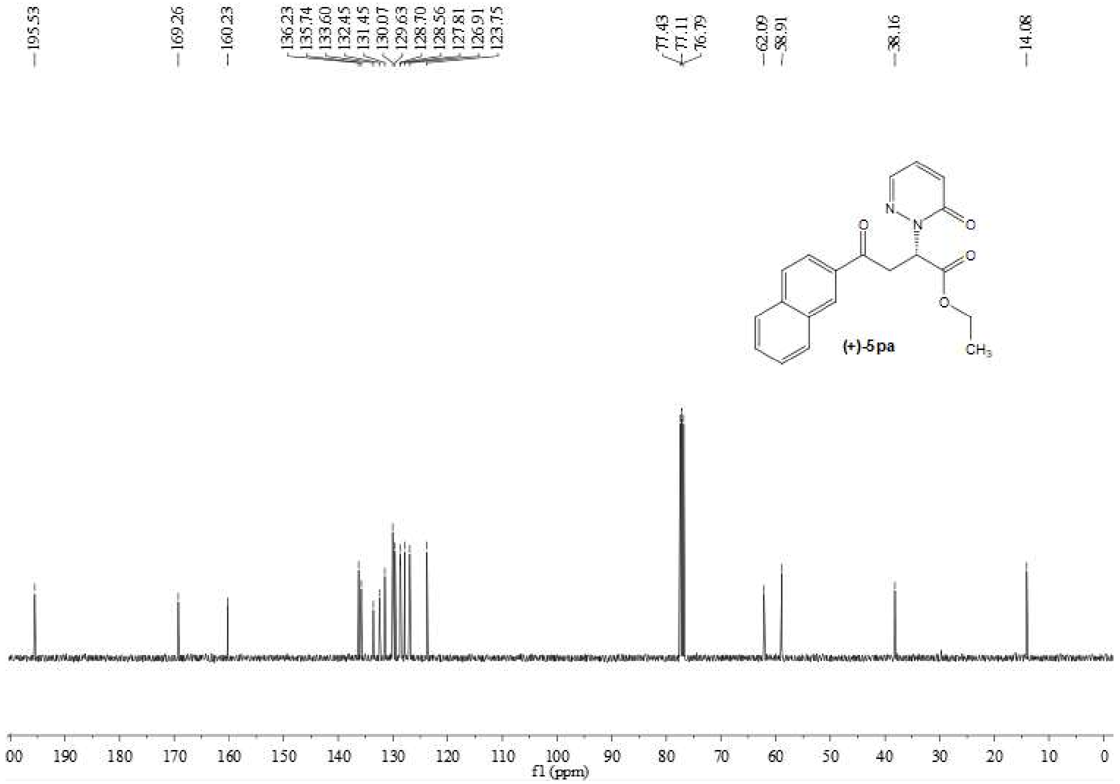
195.53
169.26
160.23
136.23
135.74
133.60
132.45
131.45
130.07
129.63
128.70
128.56
127.81
126.91
123.75
77.43
77.11
76.79
62.09
58.91
38.16
14.08
(+)-5pa
f1 (ppm)

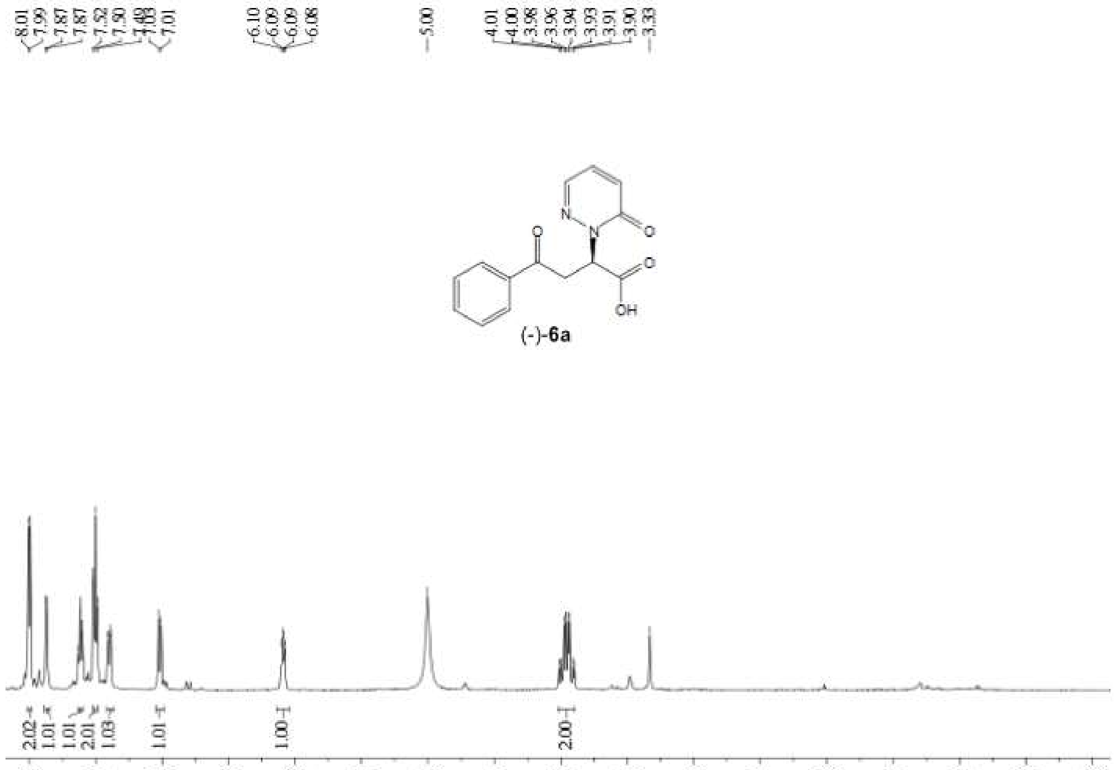
8.01
7.99
7.87
7.87
7.52
7.50
7.03
7.01
6.10
6.09
6.09
6.08
5.00
4.01
4.00
3.98
3.96
3.94
3.93
3.91
3.90
3.33
(-)-6a
2.02
1.01
1.01
2.01
1.03
1.01
1.00
2.00
f1 (ppm)

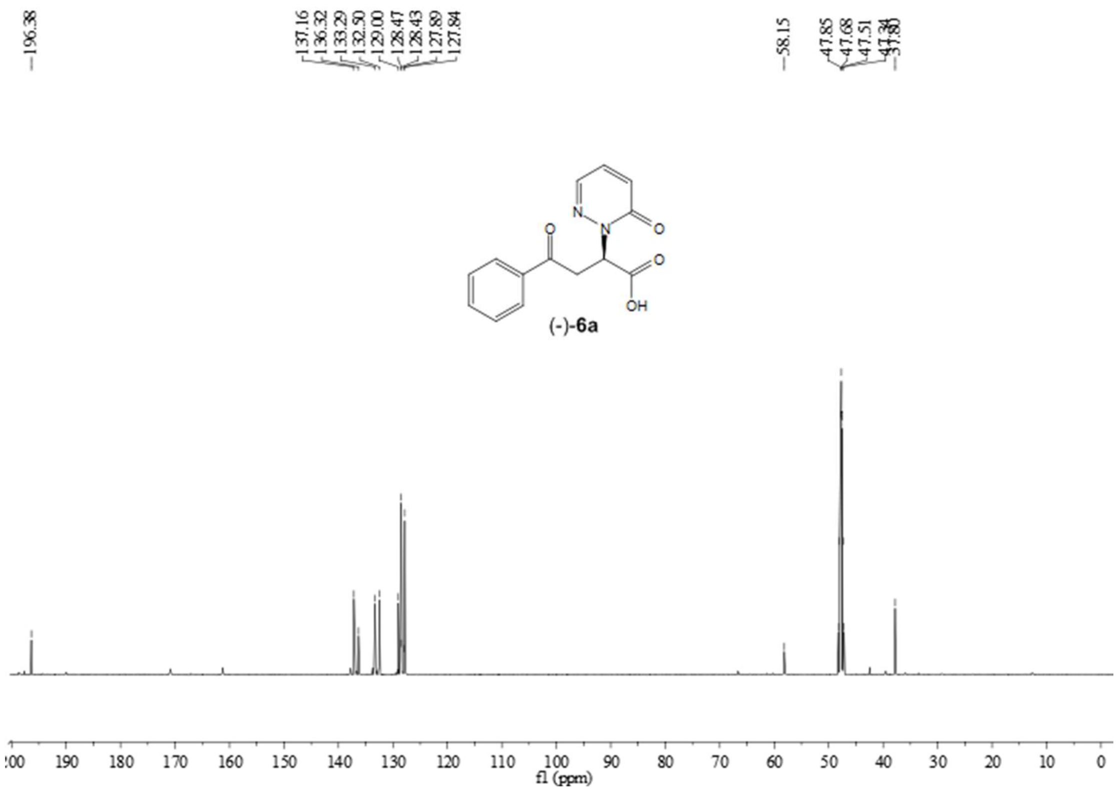

196.38
137.16
136.32
133.29
132.50
129.00
128.47
128.43
127.89
127.84
58.15
47.85
47.68
47.51
47.34
37.80
O
N
N
O
O
OH
(-)-6a
200 190 180 170 160 150 140 130 120 110 100 90 80 70 60 50 40 30 20 10 0
f1 (ppm)

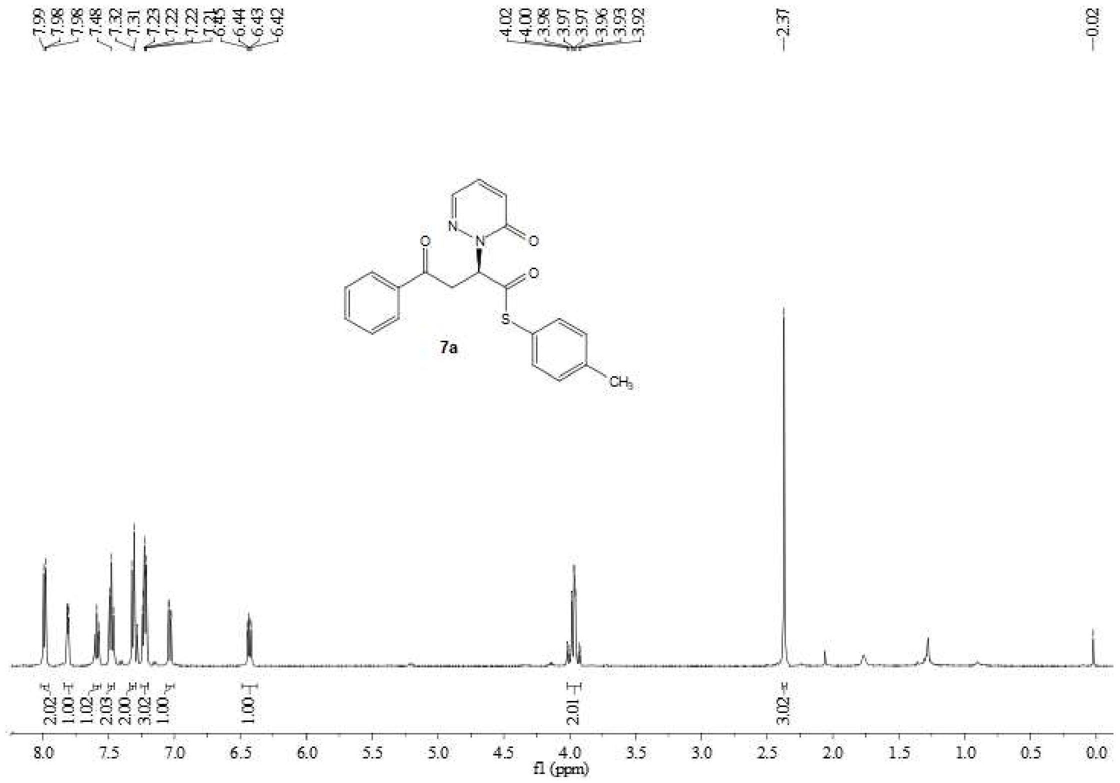

7.99
7.98
7.98
7.48
7.32
7.31
7.23
7.22
7.22
7.21
6.45
6.44
6.43
6.42
4.02
4.00
3.98
3.97
3.97
3.96
3.93
3.92
2.37
-0.02
O
N
N
O
O
S
7a
CH_3
2.02
1.00
1.02
2.03
2.00
3.02
1.00
1.00
2.01
3.02
8.0 7.5 7.0 6.5 6.0 5.5 5.0 4.5 4.0 3.5 3.0 2.5 2.0 1.5 1.0 0.5 0.0
f1 (ppm)

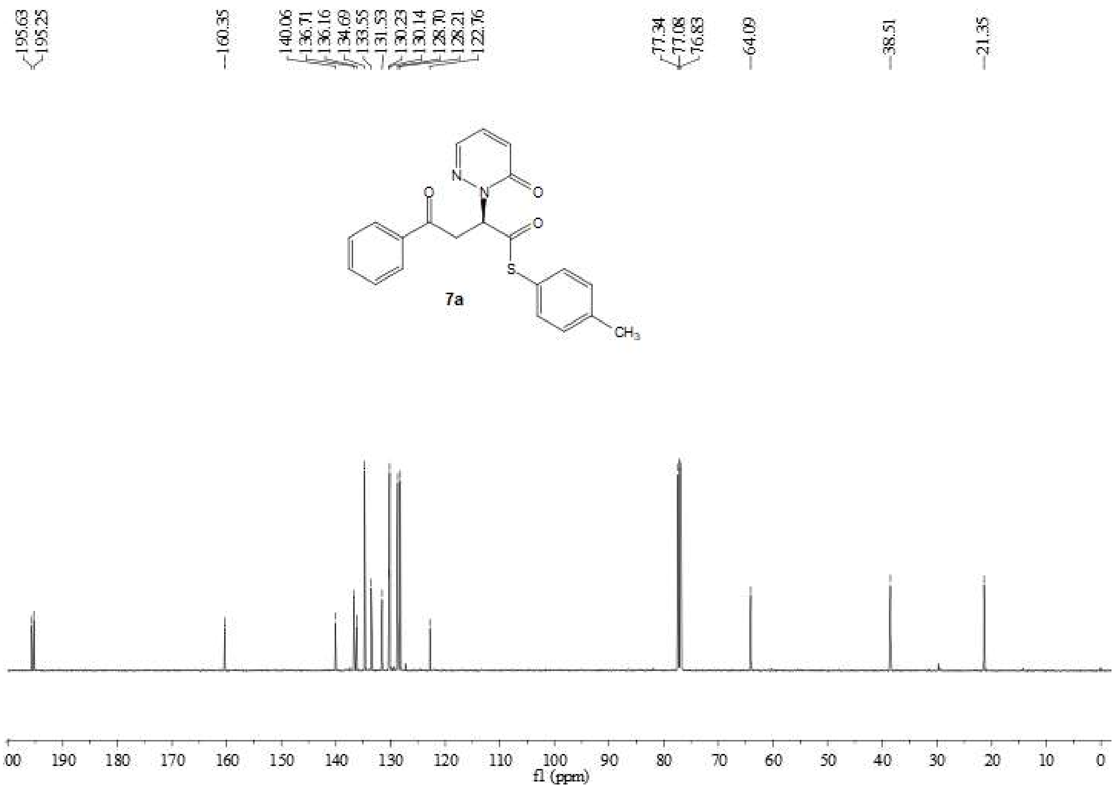

195.63
195.25
160.35
140.06
136.71
136.16
134.69
133.55
131.53
130.23
130.14
128.70
128.21
122.76
77.34
77.08
76.83
64.09
38.51
21.35
7a
f1 (ppm)

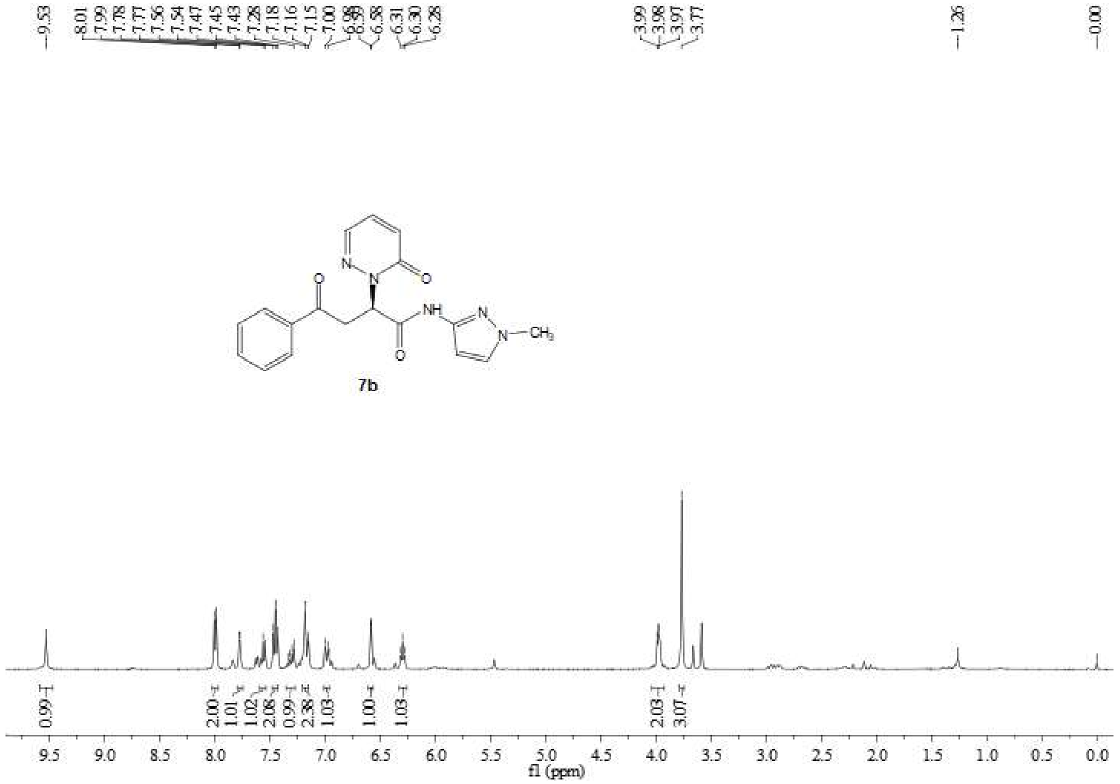

9.53
8.01
7.99
7.78
7.77
7.56
7.54
7.47
7.45
7.43
7.28
7.18
7.16
7.15
7.00
6.98
6.59
6.58
6.31
6.30
6.28
3.99
3.98
3.97
3.77
1.26
0.00
7b
0.99
2.00
1.01
1.02
2.08
0.99
2.38
1.03
1.00
1.03
2.03
3.07
f1 (ppm)

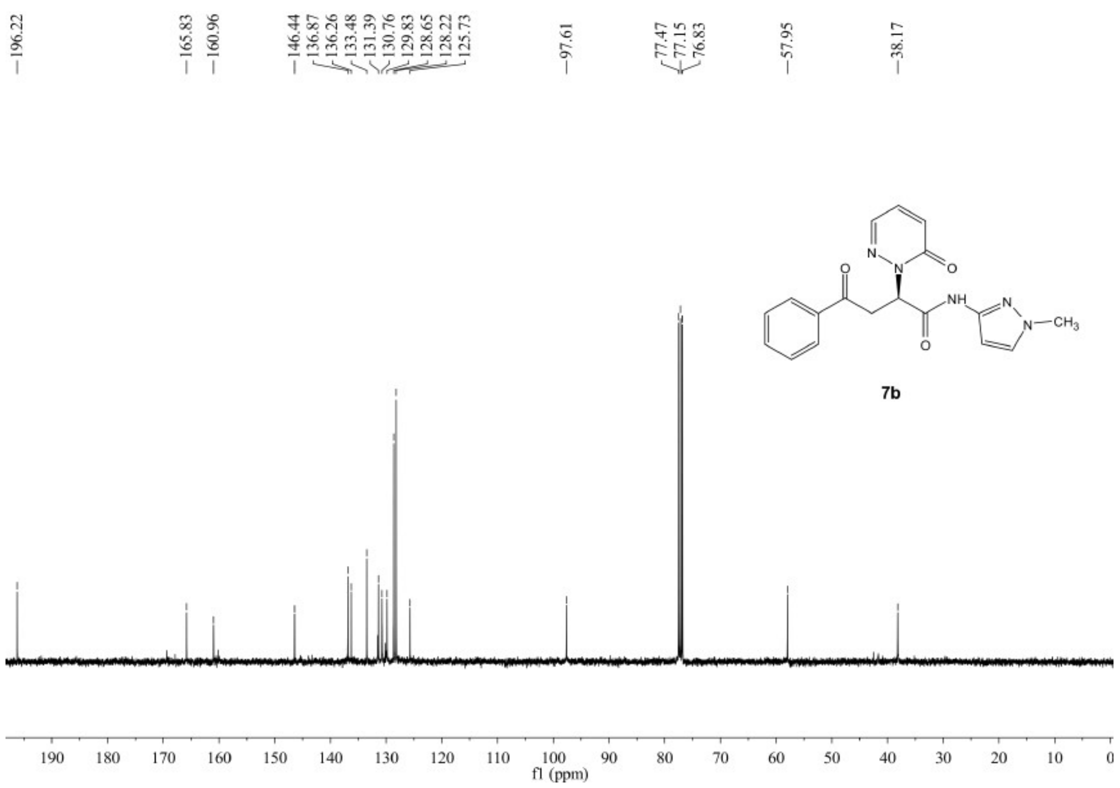

196.22
165.83
160.96
146.44
136.87
136.26
133.48
131.39
130.76
129.83
128.65
128.22
125.73
97.61
77.47
77.15
76.83
57.95
38.17
O
N
N
O
NH
N
N
CH3
O
7b
190 180 170 160 150 140 130 120 110 100 90 80 70 60 50 40 30 20 10 0
f1 (ppm)

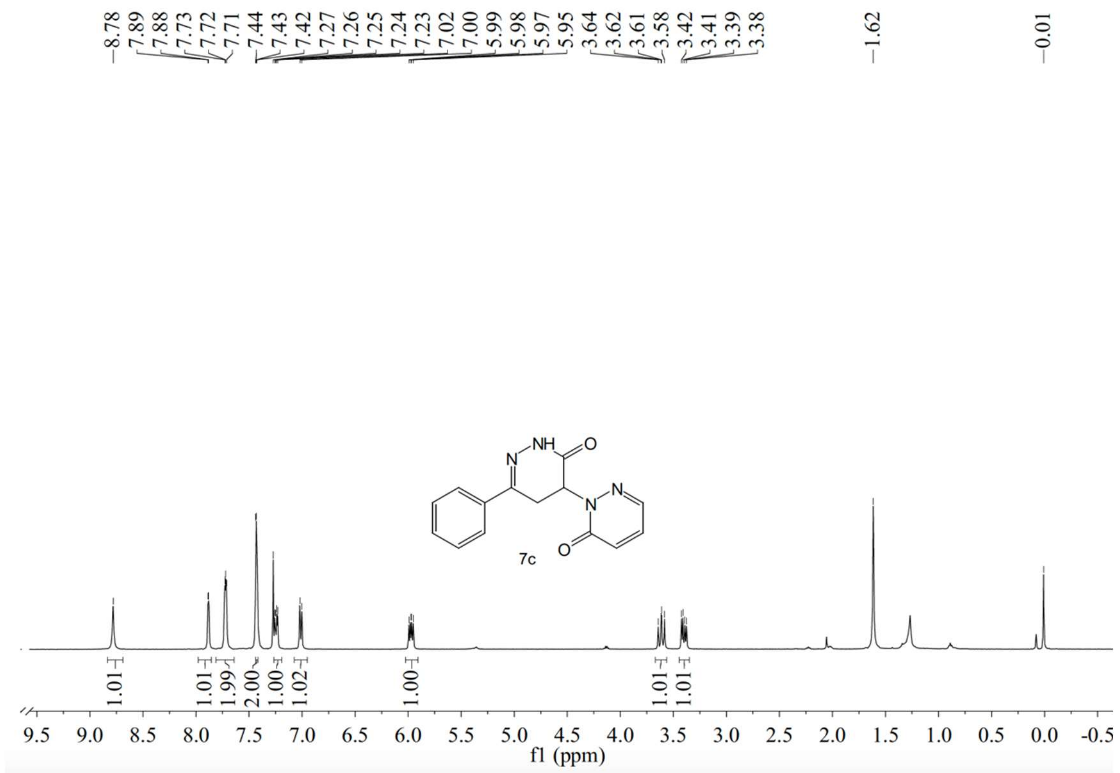

8.78
7.89
7.88
7.73
7.72
7.71
7.44
7.43
7.42
7.27
7.26
7.25
7.24
7.23
7.02
7.00
5.99
5.98
5.97
5.95
3.64
3.62
3.61
3.58
3.42
3.41
3.39
3.38
1.62
-0.01
N
NH
O
N
N
O
7c
1.01
1.01
1.99
2.00
1.00
1.02
1.00
1.01
1.01
9.5 9.0 8.5 8.0 7.5 7.0 6.5 6.0 5.5 5.0 4.5 4.0 3.5 3.0 2.5 2.0 1.5 1.0 0.5 0.0 -0.5
f1 (ppm)

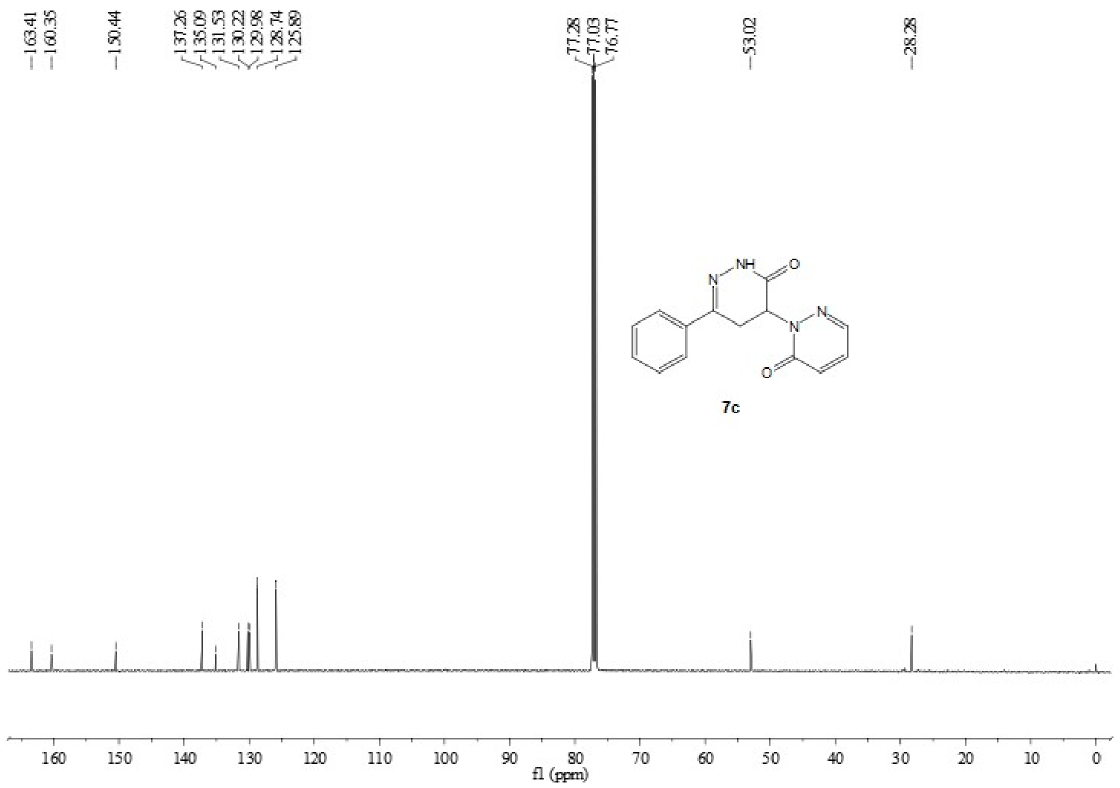
163.41
160.35
150.44
137.26
135.09
131.53
130.22
129.98
128.74
125.89
77.28
77.03
76.77
53.02
28.28
7c
f1 (ppm)

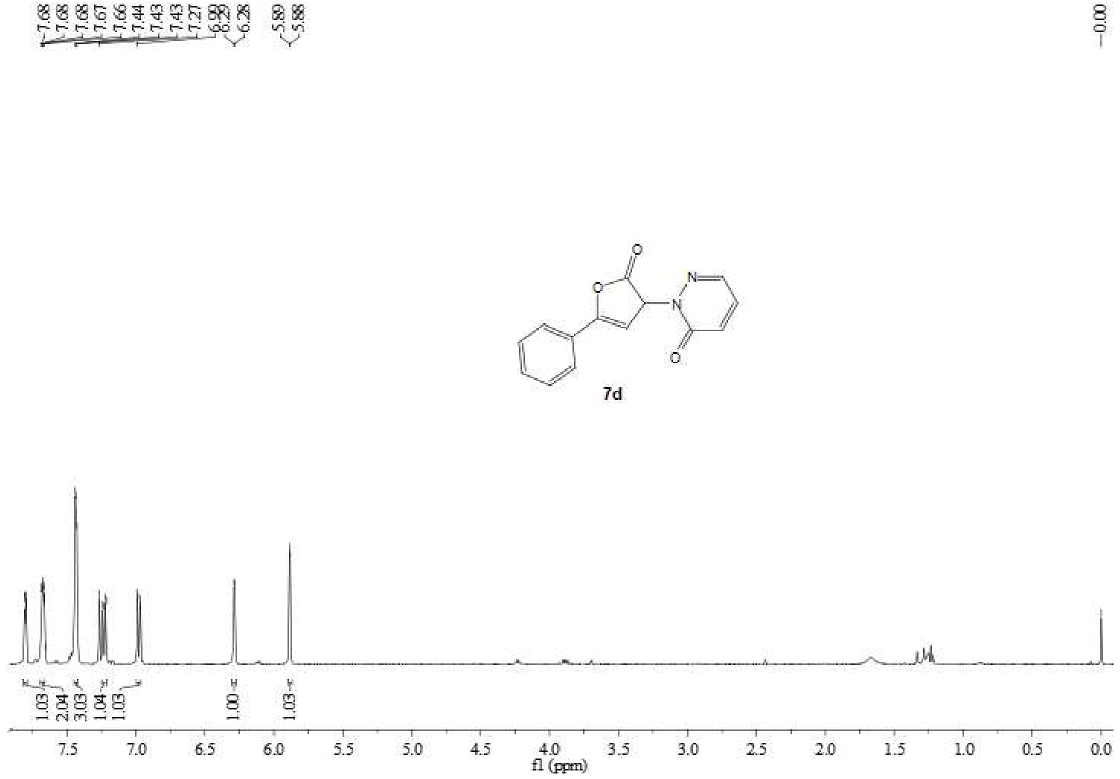
7.68
7.68
7.68
7.67
7.66
7.44
7.43
7.43
7.27
6.99
6.29
6.28
5.89
5.88
0.00
7d
1.03
2.04
3.03
1.04
1.03
1.00
1.03
f1 (ppm)

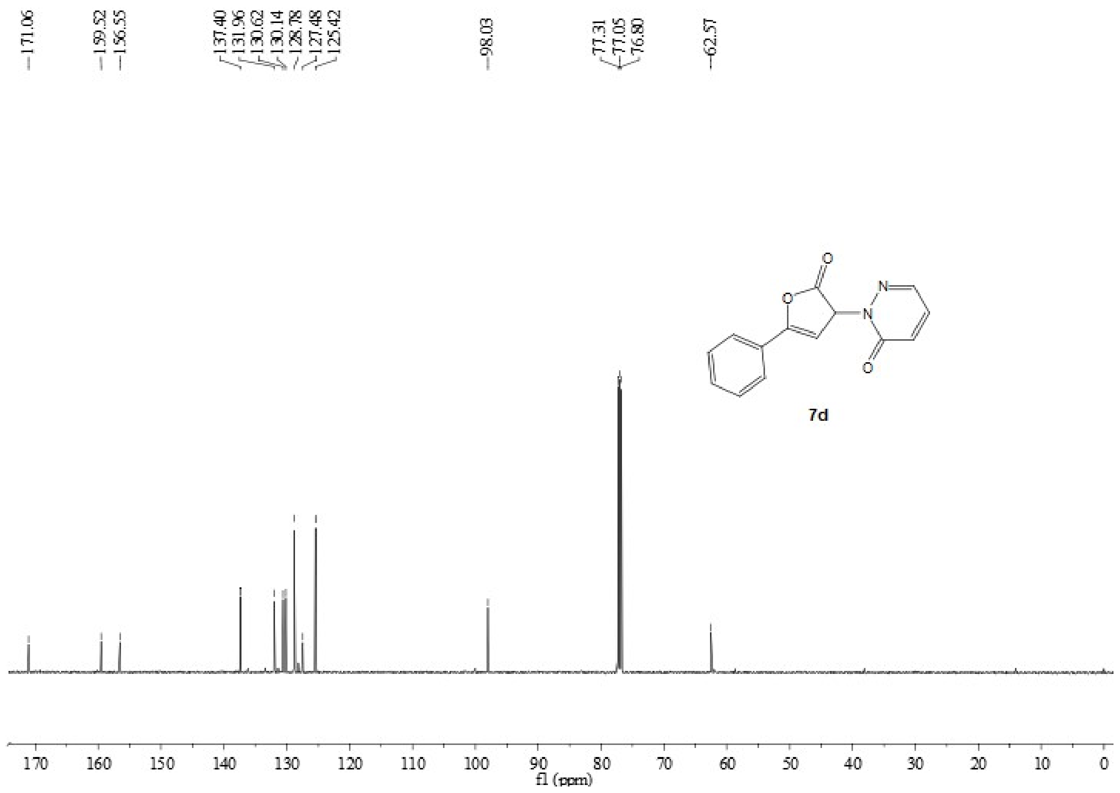

Supplemental References

Yamazaki, T., Kawasaki-Takasuka, T., Furuta, A. and Sakamoto, S. (2009). Facile conversion of 4, 4, 4-trifluorobut-2-yn-1-ols to 4, 4, 4-trifluorobut-2-en-1-ones. Tetrahedron, *65*, 5945-5948.

Daniel, D. S., Morrill, L. C., Yeh, P. P., Slawin, A. M. Z. O'Riordan, T. J. C. and Smith, A. D. (2013). Isothiourea‐Mediated One‐Pot Synthesis of Functionalized Pyridines. Angew. Chem. Int. Ed. *52*, 11642-11646.

Su, X., Zhou, W., Li, Y. and Zhang, J. (2015). Design, synthesis, and application of a chiral sulfinamide phosphine catalyst for the enantioselective intramolecular Rauhut–Currier reaction. Angew. Chem. Int. Ed. *54*, 6874-6877.

Zhou, W., Su, X., Tao, M., Zhu, C., Zhao, Q. and Zhang, J. (2015). Chiral Sulfinamide

Bisphosphine Catalysts: Design, Synthesis, and Application in Highly Enantioselective Intermolecular Cross‑Rauhut‑Currier Reactions. Angew. Chem. Int. Ed. *54*, 14853-14857.

Zhou, W., Chen, P., Tao, M., Su, X., Zhao, Q. and Zhang, J. (2016). Enantioselective intermolecular cross Rauhut–Currier reactions of activated alkenes with acrolein. Chem. Commun. *52*, 7612-7615.

Chen, P., Su, X., Zhou, W., Xiao, Y. and Zhang, J. (2016). Novel chiral sulfinamide phosphines: valuable precursors to chiral β-aminophosphines. Tetrahedron *72*, 2700-2706.

Wang, T., Yu, Z., Hoon, D., Phee, C., Lan, Y., and Lu, Y. (2015). Regiodivergent Enantioselective γ-Additions of Oxazolones to 2,3-Butadienoates Catalyzed by Phosphines: Synthesis of α,α-Disubstituted α-Amino Acids and N,O-Acetal Derivatives. J. Am. Chem. Soc. *138*, 265-271.

Wang, H., Zhou, W., Tao, M., Hu, A. and Zhang, J. (2017). Functionalized tetrahydropyridines by enantioselective phosphine-catalyzed aza-[4+ 2] cycloaddition of N-sulfonyl-1-aza-1, 3-dienes with vinyl ketones. Org. Lett. *19*, 1710-1713.
